# Supplementary material for: Determination of the instantaneous geostrophic flow within the three-dimensional magnetostrophic regime
Source: Proc Math Phys Eng Sci. 2018 Oct 3;474(2218):20180412. doi: 10.1098/rspa.2018.0412 (PMC6237501; doi:10.1098/rspa.2018.0412)
Supplement: ESM5 - ESM8 are PDF versions of the maple worksheets ESM1 - ESM4 respectively. [file rspa20180412supp2.pdf]

This worksheet solves the BWR equation analytically to determine the instantaneous geostrophic flow. The BWR equation is valid for all axisymmetric initial magnetic fields as well as purely toroidal non-axisymmetric fields.

```
> restart:
> Digits := 50:
> with(orthopoly, P):
  with(VectorCalculus):
  SetCoordinates(cartesian[x,y,z]):
```

# > # Define some useful routines to construct spherical harmonics

```
> # L1 is theta-factor in spherical harmonic (note that P(l,x) is
    the l-th Legendre polynomial)
> L1 := (l,m) -> if type(m, numeric) then if m <> 0 then sin(theta)
  ^abs(m) * subs(z=cos(theta), diff(P(l,z),z$abs(m))) else P(l, cos
  (theta)) end if else 'L1'(l,m) end if;
```

$L1 := (l, m) \rightarrow$ **if** $type(m, numeric)$ **then** **(1)**

$$\textbf{if } m \neq 0 \textbf{ then } \sin(\theta)^{|m|}\, subs\left(z=\cos(\theta), \frac{\partial^{|m|}}{\partial z^{|m|}} P(l,z)\right) \textbf{ else } P(l, \cos(\theta)) \textbf{ end if}$$

**else** $'L1'(l, m)$ **end if**

```
> # Lp is phi-factor
> Lp := m -> if type(m, numeric) then if m = 0 then 1 elif m < 0
  then sin(-m*phi) else cos(m*phi) end if else 'Lp'(m) end if;
```

$Lp := m \rightarrow$ **if** $type(m, numeric)$ **then** **(2)**

$$\textbf{if } m=0 \textbf{ then } 1 \textbf{ elif } m<0 \textbf{ then } \sin(VectorCalculus{:-}\text{`-`}(m\,\phi)) \textbf{ else } \cos(m\,\phi) \textbf{ end if}$$

**else** $'Lp'(m)$ **end if**

```
> # norm of L1*Lp is integral of (L1*Lp)^2 over sphere is int((L1*
  Lp)^2 * sin(theta), theta=0..Pi, phi=0..2*Pi)
> L2norm_squared := (l,m) -> int(L1(l,m)^2 * sin(theta), theta=0..
  Pi) * int(Lp(m)^2, phi=0..2*Pi) / (4*Pi);
```

$$L2norm\_squared := (l,m) \rightarrow VectorCalculus{:-}int\left(L1(l,m)^2 \sin(\theta), \theta=0..\pi\right) VectorCalculus{:-}int\left(Lp(m)^2, \phi=0..2\,\pi\right) \frac{1}{4\,\pi} \quad \textbf{(3)}$$

```
> # L2 is Schmidt quasi-normalised spherical harmonic
> L2 := (l,m) -> if type(l,numeric) and type(m,numeric) then L1(l,
  m) * Lp(m) / sqrt(L2norm_squared(l,m)) / sqrt(2*l+1) else 'L2'(l,
  m) end if;
```

$L2 := (l, m) \rightarrow$ **if** $type(l, numeric)$ **and** $type(m, numeric)$ **then** **(4)**

$$L1(l,m)\, Lp(m) \frac{1}{\sqrt{L2norm\_squared(l,m)}} \frac{1}{\sqrt{2\,l+1}}$$

**else** $'L2'(l, m)$ **end if**

```
> # Convert an expression in spherical coordinates to Cartesian coordinates.
> sph2cart := proc(expr)
    local res;
    res := expand(expr, trig);
    res := subs(cos(phi) = x/(r*sin(theta)), sin(phi) = y/(r*sin(theta)), res);
    res := subs(cos(theta) = z/r, sin(theta) = sqrt(x^2+y^2)/r, res);
    res := subs(r = sqrt(x^2+y^2+z^2), res);
    return simplify(res)
  end proc:
> # Construct vector field from poloidal and toroidal scalars
  scalars2vf := proc(tor_scalar, pol_scalar)
    return simplify(Curl(VectorField(sph2cart(tor_scalar/r) * <x,y,z>))
                    + Curl(Curl(VectorField(sph2cart(pol_scalar/r) * <x,y,z>))))
  end proc:
> # Define basis functions for the poloidal flow which vanish at r=1. Degree is l+2n-1.
> Chi_n := (l,n) -> r^(l+1) * (1-r^2) * P(n-1,3/2,l+1/2,2*r^2-1): # curl of this is basis for poloidal part
> W_n := (l,n) -> r^(l+1) * P(n,-1/2,l+1/2,2*r^2-1): # curl^2 of this is basis for toroidal part
> Psi_n := proc (l, n) options operator, arrow; r^(l+1)*((-2*n^2*(l+1)-n*(l+1)*(2*l-1)-l*(2*l+1))*P(n, 0, l+1/2, 2*r^2-1)+((2*l+2)*n^2+(2*l+3)*(l+1)*n+(2*l+1)^2)*P(n-1, 0, l+1/2, 2*r^2-1)+4*n*l+l*(2*l+1))end proc
```

$$Psi\_n := (l, n) \to r^{l+1} \Big( \big( VectorCalculus{:-}\text{`-`}(2\, n^2\, (l+1)) + VectorCalculus{:-}\text{`-`}(n\, (l+1)\, (2\, l + (-1))) + VectorCalculus{:-}\text{`-`}(l\, (2\, l+1)) \big)\, P\Big(n, 0, l+1\frac{1}{2}, 2\, r^2 + (-1)\Big) + \big((2\, l+2)\, n^2 + (2\, l+3)\, (l+1)\, n + (2\, l+1)^2\big)\, P\Big(n+(-1), 0, l+1\frac{1}{2}, 2\, r^2 + (-1)\Big) + 4\, n\, l + l\, (2\, l+1) \Big) \quad \textbf{(5)}$$

## > # Define magnetic field

```
> # choose a number corresponding to the chosen magnetic field 1=axisymmetric poloidal, 2=nonaxisymmetric toroidal, 3=nonaxisymmetric poloidal, 4=nonaxisymmetric mixed state
> k := 1;
```

$$k := 1 \quad \textbf{(6)}$$

```
> if k=1 then B_scalar_tor := 0 : B_scalar_pol := eval( r^2 * (30 * r^4 - 57 * r^2 + 25) * L2(l, m), {l=1, m=0, n=1})
  end if
```

$$B\_scalar\_tor := 0$$

$$B\_scalar\_pol := r^2\,(30\, r^4 - 57\, r^2 + 25)\,\cos(\theta) \quad \textbf{(7)}$$

```
> if k=2 then B_scalar_tor := simplify(eval(Chi_n(l, n)·L2(l, m), {l=1, m=1, n=1})) :
      B_scalar_pol := 0
  end if
> if k=3 then B_scalar_tor := 0 : B_scalar_pol := eval(Psi_n(l, n)·L2(l, m), {l=2, m=2, n
      =1})
  end
> if k=4 then B_scalar_tor := eval(Chi_n(l, n)·L2(l, m), {l=2, m=1, n=1}) :
      B_scalar_pol := eval(Psi_n(l, n)·L2(l, m), {l=2, m=1, n=1})
  end if
```

```
> B_cart_pol := scalars2vf(0, B_scalar_pol);
```

$$B\_cart\_pol := (-120x^3z - 120xy^2z - 120xz^3 + 114xz)\bar{e}_x + (-120x^2yz - 120y^3z - 120yz^3 + 114yz)\bar{e}_y + (180x^4 + 360x^2y^2 + 240x^2z^2 + 180y^4 + 240y^2z^2 + 60z^4 - 228x^2 - 228y^2 - 114z^2 + 50)\bar{e}_z \quad (8)$$

```
> B_cart_tor := scalars2vf(B_scalar_tor, 0);
```

$$B\_cart\_tor := 0\bar{e}_x \quad (9)$$

```
> B_sph_pol := simplify(MapToBasis(B_cart_pol, spherical[r, theta, phi]));
```

$$B\_sph\_pol := ((60r^4 - 114r^2 + 50)\cos(\theta))\bar{e}_r + ((-180r^4 + 228r^2 - 50)\sin(\theta))\bar{e}_\theta \quad (10)$$

```
> B_sph_tor := simplify(MapToBasis(B_cart_tor, spherical[r, theta, phi]));
```

$$B\_sph\_tor := 0\bar{e}_r \quad (11)$$

```
> SetCoordinates(spherical[r, theta, phi]);
```

$$spherical_{r,\theta,\phi} \quad (12)$$

```
> #Scale the magnetic field
> Scale_pol := sqrt(1/(4·Pi) · int(int(int(A_pol·B_sph_pol.B_sph_pol·r^2·sin(theta), phi=0..2
      ·Pi), theta=0..Pi), r=0..1));
```

$$Scale\_pol := \frac{2}{231}\sqrt{1188726}\sqrt{A\_pol} \quad (13)$$

```
> if Scale_pol=0 then A_pol=0
  else A_pol := simplify(solve(Scale_pol=1, A_pol)); end if;
```

$$A\_pol := \frac{231}{20584} \quad (14)$$

```
> Scale_tor := sqrt(1/(4·Pi) · int(int(int(A_tor·B_sph_tor.B_sph_tor·r^2·sin(theta), phi=0..2
      ·Pi), theta=0..Pi), r=0..1));
```

$$Scale\_tor := 0 \quad (15)$$

> **if** *Scale_tor* = 0 **then** *A_tor* = 0
> **else** *A_tor* := *simplify*(*solve*(*Scale_tor* = 1, *A_tor*)) **end if**;

$$A\_tor = 0 \quad (16)$$

> *SetCoordinates*(*cartesian*[*x*, *y*, *z*]) :

> *B_cart* := *scalars2vf*(sqrt(*A_tor*) · *B_scalar_tor*, sqrt(*A_pol*) · *B_scalar_pol*);

$$B\_cart := \left(-\frac{30}{2573}\sqrt{1188726}\,x\,y^2\,z - \frac{30}{2573}\sqrt{1188726}\,x^3\,z - \frac{30}{2573}\sqrt{1188726}\,x\,z^3 + \frac{57}{5146}\sqrt{1188726}\,x\,z\right)\bar{e}_x + \left(-\frac{30}{2573}\sqrt{1188726}\,x^2\,y\,z - \frac{30}{2573}\sqrt{1188726}\,y^3\,z - \frac{30}{2573}\sqrt{1188726}\,y\,z^3 + \frac{57}{5146}\sqrt{1188726}\,y\,z\right)\bar{e}_y + \left(\frac{25}{5146}\sqrt{1188726} + \frac{90}{2573}\sqrt{1188726}\,x^2\,y^2 + \frac{60}{2573}\sqrt{1188726}\,x^2\,z^2 + \frac{60}{2573}\sqrt{1188726}\,y^2\,z^2 + \frac{45}{2573}\sqrt{1188726}\,x^4 + \frac{45}{2573}\sqrt{1188726}\,y^4 + \frac{15}{2573}\sqrt{1188726}\,z^4 - \frac{57}{2573}\sqrt{1188726}\,x^2 - \frac{57}{2573}\sqrt{1188726}\,y^2 - \frac{57}{5146}\sqrt{1188726}\,z^2\right)\bar{e}_z \quad (17)$$

> **B_sph := simplify(MapToBasis(B_cart, spherical[r,theta,phi]));**

$$B\_sph := \frac{1}{5146}\cos(\theta)\sqrt{1188726}\,(30\,r^4 - 57\,r^2 + 25)\bar{e}_r - \frac{1}{5146}\sin(\theta)\sqrt{1188726}\,(90\,r^4 - 114\,r^2 + 25)\bar{e}_\theta \quad (18)$$

# > # Compute rhs of magnetostrophic equation

> **# slaved equation is Omega cross u = -div(p) + curl(B) cross B, we ignore the pressure**

> **RHS := CrossProduct(Curl(B_cart), B_cart): simplify(RHS);**

$$-\frac{3465}{5146}\,x\,(28\,x^2 + 28\,y^2 + 28\,z^2 - 19)\,(90\,x^4 + 180\,x^2\,y^2 + 120\,x^2\,z^2 + 90\,y^4 + 120\,y^2\,z^2 + 30\,z^4 - 114\,x^2 - 114\,y^2 - 57\,z^2 + 25)\bar{e}_x - \frac{3465}{5146}\,y\,(28\,x^2 + 28\,y^2 + 28\,z^2 - 19)\,(90\,x^4 + 180\,x^2\,y^2 + 120\,x^2\,z^2 + 90\,y^4 + 120\,y^2\,z^2 + 30\,z^4 - 114\,x^2 - 114\,y^2 - 57\,z^2 + 25)\bar{e}_y - \frac{2910600}{2573}\,z\left(x^2 + y^2 + z^2 - \frac{19}{20}\right)(x^2 + y^2)\left(x^2 + y^2 + z^2 - \frac{19}{28}\right)\bar{e}_z \quad (19)$$

```
> map(factor, simplify(MapToBasis(RHS, spherical[r,theta,phi]))); # for comparison
```

$$-\frac{3465}{5146}\sin(\theta)^2(28r^2-19)(90r^4-114r^2+25)\, r\bar{e}_r - \frac{3465}{5146}\cos(\theta)\, r\sin(\theta)(28r^2-19)(30r^4-57r^2+25)\bar{e}_\theta \quad (20)$$

# > # Construct basis for u

```
> # Let N be the degree of B. Then the degree of curl(B) cross B is (N-1) + N = 2N-1. Thus the degree of u is also 2N-1. Thus, the poloidal scalar has degree 2N+1 (we have to undo two curls) and the toroidal scalar has degree 2N. Furthermore, the m-degree of curl(B) cross B is twice the m-degree of B.
> B_degree := max(seq(degree(B_cart[idx], {x,y,z}), idx = 1 .. 3));
```

$$B\_degree := 4 \quad (21)$$

```
> Max_pol_degree := 2 * B_degree + 1; Max_tor_degree := 2 * B_degree;
```

$$Max\_pol\_degree := 9$$
$$Max\_tor\_degree := 8 \quad (22)$$

```
> Max_m_degree := 2 * max(seq(degree(expand(B_sph[idx], trig), {cos(phi), sin(phi)}), idx = 1 .. 3));
```

$$Max\_m\_degree := 0 \quad (23)$$

```
> # For the particular example here, certain modes are zero, but we will not exploit this knowledge.
> # Also note that unless B is a Taylor state, there will be no solution to the magnetostrophic equation.

> u_scalar_pol := add(add(add(S[l,m,n] * L2(l,m) * Chi_n(l,n),
                          m = -min(l, Max_m_degree) .. min(l, Max_m_degree)),
                          n = 1 .. (Max_pol_degree - l + 1) / 2),
                          l = 1 .. Max_pol_degree):
> u_scalar_tor := add(add(add(T[l,m,n] * L2(l,m) * W_n(l,n),
                          m = -min(l, Max_m_degree) .. min(l, Max_m_degree)),
                          n = 0 .. floor((Max_tor_degree - l + 1)/2)),
                          l = 1 .. Max_tor_degree):
> coeff(u_scalar_pol, S[2,-2,2]);
```

$$0 \quad (24)$$

```
> # u is poloidal part (curl^2 of scalar times hat r) + toroidal part (curl of scalar times hat r)
> u_cart := scalars2vf(u_scalar_tor, u_scalar_pol):
> variables := indets(u_cart, indexed);
```

$$variables := \{S_{1,0,1}, S_{1,0,2}, S_{1,0,3}, S_{1,0,4}, S_{2,0,1}, S_{2,0,2}, S_{2,0,3}, S_{2,0,4}, S_{3,0,1}, S_{3,0,2}, S_{3,0,3}, S_{4,0,1}, S_{4,0,2}, S_{4,0,3}, S_{5,0,1}, S_{5,0,2}, S_{6,0,1}, S_{6,0,2}, S_{7,0,1}, S_{8,0,1}, T_{1,0,0}, T_{1,0,1}, T_{1,0,2}, T_{1,0,3}, T_{1,0,4}, T_{2,0,0}, T_{2,0,1}, T_{2,0,2}, T_{2,0,3}, T_{3,0,0}, T_{3,0,1}, T_{3,0,2}, T_{3,0,3}, T_{4,0,0}, T_{4,0,1}, T_{4,0,2}, T_{5,0,0}, T_{5,0,1}, T_{5,0,2}, T_{6,0,0}, T_{6,0,1}, T_{7,0,0}, T_{7,0,1}, T_{8,0,0}\} \quad (25)$$

## > # Compute lhs of magnetostrophic equation

```
> # slaved equation is Omega cross u = -div(p) + curl(B) cross B
> Omega_vec := VectorField([0,0,1]);  # rotation vector in cartesian coordinates
```

$$Omega\_vec := \bar{e}_z \tag{26}$$

```
> LHS := CrossProduct(Omega_vec, u_cart):
```

## > # Solve magnetostrophic equation for basis coefficients

```
> # take the curl of slaved equation; pressure drops out
> eqn := simplify(Curl(LHS - RHS)):
> constraints := `union`(seq({coeffs(collect(eqn[i], [x,y,z], distributed), [x,y,z])}, i=1..3)):
> nops(constraints); nops(variables);
```

$$113$$
$$44 \tag{27}$$

```
> sol1 := solve(constraints, variables);
```

$$sol1 := \Big\{S_{1,0,1}=0, S_{1,0,2}=0, S_{1,0,3}=0, S_{1,0,4}=0, S_{2,0,1}=0, S_{2,0,2}=0, S_{2,0,3}=0, S_{2,0,4}=0, S_{3,0,1}=0, S_{3,0,2}=0, S_{3,0,3}=0, S_{4,0,1}=0, S_{4,0,2}=0, S_{4,0,3}=0, S_{5,0,1}=0, S_{5,0,2}=0, S_{6,0,1}=0, S_{6,0,2}=0, S_{7,0,1}=0, S_{8,0,1}=0, T_{1,0,0}=T_{1,0,0}, T_{1,0,1}=\frac{27489}{5146}-2\,T_{3,0,0}, T_{1,0,2}=-\frac{89397}{5146}-\frac{27}{14}\,T_{3,0,1}, T_{1,0,3}=\frac{13860}{2573}-\frac{66}{35}\,T_{3,0,2}, T_{1,0,4}=0, T_{2,0,0}=0, T_{2,0,1}=0, T_{2,0,2}=0, T_{2,0,3}=0, T_{3,0,0}=T_{3,0,0}, T_{3,0,1}=T_{3,0,1}, T_{3,0,2}=T_{3,0,2}, T_{3,0,3}=0, T_{4,0,0}=0, T_{4,0,1}=0, T_{4,0,2}=0, T_{5,0,0}=-\frac{5}{7}\,T_{3,0,1}, T_{5,0,1}=-\frac{66}{91}\,T_{3,0,2}, T_{5,0,2}=0, T_{6,0,0}=0, T_{6,0,1}=0, T_{7,0,0}=\frac{15}{26}\,T_{3,0,2}, T_{7,0,1}=0, T_{8,0,0}=0\Big\} \tag{28}$$

## > # set geostrophic component to zero to remove degeneracy

```
> u_soln := simplify(subs(sol1, u_cart));
```

$$u\_soln := \frac{3465}{32}\left(\left(-\frac{4480}{2573}+T_{3,0,2}\right)y^6+\left(\left(-\frac{13440}{2573}+3\,T_{3,0,2}\right)x^2+\frac{12768}{2573}-\frac{13440}{2573}\,z^2+\frac{20}{77}\,T_{3,0,1}-\frac{12}{7}\,T_{3,0,2}\right)y^4+\left(\left(-\frac{13440}{2573}+3\,T_{3,0,2}\right)x^4+\left(-\frac{26880}{2573}\,z^2+\frac{25536}{2573}-\frac{24}{7}\,T_{3,0,2}+\frac{40}{77}\,T_{3,0,1}\right)x^2-\frac{11200}{2573}+\frac{25536}{2573}\,z^2-\frac{13440}{2573}\,z^4 \tag{29}$$

$$+\frac{16}{231}T_{3,0,0}-\frac{24}{77}T_{3,0,1}+\frac{6}{7}T_{3,0,2}\Big)y^2+\Big(-\frac{4480}{2573}+T_{3,0,2}\Big)x^6+\Big(\frac{12768}{2573}$$
$$-\frac{13440}{2573}z^2+\frac{20}{77}T_{3,0,1}-\frac{12}{7}T_{3,0,2}\Big)x^4+\Big(-\frac{11200}{2573}+\frac{25536}{2573}z^2-\frac{13440}{2573}z^4$$
$$+\frac{16}{231}T_{3,0,0}-\frac{24}{77}T_{3,0,1}+\frac{6}{7}T_{3,0,2}\Big)x^2+\frac{8890}{7719}-\frac{4480}{2573}z^6-\frac{32}{3465}T_{1,0,0}$$
$$+\frac{12768}{2573}z^4-\frac{11200}{2573}z^2-\frac{32}{693}T_{3,0,0}+\frac{6}{77}T_{3,0,1}-\frac{4}{35}T_{3,0,2}\Big)y\bar{e}_x-\frac{3465}{32}\Big(\Big($$
$$-\frac{4480}{2573}+T_{3,0,2}\Big)x^6+\Big(\Big(-\frac{13440}{2573}+3\,T_{3,0,2}\Big)y^2+\frac{12768}{2573}-\frac{13440}{2573}z^2+\frac{20}{77}T_{3,0,1}$$
$$-\frac{12}{7}T_{3,0,2}\Big)x^4+\Big(\Big(-\frac{13440}{2573}+3\,T_{3,0,2}\Big)y^4+\Big(-\frac{26880}{2573}z^2+\frac{25536}{2573}-\frac{24}{7}T_{3,0,2}$$
$$+\frac{40}{77}T_{3,0,1}\Big)y^2-\frac{11200}{2573}+\frac{25536}{2573}z^2-\frac{13440}{2573}z^4+\frac{16}{231}T_{3,0,0}-\frac{24}{77}T_{3,0,1}$$
$$+\frac{6}{7}T_{3,0,2}\Big)x^2+\Big(-\frac{4480}{2573}+T_{3,0,2}\Big)y^6+\Big(\frac{12768}{2573}-\frac{13440}{2573}z^2+\frac{20}{77}T_{3,0,1}$$
$$-\frac{12}{7}T_{3,0,2}\Big)y^4+\Big(-\frac{11200}{2573}+\frac{25536}{2573}z^2-\frac{13440}{2573}z^4+\frac{16}{231}T_{3,0,0}-\frac{24}{77}T_{3,0,1}$$
$$+\frac{6}{7}T_{3,0,2}\Big)y^2+\frac{8890}{7719}-\frac{4480}{2573}z^6-\frac{32}{3465}T_{1,0,0}+\frac{12768}{2573}z^4-\frac{11200}{2573}z^2$$
$$-\frac{32}{693}T_{3,0,0}+\frac{6}{77}T_{3,0,1}-\frac{4}{35}T_{3,0,2}\Big)x\bar{e}_y$$

```
> u_cyl := simplify(MapToBasis(u_soln, cylindrical[s,phi,z]));
```

$$u\_cyl := -\frac{3465}{32}s\Big(\Big(\Big(-\frac{4480}{2573}+T_{3,0,2}\Big)s^6+\Big(\frac{12768}{2573}-\frac{13440}{2573}z^2+\frac{20}{77}T_{3,0,1} \qquad \textbf{(30)}$$
$$-\frac{12}{7}T_{3,0,2}\Big)s^4+\Big(-\frac{11200}{2573}+\frac{25536}{2573}z^2-\frac{13440}{2573}z^4+\frac{16}{231}T_{3,0,0}-\frac{24}{77}T_{3,0,1}$$
$$+\frac{6}{7}T_{3,0,2}\Big)s^2-\frac{4480}{2573}z^6+\frac{12768}{2573}z^4-\frac{11200}{2573}z^2-\frac{32}{3465}T_{1,0,0}-\frac{32}{693}T_{3,0,0}$$
$$+\frac{6}{77}T_{3,0,1}-\frac{4}{35}T_{3,0,2}+\frac{8890}{7719}\Big)\bar{e}_\phi$$

> $SetCoordinates(cylindrical[s, \mathrm{phi}, z])$

$$cylindrical_{s,\phi,z} \qquad \textbf{(31)}$$

> $geostrophic\_component := simplify(int(\ int(\ u\_cyl[2], \mathrm{phi}=0..2*\mathrm{Pi}), z=-\mathrm{sqrt}(1-s\text{^}2)..\mathrm{sqrt}(1-s\text{^}2))\ /\ (4*\mathrm{Pi}*\mathrm{sqrt}(1-s\text{^}2)));$

$$geostrophic\_component := -\frac{3465}{32}\Big(\Big(T_{3,0,2}-\frac{2048}{2573}\Big)s^6+\Big(\frac{20}{77}T_{3,0,1}-\frac{12}{7}T_{3,0,2} \qquad \textbf{(32)}$$
$$+\frac{28928}{12865}\Big)s^4+\Big(\frac{16}{231}T_{3,0,0}-\frac{24}{77}T_{3,0,1}+\frac{6}{7}T_{3,0,2}-\frac{72448}{38595}\Big)s^2-\frac{32}{3465}T_{1,0,0}$$
$$-\frac{32}{693}T_{3,0,0}+\frac{6}{77}T_{3,0,1}-\frac{4}{35}T_{3,0,2}+\frac{5718}{12865}\Big)s$$

```
> u_cyl := simplify(u_cyl - VectorField([0, geostrophic_component, 0])); #remove geostrophic component from u_cyl
```

$$u\_cyl := \frac{231}{2573}\, s\, (1140\, s^6 + 6300\, s^4 z^2 + 6300\, s^2 z^4 + 2100\, z^6 - 3273\, s^4 - 11970\, s^2 z^2 - 5985\, z^4 + 2986\, s^2 + 5250\, z^2 - 853)\bar{e}_{\phi} \quad (33)$$

```
> simplify(int(int( u_cyl[2],z=-sqrt(1-s^2)..sqrt(1-s^2)),phi=0..2*Pi)); #check that no geostrophic component remains
```

$$0 \quad (34)$$

```
> simplify(u_cyl[2]/A_pol);
```

$$9120\, s^7 + (50400\, z^2 - 26184)\, s^5 + (50400\, z^4 - 95760\, z^2 + 23888)\, s^3 + (16800\, z^6 - 47880\, z^4 + 42000\, z^2 - 6824)\, s \quad (35)$$

```
> map(factor, simplify(MapToBasis(u_soln, spherical[r,theta,phi]))) ;  # added
```

$$\frac{1}{82336}\, r \sin(\theta)\, \Big(8915445 \cos(\theta)^6 r^6 T_{3,0,2} - 26746335 \cos(\theta)^4 r^6 T_{3,0,2} - 2315700 \cos(\theta)^4 r^4 T_{3,0,1} + 15283620 \cos(\theta)^4 r^4 T_{3,0,2} + 26746335 \cos(\theta)^2 r^6 T_{3,0,2} + 4631400 \cos(\theta)^2 r^4 T_{3,0,1} - 30567240 \cos(\theta)^2 r^4 T_{3,0,2} - 8915445\, r^6 T_{3,0,2} + 15523200\, r^6 + 617520 \cos(\theta)^2 r^2 T_{3,0,0} - 2778840 \cos(\theta)^2 r^2 T_{3,0,1} + 7641810 \cos(\theta)^2 r^2 T_{3,0,2} - 2315700\, r^4 T_{3,0,1} + 15283620\, r^4 T_{3,0,2} - 44241120\, r^4 - 617520\, r^2 T_{3,0,0} + 2778840\, r^2 T_{3,0,1} - 7641810\, r^2 T_{3,0,2} + 38808000\, r^2 + 82336\, T_{1,0,0} + 411680\, T_{3,0,0} - 694710\, T_{3,0,1} + 1018908\, T_{3,0,2} - 10267950\Big)\bar{e}_{\phi} \quad (36)$$

# > # Construct the ODE for geostrophic flow

```
> B_cyl := simplify(MapToBasis(B_cart, cylindrical[s,phi,z]));
```

$$B\_cyl := -\frac{30}{2573}\sqrt{1188726}\, s \left(s^2 + z^2 - \frac{19}{20}\right) z\bar{e}_s + \frac{1}{5146}\, (90\, s^4 + (120\, z^2 - 114)\, s^2 + 30\, z^4 - 57\, z^2 + 25)\sqrt{1188726}\,\bar{e}_z \quad (37)$$

> $int(int(CrossProduct(s \cdot Curl(B\_cyl), B\_cyl)[2], z = -\mathrm{sqrt}(1 - s\hat{}2)..\mathrm{sqrt}(1 - s\hat{}2)), \mathrm{phi} = 0..2 \cdot \mathrm{Pi})$; #check that it is taylor state

$$0 \quad (38)$$

> $u\_geo := ug(s) \cdot VectorField([0, 1, 0])$; #introduce geostrophic component

$$u\_geo := (ug(s))\bar{e}_{\phi} \quad (39)$$

> $B\_cyl\_dot := eval(Curl(CrossProduct(u\_cyl, B\_cyl)) + Curl(CrossProduct(u\_geo, B\_cyl)) + \mathrm{eta} \cdot Laplacian(B\_cyl))$; # use induction equation

$$
\begin{aligned}
B\_cyl\_dot := {} & \Bigg(\eta\Bigg(-\frac{1}{s^2}\Bigg(-\frac{60}{2573}\sqrt{1188726}\,s\left(s^2+z^2-\frac{19}{20}\right)z-\frac{60}{2573}s^3\sqrt{1188726}\,z \\
& +\frac{1}{5146}s\left(240s^2z+120z^3-114z\right)\sqrt{1188726}\Bigg)+\frac{1}{s}\Bigg(-\frac{60}{2573}\sqrt{1188726}\Big(s^2 \\
& +z^2-\frac{19}{20}\Big)z-\frac{60}{2573}\sqrt{1188726}\,s^2z+\frac{1}{5146}\Big(240s^2z+120z^3 \\
& -114z\Big)\sqrt{1188726}\Bigg)-\frac{420}{2573}\sqrt{1188726}\,sz\Bigg)\Bigg)\bar{e}_s+\Bigg(\frac{231}{13240658}s\Big(12600s^4z \\
& +25200s^2z^3+12600z^5-23940s^2z-23940z^3+10500z\Big)\Big(90s^4+\left(120z^2-114\right)s^2 \\
& +30z^4-57z^2+25\Big)\sqrt{1188726}+\frac{231}{13240658}s\Big(1140s^6+6300s^4z^2+6300s^2z^4 \\
& +2100z^6-3273s^4-11970s^2z^2-5985z^4+2986s^2+5250z^2-853\Big)\Big(240s^2z \\
& +120z^3-114z\Big)\sqrt{1188726}-\frac{13860}{6620329}s\Big(1140s^6+6300s^4z^2+6300s^2z^4+2100z^6 \\
& -3273s^4-11970s^2z^2-5985z^4+2986s^2+5250z^2-853\Big)\sqrt{1188726}\Big(s^2+z^2 \\
& -\frac{19}{20}\Big)z-\frac{6930}{6620329}s^2\Big(6840s^5+25200s^3z^2+12600sz^4-13092s^3-23940sz^2 \\
& +5972s\Big)\sqrt{1188726}\left(s^2+z^2-\frac{19}{20}\right)z-\frac{13860}{6620329}s^3\Big(1140s^6+6300s^4z^2 \\
& +6300s^2z^4+2100z^6-3273s^4-11970s^2z^2-5985z^4+2986s^2+5250z^2-853\Big) \\
& \sqrt{1188726}\,z+\frac{1}{5146}ug(s)\left(240s^2z+120z^3-114z\right)\sqrt{1188726} \\
& -\frac{30}{2573}\left(\frac{d}{ds}ug(s)\right)\sqrt{1188726}\,s\left(s^2+z^2-\frac{19}{20}\right)z-\frac{30}{2573}ug(s)\sqrt{1188726}\Big(s^2 \\
& +z^2-\frac{19}{20}\Big)z-\frac{60}{2573}ug(s)\sqrt{1188726}\,s^2z\Bigg)\bar{e}_\phi+\Bigg(\eta\Bigg(\frac{1}{s}\Bigg(-\frac{120}{2573}\sqrt{1188726}\,sz^2 \\
& -\frac{60}{2573}\sqrt{1188726}\,s\left(s^2+z^2-\frac{19}{20}\right)-\frac{60}{2573}s^3\sqrt{1188726}+\frac{1}{5146}s\Big(240s^2 \\
& +360z^2-114\Big)\sqrt{1188726}\Bigg)-\frac{1}{s}\Bigg(-\frac{60}{2573}\sqrt{1188726}\,sz^2 \\
& -\frac{30}{2573}\sqrt{1188726}\,s\left(s^2+z^2-\frac{19}{20}\right)-\frac{1}{5146}\Big(360s^3+2\big(120z^2 \\
& -114\big)s\Big)\sqrt{1188726}+s\Bigg(-\frac{60}{2573}\sqrt{1188726}\,z^2-\frac{30}{2573}\sqrt{1188726}\left(s^2+z^2-\frac{19}{20}\right) \\
& -\frac{60}{2573}\sqrt{1188726}\,s^2-\frac{1}{5146}\left(1080s^2+240z^2-228\right)\sqrt{1188726}\Bigg)\Bigg)\Bigg)\Bigg)\bar{e}_z
\end{aligned}
\tag{40}
$$

> #*Solution for the geostrophic flow using the Braginsky equation*

> $H := simplify(2\cdot \mathrm{Pi}\cdot(\ int(B\_cyl[1]\cdot B\_cyl\_dot[2] + B\_cyl[2]\cdot B\_cyl\_dot[1],\ z=-\mathrm{sqrt}(1-s\hat{}2)..\mathrm{sqrt}(1-s\hat{}2))));$

$$H := \frac{1}{86064277}\left(4238657280\, s^2\left(s^4-\frac{73}{40}s^2+\frac{107}{128}\right)\pi\left(\frac{d}{ds}ug(s)\right)-4238657280\, s\left(s^4-\frac{73}{40}s^2+\frac{107}{128}\right)\pi\, ug(s)-198\,\pi\, s\,(15586099200\, s^{11}-62163763200\, s^9+94378876032\, s^7-67154547264\, s^5+21791077392\, s^3-2437742160\, s)\right)(-s^2+1)^{3/2} \quad \mathbf{(41)}$$

> $sol\_H := simplify(dsolve(H=0, ug(s)));$

$$sol\_H := ug(s) = \frac{1}{572646880}\, s\left(69488025600\, s^6-225496696320\, s^4+214653908\sqrt{21}\arctan\left(\frac{1}{21}(80\, s^2-73)\sqrt{21}\right)+264991586160\, s^2+711869403\ln(640\, s^4-1168\, s^2+535)-78007749120\ln(s)+572646880\, \_C1\right) \quad \mathbf{(42)}$$

> #*Alternative solution defining ODE explicitly*

> **alpha_integrand := s^2 * B_cyl[1]^2;**

$$alpha\_integrand := \frac{415800}{2573}\, s^4\left(s^2+z^2-\frac{19}{20}\right)^2 z^2 \quad \mathbf{(43)}$$

> **alpha := simplify(int(int(alpha_integrand, phi=0..2*Pi), z=-sqrt(1-s^2)..sqrt(1-s^2)));**

$$\alpha := -\frac{198}{2573}\, s^4\,\pi\sqrt{-s^2+1}\,(640\, s^6-1808\, s^4+1703\, s^2-535) \quad \mathbf{(44)}$$

> **G_new_integrand1 := simplify(B_cyl[1]*(Curl(CrossProduct(u_cyl, B_cyl)) + Laplacian(B_cyl))[2]);**

$$G\_new\_integrand1 := -\frac{1158360588000}{6620329}\, s^2\left(s^2+z^2-\frac{19}{20}\right)\left(s^8+\left(\frac{172}{67}z^2-\frac{671}{201}\right)s^6+\left(\frac{210}{67}z^4-\frac{6889}{1005}z^2+\frac{240383}{60300}\right)s^4+\left(\frac{140}{67}z^6-\frac{399}{67}z^4+\frac{35507}{6030}z^2-\frac{60629}{30150}\right)s^2+\frac{35}{67}\left(z^4-\frac{19}{10}z^2+\frac{5}{6}\right)^2\right)z^2 \quad \mathbf{(45)}$$

> **G_new_integrand2 := simplify(B_cyl[2]*(Curl(CrossProduct(u_cyl, B_cyl)) + Laplacian(B_cyl))[1]);**

$$G\_new\_integrand2 := 0 \quad \mathbf{(46)}$$

> **for** $idx$ **from** 1 **to** 2 **do** $G\_new \| idx := simplify(int(int(-G\_new\_integrand \| idx,\ \mathrm{phi}=0..2*\mathrm{Pi}),\ z=-\mathrm{sqrt}(1-s\hat{}2)..\mathrm{sqrt}(1-s\hat{}2)))$ **end do**;

$$G\_new1 := -\frac{66528}{86064277}\, s^2\,\pi\sqrt{-s^2+1}\,(46387200\, s^{12}-231398400\, s^{10}+465900712\, s^8-480754236\, s^6+264719121\, s^4-72109582\, s^2+7255185)$$

$$G\_new2 := 0 \quad (47)$$

> $G\_new := add(G\_new || idx, idx = 1..2);$

$$G\_new := -\frac{66528}{86064277} s^2 \pi \sqrt{-s^2+1} \left(46387200 s^{12} - 231398400 s^{10} + 465900712 s^8 - 480754236 s^6 + 264719121 s^4 - 72109582 s^2 + 7255185\right) \quad (48)$$

```
> correct_ode := simplify((alpha/s*diff((u_g(s)/s),s))-G_new);
```

$$correct\_ode := \frac{198}{86064277} (s-1) \left(15586099200 s^{11} - 62163763200 s^9 + 94378876032 s^7 - 21407360 \left(\frac{d}{ds} u\_g(s)\right) s^5 - 67154547264 s^5 + 21407360 u\_g(s) s^4 + 39068432 \left(\frac{d}{ds} u\_g(s)\right) s^3 + 21791077392 s^3 - 39068432 u\_g(s) s^2 - 17895215 \left(\frac{d}{ds} u\_g(s)\right) s - 2437742160 s + 17895215 u\_g(s)\right) s \sqrt{-s^2+1} (s+1) \pi \quad (49)$$

> $sol\_correct\_ode := simplify(dsolve(correct\_ode, u\_g(s)));$

$$sol\_correct\_ode := u\_g(s) = \frac{1}{572646880} s \left(69488025600 s^6 - 225496696320 s^4 + 214653908 \sqrt{21} \arctan\left(\frac{1}{21} (80 s^2 - 73) \sqrt{21}\right) + 264991586160 s^2 + 711869403 \ln(640 s^4 - 1168 s^2 + 535) - 78007749120 \ln(s) + 572646880 \_C1\right) \quad (50)$$

> *#Simplest way of solving*

> $simplest\_analytic\_solution := simplify\left(s \cdot int\left(\frac{s \cdot G\_new}{\text{alpha}}, s\right)\right) + \_C1 \cdot s;$

$$simplest\_analytic\_solution := s\,\_C1 + \frac{7}{572646880} \left(9926860800 s^6 - 32213813760 s^4 + 30664844 \sqrt{21} \arctan\left(\frac{1}{21} (80 s^2 - 73) \sqrt{21}\right) + 37855940880 s^2 + 101695629 \ln(640 s^4 - 1168 s^2 + 535) - 11143964160 \ln(s)\right) s \quad (51)$$

> ***#show that the solution is inconsistent with Taylor's ODE***

>

> *#Calculate Taylor's beta and G*

```
> beta_integrand := simplify( s* (2 * B_cyl[1]^2 + s * DotProduct
  (B_cyl, Gradient(B_cyl[1]))));
```

$$beta\_integrand := -\frac{623700}{2573} s^3 \left(s^6 + \left(z^2 - \frac{133}{60}\right) s^4 + \left(-z^4 - \frac{19}{30} z^2 + \frac{1333}{900}\right) s^2 - z^6 + \frac{19}{12} z^4 - \frac{37}{100} z^2 - \frac{19}{72}\right) \quad (52)$$

```
> beta := simplify(int(int(beta_integrand, phi=0..2*Pi), z=-sqrt(1-
  s^2)..sqrt(1-s^2)));
```

$$\beta := -\frac{198}{2573}\, s^3 \pi \sqrt{-s^2+1}\, \left(7680\, s^6 - 17440\, s^4 + 12456\, s^2 - 2689\right) \tag{53}$$

```
> G_integrand1 := simplify(CrossProduct(Curl(Curl(CrossProduct
  (u_cyl, B_cyl))), B_cyl)[2]);
```

$$G\_integrand1 := \frac{1737540882000}{6620329}\, s \left(s^{12} + \left(\frac{476}{201} z^2 - \frac{4628}{1005}\right) s^{10} + \left(\frac{1189}{201} z^4 - \frac{4529}{402} z^2 + \frac{512113}{60300}\right) s^8 + \left(-\frac{2408299}{301500} + \frac{2384}{201} z^6 - \frac{1662}{67} z^4 + \frac{923588}{45225} z^2\right) s^6 + \left(\frac{2275}{201} z^8 - \frac{33863}{1005} z^6 + \frac{3261827}{90450} z^4 - \frac{1284091}{72360} z^2 + \frac{21801737}{5427000}\right) s^4 + \left(\frac{980}{201} z^{10} - \frac{1330}{67} z^8 + \frac{54854}{1809} z^6 - \frac{109136}{5025} z^4 - \frac{27626}{27135} + \frac{1667068}{226125} z^2\right) s^2 + \frac{175}{201} \left(z^4 - \frac{19}{10} z^2 + \frac{5}{6}\right)^2 \left(z^4 - \frac{57}{50} z^2 + \frac{1}{6}\right)\right) \tag{54}$$

```
> G_integrand2 := simplify(CrossProduct(Curl(B_cyl), Curl
  (CrossProduct(u_cyl, B_cyl)))[2]);
```

$$G\_integrand2 := 0 \tag{55}$$

```
> G_integrand3 := simplify(CrossProduct(Curl(Laplacian(B_cyl)),
  B_cyl)[2]);
```

$$G\_integrand3 := 0 \tag{56}$$

```
> G_integrand4 := simplify(CrossProduct(Curl(B_cyl), Laplacian
  (B_cyl))[2]);
```

$$G\_integrand4 := 0 \tag{57}$$

```
> for idx from 1 to 4 do G||idx := simplify(int(int(-
  G_integrand||idx*s, phi=0..2*Pi), z=-sqrt(1-s^2)..sqrt(1-s^2)))
  end do;
```

$$G1 := -\frac{66528}{86064277}\, s^2 \pi \sqrt{-s^2+1}\, \left(788582400\, s^{12} - 3424588800\, s^{10} + 5871698056\, s^8 - 5008127804\, s^6 + 2183964721\, s^4 - 440479243\, s^2 + 28950670\right)$$

$$G2 := 0$$

$$G3 := 0$$

$$G4 := 0 \tag{58}$$

```
> G := add(G||idx, idx=1..4);
```

$$G := -\frac{66528}{86064277}\, s^2 \pi \sqrt{-s^2+1}\, \left(788582400\, s^{12} - 3424588800\, s^{10} + 5871698056\, s^8 - 5008127804\, s^6 + 2183964721\, s^4 - 440479243\, s^2 + 28950670\right) \tag{59}$$

> $dif1 := diff\left(\frac{simplest\_analytic\_solution}{s}, s\right)$ :

> $dif2 := diff(dif1, s)$ :

> $TayLHS := simplify(\mathrm{alpha} \cdot dif2 + \mathrm{beta} \cdot dif1)$;

$$TayLHS := -\frac{66528}{86064277} \frac{1}{640\, s^4 - 1168\, s^2 + 535} \left(\left(504692736000\, s^{16} - 3112801075200\, s^{14}\right.\right. \tag{60}$$

$$+ 8179698058240\, s^{12} - 11895688291968\, s^{10} + 10389468804472\, s^8 - 5513724758708\, s^6 + 1702884326747\, s^4 - 270157468073\, s^2 + 15627668490)\, s^2 \pi \sqrt{-s^2+1}\,)$$

> *TayRHS* := *G*

$$TayRHS := -\frac{66528}{86064277}\, s^2 \pi \sqrt{-s^2+1}\, (788582400\, s^{12} - 3424588800\, s^{10} + 5871698056\, s^8 - 5008127804\, s^6 + 2183964721\, s^4 - 440479243\, s^2 + 28950670) \quad \textbf{(61)}$$

> *factor*(*simplify*(*TayLHS* − *TayRHS*));
*# A non-zero value shows that the left hand side and right hand side are different and hence our solution does not satify Taylor's equation*

$$\frac{13039488}{86064277}\, \frac{1}{640\, s^4 - 1168\, s^2 + 535}\, \left(s^2 \pi \sqrt{-(s-1)\,(s+1)}\, (s-1)\,(s+1)\,(960000\, s^8 - 3528000\, s^6 + 4629520\, s^4 - 2794033\, s^2 + 709490)\right) \quad \textbf{(62)}$$

>

# > # Combine magnetostrophic and geostrophic flow and set angular momentum to zero

```
> u_total_cyl := simplify(u_cyl[2] + simplest_analytic_solution);
```

$$u\_total\_cyl := \frac{1}{572646880}\, s \Big(128096976000\, s^6 + 323891568000\, s^4 z^2 + 323891568000\, s^2 z^4 + 107963856000\, z^6 - 393766077600\, s^4 - 615393979200\, s^2 z^2 - 307696989600\, z^4 + 214653908 \sqrt{21} \arctan\left(\frac{1}{21}\,(80\, s^2 - 73)\sqrt{21}\right) + 418505907120\, s^2 + 269909640000\, z^2 + 711869403 \ln(640\, s^4 - 1168\, s^2 + 535) - 78007749120 \ln(s) + 572646880\, \_C1 - 43853890080\Big) \quad \textbf{(63)}$$

> *#calculate constant so the angular momentum is zero*

> *with*(*Student*[*Calculus1*]) : *with*(*Student*[*NumericalAnalysis*]) :

```
> C_val := solve(Quadrature(int(int(u_total_cyl*s^2, phi = 0 .. 2*Pi), z = -sqrt(-s^2+1) .. sqrt(-s^2+1)),s=0..1,method=gaussian[50]),_C1);
```

$$C\_val := -197.1745273052853220400779702129978255320130643388 2 \quad \textbf{(64)}$$

> *u_res* := *subs*(*_C1* = *C_val*, *simplest_analytic_solution*);

$$u\_res := -197.17452730528532204007797021299782553201306433882\, s \quad \textbf{(65)}$$

$$+\frac{7}{572646880}\left(9926860800\,s^6-32213813760\,s^4+30664844\sqrt{21}\,\arctan\left(\frac{1}{21}\left(80\,s^2-73\right)\sqrt{21}\right)+37855940880\,s^2+101695629\,\ln\left(640\,s^4-1168\,s^2+535\right)-11143964160\,\ln(s)\right)s$$

> *#Plot geostrophic flow solution*

> *with(plots)* :

> *plot(u_res, s* = 0 ..1, );

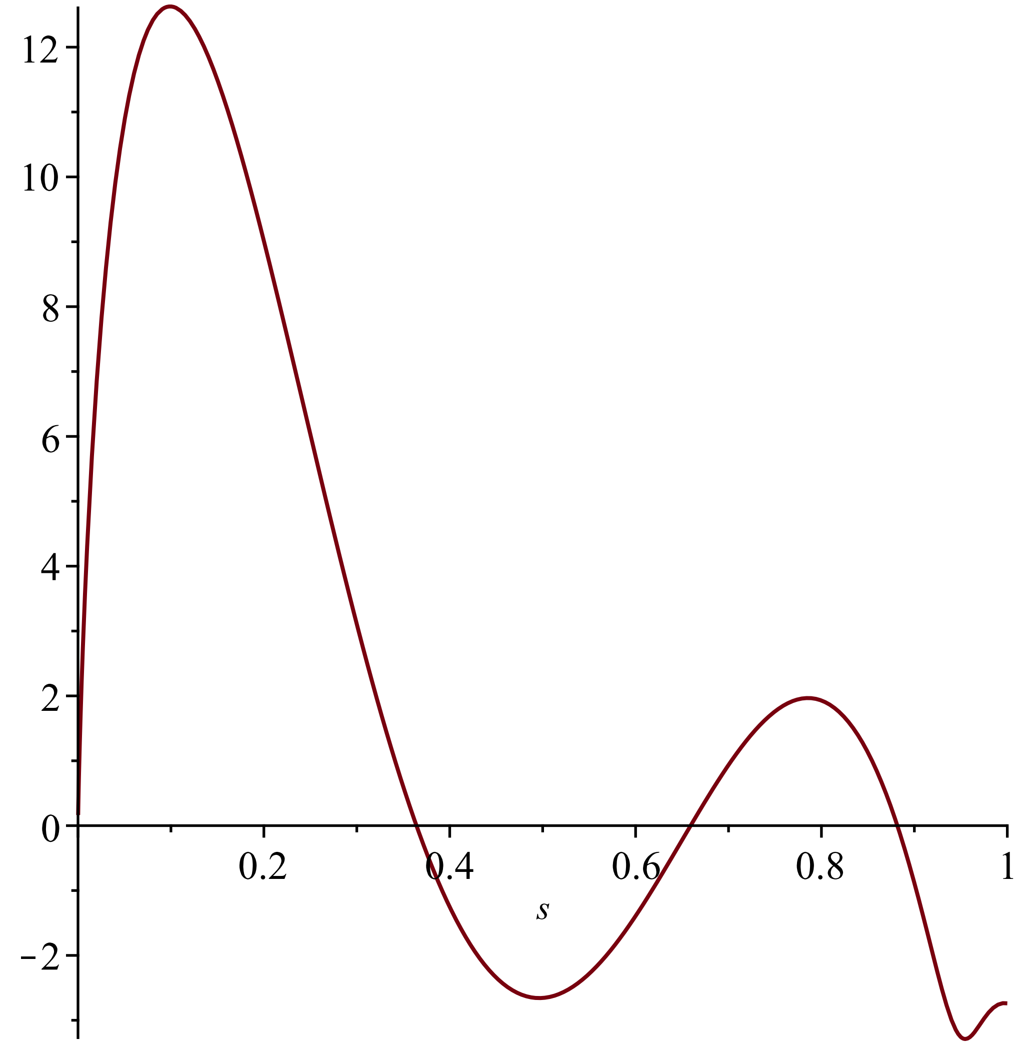

> *#compare magnitude of geostrophic and magnetrostrohic flows*

> *u_magneto* := *evalf*( *int*( *int*( *int*( (*u_cyl*[2])$^2$, phi = 0 .. 2 * Pi), *z* = - sqrt(1 - *s*^2) .. sqrt(1 - *s*^2)), *s* = 0 .. 1));

$$u\_magneto := 455.81570067946122825308331776428053715740658588577 \qquad \textbf{(66)}$$

> *#u_geostro* :=*evalf*( *int*( *int*( *int*( (*rhs*(*sol_correct_ode*))$^2$, phi = 0 .. 2 * Pi), *z* = - sqrt( - *s*^2 + 1)

.. sqrt( -s^2 + 1 ) ), s = 0 ..1 ) )

> $u\_geostro := Quadrature\Big(int\Big(int\Big( ( (u\_res) )^2$, phi = 0 .. 2 * Pi$\Big)$, $z$ = - sqrt( -s^2 + 1 ) .. sqrt( -s^2 + 1 ) $\Big)$, $s$ = 0 ..1, $method = gaussian[50]\Big)$

$u\_geostro := 356.2443079148581176080875629350376026763341430612 2$ **(67)**

> $\frac{u\_geostro}{u\_magneto}$

0.78155339402267821964024634079130815566184001546863 **(68)**
