# Supplementary material for: Determination of the instantaneous geostrophic flow within the three-dimensional magnetostrophic regime
Source: Proc Math Phys Eng Sci. 2018 Oct 3;474(2218):20180412. doi: 10.1098/rspa.2018.0412 (PMC6237501; doi:10.1098/rspa.2018.0412)

This worksheet solves Taylor's ODE to determine the instantaneous geostrophic flow, both analytically where possible and numerically using a least squares scheme.
The Taylor equation is in general not valid due to an incorrect treatmment of the boundary conditions and so these solutions are not correct, except for the specific case of purely toroidal non-axisymmetric magnetic fields.

```
> restart:
> Digits := 50:
> with(orthopoly, P):
  with(VectorCalculus):
  SetCoordinates(cartesian[x,y,z]):
```

> # Define some useful routines to construct spherical harmonics

```
> # L1 is theta-factor in spherical harmonic (note that P(l,x) is
    the l-th Legendre polynomial)
> L1 := (l,m) -> if type(m, numeric) then if m <> 0 then sin(theta)
  ^abs(m) * subs(z=cos(theta), diff(P(l,z),z$abs(m))) else P(l, cos
  (theta)) end if else 'L1'(l,m) end if;
```

$L1 := (l, m) \rightarrow \textbf{if } type(m, numeric) \textbf{ then}$ **(1)**

$\textbf{if } m \neq 0 \textbf{ then } \sin(\theta)^{|m|} subs\left(z = \cos(\theta), \frac{\partial^{|m|}}{\partial z^{|m|}} P(l, z)\right) \textbf{ else } P(l, \cos(\theta)) \textbf{ end if}$

$\textbf{else } 'L1'(l, m) \textbf{ end if}$

```
> # Lp is phi-factor
> Lp := m -> if type(m, numeric) then if m = 0 then 1 elif m < 0
  then sin(-m*phi) else cos(m*phi) end if else 'Lp'(m) end if;
```

$Lp := m \rightarrow \textbf{if } type(m, numeric) \textbf{ then}$ **(2)**

$\textbf{if } m = 0 \textbf{ then } 1 \textbf{ elif } m < 0 \textbf{ then } \sin(VectorCalculus{:}\text{-}`\text{-}`(m\,\phi)) \textbf{ else } \cos(m\,\phi) \textbf{ end if}$

$\textbf{else } 'Lp'(m) \textbf{ end if}$

```
> # norm of L1*Lp is integral of (L1*Lp)^2 over sphere is int((L1*
  Lp)^2 * sin(theta), theta=0..Pi, phi=0..2*Pi)
> L2norm_squared := (l,m) -> int(L1(l,m)^2 * sin(theta), theta=0..
  Pi) * int(Lp(m)^2, phi=0..2*Pi) / (4*Pi);
```

$L2norm\_squared := (l, m) \rightarrow VectorCalculus{:}\text{-}int\left(L1(l, m)^2 \sin(\theta), \theta = 0..\pi\right) VectorCalculus{:}\text{-}$ **(3)**

$int\left(Lp(m)^2, \phi = 0..2\,\pi\right) \frac{1}{4\,\pi}$

```
> # L2 is Schmidt quasi-normalised spherical harmonic
> L2 := (l,m) -> if type(l,numeric) and type(m,numeric) then L1(l,
  m) * Lp(m) / sqrt(L2norm_squared(l,m)) / sqrt(2*l+1) else 'L2'(l,
  m) end if;
```

$L2 := (l, m) \rightarrow \textbf{if } type(l, numeric) \textbf{ and } type(m, numeric) \textbf{ then}$ **(4)**

$L1(l, m)\, Lp(m) \frac{1}{\sqrt{L2norm\_squared(l, m)}} \frac{1}{\sqrt{2\,l + 1}}$

$\textbf{else } 'L2'(l, m) \textbf{ end if}$

```
> # Convert an expression in spherical coordinates to Cartesian
```

coordinates.

```
> sph2cart := proc(expr)
    local res;
    res := expand(expr, trig);
    res := subs(cos(phi) = x/(r*sin(theta)), sin(phi) = y/(r*sin
(theta)), res);
    res := subs(cos(theta) = z/r, sin(theta) = sqrt(x^2+y^2)/r,
res);
    res := subs(r = sqrt(x^2+y^2+z^2), res);
    return simplify(res)
end proc:
> # Construct vector field from poloidal and toroidal scalars
scalars2vf := proc(tor_scalar, pol_scalar)
    return simplify(Curl(VectorField(sph2cart(tor_scalar/r) * <x,
y,z>))
                    + Curl(Curl(VectorField(sph2cart(pol_scalar/r)
* <x,y,z>))))
end proc:
> # Define basis functions for the poloidal flow which vanish at r=
1. Degree is l+2n-1.
> Chi_n := (l,n) -> r^(l+1) * (1-r^2) * P(n-1,3/2,l+1/2,2*r^2-1): #
curl of this is basis for poloidal part
> W_n := (l,n) -> r^(l+1) * P(n,-1/2,l+1/2,2*r^2-1): # curl^2 of
this is basis for toroidal part
> Psi_n := proc (l, n) options operator, arrow; r^(l+1)*((-2*n^2*
(l+1)-n*(l+1)*(2*l-1)-l*(2*l+1))*P(n, 0, l+1/2, 2*r^2-1)+((2*l+2)
*n^2+(2*l+3)*(l+1)*n+(2*l+1)^2)*P(n-1, 0, l+1/2, 2*r^2-1)+4*n*l+
l*(2*l+1))end proc
```

$$Psi\_n := (l, n) \rightarrow r^{l+1} \Big(\big(VectorCalculus{:-}\grave{}\text{-}\grave{}(2\, n^2\, (l+1)) + VectorCalculus{:-}\grave{}\text{-}\grave{}(n\, (l+1)\, (2\, l + (-1))) + VectorCalculus{:-}\grave{}\text{-}\grave{}(l\, (2\, l+1)) \big)\, P\Big(n, 0, l+1\, \frac{1}{2}, 2\, r^2 + (-1)\Big) + \big((2\, l+2)\, n^2 + (2\, l+3)\, (l+1)\, n + (2\, l+1)^2\big)\, P\Big(n+(-1), 0, l+1\, \frac{1}{2}, 2\, r^2 + (-1)\Big) + 4\, n\, l + l\, (2\, l+1) \Big) \tag{5}$$

> # Define magnetic field

```
> # choose a number corresponding to the chosen magnetic field 1=
axisymmetric poloidal, 2=nonaxisymmetric toroidal, 3=
nonaxisymmetric poloidal, 4=nonaxisymmetric mixed state
```

> $k := 1;$

$$k := 1 \tag{6}$$

> **if** $k = 1$ **then B_scalar_tor ≔ 0 : B_scalar_pol ≔** *eval*(**r^2 * (30 * r^4 − 57 * r^2 + 25) * L2(l, m), {l = 1, m = 0, n = 1}**)
end if

$$B\_scalar\_tor := 0$$
$$B\_scalar\_pol := r^2\, (30\, r^4 - 57\, r^2 + 25)\, \cos(\theta) \tag{7}$$

```
> if k = 2 then B_scalar_tor := simplify(eval(Chi_n(l, n)·L2(l, m), {l = 1, m = 1, n = 1})) :
      B_scalar_pol := 0
  end if
> if k = 3 then B_scalar_tor := 0 : B_scalar_pol := eval(Psi_n(l, n)·L2(l, m), {l = 2, m = 2, n
      = 1})
  end
> if k = 4 then B_scalar_tor := eval(Chi_n(l, n)·L2(l, m), {l = 2, m = 1, n = 1}) :
      B_scalar_pol := eval(Psi_n(l, n)·L2(l, m), {l = 2, m = 1, n = 1})
  end if
```

```
> B_cart_pol := scalars2vf(0, B_scalar_pol);
```

$$B\_cart\_pol := (-120\,x^3\,z - 120\,x\,y^2\,z - 120\,x\,z^3 + 114\,x\,z)\bar{e}_x + (-120\,x^2\,y\,z - 120\,y^3\,z - 120\,y\,z^3 + 114\,y\,z)\bar{e}_y + (180\,x^4 + 360\,x^2\,y^2 + 240\,x^2\,z^2 + 180\,y^4 + 240\,y^2\,z^2 + 60\,z^4 - 228\,x^2 - 228\,y^2 - 114\,z^2 + 50)\bar{e}_z \quad \textbf{(8)}$$

```
> B_cart_tor := scalars2vf(B_scalar_tor, 0);
```

$$B\_cart\_tor := 0\bar{e}_x \quad \textbf{(9)}$$

```
> B_sph_pol := simplify(MapToBasis(B_cart_pol, spherical[r, theta, phi]));
```

$$B\_sph\_pol := ((60\,r^4 - 114\,r^2 + 50)\cos(\theta))\bar{e}_r + ((-180\,r^4 + 228\,r^2 - 50)\sin(\theta))\bar{e}_\theta \quad \textbf{(10)}$$

```
> B_sph_tor := simplify(MapToBasis(B_cart_tor, spherical[r, theta, phi]));
```

$$B\_sph\_tor := 0\bar{e}_r \quad \textbf{(11)}$$

```
> SetCoordinates(spherical[r, theta, phi]);
```

$$\mathit{spherical}_{r,\,\theta,\,\phi} \quad \textbf{(12)}$$

```
> #Scale the magnetic field
> Scale_pol := sqrt(1/(4·Pi) · int(int(int(A_pol·B_sph_pol.B_sph_pol·r^2·sin(theta), phi = 0..2
      · Pi), theta = 0.. Pi), r = 0..1));
```

$$Scale\_pol := \frac{2}{231}\sqrt{1188726}\,\sqrt{A\_pol} \quad \textbf{(13)}$$

```
> if Scale_pol = 0 then A_pol = 0
  else A_pol := simplify(solve(Scale_pol = 1, A_pol)); end if;
```

$$A\_pol := \frac{231}{20584} \quad \textbf{(14)}$$

```
> Scale_tor := sqrt(1/(4·Pi) · int(int(int(A_tor·B_sph_tor.B_sph_tor·r^2·sin(theta), phi = 0..2
```

$\cdot$ Pi), theta = 0 .. Pi), $r = 0..1$));

$$Scale\_tor := 0 \quad (15)$$

> **if** $Scale\_tor = 0$ **then** $A\_tor = 0$
> **else** $A\_tor := simplify(solve(Scale\_tor = 1, A\_tor))$ **end if**;

$$A\_tor = 0 \quad (16)$$

> $SetCoordinates(cartesian[x, y, z])$:

> $B\_cart := scalars2vf(\mathrm{sqrt}(A\_tor) \cdot B\_scalar\_tor, \mathrm{sqrt}(A\_pol) \cdot B\_scalar\_pol)$;

$$B\_cart := \left(-\frac{30}{2573}\sqrt{1188726}\,x\,y^2\,z - \frac{30}{2573}\sqrt{1188726}\,x^3\,z - \frac{30}{2573}\sqrt{1188726}\,x\,z^3 + \frac{57}{5146}\sqrt{1188726}\,x\,z\right)\bar{e}_x + \left(-\frac{30}{2573}\sqrt{1188726}\,x^2\,y\,z - \frac{30}{2573}\sqrt{1188726}\,y^3\,z - \frac{30}{2573}\sqrt{1188726}\,y\,z^3 + \frac{57}{5146}\sqrt{1188726}\,y\,z\right)\bar{e}_y + \left(\frac{90}{2573}\sqrt{1188726}\,x^2\,y^2 + \frac{60}{2573}\sqrt{1188726}\,x^2\,z^2 + \frac{60}{2573}\sqrt{1188726}\,y^2\,z^2 + \frac{45}{2573}\sqrt{1188726}\,x^4 + \frac{45}{2573}\sqrt{1188726}\,y^4 + \frac{15}{2573}\sqrt{1188726}\,z^4 - \frac{57}{2573}\sqrt{1188726}\,x^2 - \frac{57}{2573}\sqrt{1188726}\,y^2 - \frac{57}{5146}\sqrt{1188726}\,z^2 + \frac{25}{5146}\sqrt{1188726}\right)\bar{e}_z \quad (17)$$

```
> B_sph := simplify(MapToBasis(B_cart, spherical[r,theta,phi]));
```

$$B\_sph := \frac{1}{5146}\cos(\theta)\sqrt{1188726}\,(30\,r^4 - 57\,r^2 + 25)\bar{e}_r - \frac{1}{5146}\sin(\theta)\sqrt{1188726}\,(90\,r^4 - 114\,r^2 + 25)\bar{e}_\theta \quad (18)$$

> # Compute rhs of magnetostrophic equation

```
> # slaved equation is Omega cross u = -div(p) + curl(B) cross B,
  we ignore the pressure
> RHS := CrossProduct(Curl(B_cart), B_cart): simplify(RHS);
```

$$-\frac{3465}{5146}\,x\,(28\,x^2 + 28\,y^2 + 28\,z^2 - 19)\,(90\,x^4 + 180\,x^2\,y^2 + 120\,x^2\,z^2 + 90\,y^4 + 120\,y^2\,z^2 + 30\,z^4 - 114\,x^2 - 114\,y^2 - 57\,z^2 + 25)\bar{e}_x - \frac{3465}{5146}\,y\,(28\,x^2 + 28\,y^2 + 28\,z^2 - 19)\,(90\,x^4 \quad (19)$$

$$+ 180 x^2 y^2 + 120 x^2 z^2 + 90 y^4 + 120 y^2 z^2 + 30 z^4 - 114 x^2 - 114 y^2 - 57 z^2 + 25)\bar{e}_y$$

$$- \frac{2910600}{2573} (x^2 + y^2) \left(x^2 + y^2 + z^2 - \frac{19}{28}\right) \left(x^2 + y^2 + z^2 - \frac{19}{20}\right) z\bar{e}_z$$

```
> map(factor, simplify(MapToBasis(RHS, spherical[r,theta,phi])));
  # for comparison
```

$$-\frac{3465}{5146} \sin(\theta)^2 (28 r^2 - 19) (90 r^4 - 114 r^2 + 25) r\bar{e}_r - \frac{3465}{5146} \cos(\theta) r \sin(\theta) (28 r^2 - 19) (30 r^4 - 57 r^2 + 25)\bar{e}_\theta \quad (20)$$

> # Construct basis for u

```
> # Let N be the degree of B. Then the degree of curl(B) cross B is
  (N-1) + N = 2N-1. Thus the degree of u is also 2N-1. Thus, the
  poloidal scalar has degree 2N+1 (we have to undo two curls) and
  the toroidal scalar has degree 2N. Furthermore, the m-degree of
  curl(B) cross B is twice the m-degree of B.
> B_degree := max(seq(degree(B_cart[idx], {x,y,z}), idx = 1 .. 3));
```

$$B\_degree := 4 \quad (21)$$

```
> Max_pol_degree := 2 * B_degree + 1; Max_tor_degree := 2 *
  B_degree;
```

$$Max\_pol\_degree := 9$$
$$Max\_tor\_degree := 8 \quad (22)$$

```
> Max_m_degree := 2 * max(seq(degree(expand(B_sph[idx], trig), {cos
  (phi), sin(phi)}), idx = 1 .. 3));
```

$$Max\_m\_degree := 0 \quad (23)$$

```
> # For the particular example here, certain modes are zero, but we
  will not exploit this knowledge.
> # Also note that unless B is a Taylor state, there will be no
  solution to the magnetostrophic equation.

> u_scalar_pol := add(add(add(S[l,m,n] * L2(l,m) * Chi_n(l,n),
                          m = -min(l, Max_m_degree) .. min(l,
  Max_m_degree)),
                          n = 1 .. (Max_pol_degree - l + 1) /
  2),
                          l = 1 .. Max_pol_degree):
> u_scalar_tor := add(add(add(T[l,m,n] * L2(l,m) * W_n(l,n),
                          m = -min(l, Max_m_degree) .. min(l,
  Max_m_degree)),
                          n = 0 .. floor((Max_tor_degree - l +
  1)/2)),
                          l = 1 .. Max_tor_degree):
> coeff(u_scalar_pol, S[2,-2,2]);
```

$$0 \quad (24)$$

```
> # u is poloidal part (curl^2 of scalar times hat r) + toroidal
  part (curl of scalar times hat r)
> u_cart := scalars2vf(u_scalar_tor, u_scalar_pol):
> variables := indets(u_cart, indexed);
```

$$variables := \{S_{1,0,1}, S_{1,0,2}, S_{1,0,3}, S_{1,0,4}, S_{2,0,1}, S_{2,0,2}, S_{2,0,3}, S_{2,0,4}, S_{3,0,1}, S_{3,0,2}, S_{3,0,3}, \quad (25)$$

$S_{4,0,1}, S_{4,0,2}, S_{4,0,3}, S_{5,0,1}, S_{5,0,2}, S_{6,0,1}, S_{6,0,2}, S_{7,0,1}, S_{8,0,1}, T_{1,0,0}, T_{1,0,1}, T_{1,0,2}, T_{1,0,3}, T_{1,0,4}, T_{2,0,0}, T_{2,0,1}, T_{2,0,2}, T_{2,0,3}, T_{3,0,0}, T_{3,0,1}, T_{3,0,2}, T_{3,0,3}, T_{4,0,0}, T_{4,0,1}, T_{4,0,2}, T_{5,0,0}, T_{5,0,1}, T_{5,0,2}, T_{6,0,0}, T_{6,0,1}, T_{7,0,0}, T_{7,0,1}, T_{8,0,0}\}$

> # Compute lhs of magnetostrophic equation

```
> # slaved equation is Omega cross u = -div(p) + curl(B) cross B
> Omega_vec := VectorField([0,0,1]);  # rotation vector in cartesian coordinates
```

$$Omega\_vec := \bar{e}_z \quad (26)$$

```
> LHS := CrossProduct(Omega_vec, u_cart):
```

> # Solve magnetostrophic equation for basis coefficients

```
> # take the curl of slaved equation; pressure drops out
> eqn := simplify(Curl(LHS - RHS)):
> constraints := `union`(seq({coeffs(collect(eqn[i], [x,y,z], distributed), [x,y,z])}, i=1..3)):
> nops(constraints); nops(variables);
```

113

44 (27)

```
> sol1 := solve(constraints, variables);
```

$$sol1 := \{S_{1,0,1} = 0, S_{1,0,2} = 0, S_{1,0,3} = 0, S_{1,0,4} = 0, S_{2,0,1} = 0, S_{2,0,2} = 0, S_{2,0,3} = 0, S_{2,0,4} = 0, S_{3,0,1} = 0, S_{3,0,2} = 0, S_{3,0,3} = 0, S_{4,0,1} = 0, S_{4,0,2} = 0, S_{4,0,3} = 0, S_{5,0,1} = 0, S_{5,0,2} = 0, S_{6,0,1} = 0, S_{6,0,2} = 0, S_{7,0,1} = 0, S_{8,0,1} = 0, T_{1,0,0} = T_{1,0,0}, T_{1,0,1} = T_{1,0,1}, T_{1,0,2} = T_{1,0,2}, T_{1,0,3} = T_{1,0,3}, T_{1,0,4} = 0, T_{2,0,0} = 0, T_{2,0,1} = 0, T_{2,0,2} = 0, T_{2,0,3} = 0, T_{3,0,0} = \frac{27489}{10292} - \frac{1}{2} T_{1,0,1}, T_{3,0,1} = -\frac{23177}{2573} - \frac{14}{27} T_{1,0,2}, T_{3,0,2} = \frac{7350}{2573} - \frac{35}{66} T_{1,0,3}, T_{3,0,3} = 0, T_{4,0,0} = 0, T_{4,0,1} = 0, T_{4,0,2} = 0, T_{5,0,0} = \frac{16555}{2573} + \frac{10}{27} T_{1,0,2}, T_{5,0,1} = -\frac{69300}{33449} + \frac{5}{13} T_{1,0,3}, T_{5,0,2} = 0, T_{6,0,0} = 0, T_{6,0,1} = 0, T_{7,0,0} = \frac{55125}{33449} - \frac{175}{572} T_{1,0,3}, T_{7,0,1} = 0, T_{8,0,0} = 0\} \quad (28)$$

> # set geostrophic component to zero to remove degeneracy

```
> u_soln := simplify(subs(sol1, u_cart));
```

$$u\_soln := -\frac{3675}{64}\left(\left(\left(-\frac{5412}{2573}+T_{1,0,3}\right)y^6+\left(\left(-\frac{16236}{2573}+3\,T_{1,0,3}\right)x^2+\frac{55176}{12865}-\frac{12}{7}T_{1,0,3}+\frac{16}{63}T_{1,0,2}+\frac{25344}{2573}z^2\right)y^4+\left(\left(-\frac{16236}{2573}+3\,T_{1,0,3}\right)x^4+\left(\frac{50688}{2573}z^2+\frac{32}{63}T_{1,0,2}-\frac{24}{7}T_{1,0,3}+\frac{110352}{12865}\right)x^2-\frac{32}{105}T_{1,0,2}+\frac{6}{7}T_{1,0,3}-\frac{5280}{2573}-\frac{240768}{12865}z^2+\frac{16}{245}T_{1,0,1}+\frac{25344}{2573}z^4\right)y^2+\left(-\frac{5412}{2573}+T_{1,0,3}\right)x^6+\left(\frac{55176}{12865}-\frac{12}{7}T_{1,0,3}+\frac{16}{63}T_{1,0,2}+\frac{25344}{2573}z^2\right)x^4+\left(-\frac{32}{105}T_{1,0,2}+\frac{6}{7}T_{1,0,3}-\frac{5280}{2573}-\frac{240768}{12865}z^2+\frac{16}{245}T_{1,0,1}+\frac{25344}{2573}z^4\right)x^2-\frac{32}{735}T_{1,0,1}+\frac{8}{105}T_{1,0,2}-\frac{4}{35}T_{1,0,3}+\frac{64}{3675}T_{1,0,0}+\frac{21120}{2573}z^2-\frac{120384}{12865}z^4+\frac{8448}{2573}z^6\right)y\bar{e}_x+\frac{3675}{64}\left(\left(-\frac{5412}{2573}+T_{1,0,3}\right)x^6+\left(\left(-\frac{16236}{2573}+3\,T_{1,0,3}\right)y^2+\frac{55176}{12865}-\frac{12}{7}T_{1,0,3}+\frac{16}{63}T_{1,0,2}+\frac{25344}{2573}z^2\right)x^4+\left(\left(-\frac{16236}{2573}+3\,T_{1,0,3}\right)y^4+\left(\frac{50688}{2573}z^2+\frac{32}{63}T_{1,0,2}-\frac{24}{7}T_{1,0,3}+\frac{110352}{12865}\right)y^2-\frac{32}{105}T_{1,0,2}+\frac{6}{7}T_{1,0,3}-\frac{5280}{2573}-\frac{240768}{12865}z^2+\frac{16}{245}T_{1,0,1}+\frac{25344}{2573}z^4\right)x^2+\left(-\frac{5412}{2573}+T_{1,0,3}\right)y^6+\left(\frac{55176}{12865}-\frac{12}{7}T_{1,0,3}+\frac{16}{63}T_{1,0,2}+\frac{25344}{2573}z^2\right)y^4+\left(-\frac{32}{105}T_{1,0,2}+\frac{6}{7}T_{1,0,3}-\frac{5280}{2573}-\frac{240768}{12865}z^2+\frac{16}{245}T_{1,0,1}+\frac{25344}{2573}z^4\right)y^2-\frac{32}{735}T_{1,0,1}+\frac{8}{105}T_{1,0,2}-\frac{4}{35}T_{1,0,3}+\frac{64}{3675}T_{1,0,0}+\frac{21120}{2573}z^2-\frac{120384}{12865}z^4+\frac{8448}{2573}z^6\right)x\bar{e}_y \quad (29)$$

```
> u_cyl := simplify(MapToBasis(u_soln, cylindrical[s,phi,z]));
```

$$u\_cyl := \frac{3675}{64}\left(\left(\left(-\frac{5412}{2573}+T_{1,0,3}\right)s^6+\left(\frac{55176}{12865}-\frac{12}{7}T_{1,0,3}+\frac{16}{63}T_{1,0,2}+\frac{25344}{2573}z^2\right)s^4+\left(-\frac{32}{105}T_{1,0,2}+\frac{6}{7}T_{1,0,3}-\frac{5280}{2573}-\frac{240768}{12865}z^2+\frac{16}{245}T_{1,0,1}+\frac{25344}{2573}z^4\right)s^2+\frac{8448}{2573}z^6-\frac{120384}{12865}z^4+\frac{21120}{2573}z^2+\frac{64}{3675}T_{1,0,0}-\frac{32}{735}T_{1,0,1}+\frac{8}{105}T_{1,0,2}-\frac{4}{35}T_{1,0,3}\right)s\bar{e}_\phi \quad (30)$$

> $SetCoordinates(cylindrical[s, \mathrm{phi}, z])$

$$cylindrical_{s,\phi,z} \quad (31)$$

```
> geostrophic_component := simplify((int(int(u_cyl[2], phi = 0 .. 2*Pi), z = -sqrt(-s^2+1) .. sqrt(-s^2+1)))/(4*Pi*sqrt(-s^2+1)))
```

$$geostrophic\_component := \frac{3675}{64}\left(\left(T_{1,0,3}-\frac{349932}{90055}\right)s^6+\left(\frac{16}{63}T_{1,0,2}-\frac{12}{7}T_{1,0,3} \quad (32)\right.\right.$$

$$+\frac{4235352}{450275}\Big) s^4+\Big(\frac{16}{245}T_{1,0,1}-\frac{32}{105}T_{1,0,2}+\frac{6}{7}T_{1,0,3}-\frac{3026144}{450275}\Big)s^2 + \frac{64}{3675}T_{1,0,0}-\frac{32}{735}T_{1,0,1}+\frac{8}{105}T_{1,0,2}-\frac{4}{35}T_{1,0,3}+\frac{600512}{450275}\Big)s$$

```
> u_cyl := simplify(u_cyl - VectorField([0, geostrophic_component, 0])); #remove geostrophic component from u_cyl
```

$$u\_cyl := \frac{231}{2573}s\,(1140 s^6+6300 s^4 z^2+6300 s^2 z^4+2100 z^6-3273 s^4-11970 s^2 z^2-5985 z^4+2986 s^2+5250 z^2-853)\bar{e}_\phi \quad (33)$$

```
> simplify(int(int( u_cyl[2],z=-sqrt(1-s^2)..sqrt(1-s^2)),phi=0..2*Pi)); #check that no geostrophic component remains
```

$$0 \quad (34)$$

```
> map(factor, simplify(MapToBasis(u_soln, spherical[r,theta,phi]))); # added
```

$$-\frac{1}{494016}r\sin(\theta)\,(8645280 r^2 T_{1,0,2}-24314850 r^2 T_{1,0,3}-7204400 r^4 T_{1,0,2}+48629700 r^4 T_{1,0,3}+3241980 T_{1,0,3}-2161320 T_{1,0,2}+1235040 T_{1,0,1}-494016 T_{1,0,0}-1852560 r^2 T_{1,0,1}+28367325\cos(\theta)^6 r^6 T_{1,0,3}-85101975\cos(\theta)^4 r^6 T_{1,0,3}-7204400\cos(\theta)^4 r^4 T_{1,0,2}+48629700\cos(\theta)^4 r^4 T_{1,0,3}+85101975\cos(\theta)^2 r^6 T_{1,0,3}+14408800\cos(\theta)^2 r^4 T_{1,0,2}-97259400\cos(\theta)^2 r^4 T_{1,0,3}+1852560\cos(\theta)^2 r^2 T_{1,0,1}-8645280\cos(\theta)^2 r^2 T_{1,0,2}+24314850\cos(\theta)^2 r^2 T_{1,0,3}-28367325 r^6 T_{1,0,3}+59667300 r^6-291060000 r^2\cos(\theta)^2-387109800 r^4\cos(\theta)^4-152806500 r^6\cos(\theta)^6+458419500 r^6\cos(\theta)^4-458419500 r^6\cos(\theta)^2+774219600 r^4\cos(\theta)^2-121663080 r^4+58212000 r^2)\bar{e}_\phi \quad (35)$$

> # Construct the ODE for geostrophic flow

```
> B_cyl := simplify(MapToBasis(B_cart, cylindrical[s,phi,z]));
```

$$B\_cyl := -\frac{30}{2573}\sqrt{1188726}\Big(s^2+z^2-\frac{19}{20}\Big)z\,s\bar{e}_s+\frac{1}{5146}(90 s^4+(120 z^2-114)s^2+30 z^4-57 z^2+25)\sqrt{1188726}\,\bar{e}_z \quad (36)$$

> $int(int(CrossProduct(s\cdot Curl(B\_cyl), B\_cyl)[2], z=-\mathrm{sqrt}(1-s\text{^}2)..\mathrm{sqrt}(1-s\text{^}2)), \mathrm{phi}=0..2\cdot\mathrm{Pi})$; #check that it is taylor state

$$0 \quad (37)$$

> $u\_geo := ug(s) \cdot VectorField([0, 1, 0]);$ #introduce geostrophic component

$$u\_geo := (ug(s))\bar{e}_{\phi} \tag{38}$$

> $B\_cyl\_dot := eval(Curl(CrossProduct(u\_cyl, B\_cyl)) + Curl(CrossProduct(u\_geo, B\_cyl)) + \text{eta} \cdot Laplacian(B\_cyl));$ # use induction equation

$$B\_cyl\_dot := \Bigg(\eta \Bigg(-\frac{1}{s^2}\Bigg(-\frac{60}{2573}\sqrt{1188726}\left(s^2+z^2-\frac{19}{20}\right) z s - \frac{60}{2573} s^3 \sqrt{1188726}\, z + \frac{1}{5146} s\,(240 s^2 z + 120 z^3 - 114 z)\sqrt{1188726}\Bigg) + \frac{1}{s}\Bigg(-\frac{60}{2573}\sqrt{1188726}\, s^2 z - \frac{60}{2573}\sqrt{1188726}\left(s^2+z^2-\frac{19}{20}\right) z + \frac{1}{5146}(240 s^2 z + 120 z^3 - 114 z)\sqrt{1188726}\Bigg) - \frac{420}{2573}\sqrt{1188726}\, z s\Bigg)\Bigg)\bar{e}_s + \Bigg(\frac{231}{13240658} s\,(12600 s^4 z + 25200 s^2 z^3 + 12600 z^5 - 23940 s^2 z - 23940 z^3 + 10500 z)\,(90 s^4 + (120 z^2 - 114) s^2 + 30 z^4 - 57 z^2 + 25)\sqrt{1188726} + \frac{231}{13240658} s\,(1140 s^6 + 6300 s^4 z^2 + 6300 s^2 z^4 + 2100 z^6 - 3273 s^4 - 11970 s^2 z^2 - 5985 z^4 + 2986 s^2 + 5250 z^2 - 853)\,(240 s^2 z + 120 z^3 - 114 z)\sqrt{1188726} - \frac{13860}{6620329} s\,(1140 s^6 + 6300 s^4 z^2 + 6300 s^2 z^4 + 2100 z^6 - 3273 s^4 - 11970 s^2 z^2 - 5985 z^4 + 2986 s^2 + 5250 z^2 - 853)\sqrt{1188726}\left(s^2+z^2-\frac{19}{20}\right) z - \frac{6930}{6620329} s^2\,(6840 s^5 + 25200 s^3 z^2 + 12600 s z^4 - 13092 s^3 - 23940 s z^2 + 5972 s)\sqrt{1188726}\left(s^2+z^2-\frac{19}{20}\right) z - \frac{13860}{6620329} s^3\,(1140 s^6 + 6300 s^4 z^2 + 6300 s^2 z^4 + 2100 z^6 - 3273 s^4 - 11970 s^2 z^2 - 5985 z^4 + 2986 s^2 + 5250 z^2 - 853)\sqrt{1188726}\, z + \frac{1}{5146} ug(s)\,(240 s^2 z + 120 z^3 - 114 z)\sqrt{1188726} - \frac{30}{2573}\left(\frac{d}{ds} ug(s)\right)\sqrt{1188726}\left(s^2+z^2-\frac{19}{20}\right) z s - \frac{60}{2573} ug(s)\sqrt{1188726}\, s^2 z - \frac{30}{2573} ug(s)\sqrt{1188726}\left(s^2+z^2-\frac{19}{20}\right) z\Bigg)\bar{e}_{\phi} + \Bigg(\eta\Bigg(\frac{1}{s}\Bigg(-\frac{120}{2573}\sqrt{1188726}\, z^2 s \tag{39}$$

$$- \frac{60}{2573} \sqrt{1188726} \left(s^2 + z^2 - \frac{19}{20}\right) s - \frac{60}{2573} s^3 \sqrt{1188726} + \frac{1}{5146} s \left(240 s^2 + 360 z^2 - 114\right) \sqrt{1188726}\right) - \frac{1}{s} \left(- \frac{60}{2573} \sqrt{1188726} z^2 s - \frac{30}{2573} \sqrt{1188726} \left(s^2 + z^2 - \frac{19}{20}\right) s - \frac{1}{5146} \left(360 s^3 + 2 \left(120 z^2 - 114\right) s\right) \sqrt{1188726} + s \left(- \frac{60}{2573} \sqrt{1188726} z^2 - \frac{60}{2573} \sqrt{1188726} s^2 - \frac{30}{2573} \sqrt{1188726} \left(s^2 + z^2 - \frac{19}{20}\right) - \frac{1}{5146} \left(1080 s^2 + 240 z^2 - 228\right) \sqrt{1188726}\right)\right) \right) \bar{e}_z$$

> *#For Taylor ODE - attempt to solve analytically, only possible for a few specific magnetic fields*

> *# Taylor's ODE is alpha * diff(ug(s)/s,s,s) + beta * diff(ug(s)/s,s) = G, So we calulate all the coefficients, so we can formulate the ODE*

```
> SetCoordinates(cylindrical[s,phi,z]);
```

$$cylindrical_{s, \phi, z} \quad (40)$$

```
> alpha_integrand := s^2 * B_cyl[1]^2;
```

$$alpha\_integrand := \frac{415800}{2573} s^4 \left(s^2 + z^2 - \frac{19}{20}\right)^2 z^2 \quad (41)$$

```
> alpha := simplify(int(int(alpha_integrand, phi=0..2*Pi), z=-sqrt(1-s^2)..sqrt(1-s^2)));
```

$$\alpha := - \frac{198}{2573} s^4 \pi \sqrt{-s^2 + 1} \left(640 s^6 - 1808 s^4 + 1703 s^2 - 535\right) \quad (42)$$

```
> beta_integrand := simplify( s* (2 * B_cyl[1]^2 + s * DotProduct(B_cyl, Gradient(B_cyl[1]))));
```

$$beta\_integrand := - \frac{623700}{2573} \left(s^6 + \left(z^2 - \frac{133}{60}\right) s^4 + \left(-z^4 - \frac{19}{30} z^2 + \frac{1333}{900}\right) s^2 - z^6 + \frac{19}{12} z^4 - \frac{37}{100} z^2 - \frac{19}{72}\right) s^3 \quad (43)$$

```
> beta := simplify(int(int(beta_integrand, phi=0..2*Pi), z=-sqrt(1-s^2)..sqrt(1-s^2)));
```

$$\beta := - \frac{198}{2573} s^3 \pi \sqrt{-s^2 + 1} \left(7680 s^6 - 17440 s^4 + 12456 s^2 - 2689\right) \quad (44)$$

```
> G_integrand1 := simplify(CrossProduct(Curl(Curl(CrossProduct(u_cyl, B_cyl))), B_cyl)[2]);
```

$$G\_integrand1 := \frac{1737540882000}{6620329} \left(s^{12} + \left(\frac{476}{201} z^2 - \frac{4628}{1005}\right) s^{10} + \left(\frac{1189}{201} z^4 - \frac{4529}{402} z^2 \right.\right. \quad (45)$$

$$+\frac{512113}{60300}\Big)s^8+\Big(\frac{2384}{201}z^6-\frac{1662}{67}z^4+\frac{923588}{45225}z^2-\frac{2408299}{301500}\Big)s^6+\Big(-\frac{1284091}{72360}z^2+\frac{21801737}{5427000}+\frac{2275}{201}z^8-\frac{33863}{1005}z^6+\frac{3261827}{90450}z^4\Big)s^4+\Big(-\frac{109136}{5025}z^4-\frac{27626}{27135}+\frac{980}{201}z^{10}-\frac{1330}{67}z^8+\frac{54854}{1809}z^6+\frac{1667068}{226125}z^2\Big)s^2+\frac{175}{201}\Big(z^4-\frac{19}{10}z^2+\frac{5}{6}\Big)^2\Big(z^4-\frac{57}{50}z^2+\frac{1}{6}\Big)\Big)s$$

```
> G_integrand2 := simplify(CrossProduct(Curl(B_cyl), Curl
  (CrossProduct(u_cyl, B_cyl)))[2]);
```

$$G\_integrand2 := 0 \quad \textbf{(46)}$$

```
> G_integrand3 := simplify(CrossProduct(Curl(Laplacian(B_cyl)),
  B_cyl)[2]);
```

$$G\_integrand3 := 0 \quad \textbf{(47)}$$

```
> G_integrand4 := simplify(CrossProduct(Curl(B_cyl), Laplacian
  (B_cyl))[2]);
```

$$G\_integrand4 := 0 \quad \textbf{(48)}$$

```
> for idx from 1 to 4 do G||idx := simplify(int(int(-
  G_integrand||idx*s, phi=0..2*Pi), z=-sqrt(1-s^2)..sqrt(1-s^2)))
  end do;
```

$$G1 := -\frac{66528}{86064277}s^2\pi\sqrt{-s^2+1}\,(788582400\,s^{12}-3424588800\,s^{10}+5871698056\,s^8-5008127804\,s^6+2183964721\,s^4-440479243\,s^2+28950670)$$

$$G2 := 0$$

$$G3 := 0$$

$$G4 := 0 \quad \textbf{(49)}$$

```
> G := add(G||idx, idx=1..4);
```

$$G := -\frac{66528}{86064277}s^2\pi\sqrt{-s^2+1}\,(788582400\,s^{12}-3424588800\,s^{10}+5871698056\,s^8-5008127804\,s^6+2183964721\,s^4-440479243\,s^2+28950670) \quad \textbf{(50)}$$

```
> taylor_ode := simplify(alpha * diff(ug(s)/s,s,s) + beta * diff(ug
  (s)/s,s) - G);
```

$$taylor\_ode := -\frac{126720}{2573}\sqrt{-s^2+1}\Bigg(\Big(10\,s^7-\frac{108}{5}s^5+\frac{905}{64}s^3-\frac{1619}{640}s\Big)\Big(\frac{d}{ds}ug(s)\Big)+\Big(-10\,s^6+\frac{108}{5}s^4-\frac{905}{64}s^2+\frac{1619}{640}\Big)ug(s)+\frac{1}{21407360}s\,(s^2-1)\Big(-264963686400\,s^{10}+885698150400\,s^8+21407360\,s^5\Big(\frac{d^2}{ds^2}ug(s)\Big)-1087192396416\,s^6-39068432\,s^3\Big(\frac{d^2}{ds^2}ug(s)\Big)+595538545728\,s^4+17895215\,s\Big(\frac{d^2}{ds^2}ug(s)\Big) \quad \textbf{(51)}$$

$$-138273600528\, s^2 + 9727425120 \Big) \Big) \pi\, s$$

> *#Solve Taylor's ODE for the geostrophic flow*

```
> sol_taylor_ode := simplify(dsolve(taylor_ode)):
```

> $sol\_taylor\_ode2 := simplify(subs(\_C1 = 0, sol\_taylor\_ode))$:

> *#Combine magnetostrophic and geostrophic flow and set angular momentum to zero*

```
> u_total_cyl_t := simplify(u_cyl[2] + rhs(sol_taylor_ode2)):
```

> *#calculate constant so the angular momentum is zero*

```
> angular_momentum_t := int(int(int(u_total_cyl_t * s, phi = 0 ..
  2*Pi), z = -sqrt(1-s^2) .. sqrt(1-s^2)), s = 0 .. 1):
>

> C_val_t := solve(Quadrature(int(int(u_total_cyl_t*s, phi = 0 ..
  2*Pi), z = -sqrt(-s^2+1) .. sqrt(-s^2+1)),s=0..1,method=gaussian
  [50]),_C2):
```

> $u\_res\_t := subs(\_C2 = C\_val\_t, sol\_taylor\_ode2)$:

> $plot(rhs(u\_res\_t), s = 0 ..1,)$:

```
Warning, expecting only range variable s in expression -1/33449*
s*(-336*Int((640*s^4-1168*s^2+535)^(264/535)*(788582400*s^10
-2636006400*s^8+3235691656*s^6-1772436148*s^4+411528573*s^2
-28950670)*s^(1619/535)/(1/(s^2-1))^(1/2)*((I*21^(1/2)+80*s^2
-73)/(I*21^(1/2)-80*s^2+73))^(304/1605*I*21^(1/2)),s)*Int(1/s^
(2689/535)/(640*s^4-1168*s^2+535)^(799/535)*(1/(s^2-1))^(1/2)*(
(I*21^(1/2)+80*s^2-73)/(I*21^(1/2)-80*s^2+73))^(-304/1605*I*21^
(1/2)),s)+336*Int((640*s^4-1168*s^2+535)^(264/535)*(788582400*
s^10-2636006400*s^8+3235691656*s^6-1772436148*s^4+411528573*s^2
-28950670)*s^(1619/535)*Int(1/s^(2689/535)/(640*s^4-1168*
s^2+535)^(799/535)*(1/(s^2-1))^(1/2)*((I*21^(1/2)+80*s^2-73)/(I*
21^(1/2)-80*s^2+73))^(-304/1605*I*21^(1/2)),s)/(1/(s^2-1))^(1/2)
*((I*21^(1/2)+80*s^2-73)/(I*21^(1/2)-80*s^2+73))^(304/1605*I*21^
(1/2)),s)-33449*RootOf(Quadrature(277200/2573*s^2*Pi*(-s^2+1)^
(7/2)+1164240/2573*(-s^2+1)^(5/2)*Pi*s^4-1106028/2573*s^2*Pi*(-
s^2+1)^(5/2)+1940400/2573*(-s^2+1)^(3/2)*Pi*s^6-3686760/2573*(-
s^2+1)^(3/2)*Pi*s^4+1617000/2573*s^2*Pi*(-s^2+1)^(3/2)
+1053360/2573*s^8*Pi*(-s^2+1)^(1/2)-3024252/2573*s^6*Pi*(-s^2+1)
^(1/2)+2759064/2573*s^4*Pi*(-s^2+1)^(1/2)-38909700480/33449*s^2*
Pi*(-s^2+1)^(1/2)*Int(1/s^(2689/535)/(640*s^4-1168*s^2+535)^
(799/535)*(1/(s^2-1))^(1/2)*((I*21^(1/2)+80*s^2-73)/(I*21^(1/2)
-80*s^2+73))^(-304/1605*I*21^(1/2)),s)*Int((640*s^4-1168*
s^2+535)^(264/535)*s^(1619/535)/(1/(s^2-1))^(1/2)*((I*21^(1/2)
+80*s^2-73)/(I*21^(1/2)-80*s^2+73))^(304/1605*I*21^(1/2)),s)
+55309440212/33449*s^2*Pi*(-s^2+1)^(1/2)*Int(1/s^(2689/535)/
(640*s^4-1168*s^2+535)^(799/535)*(1/(s^2-1))^(1/2)*((I*21^(1/2)
+80*s^2-73)/(I*21^(1/2)-80*s^2+73))^(-304/1605*I*21^(1/2)),s)*
Int((640*s^4-1168*s^2+535)^(264/535)*s^(2689/535)/(1/(s^2-1))^
(1/2)*((I*21^(1/2)+80*s^2-73)/(I*21^(1/2)-80*s^2+73))^(304/1605*
I*21^(1/2)),s)-2382154182912/33449*s^2*Pi*(-s^2+1)^(1/2)*Int
```

(1/s^(2689/535)/(640*s^4-1168*s^2+535)^(799/535)*(1/(s^2-1))^
(1/2)*((I*21^(1/2)+80*s^2-73)/(I*21^(1/2)-80*s^2+73))^
(-304/1605*I*21^(1/2)),s)*Int((640*s^4-1168*s^2+535)^(264/535)*
s^(3759/535)/(1/(s^2-1))^(1/2)*((I*21^(1/2)+80*s^2-73)/(I*21^
(1/2)-80*s^2+73))^(304/1605*I*21^(1/2)),s)+4348769585664/33449*
s^2*Pi*(-s^2+1)^(1/2)*Int(1/s^(2689/535)/(640*s^4-1168*s^2+535)^
(799/535)*(1/(s^2-1))^(1/2)*((I*21^(1/2)+80*s^2-73)/(I*21^(1/2)
-80*s^2+73))^(-304/1605*I*21^(1/2)),s)*Int((640*s^4-1168*
s^2+535)^(264/535)*s^(4829/535)/(1/(s^2-1))^(1/2)*((I*21^(1/2)
+80*s^2-73)/(I*21^(1/2)-80*s^2+73))^(304/1605*I*21^(1/2)),s)
-3542792601600/33449*s^2*Pi*(-s^2+1)^(1/2)*Int(1/s^(2689/535)/
(640*s^4-1168*s^2+535)^(799/535)*(1/(s^2-1))^(1/2)*((I*21^(1/2)
+80*s^2-73)/(I*21^(1/2)-80*s^2+73))^(-304/1605*I*21^(1/2)),s)*
Int((640*s^4-1168*s^2+535)^(264/535)*s^(5899/535)/(1/(s^2-1))^
(1/2)*((I*21^(1/2)+80*s^2-73)/(I*21^(1/2)-80*s^2+73))^(304/1605*
I*21^(1/2)),s)+1059854745600/33449*s^2*Pi*(-s^2+1)^(1/2)*Int
(1/s^(2689/535)/(640*s^4-1168*s^2+535)^(799/535)*(1/(s^2-1))^
(1/2)*((I*21^(1/2)+80*s^2-73)/(I*21^(1/2)-80*s^2+73))^
(-304/1605*I*21^(1/2)),s)*Int((640*s^4-1168*s^2+535)^(264/535)*
s^(6969/535)/(1/(s^2-1))^(1/2)*((I*21^(1/2)+80*s^2-73)/(I*21^
(1/2)-80*s^2+73))^(304/1605*I*21^(1/2)),s)+4*s^2*Pi*(-s^2+1)^
(1/2)*_Z+38909700480/33449*s^2*Pi*(-s^2+1)^(1/2)*Int((640*s^4
-1168*s^2+535)^(264/535)*s^(1619/535)*Int(1/s^(2689/535)/(640*
s^4-1168*s^2+535)^(799/535)*(1/(s^2-1))^(1/2)*((I*21^(1/2)+80*
s^2-73)/(I*21^(1/2)-80*s^2+73))^(-304/1605*I*21^(1/2)),s)/(1/
(s^2-1))^(1/2)*((I*21^(1/2)+80*s^2-73)/(I*21^(1/2)-80*s^2+73))^
(304/1605*I*21^(1/2)),s)-553094402112/33449*s^2*Pi*(-s^2+1)^
(1/2)*Int((640*s^4-1168*s^2+535)^(264/535)*s^(2689/535)*Int(1/s^
(2689/535)/(640*s^4-1168*s^2+535)^(799/535)*(1/(s^2-1))^(1/2)*(
(I*21^(1/2)+80*s^2-73)/(I*21^(1/2)-80*s^2+73))^(-304/1605*I*21^
(1/2)),s)/(1/(s^2-1))^(1/2)*((I*21^(1/2)+80*s^2-73)/(I*21^(1/2)
-80*s^2+73))^(304/1605*I*21^(1/2)),s)+2382154182912/33449*s^2*
Pi*(-s^2+1)^(1/2)*Int((640*s^4-1168*s^2+535)^(264/535)*s^
(3759/535)*Int(1/s^(2689/535)/(640*s^4-1168*s^2+535)^(799/535)*
(1/(s^2-1))^(1/2)*((I*21^(1/2)+80*s^2-73)/(I*21^(1/2)-80*s^2+73)
)^(-304/1605*I*21^(1/2)),s)/(1/(s^2-1))^(1/2)*((I*21^(1/2)+80*
s^2-73)/(I*21^(1/2)-80*s^2+73))^(304/1605*I*21^(1/2)),s)
-4348769585664/33449*s^2*Pi*(-s^2+1)^(1/2)*Int((640*s^4-1168*
s^2+535)^(264/535)*s^(4829/535)*Int(1/s^(2689/535)/(640*s^4
-1168*s^2+535)^(799/535)*(1/(s^2-1))^(1/2)*((I*21^(1/2)+80*s^2
-73)/(I*21^(1/2)-80*s^2+73))^(-304/1605*I*21^(1/2)),s)/(1/(s^2
-1))^(1/2)*((I*21^(1/2)+80*s^2-73)/(I*21^(1/2)-80*s^2+73))^
(304/1605*I*21^(1/2)),s)+3542792601600/33449*s^2*Pi*(-s^2+1)^
(1/2)*Int((640*s^4-1168*s^2+535)^(264/535)*s^(5899/535)*Int(1/s^
(2689/535)/(640*s^4-1168*s^2+535)^(799/535)*(1/(s^2-1))^(1/2)*(
(I*21^(1/2)+80*s^2-73)/(I*21^(1/2)-80*s^2+73))^(-304/1605*I*21^
(1/2)),s)/(1/(s^2-1))^(1/2)*((I*21^(1/2)+80*s^2-73)/(I*21^(1/2)
-80*s^2+73))^(304/1605*I*21^(1/2)),s)-1059854745600/33449*s^2*
Pi*(-s^2+1)^(1/2)*Int((640*s^4-1168*s^2+535)^(264/535)*s^
(6969/535)*Int(1/s^(2689/535)/(640*s^4-1168*s^2+535)^(799/535)*
(1/(s^2-1))^(1/2)*((I*21^(1/2)+80*s^2-73)/(I*21^(1/2)-80*s^2+73)
)^(-304/1605*I*21^(1/2)),s)/(1/(s^2-1))^(1/2)*((I*21^(1/2)+80*
s^2-73)/(I*21^(1/2)-80*s^2+73))^(304/1605*I*21^(1/2)),s)
-788172/2573*s^2*Pi*(-s^2+1)^(1/2),s = 0 .. 1,method = gaussian
[50]))) to be plotted but found names [method, Quadrature,
gaussian[50]]

```
> #numerical solution For Taylor's ODE
> N := 50; # order of chebysev expansion
                                   N := 50                                          (52)
> M := N + 1 : # order of expansion including logarithmic term
> with(orthopoly) :

> Taylor_chebsum := add(a_t||n·s·T(n, 2 s^2 − 1), n = 1 ..N) :

> Taylor_numerical_approx := eval(Taylor_chebsum) + b_t·s·ln(s) :
      # numerical expansion for geostrophic flow
> G := add(G|| idx, idx = 1 ..4) :

> #Minimise the squared Residual of Taylor's ODE
> Taylor_Residual := simplify(alpha * diff (Taylor_numerical_approx / s, s, s) + beta
      * diff (Taylor_numerical_approx / s, s) - G) :
> Int_Res_t := int( Taylor_Residual^2, s = 0 ..1) :
> Int_Res2 _t := collect(Int_Res_t, seq( a_t|| i, i = 1 ..N), factor) :
> Int_Res2_t := evalf (minimize(Int_Res2 _t, seq( a_t|| i, i = 1 ..N), b_t, location)) :


> Solutionarray_t := {op(op(op(Int_Res2_t[2]))[1])} :




> vars_t := [seq( a_t||i,i=1..N),b_t]:


> Solutionarray2_t := evalf (subs(Solutionarray_t, vars_t)) :

> # calculate constant so angular momentum is zero
> Taylor_numerical_app := Taylor_numerical_approx + s·Cst :
> Taylor_ang_mom := evalf(int(int(int(expand(Taylor_numerical_app * s^2), phi = 0 .. 2 * Pi), z
      = -sqrt(1-s^2) .. sqrt(1-s^2)), s = 0 .. 1)) :
> for i from 1 to N do a_t|| i := (Solutionarray2_t[i]) end do:


> b_t := rhs(Solutionarray_t[M]);
          b_t := -134.76597941691038862712945900466457474747036860964            (53)

> Taylor_C_value := solve(Taylor_ang_mom = 0):
```

> Taylor_numerical_solution := subs(Cst = Taylor_C_value, Taylor_numerical_app) :

> #Plot geostrophic flow solution

> plot(Taylor_numerical_solution, s = 0 ..1);

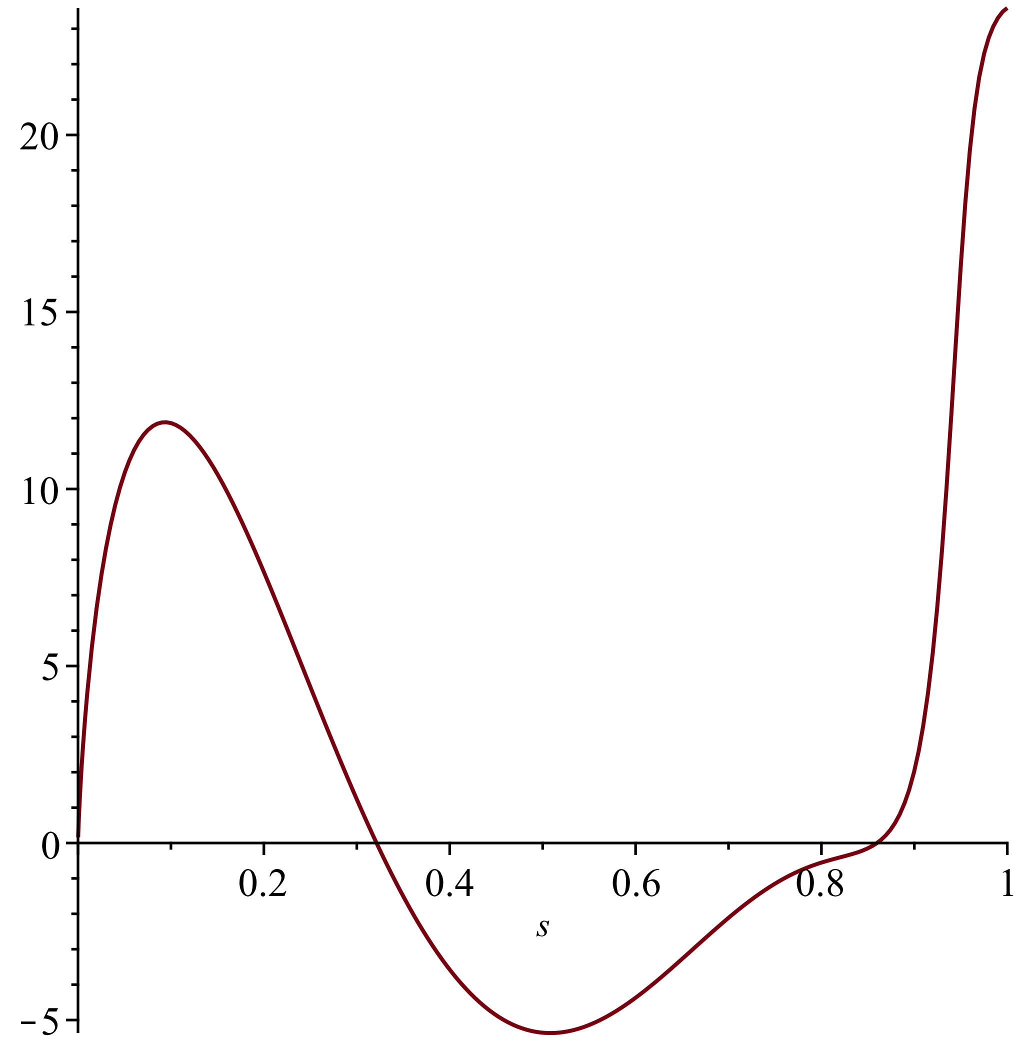

Supplement: ESM5 - ESM8 are PDF versions of the maple worksheets ESM1 - ESM4 respectively. [file rspa20180412supp3.pdf]
