# Supplementary material for: Determination of the instantaneous geostrophic flow within the three-dimensional magnetostrophic regime
Source: Proc Math Phys Eng Sci. 2018 Oct 3;474(2218):20180412. doi: 10.1098/rspa.2018.0412 (PMC6237501; doi:10.1098/rspa.2018.0412)

This worksheet solves the general equation to determine the instantaneous geostrophic flow for all initial magnetic fields including mixed states and non-axisymmetric fields. It uses the potential-based spherical transform method to ensure the boundary conditions are satisfied. A numerical solution is obtained using a least squares scheme.

```
> restart:
> Digits := 30:
> with(orthopoly, P):
  with(VectorCalculus):
  SetCoordinates(cartesian[x,y,z]):
```

> # Define some useful routines

```
> # L1 is theta-factor in spherical harmonic (note that P(l,x) is
     the l-th Legendre polynomial)
> L1 := (l,m) -> if type(m, numeric) then if m <> 0 then sin(theta)
  ^abs(m) * subs(z=cos(theta), diff(P(l,z),z$abs(m))) else P(l, cos
  (theta)) end if else 'L1'(l,m) end if;
```

$$L1 := (l, m) \rightarrow \textbf{if } type(m, numeric) \textbf{ then}$$
$$\textbf{if } m \neq 0 \textbf{ then } \sin(\theta)^{|m|}\, subs\left(z = \cos(\theta), \frac{\partial^{|m|}}{\partial z^{|m|}} P(l, z)\right) \textbf{ else } P(l, \cos(\theta)) \textbf{ end if}$$
$$\textbf{else } 'L1'(l, m) \textbf{ end if} \tag{1}$$

```
> # Lp is phi-factor
> Lp := m -> if type(m, numeric) then if m = 0 then 1 elif m < 0
  then sin(-m*phi) else cos(m*phi) end if else 'Lp'(m) end if;
```

$$Lp := m \rightarrow \textbf{if } type(m, numeric) \textbf{ then}$$
$$\textbf{if } m = 0 \textbf{ then } 1 \textbf{ elif } m < 0 \textbf{ then } \sin(VectorCalculus{:-}\text{`-`}(m\,\phi)) \textbf{ else } \cos(m\,\phi) \textbf{ end if}$$
$$\textbf{else } 'Lp'(m) \textbf{ end if} \tag{2}$$

```
> # norm of L1*Lp is integral of (L1*Lp)^2 over sphere is int((L1*
  Lp)^2 * sin(theta), theta=0..Pi, phi=0..2*Pi)
> L2norm_squared := (l,m) -> int(L1(l,m)^2 * sin(theta), theta=0..
  Pi) * int(Lp(m)^2, phi=0..2*Pi) / (4*Pi);
```

$$L2norm\_squared := (l, m) \rightarrow VectorCalculus{:-}int\left(L1(l, m)^2 \sin(\theta), \theta = 0 ..\pi\right) VectorCalculus{:-}int\left(Lp(m)^2, \phi = 0 ..2\,\pi\right) \frac{1}{4\,\pi} \tag{3}$$

```
> # L2 is Schmidt quasi-normalised spherical harmonic
> L2 := (l,m) -> if type(l,numeric) and type(m,numeric) then L1(l,
  m) * Lp(m) / sqrt(L2norm_squared(l,m)) / sqrt(2*l+1) else 'L2'(l,
  m) end if;
```

$$L2 := (l, m) \rightarrow \textbf{if } type(l, numeric) \textbf{ and } type(m, numeric) \textbf{ then}$$
$$L1(l, m)\, Lp(m) \frac{1}{\sqrt{L2norm\_squared(l, m)}} \frac{1}{\sqrt{2\,l + 1}}$$
$$\textbf{else } 'L2'(l, m) \textbf{ end if} \tag{4}$$

> $\textbf{simplify}(\textbf{eval}(\textbf{L2}(\textbf{l}, \textbf{m}), \{\textbf{l} = \textbf{3}, \textbf{m} = \textbf{1}, \textbf{n} = \textbf{1}\}));$

$$\frac{1}{4} \sqrt{6} \sin(\theta) \left(5 \cos(\theta)^2 - 1\right) \cos(\phi) \tag{5}$$

```
> # Convert an expression in spherical coordinates to Cartesian
```

coordinates.

```
> sph2cart := proc(expr)
    local res;
   res := expand(expr, trig);
   res := subs(cos(phi) = x/(r*sin(theta)), sin(phi) = y/(r*sin
(theta)), res);
   res := subs(cos(theta) = z/r, sin(theta) = sqrt(x^2+y^2)/r,
res);
   res := subs(r = sqrt(x^2+y^2+z^2), res);
   return simplify(res)
end proc:
> # Construct vector field from poloidal and toroidal scalars
scalars2vf := proc(tor_scalar, pol_scalar)
   return simplify(Curl(VectorField(sph2cart(tor_scalar/r) * <x,
y,z>))
                      + Curl(Curl(VectorField(sph2cart(pol_scalar/r)
* <x,y,z>))))
end proc:
```

> *# Define a basis function for the poloidal magnetic field satisfying insulating BCs:*

> Psi_n := (l, n) → r^(l+1) * ((-2*n^2*(l+1) - n*(l+1)*(2*l-1)-l*(2*l+1)) * P(n, 0, l+1/2, 2*r^2-1) + (2*(l+1)*n^2 + (2*l+3)*(l+1)*n + (2*l+1)^2)*P(n-1, 0, l+1/2, 2*r^2-1) + (4*n*l+l*(2*l+1)));

$$Psi\_n := (l, n) \rightarrow r^{l+1} \Big(\big(VectorCalculus{:-}\text{`-`}\big(2\, n^2\, (l+1)\big) + VectorCalculus{:-}\text{`-`}\big(n\, (l+1)\, (2\, l + (-1))\big) + VectorCalculus{:-}\text{`-`}\big(l\, (2\, l+1)\big)\big)\, P\Big(n, 0, l+1\,\frac{1}{2}, 2\, r^2 + (-1)\Big) + \big(2\, (l+1)\, n^2 + (2\, l+3)\, (l+1)\, n + (2\, l+1)^2\big)\, P\Big(n+(-1), 0, l+1\,\frac{1}{2}, 2\, r^2 + (-1)\Big) + 4\, n\, l + l\, (2\, l+1)\Big) \tag{6}$$

> *# Define basis functions for the poloidal flow which vanish at r=1.*

> Chi_n := (l, n) → r^(l+1) * (1-r^2) * P(n-1, 2, l+1/2, 2*r^2-1);

$$Chi\_n := (l, n) \rightarrow r^{l+1} \big(1 + VectorCalculus{:-}\text{`-`}(r^2)\big)\, P\Big(n+(-1), 2, l+1\,\frac{1}{2}, 2\, r^2 + (-1)\Big) \tag{7}$$

> W_n := (l, n) → r^(l+1) * P(n, 0, l+1/2, 2*r^2-1) :

> # Define magnetic field

```
> # choose a number corresponding to the chosen magnetic field 1=
axisymmetric poloidal, 2=nonaxisymmetric toroidal, 3=
nonaxisymmetric poloidal, 4=nonaxisymmetric mixed state
```

> k := 3;

$$k := 3 \tag{8}$$

> **if** *k* = 1 **then B_scalar_tor := 0 : B_scalar_pol :=** ***eval***(**r^2 * (30 * r^4 − 57 * r^2 + 25) * L2(l, m), {l = 1, m = 0, n = 1})**
end if

```
> if k = 2 then B_scalar_tor := simplify(eval(Chi_n(l, n)·L2(l, m), {l = 1, m = 1, n = 1})) :
      B_scalar_pol := 0
  end if
> if k = 3 then B_scalar_tor := 0 : B_scalar_pol := eval(Psi_n(l, n)·L2(l, m), {l = 2, m = 2, n
      = 1})
  end
```

$$B\_scalar\_tor := 0$$

$$B\_scalar\_pol := \frac{1}{10}\, r^3 \left(\frac{315}{2} - \frac{225}{2}\, r^2\right) \sin(\theta)^2 \cos(2\,\phi)\, \sqrt{15}\, \sqrt{5} \tag{9}$$

```
> if k = 4 then B_scalar_tor := eval( Chi_n(l, n)·L2(l, m), {l = 2, m = 1, n = 1}) :
      B_scalar_pol := eval( Psi_n(l, n)·L2(l, m), {l = 2, m = 1, n = 1})
  end if
>  #B_scalar_pol:=0;
```

```
>  B_cart_pol := scalars2vf(0, B_scalar_pol);
```

$$B\_cart\_pol := \left(\frac{945}{2}\sqrt{3}\,x - \frac{675}{2}\sqrt{3}\,x^3 - \frac{1125}{2}\sqrt{3}\,x\,z^2 - \frac{1575}{2}\,x\sqrt{3}\,y^2\right)\bar{e}_x + \left(\frac{1125}{2}\sqrt{3}\,y\,z^2 - \frac{945}{2}\sqrt{3}\,y + \frac{675}{2}\sqrt{3}\,y^3 + \frac{1575}{2}\,y\sqrt{3}\,x^2\right)\bar{e}_y + \left(225\sqrt{3}\,x^2\,z - 225\sqrt{3}\,y^2\,z\right)\bar{e}_z \tag{10}$$

```
>  B_cart_tor := scalars2vf(B_scalar_tor, 0);
```

$$B\_cart\_tor := 0\bar{e}_x \tag{11}$$

```
>  B_sph_pol := simplify(MapToBasis(B_cart_pol, spherical[r, theta, phi]));
```

$$B\_sph\_pol := -\frac{135}{2}\sin(\theta)^2\,(5\,r^2 - 7)\,(2\cos(\phi)^2 - 1)\,\sqrt{3}\,r\bar{e}_r - \frac{45}{2}\cos(\theta)\,\sqrt{3}\,r\sin(\theta)\,(25\,r^2 - 21)\,(2\cos(\phi)^2 - 1)\bar{e}_\theta + \left(\left(1125\,r^3 - 945\,r\right)\sin(\phi)\cos(\phi)\sin(\theta)\,\sqrt{3}\right)\bar{e}_\phi \tag{12}$$

```
>  B_sph_tor := simplify(MapToBasis(B_cart_tor, spherical[r, theta, phi]));
```

$$B\_sph\_tor := 0\bar{e}_r \tag{13}$$

```
>  SetCoordinates(spherical[r, theta, phi]);
```

$$\mathsf{spherical}_{r,\,\theta,\,\phi} \tag{14}$$

```
>  #Scale the magnetic field
>  Scale_pol := sqrt( 1/(4·Pi) · int(int(int(A_pol·B_sph_pol.B_sph_pol·r^2·sin(theta), phi = 0 ..2
       · Pi), theta = 0 .. Pi), r = 0 ..1) );
```

$$Scale\_pol := 6\sqrt{390}\,\sqrt{A\_pol} \tag{15}$$

```
>  if Scale_pol = 0 then A_pol = 0
```

else $A\_pol := simplify(solve(Scale\_pol = 1, A\_pol))$; **end if**;

$$A\_pol := \frac{1}{14040} \quad \textbf{(16)}$$

> $Scale\_tor := \mathrm{sqrt}\left(\frac{1}{4 \cdot \mathrm{Pi}} \cdot int\left(int\left(int\left(A\_tor \cdot B\_sph\_tor.B\_sph\_tor \cdot r^2 \cdot \sin(\mathrm{theta}), \mathrm{phi} = 0..2 \cdot \mathrm{Pi}\right), \mathrm{theta} = 0..\mathrm{Pi}\right), r = 0..1\right)\right)$;

$$Scale\_tor := 0 \quad \textbf{(17)}$$

> **if** $Scale\_tor = 0$ **then** $A\_tor = 0$
> **else** $A\_tor := simplify(solve(Scale\_tor = 1, A\_tor))$ **end if**;

$$A\_tor = 0 \quad \textbf{(18)}$$

> $SetCoordinates(cartesian[x, y, z])$:

> $B\_cart := scalars2vf(\mathrm{sqrt}(A\_tor) \cdot B\_scalar\_tor, \mathrm{sqrt}(A\_pol) \cdot B\_scalar\_pol)$;

$$B\_cart := \left(\frac{63}{104}\sqrt{5}\sqrt{26}\,x - \frac{45}{104}\sqrt{5}\sqrt{26}\,x^3 - \frac{75}{104}\sqrt{5}\sqrt{26}\,x\,z^2 - \frac{105}{104}\,x\sqrt{5}\sqrt{26}\,y^2\right)\bar{e}_x + \left(\frac{75}{104}\sqrt{5}\sqrt{26}\,y\,z^2 - \frac{63}{104}\sqrt{5}\sqrt{26}\,y + \frac{45}{104}\sqrt{5}\sqrt{26}\,y^3 + \frac{105}{104}\,y\sqrt{5}\sqrt{26}\,x^2\right)\bar{e}_y + \left(\frac{15}{52}\sqrt{5}\sqrt{26}\,x^2\,z - \frac{15}{52}\sqrt{5}\sqrt{26}\,y^2\,z\right)\bar{e}_z \quad \textbf{(19)}$$

> **B_sph := simplify(MapToBasis(B_cart, spherical[r,theta,phi]));**

$$B\_sph := -\frac{9}{104}\sin(\theta)^2\left(5\,r^2 - 7\right)\left(2\cos(\phi)^2 - 1\right)\sqrt{5}\sqrt{26}\,r\bar{e}_r - \frac{3}{104}\cos(\theta)\sqrt{5}\sqrt{26}\,r\sin(\theta)\left(25\,r^2 - 21\right)\left(2\cos(\phi)^2 - 1\right)\bar{e}_\theta + \frac{75}{52}\sqrt{26}\sin(\theta)\sin(\phi)\sqrt{5}\cos(\phi)\,r\left(r^2 - \frac{21}{25}\right)\bar{e}_\phi \quad \textbf{(20)}$$

> # Compute rhs of magnetostrophic equation

```
> # slaved equation is Omega cross u = -div(p) + curl(B) cross B,
  we ignore the pressure
> RHS := CrossProduct(Curl(B_cart), B_cart): simplify(RHS);
```

$$-\frac{1575}{104}\,x\left(35\,x^2\,y^2+5\,x^2\,z^2+15\,y^4+20\,y^2\,z^2-21\,y^2\right)\bar{e}_x-\frac{1575}{104}\,y\left(15\,x^4+35\,x^2\,y^2+20\,x^2\,z^2+5\,y^2\,z^2-21\,x^2\right)\bar{e}_y-\frac{1575}{208}\,z\left(15\,x^4+70\,x^2\,y^2+25\,x^2\,z^2+15\,y^4+25\,y^2\,z^2-21\,x^2-21\,y^2\right)\bar{e}_z \quad (21)$$

```
> map(factor, simplify(MapToBasis(RHS, spherical[r,theta,phi])));
  # for comparison
```

$$-\frac{1575}{208}\sin(\theta)^2\,(25\,r^2-21)\,\left(4\cos(\theta)^2\cos(\phi)^4-4\cos(\phi)^2\cos(\theta)^2-4\cos(\phi)^4+\cos(\theta)^2+4\cos(\phi)^2\right)\,r^3\bar{e}_r+\frac{4725}{208}\cos(\theta)\,\sin(\theta)^3\,(5\,r^2-7)\,\left(2\cos(\phi)^2-1\right)^2\,r^3\bar{e}_\theta-\frac{4725}{104}\sin(\phi)\,\sin(\theta)^3\cos(\phi)\,(5\,r^2-7)\,\left(2\cos(\phi)^2-1\right)\,r^3\bar{e}_\phi \quad (22)$$

> # Construct basis for u

```
> # Let N be the degree of B. Then the degree of curl(B) cross B is
  (N-1) + N = 2N-1. Thus the degree of u is also 2N-1. Thus, the
  poloidal scalar has degree 2N+1 (we have to undo two curls) and
  the toroidal scalar has degree 2N. Furthermore, the m-degree of
  curl(B) cross B is twice the m-degree of B.
> B_degree := max(seq(degree(B_cart[idx], {x,y,z}), idx = 1 .. 3));
```

$$B\_degree := 3 \quad (23)$$

```
> Max_pol_degree := 2 * B_degree + 1; Max_tor_degree := 2 *
  B_degree;
```

$$Max\_pol\_degree := 7$$
$$Max\_tor\_degree := 6 \quad (24)$$

```
> Max_m_degree := 2 * max(seq(degree(expand(B_sph[idx], trig), {cos
  (phi), sin(phi)}), idx = 1 .. 3));
```

$$Max\_m\_degree := 4 \quad (25)$$

```
> # For the particular example here, certain modes are zero, but we
  will not exploit this knowledge.
> # Also note that unless B is a Taylor state, there will be no
  solution to the magnetostrophic equation.

> u_scalar_pol := add(add(add(S[l,m,n] * L2(l,m) * Chi_n(l,n),
                            m = -min(l, Max_m_degree) .. min(l,
  Max_m_degree)),
                             n = 1 .. (Max_pol_degree - l + 1) /
  2),
                             l = 1 .. Max_pol_degree):
> u_scalar_tor := add(add(add(T[l,m,n] * L2(l,m) * W_n(l,n),
                            m = -min(l, Max_m_degree) .. min(l,
```

```
Max_m_degree)),
                        n = 0 .. floor((Max_tor_degree - l + 1)/2)),
                        l = 1 .. Max_tor_degree):
> coeff(u_scalar_pol, S[2,-2,2]);
```

$$\frac{1}{10}\sin(\theta)^2\sin(2\,\phi)\sqrt{15}\sqrt{5}\,r^3\left(-r^2+1\right)\left(-\frac{7}{2}+\frac{13}{2}\,r^2\right) \qquad (26)$$

```
> # u is poloidal part (curl^2 of scalar times hat r) + toroidal part (curl of scalar times hat r)
> u_cart := scalars2vf(u_scalar_tor, u_scalar_pol):
> variables := indets(u_cart, indexed);
```

$$variables := \{S_{1,-1,1}, S_{1,-1,2}, S_{1,-1,3}, S_{1,0,1}, S_{1,0,2}, S_{1,0,3}, S_{1,1,1}, S_{1,1,2}, S_{1,1,3}, S_{2,-2,1}, S_{2,-2,2}, S_{2,-2,3}, S_{2,-1,1}, S_{2,-1,2}, S_{2,-1,3}, S_{2,0,1}, S_{2,0,2}, S_{2,0,3}, S_{2,1,1}, S_{2,1,2}, S_{2,1,3}, S_{2,2,1}, S_{2,2,2}, S_{2,2,3}, S_{3,-3,1}, S_{3,-3,2}, S_{3,-2,1}, S_{3,-2,2}, S_{3,-1,1}, S_{3,-1,2}, S_{3,0,1}, S_{3,0,2}, S_{3,1,1}, S_{3,1,2}, S_{3,2,1}, S_{3,2,2}, S_{3,3,1}, S_{3,3,2}, S_{4,-4,1}, S_{4,-4,2}, S_{4,-3,1}, S_{4,-3,2}, S_{4,-2,1}, S_{4,-2,2}, S_{4,-1,1}, S_{4,-1,2}, S_{4,0,1}, S_{4,0,2}, S_{4,1,1}, S_{4,1,2}, S_{4,2,1}, S_{4,2,2}, S_{4,3,1}, S_{4,3,2}, S_{4,4,1}, S_{4,4,2}, S_{5,-4,1}, S_{5,-3,1}, S_{5,-2,1}, S_{5,-1,1}, S_{5,0,1}, S_{5,1,1}, S_{5,2,1}, S_{5,3,1}, S_{5,4,1}, S_{6,-4,1}, S_{6,-3,1}, S_{6,-2,1}, S_{6,-1,1}, S_{6,0,1}, S_{6,1,1}, S_{6,2,1}, S_{6,3,1}, S_{6,4,1}, T_{1,-1,0}, T_{1,-1,1}, T_{1,-1,2}, T_{1,-1,3}, T_{1,0,0}, T_{1,0,1}, T_{1,0,2}, T_{1,0,3}, T_{1,1,0}, T_{1,1,1}, T_{1,1,2}, T_{1,1,3}, T_{2,-2,0}, T_{2,-2,1}, T_{2,-2,2}, T_{2,-1,0}, T_{2,-1,1}, T_{2,-1,2}, T_{2,0,0}, T_{2,0,1}, T_{2,0,2}, T_{2,1,0}, T_{2,1,1}, T_{2,1,2}, T_{2,2,0}, T_{2,2,1}, T_{2,2,2}, T_{3,-3,0}, T_{3,-3,1}, T_{3,-3,2}, T_{3,-2,0}, T_{3,-2,1}, T_{3,-2,2}, T_{3,-1,0}, T_{3,-1,1}, T_{3,-1,2}, T_{3,0,0}, T_{3,0,1}, T_{3,0,2}, T_{3,1,0}, T_{3,1,1}, T_{3,1,2}, T_{3,2,0}, T_{3,2,1}, T_{3,2,2}, T_{3,3,0}, T_{3,3,1}, T_{3,3,2}, T_{4,-4,0}, T_{4,-4,1}, T_{4,-3,0}, T_{4,-3,1}, T_{4,-2,0}, T_{4,-2,1}, T_{4,-1,0}, T_{4,-1,1}, T_{4,0,0}, T_{4,0,1}, T_{4,1,0}, T_{4,1,1}, T_{4,2,0}, T_{4,2,1}, T_{4,3,0}, T_{4,3,1}, T_{4,4,0}, T_{4,4,1}, T_{5,-4,0}, T_{5,-4,1}, T_{5,-3,0}, T_{5,-3,1}, T_{5,-2,0}, T_{5,-2,1}, T_{5,-1,0}, T_{5,-1,1}, T_{5,0,0}, T_{5,0,1}, T_{5,1,0}, T_{5,1,1}, T_{5,2,0}, T_{5,2,1}, T_{5,3,0}, T_{5,3,1}, T_{5,4,0}, T_{5,4,1}, T_{6,-4,0}, T_{6,-3,0}, T_{6,-2,0}, T_{6,-1,0}, T_{6,0,0}, T_{6,1,0}, T_{6,2,0}, T_{6,3,0}, T_{6,4,0}\} \qquad (27)$$

```
> simplify(MapToBasis(coeff(u_cart, S[2,-2,2], 1), spherical[r,theta,phi]));
```

$$\left(\left(-39\,r^5+60\,r^3-21\,r\right)\sin(\phi)\cos(\phi)\sin(\theta)^2\sqrt{3}\right)\bar{e}_r+\left(\left(-91\,r^5+100\,r^3-21\,r\right)\sin(\phi)\cos(\phi)\sin(\theta)\cos(\theta)\sqrt{3}\right)\bar{e}_\theta-\frac{1}{2}\sqrt{3}\,r\sin(\theta)\left(91\,r^4-100\,r^2+21\right)\left(2\cos(\phi)^2-1\right)\bar{e}_\phi \qquad (28)$$

> # Compute lhs of magnetostrophic equation

```
> # slaved equation is Omega cross u = -div(p) + curl(B) cross B
> Omega_vec := VectorField([0,0,1]);  # rotation vector in cartesian coordinates
```

$$Omega\_vec := \bar{e}_z \qquad (29)$$

```
> LHS := CrossProduct(Omega_vec, u_cart):
```

> # Solve magnetostrophic equation for basis coefficients

```
> # take the curl of slaved equation; pressure drops out
> eqn := simplify(Curl(LHS - RHS));
```

$$eqn := \Big(\frac{105}{16} T_{1,\,-1,\,3} + 1170\, z^5 S_{1,\,1,\,3} - \frac{35}{8} T_{1,\,-1,\,2} + \frac{5}{2} T_{1,\,-1,\,1} - T_{1,\,-1,\,0} + \frac{1}{416} \Big(\Big(\tag{30}$$

$$-2574 \sqrt{7} \Big(x^6 S_{6,\,3,\,1} + \Big(\frac{1}{99} T_{6,\,-2,\,0} + \frac{24}{11} S_{6,\,-3,\,1} y + \frac{10}{3} S_{6,\,2,\,1} z \Big) x^5 + \Big(\frac{5}{11} S_{6,\,3,\,1} y^2$$

$$+ \Big(\frac{130}{33} S_{6,\,-2,\,1} z + \frac{17}{99} T_{6,\,2,\,0} \Big) y - \frac{35}{33} S_{6,\,3,\,1} + \frac{5}{11} S_{6,\,3,\,1} z^2 - \frac{2}{11} z\, T_{6,\,-3,\,0} \Big) x^4$$

$$+ \Big(\frac{40}{11} S_{6,\,-3,\,1} y^3 + \Big(\frac{60}{11} S_{6,\,2,\,1} z + \frac{38}{99} T_{6,\,-2,\,0} \Big) y^2 + \Big(\frac{60}{11} S_{6,\,-3,\,1} z^2 - \frac{8}{33} z\, T_{6,\,3,\,0}$$

$$- \frac{28}{11} S_{6,\,-3,\,1} \Big) y + \frac{320}{33} \Big(S_{6,\,2,\,1} z^2 - \frac{1}{20} z\, T_{6,\,-2,\,0} - \frac{7}{15} S_{6,\,2,\,1} \Big) z \Big) x^3 + \Big($$

$$-\frac{15}{11} S_{6,\,3,\,1} y^4 + \Big(\frac{20}{3} S_{6,\,-2,\,1} z - \frac{2}{99} T_{6,\,2,\,0} \Big) y^3 + \Big(-\frac{150}{11} S_{6,\,3,\,1} z^2 - \frac{20}{11} z\, T_{6,\,-3,\,0}$$

$$+ \frac{14}{11} S_{6,\,3,\,1} \Big) y^2 + \frac{640}{33} \Big(S_{6,\,-2,\,1} z^2 - \frac{1}{20} z\, T_{6,\,2,\,0} - \frac{7}{20} S_{6,\,-2,\,1} \Big) z\, y - \frac{120}{11} \Big(S_{6,\,3,\,1} z^2$$

$$- \frac{4}{45} z\, T_{6,\,-3,\,0} - \frac{7}{15} S_{6,\,3,\,1} \Big) z^2 \Big) x^2 + \Big(\frac{16}{11} S_{6,\,-3,\,1} y^5 + \Big(\frac{70}{33} S_{6,\,2,\,1} z$$

$$+ \frac{37}{99} T_{6,\,-2,\,0} \Big) y^4 + \Big(-\frac{140}{11} S_{6,\,-3,\,1} z^2 + \frac{24}{11} z\, T_{6,\,3,\,0} - \frac{28}{33} S_{6,\,-3,\,1} \Big) y^3$$

$$- \frac{320}{33} \Big(S_{6,\,2,\,1} z + \frac{7}{20} T_{6,\,-2,\,0} \Big) z^2 y^2 - \frac{240}{11} \Big(S_{6,\,-3,\,1} z^2 + \frac{4}{45} z\, T_{6,\,3,\,0}$$

$$- \frac{7}{15} S_{6,\,-3,\,1} \Big) z^2 y - \frac{96}{11} \Big(S_{6,\,2,\,1} z^2 - \frac{5}{54} z\, T_{6,\,-2,\,0} - \frac{14}{27} S_{6,\,2,\,1} \Big) z^3 \Big) x + \frac{30}{11} y \Big($$

$$-\frac{3}{10} S_{6,\,3,\,1} y^5 + \Big(S_{6,\,-2,\,1} z - \frac{19}{270} T_{6,\,2,\,0} \Big) y^4 + \Big(\frac{3}{2} S_{6,\,3,\,1} z^2 + \frac{13}{45} z\, T_{6,\,-3,\,0}$$

$$+ \frac{7}{30} S_{6,\,3,\,1} \Big) y^3 - \frac{112}{135} \Big(-\frac{6}{7} z\, T_{6,\,2,\,0} + S_{6,\,-2,\,1} \Big) z\, y^2 + 4 \Big(S_{6,\,3,\,1} z^2 - \frac{4}{45} z\, T_{6,\,-3,\,0}$$

$$- \frac{7}{15} S_{6,\,3,\,1} \Big) z^2 y - \frac{16}{5} \Big(S_{6,\,-2,\,1} z^2 + \frac{5}{54} z\, T_{6,\,2,\,0} - \frac{14}{27} S_{6,\,-2,\,1} \Big) z^3 \Big) \Big) \sqrt{2}$$

$$+ \Big(\big(3120\, z\, T_{5,\,-2,\,1} - 2288\, S_{5,\,2,\,1} \big) x^5 - 2912 \Big(-\frac{75}{28} T_{5,\,2,\,1} z + S_{5,\,-2,\,1} \Big) y\, x^4$$

$$+ \Big(\big(28080\, z\, T_{5,\,-2,\,1} - 3328\, S_{5,\,2,\,1} \big) y^2 - 6240\, T_{5,\,-2,\,1} z^3 - 6240\, S_{5,\,2,\,1} z^2 + \big($$

$$-2704\, T_{5,\,-2,\,1} + 416\, T_{5,\,-2,\,0} \big) z + 2496\, S_{5,\,2,\,1} \Big) x^3 - 4576\, y \Big(\Big(\frac{15}{11} T_{5,\,2,\,1} z + S_{5,\,-2,\,1} \Big) y^2$$

$$- \frac{75}{11} T_{5,\,2,\,1} z^3 + \frac{42}{11} S_{5,\,-2,\,1} z^2 + \Big(\frac{39}{22} T_{5,\,2,\,1} - \frac{3}{11} T_{5,\,2,\,0} \Big) z - \frac{9}{11} S_{5,\,-2,\,1} \Big) x^2$$

$$+ \Big(\big(24960\, z\, T_{5,\,-2,\,1} - 1040\, S_{5,\,2,\,1} \big) y^4 + 16224 \Big(\frac{35}{13} T_{5,\,-2,\,1} z^2 + S_{5,\,2,\,1} z - \frac{3}{2} T_{5,\,-2,\,1}$$

$$+ \frac{3}{13} T_{5,\,-2,\,0} \Big) z\, y^2 + 16640 \Big(-\frac{9}{8} T_{5,\,-2,\,1} z^3 + S_{5,\,2,\,1} z^2 + \Big(\frac{13}{20} T_{5,\,-2,\,1}$$

$$- \frac{1}{10} T_{5, -2, 0}\Big) z - \frac{9}{20} S_{5, 2, 1}\Big) z^2\Big) x - 1664 y \bigg(\Big(\Big(\frac{135}{16} T_{5, 2, 1} z + S_{5, -2, 1}\Big) y^4$$

$$+ \Big(\frac{45}{4} T_{5, 2, 1} z^3 - 3 S_{5, -2, 1} z^2 + \Big(-\frac{65}{8} T_{5, 2, 1} + \frac{5}{4} T_{5, 2, 0}\Big) z - \frac{3}{4} S_{5, -2, 1}\Big) y^2$$

$$- 10 \Big(\frac{9}{8} T_{5, 2, 1} z^3 + S_{5, -2, 1} z^2 + \Big(-\frac{13}{20} T_{5, 2, 1} + \frac{1}{10} T_{5, 2, 0}\Big) z - \frac{9}{20} S_{5, -2, 1}\Big) z^2\Big)\bigg)$$

$$\sqrt{7} - 390 x^6 T_{5, -1, 1} + \big(-10920 y T_{5, 1, 1} - 20280 z T_{3, -2, 2} + 6240 S_{3, 2, 2}\big) x^5 + \big($$

$$-12090 y^2 T_{5, -1, 1} + \big(40560 z T_{3, 2, 2} - 12480 S_{3, -2, 2}\big) y + 12870 z^2 T_{5, -1, 1}$$

$$- 13312 z S_{5, 1, 1} + 338 T_{5, -1, 1} - 52 T_{5, -1, 0}\big) x^4 + \big(-21840 y^3 T_{5, 1, 1} + 49920 y^2 S_{3, 2, 2}$$

$$+ \big(32760 z^2 T_{5, 1, 1} - 4160 z S_{5, -1, 1} - 1456 T_{5, 1, 0} + 9464 T_{5, 1, 1}\big) y - 81120 z^3 T_{3, -2, 2}$$

$$+ 93600 z^2 S_{3, 2, 2} + \big(-4576 T_{3, -2, 1} + 29744 T_{3, -2, 2}\big) z + 1248 S_{3, 2, 1} - 14976 S_{3, 2, 2}\big) x^3$$

$$+ \Big(-23010 y^4 T_{5, -1, 1} + \big(40560 z T_{3, 2, 2} + 12480 S_{3, -2, 2}\big) y^3 + \big(58500 z^2 T_{5, -1, 1}$$

$$- 22464 z S_{5, 1, 1} - 1560 T_{5, -1, 0} + 10140 T_{5, -1, 1}\big) y^2 + 6864 \Big(\frac{325}{22} T_{3, 2, 2} z^2$$

$$+ \frac{60}{11} z S_{3, -2, 2} + T_{3, 2, 1} - \frac{13}{2} T_{3, 2, 2}\Big) z y + 7800 T_{5, -1, 1} z^4 - 63232 S_{5, 1, 1} z^3 + \big($$

$$-12168 T_{5, -1, 1} + 1872 T_{5, -1, 0}\big) z^2 + 22464 z S_{5, 1, 1}\Big) x^2 + \Big(-10920 y^5 T_{5, 1, 1}$$

$$+ \big(20280 z T_{3, -2, 2} + 43680 S_{3, 2, 2}\big) y^4 + \big(32760 z^2 T_{5, 1, 1} - 4160 z S_{5, -1, 1} - 1456 T_{5, 1, 0}$$

$$+ 9464 T_{5, 1, 1}\big) y^3 + \big(-40560 z^3 T_{3, -2, 2} + 205920 z^2 S_{3, 2, 2} + 3744 S_{3, 2, 1}$$

$$- 44928 S_{3, 2, 2}\big) y^2 - 56576 \Big(-\frac{525}{272} T_{5, 1, 1} z^3 + S_{5, -1, 1} z^2 + \Big(\frac{273}{272} T_{5, 1, 1}$$

$$- \frac{21}{136} T_{5, 1, 0}\Big) z - \frac{9}{34} S_{5, -1, 1}\Big) z y - 60840 z^5 T_{3, -2, 2} + 124800 z^4 S_{3, 2, 2} + \big($$

$$-9152 T_{3, -2, 1} + 59488 T_{3, -2, 2}\big) z^3 + \big(7488 S_{3, 2, 1} - 89856 S_{3, 2, 2}\big) z^2 + \big(3744 T_{3, -2, 1}$$

$$- 10296 T_{3, -2, 2} - 832 T_{3, -2, 0}\big) z - 1664 S_{3, 2, 1} + 7488 S_{3, 2, 2}\Big) x - 11310 y^6 T_{5, -1, 1}$$

$$+ 24960 y^5 S_{3, -2, 2} + \big(45630 z^2 T_{5, -1, 1} - 9152 z S_{5, 1, 1} - 1508 T_{5, -1, 0} + 9802 T_{5, -1, 1}\big) y^4$$

$$+ \big(60840 T_{3, 2, 2} z^3 + 149760 S_{3, -2, 2} z^2 + \big(2288 T_{3, 2, 1} - 14872 T_{3, 2, 2}\big) z + 2496 S_{3, -2, 1}$$

$$- 29952 S_{3, -2, 2}\big) y^3 - 6656 \Big(-\frac{1125}{64} T_{5, -1, 1} z^3 + S_{5, 1, 1} z^2 + \Big(\frac{663}{64} T_{5, -1, 1}$$

$$- \frac{51}{32} T_{5, -1, 0}\Big) z - \frac{9}{8} S_{5, 1, 1}\Big) z y^2 + \big(60840 T_{3, 2, 2} z^5 + 124800 S_{3, -2, 2} z^4 + \big(9152 T_{3, 2, 1}$$

$$-59488\,T_{3,2,2}\big)z^3+\big(7488\,S_{3,-2,1}-89856\,S_{3,-2,2}\big)z^2+\big(-3744\,T_{3,2,1}+10296\,T_{3,2,2}$$

$$+832\,T_{3,2,0}\big)z-1664\,S_{3,-2,1}+7488\,S_{3,-2,2}\big)y+19968\left(-\frac{35}{32}\,T_{5,-1,1}z^3+S_{5,1,1}z^2\right.$$

$$\left.+\left(\frac{65}{96}\,T_{5,-1,1}-\frac{5}{48}\,T_{5,-1,0}\right)z-\frac{1}{2}\,S_{5,1,1}\right)z^3\Big)\sqrt{3}+\Big(\Big(\big(9724\,S_{4,3,2}$$

$$+585\,T_{5,-3,1}\big)x^6+5304\,y\left(S_{4,-3,2}+\frac{15}{34}\,T_{5,3,1}\right)x^5+\big(\big(52156\,S_{4,3,2}$$

$$+11115\,T_{5,-3,1}\big)y^2+\big(100776\,S_{4,3,2}-12285\,T_{5,-3,1}\big)z^2+\big(3744\,S_{5,3,1}$$

$$-4056\,T_{4,-3,1}\big)z+1352\,S_{4,3,1}-18928\,S_{4,3,2}-507\,T_{5,-3,1}+78\,T_{5,-3,0}\big)x^4$$

$$+67184\,y\left(\left(S_{4,-3,2}-\frac{45}{323}\,T_{5,3,1}\right)y^2+\left(\frac{45}{19}\,S_{4,-3,2}+\frac{675}{1292}\,T_{5,3,1}\right)z^2\right.$$

$$+\left(\frac{12}{323}\,S_{5,-3,1}+\frac{91}{646}\,T_{4,3,1}\right)z-\frac{126}{323}\,S_{4,-3,2}-\frac{39}{1292}\,T_{5,3,1}+\frac{3}{646}\,T_{5,3,0}$$

$$\left.+\frac{9}{323}\,S_{4,-3,1}\right)x^3+\big(\big(18564\,S_{4,3,2}+6435\,T_{5,-3,1}\big)y^4+\big(\big(127296\,S_{4,3,2}$$

$$+31590\,T_{5,-3,1}\big)z^2+\big(14976\,S_{5,3,1}+4056\,T_{4,-3,1}\big)z+2496\,S_{4,3,1}-34944\,S_{4,3,2}$$

$$-9126\,T_{5,-3,1}+1404\,T_{5,-3,0}\big)y^2+\big(119340\,S_{4,3,2}-23400\,T_{5,-3,1}\big)z^4+\big(19968\,S_{5,3,1}$$

$$-8112\,T_{4,-3,1}\big)z^3+\big(6552\,S_{4,3,1}-91728\,S_{4,3,2}+12168\,T_{5,-3,1}-1872\,T_{5,-3,0}\big)z^2$$

$$+\big(-7488\,S_{5,3,1}+3432\,T_{4,-3,1}-624\,T_{4,-3,0}\big)z-1560\,S_{4,3,1}+8580\,S_{4,3,2}\big)x^2$$

$$+61880\left(\left(S_{4,-3,2}-\frac{45}{238}\,T_{5,3,1}\right)y^4+\left(\left(\frac{186}{35}\,S_{4,-3,2}-\frac{27}{238}\,T_{5,3,1}\right)z^2\right.\right.$$

$$+\left(\frac{216}{595}\,S_{5,-3,1}+\frac{39}{595}\,T_{4,3,1}\right)z-\frac{20}{17}\,S_{4,-3,2}+\frac{39}{238}\,T_{5,3,1}-\frac{3}{119}\,T_{5,3,0}$$

$$\left.+\frac{10}{119}\,S_{4,-3,1}\right)y^2+\left(\frac{27}{7}\,S_{4,-3,2}+\frac{90}{119}\,T_{5,3,1}\right)z^4+\left(\frac{384}{595}\,S_{5,-3,1}\right.$$

$$\left.+\frac{156}{595}\,T_{4,3,1}\right)z^3+\left(-\frac{252}{85}\,S_{4,-3,2}-\frac{234}{595}\,T_{5,3,1}+\frac{36}{595}\,T_{5,3,0}+\frac{18}{85}\,S_{4,-3,1}\right)z^2+\Big($$

$$\left.-\frac{144}{595}\,S_{5,-3,1}-\frac{66}{595}\,T_{4,3,1}+\frac{12}{595}\,T_{4,3,0}\Big)z+\frac{33}{119}\,S_{4,-3,2}-\frac{6}{119}\,S_{4,-3,1}\right)y\,x$$

$$-2184\,y^2\left(\left(\frac{153}{14}\,S_{4,3,2}+\frac{15}{8}\,T_{5,-3,1}\right)y^4+\left(\left(\frac{459}{7}\,S_{4,3,2}-\frac{45}{56}\,T_{5,-3,1}\right)z^2\right.\right.$$

$$\left.+\left(4\,S_{5,3,1}-\frac{26}{21}\,T_{4,-3,1}\right)z+S_{4,3,1}-14\,S_{4,3,2}-\frac{13}{8}\,T_{5,-3,1}+\frac{1}{4}\,T_{5,-3,0}\right)y^2$$

$$+\left(\frac{765}{14}\,S_{4,3,2}-\frac{75}{7}\,T_{5,-3,1}\right)z^4+\left(\frac{64}{7}\,S_{5,3,1}-\frac{26}{7}\,T_{4,-3,1}\right)z^3+\Big(3\,S_{4,3,1}$$

$$-42\,S_{4,3,2}+\frac{39}{7}\,T_{5,-3,1}-\frac{6}{7}\,T_{5,-3,0}\Big)z^2+\Big(-\frac{24}{7}\,S_{5,3,1}+\frac{11}{7}\,T_{4,-3,1}$$

$$- \frac{2}{7} T_{4,\,-3,\,0}\Big) z - \frac{5}{7} S_{4,\,3,\,1} + \frac{55}{14} S_{4,\,3,\,2}\Big)\Big) \sqrt{7} + \left(-29172\, S_{4,\,1,\,2} - 7605\, T_{3,\,-3,\,2}\right) x^6$$

$$- 5304\, y \left(S_{4,\,-1,\,2} - \frac{195}{68} T_{3,\,3,\,2}\right) x^5 + \Big(\left(-82212\, S_{4,\,1,\,2} - 7605\, T_{3,\,-3,\,2}\right) y^2 + \Big($$

$$-376584\, S_{4,\,1,\,2} - 45630\, T_{3,\,-3,\,2}\Big) z^2 + \left(4056\, T_{4,\,-1,\,1} + 56160\, S_{3,\,3,\,2}\right) z - 4056\, S_{4,\,1,\,1}$$

$$+ 56784\, S_{4,\,1,\,2} - 1716\, T_{3,\,-3,\,1} + 11154\, T_{3,\,-3,\,2}\Big) x^4 - 1872\, y \Big(\Big(\frac{17}{3} S_{4,\,-1,\,2}$$

$$- \frac{65}{4} T_{3,\,3,\,2}\Big) y^2 + \left(\frac{527}{3} S_{4,\,-1,\,2} - \frac{195}{4} T_{3,\,3,\,2}\right) z^2 + \left(-\frac{65}{6} T_{4,\,1,\,1} - 50\, S_{3,\,-3,\,2}\right) z$$

$$+ S_{4,\,-1,\,1} - 14\, S_{4,\,-1,\,2} - \frac{11}{6} T_{3,\,3,\,1} + \frac{143}{12} T_{3,\,3,\,2}\Big) x^3 + \Big(\Big(-76908\, S_{4,\,1,\,2}$$

$$+ 7605\, T_{3,\,-3,\,2}\Big) y^4 + \Big(-424320\, S_{4,\,1,\,2} z^2 + \left(28392\, T_{4,\,-1,\,1} + 56160\, S_{3,\,3,\,2}\right) z$$

$$- 6240\, S_{4,\,1,\,1} + 87360\, S_{4,\,1,\,2}\Big) y^2 + \left(-322660\, S_{4,\,1,\,2} - 38025\, T_{3,\,-3,\,2}\right) z^4 + \Big($$

$$-2704\, T_{4,\,-1,\,1} + 74880\, S_{3,\,3,\,2}\Big) z^3 + \Big(-20904\, S_{4,\,1,\,1} + 292656\, S_{4,\,1,\,2} - 5148\, T_{3,\,-3,\,1}$$

$$+ 33462\, T_{3,\,-3,\,2}\Big) z^2 + \left(-3432\, T_{4,\,-1,\,1} + 624\, T_{4,\,-1,\,0} + 3744\, S_{3,\,3,\,1} - 44928\, S_{3,\,3,\,2}\right) z$$

$$+ 4680\, S_{4,\,1,\,1} - 25740\, S_{4,\,1,\,2} + 1404\, T_{3,\,-3,\,1} - 3861\, T_{3,\,-3,\,2} - 312\, T_{3,\,-3,\,0}\Big) x^2$$

$$- 1872\, y \Big(\Big(\frac{17}{6} S_{4,\,-1,\,2} - \frac{65}{8} T_{3,\,3,\,2}\Big) y^4 + \Big(\Big(\frac{527}{3} S_{4,\,-1,\,2} - \frac{195}{4} T_{3,\,3,\,2}\Big) z^2 + \Big($$

$$-\frac{65}{6} T_{4,\,1,\,1} - 90\, S_{3,\,-3,\,2}\Big) z + S_{4,\,-1,\,1} - 14\, S_{4,\,-1,\,2} - \frac{11}{6} T_{3,\,3,\,1} + \frac{143}{12} T_{3,\,3,\,2}\Big) y^2$$

$$+ \left(\frac{5015}{18} S_{4,\,-1,\,2} - \frac{325}{8} T_{3,\,3,\,2}\right) z^4 + \left(-26\, T_{4,\,1,\,1} - 80\, S_{3,\,-3,\,2}\right) z^3 + \Big(\frac{37}{3} S_{4,\,-1,\,1}$$

$$- \frac{11}{2} T_{3,\,3,\,1} - \frac{518}{3} S_{4,\,-1,\,2} + \frac{143}{4} T_{3,\,3,\,2}\Big) z^2 + \Big(-2\, T_{4,\,1,\,0} - 4\, S_{3,\,-3,\,1} + 11\, T_{4,\,1,\,1}$$

$$+ 48\, S_{3,\,-3,\,2}\Big) z - \frac{33}{8} T_{3,\,3,\,2} - \frac{1}{3} T_{3,\,3,\,0} + \frac{3}{2} T_{3,\,3,\,1} - \frac{5}{3} S_{4,\,-1,\,1} + \frac{55}{6} S_{4,\,-1,\,2}\Big) x + \Big($$

$$-23868\, S_{4,\,1,\,2} + 7605\, T_{3,\,-3,\,2}\Big) y^6 + \Big(\left(-47736\, S_{4,\,1,\,2} + 45630\, T_{3,\,-3,\,2}\right) z^2$$

$$+ \left(24336\, T_{4,\,-1,\,1} - 74880\, S_{3,\,3,\,2}\right) z - 2184\, S_{4,\,1,\,1} + 30576\, S_{4,\,1,\,2} + 1716\, T_{3,\,-3,\,1}$$

$$- 11154\, T_{3,\,-3,\,2}\Big) y^4 + \Big(\left(198900\, S_{4,\,1,\,2} + 38025\, T_{3,\,-3,\,2}\right) z^4 + \Big(45968\, T_{4,\,-1,\,1}$$

$$- 74880\, S_{3,\,3,\,2}\Big) z^3 + \left(2184\, S_{4,\,1,\,1} - 30576\, S_{4,\,1,\,2} + 5148\, T_{3,\,-3,\,1} - 33462\, T_{3,\,-3,\,2}\right) z^2$$

$$+ \left(-24024\, T_{4,\,-1,\,1} + 4368\, T_{4,\,-1,\,0} - 3744\, S_{3,\,3,\,1} + 44928\, S_{3,\,3,\,2}\right) z + 1560\, S_{4,\,1,\,1}$$

$$- 8580\, S_{4,\,1,\,2} - 1404\, T_{3,\,-3,\,1} + 3861\, T_{3,\,-3,\,2} + 312\, T_{3,\,-3,\,0}\Big) y^2 + 14560\, \Big(\frac{153}{10} z^4 S_{4,\,1,\,2}$$

$$-\frac{39}{35} T_{4,-1,1} z^3 + (S_{4,1,1} - 14 S_{4,1,2}) z^2 + (\frac{22}{35} T_{4,-1,1} - \frac{4}{35} T_{4,-1,0}) z - \frac{3}{7} S_{4,1,1}$$

$$+\frac{33}{14} S_{4,1,2}) z^2) \sqrt{2} + (((49504 S_{4,4,2} - 9360 T_{5,-4,1}) z + 3432 S_{5,4,1}$$

$$-1352 T_{4,-4,1}) x^5 + 8736 y ((\frac{195}{56} T_{5,4,1} + \frac{323}{21} S_{4,-4,2}) z + S_{5,-4,1}$$

$$+\frac{13}{28} T_{4,4,1}) x^4 + (((-42432 S_{4,4,2} + 28080 T_{5,-4,1}) z - 624 S_{5,4,1}$$

$$+2704 T_{4,-4,1}) y^2 + (63648 S_{4,4,2} - 18720 T_{5,-4,1}) z^3 + (14976 S_{5,4,1}$$

$$-4056 T_{4,-4,1}) z^2 + (2912 S_{4,4,1} - 40768 S_{4,4,2} + 8112 T_{5,-4,1} - 1248 T_{5,-4,0}) z$$

$$-3744 S_{5,4,1} + 1144 T_{4,-4,1} - 208 T_{4,-4,0}) x^3 + 16224 y (((\frac{15}{52} T_{5,4,1}$$

$$+\frac{34}{3} S_{4,-4,2}) z + S_{5,-4,1} + \frac{1}{6} T_{4,4,1}) y^2 + (\frac{45}{13} T_{5,4,1} + \frac{153}{13} S_{4,-4,2}) z^3$$

$$+(\frac{36}{13} S_{5,-4,1} + \frac{3}{4} T_{4,4,1}) z^2 + (-\frac{3}{2} T_{5,4,1} + \frac{3}{13} T_{5,4,0} + \frac{7}{13} S_{4,-4,1}$$

$$-\frac{98}{13} S_{4,-4,2}) z - \frac{9}{13} S_{5,-4,1} - \frac{11}{52} T_{4,4,1} + \frac{1}{26} T_{4,4,0}) x^2$$

$$-8736 y^2 (((\frac{493}{21} S_{4,4,2} - \frac{15}{7} T_{5,-4,1}) z + \frac{53}{28} S_{5,4,1} - \frac{13}{28} T_{4,-4,1}) y^2$$

$$+(\frac{153}{7} S_{4,4,2} - \frac{45}{7} T_{5,-4,1}) z^3 + (\frac{36}{7} S_{5,4,1} - \frac{39}{28} T_{4,-4,1}) z^2 + (S_{4,4,1} - 14 S_{4,4,2}$$

$$+\frac{39}{14} T_{5,-4,1} - \frac{3}{7} T_{5,-4,0}) z - \frac{9}{7} S_{5,4,1} + \frac{11}{28} T_{4,-4,1} - \frac{1}{14} T_{4,-4,0}) x$$

$$-4992 y^3 (((\frac{45}{32} T_{5,4,1} + \frac{51}{4} S_{4,-4,2}) z + S_{5,-4,1} + \frac{13}{48} T_{4,4,1}) y^2 + (\frac{15}{4} T_{5,4,1}$$

$$+\frac{51}{4} S_{4,-4,2}) z^3 + (3 S_{5,-4,1} + \frac{13}{16} T_{4,4,1}) z^2 + (-\frac{13}{8} T_{5,4,1} + \frac{1}{4} T_{5,4,0}$$

$$+\frac{7}{12} S_{4,-4,1} - \frac{49}{6} S_{4,-4,2}) z - \frac{3}{4} S_{5,-4,1} - \frac{11}{48} T_{4,4,1} + \frac{1}{24} T_{4,4,0})) \sqrt{7} + ($$

$$-77792 z S_{4,2,2} + 1352 T_{4,-2,1}) x^5 - 282880 (S_{4,-2,2} z - \frac{39}{1360} T_{4,2,1}) y x^4$$

$$+((254592 z S_{4,2,2} + 21632 T_{4,-2,1}) y^2 + 297024 S_{4,2,2} z^3 - 20280 T_{4,-2,1} z^2$$

$$+(1664 S_{4,2,1} - 23296 S_{4,2,2}) z - 1144 T_{4,-2,1} + 208 T_{4,-2,0}) x^3$$

$$-11232 y ((\frac{374}{27} S_{4,-2,2} z + \frac{13}{54} T_{4,2,1}) y^2 + \frac{85}{9} S_{4,-2,2} z^3 - \frac{13}{3} T_{4,2,1} z^2 + (S_{4,-2,1}$$

$$-14 S_{4,-2,2}) z + \frac{11}{18} T_{4,2,1} - \frac{1}{9} T_{4,2,0}) x^2 + ((332384 z S_{4,2,2} + 20280 T_{4,-2,1}) y^4$$

$$+(1103232 S_{4,2,2} z^3 + 36504 T_{4,-2,1} z^2 + (27456 S_{4,2,1} - 384384 S_{4,2,2}) z$$

$$- 17160\, T_{4,\,-2,\,1} + 3120\, T_{4,\,-2,\,0}\Big)\, y^{2} + 34944\, \Big(\frac{459}{28}\, S_{4,\,2,\,2}\, z^{4} - \frac{65}{56}\, T_{4,\,-2,\,1}\, z^{3} + \big(S_{4,\,2,\,1}$$

$$- 14\, S_{4,\,2,\,2}\big)\, z^{2} + \Big(\frac{33}{56}\, T_{4,\,-2,\,1} - \frac{3}{28}\, T_{4,\,-2,\,0}\Big)\, z - \frac{5}{14}\, S_{4,\,2,\,1} + \frac{55}{28}\, S_{4,\,2,\,2}\Big)\, z\Big)\, x$$

$$+ 14560\, y\, \Big(\Big(\frac{306}{35}\, S_{4,\,-2,\,2}\, z - \frac{26}{35}\, T_{4,\,2,\,1}\Big)\, y^{4} + \Big(\frac{1683}{35}\, S_{4,\,-2,\,2}\, z^{3} - \frac{39}{70}\, T_{4,\,2,\,1}\, z^{2}$$

$$+ \big(S_{4,\,-2,\,1} - 14\, S_{4,\,-2,\,2}\big)\, z + \frac{22}{35}\, T_{4,\,2,\,1} - \frac{4}{35}\, T_{4,\,2,\,0}\Big)\, y^{2} + \frac{12}{5}\, \Big(\frac{459}{28}\, S_{4,\,-2,\,2}\, z^{4}$$

$$+ \frac{65}{56}\, T_{4,\,2,\,1}\, z^{3} + \big(S_{4,\,-2,\,1} - 14\, S_{4,\,-2,\,2}\big)\, z^{2} + \Big(-\frac{33}{56}\, T_{4,\,2,\,1} + \frac{3}{28}\, T_{4,\,2,\,0}\Big)\, z$$

$$- \frac{5}{14}\, S_{4,\,-2,\,1} + \frac{55}{28}\, S_{4,\,-2,\,2}\Big)\, z\Big)\Big)\, \sqrt{5} + \frac{1}{416}\, \Big(\Big(2535\, x^{6}\, T_{3,\,-1,\,2} + 25350\, x^{5}\, y\, T_{3,\,1,\,2}$$

$$+ \big(32955\, y^{2}\, T_{3,\,-1,\,2} - 15210\, z^{2}\, T_{3,\,-1,\,2} - 81120\, z\, S_{3,\,1,\,2} + 572\, T_{3,\,-1,\,1} - 3718\, T_{3,\,-1,\,2}\big)\, x^{4}$$

$$+ 5720\, y\, \Big(\frac{195}{22}\, T_{3,\,1,\,2}\, y^{2} + \frac{585}{22}\, T_{3,\,1,\,2}\, z^{2} - \frac{204}{11}\, z\, S_{3,\,-1,\,2} + T_{3,\,1,\,1} - \frac{13}{2}\, T_{3,\,1,\,2}\Big)\, x^{3}$$

$$+ \big(58305\, y^{4}\, T_{3,\,-1,\,2} + \big(121680\, z^{2}\, T_{3,\,-1,\,2} - 56160\, z\, S_{3,\,1,\,2} + 6864\, T_{3,\,-1,\,1}$$

$$- 44616\, T_{3,\,-1,\,2}\big)\, y^{2} - 88725\, z^{4}\, T_{3,\,-1,\,2} - 24960\, z^{3}\, S_{3,\,1,\,2} + \big(-5148\, T_{3,\,-1,\,1}$$

$$+ 33462\, T_{3,\,-1,\,2}\big)\, z^{2} + \big(-3744\, S_{3,\,1,\,1} + 44928\, S_{3,\,1,\,2}\big)\, z - 468\, T_{3,\,-1,\,1} + 1287\, T_{3,\,-1,\,2}$$

$$+ 104\, T_{3,\,-1,\,0}\big)\, x^{2} + 5720\, y\, \Big(\frac{195}{44}\, T_{3,\,1,\,2}\, y^{4} + \Big(\frac{585}{22}\, T_{3,\,1,\,2}\, z^{2} - \frac{204}{11}\, z\, S_{3,\,-1,\,2} + T_{3,\,1,\,1}$$

$$- \frac{13}{2}\, T_{3,\,1,\,2}\Big)\, y^{2} + \frac{975}{44}\, T_{3,\,1,\,2}\, z^{4} - \frac{384}{11}\, z^{3}\, S_{3,\,-1,\,2} + \Big(3\, T_{3,\,1,\,1} - \frac{39}{2}\, T_{3,\,1,\,2}\Big)\, z^{2} + \Big($$

$$-\frac{72}{55}\, S_{3,\,-1,\,1} + \frac{864}{55}\, S_{3,\,-1,\,2}\Big)\, z - \frac{9}{11}\, T_{3,\,1,\,1} + \frac{9}{4}\, T_{3,\,1,\,2} + \frac{2}{11}\, T_{3,\,1,\,0}\Big)\, x$$

$$+ 27885\, y^{6}\, T_{3,\,-1,\,2} + \big(136890\, z^{2}\, T_{3,\,-1,\,2} + 24960\, z\, S_{3,\,1,\,2} + 6292\, T_{3,\,-1,\,1}$$

$$- 40898\, T_{3,\,-1,\,2}\big)\, y^{4} + \big(38025\, z^{4}\, T_{3,\,-1,\,2} + 174720\, z^{3}\, S_{3,\,1,\,2} + \big(12012\, T_{3,\,-1,\,1}$$

$$- 78078\, T_{3,\,-1,\,2}\big)\, z^{2} + \big(3744\, S_{3,\,1,\,1} - 44928\, S_{3,\,1,\,2}\big)\, z - 5148\, T_{3,\,-1,\,1} + 14157\, T_{3,\,-1,\,2}$$

$$+ 1144\, T_{3,\,-1,\,0}\big)\, y^{2} - 11440\, \Big(\frac{273}{44}\, T_{3,\,-1,\,2}\, z^{5} - \frac{144}{11}\, z^{4}\, S_{3,\,1,\,2} + \Big(T_{3,\,-1,\,1}$$

$$- \frac{13}{2}\, T_{3,\,-1,\,2}\Big)\, z^{3} + \Big(-\frac{48}{55}\, S_{3,\,1,\,1} + \frac{576}{55}\, S_{3,\,1,\,2}\Big)\, z^{2} + \Big(-\frac{27}{55}\, T_{3,\,-1,\,1} + \frac{27}{20}\, T_{3,\,-1,\,2}$$

$$+ \frac{6}{55}\, T_{3,\,-1,\,0}\Big)\, z + \frac{16}{55}\, S_{3,\,1,\,1} - \frac{72}{55}\, S_{3,\,1,\,2}\Big)\, z\Big)\, \sqrt{2} + \Big(8580\, x^{6}\, S_{6,\,1,\,1}$$

$$+ 6240\, x^{5}\, y\, S_{6,\,-1,\,1} + \big(19500\, y^{2}\, S_{6,\,1,\,1} - 11700\, z^{2}\, S_{6,\,1,\,1} - 520\, z\, T_{6,\,-1,\,0} - 9100\, S_{6,\,1,\,1}\big)\, x^{4}$$

$$+ 12480\, y\, \Big(S_{6,\,-1,\,1}\, y^{2} + \frac{3}{4}\, S_{6,\,-1,\,1}\, z^{2} - \frac{2}{3}\, z\, T_{6,\,1,\,0} - \frac{7}{12}\, S_{6,\,-1,\,1}\Big)\, x^{3} + \big(13260\, y^{4}\, S_{6,\,1,\,1}$$

$$+\left(-32760\,z^{2}\,S_{6,\,1,\,1}-9360\,z\,T_{6,\,-1,\,0}-10920\,S_{6,\,1,\,1}\right)y^{2}-146640\,S_{6,\,1,\,1}\,z^{4}$$

$$+4160\,z^{3}\,T_{6,\,-1,\,0}+65520\,S_{6,\,1,\,1}\,z^{2}\Big)\,x^{2}+6240\,\Bigg(S_{6,\,-1,\,1}\,y^{4}+\left(\frac{3}{2}\,S_{6,\,-1,\,1}\,z^{2}-\frac{4}{3}\,z\,T_{6,\,1,\,0}\right.$$

$$\left.-\frac{7}{6}\,S_{6,\,-1,\,1}\right)y^{2}-19\,S_{6,\,-1,\,1}\,z^{4}+\frac{8}{3}\,z^{3}\,T_{6,\,1,\,0}+7\,S_{6,\,-1,\,1}\,z^{2}\Bigg)\,y\,x+2340\,y^{6}\,S_{6,\,1,\,1}+\Big($$

$$-21060\,z^{2}\,S_{6,\,1,\,1}-8840\,z\,T_{6,\,-1,\,0}-1820\,S_{6,\,1,\,1}\Big)\,y^{4}-28080\,\left(S_{6,\,1,\,1}\,z^{2}-\frac{20}{27}\,z\,T_{6,\,-1,\,0}\right.$$

$$\left.-\frac{7}{9}\,S_{6,\,1,\,1}\right)z^{2}\,y^{2}+26208\,\left(S_{6,\,1,\,1}\,z^{2}-\frac{2}{21}\,z\,T_{6,\,-1,\,0}-\frac{5}{9}\,S_{6,\,1,\,1}\right)z^{4}\Bigg)\sqrt{7}$$

$$-39780\,x^{6}\,S_{2,\,1,\,3}+\left(-159120\,y\,S_{2,\,-1,\,3}+397800\,z\,S_{2,\,2,\,3}-7436\,T_{2,\,-2,\,2}\right)x^{5}$$

$$+\Big(39780\,y^{2}\,S_{2,\,1,\,3}+\left(79560\,z\,S_{2,\,-2,\,3}+7436\,T_{2,\,2,\,2}\right)y+119340\,z^{2}\,S_{2,\,1,\,3}$$

$$-14872\,T_{2,\,-1,\,2}\,z-2704\,S_{2,\,1,\,2}+27300\,S_{2,\,1,\,3}\Big)\,x^{4}+\Big(-318240\,y^{3}\,S_{2,\,-1,\,3}$$

$$+\left(1432080\,z\,S_{2,\,2,\,3}-14872\,T_{2,\,-2,\,2}\right)y^{2}+\Big(-954720\,z^{2}\,S_{2,\,-1,\,3}+29744\,z\,T_{2,\,1,\,2}$$

$$-21632\,S_{2,\,-1,\,2}+218400\,S_{2,\,-1,\,3}\Big)\,y+1113840\,z^{3}\,S_{2,\,2,\,3}-44616\,z^{2}\,T_{2,\,-2,\,2}$$

$$+\left(54080\,S_{2,\,2,\,2}-546000\,S_{2,\,2,\,3}\right)z-1872\,T_{2,\,-2,\,1}+10296\,T_{2,\,-2,\,2}\Big)\,x^{3}$$

$$+\Big(198900\,y^{4}\,S_{2,\,1,\,3}+\left(795600\,z\,S_{2,\,-2,\,3}+14872\,T_{2,\,2,\,2}\right)y^{3}+\Big(1193400\,z^{2}\,S_{2,\,1,\,3}$$

$$+16224\,S_{2,\,1,\,2}-163800\,S_{2,\,1,\,3}\Big)\,y^{2}+\Big(795600\,z^{3}\,S_{2,\,-2,\,3}+44616\,T_{2,\,2,\,2}\,z^{2}$$

$$+\left(32448\,S_{2,\,-2,\,2}-327600\,S_{2,\,-2,\,3}\right)z+1872\,T_{2,\,2,\,1}-10296\,T_{2,\,2,\,2}\Big)\,y+994500\,z^{4}\,S_{2,\,1,\,3}$$

$$-59488\,z^{3}\,T_{2,\,-1,\,2}+\left(48672\,S_{2,\,1,\,2}-491400\,S_{2,\,1,\,3}\right)z^{2}+\Big(-3744\,T_{2,\,-1,\,1}$$

$$+20592\,T_{2,\,-1,\,2}\Big)\,z+416\,S_{2,\,1,\,1}-4160\,S_{2,\,1,\,2}+17316\,S_{2,\,1,\,3}\Big)\,x^{2}+\Big(-159120\,y^{5}\,S_{2,\,-1,\,3}$$

$$+\left(1034280\,z\,S_{2,\,2,\,3}-7436\,T_{2,\,-2,\,2}\right)y^{4}+\Big(-954720\,z^{2}\,S_{2,\,-1,\,3}+29744\,z\,T_{2,\,1,\,2}$$

$$-21632\,S_{2,\,-1,\,2}+218400\,S_{2,\,-1,\,3}\Big)\,y^{3}+\Big(1750320\,z^{3}\,S_{2,\,2,\,3}-44616\,z^{2}\,T_{2,\,-2,\,2}$$

$$+\left(97344\,S_{2,\,2,\,2}-982800\,S_{2,\,2,\,3}\right)z-1872\,T_{2,\,-2,\,1}+10296\,T_{2,\,-2,\,2}\Big)\,y^{2}+\Big($$

$$-795600\,z^{4}\,S_{2,\,-1,\,3}+29744\,T_{2,\,1,\,2}\,z^{3}+\left(-64896\,S_{2,\,-1,\,2}+655200\,S_{2,\,-1,\,3}\right)z^{2}$$

$$+\left(3744\,T_{2,\,1,\,1}-20592\,T_{2,\,1,\,2}\right)z-1664\,S_{2,\,-1,\,1}+16640\,S_{2,\,-1,\,2}-69264\,S_{2,\,-1,\,3}\Big)\,y$$

$$+716040\,z^{5}\,S_{2,\,2,\,3}-37180\,z^{4}\,T_{2,\,-2,\,2}+\left(75712\,S_{2,\,2,\,2}-764400\,S_{2,\,2,\,3}\right)z^{3}+\Big($$

$$-5616\,T_{2,\,-2,\,1} + 30888\,T_{2,\,-2,\,2}\Big)\,z^2 + \Big(4160\,S_{2,\,2,\,1} - 41600\,S_{2,\,2,\,2} + 173160\,S_{2,\,2,\,3}\Big)\,z$$

$$+ 1456\,T_{2,\,-2,\,1} - 3276\,T_{2,\,-2,\,2} - 416\,T_{2,\,-2,\,0}\Big)\,x + 119340\,y^6\,S_{2,\,1,\,3} + \Big(716040\,z\,S_{2,\,-2,\,3}$$

$$+ 7436\,T_{2,\,2,\,2}\Big)\,y^5 + \Big(1074060\,z^2\,S_{2,\,1,\,3} + 14872\,z\,T_{2,\,-1,\,2} + 18928\,S_{2,\,1,\,2}$$

$$- 191100\,S_{2,\,1,\,3}\Big)\,y^4 + \Big(1432080\,z^3\,S_{2,\,-2,\,3} + 44616\,T_{2,\,2,\,2}\,z^2 + \Big(75712\,S_{2,\,-2,\,2}$$

$$- 764400\,S_{2,\,-2,\,3}\Big)\,z + 1872\,T_{2,\,2,\,1} - 10296\,T_{2,\,2,\,2}\Big)\,y^3 + \Big(1790100\,z^4\,S_{2,\,1,\,3}$$

$$- 29744\,z^3\,T_{2,\,-1,\,2} + \Big(113568\,S_{2,\,1,\,2} - 1146600\,S_{2,\,1,\,3}\Big)\,z^2 + 2080\,S_{2,\,1,\,1} - 20800\,S_{2,\,1,\,2}$$

$$+ 86580\,S_{2,\,1,\,3}\Big)\,y^2 + \Big(716040\,z^5\,S_{2,\,-2,\,3} + 37180\,T_{2,\,2,\,2}\,z^4 + \Big(75712\,S_{2,\,-2,\,2}$$

$$- 764400\,S_{2,\,-2,\,3}\Big)\,z^3 + \Big(5616\,T_{2,\,2,\,1} - 30888\,T_{2,\,2,\,2}\Big)\,z^2 + \Big(4160\,S_{2,\,-2,\,1} - 41600\,S_{2,\,-2,\,2}$$

$$+ 173160\,S_{2,\,-2,\,3}\Big)\,z - 1456\,T_{2,\,2,\,1} + 3276\,T_{2,\,2,\,2} + 416\,T_{2,\,2,\,0}\Big)\,y + 835380\,z^6\,S_{2,\,1,\,3}$$

$$- 44616\,z^5\,T_{2,\,-1,\,2} + \Big(94640\,S_{2,\,1,\,2} - 955500\,S_{2,\,1,\,3}\Big)\,z^4 + \Big(-7488\,T_{2,\,-1,\,1}$$

$$+ 41184\,T_{2,\,-1,\,2}\Big)\,z^3 + \Big(6240\,S_{2,\,1,\,1} - 62400\,S_{2,\,1,\,2} + 259740\,S_{2,\,1,\,3}\Big)\,z^2 + \Big(2912\,T_{2,\,-1,\,1}$$

$$- 6552\,T_{2,\,-1,\,2} - 832\,T_{2,\,-1,\,0}\Big)\,z - 1248\,S_{2,\,1,\,1} + 4368\,S_{2,\,1,\,2} - 9828\,S_{2,\,1,\,3}\Big)\,\sqrt{3}$$

$$+ \frac{1}{416}\,\Bigg(\Big(\Big(-716040\,S_{2,\,0,\,3} + 106080\,S_{4,\,0,\,2} + 98280\,S_{6,\,0,\,1}\Big)\,z - 60840\,S_{1,\,0,\,3}$$

$$- 18720\,S_{3,\,0,\,2} + 17160\,S_{5,\,0,\,1}\Big)\,y^4 - 243360\,y^3\,z\,S_{1,\,-1,\,3} + \Big(\Big(-2386800\,S_{2,\,0,\,3}$$

$$- 884000\,S_{4,\,0,\,2} + 262080\,S_{6,\,0,\,1}\Big)\,z^3 + \Big(-365040\,S_{1,\,0,\,3} - 393120\,S_{3,\,0,\,2}$$

$$+ 37440\,S_{5,\,0,\,1}\Big)\,z^2 + \Big(-97344\,S_{2,\,0,\,2} + 982800\,S_{2,\,0,\,3} - 6240\,S_{4,\,0,\,1} + 87360\,S_{4,\,0,\,2}$$

$$- 131040\,S_{6,\,0,\,1}\Big)\,z - 9152\,S_{1,\,0,\,2} + 78416\,S_{1,\,0,\,3} - 3744\,S_{3,\,0,\,1} + 44928\,S_{3,\,0,\,2}$$

$$- 18720\,S_{5,\,0,\,1}\Big)\,y^2 - 243360\,\left(z^2\,S_{1,\,-1,\,3} + \frac{44}{585}\,S_{1,\,-1,\,2} - \frac{29}{45}\,S_{1,\,-1,\,3}\right)\,z\,y + \Big($$

$$-1670760\,S_{2,\,0,\,3} - 1485120\,S_{4,\,0,\,2} - 366912\,S_{6,\,0,\,1}\Big)\,z^5 + \Big(-304200\,S_{1,\,0,\,3}$$

$$- 561600\,S_{3,\,0,\,2} - 187200\,S_{5,\,0,\,1}\Big)\,z^4 + \Big(-162240\,S_{2,\,0,\,2} + 1638000\,S_{2,\,0,\,3} - 83200\,S_{4,\,0,\,1}$$

$$+ 1164800\,S_{4,\,0,\,2} + 174720\,S_{6,\,0,\,1}\Big)\,z^3 + \Big(-27456\,S_{1,\,0,\,2} + 235248\,S_{1,\,0,\,3} - 29952\,S_{3,\,0,\,1}$$

$$+ 359424\,S_{3,\,0,\,2} + 74880\,S_{5,\,0,\,1}\Big)\,z^2 + \Big(-7488\,S_{2,\,0,\,1} + 74880\,S_{2,\,0,\,2} - 311688\,S_{2,\,0,\,3}$$

$$+ 24960 S_{4,0,1} - 137280 S_{4,0,2}\Big) z - 832 S_{1,0,1} + 6656 S_{1,0,2} - 22568 S_{1,0,3}$$

$$+ 4992 S_{3,0,1} - 22464 S_{3,0,2}\Big) x + \frac{1}{416} \Big(-55770 y^4 T_{1,-1,3} + \Big(\Big(223080 T_{1,0,3}$$

$$+ 121680 T_{3,0,2} - 257400 T_{5,0,1}\Big) z + 44616 T_{2,0,2} - 40560 T_{4,0,1} + 10920 T_{6,0,0}\Big) y^3$$

$$+ \Big(-334620 z^2 T_{1,-1,3} + 730080 z S_{1,1,3} - 10296 T_{1,-1,2} + 66924 T_{1,-1,3}\Big) y^2$$

$$+ \Big(\Big(223080 T_{1,0,3} + 425880 T_{3,0,2} - 93600 T_{5,0,1}\Big) z^3 + \Big(133848 T_{2,0,2} + 20280 T_{4,0,1}$$

$$- 65520 T_{6,0,0}\Big) z^2 + \Big(20592 T_{1,0,2} - 133848 T_{1,0,3} + 20592 T_{3,0,1} - 133848 T_{3,0,2}$$

$$+ 121680 T_{5,0,1} - 18720 T_{5,0,0} + 189000\Big) z + 5616 T_{2,0,1} - 30888 T_{2,0,2} + 17160 T_{4,0,1}$$

$$- 3120 T_{4,0,0}\Big) y - 278850 z^4 T_{1,-1,3} + 730080 z^3 S_{1,1,3} + \Big(-30888 T_{1,-1,2}$$

$$+ 200772 T_{1,-1,3}\Big) z^2 + \Big(36608 S_{1,1,2} - 313664 S_{1,1,3}\Big) z - 1456 T_{1,-1,1} + 6552 T_{1,-1,2}$$

$$- 18018 T_{1,-1,3}\Big) x^2 + \frac{1}{416} \Big(\Big(\Big(-1432080 S_{2,0,3} + 212160 S_{4,0,2} + 196560 S_{6,0,1}\Big) z$$

$$- 121680 S_{1,0,3} - 37440 S_{3,0,2} + 34320 S_{5,0,1}\Big) y^2 - 243360 y z S_{1,-1,3} + \Big($$

$$-2386800 S_{2,0,3} - 884000 S_{4,0,2} + 262080 S_{6,0,1}\Big) z^3 + \Big(-365040 S_{1,0,3} - 393120 S_{3,0,2}$$

$$+ 37440 S_{5,0,1}\Big) z^2 + \Big(-97344 S_{2,0,2} + 982800 S_{2,0,3} - 6240 S_{4,0,1} + 87360 S_{4,0,2}$$

$$- 131040 S_{6,0,1}\Big) z - 9152 S_{1,0,2} + 78416 S_{1,0,3} - 3744 S_{3,0,1} + 44928 S_{3,0,2}$$

$$- 18720 S_{5,0,1}\Big) x^3 + \frac{1}{416} \Big(-55770 y^2 T_{1,-1,3} + \Big(\Big(111540 T_{1,0,3} + 60840 T_{3,0,2}$$

$$- 128700 T_{5,0,1}\Big) z + 22308 T_{2,0,2} - 20280 T_{4,0,1} + 5460 T_{6,0,0}\Big) y - 167310 z^2 T_{1,-1,3}$$

$$+ 243360 z S_{1,1,3} - 5148 T_{1,-1,2} + 33462 T_{1,-1,3}\Big) x^4 + \frac{1}{416} \Big(\Big(-716040 S_{2,0,3}$$

$$+ 106080 S_{4,0,2} + 98280 S_{6,0,1}\Big) z - 60840 S_{1,0,3} - 18720 S_{3,0,2} + 17160 S_{5,0,1}\Big) x^5$$

$$+ \frac{1}{416} \left(\Big(29016 z S_{6,4,1} + 312 T_{6,-4,0}\Big) x^5 + 65520 y \left(S_{6,-4,1} z + \frac{1}{84} T_{6,4,0}\right) x^4\right.$$

$$+ \Big(\Big(28080 z S_{6,4,1} + 6240 T_{6,-4,0}\Big) y^2 + 112320 S_{6,4,1} z^3 - 9360 z^2 T_{6,-4,0}$$

$$- 43680 S_{6,4,1} z\Big) x^3 + 187200 \left(\left(S_{6,-4,1} z - \frac{7}{120} T_{6,4,0}\right) y^2 + \frac{9}{5} \left(S_{6,-4,1} z^2\right.\right.$$

$$\left.\left.+ \frac{1}{12} z T_{6,4,0} - \frac{7}{18} S_{6,-4,1}\right) z\right) y x^2 - 173160 y^2 \left(\left(S_{6,4,1} z + \frac{5}{111} T_{6,-4,0}\right) y^2\right.$$

$$+\frac{72}{37}\left(S_{6,4,1}z^2-\frac{1}{12}zT_{6,-4,0}-\frac{7}{18}S_{6,4,1}\right)z\Big)x-50544y^3\Big(\Big(S_{6,-4,1}z$$
$$-\frac{13}{324}T_{6,4,0}\Big)y^2+\frac{20}{9}\left(S_{6,-4,1}z^2+\frac{1}{12}zT_{6,4,0}-\frac{7}{18}S_{6,-4,1}\right)z\Big)\Big)\sqrt{7}$$
$$+\frac{1}{416}\left(3328S_{1,1,1}-26624S_{1,1,2}+90272S_{1,1,3}\right)z+\frac{1}{416}\Big(\Big(111540T_{1,0,3}$$
$$+365040T_{3,0,2}+280800T_{5,0,1}\Big)z^5+\Big(111540T_{2,0,2}+135200T_{4,0,1}$$
$$+43680T_{6,0,0}\Big)z^4+\Big(20592T_{1,0,2}-133848T_{1,0,3}+54912T_{3,0,1}-356928T_{3,0,2}$$
$$-162240T_{5,0,1}+24960T_{5,0,0}+157500\Big)z^3+\Big(16848T_{2,0,1}-92664T_{2,0,2}$$
$$-68640T_{4,0,1}+12480T_{4,0,0}\Big)z^2+\Big(2912T_{1,0,1}-13104T_{1,0,2}+36036T_{1,0,3}$$
$$-22464T_{3,0,1}+61776T_{3,0,2}+4992T_{3,0,0}-132300\Big)z-4368T_{2,0,1}+9828T_{2,0,2}$$
$$+1248T_{2,0,0}\Big)y+\frac{1}{416}\left(-25740T_{1,-1,2}+167310T_{1,-1,3}\right)z^4+\frac{1}{416}\Big(54912S_{1,1,2}$$
$$-470496S_{1,1,3}\Big)z^3+\frac{1}{416}\left(-4368T_{1,-1,1}+19656T_{1,-1,2}-54054T_{1,-1,3}\right)z^2$$
$$+\frac{1}{416}\Big(-278850z^4T_{1,-1,3}+973440z^3S_{1,1,3}+\left(-30888T_{1,-1,2}+200772T_{1,-1,3}\right)z^2$$
$$+\left(54912S_{1,1,2}-470496S_{1,1,3}\right)z-1456T_{1,-1,1}+6552T_{1,-1,2}-18018T_{1,-1,3}\Big)y^2$$
$$+\frac{1}{416}\Big(\Big(223080T_{1,0,3}+425880T_{3,0,2}-93600T_{5,0,1}\Big)z^3+\Big(133848T_{2,0,2}$$
$$+20280T_{4,0,1}-65520T_{6,0,0}\Big)z^2+\Big(20592T_{1,0,2}-133848T_{1,0,3}+20592T_{3,0,1}$$
$$-133848T_{3,0,2}+121680T_{5,0,1}-18720T_{5,0,0}+126000\Big)z+5616T_{2,0,1}$$
$$-30888T_{2,0,2}+17160T_{4,0,1}-3120T_{4,0,0}\Big)y^3+\frac{1}{416}\Big(\Big(111540T_{1,0,3}+60840T_{3,0,2}$$
$$-128700T_{5,0,1}\Big)z+22308T_{2,0,2}-20280T_{4,0,1}+5460T_{6,0,0}\Big)y^5+\frac{1}{416}\Big($$
$$-167310z^2T_{1,-1,3}+486720zS_{1,1,3}-5148T_{1,-1,2}+33462T_{1,-1,3}\Big)y^4$$
$$-\frac{715}{16}x^6T_{1,-1,3}-\frac{5005}{16}z^6T_{1,-1,3}-\frac{715}{16}y^6T_{1,-1,3}\Big)\bar{e}_x+\Big(\frac{1}{416}\Big(\Big(-2106\sqrt{7}\Big($$
$$-\frac{11}{9}S_{6,-3,1}y^6+\left(-\frac{8}{3}xS_{6,3,1}-\frac{110}{27}S_{6,2,1}z-\frac{1}{81}T_{6,-2,0}\right)y^5+\Big(-\frac{5}{9}x^2S_{6,-3,1}$$
$$+\left(\frac{17}{81}T_{6,2,0}+\frac{130}{27}S_{6,-2,1}z\right)x-\frac{5}{9}S_{6,-3,1}z^2-\frac{2}{9}zT_{6,3,0}+\frac{35}{27}S_{6,-3,1}\Big)y^4+\Big($$
$$-\frac{40}{9}x^3S_{6,3,1}+\left(-\frac{38}{81}T_{6,-2,0}-\frac{20}{3}S_{6,2,1}z\right)x^2+\Big(\frac{28}{9}S_{6,3,1}-\frac{20}{3}S_{6,3,1}z^2$$

$$- \frac{8}{27} z T_{6,-3,0}) x - \frac{320}{27} (S_{6,2,1} z^2 - \frac{1}{20} z T_{6,-2,0} - \frac{7}{15} S_{6,2,1}) z) y^3$$

$$+ (\frac{5}{3} x^4 S_{6,-3,1} + (-\frac{2}{81} T_{6,2,0} + \frac{220}{27} S_{6,-2,1} z) x^3 + (-\frac{14}{9} S_{6,-3,1} + \frac{50}{3} S_{6,-3,1} z^2$$

$$- \frac{20}{9} z T_{6,3,0}) x^2 + \frac{640}{27} (S_{6,-2,1} z^2 - \frac{1}{20} z T_{6,2,0} - \frac{7}{20} S_{6,-2,1}) z x$$

$$+ \frac{40}{3} (S_{6,-3,1} z^2 + \frac{4}{45} z T_{6,3,0} - \frac{7}{15} S_{6,-3,1}) z^2) y^2 + (-\frac{16}{9} x^5 S_{6,3,1} + ($$

$$-\frac{37}{81} T_{6,-2,0} - \frac{70}{27} S_{6,2,1} z) x^4 + (\frac{28}{27} S_{6,3,1} + \frac{140}{9} S_{6,3,1} z^2 + \frac{8}{3} z T_{6,-3,0}) x^3$$

$$+ \frac{320}{27} (S_{6,2,1} z + \frac{7}{20} T_{6,-2,0}) z^2 x^2 + \frac{80}{3} (S_{6,3,1} z^2 - \frac{4}{45} z T_{6,-3,0}$$

$$- \frac{7}{15} S_{6,3,1}) z^2 x + \frac{32}{3} (S_{6,2,1} z^2 - \frac{5}{54} z T_{6,-2,0} - \frac{14}{27} S_{6,2,1}) z^3) y + (x^5 S_{6,-3,1}$$

$$+ (\frac{10}{3} S_{6,-2,1} z - \frac{19}{81} T_{6,2,0}) x^4 + (-5 S_{6,-3,1} z^2 + \frac{26}{27} z T_{6,3,0} - \frac{7}{9} S_{6,-3,1}) x^3$$

$$- \frac{224}{81} (-\frac{6}{7} z T_{6,2,0} + S_{6,-2,1}) z x^2 - \frac{40}{3} (S_{6,-3,1} z^2 + \frac{4}{45} z T_{6,3,0}$$

$$- \frac{7}{15} S_{6,-3,1}) z^2 x - \frac{32}{3} (S_{6,-2,1} z^2 + \frac{5}{54} z T_{6,2,0} - \frac{14}{27} S_{6,-2,1}) z^3) x) \sqrt{2} + (($$

$$-3120 z T_{5,-2,1} + 2288 S_{5,2,1}) y^5 - 2912 (-\frac{75}{28} T_{5,2,1} z + S_{5,-2,1}) x y^4 + (($$

$$-28080 z T_{5,-2,1} + 3328 S_{5,2,1}) x^2 + 6240 T_{5,-2,1} z^3 + 6240 S_{5,2,1} z^2 + (2704 T_{5,-2,1}$$

$$- 416 T_{5,-2,0}) z - 2496 S_{5,2,1}) y^3 - 4576 ((\frac{15}{11} T_{5,2,1} z + S_{5,-2,1}) x^2 - \frac{75}{11} T_{5,2,1} z^3$$

$$+ \frac{42}{11} S_{5,-2,1} z^2 + (\frac{39}{22} T_{5,2,1} - \frac{3}{11} T_{5,2,0}) z - \frac{9}{11} S_{5,-2,1}) x y^2 + (($$

$$-24960 z T_{5,-2,1} + 1040 S_{5,2,1}) x^4 - 16224 (\frac{35}{13} T_{5,-2,1} z^2 + S_{5,2,1} z - \frac{3}{2} T_{5,-2,1}$$

$$+ \frac{3}{13} T_{5,-2,0}) z x^2 - 16640 (-\frac{9}{8} T_{5,-2,1} z^3 + S_{5,2,1} z^2 + (\frac{13}{20} T_{5,-2,1}$$

$$- \frac{1}{10} T_{5,-2,0}) z - \frac{9}{20} S_{5,2,1}) z^2) y - 1664 ((\frac{135}{16} T_{5,2,1} z + S_{5,-2,1}) x^4$$

$$+ (\frac{45}{4} T_{5,2,1} z^3 - 3 S_{5,-2,1} z^2 + (-\frac{65}{8} T_{5,2,1} + \frac{5}{4} T_{5,2,0}) z - \frac{3}{4} S_{5,-2,1}) x^2$$

$$- 10 (\frac{9}{8} T_{5,2,1} z^3 + S_{5,-2,1} z^2 + (-\frac{13}{20} T_{5,2,1} + \frac{1}{10} T_{5,2,0}) z - \frac{9}{20} S_{5,-2,1}) z^2) x)$$

$$\sqrt{7} + 390 y^6 T_{5,1,1} + (10920 x T_{5,-1,1} + 20280 z T_{3,-2,2} - 6240 S_{3,2,2}) y^5$$

$$+ (12090 x^2 T_{5,1,1} + (40560 z T_{3,2,2} - 12480 S_{3,-2,2}) x - 12870 z^2 T_{5,1,1}$$

$$- 13312 z S_{5,-1,1} - 338 T_{5,1,1} + 52 T_{5,1,0}) y^4 + (21840 x^3 T_{5,-1,1} - 49920 x^2 S_{3,2,2} + ($$

$$-32760 z^2 T_{5,\,-1,\,1} - 4160 z S_{5,\,1,\,1} + 1456 T_{5,\,-1,\,0} - 9464 T_{5,\,-1,\,1}\Big) x + 81120 z^3 T_{3,\,-2,\,2}$$
$$- 93600 z^2 S_{3,\,2,\,2} + \left(4576 T_{3,\,-2,\,1} - 29744 T_{3,\,-2,\,2}\right) z - 1248 S_{3,\,2,\,1} + 14976 S_{3,\,2,\,2}\Big) y^3$$
$$+ \Big(23010 x^4 T_{5,\,1,\,1} + \left(40560 z T_{3,\,2,\,2} + 12480 S_{3,\,-2,\,2}\right) x^3 + \Big(-58500 z^2 T_{5,\,1,\,1}$$
$$- 22464 z S_{5,\,-1,\,1} + 1560 T_{5,\,1,\,0} - 10140 T_{5,\,1,\,1}\Big) x^2 + 6864 \Big(\frac{325}{22} T_{3,\,2,\,2} z^2$$
$$+ \frac{60}{11} z S_{3,\,-2,\,2} + T_{3,\,2,\,1} - \frac{13}{2} T_{3,\,2,\,2}\Big) z x - 7800 T_{5,\,1,\,1} z^4 - 63232 S_{5,\,-1,\,1} z^3$$
$$+ \left(12168 T_{5,\,1,\,1} - 1872 T_{5,\,1,\,0}\right) z^2 + 22464 z S_{5,\,-1,\,1}\Big) y^2 + \Big(10920 x^5 T_{5,\,-1,\,1} + \Big($$
$$-20280 z T_{3,\,-2,\,2} - 43680 S_{3,\,2,\,2}\Big) x^4 + \Big(-32760 z^2 T_{5,\,-1,\,1} - 4160 z S_{5,\,1,\,1} + 1456 T_{5,\,-1,\,0}$$
$$- 9464 T_{5,\,-1,\,1}\Big) x^3 + \Big(40560 z^3 T_{3,\,-2,\,2} - 205920 z^2 S_{3,\,2,\,2} - 3744 S_{3,\,2,\,1}$$
$$+ 44928 S_{3,\,2,\,2}\Big) x^2 - 56576 \Big(\frac{525}{272} T_{5,\,-1,\,1} z^3 + S_{5,\,1,\,1} z^2 + \Big(-\frac{273}{272} T_{5,\,-1,\,1}$$
$$+ \frac{21}{136} T_{5,\,-1,\,0}\Big) z - \frac{9}{34} S_{5,\,1,\,1}\Big) z x + 60840 z^5 T_{3,\,-2,\,2} - 124800 z^4 S_{3,\,2,\,2}$$
$$+ \left(9152 T_{3,\,-2,\,1} - 59488 T_{3,\,-2,\,2}\right) z^3 + \left(-7488 S_{3,\,2,\,1} + 89856 S_{3,\,2,\,2}\right) z^2 + \Big($$
$$-3744 T_{3,\,-2,\,1} + 10296 T_{3,\,-2,\,2} + 832 T_{3,\,-2,\,0}\Big) z + 1664 S_{3,\,2,\,1} - 7488 S_{3,\,2,\,2}\Big) y$$
$$+ 11310 x^6 T_{5,\,1,\,1} + 24960 x^5 S_{3,\,-2,\,2} + \Big(-45630 z^2 T_{5,\,1,\,1} - 9152 z S_{5,\,-1,\,1} + 1508 T_{5,\,1,\,0}$$
$$- 9802 T_{5,\,1,\,1}\Big) x^4 + \Big(60840 T_{3,\,2,\,2} z^3 + 149760 S_{3,\,-2,\,2} z^2 + \Big(2288 T_{3,\,2,\,1}$$
$$- 14872 T_{3,\,2,\,2}\Big) z + 2496 S_{3,\,-2,\,1} - 29952 S_{3,\,-2,\,2}\Big) x^3 - 6656 \Big(\frac{1125}{64} T_{5,\,1,\,1} z^3$$
$$+ S_{5,\,-1,\,1} z^2 + \Big(-\frac{663}{64} T_{5,\,1,\,1} + \frac{51}{32} T_{5,\,1,\,0}\Big) z - \frac{9}{8} S_{5,\,-1,\,1}\Big) z x^2 + \Big(60840 T_{3,\,2,\,2} z^5$$
$$+ 124800 S_{3,\,-2,\,2} z^4 + \left(9152 T_{3,\,2,\,1} - 59488 T_{3,\,2,\,2}\right) z^3 + \Big(7488 S_{3,\,-2,\,1}$$
$$- 89856 S_{3,\,-2,\,2}\Big) z^2 + \left(-3744 T_{3,\,2,\,1} + 10296 T_{3,\,2,\,2} + 832 T_{3,\,2,\,0}\right) z - 1664 S_{3,\,-2,\,1}$$
$$+ 7488 S_{3,\,-2,\,2}\Big) x + 19968 \Big(\frac{35}{32} T_{5,\,1,\,1} z^3 + S_{5,\,-1,\,1} z^2 + \Big(-\frac{65}{96} T_{5,\,1,\,1} + \frac{5}{48} T_{5,\,1,\,0}\Big) z$$
$$- \frac{1}{2} S_{5,\,-1,\,1}\Big) z^3\Big) \sqrt{3} + \Big(\Big(\left(-9724 S_{4,\,-3,\,2} + 585 T_{5,\,3,\,1}\right) y^6 - 5304 x \Big(S_{4,\,3,\,2}$$
$$- \frac{15}{34} T_{5,\,-3,\,1}\Big) y^5 + \Big(\left(-52156 S_{4,\,-3,\,2} + 11115 T_{5,\,3,\,1}\right) x^2 + \Big(-100776 S_{4,\,-3,\,2}$$
$$- 12285 T_{5,\,3,\,1}\Big) z^2 + \left(-3744 S_{5,\,-3,\,1} - 4056 T_{4,\,3,\,1}\right) z + 18928 S_{4,\,-3,\,2} - 507 T_{5,\,3,\,1}$$

$$+78\,T_{5,3,0}-1352\,S_{4,-3,1}\Big)\,y^4-67184\,\bigg(\Big(S_{4,3,2}+\frac{45}{323}\,T_{5,-3,1}\Big)\,x^2+\Big(\frac{45}{19}\,S_{4,3,2}$$

$$-\frac{675}{1292}\,T_{5,-3,1}\Big)\,z^2+\Big(\frac{12}{323}\,S_{5,3,1}-\frac{91}{646}\,T_{4,-3,1}\Big)\,z+\frac{9}{323}\,S_{4,3,1}-\frac{126}{323}\,S_{4,3,2}$$

$$+\frac{39}{1292}\,T_{5,-3,1}-\frac{3}{646}\,T_{5,-3,0}\bigg)\,x\,y^3+\Big(\big(-18564\,S_{4,-3,2}+6435\,T_{5,3,1}\big)\,x^4+\Big(\Big($$

$$-127296\,S_{4,-3,2}+31590\,T_{5,3,1}\big)\,z^2+\big(-14976\,S_{5,-3,1}+4056\,T_{4,3,1}\big)\,z+34944\,S_{4,-3,2}$$

$$-9126\,T_{5,3,1}+1404\,T_{5,3,0}-2496\,S_{4,-3,1}\Big)\,x^2+\big(-119340\,S_{4,-3,2}-23400\,T_{5,3,1}\big)\,z^4$$

$$+\big(-19968\,S_{5,-3,1}-8112\,T_{4,3,1}\big)\,z^3+\big(91728\,S_{4,-3,2}+12168\,T_{5,3,1}-1872\,T_{5,3,0}$$

$$-6552\,S_{4,-3,1}\big)\,z^2+\big(7488\,S_{5,-3,1}+3432\,T_{4,3,1}-624\,T_{4,3,0}\big)\,z-8580\,S_{4,-3,2}$$

$$+1560\,S_{4,-3,1}\Big)\,y^2-61880\,\bigg(\Big(S_{4,3,2}+\frac{45}{238}\,T_{5,-3,1}\Big)\,x^4+\bigg(\Big(\frac{186}{35}\,S_{4,3,2}$$

$$+\frac{27}{238}\,T_{5,-3,1}\Big)\,z^2+\Big(\frac{216}{595}\,S_{5,3,1}-\frac{39}{595}\,T_{4,-3,1}\Big)\,z+\frac{10}{119}\,S_{4,3,1}-\frac{20}{17}\,S_{4,3,2}$$

$$-\frac{39}{238}\,T_{5,-3,1}+\frac{3}{119}\,T_{5,-3,0}\bigg)\,x^2+\Big(\frac{27}{7}\,S_{4,3,2}-\frac{90}{119}\,T_{5,-3,1}\Big)\,z^4+\Big(\frac{384}{595}\,S_{5,3,1}$$

$$-\frac{156}{595}\,T_{4,-3,1}\Big)\,z^3+\Big(\frac{18}{85}\,S_{4,3,1}-\frac{252}{85}\,S_{4,3,2}+\frac{234}{595}\,T_{5,-3,1}-\frac{36}{595}\,T_{5,-3,0}\Big)\,z^2+\Big($$

$$-\frac{144}{595}\,S_{5,3,1}+\frac{66}{595}\,T_{4,-3,1}-\frac{12}{595}\,T_{4,-3,0}\Big)\,z-\frac{6}{119}\,S_{4,3,1}+\frac{33}{119}\,S_{4,3,2}\bigg)\,x\,y$$

$$+23868\,\bigg(\Big(S_{4,-3,2}-\frac{35}{204}\,T_{5,3,1}\Big)\,x^4+\bigg(\Big(6\,S_{4,-3,2}+\frac{5}{68}\,T_{5,3,1}\Big)\,z^2+\Big(\frac{56}{153}\,S_{5,-3,1}$$

$$+\frac{52}{459}\,T_{4,3,1}\Big)\,z-\frac{196}{153}\,S_{4,-3,2}+\frac{91}{612}\,T_{5,3,1}-\frac{7}{306}\,T_{5,3,0}+\frac{14}{153}\,S_{4,-3,1}\bigg)\,x^2$$

$$+\Big(5\,S_{4,-3,2}+\frac{50}{51}\,T_{5,3,1}\Big)\,z^4+\Big(\frac{128}{153}\,S_{5,-3,1}+\frac{52}{153}\,T_{4,3,1}\Big)\,z^3+\Big(-\frac{196}{51}\,S_{4,-3,2}$$

$$-\frac{26}{51}\,T_{5,3,1}+\frac{4}{51}\,T_{5,3,0}+\frac{14}{51}\,S_{4,-3,1}\Big)\,z^2+\Big(-\frac{16}{51}\,S_{5,-3,1}-\frac{22}{153}\,T_{4,3,1}$$

$$+\frac{4}{153}\,T_{4,3,0}\Big)\,z+\frac{55}{153}\,S_{4,-3,2}-\frac{10}{153}\,S_{4,-3,1}\bigg)\,x^2\bigg)\,\sqrt{7}+\big(-29172\,S_{4,-1,2}$$

$$-7605\,T_{3,3,2}\big)\,y^6-5304\,x\,\Big(S_{4,1,2}-\frac{195}{68}\,T_{3,-3,2}\Big)\,y^5+\Big(\big(-82212\,S_{4,-1,2}$$

$$-7605\,T_{3,3,2}\big)\,x^2+\big(-376584\,S_{4,-1,2}-45630\,T_{3,3,2}\big)\,z^2+\big(-4056\,T_{4,1,1}$$

$$-56160\,S_{3,-3,2}\big)\,z-4056\,S_{4,-1,1}+56784\,S_{4,-1,2}-1716\,T_{3,3,1}+11154\,T_{3,3,2}\Big)\,y^4$$

$$-10608\,\bigg(\Big(S_{4,1,2}-\frac{195}{68}\,T_{3,-3,2}\Big)\,x^2+\Big(31\,S_{4,1,2}-\frac{585}{68}\,T_{3,-3,2}\Big)\,z^2+\Big(\frac{65}{34}\,T_{4,-1,1}$$

$$+\frac{150}{17}\,S_{3,3,2}\Big)\,z+\frac{3}{17}\,S_{4,1,1}-\frac{42}{17}\,S_{4,1,2}-\frac{11}{34}\,T_{3,-3,1}+\frac{143}{68}\,T_{3,-3,2}\bigg)\,x\,y^3+\Big(\Big($$

$$
\begin{aligned}
&-76908\, S_{4,\,-1,\,2} + 7605\, T_{3,\,3,\,2}\big)\, x^4 + \big(-424320\, S_{4,\,-1,\,2}\, z^2 + \big(-28392\, T_{4,\,1,\,1} \\
&- 56160\, S_{3,\,-3,\,2}\big)\, z - 6240\, S_{4,\,-1,\,1} + 87360\, S_{4,\,-1,\,2}\big)\, x^2 + \big(-322660\, S_{4,\,-1,\,2} \\
&- 38025\, T_{3,\,3,\,2}\big)\, z^4 + \big(2704\, T_{4,\,1,\,1} - 74880\, S_{3,\,-3,\,2}\big)\, z^3 + \big(-20904\, S_{4,\,-1,\,1} \\
&+ 292656\, S_{4,\,-1,\,2} - 5148\, T_{3,\,3,\,1} + 33462\, T_{3,\,3,\,2}\big)\, z^2 + \big(3432\, T_{4,\,1,\,1} - 624\, T_{4,\,1,\,0} \\
&- 3744\, S_{3,\,-3,\,1} + 44928\, S_{3,\,-3,\,2}\big)\, z + 4680\, S_{4,\,-1,\,1} - 25740\, S_{4,\,-1,\,2} + 1404\, T_{3,\,3,\,1} \\
&- 3861\, T_{3,\,3,\,2} - 312\, T_{3,\,3,\,0}\big)\, y^2 - 5304\, \Bigg(\bigg(\Big(S_{4,\,1,\,2} - \frac{195}{68}\, T_{3,\,-3,\,2}\Big)\, x^4 + \bigg(\Big(-\frac{585}{34}\, T_{3,\,-3,\,2} \\
&+ 62\, S_{4,\,1,\,2}\Big)\, z^2 + \Big(\frac{65}{17}\, T_{4,\,-1,\,1} + \frac{540}{17}\, S_{3,\,3,\,2}\Big)\, z - \frac{84}{17}\, S_{4,\,1,\,2} + \frac{6}{17}\, S_{4,\,1,\,1} \\
&+ \frac{143}{34}\, T_{3,\,-3,\,2} - \frac{11}{17}\, T_{3,\,-3,\,1}\bigg)\, x^2 + \Big(-\frac{975}{68}\, T_{3,\,-3,\,2} + \frac{295}{3}\, S_{4,\,1,\,2}\Big)\, z^4 + \Big(\frac{480}{17}\, S_{3,\,3,\,2} \\
&+ \frac{156}{17}\, T_{4,\,-1,\,1}\Big)\, z^3 + \Big(-\frac{33}{17}\, T_{3,\,-3,\,1} + \frac{429}{34}\, T_{3,\,-3,\,2} - \frac{1036}{17}\, S_{4,\,1,\,2} + \frac{74}{17}\, S_{4,\,1,\,1}\Big)\, z^2 \\
&+ \Big(\frac{12}{17}\, T_{4,\,-1,\,0} + \frac{24}{17}\, S_{3,\,3,\,1} - \frac{66}{17}\, T_{4,\,-1,\,1} - \frac{288}{17}\, S_{3,\,3,\,2}\Big)\, z - \frac{2}{17}\, T_{3,\,-3,\,0} \\
&+ \frac{9}{17}\, T_{3,\,-3,\,1} - \frac{99}{68}\, T_{3,\,-3,\,2} - \frac{10}{17}\, S_{4,\,1,\,1} + \frac{55}{17}\, S_{4,\,1,\,2}\bigg)\, x\, y + \big(-23868\, S_{4,\,-1,\,2} \\
&+ 7605\, T_{3,\,3,\,2}\big)\, x^6 + \big(\big(-47736\, S_{4,\,-1,\,2} + 45630\, T_{3,\,3,\,2}\big)\, z^2 + \big(-24336\, T_{4,\,1,\,1} \\
&+ 74880\, S_{3,\,-3,\,2}\big)\, z - 2184\, S_{4,\,-1,\,1} + 30576\, S_{4,\,-1,\,2} + 1716\, T_{3,\,3,\,1} - 11154\, T_{3,\,3,\,2}\big)\, x^4 \\
&+ \big(\big(198900\, S_{4,\,-1,\,2} + 38025\, T_{3,\,3,\,2}\big)\, z^4 + \big(-45968\, T_{4,\,1,\,1} + 74880\, S_{3,\,-3,\,2}\big)\, z^3 \\
&+ \big(2184\, S_{4,\,-1,\,1} - 30576\, S_{4,\,-1,\,2} + 5148\, T_{3,\,3,\,1} - 33462\, T_{3,\,3,\,2}\big)\, z^2 + \big(24024\, T_{4,\,1,\,1} \\
&- 4368\, T_{4,\,1,\,0} + 3744\, S_{3,\,-3,\,1} - 44928\, S_{3,\,-3,\,2}\big)\, z + 1560\, S_{4,\,-1,\,1} - 8580\, S_{4,\,-1,\,2} \\
&- 1404\, T_{3,\,3,\,1} + 3861\, T_{3,\,3,\,2} + 312\, T_{3,\,3,\,0}\big)\, x^2 + 14560\, \Big(\frac{153}{10}\, S_{4,\,-1,\,2}\, z^4 + \frac{39}{35}\, T_{4,\,1,\,1}\, z^3 \\
&+ \big(S_{4,\,-1,\,1} - 14\, S_{4,\,-1,\,2}\big)\, z^2 + \Big(-\frac{22}{35}\, T_{4,\,1,\,1} + \frac{4}{35}\, T_{4,\,1,\,0}\Big)\, z - \frac{3}{7}\, S_{4,\,-1,\,1} \\
&+ \frac{33}{14}\, S_{4,\,-1,\,2}\Big)\, z^2\Bigg)\, \sqrt{2} + \Big(\big(\big(49504\, S_{4,\,4,\,2} - 9360\, T_{5,\,-4,\,1}\big)\, z + 3432\, S_{5,\,4,\,1} \\
&- 1352\, T_{4,\,-4,\,1}\big)\, y^5 - 8736\, x\, \Big(\Big(\frac{195}{56}\, T_{5,\,4,\,1} + \frac{323}{21}\, S_{4,\,-4,\,2}\Big)\, z + S_{5,\,-4,\,1} \\
&+ \frac{13}{28}\, T_{4,\,4,\,1}\Big)\, y^4 + \big(\big(\big(-42432\, S_{4,\,4,\,2} + 28080\, T_{5,\,-4,\,1}\big)\, z - 624\, S_{5,\,4,\,1} \\
&+ 2704\, T_{4,\,-4,\,1}\big)\, x^2 + \big(63648\, S_{4,\,4,\,2} - 18720\, T_{5,\,-4,\,1}\big)\, z^3 + \big(14976\, S_{5,\,4,\,1}
\end{aligned}
$$

$$- 4056\, T_{4,\,-4,\,1}\big)\, z^2 + \big(2912\, S_{4,\,4,\,1} - 40768\, S_{4,\,4,\,2} + 8112\, T_{5,\,-4,\,1} - 1248\, T_{5,\,-4,\,0}\big)\, z$$

$$- 3744\, S_{5,\,4,\,1} + 1144\, T_{4,\,-4,\,1} - 208\, T_{4,\,-4,\,0}\big)\, y^3 - 16224\, \Bigg(\Bigg(\bigg(\Big(\frac{15}{52}\, T_{5,\,4,\,1} + \frac{34}{3}\, S_{4,\,-4,\,2}\Big)\, z$$

$$+ S_{5,\,-4,\,1} + \frac{1}{6}\, T_{4,\,4,\,1}\bigg)\, x^2 + \Big(\frac{45}{13}\, T_{5,\,4,\,1} + \frac{153}{13}\, S_{4,\,-4,\,2}\Big)\, z^3 + \Big(\frac{36}{13}\, S_{5,\,-4,\,1}$$

$$+ \frac{3}{4}\, T_{4,\,4,\,1}\Big)\, z^2 + \Big(-\frac{3}{2}\, T_{5,\,4,\,1} + \frac{3}{13}\, T_{5,\,4,\,0} + \frac{7}{13}\, S_{4,\,-4,\,1} - \frac{98}{13}\, S_{4,\,-4,\,2}\Big)\, z$$

$$- \frac{9}{13}\, S_{5,\,-4,\,1} - \frac{11}{52}\, T_{4,\,4,\,1} + \frac{1}{26}\, T_{4,\,4,\,0}\Bigg)\, x\, y^2 - 205088\, x^2\, \Bigg(\Bigg(\bigg(\Big(S_{4,\,4,\,2} - \frac{45}{493}\, T_{5,\,-4,\,1}\Big)\, z$$

$$+ \frac{159}{1972}\, S_{5,\,4,\,1} - \frac{39}{1972}\, T_{4,\,-4,\,1}\bigg)\, x^2 + \Big(\frac{27}{29}\, S_{4,\,4,\,2} - \frac{135}{493}\, T_{5,\,-4,\,1}\Big)\, z^3 + \Big(\frac{108}{493}\, S_{5,\,4,\,1}$$

$$- \frac{117}{1972}\, T_{4,\,-4,\,1}\Big)\, z^2 + \Big(\frac{21}{493}\, S_{4,\,4,\,1} - \frac{294}{493}\, S_{4,\,4,\,2} + \frac{117}{986}\, T_{5,\,-4,\,1} - \frac{9}{493}\, T_{5,\,-4,\,0}\Big)\, z$$

$$- \frac{27}{493}\, S_{5,\,4,\,1} + \frac{33}{1972}\, T_{4,\,-4,\,1} - \frac{3}{986}\, T_{4,\,-4,\,0}\Bigg)\, y + 4992\, \Bigg(\Bigg(\bigg(\Big(\frac{45}{32}\, T_{5,\,4,\,1}$$

$$+ \frac{51}{4}\, S_{4,\,-4,\,2}\Big)\, z + S_{5,\,-4,\,1} + \frac{13}{48}\, T_{4,\,4,\,1}\bigg)\, x^2 + \Big(\frac{15}{4}\, T_{5,\,4,\,1} + \frac{51}{4}\, S_{4,\,-4,\,2}\Big)\, z^3$$

$$+ \Big(3\, S_{5,\,-4,\,1} + \frac{13}{16}\, T_{4,\,4,\,1}\Big)\, z^2 + \Big(-\frac{13}{8}\, T_{5,\,4,\,1} + \frac{1}{4}\, T_{5,\,4,\,0} + \frac{7}{12}\, S_{4,\,-4,\,1}$$

$$- \frac{49}{6}\, S_{4,\,-4,\,2}\Big)\, z - \frac{3}{4}\, S_{5,\,-4,\,1} - \frac{11}{48}\, T_{4,\,4,\,1} + \frac{1}{24}\, T_{4,\,4,\,0}\Bigg)\, x^3\Bigg)\, \sqrt{7} + \big(77792\, z\, S_{4,\,2,\,2}$$

$$- 1352\, T_{4,\,-2,\,1}\big)\, y^5 - 282880\, \Big(S_{4,\,-2,\,2}\, z - \frac{39}{1360}\, T_{4,\,2,\,1}\Big)\, x\, y^4 + \big(\big(-254592\, z\, S_{4,\,2,\,2}$$

$$- 21632\, T_{4,\,-2,\,1}\big)\, x^2 - 297024\, S_{4,\,2,\,2}\, z^3 + 20280\, T_{4,\,-2,\,1}\, z^2 + \big(-1664\, S_{4,\,2,\,1}$$

$$+ 23296\, S_{4,\,2,\,2}\big)\, z + 1144\, T_{4,\,-2,\,1} - 208\, T_{4,\,-2,\,0}\big)\, y^3 - 155584\, \Big(\Big(S_{4,\,-2,\,2}\, z$$

$$+ \frac{13}{748}\, T_{4,\,2,\,1}\Big)\, x^2 + \frac{15}{22}\, S_{4,\,-2,\,2}\, z^3 - \frac{117}{374}\, T_{4,\,2,\,1}\, z^2 + \Big(\frac{27}{374}\, S_{4,\,-2,\,1} - \frac{189}{187}\, S_{4,\,-2,\,2}\Big)\, z$$

$$+ \frac{3}{68}\, T_{4,\,2,\,1} - \frac{3}{374}\, T_{4,\,2,\,0}\Big)\, x\, y^2 + \Big(\big(-332384\, z\, S_{4,\,2,\,2} - 20280\, T_{4,\,-2,\,1}\big)\, x^4 + \big($$

$$-1103232\, S_{4,\,2,\,2}\, z^3 - 36504\, T_{4,\,-2,\,1}\, z^2 + \big(-27456\, S_{4,\,2,\,1} + 384384\, S_{4,\,2,\,2}\big)\, z$$

$$+ 17160\, T_{4,\,-2,\,1} - 3120\, T_{4,\,-2,\,0}\big)\, x^2 - 34944\, \Big(\frac{459}{28}\, S_{4,\,2,\,2}\, z^4 - \frac{65}{56}\, T_{4,\,-2,\,1}\, z^3 + \big(S_{4,\,2,\,1}$$

$$- 14\, S_{4,\,2,\,2}\big)\, z^2 + \Big(\frac{33}{56}\, T_{4,\,-2,\,1} - \frac{3}{28}\, T_{4,\,-2,\,0}\Big)\, z - \frac{5}{14}\, S_{4,\,2,\,1} + \frac{55}{28}\, S_{4,\,2,\,2}\Big)\, z\Big)\, y$$

$$+ 127296\, x\, \Big(\Big(S_{4,\,-2,\,2}\, z - \frac{13}{153}\, T_{4,\,2,\,1}\Big)\, x^4 + \Big(\frac{11}{2}\, S_{4,\,-2,\,2}\, z^3 - \frac{13}{204}\, T_{4,\,2,\,1}\, z^2$$

$$+ \Big(\frac{35}{306}\, S_{4,\,-2,\,1} - \frac{245}{153}\, S_{4,\,-2,\,2}\Big)\, z + \frac{11}{153}\, T_{4,\,2,\,1} - \frac{2}{153}\, T_{4,\,2,\,0}\Big)\, x^2$$

$$+ \frac{14}{51}\, \Big(\frac{459}{28}\, S_{4,\,-2,\,2}\, z^4 + \frac{65}{56}\, T_{4,\,2,\,1}\, z^3 + \big(S_{4,\,-2,\,1} - 14\, S_{4,\,-2,\,2}\big)\, z^2 + \Big(-\frac{33}{56}\, T_{4,\,2,\,1}$$

$$+ \frac{3}{28} T_{4, 2, 0}\Big) z - \frac{5}{14} S_{4, -2, 1} + \frac{55}{28} S_{4, -2, 2}\Big) z\Big)\Big) \sqrt{5} + \frac{1}{416} \Big(55770 x^4 T_{1, 1, 3} + \Big(\Big($$

$$-223080 T_{1, 0, 3} - 121680 T_{3, 0, 2} + 257400 T_{5, 0, 1}\Big) z - 44616 T_{2, 0, 2} + 40560 T_{4, 0, 1}$$

$$- 10920 T_{6, 0, 0}\Big) x^3 + \Big(334620 z^2 T_{1, 1, 3} + 730080 z S_{1, -1, 3} + 10296 T_{1, 1, 2}$$

$$- 66924 T_{1, 1, 3}\Big) x^2 + \Big(\Big(-223080 T_{1, 0, 3} - 425880 T_{3, 0, 2} + 93600 T_{5, 0, 1}\Big) z^3 + \Big($$

$$-133848 T_{2, 0, 2} - 20280 T_{4, 0, 1} + 65520 T_{6, 0, 0}\Big) z^2 + \Big(-20592 T_{1, 0, 2} + 133848 T_{1, 0, 3}$$

$$- 20592 T_{3, 0, 1} + 133848 T_{3, 0, 2} - 121680 T_{5, 0, 1} + 18720 T_{5, 0, 0} - 189000\Big) z$$

$$- 5616 T_{2, 0, 1} + 30888 T_{2, 0, 2} - 17160 T_{4, 0, 1} + 3120 T_{4, 0, 0}\Big) x + 278850 z^4 T_{1, 1, 3}$$

$$+ 730080 z^3 S_{1, -1, 3} + \Big(30888 T_{1, 1, 2} - 200772 T_{1, 1, 3}\Big) z^2 + \Big(36608 S_{1, -1, 2}$$

$$- 313664 S_{1, -1, 3}\Big) z + 1456 T_{1, 1, 1} - 6552 T_{1, 1, 2} + 18018 T_{1, 1, 3}\Big) y^2 + \frac{1}{416} \Big(\Big($$

$$-111540 T_{1, 0, 3} - 60840 T_{3, 0, 2} + 128700 T_{5, 0, 1}\Big) z - 22308 T_{2, 0, 2} + 20280 T_{4, 0, 1}$$

$$- 5460 T_{6, 0, 0}\Big) x^5 + \frac{1}{416} \Big(\Big(-111540 T_{1, 0, 3} - 365040 T_{3, 0, 2} - 280800 T_{5, 0, 1}\Big) z^5 + \Big($$

$$-111540 T_{2, 0, 2} - 135200 T_{4, 0, 1} - 43680 T_{6, 0, 0}\Big) z^4 + \Big(-20592 T_{1, 0, 2} + 133848 T_{1, 0, 3}$$

$$- 54912 T_{3, 0, 1} + 356928 T_{3, 0, 2} + 162240 T_{5, 0, 1} - 24960 T_{5, 0, 0} - 157500\Big) z^3 + \Big($$

$$-16848 T_{2, 0, 1} + 92664 T_{2, 0, 2} + 68640 T_{4, 0, 1} - 12480 T_{4, 0, 0}\Big) z^2 + \Big(-2912 T_{1, 0, 1}$$

$$+ 13104 T_{1, 0, 2} - 36036 T_{1, 0, 3} + 22464 T_{3, 0, 1} - 61776 T_{3, 0, 2} - 4992 T_{3, 0, 0} + 132300\Big) z$$

$$+ 4368 T_{2, 0, 1} - 9828 T_{2, 0, 2} - 1248 T_{2, 0, 0}\Big) x + \frac{1}{416} \Big(25740 T_{1, 1, 2} - 167310 T_{1, 1, 3}\Big) z^4$$

$$+ \frac{1}{416} \Big(54912 S_{1, -1, 2} - 470496 S_{1, -1, 3}\Big) z^3 + \frac{1}{416} \Big(4368 T_{1, 1, 1} - 19656 T_{1, 1, 2}$$

$$+ 54054 T_{1, 1, 3}\Big) z^2 + \frac{1}{416} \Big(\Big(-716040 S_{2, 0, 3} + 106080 S_{4, 0, 2} + 98280 S_{6, 0, 1}\Big) z$$

$$- 60840 S_{1, 0, 3} - 18720 S_{3, 0, 2} + 17160 S_{5, 0, 1}\Big) y^5 + \frac{1}{416} \Big(3328 S_{1, -1, 1} - 26624 S_{1, -1, 2}$$

$$+ 90272 S_{1, -1, 3}\Big) z + \frac{1}{416} \Big(\Big(29016 z S_{6, 4, 1} + 312 T_{6, -4, 0}\Big) y^5 - 65520 \Big(S_{6, -4, 1} z$$

$$+ \frac{1}{84} T_{6, 4, 0}\Big) x y^4 + \Big(\Big(28080 z S_{6, 4, 1} + 6240 T_{6, -4, 0}\Big) x^2 + 112320 S_{6, 4, 1} z^3$$

$$- 9360 z^2 T_{6, -4, 0} - 43680 S_{6, 4, 1} z\Big) y^3 - 187200 x \Big(\Big(S_{6, -4, 1} z - \frac{7}{120} T_{6, 4, 0}\Big) x^2$$

$$+\frac{9}{5}\left(S_{6,\,-4,\,1} z^2+\frac{1}{12} z\, T_{6,\,4,\,0}-\frac{7}{18} S_{6,\,-4,\,1}\right) z\Big) y^2-173160\left(\left(S_{6,\,4,\,1} z\right.\right.$$

$$\left.+\frac{5}{111} T_{6,\,-4,\,0}\right) x^2+\frac{72}{37}\left(S_{6,\,4,\,1} z^2-\frac{1}{12} z\, T_{6,\,-4,\,0}-\frac{7}{18} S_{6,\,4,\,1}\right) z\Big) x^2 y$$

$$+50544\left(\left(S_{6,\,-4,\,1} z-\frac{13}{324} T_{6,\,4,\,0}\right) x^2+\frac{20}{9}\left(S_{6,\,-4,\,1} z^2+\frac{1}{12} z\, T_{6,\,4,\,0}\right.\right.$$

$$\left.\left.-\frac{7}{18} S_{6,\,-4,\,1}\right) z\right) x^3\Big)\sqrt{7}+\frac{1}{416}\left(167310 z^2 T_{1,\,1,\,3}+486720 z\, S_{1,\,-1,\,3}+5148 T_{1,\,1,\,2}\right.$$

$$\left.-33462 T_{1,\,1,\,3}\right) x^4+\frac{1}{416}\left(\left(-223080 T_{1,\,0,\,3}-425880 T_{3,\,0,\,2}+93600 T_{5,\,0,\,1}\right) z^3+\left(\right.\right.$$

$$-133848 T_{2,\,0,\,2}-20280 T_{4,\,0,\,1}+65520 T_{6,\,0,\,0}\big) z^2+\left(-20592 T_{1,\,0,\,2}+133848 T_{1,\,0,\,3}\right.$$

$$\left.-20592 T_{3,\,0,\,1}+133848 T_{3,\,0,\,2}-121680 T_{5,\,0,\,1}+18720 T_{5,\,0,\,0}-126000\right) z$$

$$\left.-5616 T_{2,\,0,\,1}+30888 T_{2,\,0,\,2}-17160 T_{4,\,0,\,1}+3120 T_{4,\,0,\,0}\right) x^3+\frac{1}{416}\left(278850 z^4 T_{1,\,1,\,3}\right.$$

$$+973440 z^3 S_{1,\,-1,\,3}+\left(30888 T_{1,\,1,\,2}-200772 T_{1,\,1,\,3}\right) z^2+\left(54912 S_{1,\,-1,\,2}\right.$$

$$\left.\left.-470496 S_{1,\,-1,\,3}\right) z+1456 T_{1,\,1,\,1}-6552 T_{1,\,1,\,2}+18018 T_{1,\,1,\,3}\right) x^2+\frac{1}{416}\left(\left(\right.\right.$$

$$-2535 y^6 T_{3,\,1,\,2}-25350 x\, y^5 T_{3,\,-1,\,2}+\left(-32955 x^2 T_{3,\,1,\,2}+15210 z^2 T_{3,\,1,\,2}\right.$$

$$\left.-81120 z\, S_{3,\,-1,\,2}-572 T_{3,\,1,\,1}+3718 T_{3,\,1,\,2}\right) y^4-50700\left(x^2 T_{3,\,-1,\,2}+3 T_{3,\,-1,\,2} z^2\right.$$

$$\left.+\frac{136}{65} z\, S_{3,\,1,\,2}+\frac{22}{195} T_{3,\,-1,\,1}-\frac{11}{15} T_{3,\,-1,\,2}\right) x\, y^3+\left(-58305 x^4 T_{3,\,1,\,2}+\left(\right.\right.$$

$$\left.-121680 z^2 T_{3,\,1,\,2}-56160 z\, S_{3,\,-1,\,2}-6864 T_{3,\,1,\,1}+44616 T_{3,\,1,\,2}\right) x^2+88725 T_{3,\,1,\,2} z^4$$

$$-24960 z^3 S_{3,\,-1,\,2}+\left(5148 T_{3,\,1,\,1}-33462 T_{3,\,1,\,2}\right) z^2+\left(-3744 S_{3,\,-1,\,1}\right.$$

$$\left.\left.+44928 S_{3,\,-1,\,2}\right) z+468 T_{3,\,1,\,1}-1287 T_{3,\,1,\,2}-104 T_{3,\,1,\,0}\right) y^2-25350 x\left(x^4 T_{3,\,-1,\,2}\right.$$

$$+\left(6 T_{3,\,-1,\,2} z^2+\frac{272}{65} z\, S_{3,\,1,\,2}+\frac{44}{195} T_{3,\,-1,\,1}-\frac{22}{15} T_{3,\,-1,\,2}\right) x^2+5 z^4 T_{3,\,-1,\,2}$$

$$+\frac{512}{65} z^3 S_{3,\,1,\,2}+\left(\frac{44}{65} T_{3,\,-1,\,1}-\frac{22}{5} T_{3,\,-1,\,2}\right) z^2+\left(\frac{96}{325} S_{3,\,1,\,1}-\frac{1152}{325} S_{3,\,1,\,2}\right) z$$

$$\left.-\frac{12}{65} T_{3,\,-1,\,1}+\frac{33}{65} T_{3,\,-1,\,2}+\frac{8}{195} T_{3,\,-1,\,0}\right) y-27885 x^6 T_{3,\,1,\,2}+\left(-136890 z^2 T_{3,\,1,\,2}\right.$$

$$\left.+24960 z\, S_{3,\,-1,\,2}-6292 T_{3,\,1,\,1}+40898 T_{3,\,1,\,2}\right) x^4+\left(-38025 T_{3,\,1,\,2} z^4\right.$$

$$+174720 z^3 S_{3,\,-1,\,2}+\left(-12012 T_{3,\,1,\,1}+78078 T_{3,\,1,\,2}\right) z^2+\left(3744 S_{3,\,-1,\,1}\right.$$

$$\left.\left.-44928 S_{3,\,-1,\,2}\right) z+5148 T_{3,\,1,\,1}-14157 T_{3,\,1,\,2}-1144 T_{3,\,1,\,0}\right) x^2$$

$$
\begin{aligned}
&+11440\left(\frac{273}{44}T_{3,1,2}z^5+\frac{144}{11}z^4S_{3,-1,2}+\left(T_{3,1,1}-\frac{13}{2}T_{3,1,2}\right)z^3+\left(\frac{48}{55}S_{3,-1,1}\right.\right.\\
&-\frac{576}{55}S_{3,-1,2}\Big)z^2+\left(-\frac{27}{55}T_{3,1,1}+\frac{27}{20}T_{3,1,2}+\frac{6}{55}T_{3,1,0}\right)z-\frac{16}{55}S_{3,-1,1}\\
&\left.\left.+\frac{72}{55}S_{3,-1,2}\right)z\right)\sqrt{2}+\Big(8580\,y^6S_{6,-1,1}+6240\,x\,y^5S_{6,1,1}+\big(19500\,x^2S_{6,-1,1}\\
&-11700\,z^2S_{6,-1,1}+520\,z\,T_{6,1,0}-9100\,S_{6,-1,1}\big)y^4+12480\left(x^2S_{6,1,1}+\frac{3}{4}S_{6,1,1}z^2\right.\\
&\left.+\frac{2}{3}z\,T_{6,-1,0}-\frac{7}{12}S_{6,1,1}\right)x\,y^3+\big(13260\,x^4S_{6,-1,1}+\big(-32760\,z^2S_{6,-1,1}\\
&+9360\,z\,T_{6,1,0}-10920\,S_{6,-1,1}\big)x^2-146640\,S_{6,-1,1}z^4-4160\,z^3T_{6,1,0}\\
&+65520\,S_{6,-1,1}z^2\big)y^2+6240\left(x^4S_{6,1,1}+\left(\frac{3}{2}S_{6,1,1}z^2+\frac{4}{3}z\,T_{6,-1,0}-\frac{7}{6}S_{6,1,1}\right)x^2\right.\\
&\left.-19\,S_{6,1,1}z^4-\frac{8}{3}z^3T_{6,-1,0}+7\,S_{6,1,1}z^2\right)x\,y+2340\,x^6S_{6,-1,1}+\big(-21060\,z^2S_{6,-1,1}\\
&+8840\,z\,T_{6,1,0}-1820\,S_{6,-1,1}\big)x^4-28080\left(S_{6,-1,1}z^2+\frac{20}{27}z\,T_{6,1,0}-\frac{7}{9}S_{6,-1,1}\right)z^2x^2\\
&+26208\left(S_{6,-1,1}z^2+\frac{2}{21}z\,T_{6,1,0}-\frac{5}{9}S_{6,-1,1}\right)z^4\Big)\sqrt{7}-39780\,y^6S_{2,-1,3}+\big(\\
&-159120\,x\,S_{2,1,3}-397800\,z\,S_{2,2,3}+7436\,T_{2,-2,2}\big)y^5+\big(39780\,x^2S_{2,-1,3}\\
&+\big(79560\,z\,S_{2,-2,3}+7436\,T_{2,2,2}\big)x+119340\,z^2S_{2,-1,3}+14872\,T_{2,1,2}z-2704\,S_{2,-1,2}\\
&+27300\,S_{2,-1,3}\big)y^4+\big(-318240\,x^3S_{2,1,3}+\big(-1432080\,z\,S_{2,2,3}+14872\,T_{2,-2,2}\big)x^2+\big(\\
&-954720\,z^2S_{2,1,3}-29744\,z\,T_{2,-1,2}-21632\,S_{2,1,2}+218400\,S_{2,1,3}\big)x\\
&-1113840\,z^3S_{2,2,3}+44616\,z^2T_{2,-2,2}+\big(-54080\,S_{2,2,2}+546000\,S_{2,2,3}\big)z\\
&+1872\,T_{2,-2,1}-10296\,T_{2,-2,2}\big)y^3+\big(198900\,x^4S_{2,-1,3}+\big(795600\,z\,S_{2,-2,3}\\
&+14872\,T_{2,2,2}\big)x^3+\big(1193400\,z^2S_{2,-1,3}+16224\,S_{2,-1,2}-163800\,S_{2,-1,3}\big)x^2\\
&+\big(795600\,z^3S_{2,-2,3}+44616\,T_{2,2,2}z^2+\big(32448\,S_{2,-2,2}-327600\,S_{2,-2,3}\big)z\\
&+1872\,T_{2,2,1}-10296\,T_{2,2,2}\big)x+994500\,z^4S_{2,-1,3}+59488\,T_{2,1,2}z^3+\big(48672\,S_{2,-1,2}\\
&-491400\,S_{2,-1,3}\big)z^2+\big(3744\,T_{2,1,1}-20592\,T_{2,1,2}\big)z+416\,S_{2,-1,1}-4160\,S_{2,-1,2}\\
&+17316\,S_{2,-1,3}\big)y^2+\big(-159120\,x^5S_{2,1,3}+\big(-1034280\,z\,S_{2,2,3}+7436\,T_{2,-2,2}\big)x^4+\big(\\
&-954720\,z^2S_{2,1,3}-29744\,z\,T_{2,-1,2}-21632\,S_{2,1,2}+218400\,S_{2,1,3}\big)x^3+\big(
\end{aligned}
$$

$$-1750320 z^3 S_{2, 2, 3} + 44616 z^2 T_{2, -2, 2} + \left(-97344 S_{2, 2, 2} + 982800 S_{2, 2, 3}\right) z$$

$$+ 1872 T_{2, -2, 1} - 10296 T_{2, -2, 2}\Big) x^2 + \Big(-795600 z^4 S_{2, 1, 3} - 29744 z^3 T_{2, -1, 2} + \Big($$

$$-64896 S_{2, 1, 2} + 655200 S_{2, 1, 3}\Big) z^2 + \left(-3744 T_{2, -1, 1} + 20592 T_{2, -1, 2}\right) z - 1664 S_{2, 1, 1}$$

$$+ 16640 S_{2, 1, 2} - 69264 S_{2, 1, 3}\Big) x - 716040 z^5 S_{2, 2, 3} + 37180 z^4 T_{2, -2, 2} + \Big(-75712 S_{2, 2, 2}$$

$$+ 764400 S_{2, 2, 3}\Big) z^3 + \left(5616 T_{2, -2, 1} - 30888 T_{2, -2, 2}\right) z^2 + \Big(-4160 S_{2, 2, 1} + 41600 S_{2, 2, 2}$$

$$- 173160 S_{2, 2, 3}\Big) z - 1456 T_{2, -2, 1} + 3276 T_{2, -2, 2} + 416 T_{2, -2, 0}\Big) y + 119340 x^6 S_{2, -1, 3}$$

$$+ \left(716040 z S_{2, -2, 3} + 7436 T_{2, 2, 2}\right) x^5 + \Big(1074060 z^2 S_{2, -1, 3} - 14872 z T_{2, 1, 2}$$

$$+ 18928 S_{2, -1, 2} - 191100 S_{2, -1, 3}\Big) x^4 + \Big(1432080 z^3 S_{2, -2, 3} + 44616 T_{2, 2, 2} z^2$$

$$+ \left(75712 S_{2, -2, 2} - 764400 S_{2, -2, 3}\right) z + 1872 T_{2, 2, 1} - 10296 T_{2, 2, 2}\Big) x^3$$

$$+ \Big(1790100 z^4 S_{2, -1, 3} + 29744 T_{2, 1, 2} z^3 + \left(113568 S_{2, -1, 2} - 1146600 S_{2, -1, 3}\right) z^2$$

$$+ 2080 S_{2, -1, 1} - 20800 S_{2, -1, 2} + 86580 S_{2, -1, 3}\Big) x^2 + \Big(716040 z^5 S_{2, -2, 3}$$

$$+ 37180 T_{2, 2, 2} z^4 + \left(75712 S_{2, -2, 2} - 764400 S_{2, -2, 3}\right) z^3 + \Big(5616 T_{2, 2, 1}$$

$$- 30888 T_{2, 2, 2}\Big) z^2 + \left(4160 S_{2, -2, 1} - 41600 S_{2, -2, 2} + 173160 S_{2, -2, 3}\right) z - 1456 T_{2, 2, 1}$$

$$+ 3276 T_{2, 2, 2} + 416 T_{2, 2, 0}\Big) x + 835380 z^6 S_{2, -1, 3} + 44616 z^5 T_{2, 1, 2} + \Big(94640 S_{2, -1, 2}$$

$$- 955500 S_{2, -1, 3}\Big) z^4 + \left(7488 T_{2, 1, 1} - 41184 T_{2, 1, 2}\right) z^3 + \Big(6240 S_{2, -1, 1} - 62400 S_{2, -1, 2}$$

$$+ 259740 S_{2, -1, 3}\Big) z^2 + \left(-2912 T_{2, 1, 1} + 6552 T_{2, 1, 2} + 832 T_{2, 1, 0}\right) z - 1248 S_{2, -1, 1}$$

$$+ 4368 S_{2, -1, 2} - 9828 S_{2, -1, 3}\Big) \sqrt{3} + \frac{1}{416} \Big(55770 x^2 T_{1, 1, 3} + \Big(\Big(-111540 T_{1, 0, 3}$$

$$- 60840 T_{3, 0, 2} + 128700 T_{5, 0, 1}\Big) z - 22308 T_{2, 0, 2} + 20280 T_{4, 0, 1} - 5460 T_{6, 0, 0}\Big) x$$

$$+ 167310 z^2 T_{1, 1, 3} + 243360 z S_{1, -1, 3} + 5148 T_{1, 1, 2} - 33462 T_{1, 1, 3}\Big) y^4 - \frac{105}{16} T_{1, 1, 3}$$

$$+ \frac{35}{8} T_{1, 1, 2} + 1170 z^5 S_{1, -1, 3} - \frac{5}{2} T_{1, 1, 1} + T_{1, 1, 0} + \frac{1}{416} \Big(\Big(\Big(-716040 S_{2, 0, 3}$$

$$+ 106080 S_{4, 0, 2} + 98280 S_{6, 0, 1}\Big) z - 60840 S_{1, 0, 3} - 18720 S_{3, 0, 2} + 17160 S_{5, 0, 1}\Big) x^4$$

$$- 243360 x^3 z S_{1, 1, 3} + \Big(\Big(-2386800 S_{2, 0, 3} - 884000 S_{4, 0, 2} + 262080 S_{6, 0, 1}\Big) z^3 + \Big($$

$$-365040\,S_{1,\,0,\,3}-393120\,S_{3,\,0,\,2}+37440\,S_{5,\,0,\,1}\big)\,z^{2}+\big(-97344\,S_{2,\,0,\,2}+982800\,S_{2,\,0,\,3}$$

$$-6240\,S_{4,\,0,\,1}+87360\,S_{4,\,0,\,2}-131040\,S_{6,\,0,\,1}\big)\,z-9152\,S_{1,\,0,\,2}+78416\,S_{1,\,0,\,3}$$

$$-3744\,S_{3,\,0,\,1}+44928\,S_{3,\,0,\,2}-18720\,S_{5,\,0,\,1}\big)\,x^{2}-243360\,\Big(z^{2}\,S_{1,\,1,\,3}+\frac{44}{585}\,S_{1,\,1,\,2}$$

$$-\frac{29}{45}\,S_{1,\,1,\,3}\Big)\,z\,x+\big(-1670760\,S_{2,\,0,\,3}-1485120\,S_{4,\,0,\,2}-366912\,S_{6,\,0,\,1}\big)\,z^{5}+\big($$

$$-304200\,S_{1,\,0,\,3}-561600\,S_{3,\,0,\,2}-187200\,S_{5,\,0,\,1}\big)\,z^{4}+\big(-162240\,S_{2,\,0,\,2}+1638000\,S_{2,\,0,\,3}$$

$$-83200\,S_{4,\,0,\,1}+1164800\,S_{4,\,0,\,2}+174720\,S_{6,\,0,\,1}\big)\,z^{3}+\big(-27456\,S_{1,\,0,\,2}+235248\,S_{1,\,0,\,3}$$

$$-29952\,S_{3,\,0,\,1}+359424\,S_{3,\,0,\,2}+74880\,S_{5,\,0,\,1}\big)\,z^{2}+\big(-7488\,S_{2,\,0,\,1}+74880\,S_{2,\,0,\,2}$$

$$-311688\,S_{2,\,0,\,3}+24960\,S_{4,\,0,\,1}-137280\,S_{4,\,0,\,2}\big)\,z-832\,S_{1,\,0,\,1}+6656\,S_{1,\,0,\,2}$$

$$-22568\,S_{1,\,0,\,3}+4992\,S_{3,\,0,\,1}-22464\,S_{3,\,0,\,2}\big)\,y+\frac{1}{416}\,\Big(\Big(\big(\big(-1432080\,S_{2,\,0,\,3}$$

$$+212160\,S_{4,\,0,\,2}+196560\,S_{6,\,0,\,1}\big)\,z-121680\,S_{1,\,0,\,3}-37440\,S_{3,\,0,\,2}+34320\,S_{5,\,0,\,1}\big)\,x^{2}$$

$$-243360\,x\,z\,S_{1,\,1,\,3}+\big(-2386800\,S_{2,\,0,\,3}-884000\,S_{4,\,0,\,2}+262080\,S_{6,\,0,\,1}\big)\,z^{3}+\big($$

$$-365040\,S_{1,\,0,\,3}-393120\,S_{3,\,0,\,2}+37440\,S_{5,\,0,\,1}\big)\,z^{2}+\big(-97344\,S_{2,\,0,\,2}+982800\,S_{2,\,0,\,3}$$

$$-6240\,S_{4,\,0,\,1}+87360\,S_{4,\,0,\,2}-131040\,S_{6,\,0,\,1}\big)\,z-9152\,S_{1,\,0,\,2}+78416\,S_{1,\,0,\,3}$$

$$-3744\,S_{3,\,0,\,1}+44928\,S_{3,\,0,\,2}-18720\,S_{5,\,0,\,1}\big)\,y^{3}+\frac{715}{16}\,y^{6}\,T_{1,\,1,\,3}+\frac{5005}{16}\,z^{6}\,T_{1,\,1,\,3}$$

$$+\frac{715}{16}\,x^{6}\,T_{1,\,1,\,3}\Big)\bar{e}_{y}+\Big(\frac{1}{208}\,\Big(\Big(-1950\,\Big(x^{6}\,S_{6,\,2,\,1}+\Big(2\,S_{6,\,-2,\,1}\,y-3\,S_{6,\,3,\,1}\,z$$

$$+\frac{3}{50}\,T_{6,\,-3,\,0}\Big)\,x^{5}+\Big(S_{6,\,2,\,1}\,y^{2}+\Big(-\frac{9}{50}\,T_{6,\,3,\,0}-9\,S_{6,\,-3,\,1}\,z\Big)\,y+\frac{16}{75}\,z\,T_{6,\,-2,\,0}$$

$$-\frac{24}{5}\,S_{6,\,2,\,1}\,z^{2}-\frac{56}{75}\,S_{6,\,2,\,1}\Big)\,x^{4}+\Big(4\,S_{6,\,-2,\,1}\,y^{3}+\Big(-\frac{3}{25}\,T_{6,\,-3,\,0}+6\,S_{6,\,3,\,1}\,z\Big)\,y^{2}+\Big($$

$$-\frac{112}{75}\,S_{6,\,-2,\,1}-\frac{32}{75}\,z\,T_{6,\,2,\,0}-\frac{48}{5}\,S_{6,\,-2,\,1}\,z^{2}\Big)\,y-\frac{16}{5}\,\Big(S_{6,\,3,\,1}\,z^{2}+\frac{3}{20}\,z\,T_{6,\,-3,\,0}$$

$$-\frac{7}{10}\,S_{6,\,3,\,1}\Big)\,z\Big)\,x^{3}+\Big(-S_{6,\,2,\,1}\,y^{4}+\Big(-6\,S_{6,\,-3,\,1}\,z-\frac{3}{25}\,T_{6,\,3,\,0}\Big)\,y^{3}-\frac{48}{5}\,\Big(S_{6,\,-3,\,1}\,z^{2}$$

$$-\frac{3}{20}\,z\,T_{6,\,3,\,0}-\frac{7}{10}\,S_{6,\,-3,\,1}\Big)\,z\,y-\frac{32}{5}\,\Big(S_{6,\,2,\,1}\,z^{2}+\frac{1}{15}\,z\,T_{6,\,-2,\,0}-\frac{7}{10}\,S_{6,\,2,\,1}\Big)\,z^{2}\Big)\,x^{2}$$

$$+2\,y\,\Big(S_{6,\,-2,\,1}\,y^{4}+\Big(\frac{9}{2}\,S_{6,\,3,\,1}\,z-\frac{9}{100}\,T_{6,\,-3,\,0}\Big)\,y^{3}+\Big(-\frac{24}{5}\,S_{6,\,-2,\,1}\,z^{2}-\frac{16}{75}\,z\,T_{6,\,2,\,0}$$

$$-\frac{56}{75}\,S_{6,\,-2,\,1}\Big)\,y^{2}+\frac{24}{5}\,\Big(S_{6,\,3,\,1}\,z^{2}+\frac{3}{20}\,z\,T_{6,\,-3,\,0}-\frac{7}{10}\,S_{6,\,3,\,1}\Big)\,z\,y-\frac{32}{5}\,\Big(S_{6,\,-2,\,1}\,z^{2}$$

$$- \frac{1}{15} z T_{6, 2, 0} - \frac{7}{10} S_{6, -2, 1} \Big) z^2 \Big) x + 3 \Big(- \frac{1}{3} S_{6, 2, 1} y^4 + \Big(\frac{1}{50} T_{6, 3, 0} + S_{6, -3, 1} z \Big) y^3$$

$$+ \Big(\frac{8}{5} S_{6, 2, 1} z^2 - \frac{16}{225} z T_{6, -2, 0} + \frac{56}{225} S_{6, 2, 1} \Big) y^2 + \frac{16}{15} \Big(S_{6, -3, 1} z^2 - \frac{3}{20} z T_{6, 3, 0}$$

$$- \frac{7}{10} S_{6, -3, 1} \Big) z y + \frac{32}{15} \Big(S_{6, 2, 1} z^2 + \frac{1}{15} z T_{6, -2, 0} - \frac{7}{10} S_{6, 2, 1} \Big) z^2 \Big) y^2 \Big) \sqrt{7} \sqrt{2} + \Big($$

$$-780 x^6 T_{5, -2, 1} + 1560 y x^5 T_{5, 2, 1} + \Big(-780 y^2 T_{5, -2, 1} + 2340 z^2 T_{5, -2, 1} + 5200 z S_{5, 2, 1}$$

$$- 104 T_{5, -2, 0} + 676 T_{5, -2, 1} \Big) x^4 + 3120 y \Big(T_{5, 2, 1} y^2 - \frac{3}{2} T_{5, 2, 1} z^2 + \frac{10}{3} z S_{5, -2, 1}$$

$$- \frac{13}{30} T_{5, 2, 1} + \frac{1}{15} T_{5, 2, 0} \Big) x^3 + \Big(780 y^4 T_{5, -2, 1} + 7800 \Big(T_{5, -2, 1} z^3 + \frac{56}{75} S_{5, 2, 1} z^2 + \Big($$

$$- \frac{13}{25} T_{5, -2, 1} + \frac{2}{25} T_{5, -2, 0} \Big) z - \frac{12}{25} S_{5, 2, 1} \Big) z \Big) x^2 + 1560 y \Big(T_{5, 2, 1} y^4 + \Big(-3 T_{5, 2, 1} z^2$$

$$+ \frac{20}{3} z S_{5, -2, 1} - \frac{13}{15} T_{5, 2, 1} + \frac{2}{15} T_{5, 2, 0} \Big) y^2 - 10 \Big(T_{5, 2, 1} z^3 - \frac{56}{75} S_{5, -2, 1} z^2 + \Big($$

$$- \frac{13}{25} T_{5, 2, 1} + \frac{2}{25} T_{5, 2, 0} \Big) z + \frac{12}{25} S_{5, -2, 1} \Big) z \Big) x + 780 y^2 \Big(y^4 T_{5, -2, 1} + \Big(-3 T_{5, -2, 1} z^2$$

$$- \frac{20}{3} S_{5, 2, 1} z - \frac{13}{15} T_{5, -2, 1} + \frac{2}{15} T_{5, -2, 0} \Big) y^2 - 10 \Big(T_{5, -2, 1} z^3 + \frac{56}{75} S_{5, 2, 1} z^2 + \Big($$

$$- \frac{13}{25} T_{5, -2, 1} + \frac{2}{25} T_{5, -2, 0} \Big) z - \frac{12}{25} S_{5, 2, 1} \Big) z \Big) \Big) \sqrt{7} + 5070 x^6 T_{3, -2, 2} + ($$

$$-10140 y T_{3, 2, 2} - 4290 z T_{5, -1, 1} - 5252 S_{5, 1, 1}) x^5 + (5070 T_{3, -2, 2} y^2 + (4290 z T_{5, 1, 1}$$

$$- 5252 S_{5, -1, 1}) y + 30420 T_{3, -2, 2} z^2 + 6240 z S_{3, 2, 2} + 1144 T_{3, -2, 1} - 7436 T_{3, -2, 2}) x^4$$

$$+ (-20280 T_{3, 2, 2} y^3 + (-8580 z T_{5, -1, 1} - 10504 S_{5, 1, 1}) y^2 + (-60840 z^2 T_{3, 2, 2}$$

$$+ 12480 z S_{3, -2, 2} - 2288 T_{3, 2, 1} + 14872 T_{3, 2, 2}) y - 3120 T_{5, -1, 1} z^3 + 14352 S_{5, 1, 1} z^2$$

$$+ (4056 T_{5, -1, 1} - 624 T_{5, -1, 0}) z + 3744 S_{5, 1, 1}) x^3 + (-5070 y^4 T_{3, -2, 2} + (8580 z T_{5, 1, 1}$$

$$- 10504 S_{5, -1, 1}) y^3 + (3120 T_{5, 1, 1} z^3 + 14352 S_{5, -1, 1} z^2 + (-4056 T_{5, 1, 1} + 624 T_{5, 1, 0}) z$$

$$+ 3744 S_{5, -1, 1}) y + 25350 T_{3, -2, 2} z^4 - 12480 z^3 S_{3, 2, 2} + (3432 T_{3, -2, 1}$$

$$- 22308 T_{3, -2, 2}) z^2 - 936 T_{3, -2, 1} + 2574 T_{3, -2, 2} + 208 T_{3, -2, 0}) x^2 + \Big(-10140 T_{3, 2, 2} y^5$$

$$+ (-4290 z T_{5, -1, 1} - 5252 S_{5, 1, 1}) y^4 + (-60840 z^2 T_{3, 2, 2} + 12480 z S_{3, -2, 2}$$

$$- 2288 T_{3, 2, 1} + 14872 T_{3, 2, 2}) y^3 + (-3120 T_{5, -1, 1} z^3 + 14352 S_{5, 1, 1} z^2 + (4056 T_{5, -1, 1}$$

$$- 624 T_{5, -1, 0}) z + 3744 S_{5, 1, 1}) y^2 + (-50700 z^4 T_{3, 2, 2} - 24960 z^3 S_{3, -2, 2} + ($$

$$-6864\, T_{3,\, 2,\, 1} + 44616\, T_{3,\, 2,\, 2}\big) z^2 + 1872\, T_{3,\, 2,\, 1} - 5148\, T_{3,\, 2,\, 2} - 416\, T_{3,\, 2,\, 0}\big)\, y$$

$$+ 9360 \left(T_{5,\, -1,\, 1} z^3 + \frac{22}{9} S_{5,\, 1,\, 1} z^2 + \left(-\frac{26}{45} T_{5,\, -1,\, 1} + \frac{4}{45} T_{5,\, -1,\, 0} \right) z - \frac{8}{5} S_{5,\, 1,\, 1} \right) z^2 \Big)\, x$$

$$- 1144\, y \left(\frac{195}{44} T_{3,\, -2,\, 2}\, y^5 + \left(-\frac{15}{4} T_{5,\, 1,\, 1}\, z + \frac{101}{22} S_{5,\, -1,\, 1} \right) y^4 + \left(\frac{585}{22} T_{3,\, -2,\, 2}\, z^2 \right.\right.$$

$$\left. + \frac{60}{11} z\, S_{3,\, 2,\, 2} + T_{3,\, -2,\, 1} - \frac{13}{2} T_{3,\, -2,\, 2} \right) y^3 + \left(-\frac{30}{11} T_{5,\, 1,\, 1} z^3 - \frac{138}{11} S_{5,\, -1,\, 1} z^2 \right.$$

$$\left. + \left(\frac{39}{11} T_{5,\, 1,\, 1} - \frac{6}{11} T_{5,\, 1,\, 0} \right) z - \frac{36}{11} S_{5,\, -1,\, 1} \right) y^2 + \left(\frac{975}{44} T_{3,\, -2,\, 2} z^4 - \frac{120}{11} z^3 S_{3,\, 2,\, 2} \right.$$

$$\left. + \left(3\, T_{3,\, -2,\, 1} - \frac{39}{2} T_{3,\, -2,\, 2} \right) z^2 - \frac{9}{11} T_{3,\, -2,\, 1} + \frac{9}{4} T_{3,\, -2,\, 2} + \frac{2}{11} T_{3,\, -2,\, 0} \right) y$$

$$\left.\left. + \frac{90}{11} \left(T_{5,\, 1,\, 1} z^3 - \frac{22}{9} S_{5,\, -1,\, 1} z^2 + \left(-\frac{26}{45} T_{5,\, 1,\, 1} + \frac{4}{45} T_{5,\, 1,\, 0} \right) z + \frac{8}{5} S_{5,\, -1,\, 1} \right) z^2 \right) \right)$$

$$\sqrt{3} + \Big(\Big(\big(\big(4095\, T_{5,\, -3,\, 1} + 1768\, S_{4,\, 3,\, 2}\big) z + 1014\, T_{4,\, -3,\, 1} + 1794\, S_{5,\, 3,\, 1}\big)\, x^5$$

$$- 3042 \left(\left(\frac{105}{26} T_{5,\, 3,\, 1} - \frac{68}{39} S_{4,\, -3,\, 2} \right) z + T_{4,\, 3,\, 1} - \frac{23}{13} S_{5,\, -3,\, 1} \right) y\, x^4 + \Big(\Big(\Big($$

$$-8190\, T_{5,\, -3,\, 1} - 3536\, S_{4,\, 3,\, 2}\big) z - 2028\, T_{4,\, -3,\, 1} - 3588\, S_{5,\, 3,\, 1}\big) y^2 + \big(9360\, T_{5,\, -3,\, 1}$$

$$- 12376\, S_{4,\, 3,\, 2}\big) z^3 + \big(3042\, T_{4,\, -3,\, 1} + 1872\, S_{5,\, 3,\, 1}\big) z^2 + \big(-4056\, T_{5,\, -3,\, 1} + 624\, T_{5,\, -3,\, 0}$$

$$- 104\, S_{4,\, 3,\, 1} + 1456\, S_{4,\, 3,\, 2}\big) z - 858\, T_{4,\, -3,\, 1} + 156\, T_{4,\, -3,\, 0} - 1248\, S_{5,\, 3,\, 1}\big)\, x^3$$

$$- 2028\, y \left(\left(\left(\frac{105}{26} T_{5,\, 3,\, 1} - \frac{68}{39} S_{4,\, -3,\, 2} \right) z + T_{4,\, 3,\, 1} - \frac{23}{13} S_{5,\, -3,\, 1} \right) y^2 + \left(\frac{180}{13} T_{5,\, 3,\, 1} \right.\right.$$

$$\left. + \frac{238}{13} S_{4,\, -3,\, 2} \right) z^3 + \left(\frac{9}{2} T_{4,\, 3,\, 1} - \frac{36}{13} S_{5,\, -3,\, 1} \right) z^2 + \left(-6\, T_{5,\, 3,\, 1} + \frac{12}{13} T_{5,\, 3,\, 0} \right.$$

$$\left.\left. + \frac{2}{13} S_{4,\, -3,\, 1} - \frac{28}{13} S_{4,\, -3,\, 2} \right) z - \frac{33}{26} T_{4,\, 3,\, 1} + \frac{3}{13} T_{4,\, 3,\, 0} + \frac{24}{13} S_{5,\, -3,\, 1} \right) x^2$$

$$- 3042\, y^2 \left(\left(\left(\frac{105}{26} T_{5,\, -3,\, 1} + \frac{68}{39} S_{4,\, 3,\, 2} \right) z + T_{4,\, -3,\, 1} + \frac{23}{13} S_{5,\, 3,\, 1} \right) y^2 + \left(\frac{120}{13} T_{5,\, -3,\, 1} \right.\right.$$

$$\left. - \frac{476}{39} S_{4,\, 3,\, 2} \right) z^3 + \left(3\, T_{4,\, -3,\, 1} + \frac{24}{13} S_{5,\, 3,\, 1} \right) z^2 + \left(-4\, T_{5,\, -3,\, 1} + \frac{8}{13} T_{5,\, -3,\, 0} \right.$$

$$\left.\left. - \frac{4}{39} S_{4,\, 3,\, 1} + \frac{56}{39} S_{4,\, 3,\, 2} \right) z - \frac{11}{13} T_{4,\, -3,\, 1} + \frac{2}{13} T_{4,\, -3,\, 0} - \frac{16}{13} S_{5,\, 3,\, 1} \right) x$$

$$+ 1014\, y^3 \left(\left(\left(\frac{105}{26} T_{5,\, 3,\, 1} - \frac{68}{39} S_{4,\, -3,\, 2} \right) z + T_{4,\, 3,\, 1} - \frac{23}{13} S_{5,\, -3,\, 1} \right) y^2 + \left(\frac{120}{13} T_{5,\, 3,\, 1} \right.\right.$$

$$\left. + \frac{476}{39} S_{4,\, -3,\, 2} \right) z^3 + \left(3\, T_{4,\, 3,\, 1} - \frac{24}{13} S_{5,\, -3,\, 1} \right) z^2 + \left(-4\, T_{5,\, 3,\, 1} + \frac{8}{13} T_{5,\, 3,\, 0} \right.$$

$$\left.\left.\left. + \frac{4}{39} S_{4,\, -3,\, 1} - \frac{56}{39} S_{4,\, -3,\, 2} \right) z - \frac{11}{13} T_{4,\, 3,\, 1} + \frac{2}{13} T_{4,\, 3,\, 0} + \frac{16}{13} S_{5,\, -3,\, 1} \right) \right) \sqrt{7}$$

$$+ \big(\big(15210\, T_{3,\, -3,\, 2} + 90168\, S_{4,\, 1,\, 2}\big) z - 1014\, T_{4,\, -1,\, 1} - 4680\, S_{3,\, 3,\, 2}\big)\, x^5$$

$$-45630\,y\left(\left(T_{3,3,2}-\frac{1156}{585}\,S_{4,-1,2}\right)z-\frac{1}{45}\,T_{4,1,1}+\frac{4}{13}\,S_{3,-3,2}\right)x^{4}+\Big(\Big(\Big($$

$$-30420\,T_{3,-3,2}+180336\,S_{4,1,2}\big)z-2028\,T_{4,-1,1}+9360\,S_{3,3,2}\big)y^{2}+\big(15210\,T_{3,-3,2}$$

$$+305864\,S_{4,1,2}\big)z^{3}+\big(1014\,T_{4,-1,1}-14040\,S_{3,3,2}\big)z^{2}+\big(1716\,T_{3,-3,1}-11154\,T_{3,-3,2}$$

$$+9048\,S_{4,1,1}-126672\,S_{4,1,2}\big)z+858\,T_{4,-1,1}-156\,T_{4,-1,0}-312\,S_{3,3,1}$$

$$+3744\,S_{3,3,2}\big)x^{3}-5148\,y\left(\left(\left(\left(\frac{65}{11}\,T_{3,3,2}-\frac{1156}{33}\,S_{4,-1,2}\right)z-\frac{13}{33}\,T_{4,1,1}\right.\right.\right.$$

$$\left.+\frac{20}{11}\,S_{3,-3,2}\right)y^{2}+\left(\frac{195}{22}\,T_{3,3,2}-\frac{5882}{99}\,S_{4,-1,2}\right)z^{3}+\left(\frac{13}{66}\,T_{4,1,1}+\frac{90}{11}\,S_{3,-3,2}\right)z^{2}$$

$$+\left(T_{3,3,1}-\frac{13}{2}\,T_{3,3,2}-\frac{58}{33}\,S_{4,-1,1}+\frac{812}{33}\,S_{4,-1,2}\right)z+\frac{1}{6}\,T_{4,1,1}-\frac{1}{33}\,T_{4,1,0}$$

$$+\frac{2}{11}\,S_{3,-3,1}-\frac{24}{11}\,S_{3,-3,2}\Big)x^{2}+\Big(\Big(\big(-45630\,T_{3,-3,2}+90168\,S_{4,1,2}\big)z-1014\,T_{4,-1,1}$$

$$+14040\,S_{3,3,2}\big)y^{4}+\big(\big(-45630\,T_{3,-3,2}+305864\,S_{4,1,2}\big)z^{3}+\big(1014\,T_{4,-1,1}$$

$$+42120\,S_{3,3,2}\big)z^{2}+\big(-5148\,T_{3,-3,1}+33462\,T_{3,-3,2}+9048\,S_{4,1,1}-126672\,S_{4,1,2}\big)z$$

$$+858\,T_{4,-1,1}-156\,T_{4,-1,0}+936\,S_{3,3,1}-11232\,S_{3,3,2}\big)y^{2}+6760\left(\frac{1122}{65}\,z^{4}\,S_{4,1,2}\right.$$

$$+T_{4,-1,1}\,z^{3}+\left(\frac{8}{5}\,S_{4,1,1}-\frac{112}{5}\,S_{4,1,2}\right)z^{2}+\left(-\frac{33}{65}\,T_{4,-1,1}+\frac{6}{65}\,T_{4,-1,0}\right)z$$

$$\left.-\frac{12}{13}\,S_{4,1,1}+\frac{66}{13}\,S_{4,1,2}\right)z\Big)x+1716\left(\left(\left(\left(\frac{195}{22}\,T_{3,3,2}+\frac{578}{11}\,S_{4,-1,2}\right)z+\frac{13}{22}\,T_{4,1,1}\right.\right.\right.$$

$$\left.+\frac{30}{11}\,S_{3,-3,2}\right)y^{4}+\left(\left(\frac{195}{22}\,T_{3,3,2}+\frac{5882}{33}\,S_{4,-1,2}\right)z^{3}+\left(-\frac{13}{22}\,T_{4,1,1}\right.\right.$$

$$\left.+\frac{90}{11}\,S_{3,-3,2}\right)z^{2}+\left(T_{3,3,1}-\frac{13}{2}\,T_{3,3,2}+\frac{58}{11}\,S_{4,-1,1}-\frac{812}{11}\,S_{4,-1,2}\right)z-\frac{1}{2}\,T_{4,1,1}$$

$$+\frac{1}{11}\,T_{4,1,0}+\frac{2}{11}\,S_{3,-3,1}-\frac{24}{11}\,S_{3,-3,2}\Big)y^{2}-\frac{130}{33}\left(-\frac{1122}{65}\,S_{4,-1,2}\,z^{4}+T_{4,1,1}\,z^{3}\right.$$

$$+\left(-\frac{8}{5}\,S_{4,-1,1}+\frac{112}{5}\,S_{4,-1,2}\right)z^{2}+\left(-\frac{33}{65}\,T_{4,1,1}+\frac{6}{65}\,T_{4,1,0}\right)z+\frac{12}{13}\,S_{4,-1,1}$$

$$\left.\left.-\frac{66}{13}\,S_{4,-1,2}\right)z\right)y\Big)\sqrt{2}+\Big(\big(2340\,T_{5,-4,1}-3536\,S_{4,4,2}\big)x^{6}-9360\,y\Big(T_{5,4,1}$$

$$+\frac{68}{45}\,S_{4,-4,2}\Big)x^{5}+\big(\big(-11700\,T_{5,-4,1}+17680\,S_{4,4,2}\big)y^{2}+\big(7020\,T_{5,-4,1}$$

$$-10608\,S_{4,4,2}\big)z^{2}+\big(1352\,T_{4,-4,1}-312\,S_{5,4,1}\big)z-2028\,T_{5,-4,1}+312\,T_{5,-4,0}$$

$$-208\,S_{4,4,1}+2912\,S_{4,4,2}\big)x^{4}-5408\,y\left(\left(\frac{135}{26}\,T_{5,4,1}+\frac{102}{13}\,S_{4,-4,2}\right)z^{2}+\Big(T_{4,4,1}\right.$$

$$+\frac{3}{13}\,S_{5,-4,1}\Big)z-\frac{3}{2}\,T_{5,4,1}+\frac{3}{13}\,T_{5,4,0}+\frac{2}{13}\,S_{4,-4,1}-\frac{28}{13}\,S_{4,-4,2}\Big)x^{3}$$

$$- 8112 \left(\left(\frac{75}{52} T_{5, -4, 1} - \frac{85}{39} S_{4, 4, 2} \right) y^2 + \left(\frac{135}{26} T_{5, -4, 1} - \frac{102}{13} S_{4, 4, 2} \right) z^2 + (T_{4, -4, 1}$$

$$- \frac{3}{13} S_{5, 4, 1}) z - \frac{3}{2} T_{5, -4, 1} + \frac{3}{13} T_{5, -4, 0} - \frac{2}{13} S_{4, 4, 1} + \frac{28}{13} S_{4, 4, 2}) y^2 x^2$$

$$+ 5408 y^3 \left(\left(\frac{45}{26} T_{5, 4, 1} + \frac{34}{13} S_{4, -4, 2} \right) y^2 + \left(\frac{135}{26} T_{5, 4, 1} + \frac{102}{13} S_{4, -4, 2} \right) z^2 + (T_{4, 4, 1}$$

$$+ \frac{3}{13} S_{5, -4, 1}) z - \frac{3}{2} T_{5, 4, 1} + \frac{3}{13} T_{5, 4, 0} + \frac{2}{13} S_{4, -4, 1} - \frac{28}{13} S_{4, -4, 2}) x$$

$$+ 1352 y^4 \left(\left(\frac{45}{26} T_{5, -4, 1} - \frac{34}{13} S_{4, 4, 2} \right) y^2 + \left(\frac{135}{26} T_{5, -4, 1} - \frac{102}{13} S_{4, 4, 2} \right) z^2$$

$$+ \left(T_{4, -4, 1} - \frac{3}{13} S_{5, 4, 1} \right) z - \frac{3}{2} T_{5, -4, 1} + \frac{3}{13} T_{5, -4, 0} - \frac{2}{13} S_{4, 4, 1} + \frac{28}{13} S_{4, 4, 2}))$$

$$\sqrt{7} + 54808 x^6 S_{4, 2, 2} + 109616 x^5 y S_{4, -2, 2} + (54808 y^2 S_{4, 2, 2} + 180336 z^2 S_{4, 2, 2}$$

$$+ 6760 z T_{4, -2, 1} + 4784 S_{4, 2, 1} - 66976 S_{4, 2, 2}) x^4 - 13520 \left(-\frac{1054}{65} y^2 S_{4, -2, 2}$$

$$- \frac{1734}{65} z^2 S_{4, -2, 2} + T_{4, 2, 1} z - \frac{46}{65} S_{4, -2, 1} + \frac{644}{65} S_{4, -2, 2} \right) y x^3 + (-54808 y^4 S_{4, 2, 2}$$

$$+ 26520 S_{4, 2, 2} z^4 + 16224 T_{4, -2, 1} z^3 + (5616 S_{4, 2, 1} - 78624 S_{4, 2, 2}) z^2 + (-6864 T_{4, -2, 1}$$

$$+ 1248 T_{4, -2, 0}) z - 3120 S_{4, 2, 1} + 17160 S_{4, 2, 2}) x^2 - 13520 y \left(-\frac{527}{65} y^4 S_{4, -2, 2} + (\right.$$

$$-\frac{1734}{65} z^2 S_{4, -2, 2} + T_{4, 2, 1} z - \frac{46}{65} S_{4, -2, 1} + \frac{644}{65} S_{4, -2, 2}) y^2 - \frac{51}{13} S_{4, -2, 2} z^4$$

$$+ \frac{12}{5} T_{4, 2, 1} z^3 + \left(-\frac{54}{65} S_{4, -2, 1} + \frac{756}{65} S_{4, -2, 2} \right) z^2 + \left(-\frac{66}{65} T_{4, 2, 1} + \frac{12}{65} T_{4, 2, 0} \right) z$$

$$+ \frac{6}{13} S_{4, -2, 1} - \frac{33}{13} S_{4, -2, 2}) x - 6760 y^2 \left(\frac{527}{65} y^4 S_{4, 2, 2} + \left(\frac{1734}{65} z^2 S_{4, 2, 2} + T_{4, -2, 1} z \right. \right.$$

$$+ \frac{46}{65} S_{4, 2, 1} - \frac{644}{65} S_{4, 2, 2}) y^2 + \frac{51}{13} S_{4, 2, 2} z^4 + \frac{12}{5} T_{4, -2, 1} z^3 + (\frac{54}{65} S_{4, 2, 1}$$

$$- \frac{756}{65} S_{4, 2, 2}) z^2 + \left(-\frac{66}{65} T_{4, -2, 1} + \frac{12}{65} T_{4, -2, 0} \right) z - \frac{6}{13} S_{4, 2, 1} + \frac{33}{13} S_{4, 2, 2})) \sqrt{5}$$

$$+ \frac{1}{208} \left(\left((5070 z T_{3, -1, 2} + 29640 S_{3, 1, 2}) x^5 - 5070 y \left(T_{3, 1, 2} z - \frac{76}{13} S_{3, -1, 2} \right) x^4 \right. \right.$$

$$+ ((10140 z T_{3, -1, 2} + 59280 S_{3, 1, 2}) y^2 + 35490 T_{3, -1, 2} z^3 + 107640 z^2 S_{3, 1, 2}$$

$$+ (1716 T_{3, -1, 1} - 11154 T_{3, -1, 2}) z + 2808 S_{3, 1, 1} - 33696 S_{3, 1, 2}) x^3$$

$$- 1716 y \left(\left(\frac{65}{11} T_{3, 1, 2} z - \frac{380}{11} S_{3, -1, 2} \right) y^2 + \frac{455}{22} T_{3, 1, 2} z^3 - \frac{690}{11} z^2 S_{3, -1, 2} + (T_{3, 1, 1} \right.$$

$$- \frac{13}{2} T_{3, 1, 2}) z - \frac{18}{11} S_{3, -1, 1} + \frac{216}{11} S_{3, -1, 2}) x^2 + ((5070 z T_{3, -1, 2} + 29640 S_{3, 1, 2}) y^4$$

$$+ (35490 T_{3, -1, 2} z^3 + 107640 z^2 S_{3, 1, 2} + (1716 T_{3, -1, 1} - 11154 T_{3, -1, 2}) z + 2808 S_{3, 1, 1}$$

$$- 33696\, S_{3,\,1,\,2}) \, y^{2} + 30420\, T_{3,\,-1,\,2}\, z^{5} + 31200\, z^{4} S_{3,\,1,\,2} + (4576\, T_{3,\,-1,\,1}$$

$$- 29744\, T_{3,\,-1,\,2}) \, z^{3} + (3744\, S_{3,\,1,\,1} - 44928\, S_{3,\,1,\,2}) \, z^{2} + (-1872\, T_{3,\,-1,\,1} + 5148\, T_{3,\,-1,\,2}$$

$$+ 416\, T_{3,\,-1,\,0}) \, z - 1664\, S_{3,\,1,\,1} + 7488\, S_{3,\,1,\,2}) \, x - 1716\, y \, ((\frac{65}{22}\, T_{3,\,1,\,2}\, z$$

$$- \frac{190}{11}\, S_{3,\,-1,\,2}) \, y^{4} + (\frac{455}{22}\, T_{3,\,1,\,2}\, z^{3} - \frac{690}{11}\, z^{2} S_{3,\,-1,\,2} + (T_{3,\,1,\,1} - \frac{13}{2}\, T_{3,\,1,\,2}) \, z$$

$$- \frac{18}{11}\, S_{3,\,-1,\,1} + \frac{216}{11}\, S_{3,\,-1,\,2}) \, y^{2} + \frac{195}{11}\, T_{3,\,1,\,2}\, z^{5} - \frac{200}{11}\, z^{4} S_{3,\,-1,\,2} + (\frac{8}{3}\, T_{3,\,1,\,1}$$

$$- \frac{52}{3}\, T_{3,\,1,\,2}) \, z^{3} + (-\frac{24}{11}\, S_{3,\,-1,\,1} + \frac{288}{11}\, S_{3,\,-1,\,2}) \, z^{2} + (-\frac{12}{11}\, T_{3,\,1,\,1} + 3\, T_{3,\,1,\,2}$$

$$+ \frac{8}{33}\, T_{3,\,1,\,0}) \, z + \frac{32}{33}\, S_{3,\,-1,\,1} - \frac{48}{11}\, S_{3,\,-1,\,2})) \sqrt{2} + ((-28860\, z\, S_{6,\,1,\,1}$$

$$+ 130\, T_{6,\,-1,\,0}) \, x^{5} - 28860\, (S_{6,\,-1,\,1}\, z + \frac{1}{222}\, T_{6,\,1,\,0}) \, y\, x^{4} + ((-57720\, z\, S_{6,\,1,\,1}$$

$$+ 260\, T_{6,\,-1,\,0}) \, y^{2} + 6240\, (S_{6,\,1,\,1}\, z^{2} - \frac{1}{4}\, z\, T_{6,\,-1,\,0} + \frac{7}{2}\, S_{6,\,1,\,1}) \, z) \, x^{3}$$

$$- 57720\, ((S_{6,\,-1,\,1}\, z + \frac{1}{222}\, T_{6,\,1,\,0}) \, y^{2} - \frac{4}{37}\, (S_{6,\,-1,\,1}\, z^{2} + \frac{1}{4}\, z\, T_{6,\,1,\,0}$$

$$+ \frac{7}{2}\, S_{6,\,-1,\,1}) \, z) \, y\, x^{2} + ((-28860\, z\, S_{6,\,1,\,1} + 130\, T_{6,\,-1,\,0}) \, y^{4} + 6240\, (S_{6,\,1,\,1}\, z^{2}$$

$$- \frac{1}{4}\, z\, T_{6,\,-1,\,0} + \frac{7}{2}\, S_{6,\,1,\,1}) \, z\, y^{2} + 41184\, S_{6,\,1,\,1}\, z^{5} + 1040\, z^{4} T_{6,\,-1,\,0} - 29120\, S_{6,\,1,\,1}\, z^{3}) \, x$$

$$- 28860\, y\, ((S_{6,\,-1,\,1}\, z + \frac{1}{222}\, T_{6,\,1,\,0}) \, y^{4} - \frac{8}{37}\, (S_{6,\,-1,\,1}\, z^{2} + \frac{1}{4}\, z\, T_{6,\,1,\,0}$$

$$+ \frac{7}{2}\, S_{6,\,-1,\,1}) \, z\, y^{2} - \frac{264}{185}\, (S_{6,\,-1,\,1}\, z^{2} - \frac{5}{198}\, z\, T_{6,\,1,\,0} - \frac{70}{99}\, S_{6,\,-1,\,1}) \, z^{3})) \sqrt{7}$$

$$- 39780\, x^{6} S_{2,\,2,\,3} + (-79560\, y\, S_{2,\,-2,\,3} + 198900\, z\, S_{2,\,1,\,3} + 3718\, T_{2,\,-1,\,2}) \, x^{5} + ($$

$$-39780\, y^{2} S_{2,\,2,\,3} + (198900\, z\, S_{2,\,-1,\,3} - 3718\, T_{2,\,1,\,2}) \, y - 238680\, z^{2} S_{2,\,2,\,3}$$

$$+ 14872\, z\, T_{2,\,-2,\,2} - 5408\, S_{2,\,2,\,2} + 54600\, S_{2,\,2,\,3}) \, x^{4} + (-159120\, y^{3} S_{2,\,-2,\,3}$$

$$+ (397800\, z\, S_{2,\,1,\,3} + 7436\, T_{2,\,-1,\,2}) \, y^{2} + (-477360\, z^{2} S_{2,\,-2,\,3} - 29744\, z\, T_{2,\,2,\,2}$$

$$- 10816\, S_{2,\,-2,\,2} + 109200\, S_{2,\,-2,\,3}) \, y + 79560\, z^{3} S_{2,\,1,\,3} + 22308\, z^{2} T_{2,\,-1,\,2}$$

$$+ (16224\, S_{2,\,1,\,2} - 163800\, S_{2,\,1,\,3}) \, z + 936\, T_{2,\,-1,\,1} - 5148\, T_{2,\,-1,\,2}) \, x^{3} + (39780\, y^{4} S_{2,\,2,\,3}$$

$$+ (397800\, z\, S_{2,\,-1,\,3} - 7436\, T_{2,\,1,\,2}) \, y^{3} + (79560\, z^{3} S_{2,\,-1,\,3} - 22308\, T_{2,\,1,\,2}\, z^{2}$$

$$+ (16224\, S_{2,\,-1,\,2} - 163800\, S_{2,\,-1,\,3}) \, z - 936\, T_{2,\,1,\,1} + 5148\, T_{2,\,1,\,2}) \, y - 198900\, z^{4} S_{2,\,2,\,3}$$

$$+ 14872 z^3 T_{2, -2, 2} + (-16224 S_{2, 2, 2} + 163800 S_{2, 2, 3}) z^2 + (1872 T_{2, -2, 1}$$

$$- 10296 T_{2, -2, 2}) z - 416 S_{2, 2, 1} + 4160 S_{2, 2, 2} - 17316 S_{2, 2, 3}) x^2 + (-79560 y^5 S_{2, -2, 3}$$

$$+ (198900 z S_{2, 1, 3} + 3718 T_{2, -1, 2}) y^4 + (-477360 z^2 S_{2, -2, 3} - 29744 z T_{2, 2, 2}$$

$$- 10816 S_{2, -2, 2} + 109200 S_{2, -2, 3}) y^3 + (79560 z^3 S_{2, 1, 3} + 22308 z^2 T_{2, -1, 2}$$

$$+ (16224 S_{2, 1, 2} - 163800 S_{2, 1, 3}) z + 936 T_{2, -1, 1} - 5148 T_{2, -1, 2}) y^2 + ($$

$$-397800 S_{2, -2, 3} z^4 - 29744 T_{2, 2, 2} z^3 + (-32448 S_{2, -2, 2} + 327600 S_{2, -2, 3}) z^2 + ($$

$$-3744 T_{2, 2, 1} + 20592 T_{2, 2, 2}) z - 832 S_{2, -2, 1} + 8320 S_{2, -2, 2} - 34632 S_{2, -2, 3}) y$$

$$- 119340 z^5 S_{2, 1, 3} + 18590 z^4 T_{2, -1, 2} + (-5408 S_{2, 1, 2} + 54600 S_{2, 1, 3}) z^3$$

$$+ (2808 T_{2, -1, 1} - 15444 T_{2, -1, 2}) z^2 + (416 S_{2, 1, 1} - 4160 S_{2, 1, 2} + 17316 S_{2, 1, 3}) z$$

$$- 728 T_{2, -1, 1} + 1638 T_{2, -1, 2} + 208 T_{2, -1, 0}) x - 1872 y \left(-\frac{85}{4} y^5 S_{2, 2, 3} + \left(\frac{143}{72} T_{2, 1, 2}\right.\right.$$

$$\left.- \frac{425}{4} z S_{2, -1, 3}\right) y^4 + \left(-\frac{26}{9} S_{2, 2, 2} + \frac{143}{18} z T_{2, -2, 2} + \frac{175}{6} S_{2, 2, 3}\right.$$

$$\left.- \frac{255}{2} z^2 S_{2, 2, 3}\right) y^3 + \left(-\frac{85}{2} z^3 S_{2, -1, 3} + \frac{143}{12} T_{2, 1, 2} z^2 + \left(-\frac{26}{3} S_{2, -1, 2}\right.\right.$$

$$\left.\left.+ \frac{175}{2} S_{2, -1, 3}\right) z + \frac{1}{2} T_{2, 1, 1} - \frac{11}{4} T_{2, 1, 2}\right) y^2 + \left(-\frac{425}{4} z^4 S_{2, 2, 3} + \frac{143}{18} z^3 T_{2, -2, 2}\right.$$

$$+ \left(-\frac{26}{3} S_{2, 2, 2} + \frac{175}{2} S_{2, 2, 3}\right) z^2 + \left(T_{2, -2, 1} - \frac{11}{2} T_{2, -2, 2}\right) z - \frac{2}{9} S_{2, 2, 1} + \frac{20}{9} S_{2, 2, 2}$$

$$\left.- \frac{37}{4} S_{2, 2, 3}\right) y + \frac{255}{4} z^5 S_{2, -1, 3} + \frac{715}{72} T_{2, 1, 2} z^4 + \left(\frac{26}{9} S_{2, -1, 2} - \frac{175}{6} S_{2, -1, 3}\right) z^3$$

$$+ \left(\frac{3}{2} T_{2, 1, 1} - \frac{33}{4} T_{2, 1, 2}\right) z^2 + \left(\frac{20}{9} S_{2, -1, 2} - \frac{37}{4} S_{2, -1, 3} - \frac{2}{9} S_{2, -1, 1}\right) z$$

$$\left.\left.+ \frac{1}{9} T_{2, 1, 0} + \frac{7}{8} T_{2, 1, 2} - \frac{7}{18} T_{2, 1, 1}\right)\right) \sqrt{3} + \frac{1}{208} \left(7488 x^6 S_{6, 4, 1} + 29952 x^5 y S_{6, -4, 1}\right.$$

$$+ (-37440 y^2 S_{6, 4, 1} + 7020 z^2 S_{6, 4, 1} + 3120 z T_{6, -4, 0} - 5460 S_{6, 4, 1}) x^4$$

$$+ 28080 \left(S_{6, -4, 1} z^2 - \frac{4}{9} z T_{6, 4, 0} - \frac{7}{9} S_{6, -4, 1}\right) y x^3 - 37440 \left(S_{6, 4, 1} y^2 + \frac{9}{8} S_{6, 4, 1} z^2\right.$$

$$\left.+ \frac{1}{2} z T_{6, -4, 0} - \frac{7}{8} S_{6, 4, 1}\right) y^2 x^2 - 29952 y^3 \left(S_{6, -4, 1} y^2 + \frac{15}{16} S_{6, -4, 1} z^2 - \frac{5}{12} z T_{6, 4, 0}\right.$$

$$\left.- \frac{35}{48} S_{6, -4, 1}\right) x + 7488 y^4 \left(S_{6, 4, 1} y^2 + \frac{15}{16} S_{6, 4, 1} z^2 + \frac{5}{12} z T_{6, -4, 0} - \frac{35}{48} S_{6, 4, 1}\right)\Bigg)$$

$$\sqrt{7} + \frac{1}{208} (-106080 S_{4, 0, 2} + 159120 S_{2, 0, 3} + 21840 S_{6, 0, 1}) x^6$$

$$+\frac{1}{208}\left(55770\,z\,T_{1,\,-1,\,3}-30420\,S_{1,\,1,\,3}\right)x^{5}+\frac{1}{208}\left(\left(477360\,S_{2,\,0,\,3}-318240\,S_{4,\,0,\,2}\right.\right.$$

$$\left.+65520\,S_{6,\,0,\,1}\right)y^{2}+\left(-55770\,z\,T_{1,\,1,\,3}-30420\,S_{1,\,-1,\,3}\right)y+\left(1074060\,S_{2,\,0,\,3}\right.$$

$$\left.-159120\,S_{4,\,0,\,2}-147420\,S_{6,\,0,\,1}\right)z^{2}+\left(182520\,S_{1,\,0,\,3}+56160\,S_{3,\,0,\,2}-51480\,S_{5,\,0,\,1}\right)z$$

$$\left.+24336\,S_{2,\,0,\,2}-245700\,S_{2,\,0,\,3}-9360\,S_{4,\,0,\,1}+131040\,S_{4,\,0,\,2}-16380\,S_{6,\,0,\,1}\right)x^{4}$$

$$+\frac{1}{208}\left(\left(111540\,z\,T_{1,\,-1,\,3}-60840\,S_{1,\,1,\,3}\right)y^{2}-31500\,y+111540\,z^{3}\,T_{1,\,-1,\,3}\right.$$

$$\left.-182520\,z^{2}\,S_{1,\,1,\,3}+\left(10296\,T_{1,\,-1,\,2}-66924\,T_{1,\,-1,\,3}\right)z-4576\,S_{1,\,1,\,2}+39208\,S_{1,\,1,\,3}\right)x^{3}$$

$$+\frac{1}{208}\left(\left(477360\,S_{2,\,0,\,3}-318240\,S_{4,\,0,\,2}+65520\,S_{6,\,0,\,1}\right)y^{4}+\left(-111540\,z\,T_{1,\,1,\,3}\right.\right.$$

$$\left.-60840\,S_{1,\,-1,\,3}\right)y^{3}+\left(\left(2148120\,S_{2,\,0,\,3}-318240\,S_{4,\,0,\,2}-294840\,S_{6,\,0,\,1}\right)z^{2}\right.$$

$$+\left(365040\,S_{1,\,0,\,3}+112320\,S_{3,\,0,\,2}-102960\,S_{5,\,0,\,1}\right)z+48672\,S_{2,\,0,\,2}-491400\,S_{2,\,0,\,3}$$

$$\left.-18720\,S_{4,\,0,\,1}+262080\,S_{4,\,0,\,2}-32760\,S_{6,\,0,\,1}\right)y^{2}+\left(-111540\,z^{3}\,T_{1,\,1,\,3}\right.$$

$$-182520\,z^{2}\,S_{1,\,-1,\,3}+\left(-10296\,T_{1,\,1,\,2}+66924\,T_{1,\,1,\,3}\right)z-4576\,S_{1,\,-1,\,2}$$

$$\left.+39208\,S_{1,\,-1,\,3}\right)y+\left(1193400\,S_{2,\,0,\,3}+442000\,S_{4,\,0,\,2}-131040\,S_{6,\,0,\,1}\right)z^{4}$$

$$+\left(243360\,S_{1,\,0,\,3}+262080\,S_{3,\,0,\,2}-24960\,S_{5,\,0,\,1}\right)z^{3}+\left(97344\,S_{2,\,0,\,2}-982800\,S_{2,\,0,\,3}\right.$$

$$\left.+6240\,S_{4,\,0,\,1}-87360\,S_{4,\,0,\,2}+131040\,S_{6,\,0,\,1}\right)z^{2}+\left(-156832\,S_{1,\,0,\,3}+7488\,S_{3,\,0,\,1}\right.$$

$$\left.-89856\,S_{3,\,0,\,2}+37440\,S_{5,\,0,\,1}+18304\,S_{1,\,0,\,2}\right)z+2496\,S_{2,\,0,\,1}-24960\,S_{2,\,0,\,2}$$

$$\left.+103896\,S_{2,\,0,\,3}+6240\,S_{4,\,0,\,1}-34320\,S_{4,\,0,\,2}\right)x^{2}+\frac{1}{208}\left(\left(55770\,z\,T_{1,\,-1,\,3}\right.\right.$$

$$\left.-30420\,S_{1,\,1,\,3}\right)y^{4}+31500\,y^{3}+\left(111540\,z^{3}\,T_{1,\,-1,\,3}-182520\,z^{2}\,S_{1,\,1,\,3}+\left(10296\,T_{1,\,-1,\,2}\right.\right.$$

$$\left.\left.-66924\,T_{1,\,-1,\,3}\right)z-4576\,S_{1,\,1,\,2}+39208\,S_{1,\,1,\,3}\right)y^{2}+55770\,z^{5}\,T_{1,\,-1,\,3}$$

$$-152100\,z^{4}\,S_{1,\,1,\,3}+\left(10296\,T_{1,\,-1,\,2}-66924\,T_{1,\,-1,\,3}\right)z^{3}+\left(-13728\,S_{1,\,1,\,2}\right.$$

$$\left.+117624\,S_{1,\,1,\,3}\right)z^{2}+\left(1456\,T_{1,\,-1,\,1}-6552\,T_{1,\,-1,\,2}+18018\,T_{1,\,-1,\,3}\right)z-416\,S_{1,\,1,\,1}$$

$$\left.+3328\,S_{1,\,1,\,2}-11284\,S_{1,\,1,\,3}\right)x+\frac{1}{208}\left(-106080\,S_{4,\,0,\,2}+159120\,S_{2,\,0,\,3}\right.$$

$$\left.+21840\,S_{6,\,0,\,1}\right)y^{6}+\frac{1}{208}\left(-55770\,z\,T_{1,\,1,\,3}-30420\,S_{1,\,-1,\,3}\right)y^{5}$$

$$+\frac{1}{208}\left(\left(1074060\,S_{2,0,3}-159120\,S_{4,0,2}-147420\,S_{6,0,1}\right)z^2+\left(182520\,S_{1,0,3}\right.\right.$$

$$+56160\,S_{3,0,2}-51480\,S_{5,0,1}\big)\,z+24336\,S_{2,0,2}-245700\,S_{2,0,3}-9360\,S_{4,0,1}$$

$$+131040\,S_{4,0,2}-16380\,S_{6,0,1}\big)\,y^4+\frac{1}{208}\left(-111540\,z^3\,T_{1,1,3}-182520\,z^2\,S_{1,-1,3}+\left(\right.\right.$$

$$-10296\,T_{1,1,2}+66924\,T_{1,1,3}\big)\,z-4576\,S_{1,-1,2}+39208\,S_{1,-1,3}\big)\,y^3$$

$$+\frac{1}{208}\left(\left(1193400\,S_{2,0,3}+442000\,S_{4,0,2}-131040\,S_{6,0,1}\right)z^4+\left(243360\,S_{1,0,3}\right.\right.$$

$$+262080\,S_{3,0,2}-24960\,S_{5,0,1}\big)\,z^3+\big(97344\,S_{2,0,2}-982800\,S_{2,0,3}+6240\,S_{4,0,1}$$

$$-87360\,S_{4,0,2}+131040\,S_{6,0,1}\big)\,z^2+\big(-156832\,S_{1,0,3}+7488\,S_{3,0,1}-89856\,S_{3,0,2}$$

$$+37440\,S_{5,0,1}+18304\,S_{1,0,2}\big)\,z+2496\,S_{2,0,1}-24960\,S_{2,0,2}+103896\,S_{2,0,3}$$

$$+6240\,S_{4,0,1}-34320\,S_{4,0,2}\big)\,y^2+\frac{1}{208}\left(-55770\,z^5\,T_{1,1,3}-152100\,z^4\,S_{1,-1,3}+\left(\right.\right.$$

$$-10296\,T_{1,1,2}+66924\,T_{1,1,3}\big)\,z^3+\big(-13728\,S_{1,-1,2}+117624\,S_{1,-1,3}\big)\,z^2+\big($$

$$-1456\,T_{1,1,1}+6552\,T_{1,1,2}-18018\,T_{1,1,3}\big)\,z-416\,S_{1,-1,1}+3328\,S_{1,-1,2}$$

$$-11284\,S_{1,-1,3}\big)\,y+\frac{1}{208}\left(278460\,S_{2,0,3}+247520\,S_{4,0,2}+61152\,S_{6,0,1}\right)z^6$$

$$+\frac{1}{208}\left(60840\,S_{1,0,3}+112320\,S_{3,0,2}+37440\,S_{5,0,1}\right)z^5+\frac{1}{208}\left(40560\,S_{2,0,2}\right.$$

$$-409500\,S_{2,0,3}+20800\,S_{4,0,1}-291200\,S_{4,0,2}-43680\,S_{6,0,1}\big)\,z^4+\frac{1}{208}\,\big($$

$$-78416\,S_{1,0,3}+9984\,S_{3,0,1}-119808\,S_{3,0,2}-24960\,S_{5,0,1}+9152\,S_{1,0,2}\big)\,z^3$$

$$+\frac{1}{208}\left(3744\,S_{2,0,1}-37440\,S_{2,0,2}+155844\,S_{2,0,3}-12480\,S_{4,0,1}+68640\,S_{4,0,2}\right)z^2$$

$$+\frac{1}{208}\left(22568\,S_{1,0,3}-4992\,S_{3,0,1}+22464\,S_{3,0,2}+832\,S_{1,0,1}-6656\,S_{1,0,2}\right)z$$

$$-6\,S_{2,0,1}+21\,S_{2,0,2}-\frac{189}{4}\,S_{2,0,3}\Big)\,\bar{e}_z$$

```
> constraints := `union`(seq({coeffs(collect(eqn[i], [x,y,z],
  distributed), [x,y,z])}, i=1..3)):
> nops(constraints); nops(variables);
```

$$252$$

$$167 \tag{31}$$

```
> sol1 := solve(constraints, variables);
```

$$sol1 := \left\{S_{1,-1,1}=0,\ S_{1,-1,2}=0,\ S_{1,-1,3}=0,\ S_{1,0,1}=0,\ S_{1,0,2}=0,\ S_{1,0,3}=0,\ S_{1,1,1}=0,\ S_{1,1,2}\right. \tag{32}$$

$= 0, S_{1,\,1,\,3} = 0, S_{2,\,-2,\,1} = 0, S_{2,\,-2,\,2} = 0, S_{2,\,-2,\,3} = 0, S_{2,\,-1,\,1} = 0, S_{2,\,-1,\,2} = 0, S_{2,\,-1,\,3} = 0,$

$S_{2,\,0,\,1} = 0, S_{2,\,0,\,2} = 0, S_{2,\,0,\,3} = 0, S_{2,\,1,\,1} = 0, S_{2,\,1,\,2} = 0, S_{2,\,1,\,3} = 0, S_{2,\,2,\,1} = 0, S_{2,\,2,\,2} = 0, S_{2,\,2,\,3}$

$= 0, S_{3,\,-3,\,1} = 0, S_{3,\,-3,\,2} = 0, S_{3,\,-2,\,1} = 0, S_{3,\,-2,\,2} = 0, S_{3,\,-1,\,1} = 0, S_{3,\,-1,\,2} = 0, S_{3,\,0,\,1} = 0,$

$S_{3,\,0,\,2} = 0, S_{3,\,1,\,1} = 0, S_{3,\,1,\,2} = 0, S_{3,\,2,\,1} = 0, S_{3,\,2,\,2} = 0, S_{3,\,3,\,1} = 0, S_{3,\,3,\,2} = 0, S_{4,\,-4,\,1}$

$= \frac{45}{208} \sqrt{7} \sqrt{5}, S_{4,\,-4,\,2} = 0, S_{4,\,-3,\,1} = 0, S_{4,\,-3,\,2} = 0, S_{4,\,-2,\,1} = 0, S_{4,\,-2,\,2} = 0, S_{4,\,-1,\,1} = 0,$

$S_{4,\,-1,\,2} = 0, S_{4,\,0,\,1} = 0, S_{4,\,0,\,2} = 0, S_{4,\,1,\,1} = 0, S_{4,\,1,\,2} = 0, S_{4,\,2,\,1} = 0, S_{4,\,2,\,2} = 0, S_{4,\,3,\,1} = 0,$

$S_{4,\,3,\,2} = 0, S_{4,\,4,\,1} = 0, S_{4,\,4,\,2} = 0, S_{5,\,-4,\,1} = 0, S_{5,\,-3,\,1} = 0, S_{5,\,-2,\,1} = 0, S_{5,\,-1,\,1} = 0, S_{5,\,0,\,1} = 0,$

$S_{5,\,1,\,1} = 0, S_{5,\,2,\,1} = 0, S_{5,\,3,\,1} = 0, S_{5,\,4,\,1} = 0, S_{6,\,-4,\,1} = 0, S_{6,\,-3,\,1} = 0, S_{6,\,-2,\,1} = 0, S_{6,\,-1,\,1} = 0,$

$S_{6,\,0,\,1} = 0, S_{6,\,1,\,1} = 0, S_{6,\,2,\,1} = 0, S_{6,\,3,\,1} = 0, S_{6,\,4,\,1} = 0, T_{1,\,-1,\,0} = 0, T_{1,\,-1,\,1} = 0, T_{1,\,-1,\,2} = 0,$

$T_{1,\,-1,\,3} = 0, T_{1,\,0,\,0} = T_{1,\,0,\,0}, T_{1,\,0,\,1} = \frac{675}{52} - \frac{12}{7} T_{3,\,0,\,0}, T_{1,\,0,\,2} = -\frac{3675}{572} + \frac{24}{11} T_{5,\,0,\,0},$

$T_{1,\,0,\,3} = 0, T_{1,\,1,\,0} = 0, T_{1,\,1,\,1} = 0, T_{1,\,1,\,2} = 0, T_{1,\,1,\,3} = 0, T_{2,\,-2,\,0} = 0, T_{2,\,-2,\,1} = 0, T_{2,\,-2,\,2} = 0,$

$T_{2,\,-1,\,0} = 0, T_{2,\,-1,\,1} = 0, T_{2,\,-1,\,2} = 0, T_{2,\,0,\,0} = 0, T_{2,\,0,\,1} = 0, T_{2,\,0,\,2} = 0, T_{2,\,1,\,0} = 0, T_{2,\,1,\,1} = 0,$

$T_{2,\,1,\,2} = 0, T_{2,\,2,\,0} = 0, T_{2,\,2,\,1} = 0, T_{2,\,2,\,2} = 0, T_{3,\,-3,\,0} = 0, T_{3,\,-3,\,1} = 0, T_{3,\,-3,\,2} = 0, T_{3,\,-2,\,0} = 0,$

$T_{3,\,-2,\,1} = 0, T_{3,\,-2,\,2} = 0, T_{3,\,-1,\,0} = 0, T_{3,\,-1,\,1} = 0, T_{3,\,-1,\,2} = 0, T_{3,\,0,\,0} = T_{3,\,0,\,0}, T_{3,\,0,\,1} = -\frac{525}{1144}$

$- \frac{14}{11} T_{5,\,0,\,0}, T_{3,\,0,\,2} = 0, T_{3,\,1,\,0} = 0, T_{3,\,1,\,1} = 0, T_{3,\,1,\,2} = 0, T_{3,\,2,\,0} = 0, T_{3,\,2,\,1} = 0, T_{3,\,2,\,2} = 0,$

$T_{3,\,3,\,0} = 0, T_{3,\,3,\,1} = 0, T_{3,\,3,\,2} = 0, T_{4,\,-4,\,0} = 0, T_{4,\,-4,\,1} = 0, T_{4,\,-3,\,0} = 0, T_{4,\,-3,\,1} = 0, T_{4,\,-2,\,0}$

$= 0, T_{4,\,-2,\,1} = 0, T_{4,\,-1,\,0} = 0, T_{4,\,-1,\,1} = 0, T_{4,\,0,\,0} = 0, T_{4,\,0,\,1} = 0, T_{4,\,1,\,0} = 0, T_{4,\,1,\,1} = 0, T_{4,\,2,\,0}$

$= 0, T_{4,\,2,\,1} = 0, T_{4,\,3,\,0} = 0, T_{4,\,3,\,1} = 0, T_{4,\,4,\,0} = 0, T_{4,\,4,\,1} = 0, T_{5,\,-4,\,0} = 0, T_{5,\,-4,\,1} = 0, T_{5,\,-3,\,0}$

$= 0, T_{5,\,-3,\,1} = 0, T_{5,\,-2,\,0} = 0, T_{5,\,-2,\,1} = 0, T_{5,\,-1,\,0} = 0, T_{5,\,-1,\,1} = 0, T_{5,\,0,\,0} = T_{5,\,0,\,0}, T_{5,\,0,\,1} = 0,$

$T_{5,\,1,\,0} = 0, T_{5,\,1,\,1} = 0, T_{5,\,2,\,0} = 0, T_{5,\,2,\,1} = 0, T_{5,\,3,\,0} = 0, T_{5,\,3,\,1} = 0, T_{5,\,4,\,0} = -\frac{45}{52} \sqrt{7} \sqrt{5},$

$T_{5,\,4,\,1} = 0, T_{6,\,-4,\,0} = 0, T_{6,\,-3,\,0} = 0, T_{6,\,-2,\,0} = 0, T_{6,\,-1,\,0} = 0, T_{6,\,0,\,0} = 0, T_{6,\,1,\,0} = 0, T_{6,\,2,\,0} = 0,$

$T_{6,\,3,\,0} = 0, T_{6,\,4,\,0} = 0 \}$

> # set geostrophic component to zero to remove degeneracy

```
> u_soln := simplify(subs(sol1, u_cart));
```

$$u\_soln := -\frac{315}{8}\left(\left(T_{5,0,0}-\frac{75}{26}\right)y^4+\left(2x^2T_{5,0,0}-\frac{50}{13}z^2-\frac{4}{21}T_{3,0,0}-\frac{12}{11}T_{5,0,0}+\frac{590}{143}\right)y^2+\left(T_{5,0,0}-\frac{25}{26}\right)x^4+\left(-\frac{75}{13}z^2-\frac{4}{21}T_{3,0,0}-\frac{12}{11}T_{5,0,0}+\frac{315}{143}\right)x^2-\frac{125}{52}z^4+\frac{105}{26}z^2+\frac{8}{315}T_{1,0,0}+\frac{16}{147}T_{3,0,0}+\frac{8}{33}T_{5,0,0}-\frac{18475}{12012}\right)y\bar{e}_x+\frac{315}{8}\left(\left(T_{5,0,0}-\frac{75}{26}\right)x^4+\left(2y^2T_{5,0,0}-\frac{50}{13}z^2-\frac{4}{21}T_{3,0,0}-\frac{12}{11}T_{5,0,0}+\frac{590}{143}\right)x^2+\left(T_{5,0,0}-\frac{25}{26}\right)y^4+\left(-\frac{75}{13}z^2-\frac{4}{21}T_{3,0,0}-\frac{12}{11}T_{5,0,0}+\frac{315}{143}\right)y^2-\frac{125}{52}z^4+\frac{105}{26}z^2+\frac{8}{315}T_{1,0,0}+\frac{16}{147}T_{3,0,0}+\frac{8}{33}T_{5,0,0}-\frac{18475}{12012}\right)x\bar{e}_y-\frac{7875}{52}xyz\left(x^2-y^2\right)\bar{e}_z \quad (33)$$

```
> u_cyl := simplify(MapToBasis(u_soln, cylindrical[s,phi,z]));
```

$$u\_cyl := -\frac{7875}{52}\left(\cos(\phi)^2-\frac{1}{2}\right)\sin(\phi)\left(s^2-z^2-1\right)\cos(\phi)\,s^3\bar{e}_s+\frac{315}{8}\left(-\frac{100}{13}\left(s^2-\frac{1}{2}z^2-\frac{1}{2}\right)s^2\cos(\phi)^4+\frac{100}{13}\left(s^2-\frac{1}{2}z^2-\frac{1}{2}\right)s^2\cos(\phi)^2+\left(T_{5,0,0}-\frac{75}{26}\right)s^4+\left(-\frac{50}{13}z^2-\frac{4}{21}T_{3,0,0}-\frac{12}{11}T_{5,0,0}+\frac{590}{143}\right)s^2-\frac{125}{52}z^4+\frac{105}{26}z^2+\frac{8}{315}T_{1,0,0}+\frac{16}{147}T_{3,0,0}+\frac{8}{33}T_{5,0,0}-\frac{18475}{12012}\right)s\bar{e}_\phi-\frac{7875}{52}s^4z\left(2\cos(\phi)^2-1\right)\cos(\phi)\sin(\phi)\bar{e}_z \quad (34)$$

> $SetCoordinates(cylindrical[s, \text{phi}, z])$

$$\text{cylindrical}_{s,\phi,z} \quad (35)$$

```
> geostrophic_component := simplify(int( int( u_cyl[2], phi=0..2*Pi),z=-sqrt(1-s^2)..sqrt(1-s^2))/(4*Pi*sqrt(1-s^2)));
```

$$geostrophic\_component := \frac{315}{8}\left(\left(T_{5,0,0}-\frac{25}{26}\right)s^4+\left(-\frac{4}{21}T_{3,0,0}-\frac{12}{11}T_{5,0,0}+\frac{20}{11}\right)s^2+\frac{8}{315}T_{1,0,0}+\frac{16}{147}T_{3,0,0}+\frac{8}{33}T_{5,0,0}-\frac{2020}{3003}\right)s \quad (36)$$

```
> u_cyl := simplify(u_cyl - VectorField([0, geostrophic_component, 0])); #remove geostrophic component from u_cyl
```

$$u\_cyl := -\frac{7875}{52}\left(\cos(\phi)^2-\frac{1}{2}\right)\sin(\phi)\left(s^2-z^2-1\right)\cos(\phi)\,s^3\bar{e}_s-\frac{7875}{26}\left(\left(s^2-\frac{1}{2}z^2\right.\right. \quad (37)$$

$$-\frac{1}{2}\Big) s^2\cos(\phi)^4 - \Big(s^2 - \frac{1}{2}z^2 - \frac{1}{2}\Big) s^2\cos(\phi)^2 + \frac{1}{4}s^4 + \Big(\frac{1}{2}z^2 - \frac{3}{10}\Big)s^2 + \frac{5}{16}z^4$$
$$-\frac{21}{40}z^2 + \frac{9}{80}\Big) s\bar{e}_\phi - \frac{7875}{52}s^4 z\left(2\cos(\phi)^2 - 1\right)\cos(\phi)\sin(\phi)\bar{e}_z$$

```
> simplify(int( u_cyl[2],z=-sqrt(1-s^2)..sqrt(1-s^2))); #check that no geostrophic component remains
```

$$-\frac{2625}{208}s^3\sqrt{-s^2+1}\,\left(7s^2-4\right)\left(8\cos(\phi)^4 - 8\cos(\phi)^2 + 1\right) \quad \textbf{(38)}$$

```
> map(factor, simplify(MapToBasis(u_soln, spherical[r,theta,phi])));
```

$$-\frac{7875}{104}\sin(\theta)^4\sin(\phi)\cos(\phi)\,(r-1)\,(r+1)\left(2\cos(\phi)^2-1\right)r^3\bar{e}_r \quad \textbf{(39)}$$
$$+\frac{7875}{104}\sin(\theta)^3\sin(\phi)\cos(\phi)\cos(\theta)\left(r^2+1\right)\left(2\cos(\phi)^2-1\right)r^3\bar{e}_\theta$$
$$-\frac{1}{32032}r\sin(\theta)\,\big(1939875 + 4851000\cos(\phi)^2r^2 - 1261260r^4T_{5,0,0}$$
$$-14553000\cos(\phi)^2r^4\cos(\theta)^4 + 110250r^2\cos(\theta)^2 + 1819125r^4\cos(\theta)^4$$
$$+14553000\cos(\theta)^4\cos(\phi)^4r^4 - 24255000\cos(\phi)^4r^4\cos(\theta)^2$$
$$-1261260\cos(\theta)^4r^4T_{5,0,0} + 4851000\cos(\theta)^2\cos(\phi)^4r^2$$
$$+24255000r^4\cos(\phi)^2\cos(\theta)^2 + 2522520\cos(\theta)^2r^4T_{5,0,0} - 240240\cos(\theta)^2r^2T_{3,0,0}$$
$$-1375920\cos(\theta)^2r^2T_{5,0,0} - 305760T_{5,0,0} - 137280T_{3,0,0} - 32032T_{1,0,0}$$
$$+240240r^2T_{3,0,0} + 9702000r^4\cos(\phi)^4 - 2425500r^4\cos(\theta)^2 - 4851000r^2\cos(\phi)^4$$
$$-9702000\cos(\phi)^2r^4 + 1375920r^2T_{5,0,0} - 5203800r^2 + 3638250r^4$$
$$-4851000\cos(\phi)^2r^2\cos(\theta)^2\big)\bar{e}_\phi$$

> # Construct the ODE for geostrophic flow

```
> B_cyl := simplify(MapToBasis(B_cart, cylindrical[s,phi,z]));
```

$$B\_cyl := -\frac{3}{104}\sqrt{5}\sqrt{26}\,s\left(15s^2+25z^2-21\right)\left(2\cos(\phi)^2-1\right)\bar{e}_s + \frac{75}{52}\sqrt{26}\Big(s^2+z^2 \quad \textbf{(40)}$$
$$-\frac{21}{25}\Big)\sin(\phi)\sqrt{5}\cos(\phi)\,s\bar{e}_\phi + \frac{15}{26}\sqrt{26}\,z\sqrt{5}\left(\cos(\phi)^2 - \frac{1}{2}\right)s^2\bar{e}_z$$

> $B\_sph := simplify(MapToBasis(B\_cyl, spherical[r, \text{theta}, \text{phi}]));$

$$B\_sph := -\frac{9}{104}\sin(\theta)^2\left(5r^2-7\right)\left(2\cos(\phi)^2-1\right)\sqrt{5}\sqrt{26}\,r\bar{e}_r \quad \textbf{(41)}$$
$$-\frac{3}{104}\cos(\theta)\sqrt{5}\sqrt{26}\,r\sin(\theta)\left(25r^2-21\right)\left(2\cos(\phi)^2-1\right)\bar{e}_\theta$$
$$+\frac{75}{52}\sqrt{26}\sin(\theta)\sin(\phi)\sqrt{5}\cos(\phi)\,r\left(r^2-\frac{21}{25}\right)\bar{e}_\phi$$

```
> SetCoordinates(cylindrical[s, phi, z])
```
$$cylindrical_{s,\,\phi,\,z} \tag{42}$$

```
> int(int(CrossProduct(Curl(B_cyl), B_cyl)[2], z=-sqrt(1-s^2)..sqrt(1 - s^2)), phi = 0..2·Pi);#check that it is a Taylor state
```
$$0 \tag{43}$$

```
> u_geo := ug(s) · VectorField([0, 1, 0]);#introduce geostrophic component
```
$$u\_geo := (ug(s))\bar{e}_{\phi} \tag{44}$$

```
> B_cyl_dot := simplify(eval(Curl(CrossProduct(u_cyl, B_cyl)) + Curl(CrossProduct(u_geo, B_cyl)) + eta · Laplacian(B_cyl))); # use induction equation
```
$$B\_cyl\_dot := -\frac{118125}{1352}\left(\sin(\phi)\,s^5\left(s^2-9z^2+\frac{7}{5}\right)\cos(\phi)^5-\sin(\phi)\,s^5\left(s^2-9z^2+\frac{7}{5}\right)\cos(\phi)^3+\frac{52}{375}\cos(\phi)^2\,\eta\,s-\left(\left(-\frac{52}{2625}s^2-\frac{52}{1575}z^2+\frac{52}{1875}\right)ug(s)+\left(s^6+\left(6z^2-\frac{37}{10}\right)s^4+\left(\frac{55}{8}z^4-\frac{207}{20}z^2+\frac{639}{200}\right)s^2+\frac{25}{8}z^6-\frac{63}{8}z^4+\frac{1107}{200}z^2-\frac{189}{200}\right)s\right)\sin(\phi)\cos(\phi)-\frac{26}{375}\,\eta\,s\right)\sqrt{26}\,\sqrt{5}\,\bar{e}_s-\frac{354375}{1352}\left(\frac{26}{7875}\left(\cos(\phi)^2-\frac{1}{2}\right)s\left(s^2+\frac{5}{3}z^2-\frac{7}{5}\right)\left(\frac{d}{ds}ug(s)\right)+s^5\left(s^2+\frac{7}{15}-3z^2\right)\cos(\phi)^6-\frac{3}{2}s^5\left(s^2+\frac{7}{15}-3z^2\right)\cos(\phi)^4+\left(\left(-\frac{26}{5625}+\frac{26}{3375}s^2+\frac{26}{4725}z^2\right)ug(s)-\frac{4}{3}\left(s^6+\left(\frac{9}{2}z^2-\frac{59}{20}\right)s^4+\left(\frac{85}{32}z^4-\frac{351}{80}z^2+\frac{1233}{800}\right)s^2+\frac{25}{32}z^6-\frac{63}{32}z^4+\frac{1107}{800}z^2-\frac{189}{800}\right)s\right)\cos(\phi)^2-\frac{52}{1125}\,\eta\sin(\phi)\,s\cos(\phi)+\left(-\frac{13}{3375}s^2-\frac{13}{4725}z^2+\frac{13}{5625}\right)ug(s)+\frac{11}{12}\left(s^6+\left(\frac{27}{11}z^2-\frac{111}{55}\right)s^4+\left(\frac{85}{44}z^4-\frac{351}{110}z^2+\frac{1233}{1100}\right)s^2+\frac{25}{44}z^6-\frac{63}{44}z^4+\frac{1107}{1100}z^2-\frac{189}{1100}\right)s\right)\sqrt{26}\,\sqrt{5}\,\bar{e}_{\phi}-\frac{590625}{676}\sqrt{5}\,\sin(\phi)\,z\cos(\phi)\,\sqrt{26}\left(\cos(\phi)^4 s^5-\cos(\phi)^2 s^5-\frac{52}{39375}ug(s)+\frac{7}{50}s^3+\left(\frac{1}{8}z^4-\frac{21}{100}z^2+\frac{9}{200}\right)s\right)s\bar{e}_z \tag{45}$$

```
> B_sph_dot := simplify(MapToBasis(B_cyl_dot, spherical[r, theta, phi]));
```
$$B\_sph\_dot := \frac{118125}{1352}\,\frac{1}{r}\left(\sqrt{5}\,\sqrt{26}\left(-\frac{52}{2625}\sin(\phi)\cos(\phi)\sqrt{r^2\sin(\theta)^2}\left(r^2-\frac{7}{5}\right)ug\left(\sqrt{r^2\sin(\theta)^2}\right)+(\cos(\theta)-1)\left(\left(\left(r^2+\frac{7}{5}\right)\sin(\phi)\,(\cos(\theta)-1)^2 r^4\,(\cos(\theta)+1)^2\cos(\phi)^5-\left(r^2+\frac{7}{5}\right)\sin(\phi)\,(\cos(\theta)-1)^2 r^4\,(\cos(\theta)+1)^2\cos(\phi)^3+\frac{52}{375}\cos(\phi)^2\,\eta+\frac{17}{8}\sin(\phi)\left(\left(r^6-\frac{91}{85}r^4\right)\cos(\theta)^4+\left(-\frac{24}{17}r^6\right.\right.\right.\right.\right.\right.$$

$$+\frac{174}{85} r^4 - \frac{378}{425} r^2\Big) \cos(\theta)^2 - \frac{8}{17} r^6 + \frac{148}{85} r^4 - \frac{639}{425} r^2 + \frac{189}{425}\Big)\cos(\phi)$$

$$-\frac{26}{375}\eta\Big) r^2 (\cos(\theta)+1)\Big)\Big)\bar{e}_r - \frac{1063125}{1352}\frac{1}{\sin(\theta)\, r}\Big(\sqrt{5}\Big(\frac{52}{14175}\Big(r^2$$

$$-\frac{21}{25}\Big)\sin(\phi)\cos(\phi)\sqrt{r^2\sin(\theta)^2}\, ug\Big(\sqrt{r^2\sin(\theta)^2}\Big) + \Big(\sin(\phi)\,(\cos(\theta)-1)^2\Big(r^2$$

$$-\frac{7}{45}\Big) r^4 (\cos(\theta)+1)^2\cos(\phi)^5 - \sin(\phi)\,(\cos(\theta)-1)^2\Big(r^2 - \frac{7}{45}\Big) r^4 (\cos(\theta)$$

$$+1)^2\cos(\phi)^3 - \frac{52}{3375}\cos(\phi)^2\eta - \frac{7}{72}\sin(\phi)\Big(\Big(r^6 - \frac{13}{5}r^4\Big)\cos(\theta)^4 + \Big(-\frac{24}{7} r^6$$

$$+\frac{314}{35} r^4 - \frac{54}{25} r^2\Big)\cos(\theta)^2 - \frac{8}{7} r^6 + \frac{92}{35} r^4 - \frac{729}{175} r^2 + \frac{27}{25}\Big)\cos(\phi) + \frac{26}{3375}\eta\Big)$$

$$(\cos(\theta)-1)\, r^2 (\cos(\theta)+1)\Big)\sqrt{26}\cos(\theta)\Big)\bar{e}_\theta - \frac{354375}{338}\frac{1}{\sin(\theta)\, r}\Big(\sqrt{26}\Big($$

$$-\frac{13}{23625}(\cos(\theta)-1)\Big(\cos(\phi)^2-\frac{1}{2}\Big)\Big(r^2\cos(\theta)^2+\frac{3}{2} r^2 - \frac{21}{10}\Big)(\cos(\theta)$$

$$+1)\, r^2 \left(\left.\frac{d}{d\_x}\, ug(\_x)\right|_{\_x=\sqrt{r^2\sin(\theta)^2}}\right) - \frac{13}{23625}\sqrt{r^2\sin(\theta)^2}\Big(\cos(\phi)^2$$

$$-\frac{1}{2}\Big)\Big(r^2\cos(\theta)^2 - \frac{7}{2} r^2 + \frac{21}{10}\Big)\, ug\Big(\sqrt{r^2\sin(\theta)^2}\Big) + (\cos(\theta)-1)\Big(\Big(\cos(\phi)^2$$

$$-\frac{1}{2}\Big)\Big(\cos(\phi)^4 - \cos(\phi)^2 + \frac{1}{24}\Big) r^6\cos(\theta)^6 - \frac{9}{4}\Big(\cos(\phi)^2-\frac{1}{2}\Big) r^4\Big(\Big(r^2$$

$$+\frac{7}{135}\Big)\cos(\phi)^4 + \Big(-r^2 - \frac{7}{135}\Big)\cos(\phi)^2 - \frac{1}{216} r^2 + \frac{19}{360}\Big)\cos(\theta)^4 + \frac{3}{2}\Big(\cos(\phi)^2$$

$$-\frac{1}{2}\Big)\Big(\Big(r^4+\frac{7}{45}r^2\Big)\cos(\phi)^4 + \Big(-r^4 - \frac{7}{45}r^2\Big)\cos(\phi)^2 - \frac{1}{6} r^4 + \frac{31}{120} r^2$$

$$-\frac{7}{200}\Big) r^2\cos(\theta)^2 + \Big(-\frac{7}{60}r^4 - \frac{1}{4} r^6\Big)\cos(\phi)^6 + \Big(\frac{7}{40}r^4 + \frac{3}{8} r^6\Big)\cos(\phi)^4 + \Big($$

$$-\frac{59}{60} r^4 + \frac{411}{800} r^2 - \frac{63}{800} + \frac{1}{3} r^6\Big)\cos(\phi)^2 + \frac{13}{1125}\sin(\phi)\cos(\phi)\,\eta + \frac{37}{80} r^4$$

$$-\frac{11}{48} r^6 - \frac{411}{1600} r^2 + \frac{63}{1600}\Big)(\cos(\theta)+1)\, r^2\Big)\sqrt{5}\Big)\bar{e}_\phi$$

> *SetCoordinates*(*spherical*[*r*, theta, phi])

$$\mathit{spherical}_{r,\,\theta,\,\phi} \quad (47)$$

> #numerically expand B_dot

> $N := 5;$ # order of chebysev expansion

$$N := 5 \quad (48)$$

> $M := N + 1 :$ # order of expansion including logarithmic term

> with(orthopoly) :

> $chebsum := add(a\_p \| n \cdot s \cdot T(n, 2 s^2 - 1), n = 1 ..N) :$

> $expansion := eval(chebsum) + b\_p \cdot s \cdot \ln(s);$

$$expansion := a\_p1\, s\,(2 s^2 - 1) + a\_p2\, s\,(-1 + 2 (2 s^2 - 1)^2) + a\_p3\, s\,(4 (2 s^2 - 1)^3 - 6 s^2 + 3) + a\_p4\, s\,(1 + 8 (2 s^2 - 1)^4 - 8 (2 s^2 - 1)^2) + a\_p5\, s\,(16 (2 s^2 - 1)^5 - 20 (2 s^2 - 1)^3 + 10 s^2 - 5) + b\_p\, s \ln(s) \quad (49)$$

> $B\_cyl\_dot\_approx := simplify(subs(ug(s) = expansion, B\_cyl\_dot));$

$$B\_cyl\_dot\_approx := -\frac{11520}{13}\sqrt{5}\left(\frac{2625}{26624}\sin(\phi)\left(s^2 - 9 z^2 + \frac{7}{5}\right) s^4 \cos(\phi)^5 - \frac{2625}{26624}\sin(\phi)\left(s^2 - 9 z^2 + \frac{7}{5}\right) s^4 \cos(\phi)^3 + \frac{7}{512}\cos(\phi)^2 \eta + \sin(\phi)\left(\frac{1}{512}\left(s^2 + \frac{5}{3} z^2 - \frac{7}{5}\right) b\_p \ln(s) + s^{12} a\_p5 + \left(\frac{1}{4} a\_p4 - \frac{39}{10} a\_p5 + \frac{5}{3} z^2 a\_p5\right) s^{10} + \left(\left(\frac{5}{12} a\_p4 - \frac{25}{6} a\_p5\right) z^2 + \frac{1}{16} a\_p3 - \frac{17}{20} a\_p4 + \frac{91}{16} a\_p5\right) s^8 + \left(\left(\frac{5}{48} a\_p3 - \frac{5}{6} a\_p4 + \frac{175}{48} a\_p5\right) z^2 - \frac{29}{160} a\_p3 + \frac{1}{64} a\_p2 + \frac{81}{80} a\_p4 - \frac{123}{32} a\_p5 - \frac{2625}{26624}\right) s^6 + \left(\left(\frac{5}{192} a\_p2 - \frac{5}{32} a\_p3 + \frac{25}{48} a\_p4 - \frac{125}{96} a\_p5 - \frac{7875}{13312}\right) z^2 + \frac{19425}{53248} + \frac{1}{256} a\_p1 - \frac{3}{80} a\_p2 - \frac{1}{2} a\_p4 + \frac{305}{256} a\_p5 + \frac{213}{1280} a\_p3\right) s^4 + \left(-\frac{144375}{212992} z^4 + \left(\frac{108675}{106496} + \frac{5}{768} a\_p1 - \frac{5}{192} a\_p2 + \frac{15}{256} a\_p3 - \frac{5}{48} a\_p4 + \frac{125}{768} a\_p5\right) z^2 - \frac{19}{2560} a\_p1 - \frac{131}{2560} a\_p3 + \frac{61}{2560} a\_p2 - \frac{71}{512} a\_p5 + \frac{229}{2560} a\_p4 - \frac{67095}{212992}\right) s^2 - \frac{5}{1536}\left(\frac{39375}{416} z^4 - \frac{33075}{208} z^2 + a\_p5 + a\_p1 - a\_p2 + a\_p3 - a\_p4 + \frac{14175}{416}\right)\left(z^2 - \frac{21}{25}\right)\right)\cos(\phi) - \frac{7}{1024}\eta\right)\sqrt{26}\, s\bar{e}_s - \frac{76800}{13}\sqrt{5}\left(\frac{4725}{106496}\left(s^2 + \frac{7}{15} - 3 z^2\right) s^4 \cos(\phi)^6 - \frac{14175}{212992}\left(s^2 + \frac{7}{15} - 3 z^2\right) s^4 \cos(\phi)^4 + \left(\frac{1}{2048}\left(s^2 + z^2 - \frac{21}{25}\right) b\_p \ln(s) + s^{12} a\_p5 + \left(\frac{17}{80} a\_p4\right.\right.\right.$$

$$- \frac{677}{200} a\_p5 + \frac{3}{2} z^2 a\_p5 \Big) s^{10} + \Big(\Big(\frac{5}{16} a\_p4 - \frac{25}{8} a\_p5 \Big) z^2 + \frac{7}{160} a\_p3$$

$$- \frac{49}{80} a\_p4 + \frac{133}{32} a\_p5 \Big) s^8 + \Big(\Big(\frac{1}{16} a\_p3 - \frac{1}{2} a\_p4 + \frac{35}{16} a\_p5 \Big) z^2 - \frac{333}{3200} a\_p3$$

$$+ \frac{11}{1280} a\_p2 + \frac{947}{1600} a\_p4 - \frac{1451}{640} a\_p5 - \frac{1575}{26624} \Big) s^6 + \Big(\Big(\frac{3}{256} a\_p2$$

$$- \frac{9}{128} a\_p3 + \frac{15}{64} a\_p4 - \frac{75}{128} a\_p5 - \frac{14175}{53248} \Big) z^2 + \frac{18585}{106496} + \frac{1}{640} a\_p1$$

$$- \frac{103}{6400} a\_p2 - \frac{71}{320} a\_p4 + \frac{17}{32} a\_p5 + \frac{117}{1600} a\_p3 \Big) s^4 + \Big(-\frac{133875}{851968} z^4 + \Big(\frac{8505}{32768}$$

$$+ \frac{1}{512} a\_p1 - \frac{1}{128} a\_p2 + \frac{9}{512} a\_p3 - \frac{1}{32} a\_p4 + \frac{25}{512} a\_p5 \Big) z^2 - \frac{109}{51200} a\_p1$$

$$- \frac{781}{51200} a\_p3 + \frac{361}{51200} a\_p2 - \frac{85}{2048} a\_p5 + \frac{3}{20480} b\_p + \frac{1369}{51200} a\_p4$$

$$- \frac{77679}{851968} \Big) s^2 - \frac{1}{2048} \Big(\frac{39375}{416} z^4 - \frac{33075}{208} z^2 + a\_p5 - \frac{1}{2} b\_p + a\_p1 - a\_p2$$

$$+ a\_p3 - a\_p4 + \frac{14175}{416} \Big) \Big(z^2 - \frac{21}{25} \Big) \Big) \cos(\phi)^2 - \frac{21}{10240} \sin(\phi) \cos(\phi) \eta$$

$$- \frac{1}{4096} \Big(s^2 + z^2 - \frac{21}{25} \Big) b\_p \ln(s) - \frac{1}{2} s^{12} a\_p5 + \Big(-\frac{17}{160} a\_p4 + \frac{677}{400} a\_p5$$

$$- \frac{3}{4} z^2 a\_p5 \Big) s^{10} + \Big(\Big(-\frac{5}{32} a\_p4 + \frac{25}{16} a\_p5 \Big) z^2 - \frac{7}{320} a\_p3 + \frac{49}{160} a\_p4$$

$$- \frac{133}{64} a\_p5 \Big) s^8 + \Big(\Big(-\frac{1}{32} a\_p3 + \frac{1}{4} a\_p4 - \frac{35}{32} a\_p5 \Big) z^2 + \frac{333}{6400} a\_p3$$

$$- \frac{11}{2560} a\_p2 - \frac{947}{3200} a\_p4 + \frac{1451}{1280} a\_p5 + \frac{17325}{425984} \Big) s^6 + \Big(\Big(-\frac{3}{512} a\_p2$$

$$+ \frac{9}{256} a\_p3 - \frac{15}{128} a\_p4 + \frac{75}{256} a\_p5 + \frac{42525}{425984} \Big) z^2 - \frac{34965}{425984} - \frac{1}{1280} a\_p1$$

$$+ \frac{103}{12800} a\_p2 + \frac{71}{640} a\_p4 - \frac{17}{64} a\_p5 - \frac{117}{3200} a\_p3 \Big) s^4 + \Big(\frac{133875}{1703936} z^4 + \Big($$

$$-\frac{8505}{65536} - \frac{1}{1024} a\_p1 + \frac{1}{256} a\_p2 - \frac{9}{1024} a\_p3 + \frac{1}{64} a\_p4 - \frac{25}{1024} a\_p5 \Big) z^2$$

$$+ \frac{109}{102400} a\_p1 + \frac{781}{102400} a\_p3 - \frac{361}{102400} a\_p2 + \frac{85}{4096} a\_p5 - \frac{3}{40960} b\_p$$

$$- \frac{1369}{102400} a\_p4 + \frac{77679}{1703936} \Big) s^2 + \frac{1}{4096} \Big(\frac{39375}{416} z^4 - \frac{33075}{208} z^2 + a\_p5 - \frac{1}{2} b\_p$$

$$+ a\_p1 - a\_p2 + a\_p3 - a\_p4 + \frac{14175}{416} \Big) \Big(z^2 - \frac{21}{25} \Big) \Big) \sqrt{26}\, \bar{se}_{\phi}$$

$$+ \frac{7680}{13} \sqrt{26}\, z \sin(\phi) \sqrt{5} \cos(\phi) s^2 \Big(-\frac{39375}{26624} s^4 \cos(\phi)^4 + \frac{39375}{26624} \cos(\phi)^2 s^4$$

$$+ \frac{1}{512} b\_p \ln(s) + a\_p5\, s^{10} + \Big(\frac{1}{4} a\_p4 - \frac{5}{2} a\_p5 \Big) s^8 + \Big(\frac{1}{16} a\_p3 - \frac{1}{2} a\_p4$$

$$+ \frac{35}{16} a\_p5 \Big) s^6 + \Big(\frac{1}{64} a\_p2 - \frac{3}{32} a\_p3 - \frac{25}{32} a\_p5 + \frac{5}{16} a\_p4 \Big) s^4 + \Big(\frac{1}{256} a\_p1$$

$$- \frac{1}{64}\, a\_p2 - \frac{1}{16}\, a\_p4 + \frac{9}{256}\, a\_p3 + \frac{25}{256}\, a\_p5 - \frac{11025}{53248} \Big) s^2 - \frac{1}{512}\, a\_p5 + \frac{33075}{106496}\, z^2 + \frac{1}{512}\, a\_p4 - \frac{14175}{212992} - \frac{1}{512}\, a\_p3 - \frac{1}{512}\, a\_p1 + \frac{1}{512}\, a\_p2 - \frac{39375}{212992}\, z^4 \Big) \bar{e}_z$$

> B_sph_dot_approx := simplify(MapToBasis(B_cyl_dot_approx, spherical[r, theta, phi]));

$$B\_sph\_dot\_approx := \frac{11520}{13} \Big(-\frac{1}{1024}\, b\_p \sin(\phi) \left(r^2 - \frac{7}{5}\right) \cos(\phi) \ln\left(r^2 \sin(\theta)^2\right) - \frac{2625}{26624} \left(r^2 + \frac{7}{5}\right) \sin(\phi) (\cos(\theta) - 1)^2 r^4 (\cos(\theta) + 1)^2 \cos(\phi)^5 + \frac{2625}{26624} \left(r^2 + \frac{7}{5}\right) \sin(\phi) (\cos(\theta) - 1)^2 r^4 (\cos(\theta) + 1)^2 \cos(\phi)^3 - \frac{7}{512} \cos(\phi)^2 \eta + \Big(r^{10}\, a\_p5 \left(r^2 - \frac{7}{5}\right) \cos(\theta)^{10} - 5 \left(r^2 - \frac{7}{5}\right) r^8 \left(r^2\, a\_p5 + \frac{1}{20}\, a\_p4 - \frac{1}{2}\, a\_p5\right) \cos(\theta)^8 + 10 \left(r^2 - \frac{7}{5}\right) r^6 \Big(a\_p5\, r^4 + \left(\frac{1}{10}\, a\_p4 - a\_p5\right) r^2 + \frac{1}{160}\, a\_p3 - \frac{1}{20}\, a\_p4 + \frac{7}{32}\, a\_p5\Big) \cos(\theta)^6 - 10\, r^4 \Big(a\_p5\, r^8 + \left(\frac{3}{20}\, a\_p4 - \frac{29}{10}\, a\_p5\right) r^6 + \left(\frac{3}{160}\, a\_p3 - \frac{9}{25}\, a\_p4 + \frac{441}{160}\, a\_p5\right) r^4 + \left(\frac{1}{640}\, a\_p2 - \frac{57}{1600}\, a\_p3 + \frac{193}{800}\, a\_p4 - \frac{319}{320}\, a\_p5 + \frac{8925}{425984}\right) r^2 - \frac{7}{3200}\, a\_p2 + \frac{21}{1600}\, a\_p3 - \frac{7}{160}\, a\_p4 + \frac{7}{64}\, a\_p5 - \frac{735}{32768}\Big) \cos(\theta)^4 + 5 \Big(a\_p5\, r^{10} + \left(\frac{1}{5}\, a\_p4 - \frac{17}{5}\, a\_p5\right) r^8 + \left(\frac{3}{80}\, a\_p3 - \frac{29}{50}\, a\_p4 + \frac{329}{80}\, a\_p5\right) r^6 + \left(\frac{1}{160}\, a\_p2 - \frac{9}{100}\, a\_p3 + \frac{109}{200}\, a\_p4 - \frac{43}{20}\, a\_p5 + \frac{1575}{26624}\right) r^4 + \left(\frac{1}{1280}\, a\_p1 - \frac{19}{1600}\, a\_p2 + \frac{381}{6400}\, a\_p3 - \frac{3}{16}\, a\_p4 + \frac{117}{256}\, a\_p5 - \frac{9135}{106496}\right) r^2 - \frac{7}{6400}\, a\_p1 + \frac{7}{1600}\, a\_p2 - \frac{63}{6400}\, a\_p3 + \frac{7}{400}\, a\_p4 - \frac{7}{256}\, a\_p5 + \frac{3969}{106496}\Big) r^2 \cos(\theta)^2 - a\_p5\, r^{12} + \left(\frac{39}{10}\, a\_p5 - \frac{1}{4}\, a\_p4\right) r^{10} + \left(\frac{17}{20}\, a\_p4 - \frac{91}{16}\, a\_p5 - \frac{1}{16}\, a\_p3\right) r^8 + \left(-\frac{81}{80}\, a\_p4 + \frac{29}{160}\, a\_p3 + \frac{123}{32}\, a\_p5 - \frac{1}{64}\, a\_p2 + \frac{2625}{26624}\right) r^6 + \left(-\frac{19425}{53248} + \frac{3}{80}\, a\_p2 - \frac{213}{1280}\, a\_p3 - \frac{305}{256}\, a\_p5 - \frac{1}{256}\, a\_p1 + \frac{1}{2}\, a\_p4\right) r^4 + \left(\frac{19}{2560}\, a\_p1 - \frac{61}{2560}\, a\_p2 - \frac{229}{2560}\, a\_p4 + \frac{131}{2560}\, a\_p3 + \frac{71}{512}\, a\_p5 + \frac{67095}{212992}\right) r^2 - \frac{7}{2560}\, a\_p5 + \frac{7}{2560}\, a\_p4 - \frac{19845}{212992} - \frac{7}{2560}\, a\_p3 - \frac{7}{2560}\, a\_p1 + \frac{7}{2560}\, a\_p2 \Big) \sin(\phi) \cos(\phi) + \frac{7}{1024}\, \eta \Big) \sqrt{26}\, \sqrt{5}\, \sin(\theta)^2\, r \bar{e}_r \qquad \textbf{(51)}$$

$$+\frac{19200}{13}\sqrt{26}\left(-\frac{1}{1024}\left(r^2-\frac{21}{25}\right)b\_p\sin(\phi)\cos(\phi)\ln\left(r^2\sin(\theta)^2\right)\right.$$
$$+\frac{14175}{26624}\sin(\phi)\left(\cos(\theta)-1\right)^2\left(r^2-\frac{7}{45}\right)r^4\left(\cos(\theta)+1\right)^2\cos(\phi)^5$$
$$-\frac{14175}{26624}\sin(\phi)\left(\cos(\theta)-1\right)^2\left(r^2-\frac{7}{45}\right)r^4\left(\cos(\theta)+1\right)^2\cos(\phi)^3$$
$$-\frac{21}{2560}\cos(\phi)^2\eta+\sin(\phi)\left(r^{10}a\_p5\left(r^2-\frac{21}{25}\right)\cos(\theta)^{10}-5\left(r^2\right.\right.$$
$$\left.-\frac{21}{25}\right)r^8\left(r^2a\_p5+\frac{1}{20}a\_p4-\frac{1}{2}a\_p5\right)\cos(\theta)^8+10\left(r^2-\frac{21}{25}\right)r^6\left(a\_p5\,r^4\right.$$
$$\left.+\left(\frac{1}{10}a\_p4-a\_p5\right)r^2+\frac{1}{160}a\_p3-\frac{1}{20}a\_p4+\frac{7}{32}a\_p5\right)\cos(\theta)^6$$
$$-10\,r^4\left(a\_p5\,r^8+\left(\frac{3}{20}a\_p4-\frac{117}{50}a\_p5\right)r^6+\left(\frac{3}{160}a\_p3-\frac{69}{250}a\_p4\right.\right.$$
$$\left.+\frac{1533}{800}a\_p5\right)r^4+\left(\frac{1}{640}a\_p2-\frac{201}{8000}a\_p3+\frac{629}{4000}a\_p4-\frac{1007}{1600}a\_p5\right.$$
$$\left.\left.+\frac{2205}{425984}\right)r^2-\frac{21}{16000}a\_p2+\frac{63}{8000}a\_p3-\frac{21}{800}a\_p4+\frac{21}{320}a\_p5-\frac{441}{32768}\right)$$
$$\cos(\theta)^4+5\left(a\_p5\,r^{10}+\left(\frac{1}{5}a\_p4-\frac{71}{25}a\_p5\right)r^8+\left(\frac{3}{80}a\_p3-\frac{117}{250}a\_p4\right.\right.$$
$$\left.+\frac{1197}{400}a\_p5\right)r^6+\left(\frac{1}{160}a\_p2-\frac{69}{1000}a\_p3+\frac{377}{1000}a\_p4-\frac{283}{200}a\_p5\right.$$
$$\left.+\frac{945}{26624}\right)r^4+\left(\frac{1}{1280}a\_p1-\frac{67}{8000}a\_p2+\frac{1233}{32000}a\_p3-\frac{47}{400}a\_p4\right.$$
$$\left.+\frac{361}{1280}a\_p5-\frac{9891}{106496}\right)r^2-\frac{21}{32000}a\_p1+\frac{21}{8000}a\_p2-\frac{189}{32000}a\_p3$$
$$\left.+\frac{21}{2000}a\_p4-\frac{21}{1280}a\_p5+\frac{11907}{532480}\right)r^2\cos(\theta)^2-a\_p5\,r^{12}+\left(\frac{167}{50}a\_p5\right.$$
$$\left.-\frac{1}{4}a\_p4\right)r^{10}+\left(\frac{71}{100}a\_p4-\frac{343}{80}a\_p5-\frac{1}{16}a\_p3\right)r^8+\left(-\frac{293}{400}a\_p4\right.$$
$$\left.+\frac{117}{800}a\_p3+\frac{419}{160}a\_p5-\frac{1}{64}a\_p2+\frac{1575}{26624}\right)r^6+\left(-\frac{7245}{53248}+\frac{23}{800}a\_p2\right.$$
$$\left.-\frac{729}{6400}a\_p3-\frac{193}{256}a\_p5-\frac{1}{256}a\_p1+\frac{13}{40}a\_p4\right)r^4+\left(\frac{67}{12800}a\_p1\right.$$
$$\left.-\frac{193}{12800}a\_p2-\frac{697}{12800}a\_p4+\frac{403}{12800}a\_p3+\frac{43}{512}a\_p5+\frac{45927}{212992}\right)r^2-\frac{11907}{212992}$$
$$\left.-\frac{21}{12800}a\_p5+\frac{21}{12800}a\_p4-\frac{21}{12800}a\_p3-\frac{21}{12800}a\_p1+\frac{21}{12800}a\_p2\right)$$
$$\left.\cos(\phi)+\frac{21}{5120}\eta\right)\cos(\theta)\sqrt{5}\sin(\theta)\,r\bar{e}_{\theta}+\frac{38400}{13}\sqrt{26}\sqrt{5}\left(-\frac{1}{2048}\left(r^2\right.\right.$$
$$\left.-\frac{21}{25}\right)b\_p\left(\cos(\phi)^2-\frac{1}{2}\right)\ln\left(r^2\sin(\theta)^2\right)+a\_p5\,r^{12}\left(\cos(\phi)^2-\frac{1}{2}\right)\cos(\theta)^{12}$$
$$-3\left(r^2a\_p5+\frac{1}{15}a\_p4+\frac{13}{75}a\_p5\right)\left(\cos(\phi)^2-\frac{1}{2}\right)r^{10}\cos(\theta)^{10}+\frac{3}{8}\left(\cos(\phi)^2\right.$$

$$-\frac{1}{2}\Big)\Big(\Big(\Big(a\_p4+\frac{118}{5}a\_p5\Big)r^2+\frac{1}{10}a\_p3+\frac{3}{5}a\_p4-\frac{21}{2}a\_p5\Big)r^8\cos(\theta)^8$$

$$+10\Big(\frac{945}{26624}\cos(\phi)^4-\frac{945}{26624}\cos(\phi)^2+a\_p5\,r^6+\Big(\frac{1}{20}a\_p4-\frac{151}{50}a\_p5\Big)r^4+\Big($$

$$-\frac{1}{400}a\_p3-\frac{19}{100}a\_p4+\frac{161}{80}a\_p5\Big)r^2-\frac{1}{1600}a\_p2-\frac{27}{4000}a\_p3+\frac{143}{2000}a\_p4$$

$$-\frac{269}{800}a\_p5+\frac{315}{212992}\Big)\Big(\cos(\phi)^2-\frac{1}{2}\Big)r^6\cos(\theta)^6-15\Big(\cos(\phi)^2$$

$$-\frac{1}{2}\Big)r^4\Big(\Big(\Big(\frac{2835}{53248}r^2+\frac{147}{53248}\Big)\cos(\phi)^4+\Big(-\frac{2835}{53248}r^2-\frac{147}{53248}\Big)\cos(\phi)^2$$

$$+a\_p5\,r^8+\Big(\frac{7}{60}a\_p4-\frac{427}{150}a\_p5\Big)r^6+\Big(\frac{1}{100}a\_p3-\frac{29}{100}a\_p4+\frac{49}{20}a\_p5\Big)r^4$$

$$+\Big(\frac{1}{3200}a\_p2-\frac{183}{8000}a\_p3+\frac{697}{4000}a\_p4-\frac{1201}{1600}a\_p5-\frac{105}{425984}\Big)r^2$$

$$-\frac{1}{19200}a\_p1-\frac{53}{48000}a\_p2+\frac{237}{32000}a\_p3-\frac{61}{2400}a\_p4+\frac{247}{3840}a\_p5$$

$$+\frac{1197}{425984}\Big)\cos(\theta)^4+9\Big(\cos(\phi)^2-\frac{1}{2}\Big)\Big(\frac{1575}{26624}r^2\Big(r^2+\frac{7}{45}\Big)\cos(\phi)^4$$

$$-\frac{1575}{26624}r^2\Big(r^2+\frac{7}{45}\Big)\cos(\phi)^2+a\_p5\,r^{10}+\Big(\frac{1}{6}a\_p4-\frac{46}{15}a\_p5\Big)r^8+\Big($$

$$-\frac{13}{30}a\_p4+\frac{1}{40}a\_p3+\frac{77}{24}a\_p5\Big)r^6+\Big(-\frac{525}{53248}+\frac{1}{320}a\_p2-\frac{43}{800}a\_p3$$

$$-\frac{221}{160}a\_p5+\frac{137}{400}a\_p4\Big)r^4+\Big(\frac{1}{3840}a\_p1-\frac{13}{2400}a\_p2-\frac{11}{120}a\_p4$$

$$+\frac{183}{6400}a\_p3+\frac{173}{768}a\_p5+\frac{3255}{212992}\Big)r^2-\frac{441}{212992}-\frac{7}{768}a\_p5+\frac{7}{1200}a\_p4$$

$$-\frac{1}{46080}b\_p-\frac{21}{6400}a\_p3-\frac{7}{19200}a\_p1+\frac{7}{4800}a\_p2\Big)r^2\cos(\theta)^2-\frac{4725}{53248}\Big(r^2$$

$$+\frac{7}{15}\Big)r^4\cos(\phi)^6+\frac{14175}{106496}\Big(r^2+\frac{7}{15}\Big)r^4\cos(\phi)^4+\Big(-2\,a\_p5\,r^{12}+\Big(\frac{677}{100}a\_p5$$

$$-\frac{17}{40}a\_p4\Big)r^{10}+\Big(\frac{49}{40}a\_p4-\frac{133}{16}a\_p5-\frac{7}{80}a\_p3\Big)r^8+\Big(-\frac{947}{800}a\_p4$$

$$+\frac{333}{1600}a\_p3+\frac{1451}{320}a\_p5-\frac{11}{640}a\_p2+\frac{1575}{13312}\Big)r^6+\Big(-\frac{18585}{53248}+\frac{103}{3200}a\_p2$$

$$-\frac{117}{800}a\_p3-\frac{17}{16}a\_p5-\frac{1}{320}a\_p1+\frac{71}{160}a\_p4\Big)r^4+\Big(\frac{109}{25600}a\_p1$$

$$-\frac{361}{25600}a\_p2-\frac{1369}{25600}a\_p4+\frac{781}{25600}a\_p3+\frac{85}{1024}a\_p5-\frac{3}{10240}b\_p$$

$$+\frac{77679}{425984}\Big)r^2-\frac{11907}{425984}-\frac{21}{25600}a\_p5+\frac{21}{25600}a\_p4+\frac{21}{51200}b\_p$$

$$-\frac{21}{25600}a\_p3-\frac{21}{25600}a\_p1+\frac{21}{25600}a\_p2\Big)\cos(\phi)^2+\frac{21}{5120}\sin(\phi)\cos(\phi)\,\eta$$

$$+a\_p5\,r^{12}+\Big(-\frac{677}{200}a\_p5+\frac{17}{80}a\_p4\Big)r^{10}+\Big(-\frac{49}{80}a\_p4+\frac{133}{32}a\_p5$$

$$+\frac{7}{160}\,a\_p3\Big)\,r^8+\Big(\frac{947}{1600}\,a\_p4-\frac{333}{3200}\,a\_p3-\frac{1451}{640}\,a\_p5+\frac{11}{1280}\,a\_p2$$
$$-\frac{17325}{212992}\Big)\,r^6+\Big(\frac{34965}{212992}-\frac{103}{6400}\,a\_p2+\frac{117}{1600}\,a\_p3+\frac{17}{32}\,a\_p5+\frac{1}{640}\,a\_p1$$
$$-\frac{71}{320}\,a\_p4\Big)\,r^4+\Big(-\frac{109}{51200}\,a\_p1+\frac{361}{51200}\,a\_p2+\frac{1369}{51200}\,a\_p4-\frac{781}{51200}\,a\_p3$$
$$-\frac{85}{2048}\,a\_p5+\frac{3}{20480}\,b\_p-\frac{77679}{851968}\Big)\,r^2+\frac{21}{51200}\,a\_p5-\frac{21}{51200}\,a\_p4$$
$$-\frac{21}{102400}\,b\_p+\frac{11907}{851968}+\frac{21}{51200}\,a\_p3+\frac{21}{51200}\,a\_p1-\frac{21}{51200}\,a\_p2\Big)\sin(\theta)\,\bar{re}_{\phi}$$

> *#find toroidal and poloidal components of B_dot*

> *toroidal_part := simplify(expand(Curl(B_sph_dot_approx)[1]*r^2));*

$$toroidal\_part := \frac{537600}{13}\left(\cos(\phi)^2-\frac{1}{2}\right)\sqrt{5}\,\Big(\cos(\theta)^{12}\,a\_p5\,r^{12}-\frac{7}{2}\,\Big(r^2\,a\_p5 \tag{52}$$
$$+\frac{12}{245}\,a\_p4+\frac{27}{245}\,a\_p5\Big)\,r^{10}\cos(\theta)^{10}+\frac{5}{2}\,\Big(a\_p5\,r^4+\Big(\frac{6}{35}\,a\_p4+\frac{87}{35}\,a\_p5\Big)\,r^2$$
$$+\frac{3}{280}\,a\_p3+\frac{51}{875}\,a\_p4-\frac{213}{200}\,a\_p5\Big)\,r^8\cos(\theta)^8+5\,\Big(\frac{135}{3328}\cos(\phi)^4$$
$$-\frac{135}{3328}\cos(\phi)^2+a\_p5\,r^6-\frac{21}{5}\,a\_p5\,r^4+\Big(-\frac{9}{1120}\,a\_p3-\frac{783}{3500}\,a\_p4$$
$$+\frac{2079}{800}\,a\_p5\Big)\,r^2-\frac{1}{1400}\,a\_p2-\frac{39}{5600}\,a\_p3+\frac{53}{700}\,a\_p4-\frac{401}{1120}\,a\_p5+\frac{45}{26624}\Big)$$
$$r^6\cos(\theta)^6-10\,\Big(\frac{405}{6656}\,r^2\cos(\phi)^4-\frac{405}{6656}\cos(\phi)^2\,r^2+a\_p5\,r^8+\Big(\frac{3}{35}\,a\_p4$$
$$-\frac{207}{70}\,a\_p5\Big)\,r^6+\Big(\frac{9}{2240}\,a\_p3-\frac{1737}{7000}\,a\_p4+\frac{3681}{1600}\,a\_p5\Big)\,r^4+\Big(-\frac{1}{5600}\,a\_p2$$
$$-\frac{177}{11200}\,a\_p3+\frac{23}{175}\,a\_p4-\frac{1303}{2240}\,a\_p5+\frac{45}{425984}\Big)\,r^2-\frac{3}{89600}\,a\_p1$$
$$-\frac{69}{112000}\,a\_p2+\frac{1881}{448000}\,a\_p3-\frac{81}{5600}\,a\_p4+\frac{657}{17920}\,a\_p5+\frac{1179}{425984}\Big)$$
$$r^4\cos(\theta)^4+\frac{13}{2}\,\Big(\frac{2025}{21632}\,r^4\cos(\phi)^4-\frac{2025}{21632}\cos(\phi)^2\,r^4+a\_p5\,r^{10}+\Big(-\frac{267}{91}\,a\_p5$$
$$+\frac{12}{91}\,a\_p4\Big)\,r^8+\Big(\frac{2829}{1040}\,a\_p5-\frac{219}{650}\,a\_p4+\frac{3}{208}\,a\_p3\Big)\,r^6+\Big(-\frac{8325}{1384448}$$
$$-\frac{237}{7280}\,a\_p3+\frac{209}{910}\,a\_p4+\frac{1}{910}\,a\_p2-\frac{1403}{1456}\,a\_p5\Big)\,r^4+\Big(-\frac{27}{11200}\,a\_p2$$
$$+\frac{8199}{582400}\,a\_p3+\frac{2703}{23296}\,a\_p5-\frac{339}{7280}\,a\_p4+\frac{1125}{86528}+\frac{3}{116480}\,a\_p1\Big)\,r^2$$
$$-\frac{2835}{1384448}-\frac{9}{3328}\,a\_p5-\frac{1}{116480}\,b\_p+\frac{9}{5200}\,a\_p4-\frac{9}{83200}\,a\_p1$$
$$+\frac{9}{20800}\,a\_p2-\frac{81}{83200}\,a\_p3\Big)\,r^2\cos(\theta)^2-\frac{675}{3328}\cos(\phi)^4\,r^6+\frac{675}{3328}\cos(\phi)^2\,r^6$$

$$- \frac{3}{2}\, a\_p5\, r^{12} + \left(\frac{327}{70}\, a\_p5 - \frac{9}{35}\, a\_p4\right) r^{10} + \left(-\frac{801}{160}\, a\_p5 + \frac{477}{700}\, a\_p4 - \frac{9}{224}\, a\_p3\right) r^{8} + \left(\frac{501}{224}\, a\_p5 - \frac{39}{70}\, a\_p4 + \frac{99}{1120}\, a\_p3 + \frac{3375}{106496} - \frac{3}{560}\, a\_p2\right) r^{6} + \left(-\frac{12105}{212992} - \frac{4437}{89600}\, a\_p3 + \frac{177}{1120}\, a\_p4 + \frac{213}{22400}\, a\_p2 - \frac{1389}{3584}\, a\_p5 - \frac{9}{17920}\, a\_p1\right) r^{4} + \left(-\frac{9}{3200}\, a\_p2 + \frac{81}{12800}\, a\_p3 + \frac{9}{512}\, a\_p5 - \frac{9}{800}\, a\_p4 - \frac{3}{35840}\, b\_p + \frac{2835}{212992} + \frac{9}{12800}\, a\_p1\right) r^{2} + \frac{3}{25600}\, b\_p\Bigg) \sqrt{26}\, \cos(\theta)\, r^{2}$$

```
> poloidal_part := simplify(B_sph_dot_approx[1]*r^2);
```

$$poloidal\_part := \frac{11520}{13} \Bigg(-\frac{1}{1024}\, b\_p \sin(\phi) \left(r^{2} - \frac{7}{5}\right) \cos(\phi) \ln\left(r^{2} \sin(\theta)^{2}\right) - \frac{2625}{26624} \left(r^{2} + \frac{7}{5}\right) \sin(\phi) \left(\cos(\theta) - 1\right)^{2} r^{4} \left(\cos(\theta) + 1\right)^{2} \cos(\phi)^{5} + \frac{2625}{26624} \left(r^{2} + \frac{7}{5}\right) \sin(\phi) \left(\cos(\theta) - 1\right)^{2} r^{4} \left(\cos(\theta) + 1\right)^{2} \cos(\phi)^{3} - \frac{7}{512} \cos(\phi)^{2}\, \eta + \Bigg(r^{10}\, a\_p5 \left(r^{2} - \frac{7}{5}\right) \cos(\theta)^{10} - 5 \left(r^{2} - \frac{7}{5}\right) r^{8} \left(r^{2}\, a\_p5 + \frac{1}{20}\, a\_p4 - \frac{1}{2}\, a\_p5\right) \cos(\theta)^{8} + 10 \left(r^{2} - \frac{7}{5}\right) r^{6} \left(a\_p5\, r^{4} + \left(\frac{1}{10}\, a\_p4 - a\_p5\right) r^{2} + \frac{1}{160}\, a\_p3 - \frac{1}{20}\, a\_p4 + \frac{7}{32}\, a\_p5\right) \cos(\theta)^{6} - 10\, r^{4} \Bigg(a\_p5\, r^{8} + \left(\frac{3}{20}\, a\_p4 - \frac{29}{10}\, a\_p5\right) r^{6} + \left(\frac{3}{160}\, a\_p3 - \frac{9}{25}\, a\_p4 + \frac{441}{160}\, a\_p5\right) r^{4} + \left(\frac{1}{640}\, a\_p2 - \frac{57}{1600}\, a\_p3 + \frac{193}{800}\, a\_p4 - \frac{319}{320}\, a\_p5 + \frac{8925}{425984}\right) r^{2} - \frac{7}{3200}\, a\_p2 + \frac{21}{1600}\, a\_p3 - \frac{7}{160}\, a\_p4 + \frac{7}{64}\, a\_p5 - \frac{735}{32768}\Bigg) \cos(\theta)^{4} + 5 \Bigg(a\_p5\, r^{10} + \left(\frac{1}{5}\, a\_p4 - \frac{17}{5}\, a\_p5\right) r^{8} + \left(\frac{3}{80}\, a\_p3 - \frac{29}{50}\, a\_p4 + \frac{329}{80}\, a\_p5\right) r^{6} + \left(\frac{1}{160}\, a\_p2 - \frac{9}{100}\, a\_p3 + \frac{109}{200}\, a\_p4 - \frac{43}{20}\, a\_p5 + \frac{1575}{26624}\right) r^{4} + \left(\frac{1}{1280}\, a\_p1 - \frac{19}{1600}\, a\_p2 + \frac{381}{6400}\, a\_p3 - \frac{3}{16}\, a\_p4 + \frac{117}{256}\, a\_p5 - \frac{9135}{106496}\right) r^{2} - \frac{7}{6400}\, a\_p1 + \frac{7}{1600}\, a\_p2 - \frac{63}{6400}\, a\_p3 + \frac{7}{400}\, a\_p4 - \frac{7}{256}\, a\_p5 + \frac{3969}{106496}\Bigg) r^{2} \cos(\theta)^{2} - a\_p5\, r^{12} + \left(\frac{39}{10}\, a\_p5 - \frac{1}{4}\, a\_p4\right) r^{10} + \left(\frac{17}{20}\, a\_p4 - \frac{91}{16}\, a\_p5 - \frac{1}{16}\, a\_p3\right) r^{8} + \left(-\frac{81}{80}\, a\_p4 + \frac{29}{160}\, a\_p3 + \frac{123}{32}\, a\_p5 - \frac{1}{64}\, a\_p2 + \frac{2625}{26624}\right) r^{6} + \left(-\frac{19425}{53248} + \frac{3}{80}\, a\_p2 - \frac{213}{1280}\, a\_p3 - \frac{305}{256}\, a\_p5 - \frac{1}{256}\, a\_p1 + \frac{1}{2}\, a\_p4\right) r^{4} + \Bigg(\frac{19}{2560}\, a\_p1 - \frac{61}{2560}\, a\_p2 - \frac{229}{2560}\, a\_p4 + \frac{131}{2560}\, a\_p3 \tag{53}$$

$$+\frac{71}{512}\,a\_p5+\frac{67095}{212992}\Big)r^2-\frac{7}{2560}\,a\_p5+\frac{7}{2560}\,a\_p4-\frac{19845}{212992}-\frac{7}{2560}\,a\_p3$$
$$-\frac{7}{2560}\,a\_p1+\frac{7}{2560}\,a\_p2\Big)\sin(\phi)\cos(\phi)+\frac{7}{1024}\,\eta\Big)\sqrt{26}\,\sqrt{5}\,\sin(\theta)^2\,r^3$$

#Project B_dot onto boundary conditions

```
> #Define maximum values of m,l,n which are considered
> Mmax := 4;
```

$$Mmax := 4 \qquad (54)$$

```
> Lmax := 10;
```

$$Lmax := 10 \qquad (55)$$

```
> Nmax := 10;
```

$$Nmax := 10 \qquad (56)$$

```
> #initialise poloidal array
> pol_proj := Array(0 .. Lmax, 0 .. Nmax, -Mmax .. Mmax );
```

$$pol\_proj := \left[\begin{array}{c} \text{0..10 x 0..10 x -4..4 Array} \\ \text{Data Type: anything} \\ \text{Storage: rectangular} \\ \text{Order: Fortran\_order} \end{array}\right] \qquad (57)$$

```
> #Project poloidal component
> for m from -Mmax to Mmax do:
   for i from max(1, abs(m) ) to Lmax do:
   a_lm := subs(r=1, (int(int( poloidal_part * L2(i, m) ·sin(theta), theta=0..Pi), phi=0..2
       ·Pi)) / (4 * Pi / (2*i+1)) / ((i+1))) :
   dYlm_dphi := diff(L2(i, m), phi) :
   B_phipart[i, m] := - 1/sin(theta) · a_lm·dYlm_dphi :
   od:
   od:
```

```
> #Recomplie magnetic field phi component time derivative
> B_proj_phi_dot := simplify(add(add(B_phipart[i, m], i=max(1, abs(m))..Lmax), m=-Mmax
       ..Mmax));
```

$$B\_proj\_phi\_dot := -\frac{3}{13}\sqrt{13}\,\sqrt{2}\,\Bigg(\Bigg(\Bigg(\Big(\frac{384}{11}\,a\_p4+\frac{19200}{253}\,a\_p5-\frac{6783}{11264}\,b\_p\Big)\cos(\theta)^8 \qquad (58)$$
$$+\Big(-\frac{32}{3}\,a\_p3-\frac{46336}{627}\,a\_p4-\frac{1815200}{14421}\,a\_p5+\frac{12767}{25344}\,b\_p\Big)\cos(\theta)^6+\Big(\frac{3375}{104}$$
$$+\frac{24}{7}\,a\_p2+\frac{368}{21}\,a\_p3+\frac{3669920}{74613}\,a\_p4+\frac{109668400}{1716099}\,a\_p5-\frac{116035}{354816}\,b\_p\Big)$$

$$\cos(\theta)^4 + \left(-\frac{23085}{1144} - \frac{6}{5} a\_p1 - \frac{312}{77} a\_p2 - \frac{7342}{1001} a\_p3 - \frac{17043616}{1616615} a\_p4 - \frac{75068230}{7436429} a\_p5 - \frac{92321}{295680} b\_p\right) \cos(\theta)^2 - \frac{885}{572} + \frac{31}{35} a\_p1 + \frac{151}{231} a\_p2 + \frac{1399}{3003} a\_p3 + \frac{573863}{1616615} a\_p4 + \frac{1779633}{7436429} a\_p5 - \frac{33535553}{46126080} b\_p + b\_p \ln(2)\Big) \cos(2\phi) + \frac{35}{2} \sin(2\phi)\,\eta\Big) \sin(\theta)\sqrt{5}$$

> *B_sph_dot*[2];

$$-\frac{1063125}{1352}\,\frac{1}{\sin(\theta)\,r}\Bigg(\sqrt{5}\,\Bigg(\frac{52}{14175}\left(r^2 - \frac{21}{25}\right)\sin(\phi)\cos(\phi)\sqrt{r^2\sin(\theta)^2}\;ug\left(\sqrt{r^2\sin(\theta)^2}\right) + \Big(\sin(\phi)\left(\cos(\theta)-1\right)^2\left(r^2 - \frac{7}{45}\right) r^4 \left(\cos(\theta)+1\right)^2\cos(\phi)^5 - \sin(\phi)\left(\cos(\theta)-1\right)^2\left(r^2 - \frac{7}{45}\right) r^4 \left(\cos(\theta)+1\right)^2\cos(\phi)^3 - \frac{52}{3375}\cos(\phi)^2\eta - \frac{7}{72}\sin(\phi)\Big(\left(\left(r^6 - \frac{13}{5} r^4\right)\cos(\theta)^4 + \left(-\frac{24}{7} r^6 + \frac{314}{35} r^4 - \frac{54}{25} r^2\right)\cos(\theta)^2 - \frac{8}{7} r^6 + \frac{92}{35} r^4 - \frac{729}{175} r^2 + \frac{27}{25}\right)\cos(\phi) + \frac{26}{3375}\eta\Big)\left(\cos(\theta)-1\right) r^2 \left(\cos(\theta)+1\right)\Big)\sqrt{26}\,\cos(\theta)\Bigg) \tag{59}$$

> *B_proj_sph_dot*[3] := *B_proj_phi_dot*;

$$B\_proj\_sph\_dot_3 := -\frac{3}{13}\sqrt{13}\sqrt{2}\Bigg(\Bigg(\Bigg(\left(\frac{384}{11} a\_p4 + \frac{19200}{253} a\_p5 - \frac{6783}{11264} b\_p\right)\cos(\theta)^8 + \left(-\frac{32}{3} a\_p3 - \frac{46336}{627} a\_p4 - \frac{1815200}{14421} a\_p5 + \frac{12767}{25344} b\_p\right)\cos(\theta)^6 + \left(\frac{3375}{104} + \frac{24}{7} a\_p2 + \frac{368}{21} a\_p3 + \frac{3669920}{74613} a\_p4 + \frac{109668400}{1716099} a\_p5 - \frac{116035}{354816} b\_p\right)\cos(\theta)^4 + \left(-\frac{23085}{1144} - \frac{6}{5} a\_p1 - \frac{312}{77} a\_p2 - \frac{7342}{1001} a\_p3 - \frac{17043616}{1616615} a\_p4 - \frac{75068230}{7436429} a\_p5 - \frac{92321}{295680} b\_p\right)\cos(\theta)^2 - \frac{885}{572} + \frac{31}{35} a\_p1 + \frac{151}{231} a\_p2 + \frac{1399}{3003} a\_p3 + \frac{573863}{1616615} a\_p4 + \frac{1779633}{7436429} a\_p5 - \frac{33535553}{46126080} b\_p + b\_p \ln(2)\Bigg)\cos(2\phi) + \frac{35}{2}\sin(2\phi)\,\eta\Bigg)\sin(\theta)\sqrt{5} \tag{60}$$

> *#Projected B_dot*

> *B_proj_sph_dot* := *B_sph_dot_approx*[1]·*VectorField*([1, 0, 0]) + *B_sph_dot_approx*[2]·*VectorField*([0, 1, 0]) + *B_proj_sph_dot*[3]·*VectorField*([0, 0, 1]) :

> *B_proj_cyl_dot* := *simplify*(*MapToBasis*(*B_proj_sph_dot*, *cylindrical*[*s*, phi, *z*])) :

```
> SetCoordinates(cylindrical[s, phi, z]);
```
$$cylindrical_{s,\,\phi,\,z} \quad \textbf{(61)}$$

```
> #Construct non-axisymmetric equation
> assume(s > 0);
> interior1 := simplify( (int( int(B_cyl[1]·B_cyl_dot[2] + B_cyl[2]·B_cyl_dot[1], z=-sqrt(1-s^2) ..sqrt(1 - s^2) ), phi = 0 ..2·Pi) ) );
```
$$interior1 := \frac{1}{21632}\left(468000\left(s\sim^4 - \frac{14}{5}\,s\sim^2 + \frac{54}{25}\right)s\sim^2\,\pi\left(\frac{d}{ds\sim}\,ug(s\sim)\right) - 45\,\pi\,(3318750\,s\sim^9 - 11491875\,s\sim^7 + 11940750\,s\sim^5 + 10400\,ug(s\sim)\,s\sim^4 - 3668400\,s\sim^3 - 29120\,ug(s\sim)\,s\sim^2 + 22464\,ug(s\sim))\,s\sim\right)\sqrt{-s\sim^2+1} \quad \textbf{(62)}$$

```
> interior2 := simplify( (1/s) · diff(s^2·interior1, s) );
   #This is the interior term in time differentiated equation
```
$$interior2 := \frac{1}{21632}\,\frac{1}{\sqrt{-s\sim^2+1}}\left(45\,s\sim\,\pi\,(-10400\,s\sim^8 + 39520\,s\sim^6 - 51584\,s\sim^4 + 22464\,s\sim^2)\left(\frac{d^2}{ds\sim^2}\,ug(s\sim)\right) - 3744000\,s\sim^2\,\pi\left(s\sim^6 - \frac{119}{40}\,s\sim^4 + \frac{283}{100}\,s\sim^2 - \frac{81}{100}\right)\left(\frac{d}{ds\sim}\,ug(s\sim)\right) + 45\,\pi\,s\sim\,(83200\,s\sim^6 - 247520\,s\sim^4 + 235456\,s\sim^2 - 67392)\,ug(s\sim) + 1941468750\left(s\sim^8 - \frac{29553}{7670}\,s\sim^6 + \frac{98838}{19175}\,s\sim^4 - \frac{269344}{95875}\,s\sim^2 + \frac{48912}{95875}\right)s\sim^4\,\pi\right) \quad \textbf{(63)}$$

```
> boundary_term1_not_proj := simplify( s/(1 - s^2)^(1/2) int(B_sph[1]·B_sph_dot[3] + B_sph[3]·B_sph_dot[1], phi = 0 ..2·Pi) );
```
$$boundary\_term1\_not\_proj := -\frac{135}{86528}\,\frac{1}{\sqrt{-s\sim^2+1}}\left(s\sim\,\sin(\theta)\left(\int_0^{2\pi}\left(41600\,(r^2\right.\right.\right. \quad \textbf{(64)}$$

$$- \frac{7}{5} \Big) r^2 (\cos(\theta) + 1) \left(\cos(\phi)^2 - \frac{1}{2}\right)^2 \left(r^2 \cos(\theta)^2 + \frac{3}{2} r^2 - \frac{21}{10}\right) (\cos(\theta)$$

$$- 1) \left(\frac{\mathrm{d}}{\mathrm{d}\_x} ug(\_x) \Big|_{\_x = \sqrt{r^2 \sin(\theta)^2}} \right) + 41600 \left(r^2 - \frac{7}{5}\right) \Bigg(\Big(\Big(r^2 \cos(\theta)^2 - \frac{17}{2} r^2$$

$$+ \frac{63}{10}\Big) \cos(\phi)^4 + \left(-r^2 \cos(\theta)^2 + \frac{17}{2} r^2 - \frac{63}{10}\right) \cos(\phi)^2 + \frac{1}{4} r^2 \cos(\theta)^2 - \frac{7}{8} r^2$$

$$+ \frac{21}{40}\Big) \sqrt{r^2 \sin(\theta)^2}\; ug\left(\sqrt{r^2 \sin(\theta)^2}\right) - 75600000 \Big(r^4 (\cos(\theta) + 1)^2 \Big(\Big(\Big(r^4$$

$$- \frac{7}{5} r^2\Big) \cos(\theta)^2 - \frac{7}{18} r^4 + \frac{7}{45} r^2 + \frac{49}{150} \Big) (\cos(\theta) - 1)^2 \cos(\phi)^8$$

$$- 2 r^4 (\cos(\theta) + 1)^2 \left(\left(r^4 - \frac{7}{5} r^2\right) \cos(\theta)^2 - \frac{7}{18} r^4 + \frac{7}{45} r^2 + \frac{49}{150} \right) (\cos(\theta)$$

$$- 1)^2 \cos(\phi)^6 + \Big(\Big(\frac{31}{24} r^8 - \frac{217}{120} r^6 \Big) \cos(\theta)^6 + \Big(\frac{161}{600} r^4 - \frac{233}{72} r^8$$

$$+ \frac{373}{90} r^6 \Big) \cos(\theta)^4 + \left(-\frac{14}{25} r^4 - \frac{147}{1000} r^2 + \frac{167}{72} r^8 - \frac{431}{180} r^6 \right) \cos(\theta)^2 + \frac{441}{2000}$$

$$+ \frac{7}{48} r^8 + \frac{1831}{600} r^4 - \frac{651}{500} r^2 - \frac{119}{60} r^6 \Big) \cos(\phi)^4 + \frac{104}{3375} \Big(r^2$$

$$- \frac{21}{20}\Big) \sin(\phi)\, \eta \cos(\phi)^3 + \Big(\Big(-\frac{7}{24} r^8 + \frac{49}{120} r^6 \Big) \cos(\theta)^6 + \Big(\frac{7}{120} r^4 + \frac{61}{72} r^8$$

$$- \frac{107}{90} r^6 \Big) \cos(\theta)^4 + \left(-\frac{7}{75} r^4 - \frac{13}{24} r^8 + \frac{41}{60} r^6 + \frac{147}{1000} r^2 \right) \cos(\theta)^2 - \frac{441}{2000}$$

$$+ \frac{651}{500} r^2 - \frac{77}{144} r^8 - \frac{109}{40} r^4 + \frac{77}{36} r^6 \Big) \cos(\phi)^2 - \frac{52}{3375} \Big(r^2$$

$$- \frac{21}{20}\Big) \sin(\phi)\, \eta \cos(\phi) + \frac{1}{96} \left(r^2 - \frac{7}{5}\right) \Big(r^6 \cos(\theta)^6 + \left(\frac{1}{4} r^6 - \frac{57}{20} r^4 \right) \cos(\theta)^4$$

$$+ \left(-6 r^6 + \frac{93}{10} r^4 - \frac{63}{50} r^2 \right) \cos(\theta)^2 + 11 r^6 - \frac{111}{5} r^4 + \frac{1233}{100} r^2 - \frac{189}{100} \Big) \Big)$$

$$r^2 (\cos(\theta) + 1) (\cos(\theta) - 1) \Big) \mathrm{d}\phi \Big) \Big)$$

> *boundary_term1_not_proj* := *subs*$\left(\left[r = (s^2 + z^2)^{\frac{1}{2}}, \text{theta} = \arccos\left(\frac{z}{\text{sqrt}(s^2 + z^2)} \right) \right], \right.$
boundary_term1_not_proj$\Big)$;

(65)

$$boundary\_term1\_not\_proj := -\frac{135}{86528}\frac{1}{\sqrt{-s\sim^2+1}}\Bigg(s\sim\sin\left(\arccos\left(\frac{z}{\sqrt{s\sim^2+z^2}}\right)\right)\Bigg(\qquad \textbf{(65)}$$

$$\int_0^{2\pi}\Bigg(41600\left(s\sim^2+z^2-\frac{7}{5}\right)(s\sim^2+z^2)\left(\cos\left(\arccos\left(\frac{z}{\sqrt{s\sim^2+z^2}}\right)\right)+1\right)\left(\cos(\phi)^2\right.$$

$$\left.-\frac{1}{2}\right)^2\left((s\sim^2+z^2)\cos\left(\arccos\left(\frac{z}{\sqrt{s\sim^2+z^2}}\right)\right)^2+\frac{3}{2}s\sim^2+\frac{3}{2}z^2\right.$$

$$\left.-\frac{21}{10}\right)\left(\cos\left(\arccos\left(\frac{z}{\sqrt{s\sim^2+z^2}}\right)\right)-1\right)\left(\frac{d}{d\_x}ug(\_x)\right|$$

$$\_x=\sqrt{(s\sim^2+z^2)\sin\left(\arccos\left(\frac{z}{\sqrt{s\sim^2+z^2}}\right)\right)^2}\Bigg)+41600\left(s\sim^2+z^2-\frac{7}{5}\right)\Bigg(\Bigg((s\sim^2$$

$$+z^2)\cos\left(\arccos\left(\frac{z}{\sqrt{s\sim^2+z^2}}\right)\right)^2-\frac{17}{2}s\sim^2-\frac{17}{2}z^2+\frac{63}{10}\Bigg)\cos(\phi)^4+\Bigg(-(s\sim^2$$

$$+z^2)\cos\left(\arccos\left(\frac{z}{\sqrt{s\sim^2+z^2}}\right)\right)^2+\frac{17}{2}s\sim^2+\frac{17}{2}z^2-\frac{63}{10}\Bigg)\cos(\phi)^2+\frac{1}{4}(s\sim^2$$

$$+z^2)\cos\left(\arccos\left(\frac{z}{\sqrt{s\sim^2+z^2}}\right)\right)^2-\frac{7}{8}s\sim^2-\frac{7}{8}z^2+\frac{21}{40}\Bigg)$$

$$\sqrt{(s\sim^2+z^2)\sin\left(\arccos\left(\frac{z}{\sqrt{s\sim^2+z^2}}\right)\right)^2}\,ug\Bigg($$

$$\sqrt{(s\sim^2+z^2)\sin\left(\arccos\left(\frac{z}{\sqrt{s\sim^2+z^2}}\right)\right)^2}\Bigg)-75600000\Big((s\sim^2$$

$$+z^2)^2 \left(\cos\left(\arccos\left(\frac{z}{\sqrt{s\sim^2+z^2}}\right)\right)+1\right)^2 \left(\left(\left((s\sim^2+z^2)^2-\frac{7}{5}\,s\sim^2\right.\right.\right.$$

$$\left.-\frac{7}{5}\,z^2\right)\cos\left(\arccos\left(\frac{z}{\sqrt{s\sim^2+z^2}}\right)\right)^2-\frac{7}{18}\,(s\sim^2+z^2)^2+\frac{7}{45}\,s\sim^2+\frac{7}{45}\,z^2+\frac{49}{150}\right)$$

$$\left(\cos\left(\arccos\left(\frac{z}{\sqrt{s\sim^2+z^2}}\right)\right)-1\right)^2\cos(\phi)^8-2\,(s\sim^2$$

$$+z^2)^2 \left(\cos\left(\arccos\left(\frac{z}{\sqrt{s\sim^2+z^2}}\right)\right)+1\right)^2 \left(\left(\left((s\sim^2+z^2)^2-\frac{7}{5}\,s\sim^2\right.\right.\right.$$

$$\left.-\frac{7}{5}\,z^2\right)\cos\left(\arccos\left(\frac{z}{\sqrt{s\sim^2+z^2}}\right)\right)^2-\frac{7}{18}\,(s\sim^2+z^2)^2+\frac{7}{45}\,s\sim^2+\frac{7}{45}\,z^2+\frac{49}{150}\right)$$

$$\left(\cos\left(\arccos\left(\frac{z}{\sqrt{s\sim^2+z^2}}\right)\right)-1\right)^2\cos(\phi)^6+\left(\left(\frac{31}{24}\,(s\sim^2+z^2)^4-\frac{217}{120}\,(s\sim^2\right.\right.$$

$$\left.+z^2)^3\right)\cos\left(\arccos\left(\frac{z}{\sqrt{s\sim^2+z^2}}\right)\right)^6+\left(\frac{161}{600}\,(s\sim^2+z^2)^2-\frac{233}{72}\,(s\sim^2+z^2)^4\right.$$

$$\left.+\frac{373}{90}\,(s\sim^2+z^2)^3\right)\cos\left(\arccos\left(\frac{z}{\sqrt{s\sim^2+z^2}}\right)\right)^4+\left(-\frac{14}{25}\,(s\sim^2+z^2)^2-\frac{147}{1000}\,s\sim^2\right.$$

$$\left.-\frac{147}{1000}\,z^2+\frac{167}{72}\,(s\sim^2+z^2)^4-\frac{431}{180}\,(s\sim^2+z^2)^3\right)\cos\left(\arccos\left(\frac{z}{\sqrt{s\sim^2+z^2}}\right)\right)^2$$

$$+\frac{441}{2000}+\frac{7}{48}\,(s\sim^2+z^2)^4+\frac{1831}{600}\,(s\sim^2+z^2)^2-\frac{651}{500}\,s\sim^2-\frac{651}{500}\,z^2-\frac{119}{60}\,(s\sim^2$$

$$\left.+z^2)^3\right)\cos(\phi)^4+\frac{104}{3375}\left(s\sim^2+z^2-\frac{21}{20}\right)\sin(\phi)\,\eta\,\cos(\phi)^3+\left(\left(-\frac{7}{24}\,(s\sim^2+z^2)^4\right.\right.$$

$$\left.+\frac{49}{120}\,(s\sim^2+z^2)^3\right)\cos\left(\arccos\left(\frac{z}{\sqrt{s\sim^2+z^2}}\right)\right)^6+\left(\frac{7}{120}\,(s\sim^2+z^2)^2+\frac{61}{72}\,(s\sim^2\right.$$

$$\left.+z^2)^4-\frac{107}{90}\,(s\sim^2+z^2)^3\right)\cos\left(\arccos\left(\frac{z}{\sqrt{s\sim^2+z^2}}\right)\right)^4+\left(-\frac{7}{75}\,(s\sim^2+z^2)^2\right.$$

$$\left.-\frac{13}{24}\,(s\sim^2+z^2)^4+\frac{41}{60}\,(s\sim^2+z^2)^3+\frac{147}{1000}\,s\sim^2+\frac{147}{1000}\,z^2\right)$$

$$\cos\left(\arccos\left(\frac{z}{\sqrt{s\sim^2+z^2}}\right)\right)^2-\frac{441}{2000}+\frac{651}{500}\,s\sim^2+\frac{651}{500}\,z^2-\frac{77}{144}\,(s\sim^2+z^2)^4$$

$$\left.-\frac{109}{40}\,(s\sim^2+z^2)^2+\frac{77}{36}\,(s\sim^2+z^2)^3\right)\cos(\phi)^2-\frac{52}{3375}\left(s\sim^2+z^2\right.$$

$$- \frac{21}{20} \Big) \sin(\phi)\, \eta \cos(\phi) + \frac{1}{96} \left(s\sim^2 + z^2 - \frac{7}{5} \right) \Bigg(\Big((s\sim^2 + z^2)^3 \cos\left(\arccos\left(\frac{z}{\sqrt{s\sim^2 + z^2}} \right) \right)^6 + \left(\frac{1}{4} (s\sim^2 + z^2)^3 - \frac{57}{20} (s\sim^2 + z^2)^2 \right) \cos\left(\arccos\left(\frac{z}{\sqrt{s\sim^2 + z^2}} \right) \right)^4 + \left(-6 (s\sim^2 + z^2)^3 + \frac{93}{10} (s\sim^2 + z^2)^2 - \frac{63}{50} s\sim^2 - \frac{63}{50} z^2 \right) \cos\left(\arccos\left(\frac{z}{\sqrt{s\sim^2 + z^2}} \right) \right)^2 + 11 (s\sim^2 + z^2)^3 - \frac{111}{5} (s\sim^2 + z^2)^2 + \frac{1233}{100} s\sim^2 + \frac{1233}{100} z^2 - \frac{189}{100} \Big) \Bigg) (s\sim^2 + z^2) \left(\cos\left(\arccos\left(\frac{z}{\sqrt{s\sim^2 + z^2}} \right) \right) + 1 \right) \left(\cos\left(\arccos\left(\frac{z}{\sqrt{s\sim^2 + z^2}} \right) \right) - 1 \right) \Big) \, d\phi \Big) \Big)$$

> $boundary\_term1\_proj := simplify\left(\frac{s}{(1 - s^2)^{\frac{1}{2}}} int(B\_sph[1] \cdot B\_proj\_sph\_dot[3] + B\_sph[3] \cdot B\_proj\_sph\_dot[1], \mathrm{phi} = 0 .. 2 \cdot \mathrm{Pi}) \right);$

$$boundary\_term1\_proj := -\frac{16875}{416} \frac{1}{\sqrt{-s\sim^2 + 1}} \Bigg(\sin(\theta)^3 \pi r \Big(-1024 \left(r^2 - \frac{7}{5} \right) r^{11} \left(r^2 - \frac{21}{25} \right) a\_p5 \cos(\theta)^{10} + 5120 \left(r^2 - \frac{7}{5} \right) \left(r^{13} a\_p5 + \left(\frac{1}{20} a\_p4 - \frac{67}{50} a\_p5 \right) r^{11} + \left(-\frac{21}{500} a\_p4 + \frac{21}{50} a\_p5 \right) r^9 + \frac{6783}{180224000} b\_p - \frac{3}{1375} a\_p4 - \frac{6}{1265} a\_p5 \right) \cos(\theta)^8 - 10240 \left(r^2 - \frac{7}{5} \right) \left(r^{13} a\_p5 + \left(\frac{1}{10} a\_p4 - \frac{46}{25} a\_p5 \right) r^{11} + \left(\frac{1}{160} a\_p3 - \frac{67}{500} a\_p4 + \frac{847}{800} a\_p5 \right) r^9 + \left(-\frac{21}{4000} a\_p3 + \frac{21}{500} a\_p4 - \frac{147}{800} a\_p5 \right) r^7 + \frac{12767}{811008000} b\_p - \frac{1}{3000} a\_p3 - \frac{181}{78375} a\_p4 - \frac{2269}{576840} a\_p5 \right) \cos(\theta)^6 + \Big(10240 r^{15} a\_p5 + \left(1536 a\_p4 - \frac{191488}{5} a\_p5 \right) r^{13} + \left(192 a\_p3 + \frac{1329216}{25} a\_p5 - \frac{124416}{25} a\_p4 \right) r^{11} + \left(16 a\_p2 - \frac{13152}{25} a\_p3 + \frac{695872}{125} a\_p4 - \frac{847904}{25} a\_p5 + \frac{81375}{416} \right) r^9 + \left(\frac{242368}{25} a\_p5 - \frac{896}{25} a\_p2 + \frac{55104}{125} a\_p3 - \frac{315392}{125} a\_p4 - \frac{87465}{208} \right) r^7 + \Big(\frac{9408}{25} a\_p4 - \frac{4704}{5} a\_p5 + \frac{2352}{125} a\_p2 - \frac{14112}{125} a\_p3 \qquad \textbf{(66)}$$

$$+\frac{89523}{416}\Big)r^5+\Big(\frac{23207}{221760}b\_p-\frac{135}{13}-\frac{192}{175}a\_p2-\frac{2944}{525}a\_p3-\frac{5871872}{373065}a\_p4$$
$$-\frac{35093888}{1716099}a\_p5\Big)r^2-\frac{23207}{158400}b\_p+\frac{192}{125}a\_p2+\frac{2944}{375}a\_p3+\frac{5871872}{266475}a\_p4$$
$$+\frac{35093888}{1225785}a\_p5+\frac{189}{13}\Big)\cos(\theta)^4+\Big(-5120\,r^{15}a\_p5+\Big(-1024\,a\_p4$$
$$+\frac{108544}{5}a\_p5\Big)r^{13}+\Big(-192\,a\_p3-\frac{891968}{25}a\_p5+\frac{95744}{25}a\_p4\Big)r^{11}+\Big($$
$$-32\,a\_p2+\frac{15552}{25}a\_p3-\frac{660608}{125}a\_p4+\frac{717376}{25}a\_p5-\frac{55125}{208}\Big)r^9+\Big(-4\,a\_p1$$
$$-\frac{289668}{25}a\_p5+\frac{2192}{25}a\_p2-\frac{86484}{125}a\_p3+\frac{412992}{125}a\_p4+\frac{18585}{26}\Big)r^7+\Big($$
$$-896\,a\_p4+\frac{10528}{5}a\_p5+\frac{224}{25}a\_p1-\frac{9184}{125}a\_p2+\frac{38304}{125}a\_p3-\frac{125685}{208}\Big)r^5$$
$$+\Big(-\frac{588}{125}a\_p1+\frac{2352}{125}a\_p2+\frac{9408}{125}a\_p4+\frac{83349}{520}-\frac{5292}{125}a\_p3$$
$$-\frac{588}{5}a\_p5\Big)r^3+\Big(\frac{48}{125}a\_p1+\frac{92321}{924000}b\_p+\frac{4617}{715}+\frac{2496}{1925}a\_p2+\frac{58736}{25025}a\_p3$$
$$+\frac{136348928}{40415375}a\_p4+\frac{120109168}{37182145}a\_p5\Big)r^2-\frac{92321}{660000}b\_p-\frac{336}{625}a\_p1$$
$$-\frac{2496}{1375}a\_p2-\frac{58736}{17875}a\_p3-\frac{136348928}{28868125}a\_p4-\frac{120109168}{26558675}a\_p5-\frac{32319}{3575}\Big)$$
$$\cos(\theta)^2-\frac{8}{25}\Big(r^2-\frac{7}{5}\Big)b\_p\ln(2)+1024\,r^{15}a\_p5+\Big(256\,a\_p4-\frac{121344}{25}a\_p5\Big)r^{13}$$
$$+\Big(64\,a\_p3+\frac{1147328}{125}a\_p5-\frac{27136}{25}a\_p4\Big)r^{11}+\Big(16\,a\_p2-\frac{5984}{25}a\_p3$$
$$+\frac{220992}{125}a\_p4-\frac{220704}{25}a\_p5-\frac{49875}{416}\Big)r^9+\Big(4\,a\_p1+\frac{113156}{25}a\_p5$$
$$-\frac{1296}{25}a\_p2+\frac{40788}{125}a\_p3-\frac{172864}{125}a\_p4+\frac{93135}{208}\Big)r^7+\Big(\frac{13042}{25}a\_p4$$
$$-\frac{5834}{5}a\_p5-\frac{274}{25}a\_p1+\frac{7082}{125}a\_p2-\frac{24442}{125}a\_p3+b\_p\ln\big(r^2\sin(\theta)^2\big)$$
$$-\frac{255465}{416}\Big)r^5+\Big(\frac{1148}{125}a\_p1-\frac{2912}{125}a\_p2-\frac{9968}{125}a\_p4-\frac{56}{25}b\_p\ln\big(r^2\sin(\theta)^2\big)$$
$$+\frac{23814}{65}+\frac{5852}{125}a\_p3+\frac{3052}{25}a\_p5\Big)r^3+\Big(-\frac{248}{875}a\_p1+\frac{33535553}{144144000}b\_p+\frac{354}{715}$$
$$-\frac{1208}{5775}a\_p2-\frac{11192}{75075}a\_p3-\frac{4590904}{40415375}a\_p4-\frac{14237064}{185910725}a\_p5\Big)r^2$$
$$+\Big(\frac{294}{125}a\_p4-\frac{294}{125}a\_p1+\frac{294}{125}a\_p2-\frac{83349}{1040}+\frac{147}{125}b\_p\ln\big(r^2\sin(\theta)^2\big)$$
$$-\frac{294}{125}a\_p3-\frac{294}{125}a\_p5\Big)r-\frac{33535553}{102960000}b\_p+\frac{248}{625}a\_p1+\frac{1208}{4125}a\_p2$$
$$+\frac{11192}{53625}a\_p3+\frac{4590904}{28868125}a\_p4+\frac{14237064}{132793375}a\_p5-\frac{2478}{3575}\Big)s\sim\Big)$$

> $boundary\_term1\_proj := subs\left(\left[r=(s^2+z^2)^{\frac{1}{2}}, \text{theta}=\arccos\left(\frac{z}{\text{sqrt}(s^2+z^2)}\right)\right], boundary\_term1\_proj\right);$

$$boundary\_term1\_proj := -\frac{16875}{416}\frac{1}{\sqrt{-s\sim^2+1}}\left(\sin\left(\arccos\left(\frac{z}{\sqrt{s\sim^2+z^2}}\right)\right)\right)^3\pi\sqrt{s\sim^2+z^2}\Bigg(\tag{67}$$

$$-1024\left(s\sim^2+z^2-\frac{7}{5}\right)(s\sim^2+z^2)^{11/2}\Big(s\sim^2+z^2$$

$$-\frac{21}{25}\Big)a\_p5\cos\left(\arccos\left(\frac{z}{\sqrt{s\sim^2+z^2}}\right)\right)^{10}+5120\left(s\sim^2+z^2-\frac{7}{5}\right)\Big((s\sim^2+z^2)^{13}$$

$$^{/2}a\_p5+\left(\frac{1}{20}a\_p4-\frac{67}{50}a\_p5\right)(s\sim^2+z^2)^{11/2}+\Big(-\frac{21}{500}a\_p4$$

$$+\frac{21}{50}a\_p5\Big)(s\sim^2+z^2)^{9/2}+\frac{6783}{180224000}b\_p-\frac{3}{1375}a\_p4-\frac{6}{1265}a\_p5\Big)$$

$$\cos\left(\arccos\left(\frac{z}{\sqrt{s\sim^2+z^2}}\right)\right)^8-10240\left(s\sim^2+z^2-\frac{7}{5}\right)\Big((s\sim^2+z^2)^{13/2}a\_p5$$

$$+\left(\frac{1}{10}a\_p4-\frac{46}{25}a\_p5\right)(s\sim^2+z^2)^{11/2}+\Big(\frac{1}{160}a\_p3-\frac{67}{500}a\_p4$$

$$+\frac{847}{800}a\_p5\Big)(s\sim^2+z^2)^{9/2}+\left(-\frac{21}{4000}a\_p3+\frac{21}{500}a\_p4-\frac{147}{800}a\_p5\right)(s\sim^2+z^2)^{7/2}$$

$$+\frac{12767}{811008000}b\_p-\frac{1}{3000}a\_p3-\frac{181}{78375}a\_p4-\frac{2269}{576840}a\_p5\Big)$$

$$\cos\left(\arccos\left(\frac{z}{\sqrt{s\sim^2+z^2}}\right)\right)^6+\Big(10240(s\sim^2+z^2)^{15/2}a\_p5+\Big(1536\,a\_p4$$

$$-\frac{191488}{5}a\_p5\Big)(s\sim^2+z^2)^{13/2}+\Big(192\,a\_p3+\frac{1329216}{25}a\_p5$$

$$-\frac{124416}{25}a\_p4\Big)(s\sim^2+z^2)^{11/2}+\Big(16\,a\_p2-\frac{13152}{25}a\_p3+\frac{695872}{125}a\_p4$$

$$-\frac{847904}{25}a\_p5+\frac{81375}{416}\Big)(s\sim^2+z^2)^{9/2}+\Big(\frac{242368}{25}a\_p5-\frac{896}{25}a\_p2$$

$$+\frac{55104}{125}a\_p3-\frac{315392}{125}a\_p4-\frac{87465}{208}\Big)(s\sim^2+z^2)^{7/2}+\Big(\frac{9408}{25}a\_p4$$

$$- \frac{4704}{5} a\_p5 + \frac{2352}{125} a\_p2 - \frac{14112}{125} a\_p3 + \frac{89523}{416} \Big) \left(s\sim^2 + z^2\right)^{5/2} + \Big(\frac{23207}{221760} b\_p$$

$$- \frac{135}{13} - \frac{192}{175} a\_p2 - \frac{2944}{525} a\_p3 - \frac{5871872}{373065} a\_p4 - \frac{35093888}{1716099} a\_p5 \Big) \left(s\sim^2 + z^2\right)$$

$$- \frac{23207}{158400} b\_p + \frac{192}{125} a\_p2 + \frac{2944}{375} a\_p3 + \frac{5871872}{266475} a\_p4 + \frac{35093888}{1225785} a\_p5$$

$$+ \frac{189}{13} \Big) \cos\left(\arccos\left(\frac{z}{\sqrt{s\sim^2 + z^2}}\right)\right)^4 + \Big(-5120 \left(s\sim^2 + z^2\right)^{15/2} a\_p5 + \Big(-1024 a\_p4$$

$$+ \frac{108544}{5} a\_p5 \Big) \left(s\sim^2 + z^2\right)^{13/2} + \Big(-192 a\_p3 - \frac{891968}{25} a\_p5$$

$$+ \frac{95744}{25} a\_p4 \Big) \left(s\sim^2 + z^2\right)^{11/2} + \Big(-32 a\_p2 + \frac{15552}{25} a\_p3 - \frac{660608}{125} a\_p4$$

$$+ \frac{717376}{25} a\_p5 - \frac{55125}{208} \Big) \left(s\sim^2 + z^2\right)^{9/2} + \Big(-4 a\_p1 - \frac{289668}{25} a\_p5 + \frac{2192}{25} a\_p2$$

$$- \frac{86484}{125} a\_p3 + \frac{412992}{125} a\_p4 + \frac{18585}{26} \Big) \left(s\sim^2 + z^2\right)^{7/2} + \Big(-896 a\_p4$$

$$+ \frac{10528}{5} a\_p5 + \frac{224}{25} a\_p1 - \frac{9184}{125} a\_p2 + \frac{38304}{125} a\_p3 - \frac{125685}{208} \Big) \left(s\sim^2 + z^2\right)^{5/2}$$

$$+ \Big(-\frac{588}{125} a\_p1 + \frac{2352}{125} a\_p2 + \frac{9408}{125} a\_p4 + \frac{83349}{520} - \frac{5292}{125} a\_p3$$

$$- \frac{588}{5} a\_p5 \Big) \left(s\sim^2 + z^2\right)^{3/2} + \Big(\frac{48}{125} a\_p1 + \frac{92321}{924000} b\_p + \frac{4617}{715} + \frac{2496}{1925} a\_p2$$

$$+ \frac{58736}{25025} a\_p3 + \frac{136348928}{40415375} a\_p4 + \frac{120109168}{37182145} a\_p5 \Big) \left(s\sim^2 + z^2\right) - \frac{92321}{660000} b\_p$$

$$- \frac{336}{625} a\_p1 - \frac{2496}{1375} a\_p2 - \frac{58736}{17875} a\_p3 - \frac{136348928}{28868125} a\_p4 - \frac{120109168}{26558675} a\_p5$$

$$- \frac{32319}{3575} \Big) \cos\left(\arccos\left(\frac{z}{\sqrt{s\sim^2 + z^2}}\right)\right)^2 - \frac{8}{25} \left(s\sim^2 + z^2 - \frac{7}{5}\right) b\_p \ln(2)$$

$$+ 1024 \left(s\sim^2 + z^2\right)^{15/2} a\_p5 + \Big(256 a\_p4 - \frac{121344}{25} a\_p5 \Big) \left(s\sim^2 + z^2\right)^{13/2} + \Big(64 a\_p3$$

$$+ \frac{1147328}{125} a\_p5 - \frac{27136}{25} a\_p4 \Big) \left(s\sim^2 + z^2\right)^{11/2} + \Big(16 a\_p2 - \frac{5984}{25} a\_p3$$

$$+\frac{220992}{125}\,a\_p4-\frac{220704}{25}\,a\_p5-\frac{49875}{416}\Big)\,(s\sim^2+z^2)^{9/2}+\Big(4\,a\_p1+\frac{113156}{25}\,a\_p5$$

$$-\frac{1296}{25}\,a\_p2+\frac{40788}{125}\,a\_p3-\frac{172864}{125}\,a\_p4+\frac{93135}{208}\Big)\,(s\sim^2+z^2)^{7/2}$$

$$+\Big(\frac{13042}{25}\,a\_p4-\frac{5834}{5}\,a\_p5-\frac{274}{25}\,a\_p1+\frac{7082}{125}\,a\_p2-\frac{24442}{125}\,a\_p3$$

$$+b\_p\ln\left((s\sim^2+z^2)\sin\left(\arccos\left(\frac{z}{\sqrt{s\sim^2+z^2}}\right)\right)^2\right)-\frac{255465}{416}\Big)\,(s\sim^2+z^2)^{5/2}$$

$$+\Big(\frac{1148}{125}\,a\_p1-\frac{2912}{125}\,a\_p2-\frac{9968}{125}\,a\_p4-\frac{56}{25}\,b\_p\ln\Big((s\sim^2$$

$$+z^2)\sin\left(\arccos\left(\frac{z}{\sqrt{s\sim^2+z^2}}\right)\right)^2\Big)+\frac{23814}{65}+\frac{5852}{125}\,a\_p3+\frac{3052}{25}\,a\_p5\Big)\,(s\sim^2$$

$$+z^2)^{3/2}+\Big(-\frac{248}{875}\,a\_p1+\frac{33535553}{144144000}\,b\_p+\frac{354}{715}-\frac{1208}{5775}\,a\_p2-\frac{11192}{75075}\,a\_p3$$

$$-\frac{4590904}{40415375}\,a\_p4-\frac{14237064}{185910725}\,a\_p5\Big)\,(s\sim^2+z^2)+\Big(\frac{294}{125}\,a\_p4-\frac{294}{125}\,a\_p1$$

$$+\frac{294}{125}\,a\_p2-\frac{83349}{1040}+\frac{147}{125}\,b\_p\ln\left((s\sim^2+z^2)\sin\left(\arccos\left(\frac{z}{\sqrt{s\sim^2+z^2}}\right)\right)^2\right)$$

$$-\frac{294}{125}\,a\_p3-\frac{294}{125}\,a\_p5\Big)\sqrt{s\sim^2+z^2}-\frac{33535553}{102960000}\,b\_p+\frac{248}{625}\,a\_p1+\frac{1208}{4125}\,a\_p2$$

$$+\frac{11192}{53625}\,a\_p3+\frac{4590904}{28868125}\,a\_p4+\frac{14237064}{132793375}\,a\_p5-\frac{2478}{3575}\Big)\,s\sim\Big)$$

> $boundary\_term2\_not\_proj := simplify\Big(subs\Big(z=(1-s^2)^{\frac{1}{2}},\ boundary\_term1\_not\_proj\Big)$
> $\quad +subs\Big(z=-(1-s^2)^{\frac{1}{2}},\ boundary\_term1\_not\_proj\Big)\Big);$

$$boundary\_term2\_not\_proj := \frac{135}{21632}\,\frac{1}{\sqrt{-s\sim^2+1}}\Big(s\sim^3\,\pi\,\Big(78750\,s\sim^7+39375\,s\sim^5 \tag{68}$$

$$+2080\left(\frac{d}{ds\sim}\,ug(s\sim)\right)s\sim^3-2080\,ug(s\sim)\,s\sim^2-151200\,s\sim^3-832\,s\sim\left(\frac{d}{ds\sim}\,ug(s\sim)\right)$$

$$+832\,ug(s\sim)\Big)\Big)$$

> $boundary\_term2\_proj := simplify\Big(subs\Big(z=(1-s^2)^{\frac{1}{2}},\ boundary\_term1\_proj\Big)+subs\Big(z=-(1$

$-s^2)^{\frac{1}{2}}$, *boundary_term1_proj*$\Big)\Big)$;

#This is the boundary term in time differentiated equation

$$boundary\_term2\_proj := \frac{135}{13}\frac{1}{\sqrt{-s{\sim}^2+1}}\Big(s{\sim}^4\Big(b\_p\ln(s{\sim}) - b\_p\ln(2) + 512\,a\_p5\,s{\sim}^{10} + \Big(\frac{1024}{11}a\_p4 - \frac{343040}{253}a\_p5 + \frac{6783}{11264}b\_p\Big)s{\sim}^8 + \Big(\frac{18713920}{14421}a\_p5 - \frac{119296}{627}a\_p4 + \frac{64}{3}a\_p3 - \frac{6035}{3168}b\_p\Big)s{\sim}^6 + \Big(\frac{1125}{26} - \frac{929483200}{1716099}a\_p5 + \frac{9182080}{74613}a\_p4 + \frac{32}{7}a\_p2 - \frac{704}{21}a\_p3 + \frac{53863}{22176}b\_p\Big)s{\sim}^4 + \Big(-\frac{6458}{3465}b\_p - \frac{35055}{572} - \frac{400}{77}a\_p2 + \frac{4}{5}a\_p1 + \frac{41180}{3003}a\_p3 - \frac{127248448}{4849845}a\_p4 + \frac{2089442060}{22309287}a\_p5\Big)s{\sim}^2 + \frac{527833}{360360}b\_p - \frac{24}{35}a\_p1 + \frac{32}{33}a\_p2 - \frac{424}{429}a\_p3 + \frac{20864}{20995}a\_p4 - \frac{489416}{96577}a\_p5 + \frac{5595}{286}\Big)\pi\Big) \quad \textbf{(69)}$$

> *Taylor_equation* := *simplify*(*interior2* + *boundary_term2_not_proj*);
> *#non-projected equation - equivalent to Taylor's ODE*

$$Taylor\_equation := \frac{74671875}{832}\frac{1}{\sqrt{-s{\sim}^2+1}}\Big(s{\sim}\Big(-\frac{16}{66375}s{\sim}^2\Big(s{\sim}^4 - \frac{14}{5}s{\sim}^2 + \frac{54}{25}\Big)\Big(\frac{d^2}{ds{\sim}^2}ug(s{\sim})\Big) + \Big(-\frac{128}{66375}s{\sim}^5 + \frac{1312}{331875}s{\sim}^3 - \frac{288}{184375}s{\sim}\Big)\Big(\frac{d}{ds{\sim}}ug(s{\sim})\Big) + \Big(\frac{128}{66375}s{\sim}^4 - \frac{1312}{331875}s{\sim}^2 + \frac{288}{184375}\Big)ug(s{\sim}) + s{\sim}^3\Big(s{\sim}^6 - \frac{21841}{7670}s{\sim}^4 + \frac{44288}{19175}s{\sim}^2 - \frac{48912}{95875}\Big)\Big)\pi\,(s{\sim}+1)\,(s{\sim}-1)\Big) \quad \textbf{(70)}$$

> *Projection_equation* := *simplify*(*interior2* + *boundary_term2_proj*);*#Projected equation*

$$Projection\_equation := \frac{135}{13}\frac{1}{\sqrt{-s{\sim}^2+1}}\Big(s{\sim}\,\pi\Big(\Big(-\frac{25}{12}s{\sim}^8 + \frac{95}{12}s{\sim}^6 - \frac{31}{3}s{\sim}^4 + \frac{9}{2}s{\sim}^2\Big)\Big(\frac{d^2}{ds{\sim}^2}ug(s{\sim})\Big) + \Big(\frac{27}{2}s{\sim} - \frac{50}{3}s{\sim}^7 + \frac{595}{12}s{\sim}^5 - \frac{283}{6}s{\sim}^3\Big)\Big(\frac{d}{ds{\sim}}ug(s{\sim})\Big) + \Big(-\frac{27}{2} + \frac{50}{3}s{\sim}^6 - \frac{595}{12}s{\sim}^4 + \frac{283}{6}s{\sim}^2\Big)ug(s{\sim}) + s{\sim}^3\Big(b\_p\ln(s{\sim}) - b\_p\ln(2) + 512\,a\_p5\,s{\sim}^{10} + \Big(\frac{1024}{11}a\_p4 - \frac{343040}{253}a\_p5 + \frac{6783}{11264}b\_p + \frac{553125}{64}\Big)s{\sim}^8 + \Big(-\frac{55411875}{1664} + \frac{18713920}{14421}a\_p5 - \frac{119296}{627}a\_p4 \quad \textbf{(71)}$$

$$+\frac{64}{3}\,a\_p3-\frac{6035}{3168}\,b\_p\Big)\,s\sim^6+\Big(\frac{18550125}{416}-\frac{929483200}{1716099}\,a\_p5+\frac{9182080}{74613}\,a\_p4$$
$$+\frac{32}{7}\,a\_p2-\frac{704}{21}\,a\_p3+\frac{53863}{22176}\,b\_p\Big)\,s\sim^4+\Big(-\frac{6458}{3465}\,b\_p-\frac{13923105}{572}$$
$$-\frac{400}{77}\,a\_p2+\frac{4}{5}\,a\_p1+\frac{41180}{3003}\,a\_p3-\frac{127248448}{4849845}\,a\_p4+\frac{2089442060}{22309287}\,a\_p5\Big)\,s\sim^2$$
$$+\frac{2533215}{572}+\frac{527833}{360360}\,b\_p-\frac{24}{35}\,a\_p1+\frac{32}{33}\,a\_p2-\frac{424}{429}\,a\_p3+\frac{20864}{20995}\,a\_p4$$
$$-\frac{489416}{96577}\,a\_p5\Big)\Big)\Big)$$

> **#Numerical Solution**

> $Proj\_chebsum := add(a\_p\|n\cdot s\cdot T(n, 2\,s^2-1), n=1..N);$

$$Proj\_chebsum := a\_p1\,s\sim\,(2\,s\sim^2-1)+a\_p2\,s\sim\,\big(-1+2\,(2\,s\sim^2-1)^2\big)+a\_p3\,s\sim\,\big(4\,(2\,s\sim^2-1)^3-6\,s\sim^2+3\big)+a\_p4\,s\sim\,\big(1+8\,(2\,s\sim^2-1)^4-8\,(2\,s\sim^2-1)^2\big)+a\_p5\,s\sim\,\big(16\,(2\,s\sim^2-1)^5-20\,(2\,s\sim^2-1)^3+10\,s\sim^2-5\big) \quad \textbf{(72)}$$

> $Proj\_numerical\_approx := eval(Proj\_chebsum)+b\_p\cdot s\cdot \ln(s);$

$$Proj\_numerical\_approx := a\_p1\,s\sim\,(2\,s\sim^2-1)+a\_p2\,s\sim\,\big(-1+2\,(2\,s\sim^2-1)^2\big)+a\_p3\,s\sim\,\big(4\,(2\,s\sim^2-1)^3-6\,s\sim^2+3\big)+a\_p4\,s\sim\,\big(1+8\,(2\,s\sim^2-1)^4-8\,(2\,s\sim^2-1)^2\big)+a\_p5\,s\sim\,\big(16\,(2\,s\sim^2-1)^5-20\,(2\,s\sim^2-1)^3+10\,s\sim^2-5\big)+b\_p\,s\sim\,\ln(s\sim) \quad \textbf{(73)}$$

> $Proj\_Residual := simplify(subs(ug(s)=Proj\_numerical\_approx, interior2 + boundary\_term2\_proj));$

$$Proj\_Residual := \frac{135}{13}\,\frac{1}{\sqrt{-s\sim^2+1}}\Big(s\sim^2\,\Big(\ln(s\sim)\,b\_p\,s\sim^2-\ln(2)\,b\_p\,s\sim^2 \quad \textbf{(74)}$$
$$-\frac{608000}{3}\,a\_p5\,s\sim^{16}+\Big(-\frac{108800}{3}\,a\_p4+1062400\,a\_p5\Big)\,s\sim^{14}+\Big(-6000\,a\_p3$$
$$+\frac{515200}{3}\,a\_p4-2270288\,a\_p5\Big)\,s\sim^{12}+\Big(\frac{553125}{64}+\frac{633877360}{253}\,a\_p5$$
$$-\frac{2600}{3}\,a\_p2+25360\,a\_p3-\frac{3542560}{11}\,a\_p4+\frac{6783}{11264}\,b\_p\Big)\,s\sim^{10}+\Big(-\frac{122603}{3}\,a\_p3$$
$$+\frac{186338800}{627}\,a\_p4-\frac{7208868735}{4807}\,a\_p5-\frac{6035}{3168}\,b\_p-\frac{55411875}{1664}-\frac{275}{3}\,a\_p1$$
$$+3220\,a\_p2\Big)\,s\sim^8+\Big(\frac{18550125}{416}+\frac{267325345200}{572033}\,a\_p5-\frac{10217773120}{74613}\,a\_p4$$

$$+\frac{880}{3}\,a\_p1-\frac{30320}{7}\,a\_p2+\frac{634672}{21}\,a\_p3-\frac{361937}{22176}\,b\_p\Big)\,s{\sim}^{6}+\Big(-\frac{13923105}{572}$$

$$-\frac{1457309749190}{22309287}\,a\_p5+\frac{45291779424}{1616615}\,a\_p4+\frac{553816}{231}\,a\_p2-\frac{4678}{15}\,a\_p1$$

$$-\frac{29165998}{3003}\,a\_p3+\frac{385559}{6930}\,b\_p\Big)\,s{\sim}^{4}+\Big(-\frac{20192867}{360360}\,b\_p+\frac{2533215}{572}$$

$$-\frac{14224}{33}\,a\_p2+\frac{3756}{35}\,a\_p1+\frac{416564}{429}\,a\_p3-\frac{36258496}{20995}\,a\_p4$$

$$+\frac{260268484}{96577}\,a\_p5\Big)\,s{\sim}^{2}+18\,b\_p\Big)\,\pi\Big)$$

> *with*(*Student*[*Calculus1*]) :

> $approxProj\_Int\_Res := ApproximateInt\left(Proj\_Residual^2, s=0..1\right)$:

> $\#Proj\_Int\_Res{:=}int\left(Proj\_Residual^2, s=0..1\right)$;

> $Proj\_Int\_Res2 := collect(approxProj\_Int\_Res, seq(a\_p \| i, i=1..N), factor)$;

$$Proj\_Int\_Res2 := \frac{4137102026022700846835603900804097375 3}{3436596411008411710259200000000000000}\,\pi^2\,a\_p1^2 \tag{75}$$

$$-\frac{3}{2336549047965154019301587419136000000000000000000000}\,\pi^2$$

$$\Big(700825395336484283232372107182080000000000\,\ln(20)\,b\_p$$

$$-44238968018699359495077726604266700800000000000\,\ln\left(\frac{3}{20}\right)\,b\_p$$

$$-3161738656856732072231950561341109370880000000000000\,\ln\left(\frac{7}{20}\right)\,b\_p$$

$$-1963214981958270552582818626539526914048000000000000\,\ln\left(\frac{9}{20}\right)\,b\_p$$

$$-7517730210478573785252571523102954323968000000000000\,\ln\left(\frac{11}{20}\right)\,b\_p$$

$$-19463416299346922449558318073534354915328000000000000\,\ln\left(\frac{13}{20}\right)\,b\_p$$

$$-322394591997770220744409930976640000000000000000000000\,\ln\left(\frac{3}{4}\right)\,b\_p$$

$$-89141316111272201299457270225248534855680000000000000\,\ln\left(\frac{17}{20}\right)\,b\_p$$

$$+297978472651581012281947114852683045568512000000000000\,\ln\left(\frac{19}{20}\right)\,b\_p$$

$$-227490947975385512308833923231131191607296000000000000\,b\_p\,\ln(2)$$

$$-22622419410914867449929501767581367102310947589120000\,a\_p2$$

$$+62444334904549889085893017677057977486143578216576000\,a\_p3$$

$+ 185028150823975070442312076399968354489980793069671424\ a\_p4$

$+ 255429862809394907036260403836930672870299784162559424\ a\_p5$

$- 510406060671132018418084278388355092764987638913 7675\ b\_p$

$+ 158999769036412328874032244522341290764725064671250000 \Big)\ a\_p1$

$+ \dfrac{1}{1906349469280233582292211320286877843456000000000000000000000000000}\ \pi^2$

$\Big(6598623101195783505719102025318228147559639313219697376417968750000000$

$- 703323834266254104020592687663452497047552000000000000000000\ \ln(20)\ b\_p$

$+ 420774803870649357084774214227054103408092364800000000000000000\ \ln(3/20)\ b\_p$

$+ 21702126077182640724006857727759794143493104558080000000000000000000\ \ln\left(7/20\right)\ b\_p$

$+ 92429755578460977469404029722187506209791074590720000000000000000\ \ln\left(9/20\right)\ b\_p$

$+ 146362007333701367827401477553291416635740592824320000000000000000\ \ln\left(11/20\right)\ b\_p$

$- 298588531120489917831790775155151888980156821381120000000000000000\ \ln\left(13/20\right)\ b\_p$

$- 20286319560718289848724439800819381833956000000000000000000000000000\ \ln\left(\frac{3}{4}\right)\ b\_p$

$- 342512256738158241636545692142558666750120378437632000000000000000000$

$$\ln\left(\frac{17}{20}\right) b\_p$$

$$+ 1002717396815833970030425269125389733335302749908992000000000000000000$$

$$\ln\left(\frac{19}{20}\right) b\_p$$

$$+ 44080827601720777770467848081343453898665034445836421069952022085632000$$

$$a\_p4^{2}$$

$$+ 128917169200957522574081044785960151325212428823150184166317949545742333\backslash$$

$$6\ a\_p4\ a\_p5$$

$$- 3121945612925489505206696748425585266331968450227634809671408294400000$$

$$a\_p4\ b\_p$$

$$+ 155196878935945547713758255201359965248075106546453296511417802453299200\backslash$$

$$0\ a\_p5^{2}$$

$$- 1891165127893460149143136780118710912419122037370698361264269062112000$$

$$a\_p5\ b\_p$$

$$+ 316221274851407641554359866925199204161170165577352810607463597968750$$

$$b\_p^{2} + 80506413680355328588843482857078784000000000000000000000 \ln(20)^{2}\ b\_p^{2}$$

$$+ 539009794073063204699972977798189940736000000000000000000000 \ln\left(\frac{3}{20}\right)^{2}$$

$$b\_p^{2}$$

$$+ 527570593111907422686931926006752939606016000000000000000000000 \ln\left(\frac{7}{20}\right)^{2}$$

$$b\_p^{2}$$

$$+ 43346373486994569443268977384563146164797440000000000000000000000$$

$$\ln\left(\frac{9}{20}\right)^2 b\_p^2$$

$$+\ 24679744212142580313828055303530646364749824000000000000000000000$$

$$\ln\left(\frac{11}{20}\right)^2 b\_p^2$$

$$+\ 1134326856959463453448559027014513010996674560000000000000000000000$$

$$\ln\left(\frac{13}{20}\right)^2 b\_p^2$$

$$+\ 470430422952160073744842487319402381312000000000000000000000000000$$

$$\ln\left(\frac{3}{4}\right)^2 b\_p^2$$

$$+\ 2018699932408163862091192406258780932770103296000000000000000000000$$

$$\ln\left(\frac{17}{20}\right)^2 b\_p^2$$

$$+\ 13988385000669504589774057725479357131945672704000000000000000000000$$

$$\ln\left(\frac{19}{20}\right)^2 b\_p^2$$

$$+\ 16620791677273702381530353077941869002421698560000000000000000000000$$

$$b\_p^2 \ln(2)^2$$

$$-\ 115029233997022716012383016770014407769080815616000000000000000 \ln(20)\ b\_p^2$$

$$+\ 8033491110867959933311284872603779099847040685670400000000000000 \ln\left(\frac{3}{20}\right)$$

$$b\_p^2$$

$$+\ 1028453135643748308072674771389836064224238371493478400000000000000$$

$$\ln(7/20)\ b\_p^2$$

$$+\ 3765576423608406198624973826723666587647735740991897600000000000000$$

$$\ln(9/20)\ b\_p^2$$

$$-\ 1055141186223814845373863852013505879212032000000000000000000000 \ln(7$$

$/20)\, b\_p^2 \ln(2)$

$- 2797677000133900917954811545095871426389134540800000000000000000000$

$\ln(19/20)\, b\_p^2 \ln(2)$

$+ 16101282736071065717768696571415756800000000000000000000 \ln(20)\, b\_p^2$

$\ln(2)$

$- 4935948842428516062765611060706129272949964800000000000000000000$

$\ln(11/20)\, b\_p^2 \ln(2)$

$+ 68440699213467713072873239610686592029556736000000000000000 \ln(20)\, b\_p$

a_p2

$- 2268653713918926906897118054029026021993349120000000000000000000000$

$\ln(13/20)\, b\_p^2 \ln(2)$

$- 107801958814612640939994595559637988147200000000000000000000 \ln\left(\frac{3}{20}\right)$

$b\_p^2 \ln(2)$

$- 86692746973989138886537954769126292329594880000000000000000000000 \ln(9$

$/20)\, b\_p^2 \ln(2)$

$- 940860845904320147489684974638804762624000000000000000000000000000$

$\ln(3/4)\, b\_p^2 \ln(2)$

$- 40373998648163277241823848125175618655402065920000000000000000000000$

$\ln(17/20)\, b\_p^2 \ln(2)$

$- 15246613611310280220585100354764782606179368960000000000000000 \ln(20)\, b\_p$

a_p3

$+ 266929711198289553878488260475290914957082545356800000000000 \ln(20)\, b\_p$

a_p4

$- 4080906015905258561475727095839818064146243308748800000000000 \ln(20)\ b\_p$

a_p5

$- 4088319391045319477018104027795993083323130839040000000000000000 \ln\left(\frac{3}{20}\right)$

$b\_p\ a\_p2$

$+ 8271927621370064686053146701394209028985638092800000000000000000 \ln\left(\frac{3}{20}\right)$

$b\_p\ a\_p3$

$- 1253370951081336607369082059064713581841112120898355200000000000 \ln(3$

$/20)\ b\_p\ a\_p4$

$+ 1558056398867248719730313309249190167237124507020820480000000000 \ln(3$

$/20)\ b\_p\ a\_p5$

$- 207456001069879272045823431435619116381762050064384000000000000000$

$\ln(7/20)\ b\_p\ a\_p2$

$+ 174311583566906590563483893747441252619426849619968000000000000000$

$\ln(7/20)\ b\_p\ a\_p3$

$+ 135485854269016433255749774597216417788718983211175116800000000000$

$\ln(7/20)\ b\_p\ a\_p4$

$- 576149716417245896626942353368603881753118628116976107520000000000$

$\ln(7/20)\ b\_p\ a\_p5$

$- 848523288713342310186278494874224055410081709162496000000000000000$

$\ln(9/20)\ b\_p\ a\_p2$

$- 478296567863838374755739625047650722630645422909030400000000000000$

$\ln\left(9/20\right) b\_p\, a\_p3$

$+ 27136990244199632702391040833855210940612827819144118272000000000000$

$\ln\left(9/20\right) b\_p\, a\_p4$

$- 27069486789651397884259083145893323617283606872138658611200000000000$

$\ln\left(9/20\right) b\_p\, a\_p5$

$- 10006712821574677631523732993315753466194894268661760000000000000000$

$\ln\left(11/20\right) b\_p\, a\_p2$

$- 66882774157277642125087032312421382548606951624802304000000000000000$

$\ln\left(11/20\right) b\_p\, a\_p3$

$+ 75891214412998261444600810935474903226793349367612833792000000000000$

$\ln\left(11/20\right) b\_p\, a\_p4$

$+ 89791276485678651732859639587470972281349586696534805708800000000000$

$\ln\left(11/20\right) b\_p\, a\_p5$

$+ 54328428371417753122631768491760379304917369366773760000000000000000$

$\ln\left(13/20\right) b\_p\, a\_p2$

$+ 149418020061470185558065314923072179677017927275781185798144000000000$

a_p2^2

$+ 184974402493122219503146128797841625721977060833570568724152320000000$

$a\_p2\, a\_p3$

$- 852056367881385108426467209472086419525996380229225478598610124800000$

$a\_p2\, a\_p4$

$- 1750415325190937314543399672432141342341959452237160086888969863168000$

$a\_p2\ a\_p5$

$+ 30368535152694655177549906334925129989385997211896537402924800000000$

$a\_p2\ b\_p$

$+ 7934500099525392328714205387433388459084899988221828165763645440000000$

a_p3^2

$+ 201290213561669706865896414413828893946358182617240553284643625369600000$

$a\_p3\ a\_p4$

$+ 524371233700058743781527015065772404439616608091499205141867693670400 0$

$a\_p3\ a\_p5$

$- 332807487438887887387677191443510423360483729897401676339692192000000$

$a\_p3\ b\_p$

$+ 92586819729265444010018590037718817958785301124243456000000000000000$

$\ln\left(11/20\right) b\_p^2$

$+ 1596380516082188007544006944528103623455887273242378240000000000000$

$\ln\left(13/20\right) b\_p^2$

$+ 1709778288904356131621668419998202017401500000000000000000000000000$

$\ln\left(3/4\right) b\_p^2$

$+ 525962759348160099992830906115712693118872868184064000000000000000$

$\ln\left(17/20\right) b\_p^2$

$- 485295634914783402578390898892424334691074233230270464000000000000000$

$\ln\left(19/20\right) b\_p^2$

$+ 882071431979813152260955460282532037107935130107904000000000000000\ b\_p^2$

$$\ln(2)$$

$$-4541537468931803752031075369310481879204437445017600000000000000000000$$

$$b\_p \ln(2)$$

$$-2096599664588262549662856967425876099182359362718924800000000000000000$$

$$\ln\left(\frac{13}{20}\right) b\_p\ a\_p3$$

$$-1765057520492108374667075119977621022154303467344856350720000000000000$$

$$\ln\left(\frac{13}{20}\right) b\_p\ a\_p4$$

$$+5443119762035697917352241743739168464590397647055747022848000000000000$$

$$\ln\left(\frac{13}{20}\right) b\_p\ a\_p5$$

$$+3140860855293375969855038112384766049894400000000000000000000000000000$$

$$\ln\left(\frac{3}{4}\right) b\_p\ a\_p2$$

$$-2026298733843659478103627037006439744000000000000000000000000000000000$$

$$\ln\left(\frac{3}{4}\right) b\_p\ a\_p3$$

$$-1160036758358208469154487799424023606895616000000000000000000000000000$$

$$\ln\left(\frac{3}{4}\right) b\_p\ a\_p4$$

$$-6661612200324816613235539731997193906160000000000000000000000000000000$$

$$\ln\left(\frac{3}{4}\right) b\_p\ a\_p5$$

$$+7418042534127125928334042518363464313652736450730393600000000000000000$$

$$\ln\left(\frac{17}{20}\right) b\_p\ a\_p2$$

$$+1861173786450294423554967935800759816843334055006044160000000000000000$$

$$\ln\left(\frac{17}{20}\right) b\_p\ a\_p3$$

$$+4181195206463480206437662106390416914088183780978070650880000000000000$$

$\ln\left(\frac{17}{20}\right) b\_p\ a\_p4$

$- 4554037903343553805312692712854483646027038030061597045555200000000000$

$\ln\left(\frac{17}{20}\right) b\_p\ a\_p5$

$- 9109451399088929893450307536515298715784816545601945600000000000000000$

$\ln\left(\frac{19}{20}\right) b\_p\ a\_p2$

$+ 3496673796660820056122936792925501201374983370462724096000000000000000$

$\ln\left(\frac{19}{20}\right) b\_p\ a\_p3$

$+ 1399463100034622128069562220106606712584594880748785447731200000000000$

$\ln\left(\frac{19}{20}\right) b\_p\ a\_p4$

$+ 2582858262896481257595451119726704985621837880035788067962880000000000$

$\ln\left(\frac{19}{20}\right) b\_p\ a\_p5$

$- 1781060943121011239802942077004698450916147845922816000000000000000000\ b\_p \ln(2)\ a\_p2$

$- 5058958791062684545756787012630101620918885598095212544000000000000000\ b\_p \ln(2)\ a\_p3$

$- 1317968893413496295704571397036887025700637944517801017344000000000000\ b\_p \ln(2)\ a\_p4$

$- 2120988764518626406263055936164537810228422976393639823933440000000000\ b\_p \ln(2)\ a\_p5$

$- 1873604968529371254569482235791359193630735430702385608352000000000000\ a\_p2$

$- 2094695818115999043510990974835530625724695970480252001102400000000000\ a\_p3$

$+ 7927255103010284288007177393374026476755858306930567512434988800000000\ a\_p4$

$+ 1370256153488125729254186472423180927797573049361205721921173729600000\backslash$
$0\ a\_p5$

$+ 1859626543537510111058640612731504126155128167760096758443359375000000\ b\_p)$

> *Proj_Min_Int_Res2* := *evalf*(*minimize*(*Proj_Int_Res2*, *seq*(*a_p* || *i*, *i* = 1 ..*N*), *b_p*, *location*));

Proj_Min_Int_Res2 := 30.18186043498688347594519101 20, {[{*a_p1* = -7.4803318263837783290307290 1222, *a_p2* = 7.4234013745754694295535944 1819, **(76)**

$a\_p3 = -1.1459072122150724609670653200 7, a\_p4 =$
$-0.182455191162990329261343966310, a\_p5 =$
$-0.0718897731075194738196217630786, b\_p = 1.16425433583241005999153448444\},$
$30.1818604349868834759451910120]\}$

> $Proj\_Solutionarray := \{op(op(op(Proj\_Min\_Int\_Res2[2]))[1])\};$

$Proj\_Solutionarray := \{a\_p1 = -7.48033182638377832903072901222, a\_p2 = 7.42340137457546942955359441819, a\_p3 = -1.14590721221507246096706532007, a\_p4 = -0.182455191162990329261343966310, a\_p5 = -0.0718897731075194738196217630786, b\_p = 1.16425433583241005999153448444\}$ **(77)**

```
> vars_p := [seq( a_p||i,i=1..N),b_p];
```

$vars\_p := [a\_p1, a\_p2, a\_p3, a\_p4, a\_p5, b\_p]$ **(78)**

> $Proj\_Solutionarray2 := evalf(subs(Proj\_Solutionarray, vars\_p));$

$Proj\_Solutionarray2 := [-7.48033182638377832903072901222, 7.42340137457546942955359441819, -1.14590721221507246096706532007, -0.182455191162990329261343966310, -0.0718897731075194738196217630786, 1.16425433583241005999153448444]$ **(79)**

> *# calculate constant so angular momentum is zero*

> $Proj\_numerical\_app := Proj\_numerical\_approx + s \cdot Cst :$

> $Proj\_ang\_mom := evalf(int(int(int(expand(Proj\_numerical\_app * s^2), \mathrm{phi} = 0 .. 2 * \mathrm{Pi}), z = -\mathrm{sqrt}(1 - s\text{^}2) .. \mathrm{sqrt}(1 - s\text{^}2)), s = 0 .. 1)) :$

> **for** i **from** 1 **to** N **do** $a\_p \| i := (Proj\_Solutionarray2[i])$ **end do**:

> $b\_p := rhs(Proj\_Solutionarray[M]);$

$b\_p := 1.16425433583241005999153448444$ **(80)**

```
> Proj_C_value := solve(Proj_ang_mom = 0):
```

> *Proj_numerical_solution* ≔ *subs*(*Cst* = *Proj_C_value*, *Proj_numerical_app*) :

> *#Plot geostrophic flow solution*

> *plot*([*Proj_numerical_solution*], *s* = 0 ..1);

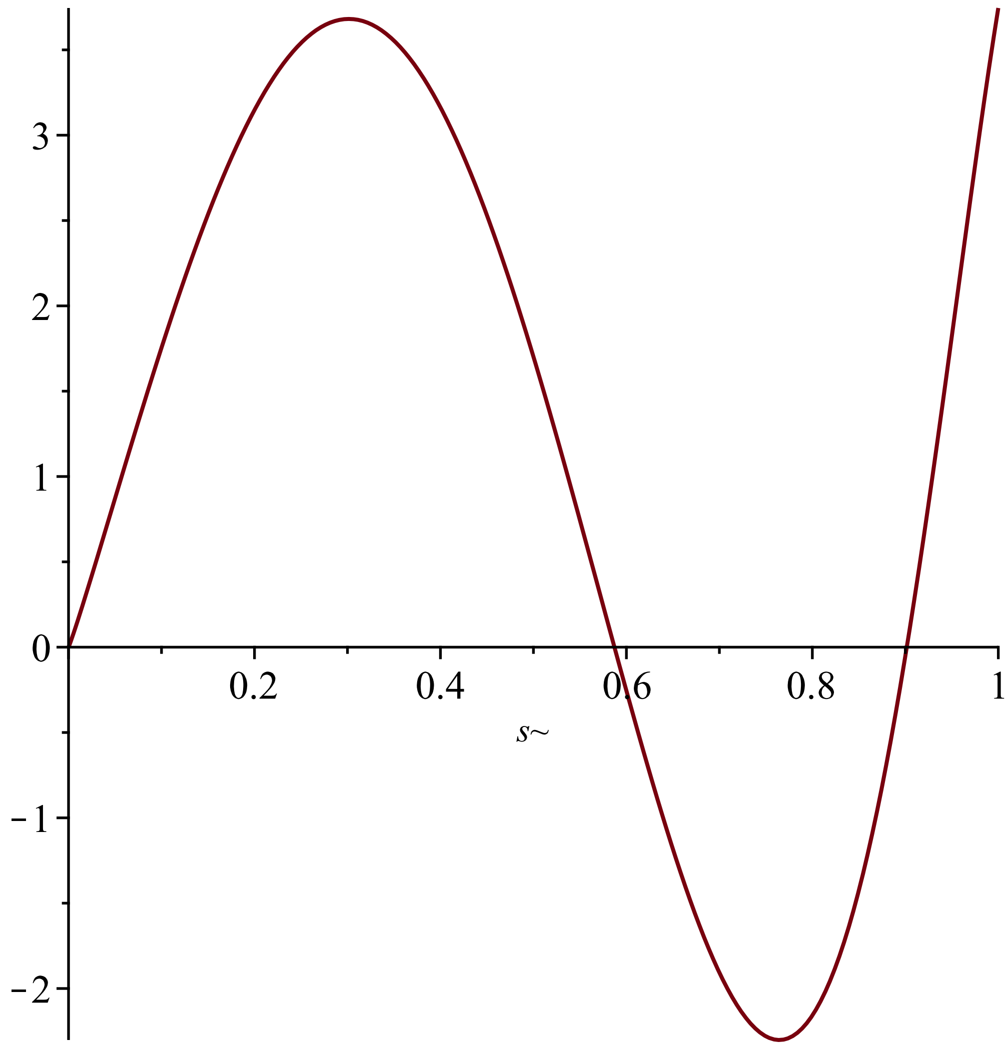

Supplement: ESM5 - ESM8 are PDF versions of the maple worksheets ESM1 - ESM4 respectively. [file rspa20180412supp4.pdf]
