# Supplementary material for: Determination of the instantaneous geostrophic flow within the three-dimensional magnetostrophic regime
Source: Proc Math Phys Eng Sci. 2018 Oct 3;474(2218):20180412. doi: 10.1098/rspa.2018.0412 (PMC6237501; doi:10.1098/rspa.2018.0412)

This worksheet uses the implicit timestep method to determine the geostrophic flow for all initial magnetic fields including mixed states and non-axisymmetric fields. The method consists of discretising the induction equation and using a finite time step to evolve the magnetic field according to this. Choosing a very small timestep gives an approximate solution for the instantaneous geostrophic flow.

```
> restart:
> Digits := 10:
> with(orthopoly, P):
  with(VectorCalculus):
  SetCoordinates(cartesian[x,y,z]):
```

> # Define some useful routines

```
> # L1 is theta-factor in spherical harmonic (note that P(l,x) is
     the l-th Legendre polynomial)
> L1 := (l,m) -> if type(m, numeric) then if m <> 0 then sin(theta)
  ^abs(m) * subs(z=cos(theta), diff(P(l,z),z$abs(m))) else P(l, cos
  (theta)) end if else 'L1'(l,m) end if;
```

$$L1 := (l, m) \rightarrow \textbf{if } type(m, numeric) \textbf{ then if } m \neq 0 \textbf{ then } \sin(\theta)^{|m|} subs\left(z = \cos(\theta), \frac{\partial^{|m|}}{\partial z^{|m|}} P(l, z)\right) \textbf{ else } P(l, \cos(\theta)) \textbf{ end if else } 'L1'(l, m) \textbf{ end if} \quad (1)$$

```
> # Lp is phi-factor
> Lp := m -> if type(m, numeric) then if m = 0 then 1 elif m < 0
  then sin(-m*phi) else cos(m*phi) end if else 'Lp'(m) end if;
```

$$Lp := m \rightarrow \textbf{if } type(m, numeric) \textbf{ then if } m = 0 \textbf{ then } 1 \textbf{ elif } m < 0 \textbf{ then } \sin(VectorCalculus\text{:-`-`}(m\,\phi)) \textbf{ else } \cos(m\,\phi) \textbf{ end if else } 'Lp'(m) \textbf{ end if} \quad (2)$$

```
> # norm of L1*Lp is integral of (L1*Lp)^2 over sphere is int((L1*
  Lp)^2 * sin(theta), theta=0..Pi, phi=0..2*Pi)
> L2norm_squared := (l,m) -> int(L1(l,m)^2 * sin(theta), theta=0..
  Pi) * int(Lp(m)^2, phi=0..2*Pi) / (4*Pi);
```

$$L2norm\_squared := (l, m) \rightarrow VectorCalculus\text{:-}int\left(L1(l, m)^2 \sin(\theta), \theta = 0..\pi\right) VectorCalculus\text{:-}int\left(Lp(m)^2, \phi = 0..2\,\pi\right) \frac{1}{4\,\pi} \quad (3)$$

```
> # L2 is Schmidt quasi-normalised spherical harmonic
> L2 := (l,m) -> if type(l,numeric) and type(m,numeric) then L1(l,
  m) * Lp(m) / sqrt(L2norm_squared(l,m)) / sqrt(2*l+1) else 'L2'(l,
  m) end if;
```

$$L2 := (l, m) \rightarrow \textbf{if } type(l, numeric) \textbf{ and } type(m, numeric) \textbf{ then } L1(l, m)\, Lp(m) \frac{1}{\sqrt{L2norm\_squared(l, m)}} \frac{1}{\sqrt{2\,l + 1}} \textbf{ else } 'L2'(l, m) \textbf{ end if} \quad (4)$$

```
> # Convert an expression in spherical coordinates to Cartesian
  coordinates.
```

```
> sph2cart := proc(expr)
    local res;
    res := expand(expr, trig);
    res := subs(cos(phi) = x/(r*sin(theta)), sin(phi) = y/(r*sin
  (theta)), res);
    res := subs(cos(theta) = z/r, sin(theta) = sqrt(x^2+y^2)/r,
  res);
    res := subs(r = sqrt(x^2+y^2+z^2), res);
    return simplify(res)
  end proc:
> # Construct vector field from poloidal and toroidal scalars
  scalars2vf := proc(tor_scalar, pol_scalar)
    return simplify(Curl(VectorField(sph2cart(tor_scalar/r) * <x,
  y,z>))
                    + Curl(Curl(VectorField(sph2cart(pol_scalar/r)
  * <x,y,z>))))
  end proc:
```

> *# Define a basis function for the poloidal magnetic field satisfying insulating BCs:*

```
> Psi_n := (l, n) → r^(l+1) * ( (-2*n^2*(l+1) - n*(l+1)*(2*l-1)-l*(2*l+1))
      * P(n, 0, l+1/2, 2*r^2-1) + (2*(l+1)*n^2 + (2*l+3)*(l+1)*n + (2*l+1)
      ^2)*P(n-1, 0, l+1/2, 2*r^2-1) + (4*n*l+l*(2*l+1)) );
```

$$Psi\_n := (l, n) \rightarrow r^{l+1} \Big(\big(VectorCalculus{:-}\text{`-`}\big(2\, n^2\, (l+1)\big) + VectorCalculus{:-}\text{`-`}\big(n\, (l+1)\, (2\, l + (-1))\big) + VectorCalculus{:-}\text{`-`}\big(l\, (2\, l+1)\big)\big)\, P\Big(n, 0, l+1\,\frac{1}{2}, 2\, r^2 + (-1)\Big) + \big(2\, (l+1)\, n^2 + (2\, l+3)\, (l+1)\, n + (2\, l+1)^2\big)\, P\Big(n + (-1), 0, l+1\,\frac{1}{2}, 2\, r^2 + (-1)\Big) + 4\, n\, l + l\, (2\, l+1)\Big) \quad \textbf{(5)}$$

> *# Define basis functions for the poloidal flow which vanish at r=1.*

```
> Chi_n := (l, n) → r^(l+1) * (1-r^2) * P(n-1, 2, l+1/2, 2*r^2-1);
```

$$Chi\_n := (l, n) \rightarrow r^{l+1} \big(1 + VectorCalculus{:-}\text{`-`}(r^2)\big)\, P\Big(n + (-1), 2, l+1\,\frac{1}{2}, 2\, r^2 + (-1)\Big) \quad \textbf{(6)}$$

```
> W_n := (l, n) → r^(l+1) * P(n, 0, l+1/2, 2*r^2-1) :
```

> # Define magnetic field

```
> # choose a number corresponding to the chosen magnetic field 1=
  axisymmetric poloidal, 2=nonaxisymmetric toroidal, 3=
  nonaxisymmetric poloidal, 4=nonaxisymmetric mixed state
> k := 3;
```

$$k := 3 \quad \textbf{(7)}$$

```
> if k = 1 then B_scalar_tor := 0 : B_scalar_pol := eval( r^2 * (30*r^4 − 57*r^2 + 25)
      * L2(l, m), {l = 1, m = 0, n = 1})
  end if

> if k = 2 then B_scalar_tor := simplify(eval(Chi_n(l, n)·L2(l, m), {l = 1, m = 1, n = 1})) :
```

```
        B_scalar_pol := 0
    end if
> if k = 3 then B_scalar_tor := 0 : B_scalar_pol := eval(Psi_n(l, n)·L2(l, m), {l = 2, m = 2, n
        = 1})
    end
```

$$B\_scalar\_tor := 0$$

$$B\_scalar\_pol := \frac{1}{10}\, r^3 \left(\frac{315}{2} - \frac{225}{2}\, r^2\right) \sin(\theta)^2 \cos(2\,\phi)\, \sqrt{15}\, \sqrt{5} \qquad \textbf{(8)}$$

```
> if k = 4 then B_scalar_tor := simplify(eval( Chi_n(l, n)·L2(l, m), {l = 2, m = 1, n = 1})) :
        B_scalar_pol := eval(Psi_n(l, n)·L2(l, m) + Psi_n(l + 2, n)·L2(l + 2, m), {l = 2, m = 1, n
        = 1})
    end if
> #B_scalar_tor :=0;
```

```
> B_cart_pol := scalars2vf(0, B_scalar_pol);
```

$$B\_cart\_pol := \left(\frac{945}{2}\sqrt{3}\,x - \frac{675}{2}\sqrt{3}\,x^3 - \frac{1125}{2}\sqrt{3}\,x\,z^2 - \frac{1575}{2}\,x\sqrt{3}\,y^2\right)\bar{e}_x + \left(\frac{1125}{2}\sqrt{3}\,y\,z^2 - \frac{945}{2}\sqrt{3}\,y + \frac{675}{2}\sqrt{3}\,y^3 + \frac{1575}{2}\,y\sqrt{3}\,x^2\right)\bar{e}_y + \left(225\sqrt{3}\,x^2 z - 225\sqrt{3}\,y^2 z\right)\bar{e}_z \qquad \textbf{(9)}$$

```
> B_cart_tor := scalars2vf(B_scalar_tor, 0);
```

$$B\_cart\_tor := 0\bar{e}_x \qquad \textbf{(10)}$$

```
> B_sph_pol := simplify(MapToBasis(B_cart_pol, spherical[r, theta, phi]));
```

$$B\_sph\_pol := -\frac{135}{2}\sin(\theta)^2\left(5\,r^2 - 7\right)\left(2\cos(\phi)^2 - 1\right)\sqrt{3}\,r\bar{e}_r - \frac{45}{2}\cos(\theta)\sqrt{3}\,r\sin(\theta)\left(25\,r^2 - 21\right)\left(2\cos(\phi)^2 - 1\right)\bar{e}_\theta + \left(\left(1125\,r^3 - 945\,r\right)\sin(\phi)\cos(\phi)\sin(\theta)\sqrt{3}\right)\bar{e}_\phi \qquad \textbf{(11)}$$

```
> B_sph_tor := simplify(MapToBasis(B_cart_tor, spherical[r, theta, phi]));
```

$$B\_sph\_tor := 0\bar{e}_r \qquad \textbf{(12)}$$

```
> SetCoordinates(spherical[r, theta, phi]);
```

$$\mathrm{spherical}_{r,\,\theta,\,\phi} \qquad \textbf{(13)}$$

```
> #Scale the magnetic field
```

```
> Scale_pol := sqrt( 1/(4·Pi) · int(int(int(A_pol·B_sph_pol.B_sph_pol·r^2·sin(theta), phi = 0..2
        · Pi), theta = 0.. Pi), r = 0..1) );
```

$$Scale\_pol := 6\sqrt{390}\,\sqrt{A\_pol} \qquad \textbf{(14)}$$

```
> if Scale_pol = 0 then A_pol = 0
  else A_pol := simplify(solve(Scale_pol = 1, A_pol)); end if;
```

$$A\_pol := \frac{1}{14040} \quad (15)$$

> $Scale\_tor := \mathrm{sqrt}\left(\frac{1}{4\cdot \mathrm{Pi}} \cdot int(int(int(A\_tor\cdot B\_sph\_tor.B\_sph\_tor\cdot \sin(\mathrm{theta}), \mathrm{phi}=0..2\cdot \mathrm{Pi}), \mathrm{theta}=0..\mathrm{Pi}), r=0..1)\right);$

$$Scale\_tor := 0 \quad (16)$$

> **if** $Scale\_tor=0$ **then** $A\_tor=0$
> **else** $A\_tor := simplify(solve(Scale\_tor=1, A\_tor))$ **end if**;

$$A\_tor=0 \quad (17)$$

> $SetCoordinates(cartesian[x, y, z])$:

> $B\_cart := scalars2vf(\mathrm{sqrt}(A\_tor)\cdot B\_scalar\_tor, \mathrm{sqrt}(A\_pol)\cdot B\_scalar\_pol);$

$$B\_cart := \left(\frac{63}{104}\sqrt{5}\sqrt{26}\,x - \frac{45}{104}\sqrt{5}\sqrt{26}\,x^3 - \frac{75}{104}\sqrt{5}\sqrt{26}\,x z^2 - \frac{105}{104}\,x\sqrt{5}\sqrt{26}\,y^2\right)\bar{e}_x + \left(\frac{75}{104}\sqrt{5}\sqrt{26}\,y z^2 - \frac{63}{104}\sqrt{5}\sqrt{26}\,y + \frac{45}{104}\sqrt{5}\sqrt{26}\,y^3 + \frac{105}{104}\,y\sqrt{5}\sqrt{26}\,x^2\right)\bar{e}_y + \left(\frac{15}{52}\sqrt{5}\sqrt{26}\,x^2 z - \frac{15}{52}\sqrt{5}\sqrt{26}\,y^2 z\right)\bar{e}_z \quad (18)$$

> **B_sph := simplify(MapToBasis(B_cart, spherical[r,theta,phi]));**

$$B\_sph := -\frac{9}{104}\sin(\theta)^2(5r^2-7)(2\cos(\phi)^2-1)\sqrt{5}\sqrt{26}\,r\bar{e}_r - \frac{3}{104}\cos(\theta)\sqrt{5}\sqrt{26}\,r\sin(\theta)(25r^2-21)(2\cos(\phi)^2-1)\bar{e}_\theta + \frac{75}{52}\,r\sin(\theta)\cos(\phi)\sqrt{26}\left(r^2-\frac{21}{25}\right)\sin(\phi)\sqrt{5}\,\bar{e}_\phi \quad (19)$$

> # **Compute rhs of magnetostrophic equation**

```
> # slaved equation is Omega cross u = -div(p) + curl(B) cross B,
  we ignore the pressure
> RHS := CrossProduct(Curl(B_cart), B_cart): simplify(RHS);
```

$$-\frac{1575}{104} x \left(35 x^2 y^2 + 5 x^2 z^2 + 15 y^4 + 20 y^2 z^2 - 21 y^2\right)\bar{e}_x - \frac{1575}{104} y \left(15 x^4 + 35 x^2 y^2 + 20 x^2 z^2 + 5 y^2 z^2 - 21 x^2\right)\bar{e}_y - \frac{1575}{208} z \left(15 x^4 + 70 x^2 y^2 + 25 x^2 z^2 + 15 y^4 + 25 y^2 z^2 - 21 x^2 - 21 y^2\right)\bar{e}_z \quad (20)$$

```
> map(factor, simplify(MapToBasis(RHS, spherical[r,theta,phi])));
  # for comparison
```

$$-\frac{1575}{208} \sin(\theta)^2 \left(25 r^2 - 21\right) \left(4 \cos(\phi)^4 \cos(\theta)^2 - 4 \cos(\phi)^4 - 4 \cos(\phi)^2 \cos(\theta)^2 + 4 \cos(\phi)^2 + \cos(\theta)^2\right) r^3 \bar{e}_r + \frac{4725}{208} \cos(\theta) \sin(\theta)^3 \left(5 r^2 - 7\right) \left(2 \cos(\phi)^2 - 1\right)^2 r^3 \bar{e}_\theta - \frac{4725}{104} \sin(\phi) \sin(\theta)^3 \cos(\phi) \left(5 r^2 - 7\right) \left(2 \cos(\phi)^2 - 1\right) r^3 \bar{e}_\phi \quad (21)$$

> # Construct basis for u

```
> # Let N be the degree of B. Then the degree of curl(B) cross B is
  (N-1) + N = 2N-1. Thus the degree of u is also 2N-1. Thus, the
  poloidal scalar has degree 2N+1 (we have to undo two curls) and
  the toroidal scalar has degree 2N. Furthermore, the m-degree of
  curl(B) cross B is twice the m-degree of B.
> B_degree := max(seq(degree(B_cart[idx], {x,y,z}), idx = 1 .. 3));
```

$$B\_degree := 3 \quad (22)$$

```
> Max_pol_degree := 2 * B_degree + 1; Max_tor_degree := 2 *
  B_degree;
```

$$Max\_pol\_degree := 7$$
$$Max\_tor\_degree := 6 \quad (23)$$

```
> Max_m_degree := 2 * max(seq(degree(expand(B_sph[idx], trig), {cos
  (phi), sin(phi)}), idx = 1 .. 3));
```

$$Max\_m\_degree := 4 \quad (24)$$

```
> # For the particular example here, certain modes are zero, but we
  will not exploit this knowledge.
> # Also note that unless B is a Taylor state, there will be no
  solution to the magnetostrophic equation.

> u_scalar_pol := add(add(add(S[l,m,n] * L2(l,m) * Chi_n(l,n),
                              m = -min(l, Max_m_degree) .. min(l,
  Max_m_degree)),
                               n = 1 .. (Max_pol_degree - l + 1) /
  2),
                               l = 1 .. Max_pol_degree):
> u_scalar_tor := add(add(add(T[l,m,n] * L2(l,m) * W_n(l,n),
                              m = -min(l, Max_m_degree) .. min(l,
  Max_m_degree)),
                              n = 0 .. floor((Max_tor_degree - l +
  1)/2)),
```

```
                    l = 1 .. Max_tor_degree):
> coeff(u_scalar_pol, S[2,-2,2]);
```
$$\frac{1}{10}\sin(\theta)^2\sin(2\phi)\sqrt{15}\sqrt{5}\,r^3\left(-r^2+1\right)\left(-\frac{7}{2}+\frac{13}{2}r^2\right) \quad (25)$$

```
> # u is poloidal part (curl^2 of scalar times hat r) + toroidal
  part (curl of scalar times hat r)
> u_cart := scalars2vf(u_scalar_tor, u_scalar_pol):
> variables := indets(u_cart, indexed);
```
$$variables := \{S_{1,-1,1}, S_{1,-1,2}, S_{1,-1,3}, S_{1,0,1}, S_{1,0,2}, S_{1,0,3}, S_{1,1,1}, S_{1,1,2}, S_{1,1,3}, S_{2,-2,1}, S_{2,-2,2}, S_{2,-2,3}, S_{2,-1,1}, S_{2,-1,2}, S_{2,-1,3}, S_{2,0,1}, S_{2,0,2}, S_{2,0,3}, S_{2,1,1}, S_{2,1,2}, S_{2,1,3}, S_{2,2,1}, S_{2,2,2}, S_{2,2,3}, S_{3,-3,1}, S_{3,-3,2}, S_{3,-2,1}, S_{3,-2,2}, S_{3,-1,1}, S_{3,-1,2}, S_{3,0,1}, S_{3,0,2}, S_{3,1,1}, S_{3,1,2}, S_{3,2,1}, S_{3,2,2}, S_{3,3,1}, S_{3,3,2}, S_{4,-4,1}, S_{4,-4,2}, S_{4,-3,1}, S_{4,-3,2}, S_{4,-2,1}, S_{4,-2,2}, S_{4,-1,1}, S_{4,-1,2}, S_{4,0,1}, S_{4,0,2}, S_{4,1,1}, S_{4,1,2}, S_{4,2,1}, S_{4,2,2}, S_{4,3,1}, S_{4,3,2}, S_{4,4,1}, S_{4,4,2}, S_{5,-4,1}, S_{5,-3,1}, S_{5,-2,1}, S_{5,-1,1}, S_{5,0,1}, S_{5,1,1}, S_{5,2,1}, S_{5,3,1}, S_{5,4,1}, S_{6,-4,1}, S_{6,-3,1}, S_{6,-2,1}, S_{6,-1,1}, S_{6,0,1}, S_{6,1,1}, S_{6,2,1}, S_{6,3,1}, S_{6,4,1}, T_{1,-1,0}, T_{1,-1,1}, T_{1,-1,2}, T_{1,-1,3}, T_{1,0,0}, T_{1,0,1}, T_{1,0,2}, T_{1,0,3}, T_{1,1,0}, T_{1,1,1}, T_{1,1,2}, T_{1,1,3}, T_{2,-2,0}, T_{2,-2,1}, T_{2,-2,2}, T_{2,-1,0}, T_{2,-1,1}, T_{2,-1,2}, T_{2,0,0}, T_{2,0,1}, T_{2,0,2}, T_{2,1,0}, T_{2,1,1}, T_{2,1,2}, T_{2,2,0}, T_{2,2,1}, T_{2,2,2}, T_{3,-3,0}, T_{3,-3,1}, T_{3,-3,2}, T_{3,-2,0}, T_{3,-2,1}, T_{3,-2,2}, T_{3,-1,0}, T_{3,-1,1}, T_{3,-1,2}, T_{3,0,0}, T_{3,0,1}, T_{3,0,2}, T_{3,1,0}, T_{3,1,1}, T_{3,1,2}, T_{3,2,0}, T_{3,2,1}, T_{3,2,2}, T_{3,3,0}, T_{3,3,1}, T_{3,3,2}, T_{4,-4,0}, T_{4,-4,1}, T_{4,-3,0}, T_{4,-3,1}, T_{4,-2,0}, T_{4,-2,1}, T_{4,-1,0}, T_{4,-1,1}, T_{4,0,0}, T_{4,0,1}, T_{4,1,0}, T_{4,1,1}, T_{4,2,0}, T_{4,2,1}, T_{4,3,0}, T_{4,3,1}, T_{4,4,0}, T_{4,4,1}, T_{5,-4,0}, T_{5,-4,1}, T_{5,-3,0}, T_{5,-3,1}, T_{5,-2,0}, T_{5,-2,1}, T_{5,-1,0}, T_{5,-1,1}, T_{5,0,0}, T_{5,0,1}, T_{5,1,0}, T_{5,1,1}, T_{5,2,0}, T_{5,2,1}, T_{5,3,0}, T_{5,3,1}, T_{5,4,0}, T_{5,4,1}, T_{6,-4,0}, T_{6,-3,0}, T_{6,-2,0}, T_{6,-1,0}, T_{6,0,0}, T_{6,1,0}, T_{6,2,0}, T_{6,3,0}, T_{6,4,0}\} \quad (26)$$

```
> simplify(MapToBasis(coeff(u_cart, S[2,-2,2], 1), spherical[r,
  theta,phi]));
```
$$\left(\left(-39r^5+60r^3-21r\right)\sin(\phi)\cos(\phi)\sin(\theta)^2\sqrt{3}\right)\bar{e}_r+\left(\left(-91r^5+100r^3-21r\right)\sin(\phi)\cos(\phi)\sin(\theta)\cos(\theta)\sqrt{3}\right)\bar{e}_\theta-\frac{1}{2}\sqrt{3}\,r\sin(\theta)\left(91r^4-100r^2+21\right)\left(2\cos(\phi)^2-1\right)\bar{e}_\phi \quad (27)$$

> # Compute lhs of magnetostrophic equation

```
> # slaved equation is Omega cross u = -div(p) + curl(B) cross B
> Omega_vec := VectorField([0,0,1]);  # rotation vector in
  cartesian coordinates
```
$$Omega\_vec := \bar{e}_z \quad (28)$$

```
> LHS := CrossProduct(Omega_vec, u_cart):
```

> # Solve magnetostrophic equation for basis coefficients

```
> # take the curl of slaved equation; pressure drops out
> eqn := simplify(Curl(LHS - RHS));
```

$$
\begin{aligned}
eqn := \Bigg(& -\frac{715}{16}\, x^6\, T_{1,\,-1,\,3} - \frac{5005}{16}\, z^6\, T_{1,\,-1,\,3} + 1170\, z^5\, S_{1,\,1,\,3} - \frac{715}{16}\, y^6\, T_{1,\,-1,\,3} \\
& + \frac{1}{416} \Bigg(\Big(\big(2535\, x^6\, T_{3,\,-1,\,2} + 25350\, x^5\, y\, T_{3,\,1,\,2} + \big(32955\, y^2\, T_{3,\,-1,\,2} - 15210\, z^2\, T_{3,\,-1,\,2} \\
& - 81120\, z\, S_{3,\,1,\,2} + 572\, T_{3,\,-1,\,1} - 3718\, T_{3,\,-1,\,2}\big)\, x^4 + 5720\, y \Big(\frac{195}{22}\, T_{3,\,1,\,2}\, y^2 \\
& + \frac{585}{22}\, T_{3,\,1,\,2}\, z^2 - \frac{204}{11}\, z\, S_{3,\,-1,\,2} + T_{3,\,1,\,1} - \frac{13}{2}\, T_{3,\,1,\,2} \Big)\, x^3 + \big(58305\, y^4\, T_{3,\,-1,\,2} \\
& + \big(121680\, z^2\, T_{3,\,-1,\,2} - 56160\, z\, S_{3,\,1,\,2} + 6864\, T_{3,\,-1,\,1} - 44616\, T_{3,\,-1,\,2}\big)\, y^2 \\
& - 88725\, z^4\, T_{3,\,-1,\,2} - 24960\, z^3\, S_{3,\,1,\,2} + \big(-5148\, T_{3,\,-1,\,1} + 33462\, T_{3,\,-1,\,2}\big)\, z^2 + \big(\\
& -3744\, S_{3,\,1,\,1} + 44928\, S_{3,\,1,\,2}\big)\, z - 468\, T_{3,\,-1,\,1} + 1287\, T_{3,\,-1,\,2} + 104\, T_{3,\,-1,\,0}\big)\, x^2 \\
& + 5720\, y \Big(\frac{195}{44}\, T_{3,\,1,\,2}\, y^4 + \Big(\frac{585}{22}\, T_{3,\,1,\,2}\, z^2 - \frac{204}{11}\, z\, S_{3,\,-1,\,2} + T_{3,\,1,\,1} - \frac{13}{2}\, T_{3,\,1,\,2} \Big)\, y^2 \\
& + \frac{975}{44}\, T_{3,\,1,\,2}\, z^4 - \frac{384}{11}\, z^3\, S_{3,\,-1,\,2} + \Big(3\, T_{3,\,1,\,1} - \frac{39}{2}\, T_{3,\,1,\,2} \Big)\, z^2 + \Big(-\frac{72}{55}\, S_{3,\,-1,\,1} \\
& + \frac{864}{55}\, S_{3,\,-1,\,2} \Big)\, z - \frac{9}{11}\, T_{3,\,1,\,1} + \frac{9}{4}\, T_{3,\,1,\,2} + \frac{2}{11}\, T_{3,\,1,\,0} \Big)\, x + 27885\, y^6\, T_{3,\,-1,\,2} \\
& + \big(136890\, z^2\, T_{3,\,-1,\,2} + 24960\, z\, S_{3,\,1,\,2} + 6292\, T_{3,\,-1,\,1} - 40898\, T_{3,\,-1,\,2}\big)\, y^4 \\
& + \big(38025\, z^4\, T_{3,\,-1,\,2} + 174720\, z^3\, S_{3,\,1,\,2} + \big(12012\, T_{3,\,-1,\,1} - 78078\, T_{3,\,-1,\,2}\big)\, z^2 \\
& + \big(3744\, S_{3,\,1,\,1} - 44928\, S_{3,\,1,\,2}\big)\, z - 5148\, T_{3,\,-1,\,1} + 14157\, T_{3,\,-1,\,2} + 1144\, T_{3,\,-1,\,0}\big)\, y^2 \\
& - 11440 \Big(\frac{273}{44}\, T_{3,\,-1,\,2}\, z^5 - \frac{144}{11}\, z^4\, S_{3,\,1,\,2} + \Big(T_{3,\,-1,\,1} - \frac{13}{2}\, T_{3,\,-1,\,2} \Big)\, z^3 + \Big(-\frac{48}{55}\, S_{3,\,1,\,1} \\
& + \frac{576}{55}\, S_{3,\,1,\,2} \Big)\, z^2 + \Big(-\frac{27}{55}\, T_{3,\,-1,\,1} + \frac{27}{20}\, T_{3,\,-1,\,2} + \frac{6}{55}\, T_{3,\,-1,\,0} \Big)\, z + \frac{16}{55}\, S_{3,\,1,\,1} \\
& - \frac{72}{55}\, S_{3,\,1,\,2} \Big)\, z \Big)\, \sqrt{2} + \Big(8580\, x^6\, S_{6,\,1,\,1} + 6240\, x^5\, y\, S_{6,\,-1,\,1} + \big(19500\, y^2\, S_{6,\,1,\,1} \\
& - 11700\, z^2\, S_{6,\,1,\,1} - 520\, z\, T_{6,\,-1,\,0} - 9100\, S_{6,\,1,\,1}\big)\, x^4 + 12480 \Big(S_{6,\,-1,\,1}\, y^2 + \frac{3}{4}\, S_{6,\,-1,\,1}\, z^2 \\
& - \frac{2}{3}\, z\, T_{6,\,1,\,0} - \frac{7}{12}\, S_{6,\,-1,\,1} \Big)\, y\, x^3 + \big(13260\, y^4\, S_{6,\,1,\,1} + \big(-32760\, z^2\, S_{6,\,1,\,1} \\
& - 9360\, z\, T_{6,\,-1,\,0} - 10920\, S_{6,\,1,\,1}\big)\, y^2 - 146640\, S_{6,\,1,\,1}\, z^4 + 4160\, z^3\, T_{6,\,-1,\,0} \\
& + 65520\, S_{6,\,1,\,1}\, z^2\big)\, x^2 + 6240\, y \Big(S_{6,\,-1,\,1}\, y^4 + \Big(\frac{3}{2}\, S_{6,\,-1,\,1}\, z^2 - \frac{4}{3}\, z\, T_{6,\,1,\,0} \\
& - \frac{7}{6}\, S_{6,\,-1,\,1} \Big)\, y^2 - 19\, S_{6,\,-1,\,1}\, z^4 + \frac{8}{3}\, z^3\, T_{6,\,1,\,0} + 7\, S_{6,\,-1,\,1}\, z^2 \Big)\, x + 2340\, y^6\, S_{6,\,1,\,1} + \big(
\end{aligned}
\qquad \textbf{(29)}
$$

$$-21060\, z^2 S_{6,1,1} - 8840\, z\, T_{6,-1,0} - 1820\, S_{6,1,1}\Big) y^4 - 28080 \left(S_{6,1,1} z^2 - \frac{20}{27}\, z\, T_{6,-1,0}\right.$$
$$\left. - \frac{7}{9}\, S_{6,1,1}\right) z^2 y^2 + 26208 \left(S_{6,1,1} z^2 - \frac{2}{21}\, z\, T_{6,-1,0} - \frac{5}{9}\, S_{6,1,1}\right) z^4\Big) \sqrt{7}$$
$$- 39780\, x^6 S_{2,1,3} + \left(-159120\, y\, S_{2,-1,3} + 397800\, z\, S_{2,2,3} - 7436\, T_{2,-2,2}\right) x^5$$
$$+ \Big(39780\, y^2 S_{2,1,3} + \left(79560\, z\, S_{2,-2,3} + 7436\, T_{2,2,2}\right) y + 119340\, z^2 S_{2,1,3}$$
$$- 14872\, T_{2,-1,2}\, z - 2704\, S_{2,1,2} + 27300\, S_{2,1,3}\Big) x^4 + \Big(-318240\, y^3 S_{2,-1,3}$$
$$+ \left(1432080\, z\, S_{2,2,3} - 14872\, T_{2,-2,2}\right) y^2 + \Big(-954720\, z^2 S_{2,-1,3} + 29744\, z\, T_{2,1,2}$$
$$- 21632\, S_{2,-1,2} + 218400\, S_{2,-1,3}\Big) y + 1113840\, z^3 S_{2,2,3} - 44616\, z^2\, T_{2,-2,2}$$
$$+ \left(54080\, S_{2,2,2} - 546000\, S_{2,2,3}\right) z - 1872\, T_{2,-2,1} + 10296\, T_{2,-2,2}\Big) x^3$$
$$+ \Big(198900\, y^4 S_{2,1,3} + \left(795600\, z\, S_{2,-2,3} + 14872\, T_{2,2,2}\right) y^3 + \Big(1193400\, z^2 S_{2,1,3}$$
$$+ 16224\, S_{2,1,2} - 163800\, S_{2,1,3}\Big) y^2 + \Big(795600\, z^3 S_{2,-2,3} + 44616\, T_{2,2,2}\, z^2$$
$$+ \left(32448\, S_{2,-2,2} - 327600\, S_{2,-2,3}\right) z + 1872\, T_{2,2,1} - 10296\, T_{2,2,2}\Big) y + 994500\, z^4 S_{2,1,3}$$
$$- 59488\, z^3\, T_{2,-1,2} + \left(48672\, S_{2,1,2} - 491400\, S_{2,1,3}\right) z^2 + \Big(-3744\, T_{2,-1,1}$$
$$+ 20592\, T_{2,-1,2}\Big) z + 416\, S_{2,1,1} - 4160\, S_{2,1,2} + 17316\, S_{2,1,3}\Big) x^2 + \Big(-159120\, y^5 S_{2,-1,3}$$
$$+ \left(1034280\, z\, S_{2,2,3} - 7436\, T_{2,-2,2}\right) y^4 + \Big(-954720\, z^2 S_{2,-1,3} + 29744\, z\, T_{2,1,2}$$
$$- 21632\, S_{2,-1,2} + 218400\, S_{2,-1,3}\Big) y^3 + \Big(1750320\, z^3 S_{2,2,3} - 44616\, z^2\, T_{2,-2,2}$$
$$+ \left(97344\, S_{2,2,2} - 982800\, S_{2,2,3}\right) z - 1872\, T_{2,-2,1} + 10296\, T_{2,-2,2}\Big) y^2 + \Big($$
$$-795600\, z^4 S_{2,-1,3} + 29744\, T_{2,1,2}\, z^3 + \left(-64896\, S_{2,-1,2} + 655200\, S_{2,-1,3}\right) z^2$$
$$+ \left(3744\, T_{2,1,1} - 20592\, T_{2,1,2}\right) z - 1664\, S_{2,-1,1} + 16640\, S_{2,-1,2} - 69264\, S_{2,-1,3}\Big) y$$
$$+ 716040\, z^5 S_{2,2,3} - 37180\, z^4\, T_{2,-2,2} + \left(75712\, S_{2,2,2} - 764400\, S_{2,2,3}\right) z^3 + \Big($$
$$-5616\, T_{2,-2,1} + 30888\, T_{2,-2,2}\Big) z^2 + \left(4160\, S_{2,2,1} - 41600\, S_{2,2,2} + 173160\, S_{2,2,3}\right) z$$
$$+ 1456\, T_{2,-2,1} - 3276\, T_{2,-2,2} - 416\, T_{2,-2,0}\Big) x + 119340\, y^6 S_{2,1,3} + \Big(716040\, z\, S_{2,-2,3}$$
$$+ 7436\, T_{2,2,2}\Big) y^5 + \Big(1074060\, z^2 S_{2,1,3} + 14872\, z\, T_{2,-1,2} + 18928\, S_{2,1,2}$$

$$- 191100\, S_{2,\, 1,\, 3}\big) y^4 + \big(1432080\, z^3\, S_{2,\, -2,\, 3} + 44616\, T_{2,\, 2,\, 2}\, z^2 + \big(75712\, S_{2,\, -2,\, 2}$$

$$- 764400\, S_{2,\, -2,\, 3}\big) z + 1872\, T_{2,\, 2,\, 1} - 10296\, T_{2,\, 2,\, 2}\big) y^3 + \big(1790100\, z^4\, S_{2,\, 1,\, 3}$$

$$- 29744\, z^3\, T_{2,\, -1,\, 2} + \big(113568\, S_{2,\, 1,\, 2} - 1146600\, S_{2,\, 1,\, 3}\big) z^2 + 2080\, S_{2,\, 1,\, 1} - 20800\, S_{2,\, 1,\, 2}$$

$$+ 86580\, S_{2,\, 1,\, 3}\big) y^2 + \big(716040\, z^5\, S_{2,\, -2,\, 3} + 37180\, T_{2,\, 2,\, 2}\, z^4 + \big(75712\, S_{2,\, -2,\, 2}$$

$$- 764400\, S_{2,\, -2,\, 3}\big) z^3 + \big(5616\, T_{2,\, 2,\, 1} - 30888\, T_{2,\, 2,\, 2}\big) z^2 + \big(4160\, S_{2,\, -2,\, 1} - 41600\, S_{2,\, -2,\, 2}$$

$$+ 173160\, S_{2,\, -2,\, 3}\big) z - 1456\, T_{2,\, 2,\, 1} + 3276\, T_{2,\, 2,\, 2} + 416\, T_{2,\, 2,\, 0}\big) y + 835380\, z^6\, S_{2,\, 1,\, 3}$$

$$- 44616\, z^5\, T_{2,\, -1,\, 2} + \big(94640\, S_{2,\, 1,\, 2} - 955500\, S_{2,\, 1,\, 3}\big) z^4 + \big(-7488\, T_{2,\, -1,\, 1}$$

$$+ 41184\, T_{2,\, -1,\, 2}\big) z^3 + \big(6240\, S_{2,\, 1,\, 1} - 62400\, S_{2,\, 1,\, 2} + 259740\, S_{2,\, 1,\, 3}\big) z^2 + \big(2912\, T_{2,\, -1,\, 1}$$

$$- 6552\, T_{2,\, -1,\, 2} - 832\, T_{2,\, -1,\, 0}\big) z - 1248\, S_{2,\, 1,\, 1} + 4368\, S_{2,\, 1,\, 2} - 9828\, S_{2,\, 1,\, 3}\big) \sqrt{3}$$

$$+ \frac{1}{416} \big(\big(223080\, T_{1,\, 0,\, 3} + 425880\, T_{3,\, 0,\, 2} - 93600\, T_{5,\, 0,\, 1}\big) z^3 + \big(133848\, T_{2,\, 0,\, 2}$$

$$+ 20280\, T_{4,\, 0,\, 1} - 65520\, T_{6,\, 0,\, 0}\big) z^2 + \big(20592\, T_{1,\, 0,\, 2} - 133848\, T_{1,\, 0,\, 3} + 20592\, T_{3,\, 0,\, 1}$$

$$- 133848\, T_{3,\, 0,\, 2} + 121680\, T_{5,\, 0,\, 1} - 18720\, T_{5,\, 0,\, 0} + 126000\big) z + 5616\, T_{2,\, 0,\, 1}$$

$$- 30888\, T_{2,\, 0,\, 2} + 17160\, T_{4,\, 0,\, 1} - 3120\, T_{4,\, 0,\, 0}\big) y^3 + \frac{1}{416} \big(-278850\, z^4\, T_{1,\, -1,\, 3}$$

$$+ 973440\, z^3\, S_{1,\, 1,\, 3} + \big(-30888\, T_{1,\, -1,\, 2} + 200772\, T_{1,\, -1,\, 3}\big) z^2 + \big(54912\, S_{1,\, 1,\, 2}$$

$$- 470496\, S_{1,\, 1,\, 3}\big) z - 1456\, T_{1,\, -1,\, 1} + 6552\, T_{1,\, -1,\, 2} - 18018\, T_{1,\, -1,\, 3}\big) y^2$$

$$+ \frac{1}{416} \big(\big(111540\, T_{1,\, 0,\, 3} + 60840\, T_{3,\, 0,\, 2} - 128700\, T_{5,\, 0,\, 1}\big) z + 22308\, T_{2,\, 0,\, 2}$$

$$- 20280\, T_{4,\, 0,\, 1} + 5460\, T_{6,\, 0,\, 0}\big) y^5 + \frac{1}{416} \big(-167310\, z^2\, T_{1,\, -1,\, 3} + 486720\, z\, S_{1,\, 1,\, 3}$$

$$- 5148\, T_{1,\, -1,\, 2} + 33462\, T_{1,\, -1,\, 3}\big) y^4 + \frac{1}{416} \big(\big(\big(-716040\, S_{2,\, 0,\, 3} + 106080\, S_{4,\, 0,\, 2}$$

$$+ 98280\, S_{6,\, 0,\, 1}\big) z - 60840\, S_{1,\, 0,\, 3} - 18720\, S_{3,\, 0,\, 2} + 17160\, S_{5,\, 0,\, 1}\big) y^4 - 243360\, y^3\, z\, S_{1,\, -1,\, 3}$$

$$+ \big(\big(-2386800\, S_{2,\, 0,\, 3} - 884000\, S_{4,\, 0,\, 2} + 262080\, S_{6,\, 0,\, 1}\big) z^3 + \big(-365040\, S_{1,\, 0,\, 3}$$

$$- 393120\, S_{3,\, 0,\, 2} + 37440\, S_{5,\, 0,\, 1}\big) z^2 + \big(-97344\, S_{2,\, 0,\, 2} + 982800\, S_{2,\, 0,\, 3} - 6240\, S_{4,\, 0,\, 1}$$

$$+ 87360\, S_{4,\, 0,\, 2} - 131040\, S_{6,\, 0,\, 1}\big) z - 9152\, S_{1,\, 0,\, 2} + 78416\, S_{1,\, 0,\, 3} - 3744\, S_{3,\, 0,\, 1}$$

$$+44928\, S_{3,\,0,\,2}-18720\, S_{5,\,0,\,1}\Big)\, y^2-243360\, z\left(z^2\, S_{1,\,-1,\,3}+\frac{44}{585}\, S_{1,\,-1,\,2}\right.$$

$$\left.-\frac{29}{45}\, S_{1,\,-1,\,3}\right) y+\big(-1670760\, S_{2,\,0,\,3}-1485120\, S_{4,\,0,\,2}-366912\, S_{6,\,0,\,1}\big)\, z^5+\big($$

$$-304200\, S_{1,\,0,\,3}-561600\, S_{3,\,0,\,2}-187200\, S_{5,\,0,\,1}\big)\, z^4+\big(-162240\, S_{2,\,0,\,2}+1638000\, S_{2,\,0,\,3}$$

$$-83200\, S_{4,\,0,\,1}+1164800\, S_{4,\,0,\,2}+174720\, S_{6,\,0,\,1}\big)\, z^3+\big(-27456\, S_{1,\,0,\,2}+235248\, S_{1,\,0,\,3}$$

$$-29952\, S_{3,\,0,\,1}+359424\, S_{3,\,0,\,2}+74880\, S_{5,\,0,\,1}\big)\, z^2+\big(-7488\, S_{2,\,0,\,1}+74880\, S_{2,\,0,\,2}$$

$$-311688\, S_{2,\,0,\,3}+24960\, S_{4,\,0,\,1}-137280\, S_{4,\,0,\,2}\big)\, z-832\, S_{1,\,0,\,1}+6656\, S_{1,\,0,\,2}$$

$$-22568\, S_{1,\,0,\,3}+4992\, S_{3,\,0,\,1}-22464\, S_{3,\,0,\,2}\Big)\, x+\frac{1}{416}\,\Big(-55770\, y^4\, T_{1,\,-1,\,3}$$

$$+\Big(\big(223080\, T_{1,\,0,\,3}+121680\, T_{3,\,0,\,2}-257400\, T_{5,\,0,\,1}\big)\, z+44616\, T_{2,\,0,\,2}-40560\, T_{4,\,0,\,1}$$

$$+10920\, T_{6,\,0,\,0}\Big)\, y^3+\big(-334620\, z^2\, T_{1,\,-1,\,3}+730080\, z\, S_{1,\,1,\,3}-10296\, T_{1,\,-1,\,2}$$

$$+66924\, T_{1,\,-1,\,3}\big)\, y^2+\Big(\big(223080\, T_{1,\,0,\,3}+425880\, T_{3,\,0,\,2}-93600\, T_{5,\,0,\,1}\big)\, z^3$$

$$+\big(133848\, T_{2,\,0,\,2}+20280\, T_{4,\,0,\,1}-65520\, T_{6,\,0,\,0}\big)\, z^2+\big(20592\, T_{1,\,0,\,2}-133848\, T_{1,\,0,\,3}$$

$$+20592\, T_{3,\,0,\,1}-133848\, T_{3,\,0,\,2}+121680\, T_{5,\,0,\,1}-18720\, T_{5,\,0,\,0}+189000\big)\, z$$

$$+5616\, T_{2,\,0,\,1}-30888\, T_{2,\,0,\,2}+17160\, T_{4,\,0,\,1}-3120\, T_{4,\,0,\,0}\Big)\, y-278850\, z^4\, T_{1,\,-1,\,3}$$

$$+730080\, z^3\, S_{1,\,1,\,3}+\big(-30888\, T_{1,\,-1,\,2}+200772\, T_{1,\,-1,\,3}\big)\, z^2+\big(36608\, S_{1,\,1,\,2}$$

$$-313664\, S_{1,\,1,\,3}\big)\, z-1456\, T_{1,\,-1,\,1}+6552\, T_{1,\,-1,\,2}-18018\, T_{1,\,-1,\,3}\Big)\, x^2+\frac{1}{416}\,\Big(\Big($$

$$-2574\left(x^6\, S_{6,\,3,\,1}+\left(\frac{1}{99}\, T_{6,\,-2,\,0}+\frac{24}{11}\, S_{6,\,-3,\,1}\, y+\frac{10}{3}\, S_{6,\,2,\,1}\, z\right) x^5+\left(\frac{5}{11}\, S_{6,\,3,\,1}\, y^2\right.\right.$$

$$\left.+\left(\frac{130}{33}\, S_{6,\,-2,\,1}\, z+\frac{17}{99}\, T_{6,\,2,\,0}\right) y-\frac{2}{11}\, z\, T_{6,\,-3,\,0}-\frac{35}{33}\, S_{6,\,3,\,1}+\frac{5}{11}\, S_{6,\,3,\,1}\, z^2\right) x^4$$

$$+\left(\frac{40}{11}\, S_{6,\,-3,\,1}\, y^3+\left(\frac{38}{99}\, T_{6,\,-2,\,0}+\frac{60}{11}\, S_{6,\,2,\,1}\, z\right) y^2+\left(-\frac{28}{11}\, S_{6,\,-3,\,1}+\frac{60}{11}\, S_{6,\,-3,\,1}\, z^2\right.\right.$$

$$\left.\left.-\frac{8}{33}\, z\, T_{6,\,3,\,0}\right) y+\frac{320}{33}\left(S_{6,\,2,\,1}\, z^2-\frac{1}{20}\, z\, T_{6,\,-2,\,0}-\frac{7}{15}\, S_{6,\,2,\,1}\right) z\right) x^3+\Big($$

$$-\frac{15}{11}\, S_{6,\,3,\,1}\, y^4+\left(-\frac{2}{99}\, T_{6,\,2,\,0}+\frac{20}{3}\, S_{6,\,-2,\,1}\, z\right) y^3+\left(\frac{14}{11}\, S_{6,\,3,\,1}-\frac{150}{11}\, S_{6,\,3,\,1}\, z^2\right.$$

$$\left.-\frac{20}{11}\, z\, T_{6,\,-3,\,0}\right) y^2+\frac{640}{33}\left(S_{6,\,-2,\,1}\, z^2-\frac{1}{20}\, z\, T_{6,\,2,\,0}-\frac{7}{20}\, S_{6,\,-2,\,1}\right) z\, y$$

$$-\frac{120}{11}\left(S_{6,\,3,\,1}\, z^2-\frac{4}{45}\, z\, T_{6,\,-3,\,0}-\frac{7}{15}\, S_{6,\,3,\,1}\right) z^2\Big)\, x^2+\left(\frac{16}{11}\, S_{6,\,-3,\,1}\, y^5\right.$$

$$+ \left(\frac{37}{99} T_{6,\,-2,\,0} + \frac{70}{33} S_{6,\,2,\,1} z \right) y^4 + \left(-\frac{28}{33} S_{6,\,-3,\,1} - \frac{140}{11} S_{6,\,-3,\,1} z^2 + \frac{24}{11} z T_{6,\,3,\,0} \right) y^3$$

$$- \frac{320}{33} \left(S_{6,\,2,\,1} z + \frac{7}{20} T_{6,\,-2,\,0} \right) z^2 y^2 - \frac{240}{11} z^2 \Big(S_{6,\,-3,\,1} z^2 + \frac{4}{45} z T_{6,\,3,\,0}$$

$$- \frac{7}{15} S_{6,\,-3,\,1} \Big) y - \frac{96}{11} \left(S_{6,\,2,\,1} z^2 - \frac{5}{54} z T_{6,\,-2,\,0} - \frac{14}{27} S_{6,\,2,\,1} \right) z^3 \Big) x + \frac{30}{11} y \Big($$

$$-\frac{3}{10} S_{6,\,3,\,1} y^5 + \left(S_{6,\,-2,\,1} z - \frac{19}{270} T_{6,\,2,\,0} \right) y^4 + \Big(\frac{3}{2} S_{6,\,3,\,1} z^2 + \frac{13}{45} z T_{6,\,-3,\,0}$$

$$+ \frac{7}{30} S_{6,\,3,\,1} \Big) y^3 - \frac{112}{135} \left(-\frac{6}{7} z T_{6,\,2,\,0} + S_{6,\,-2,\,1} \right) z y^2 + 4 \Big(S_{6,\,3,\,1} z^2 - \frac{4}{45} z T_{6,\,-3,\,0}$$

$$- \frac{7}{15} S_{6,\,3,\,1} \Big) z^2 y - \frac{16}{5} \left(S_{6,\,-2,\,1} z^2 + \frac{5}{54} z T_{6,\,2,\,0} - \frac{14}{27} S_{6,\,-2,\,1} \right) z^3 \Big) \Big) \sqrt{7} \sqrt{2}$$

$$+ \Big(\left(3120 z T_{5,\,-2,\,1} - 2288 S_{5,\,2,\,1}\right) x^5 - 2912 \left(-\frac{75}{28} T_{5,\,2,\,1} z + S_{5,\,-2,\,1} \right) y x^4$$

$$+ \Big(\left(28080 z T_{5,\,-2,\,1} - 3328 S_{5,\,2,\,1}\right) y^2 - 6240 T_{5,\,-2,\,1} z^3 - 6240 S_{5,\,2,\,1} z^2 + \Big($$

$$-2704 T_{5,\,-2,\,1} + 416 T_{5,\,-2,\,0} \Big) z + 2496 S_{5,\,2,\,1} \Big) x^3 - 4576 y \Big(\left(\frac{15}{11} T_{5,\,2,\,1} z + S_{5,\,-2,\,1} \right) y^2$$

$$- \frac{75}{11} T_{5,\,2,\,1} z^3 + \frac{42}{11} S_{5,\,-2,\,1} z^2 + \left(\frac{39}{22} T_{5,\,2,\,1} - \frac{3}{11} T_{5,\,2,\,0} \right) z - \frac{9}{11} S_{5,\,-2,\,1} \Big) x^2$$

$$+ \Big(\left(24960 z T_{5,\,-2,\,1} - 1040 S_{5,\,2,\,1}\right) y^4 + 16224 \Big(\frac{35}{13} T_{5,\,-2,\,1} z^2 + S_{5,\,2,\,1} z - \frac{3}{2} T_{5,\,-2,\,1}$$

$$+ \frac{3}{13} T_{5,\,-2,\,0} \Big) z y^2 + 16640 \Big(-\frac{9}{8} T_{5,\,-2,\,1} z^3 + S_{5,\,2,\,1} z^2 + \Big(\frac{13}{20} T_{5,\,-2,\,1}$$

$$- \frac{1}{10} T_{5,\,-2,\,0} \Big) z - \frac{9}{20} S_{5,\,2,\,1} \Big) z^2 \Big) x - 1664 \Big(\left(\frac{135}{16} T_{5,\,2,\,1} z + S_{5,\,-2,\,1} \right) y^4$$

$$+ \left(\frac{45}{4} T_{5,\,2,\,1} z^3 - 3 S_{5,\,-2,\,1} z^2 + \left(-\frac{65}{8} T_{5,\,2,\,1} + \frac{5}{4} T_{5,\,2,\,0} \right) z - \frac{3}{4} S_{5,\,-2,\,1} \right) y^2$$

$$- 10 \left(\frac{9}{8} T_{5,\,2,\,1} z^3 + S_{5,\,-2,\,1} z^2 + \left(-\frac{13}{20} T_{5,\,2,\,1} + \frac{1}{10} T_{5,\,2,\,0} \right) z - \frac{9}{20} S_{5,\,-2,\,1} \right) z^2 \Big) y \Big)$$

$$\sqrt{7} - 390 x^6 T_{5,\,-1,\,1} + \left(-10920 y T_{5,\,1,\,1} - 20280 z T_{3,\,-2,\,2} + 6240 S_{3,\,2,\,2} \right) x^5 + \Big($$

$$-12090 y^2 T_{5,\,-1,\,1} + \left(40560 z T_{3,\,2,\,2} - 12480 S_{3,\,-2,\,2}\right) y + 12870 z^2 T_{5,\,-1,\,1}$$

$$- 13312 z S_{5,\,1,\,1} + 338 T_{5,\,-1,\,1} - 52 T_{5,\,-1,\,0} \Big) x^4 + \Big(-21840 y^3 T_{5,\,1,\,1} + 49920 y^2 S_{3,\,2,\,2}$$

$$+ \left(32760 z^2 T_{5,\,1,\,1} - 4160 z S_{5,\,-1,\,1} - 1456 T_{5,\,1,\,0} + 9464 T_{5,\,1,\,1}\right) y - 81120 z^3 T_{3,\,-2,\,2}$$

$$+ 93600 z^2 S_{3,\,2,\,2} + \left(-4576 T_{3,\,-2,\,1} + 29744 T_{3,\,-2,\,2} \right) z + 1248 S_{3,\,2,\,1} - 14976 S_{3,\,2,\,2} \Big) x^3$$

$$+ \Big(-23010 y^4 T_{5,\,-1,\,1} + \left(40560 z T_{3,\,2,\,2} + 12480 S_{3,\,-2,\,2}\right) y^3 + \Big(58500 z^2 T_{5,\,-1,\,1}$$

$$- 22464 z S_{5,\,1,\,1} - 1560 T_{5,\,-1,\,0} + 10140 T_{5,\,-1,\,1} \Big) y^2 + 6864 \Big(\frac{325}{22} T_{3,\,2,\,2} z^2$$

$$+\frac{60}{11}\,z\,S_{3,\,-2,\,2}+T_{3,\,2,\,1}-\frac{13}{2}\,T_{3,\,2,\,2}\Big)\,z\,y+7800\,T_{5,\,-1,\,1}\,z^4-63232\,S_{5,\,1,\,1}\,z^3+\Big($$

$$-12168\,T_{5,\,-1,\,1}+1872\,T_{5,\,-1,\,0}\big)\,z^2+22464\,z\,S_{5,\,1,\,1}\Big)\,x^2+\Big(-10920\,y^5\,T_{5,\,1,\,1}$$

$$+\big(20280\,z\,T_{3,\,-2,\,2}+43680\,S_{3,\,2,\,2}\big)\,y^4+\big(32760\,z^2\,T_{5,\,1,\,1}-4160\,z\,S_{5,\,-1,\,1}-1456\,T_{5,\,1,\,0}$$

$$+9464\,T_{5,\,1,\,1}\big)\,y^3+\big(-40560\,z^3\,T_{3,\,-2,\,2}+205920\,z^2\,S_{3,\,2,\,2}+3744\,S_{3,\,2,\,1}$$

$$-44928\,S_{3,\,2,\,2}\big)\,y^2-56576\,\Big(-\frac{525}{272}\,T_{5,\,1,\,1}\,z^3+S_{5,\,-1,\,1}\,z^2+\Big(\frac{273}{272}\,T_{5,\,1,\,1}$$

$$-\frac{21}{136}\,T_{5,\,1,\,0}\Big)\,z-\frac{9}{34}\,S_{5,\,-1,\,1}\Big)\,z\,y-60840\,z^5\,T_{3,\,-2,\,2}+124800\,z^4\,S_{3,\,2,\,2}+\big($$

$$-9152\,T_{3,\,-2,\,1}+59488\,T_{3,\,-2,\,2}\big)\,z^3+\big(7488\,S_{3,\,2,\,1}-89856\,S_{3,\,2,\,2}\big)\,z^2+\big(3744\,T_{3,\,-2,\,1}$$

$$-10296\,T_{3,\,-2,\,2}-832\,T_{3,\,-2,\,0}\big)\,z-1664\,S_{3,\,2,\,1}+7488\,S_{3,\,2,\,2}\Big)\,x-11310\,y^6\,T_{5,\,-1,\,1}$$

$$+24960\,y^5\,S_{3,\,-2,\,2}+\big(45630\,z^2\,T_{5,\,-1,\,1}-9152\,z\,S_{5,\,1,\,1}-1508\,T_{5,\,-1,\,0}+9802\,T_{5,\,-1,\,1}\big)\,y^4$$

$$+\big(60840\,T_{3,\,2,\,2}\,z^3+149760\,S_{3,\,-2,\,2}\,z^2+\big(2288\,T_{3,\,2,\,1}-14872\,T_{3,\,2,\,2}\big)\,z+2496\,S_{3,\,-2,\,1}$$

$$-29952\,S_{3,\,-2,\,2}\big)\,y^3-6656\,\Big(-\frac{1125}{64}\,T_{5,\,-1,\,1}\,z^3+S_{5,\,1,\,1}\,z^2+\Big(\frac{663}{64}\,T_{5,\,-1,\,1}$$

$$-\frac{51}{32}\,T_{5,\,-1,\,0}\Big)\,z-\frac{9}{8}\,S_{5,\,1,\,1}\Big)\,z\,y^2+\big(60840\,T_{3,\,2,\,2}\,z^5+124800\,S_{3,\,-2,\,2}\,z^4+\big(9152\,T_{3,\,2,\,1}$$

$$-59488\,T_{3,\,2,\,2}\big)\,z^3+\big(7488\,S_{3,\,-2,\,1}-89856\,S_{3,\,-2,\,2}\big)\,z^2+\big(-3744\,T_{3,\,2,\,1}+10296\,T_{3,\,2,\,2}$$

$$+832\,T_{3,\,2,\,0}\big)\,z-1664\,S_{3,\,-2,\,1}+7488\,S_{3,\,-2,\,2}\big)\,y+19968\,\Big(-\frac{35}{32}\,T_{5,\,-1,\,1}\,z^3+S_{5,\,1,\,1}\,z^2$$

$$+\Big(\frac{65}{96}\,T_{5,\,-1,\,1}-\frac{5}{48}\,T_{5,\,-1,\,0}\Big)\,z-\frac{1}{2}\,S_{5,\,1,\,1}\Big)\,z^3\Big)\,\sqrt{3}+\Big(\Big(\big(9724\,S_{4,\,3,\,2}$$

$$+585\,T_{5,\,-3,\,1}\big)\,x^6+5304\,y\,\Big(S_{4,\,-3,\,2}+\frac{15}{34}\,T_{5,\,3,\,1}\Big)\,x^5+\Big(\big(52156\,S_{4,\,3,\,2}$$

$$+11115\,T_{5,\,-3,\,1}\big)\,y^2+\big(100776\,S_{4,\,3,\,2}-12285\,T_{5,\,-3,\,1}\big)\,z^2+\big(3744\,S_{5,\,3,\,1}$$

$$-4056\,T_{4,\,-3,\,1}\big)\,z+1352\,S_{4,\,3,\,1}-18928\,S_{4,\,3,\,2}-507\,T_{5,\,-3,\,1}+78\,T_{5,\,-3,\,0}\Big)\,x^4$$

$$+67184\,y\,\Big(\Big(S_{4,\,-3,\,2}-\frac{45}{323}\,T_{5,\,3,\,1}\Big)\,y^2+\Big(\frac{45}{19}\,S_{4,\,-3,\,2}+\frac{675}{1292}\,T_{5,\,3,\,1}\Big)\,z^2$$

$$+\Big(\frac{12}{323}\,S_{5,\,-3,\,1}+\frac{91}{646}\,T_{4,\,3,\,1}\Big)\,z-\frac{126}{323}\,S_{4,\,-3,\,2}-\frac{39}{1292}\,T_{5,\,3,\,1}+\frac{3}{646}\,T_{5,\,3,\,0}$$

$$+\frac{9}{323}\,S_{4,\,-3,\,1}\Big)\,x^3+\Big(\big(18564\,S_{4,\,3,\,2}+6435\,T_{5,\,-3,\,1}\big)\,y^4+\Big(\big(127296\,S_{4,\,3,\,2}$$

$$+31590\,T_{5,\,-3,\,1}\big)\,z^2+\big(14976\,S_{5,\,3,\,1}+4056\,T_{4,\,-3,\,1}\big)\,z+2496\,S_{4,\,3,\,1}-34944\,S_{4,\,3,\,2}$$

$$- 9126\, T_{5,\,-3,\,1} + 1404\, T_{5,\,-3,\,0}\big)\, y^{2} + \big(119340\, S_{4,\,3,\,2} - 23400\, T_{5,\,-3,\,1}\big)\, z^{4} + \big(19968\, S_{5,\,3,\,1}$$

$$- 8112\, T_{4,\,-3,\,1}\big)\, z^{3} + \big(6552\, S_{4,\,3,\,1} - 91728\, S_{4,\,3,\,2} + 12168\, T_{5,\,-3,\,1} - 1872\, T_{5,\,-3,\,0}\big)\, z^{2}$$

$$+ \big(-7488\, S_{5,\,3,\,1} + 3432\, T_{4,\,-3,\,1} - 624\, T_{4,\,-3,\,0}\big)\, z - 1560\, S_{4,\,3,\,1} + 8580\, S_{4,\,3,\,2}\big)\, x^{2}$$

$$+ 61880\, y\, \Big(\Big(S_{4,\,-3,\,2} - \frac{45}{238}\, T_{5,\,3,\,1}\Big)\, y^{4} + \Big(\Big(\frac{186}{35}\, S_{4,\,-3,\,2} - \frac{27}{238}\, T_{5,\,3,\,1}\Big)\, z^{2}$$

$$+ \Big(\frac{216}{595}\, S_{5,\,-3,\,1} + \frac{39}{595}\, T_{4,\,3,\,1}\Big)\, z - \frac{20}{17}\, S_{4,\,-3,\,2} + \frac{39}{238}\, T_{5,\,3,\,1} - \frac{3}{119}\, T_{5,\,3,\,0}$$

$$+ \frac{10}{119}\, S_{4,\,-3,\,1}\Big)\, y^{2} + \Big(\frac{27}{7}\, S_{4,\,-3,\,2} + \frac{90}{119}\, T_{5,\,3,\,1}\Big)\, z^{4} + \Big(\frac{384}{595}\, S_{5,\,-3,\,1}$$

$$+ \frac{156}{595}\, T_{4,\,3,\,1}\Big)\, z^{3} + \Big(-\frac{252}{85}\, S_{4,\,-3,\,2} - \frac{234}{595}\, T_{5,\,3,\,1} + \frac{36}{595}\, T_{5,\,3,\,0} + \frac{18}{85}\, S_{4,\,-3,\,1}\Big)\, z^{2} + \Big($$

$$-\frac{144}{595}\, S_{5,\,-3,\,1} - \frac{66}{595}\, T_{4,\,3,\,1} + \frac{12}{595}\, T_{4,\,3,\,0}\Big)\, z + \frac{33}{119}\, S_{4,\,-3,\,2} - \frac{6}{119}\, S_{4,\,-3,\,1}\Big)\, x$$

$$- 2184\, y^{2}\, \Big(\Big(\frac{153}{14}\, S_{4,\,3,\,2} + \frac{15}{8}\, T_{5,\,-3,\,1}\Big)\, y^{4} + \Big(\Big(\frac{459}{7}\, S_{4,\,3,\,2} - \frac{45}{56}\, T_{5,\,-3,\,1}\Big)\, z^{2}$$

$$+ \Big(4\, S_{5,\,3,\,1} - \frac{26}{21}\, T_{4,\,-3,\,1}\Big)\, z + S_{4,\,3,\,1} - 14\, S_{4,\,3,\,2} - \frac{13}{8}\, T_{5,\,-3,\,1} + \frac{1}{4}\, T_{5,\,-3,\,0}\Big)\, y^{2}$$

$$+ \Big(\frac{765}{14}\, S_{4,\,3,\,2} - \frac{75}{7}\, T_{5,\,-3,\,1}\Big)\, z^{4} + \Big(\frac{64}{7}\, S_{5,\,3,\,1} - \frac{26}{7}\, T_{4,\,-3,\,1}\Big)\, z^{3} + \Big(3\, S_{4,\,3,\,1}$$

$$- 42\, S_{4,\,3,\,2} + \frac{39}{7}\, T_{5,\,-3,\,1} - \frac{6}{7}\, T_{5,\,-3,\,0}\Big)\, z^{2} + \Big(-\frac{24}{7}\, S_{5,\,3,\,1} + \frac{11}{7}\, T_{4,\,-3,\,1}$$

$$- \frac{2}{7}\, T_{4,\,-3,\,0}\Big)\, z - \frac{5}{7}\, S_{4,\,3,\,1} + \frac{55}{14}\, S_{4,\,3,\,2}\Big)\Big)\, \sqrt{7} + \big(-29172\, S_{4,\,1,\,2} - 7605\, T_{3,\,-3,\,2}\big)\, x^{6}$$

$$- 5304\, y\, \Big(S_{4,\,-1,\,2} - \frac{195}{68}\, T_{3,\,3,\,2}\Big)\, x^{5} + \big(\big(-82212\, S_{4,\,1,\,2} - 7605\, T_{3,\,-3,\,2}\big)\, y^{2} + \big($$

$$-376584\, S_{4,\,1,\,2} - 45630\, T_{3,\,-3,\,2}\big)\, z^{2} + \big(4056\, T_{4,\,-1,\,1} + 56160\, S_{3,\,3,\,2}\big)\, z - 4056\, S_{4,\,1,\,1}$$

$$+ 56784\, S_{4,\,1,\,2} - 1716\, T_{3,\,-3,\,1} + 11154\, T_{3,\,-3,\,2}\big)\, x^{4} - 1872\, y\, \Big(\Big(\frac{17}{3}\, S_{4,\,-1,\,2}$$

$$- \frac{65}{4}\, T_{3,\,3,\,2}\Big)\, y^{2} + \Big(\frac{527}{3}\, S_{4,\,-1,\,2} - \frac{195}{4}\, T_{3,\,3,\,2}\Big)\, z^{2} + \Big(-\frac{65}{6}\, T_{4,\,1,\,1} - 50\, S_{3,\,-3,\,2}\Big)\, z$$

$$+ S_{4,\,-1,\,1} - 14\, S_{4,\,-1,\,2} - \frac{11}{6}\, T_{3,\,3,\,1} + \frac{143}{12}\, T_{3,\,3,\,2}\Big)\, x^{3} + \big(\big(-76908\, S_{4,\,1,\,2}$$

$$+ 7605\, T_{3,\,-3,\,2}\big)\, y^{4} + \big(-424320\, S_{4,\,1,\,2}\, z^{2} + \big(28392\, T_{4,\,-1,\,1} + 56160\, S_{3,\,3,\,2}\big)\, z$$

$$- 6240\, S_{4,\,1,\,1} + 87360\, S_{4,\,1,\,2}\big)\, y^{2} + \big(-322660\, S_{4,\,1,\,2} - 38025\, T_{3,\,-3,\,2}\big)\, z^{4} + \big($$

$$-2704\, T_{4,\,-1,\,1} + 74880\, S_{3,\,3,\,2}\big)\, z^{3} + \big(-20904\, S_{4,\,1,\,1} + 292656\, S_{4,\,1,\,2} - 5148\, T_{3,\,-3,\,1}$$

$$+ 33462\, T_{3,\,-3,\,2}\big)\, z^{2} + \big(-3432\, T_{4,\,-1,\,1} + 624\, T_{4,\,-1,\,0} + 3744\, S_{3,\,3,\,1} - 44928\, S_{3,\,3,\,2}\big)\, z$$

$$+4680 S_{4,1,1}-25740 S_{4,1,2}+1404 T_{3,-3,1}-3861 T_{3,-3,2}-312 T_{3,-3,0}\Big) x^2$$
$$-1872 y \Big(\Big(\frac{17}{6} S_{4,-1,2}-\frac{65}{8} T_{3,3,2}\Big) y^4+\Big(\Big(\frac{527}{3} S_{4,-1,2}-\frac{195}{4} T_{3,3,2}\Big) z^2+\Big($$
$$-\frac{65}{6} T_{4,1,1}-90 S_{3,-3,2}\Big) z+S_{4,-1,1}-14 S_{4,-1,2}-\frac{11}{6} T_{3,3,1}+\frac{143}{12} T_{3,3,2}\Big) y^2$$
$$+\Big(\frac{5015}{18} S_{4,-1,2}-\frac{325}{8} T_{3,3,2}\Big) z^4+\big(-26 T_{4,1,1}-80 S_{3,-3,2}\big) z^3+\Big(\frac{37}{3} S_{4,-1,1}$$
$$-\frac{11}{2} T_{3,3,1}-\frac{518}{3} S_{4,-1,2}+\frac{143}{4} T_{3,3,2}\Big) z^2+\big(-2 T_{4,1,0}-4 S_{3,-3,1}+11 T_{4,1,1}$$
$$+48 S_{3,-3,2}\big) z+\frac{3}{2} T_{3,3,1}-\frac{33}{8} T_{3,3,2}-\frac{1}{3} T_{3,3,0}-\frac{5}{3} S_{4,-1,1}+\frac{55}{6} S_{4,-1,2}\Big) x+\big($$
$$-23868 S_{4,1,2}+7605 T_{3,-3,2}\big) y^6+\big(\big(-47736 S_{4,1,2}+45630 T_{3,-3,2}\big) z^2$$
$$+\big(24336 T_{4,-1,1}-74880 S_{3,3,2}\big) z-2184 S_{4,1,1}+30576 S_{4,1,2}+1716 T_{3,-3,1}$$
$$-11154 T_{3,-3,2}\big) y^4+\big(\big(198900 S_{4,1,2}+38025 T_{3,-3,2}\big) z^4+\big(45968 T_{4,-1,1}$$
$$-74880 S_{3,3,2}\big) z^3+\big(2184 S_{4,1,1}-30576 S_{4,1,2}+5148 T_{3,-3,1}-33462 T_{3,-3,2}\big) z^2$$
$$+\big(-24024 T_{4,-1,1}+4368 T_{4,-1,0}-3744 S_{3,3,1}+44928 S_{3,3,2}\big) z+1560 S_{4,1,1}$$
$$-8580 S_{4,1,2}-1404 T_{3,-3,1}+3861 T_{3,-3,2}+312 T_{3,-3,0}\big) y^2+14560 \Big(\frac{153}{10} z^4 S_{4,1,2}$$
$$-\frac{39}{35} T_{4,-1,1} z^3+\big(S_{4,1,1}-14 S_{4,1,2}\big) z^2+\Big(\frac{22}{35} T_{4,-1,1}-\frac{4}{35} T_{4,-1,0}\Big) z-\frac{3}{7} S_{4,1,1}$$
$$+\frac{33}{14} S_{4,1,2}\Big) z^2\Big) \sqrt{2}+\Big(\big(\big(49504 S_{4,4,2}-9360 T_{5,-4,1}\big) z+3432 S_{5,4,1}$$
$$-1352 T_{4,-4,1}\big) x^5+8736 \Big(\Big(\frac{195}{56} T_{5,4,1}+\frac{323}{21} S_{4,-4,2}\Big) z+S_{5,-4,1}$$
$$+\frac{13}{28} T_{4,4,1}\Big) y x^4+\big(\big(\big(-42432 S_{4,4,2}+28080 T_{5,-4,1}\big) z-624 S_{5,4,1}$$
$$+2704 T_{4,-4,1}\big) y^2+\big(63648 S_{4,4,2}-18720 T_{5,-4,1}\big) z^3+\big(14976 S_{5,4,1}$$
$$-4056 T_{4,-4,1}\big) z^2+\big(2912 S_{4,4,1}-40768 S_{4,4,2}+8112 T_{5,-4,1}-1248 T_{5,-4,0}\big) z$$
$$-3744 S_{5,4,1}+1144 T_{4,-4,1}-208 T_{4,-4,0}\big) x^3+16224 \Big(\Big(\Big(\frac{15}{52} T_{5,4,1}+\frac{34}{3} S_{4,-4,2}\Big) z$$
$$+S_{5,-4,1}+\frac{1}{6} T_{4,4,1}\Big) y^2+\Big(\frac{45}{13} T_{5,4,1}+\frac{153}{13} S_{4,-4,2}\Big) z^3+\Big(\frac{36}{13} S_{5,-4,1}$$
$$+\frac{3}{4} T_{4,4,1}\Big) z^2+\Big(-\frac{3}{2} T_{5,4,1}+\frac{3}{13} T_{5,4,0}+\frac{7}{13} S_{4,-4,1}-\frac{98}{13} S_{4,-4,2}\Big) z$$
$$-\frac{9}{13} S_{5,-4,1}-\frac{11}{52} T_{4,4,1}+\frac{1}{26} T_{4,4,0}\Big) y x^2-8736 y^2 \Big(\Big(\Big(\frac{493}{21} S_{4,4,2}$$

$$- \frac{15}{7} T_{5,\,-4,\,1}\Big) z + \frac{53}{28} S_{5,\,4,\,1} - \frac{13}{28} T_{4,\,-4,\,1}\Big) y^2 + \left(\frac{153}{7} S_{4,\,4,\,2} - \frac{45}{7} T_{5,\,-4,\,1}\right) z^3$$

$$+ \left(\frac{36}{7} S_{5,\,4,\,1} - \frac{39}{28} T_{4,\,-4,\,1}\right) z^2 + \left(S_{4,\,4,\,1} - 14\, S_{4,\,4,\,2} + \frac{39}{14} T_{5,\,-4,\,1} - \frac{3}{7} T_{5,\,-4,\,0}\right) z$$

$$- \frac{9}{7} S_{5,\,4,\,1} + \frac{11}{28} T_{4,\,-4,\,1} - \frac{1}{14} T_{4,\,-4,\,0}\Big) x - 4992\, y^3 \Bigg(\bigg(\Big(\Big(\frac{45}{32} T_{5,\,4,\,1} + \frac{51}{4} S_{4,\,-4,\,2}\Big) z$$

$$+ S_{5,\,-4,\,1} + \frac{13}{48} T_{4,\,4,\,1}\Big) y^2 + \left(\frac{15}{4} T_{5,\,4,\,1} + \frac{51}{4} S_{4,\,-4,\,2}\right) z^3 + \Big(3\, S_{5,\,-4,\,1}$$

$$+ \frac{13}{16} T_{4,\,4,\,1}\Big) z^2 + \left(-\frac{13}{8} T_{5,\,4,\,1} + \frac{1}{4} T_{5,\,4,\,0} + \frac{7}{12} S_{4,\,-4,\,1} - \frac{49}{6} S_{4,\,-4,\,2}\right) z$$

$$- \frac{3}{4} S_{5,\,-4,\,1} - \frac{11}{48} T_{4,\,4,\,1} + \frac{1}{24} T_{4,\,4,\,0}\bigg)\Bigg) \sqrt{7} + \left(-77792\, z\, S_{4,\,2,\,2} + 1352\, T_{4,\,-2,\,1}\right) x^5$$

$$- 282880 \left(S_{4,\,-2,\,2}\, z - \frac{39}{1360} T_{4,\,2,\,1}\right) y\, x^4 + \Big(\left(254592\, z\, S_{4,\,2,\,2} + 21632\, T_{4,\,-2,\,1}\right) y^2$$

$$+ 297024\, S_{4,\,2,\,2}\, z^3 - 20280\, T_{4,\,-2,\,1}\, z^2 + \left(1664\, S_{4,\,2,\,1} - 23296\, S_{4,\,2,\,2}\right) z - 1144\, T_{4,\,-2,\,1}$$

$$+ 208\, T_{4,\,-2,\,0}\Big) x^3 - 11232 \left(\left(\frac{374}{27} S_{4,\,-2,\,2}\, z + \frac{13}{54} T_{4,\,2,\,1}\right) y^2 + \frac{85}{9} S_{4,\,-2,\,2}\, z^3\right.$$

$$\left. - \frac{13}{3} T_{4,\,2,\,1}\, z^2 + \left(S_{4,\,-2,\,1} - 14\, S_{4,\,-2,\,2}\right) z + \frac{11}{18} T_{4,\,2,\,1} - \frac{1}{9} T_{4,\,2,\,0}\right) y\, x^2$$

$$+ \Big(\left(332384\, z\, S_{4,\,2,\,2} + 20280\, T_{4,\,-2,\,1}\right) y^4 + \Big(1103232\, S_{4,\,2,\,2}\, z^3 + 36504\, T_{4,\,-2,\,1}\, z^2$$

$$+ \left(27456\, S_{4,\,2,\,1} - 384384\, S_{4,\,2,\,2}\right) z - 17160\, T_{4,\,-2,\,1} + 3120\, T_{4,\,-2,\,0}\Big) y^2$$

$$+ 34944 \left(\frac{459}{28} S_{4,\,2,\,2}\, z^4 - \frac{65}{56} T_{4,\,-2,\,1}\, z^3 + \left(S_{4,\,2,\,1} - 14\, S_{4,\,2,\,2}\right) z^2 + \Big(\frac{33}{56} T_{4,\,-2,\,1}\right.$$

$$\left. - \frac{3}{28} T_{4,\,-2,\,0}\Big) z - \frac{5}{14} S_{4,\,2,\,1} + \frac{55}{28} S_{4,\,2,\,2}\right) z\Big) x + 14560\, y \left(\left(\frac{306}{35} S_{4,\,-2,\,2}\, z\right.\right.$$

$$\left. - \frac{26}{35} T_{4,\,2,\,1}\right) y^4 + \left(\frac{1683}{35} S_{4,\,-2,\,2}\, z^3 - \frac{39}{70} T_{4,\,2,\,1}\, z^2 + \left(S_{4,\,-2,\,1} - 14\, S_{4,\,-2,\,2}\right) z\right.$$

$$\left. + \frac{22}{35} T_{4,\,2,\,1} - \frac{4}{35} T_{4,\,2,\,0}\right) y^2 + \frac{12}{5} \left(\frac{459}{28} S_{4,\,-2,\,2}\, z^4 + \frac{65}{56} T_{4,\,2,\,1}\, z^3 + \Big(S_{4,\,-2,\,1}\right.$$

$$\left.\left. - 14\, S_{4,\,-2,\,2}\Big) z^2 + \left(-\frac{33}{56} T_{4,\,2,\,1} + \frac{3}{28} T_{4,\,2,\,0}\right) z - \frac{5}{14} S_{4,\,-2,\,1} + \frac{55}{28} S_{4,\,-2,\,2}\right) z\Big)\Big) \sqrt{5}$$

$$+ \frac{1}{416} \left(3328\, S_{1,\,1,\,1} - 26624\, S_{1,\,1,\,2} + 90272\, S_{1,\,1,\,3}\right) z + \frac{1}{416} \Big(\Big(111540\, T_{1,\,0,\,3}$$

$$+ 365040\, T_{3,\,0,\,2} + 280800\, T_{5,\,0,\,1}\Big) z^5 + \Big(111540\, T_{2,\,0,\,2} + 135200\, T_{4,\,0,\,1}$$

$$+ 43680\, T_{6,\,0,\,0}\Big) z^4 + \Big(20592\, T_{1,\,0,\,2} - 133848\, T_{1,\,0,\,3} + 54912\, T_{3,\,0,\,1} - 356928\, T_{3,\,0,\,2}$$

$$- 162240\, T_{5,\,0,\,1} + 24960\, T_{5,\,0,\,0} + 157500\Big) z^3 + \Big(16848\, T_{2,\,0,\,1} - 92664\, T_{2,\,0,\,2}$$

$$- 68640\, T_{4,\,0,\,1} + 12480\, T_{4,\,0,\,0}\Big) z^2 + \Big(2912\, T_{1,\,0,\,1} - 13104\, T_{1,\,0,\,2} + 36036\, T_{1,\,0,\,3}$$

$$- 22464\, T_{3,\, 0,\, 1} + 61776\, T_{3,\, 0,\, 2} + 4992\, T_{3,\, 0,\, 0} - 132300 \big) z - 4368\, T_{2,\, 0,\, 1} + 9828\, T_{2,\, 0,\, 2}$$

$$+ 1248\, T_{2,\, 0,\, 0} \big) y + \frac{1}{416} \left(-25740\, T_{1,\, -1,\, 2} + 167310\, T_{1,\, -1,\, 3} \right) z^4 + \frac{1}{416} \left(54912\, S_{1,\, 1,\, 2} \right.$$

$$\left. - 470496\, S_{1,\, 1,\, 3} \right) z^3 + \frac{1}{416} \left(-4368\, T_{1,\, -1,\, 1} + 19656\, T_{1,\, -1,\, 2} - 54054\, T_{1,\, -1,\, 3} \right) z^2$$

$$+ \frac{1}{416} \Bigg(\left(29016\, z\, S_{6,\, 4,\, 1} + 312\, T_{6,\, -4,\, 0} \right) x^5 + 65520 \left(S_{6,\, -4,\, 1}\, z + \frac{1}{84}\, T_{6,\, 4,\, 0} \right) y\, x^4$$

$$+ \Big(\left(28080\, z\, S_{6,\, 4,\, 1} + 6240\, T_{6,\, -4,\, 0} \right) y^2 + 112320\, S_{6,\, 4,\, 1}\, z^3 - 9360\, z^2\, T_{6,\, -4,\, 0}$$

$$- 43680\, S_{6,\, 4,\, 1}\, z \Big) x^3 + 187200\, y \left(\left(S_{6,\, -4,\, 1}\, z - \frac{7}{120}\, T_{6,\, 4,\, 0} \right) y^2 + \frac{9}{5} \left(S_{6,\, -4,\, 1}\, z^2 \right. \right.$$

$$\left. \left. + \frac{1}{12}\, z\, T_{6,\, 4,\, 0} - \frac{7}{18}\, S_{6,\, -4,\, 1} \right) z \right) x^2 - 173160\, y^2 \left(\left(S_{6,\, 4,\, 1}\, z + \frac{5}{111}\, T_{6,\, -4,\, 0} \right) y^2 \right.$$

$$\left. + \frac{72}{37} \left(S_{6,\, 4,\, 1}\, z^2 - \frac{1}{12}\, z\, T_{6,\, -4,\, 0} - \frac{7}{18}\, S_{6,\, 4,\, 1} \right) z \right) x - 50544\, y^3 \left(\left(S_{6,\, -4,\, 1}\, z \right. \right.$$

$$\left. \left. - \frac{13}{324}\, T_{6,\, 4,\, 0} \right) y^2 + \frac{20}{9} \left(S_{6,\, -4,\, 1}\, z^2 + \frac{1}{12}\, z\, T_{6,\, 4,\, 0} - \frac{7}{18}\, S_{6,\, -4,\, 1} \right) z \right) \Bigg) \sqrt{7}$$

$$+ \frac{1}{416} \Big(\Big(\big(-1432080\, S_{2,\, 0,\, 3} + 212160\, S_{4,\, 0,\, 2} + 196560\, S_{6,\, 0,\, 1} \big) z - 121680\, S_{1,\, 0,\, 3}$$

$$- 37440\, S_{3,\, 0,\, 2} + 34320\, S_{5,\, 0,\, 1} \Big) y^2 - 243360\, y\, z\, S_{1,\, -1,\, 3} + \big(-2386800\, S_{2,\, 0,\, 3}$$

$$- 884000\, S_{4,\, 0,\, 2} + 262080\, S_{6,\, 0,\, 1} \big) z^3 + \big(-365040\, S_{1,\, 0,\, 3} - 393120\, S_{3,\, 0,\, 2}$$

$$+ 37440\, S_{5,\, 0,\, 1} \big) z^2 + \big(-97344\, S_{2,\, 0,\, 2} + 982800\, S_{2,\, 0,\, 3} - 6240\, S_{4,\, 0,\, 1} + 87360\, S_{4,\, 0,\, 2}$$

$$- 131040\, S_{6,\, 0,\, 1} \big) z - 9152\, S_{1,\, 0,\, 2} + 78416\, S_{1,\, 0,\, 3} - 3744\, S_{3,\, 0,\, 1} + 44928\, S_{3,\, 0,\, 2}$$

$$- 18720\, S_{5,\, 0,\, 1} \Big) x^3 + \frac{1}{416} \Big(-55770\, y^2\, T_{1,\, -1,\, 3} + \big(\big(111540\, T_{1,\, 0,\, 3} + 60840\, T_{3,\, 0,\, 2}$$

$$- 128700\, T_{5,\, 0,\, 1} \big) z + 22308\, T_{2,\, 0,\, 2} - 20280\, T_{4,\, 0,\, 1} + 5460\, T_{6,\, 0,\, 0} \big) y - 167310\, z^2\, T_{1,\, -1,\, 3}$$

$$+ 243360\, z\, S_{1,\, 1,\, 3} - 5148\, T_{1,\, -1,\, 2} + 33462\, T_{1,\, -1,\, 3} \Big) x^4 + \frac{1}{416} \Big(\big(-716040\, S_{2,\, 0,\, 3}$$

$$+ 106080\, S_{4,\, 0,\, 2} + 98280\, S_{6,\, 0,\, 1} \big) z - 60840\, S_{1,\, 0,\, 3} - 18720\, S_{3,\, 0,\, 2} + 17160\, S_{5,\, 0,\, 1} \Big) x^5$$

$$+ \frac{105}{16}\, T_{1,\, -1,\, 3} - \frac{35}{8}\, T_{1,\, -1,\, 2} + \frac{5}{2}\, T_{1,\, -1,\, 1} - T_{1,\, -1,\, 0} \Big) \bar{e}_x + \Big(\frac{715}{16}\, y^6\, T_{1,\, 1,\, 3}$$

$$+ \frac{5005}{16}\, z^6\, T_{1,\, 1,\, 3} + 1170\, z^5\, S_{1,\, -1,\, 3} + \frac{715}{16}\, x^6\, T_{1,\, 1,\, 3} + \frac{1}{416} \Big(\Big(-2106 \sqrt{7} \Big($$

$$- \frac{11}{9}\, S_{6,\, -3,\, 1}\, y^6 + \left(-\frac{8}{3}\, x\, S_{6,\, 3,\, 1} - \frac{110}{27}\, S_{6,\, 2,\, 1}\, z - \frac{1}{81}\, T_{6,\, -2,\, 0} \right) y^5 + \Big(-\frac{5}{9}\, x^2\, S_{6,\, -3,\, 1}$$

$$+ \left(\frac{17}{81}\, T_{6,\, 2,\, 0} + \frac{130}{27}\, S_{6,\, -2,\, 1}\, z \right) x - \frac{5}{9}\, S_{6,\, -3,\, 1}\, z^2 - \frac{2}{9}\, z\, T_{6,\, 3,\, 0} + \frac{35}{27}\, S_{6,\, -3,\, 1} \Big) y^4 + \Big($$

$$-\frac{40}{9} x^3 S_{6, 3, 1} + \Big(-\frac{38}{81} T_{6, -2, 0} - \frac{20}{3} S_{6, 2, 1} z \Big) x^2 + \Big(\frac{28}{9} S_{6, 3, 1} - \frac{20}{3} S_{6, 3, 1} z^2$$

$$- \frac{8}{27} z T_{6, -3, 0} \Big) x - \frac{320}{27} \Big(S_{6, 2, 1} z^2 - \frac{1}{20} z T_{6, -2, 0} - \frac{7}{15} S_{6, 2, 1} \Big) z \Big) y^3$$

$$+ \Big(\frac{5}{3} x^4 S_{6, -3, 1} + \Big(-\frac{2}{81} T_{6, 2, 0} + \frac{220}{27} S_{6, -2, 1} z \Big) x^3 + \Big(-\frac{14}{9} S_{6, -3, 1} + \frac{50}{3} S_{6, -3, 1} z^2$$

$$- \frac{20}{9} z T_{6, 3, 0} \Big) x^2 + \frac{640}{27} \Big(S_{6, -2, 1} z^2 - \frac{1}{20} z T_{6, 2, 0} - \frac{7}{20} S_{6, -2, 1} \Big) z x$$

$$+ \frac{40}{3} \Big(S_{6, -3, 1} z^2 + \frac{4}{45} z T_{6, 3, 0} - \frac{7}{15} S_{6, -3, 1} \Big) z^2 \Big) y^2 + \Big(-\frac{16}{9} x^5 S_{6, 3, 1} + \Big($$

$$-\frac{37}{81} T_{6, -2, 0} - \frac{70}{27} S_{6, 2, 1} z \Big) x^4 + \Big(\frac{28}{27} S_{6, 3, 1} + \frac{140}{9} S_{6, 3, 1} z^2 + \frac{8}{3} z T_{6, -3, 0} \Big) x^3$$

$$+ \frac{320}{27} \Big(S_{6, 2, 1} z + \frac{7}{20} T_{6, -2, 0} \Big) z^2 x^2 + \frac{80}{3} z^2 \Big(S_{6, 3, 1} z^2 - \frac{4}{45} z T_{6, -3, 0}$$

$$- \frac{7}{15} S_{6, 3, 1} \Big) x + \frac{32}{3} \Big(S_{6, 2, 1} z^2 - \frac{5}{54} z T_{6, -2, 0} - \frac{14}{27} S_{6, 2, 1} \Big) z^3 \Big) y + x \Big(x^5 S_{6, -3, 1}$$

$$+ \Big(\frac{10}{3} S_{6, -2, 1} z - \frac{19}{81} T_{6, 2, 0} \Big) x^4 + \Big(-5 S_{6, -3, 1} z^2 + \frac{26}{27} z T_{6, 3, 0} - \frac{7}{9} S_{6, -3, 1} \Big) x^3$$

$$- \frac{224}{81} \Big(-\frac{6}{7} z T_{6, 2, 0} + S_{6, -2, 1} \Big) z x^2 - \frac{40}{3} \Big(S_{6, -3, 1} z^2 + \frac{4}{45} z T_{6, 3, 0}$$

$$- \frac{7}{15} S_{6, -3, 1} \Big) z^2 x - \frac{32}{3} \Big(S_{6, -2, 1} z^2 + \frac{5}{54} z T_{6, 2, 0} - \frac{14}{27} S_{6, -2, 1} \Big) z^3 \Big) \Big) \sqrt{2} + \Big(\Big($$

$$-3120 z T_{5, -2, 1} + 2288 S_{5, 2, 1} \big) y^5 - 2912 x \Big(-\frac{75}{28} T_{5, 2, 1} z + S_{5, -2, 1} \Big) y^4 + \Big(\big($$

$$-28080 z T_{5, -2, 1} + 3328 S_{5, 2, 1} \big) x^2 + 6240 T_{5, -2, 1} z^3 + 6240 S_{5, 2, 1} z^2 + \big(2704 T_{5, -2, 1}$$

$$- 416 T_{5, -2, 0} \big) z - 2496 S_{5, 2, 1} \Big) y^3 - 4576 x \Big(\Big(\frac{15}{11} T_{5, 2, 1} z + S_{5, -2, 1} \Big) x^2 - \frac{75}{11} T_{5, 2, 1} z^3$$

$$+ \frac{42}{11} S_{5, -2, 1} z^2 + \Big(\frac{39}{22} T_{5, 2, 1} - \frac{3}{11} T_{5, 2, 0} \Big) z - \frac{9}{11} S_{5, -2, 1} \Big) y^2 + \Big(\big(-24960 z T_{5, -2, 1}$$

$$+ 1040 S_{5, 2, 1} \big) x^4 - 16224 z \Big(\frac{35}{13} T_{5, -2, 1} z^2 + S_{5, 2, 1} z - \frac{3}{2} T_{5, -2, 1} + \frac{3}{13} T_{5, -2, 0} \Big) x^2$$

$$- 16640 \Big(-\frac{9}{8} T_{5, -2, 1} z^3 + S_{5, 2, 1} z^2 + \Big(\frac{13}{20} T_{5, -2, 1} - \frac{1}{10} T_{5, -2, 0} \Big) z - \frac{9}{20} S_{5, 2, 1} \Big) z^2 \Big)$$

$$y - 1664 x \Big(\Big(\frac{135}{16} T_{5, 2, 1} z + S_{5, -2, 1} \Big) x^4 + \Big(\frac{45}{4} T_{5, 2, 1} z^3 - 3 S_{5, -2, 1} z^2 + \Big(-\frac{65}{8} T_{5, 2, 1}$$

$$+ \frac{5}{4} T_{5, 2, 0} \Big) z - \frac{3}{4} S_{5, -2, 1} \Big) x^2 - 10 \Big(\frac{9}{8} T_{5, 2, 1} z^3 + S_{5, -2, 1} z^2 + \Big(-\frac{13}{20} T_{5, 2, 1}$$

$$+ \frac{1}{10} T_{5, 2, 0} \Big) z - \frac{9}{20} S_{5, -2, 1} \Big) z^2 \Big) \Big) \sqrt{7} + 390 y^6 T_{5, 1, 1} + \big(10920 x T_{5, -1, 1}$$

$$+ 20280 z T_{3, -2, 2} - 6240 S_{3, 2, 2} \big) y^5 + \big(12090 x^2 T_{5, 1, 1} + \big(40560 z T_{3, 2, 2}$$

$$- 12480 S_{3, -2, 2} \big) x - 12870 z^2 T_{5, 1, 1} - 13312 z S_{5, -1, 1} - 338 T_{5, 1, 1} + 52 T_{5, 1, 0} \big) y^4$$

$$+\left(21840\,x^3\,T_{5,\,-1,\,1}-49920\,x^2\,S_{3,\,2,\,2}+\left(-32760\,z^2\,T_{5,\,-1,\,1}-4160\,z\,S_{5,\,1,\,1}\right.\right.$$

$$\left.+1456\,T_{5,\,-1,\,0}-9464\,T_{5,\,-1,\,1}\right)x+81120\,z^3\,T_{3,\,-2,\,2}-93600\,z^2\,S_{3,\,2,\,2}+\left(4576\,T_{3,\,-2,\,1}\right.$$

$$\left.\left.-29744\,T_{3,\,-2,\,2}\right)z-1248\,S_{3,\,2,\,1}+14976\,S_{3,\,2,\,2}\right)y^3+\left(23010\,x^4\,T_{5,\,1,\,1}\right.$$

$$+\left(40560\,z\,T_{3,\,2,\,2}+12480\,S_{3,\,-2,\,2}\right)x^3+\left(-58500\,z^2\,T_{5,\,1,\,1}-22464\,z\,S_{5,\,-1,\,1}\right.$$

$$\left.+1560\,T_{5,\,1,\,0}-10140\,T_{5,\,1,\,1}\right)x^2+6864\left(\frac{325}{22}\,T_{3,\,2,\,2}\,z^2+\frac{60}{11}\,z\,S_{3,\,-2,\,2}+T_{3,\,2,\,1}\right.$$

$$\left.-\frac{13}{2}\,T_{3,\,2,\,2}\right)z\,x-7800\,T_{5,\,1,\,1}\,z^4-63232\,S_{5,\,-1,\,1}\,z^3+\left(12168\,T_{5,\,1,\,1}-1872\,T_{5,\,1,\,0}\right)z^2$$

$$\left.+22464\,z\,S_{5,\,-1,\,1}\right)y^2+\left(10920\,x^5\,T_{5,\,-1,\,1}+\left(-20280\,z\,T_{3,\,-2,\,2}-43680\,S_{3,\,2,\,2}\right)x^4+\left(\right.\right.$$

$$\left.-32760\,z^2\,T_{5,\,-1,\,1}-4160\,z\,S_{5,\,1,\,1}+1456\,T_{5,\,-1,\,0}-9464\,T_{5,\,-1,\,1}\right)x^3+\left(40560\,z^3\,T_{3,\,-2,\,2}\right.$$

$$\left.-205920\,z^2\,S_{3,\,2,\,2}-3744\,S_{3,\,2,\,1}+44928\,S_{3,\,2,\,2}\right)x^2-56576\left(\frac{525}{272}\,T_{5,\,-1,\,1}\,z^3+S_{5,\,1,\,1}\,z^2\right.$$

$$\left.+\left(-\frac{273}{272}\,T_{5,\,-1,\,1}+\frac{21}{136}\,T_{5,\,-1,\,0}\right)z-\frac{9}{34}\,S_{5,\,1,\,1}\right)z\,x+60840\,z^5\,T_{3,\,-2,\,2}$$

$$-124800\,z^4\,S_{3,\,2,\,2}+\left(9152\,T_{3,\,-2,\,1}-59488\,T_{3,\,-2,\,2}\right)z^3+\left(-7488\,S_{3,\,2,\,1}\right.$$

$$\left.+89856\,S_{3,\,2,\,2}\right)z^2+\left(-3744\,T_{3,\,-2,\,1}+10296\,T_{3,\,-2,\,2}+832\,T_{3,\,-2,\,0}\right)z+1664\,S_{3,\,2,\,1}$$

$$\left.-7488\,S_{3,\,2,\,2}\right)y+11310\,x^6\,T_{5,\,1,\,1}+24960\,x^5\,S_{3,\,-2,\,2}+\left(-45630\,z^2\,T_{5,\,1,\,1}\right.$$

$$\left.-9152\,z\,S_{5,\,-1,\,1}+1508\,T_{5,\,1,\,0}-9802\,T_{5,\,1,\,1}\right)x^4+\left(60840\,T_{3,\,2,\,2}\,z^3+149760\,S_{3,\,-2,\,2}\,z^2\right.$$

$$\left.+\left(2288\,T_{3,\,2,\,1}-14872\,T_{3,\,2,\,2}\right)z+2496\,S_{3,\,-2,\,1}-29952\,S_{3,\,-2,\,2}\right)x^3$$

$$-6656\left(\frac{1125}{64}\,T_{5,\,1,\,1}\,z^3+S_{5,\,-1,\,1}\,z^2+\left(-\frac{663}{64}\,T_{5,\,1,\,1}+\frac{51}{32}\,T_{5,\,1,\,0}\right)z\right.$$

$$\left.-\frac{9}{8}\,S_{5,\,-1,\,1}\right)z\,x^2+\left(60840\,T_{3,\,2,\,2}\,z^5+124800\,S_{3,\,-2,\,2}\,z^4+\left(9152\,T_{3,\,2,\,1}\right.\right.$$

$$\left.-59488\,T_{3,\,2,\,2}\right)z^3+\left(7488\,S_{3,\,-2,\,1}-89856\,S_{3,\,-2,\,2}\right)z^2+\left(-3744\,T_{3,\,2,\,1}+10296\,T_{3,\,2,\,2}\right.$$

$$\left.\left.+832\,T_{3,\,2,\,0}\right)z-1664\,S_{3,\,-2,\,1}+7488\,S_{3,\,-2,\,2}\right)x+19968\left(\frac{35}{32}\,T_{5,\,1,\,1}\,z^3+S_{5,\,-1,\,1}\,z^2\right.$$

$$\left.\left.+\left(-\frac{65}{96}\,T_{5,\,1,\,1}+\frac{5}{48}\,T_{5,\,1,\,0}\right)z-\frac{1}{2}\,S_{5,\,-1,\,1}\right)z^3\right)\sqrt{3}+\left(\left(\left(-9724\,S_{4,\,-3,\,2}\right.\right.\right.$$

$$\left.+585\,T_{5,\,3,\,1}\right)y^6-5304\left(S_{4,\,3,\,2}-\frac{15}{34}\,T_{5,\,-3,\,1}\right)x\,y^5+\left(\left(-52156\,S_{4,\,-3,\,2}\right.\right.$$

$$\left.+11115\,T_{5,\,3,\,1}\right)x^2+\left(-100776\,S_{4,\,-3,\,2}-12285\,T_{5,\,3,\,1}\right)z^2+\left(-3744\,S_{5,\,-3,\,1}\right.$$

$$- 4056\, T_{4,\,3,\,1}\big)\, z + 18928\, S_{4,\,-3,\,2} - 507\, T_{5,\,3,\,1} + 78\, T_{5,\,3,\,0} - 1352\, S_{4,\,-3,\,1}\big)\, y^4$$

$$- 67184\, x\, \bigg(\bigg(S_{4,\,3,\,2} + \frac{45}{323}\, T_{5,\,-3,\,1}\bigg)\, x^2 + \bigg(\frac{45}{19}\, S_{4,\,3,\,2} - \frac{675}{1292}\, T_{5,\,-3,\,1}\bigg)\, z^2$$

$$+ \bigg(\frac{12}{323}\, S_{5,\,3,\,1} - \frac{91}{646}\, T_{4,\,-3,\,1}\bigg)\, z + \frac{9}{323}\, S_{4,\,3,\,1} - \frac{126}{323}\, S_{4,\,3,\,2} + \frac{39}{1292}\, T_{5,\,-3,\,1}$$

$$- \frac{3}{646}\, T_{5,\,-3,\,0}\bigg)\, y^3 + \big(\big(-18564\, S_{4,\,-3,\,2} + 6435\, T_{5,\,3,\,1}\big)\, x^4 + \big(\big(-127296\, S_{4,\,-3,\,2}$$

$$+ 31590\, T_{5,\,3,\,1}\big)\, z^2 + \big(-14976\, S_{5,\,-3,\,1} + 4056\, T_{4,\,3,\,1}\big)\, z + 34944\, S_{4,\,-3,\,2} - 9126\, T_{5,\,3,\,1}$$

$$+ 1404\, T_{5,\,3,\,0} - 2496\, S_{4,\,-3,\,1}\big)\, x^2 + \big(-119340\, S_{4,\,-3,\,2} - 23400\, T_{5,\,3,\,1}\big)\, z^4 + \big($$

$$-19968\, S_{5,\,-3,\,1} - 8112\, T_{4,\,3,\,1}\big)\, z^3 + \big(91728\, S_{4,\,-3,\,2} + 12168\, T_{5,\,3,\,1} - 1872\, T_{5,\,3,\,0}$$

$$- 6552\, S_{4,\,-3,\,1}\big)\, z^2 + \big(7488\, S_{5,\,-3,\,1} + 3432\, T_{4,\,3,\,1} - 624\, T_{4,\,3,\,0}\big)\, z - 8580\, S_{4,\,-3,\,2}$$

$$+ 1560\, S_{4,\,-3,\,1}\big)\, y^2 - 61880\, x\, \bigg(\bigg(S_{4,\,3,\,2} + \frac{45}{238}\, T_{5,\,-3,\,1}\bigg)\, x^4 + \bigg(\bigg(\frac{186}{35}\, S_{4,\,3,\,2}$$

$$+ \frac{27}{238}\, T_{5,\,-3,\,1}\bigg)\, z^2 + \bigg(\frac{216}{595}\, S_{5,\,3,\,1} - \frac{39}{595}\, T_{4,\,-3,\,1}\bigg)\, z + \frac{10}{119}\, S_{4,\,3,\,1} - \frac{20}{17}\, S_{4,\,3,\,2}$$

$$- \frac{39}{238}\, T_{5,\,-3,\,1} + \frac{3}{119}\, T_{5,\,-3,\,0}\bigg)\, x^2 + \bigg(\frac{27}{7}\, S_{4,\,3,\,2} - \frac{90}{119}\, T_{5,\,-3,\,1}\bigg)\, z^4 + \bigg(\frac{384}{595}\, S_{5,\,3,\,1}$$

$$- \frac{156}{595}\, T_{4,\,-3,\,1}\bigg)\, z^3 + \bigg(\frac{18}{85}\, S_{4,\,3,\,1} - \frac{252}{85}\, S_{4,\,3,\,2} + \frac{234}{595}\, T_{5,\,-3,\,1} - \frac{36}{595}\, T_{5,\,-3,\,0}\bigg)\, z^2 + \bigg($$

$$-\frac{144}{595}\, S_{5,\,3,\,1} + \frac{66}{595}\, T_{4,\,-3,\,1} - \frac{12}{595}\, T_{4,\,-3,\,0}\bigg)\, z - \frac{6}{119}\, S_{4,\,3,\,1} + \frac{33}{119}\, S_{4,\,3,\,2}\bigg)\, y$$

$$+ 23868\, x^2\, \bigg(\bigg(S_{4,\,-3,\,2} - \frac{35}{204}\, T_{5,\,3,\,1}\bigg)\, x^4 + \bigg(\bigg(6\, S_{4,\,-3,\,2} + \frac{5}{68}\, T_{5,\,3,\,1}\bigg)\, z^2$$

$$+ \bigg(\frac{56}{153}\, S_{5,\,-3,\,1} + \frac{52}{459}\, T_{4,\,3,\,1}\bigg)\, z - \frac{196}{153}\, S_{4,\,-3,\,2} + \frac{91}{612}\, T_{5,\,3,\,1} - \frac{7}{306}\, T_{5,\,3,\,0}$$

$$+ \frac{14}{153}\, S_{4,\,-3,\,1}\bigg)\, x^2 + \bigg(5\, S_{4,\,-3,\,2} + \frac{50}{51}\, T_{5,\,3,\,1}\bigg)\, z^4 + \bigg(\frac{128}{153}\, S_{5,\,-3,\,1} + \frac{52}{153}\, T_{4,\,3,\,1}\bigg)\, z^3$$

$$+ \bigg(-\frac{196}{51}\, S_{4,\,-3,\,2} - \frac{26}{51}\, T_{5,\,3,\,1} + \frac{4}{51}\, T_{5,\,3,\,0} + \frac{14}{51}\, S_{4,\,-3,\,1}\bigg)\, z^2 + \bigg(-\frac{16}{51}\, S_{5,\,-3,\,1}$$

$$- \frac{22}{153}\, T_{4,\,3,\,1} + \frac{4}{153}\, T_{4,\,3,\,0}\bigg)\, z + \frac{55}{153}\, S_{4,\,-3,\,2} - \frac{10}{153}\, S_{4,\,-3,\,1}\bigg)\bigg)\, \sqrt{7} + \big($$

$$-29172\, S_{4,\,-1,\,2} - 7605\, T_{3,\,3,\,2}\big)\, y^6 - 5304\, x\, \bigg(S_{4,\,1,\,2} - \frac{195}{68}\, T_{3,\,-3,\,2}\bigg)\, y^5 + \big(\big($$

$$-82212\, S_{4,\,-1,\,2} - 7605\, T_{3,\,3,\,2}\big)\, x^2 + \big(-376584\, S_{4,\,-1,\,2} - 45630\, T_{3,\,3,\,2}\big)\, z^2 + \big($$

$$-4056\, T_{4,\,1,\,1} - 56160\, S_{3,\,-3,\,2}\big)\, z - 4056\, S_{4,\,-1,\,1} + 56784\, S_{4,\,-1,\,2} - 1716\, T_{3,\,3,\,1}$$

$$+ 11154\, T_{3,\,3,\,2}\big)\, y^4 - 10608\, x\, \bigg(\bigg(S_{4,\,1,\,2} - \frac{195}{68}\, T_{3,\,-3,\,2}\bigg)\, x^2 + \bigg(31\, S_{4,\,1,\,2}$$

$$
\begin{aligned}
&- \frac{585}{68}\, T_{3,\,-3,\,2}\Big)\, z^2 + \left(\frac{65}{34}\, T_{4,\,-1,\,1} + \frac{150}{17}\, S_{3,\,3,\,2}\right) z + \frac{3}{17}\, S_{4,\,1,\,1} - \frac{42}{17}\, S_{4,\,1,\,2} \\
&- \frac{11}{34}\, T_{3,\,-3,\,1} + \frac{143}{68}\, T_{3,\,-3,\,2}\Big)\, y^3 + \Big(\big(-76908\, S_{4,\,-1,\,2} + 7605\, T_{3,\,3,\,2}\big)\, x^4 + \big(\\
&-424320\, S_{4,\,-1,\,2}\, z^2 + \big(-28392\, T_{4,\,1,\,1} - 56160\, S_{3,\,-3,\,2}\big)\, z - 6240\, S_{4,\,-1,\,1} + 87360\, S_{4,\,-1,\,2}\big) \\
&x^2 + \big(-322660\, S_{4,\,-1,\,2} - 38025\, T_{3,\,3,\,2}\big)\, z^4 + \big(2704\, T_{4,\,1,\,1} - 74880\, S_{3,\,-3,\,2}\big)\, z^3 + \big(\\
&-20904\, S_{4,\,-1,\,1} + 292656\, S_{4,\,-1,\,2} - 5148\, T_{3,\,3,\,1} + 33462\, T_{3,\,3,\,2}\big)\, z^2 + \big(3432\, T_{4,\,1,\,1} \\
&- 624\, T_{4,\,1,\,0} - 3744\, S_{3,\,-3,\,1} + 44928\, S_{3,\,-3,\,2}\big)\, z + 4680\, S_{4,\,-1,\,1} - 25740\, S_{4,\,-1,\,2} \\
&+ 1404\, T_{3,\,3,\,1} - 3861\, T_{3,\,3,\,2} - 312\, T_{3,\,3,\,0}\Big)\, y^2 - 5304\, x \left(\left(S_{4,\,1,\,2} - \frac{195}{68}\, T_{3,\,-3,\,2}\right) x^4\right. \\
&+ \left(\left(-\frac{585}{34}\, T_{3,\,-3,\,2} + 62\, S_{4,\,1,\,2}\right) z^2 + \left(\frac{540}{17}\, S_{3,\,3,\,2} + \frac{65}{17}\, T_{4,\,-1,\,1}\right) z + \frac{6}{17}\, S_{4,\,1,\,1}\right. \\
&\left. - \frac{84}{17}\, S_{4,\,1,\,2} - \frac{11}{17}\, T_{3,\,-3,\,1} + \frac{143}{34}\, T_{3,\,-3,\,2}\right) x^2 + \left(\frac{295}{3}\, S_{4,\,1,\,2} - \frac{975}{68}\, T_{3,\,-3,\,2}\right) z^4 \\
&+ \left(\frac{156}{17}\, T_{4,\,-1,\,1} + \frac{480}{17}\, S_{3,\,3,\,2}\right) z^3 + \left(\frac{74}{17}\, S_{4,\,1,\,1} - \frac{1036}{17}\, S_{4,\,1,\,2} + \frac{429}{34}\, T_{3,\,-3,\,2}\right. \\
&\left. - \frac{33}{17}\, T_{3,\,-3,\,1}\right) z^2 + \left(-\frac{66}{17}\, T_{4,\,-1,\,1} - \frac{288}{17}\, S_{3,\,3,\,2} + \frac{12}{17}\, T_{4,\,-1,\,0} + \frac{24}{17}\, S_{3,\,3,\,1}\right) z \\
&\left. + \frac{9}{17}\, T_{3,\,-3,\,1} - \frac{99}{68}\, T_{3,\,-3,\,2} - \frac{2}{17}\, T_{3,\,-3,\,0} - \frac{10}{17}\, S_{4,\,1,\,1} + \frac{55}{17}\, S_{4,\,1,\,2}\right) y + \big(\\
&-23868\, S_{4,\,-1,\,2} + 7605\, T_{3,\,3,\,2}\big)\, x^6 + \Big(\big(-47736\, S_{4,\,-1,\,2} + 45630\, T_{3,\,3,\,2}\big)\, z^2 + \big(\\
&-24336\, T_{4,\,1,\,1} + 74880\, S_{3,\,-3,\,2}\big)\, z - 2184\, S_{4,\,-1,\,1} + 30576\, S_{4,\,-1,\,2} + 1716\, T_{3,\,3,\,1} \\
&- 11154\, T_{3,\,3,\,2}\Big)\, x^4 + \Big(\big(198900\, S_{4,\,-1,\,2} + 38025\, T_{3,\,3,\,2}\big)\, z^4 + \big(-45968\, T_{4,\,1,\,1} \\
&+ 74880\, S_{3,\,-3,\,2}\big)\, z^3 + \big(2184\, S_{4,\,-1,\,1} - 30576\, S_{4,\,-1,\,2} + 5148\, T_{3,\,3,\,1} - 33462\, T_{3,\,3,\,2}\big)\, z^2 \\
&+ \big(24024\, T_{4,\,1,\,1} - 4368\, T_{4,\,1,\,0} + 3744\, S_{3,\,-3,\,1} - 44928\, S_{3,\,-3,\,2}\big)\, z + 1560\, S_{4,\,-1,\,1} \\
&- 8580\, S_{4,\,-1,\,2} - 1404\, T_{3,\,3,\,1} + 3861\, T_{3,\,3,\,2} + 312\, T_{3,\,3,\,0}\Big)\, x^2 + 14560 \left(\frac{153}{10}\, S_{4,\,-1,\,2}\, z^4\right. \\
&+ \frac{39}{35}\, T_{4,\,1,\,1}\, z^3 + \big(S_{4,\,-1,\,1} - 14\, S_{4,\,-1,\,2}\big)\, z^2 + \left(-\frac{22}{35}\, T_{4,\,1,\,1} + \frac{4}{35}\, T_{4,\,1,\,0}\right) z - \frac{3}{7}\, S_{4,\,-1,\,1} \\
&\left.\left. + \frac{33}{14}\, S_{4,\,-1,\,2}\right) z^2\right) \sqrt{2} + \Big(\Big(\big(49504\, S_{4,\,4,\,2} - 9360\, T_{5,\,-4,\,1}\big)\, z + 3432\, S_{5,\,4,\,1} \\
&- 1352\, T_{4,\,-4,\,1}\Big)\, y^5 - 8736\, x \left(\left(\frac{195}{56}\, T_{5,\,4,\,1} + \frac{323}{21}\, S_{4,\,-4,\,2}\right) z + S_{5,\,-4,\,1}\right. \\
&\left. + \frac{13}{28}\, T_{4,\,4,\,1}\right) y^4 + \Big(\Big(\big(-42432\, S_{4,\,4,\,2} + 28080\, T_{5,\,-4,\,1}\big)\, z - 624\, S_{5,\,4,\,1}
\end{aligned}
$$

$$+ 2704\, T_{4,\, -4,\, 1}) x^{2} + (63648\, S_{4,\, 4,\, 2} - 18720\, T_{5,\, -4,\, 1}) z^{3} + (14976\, S_{5,\, 4,\, 1}$$

$$- 4056\, T_{4,\, -4,\, 1}) z^{2} + (2912\, S_{4,\, 4,\, 1} - 40768\, S_{4,\, 4,\, 2} + 8112\, T_{5,\, -4,\, 1} - 1248\, T_{5,\, -4,\, 0}) z$$

$$- 3744\, S_{5,\, 4,\, 1} + 1144\, T_{4,\, -4,\, 1} - 208\, T_{4,\, -4,\, 0}) y^{3} - 16224\, x \Big(\Big(\Big(\Big(\frac{15}{52}\, T_{5,\, 4,\, 1}$$

$$+ \frac{34}{3}\, S_{4,\, -4,\, 2}\Big) z + S_{5,\, -4,\, 1} + \frac{1}{6}\, T_{4,\, 4,\, 1}\Big) x^{2} + \Big(\frac{45}{13}\, T_{5,\, 4,\, 1} + \frac{153}{13}\, S_{4,\, -4,\, 2}\Big) z^{3}$$

$$+ \Big(\frac{36}{13}\, S_{5,\, -4,\, 1} + \frac{3}{4}\, T_{4,\, 4,\, 1}\Big) z^{2} + \Big(-\frac{3}{2}\, T_{5,\, 4,\, 1} + \frac{3}{13}\, T_{5,\, 4,\, 0} + \frac{7}{13}\, S_{4,\, -4,\, 1}$$

$$- \frac{98}{13}\, S_{4,\, -4,\, 2}\Big) z - \frac{9}{13}\, S_{5,\, -4,\, 1} - \frac{11}{52}\, T_{4,\, 4,\, 1} + \frac{1}{26}\, T_{4,\, 4,\, 0}\Big) y^{2} - 205088\, x^{2} \Big(\Big(\Big(S_{4,\, 4,\, 2}$$

$$- \frac{45}{493}\, T_{5,\, -4,\, 1}\Big) z + \frac{159}{1972}\, S_{5,\, 4,\, 1} - \frac{39}{1972}\, T_{4,\, -4,\, 1}\Big) x^{2} + \Big(\frac{27}{29}\, S_{4,\, 4,\, 2}$$

$$- \frac{135}{493}\, T_{5,\, -4,\, 1}\Big) z^{3} + \Big(\frac{108}{493}\, S_{5,\, 4,\, 1} - \frac{117}{1972}\, T_{4,\, -4,\, 1}\Big) z^{2} + \Big(\frac{21}{493}\, S_{4,\, 4,\, 1} - \frac{294}{493}\, S_{4,\, 4,\, 2}$$

$$+ \frac{117}{986}\, T_{5,\, -4,\, 1} - \frac{9}{493}\, T_{5,\, -4,\, 0}\Big) z - \frac{27}{493}\, S_{5,\, 4,\, 1} + \frac{33}{1972}\, T_{4,\, -4,\, 1} - \frac{3}{986}\, T_{4,\, -4,\, 0}\Big) y$$

$$+ 4992\, x^{3} \Big(\Big(\Big(\frac{45}{32}\, T_{5,\, 4,\, 1} + \frac{51}{4}\, S_{4,\, -4,\, 2}\Big) z + S_{5,\, -4,\, 1} + \frac{13}{48}\, T_{4,\, 4,\, 1}\Big) x^{2} + \Big(\frac{15}{4}\, T_{5,\, 4,\, 1}$$

$$+ \frac{51}{4}\, S_{4,\, -4,\, 2}\Big) z^{3} + \Big(3\, S_{5,\, -4,\, 1} + \frac{13}{16}\, T_{4,\, 4,\, 1}\Big) z^{2} + \Big(-\frac{13}{8}\, T_{5,\, 4,\, 1} + \frac{1}{4}\, T_{5,\, 4,\, 0}$$

$$+ \frac{7}{12}\, S_{4,\, -4,\, 1} - \frac{49}{6}\, S_{4,\, -4,\, 2}\Big) z - \frac{3}{4}\, S_{5,\, -4,\, 1} - \frac{11}{48}\, T_{4,\, 4,\, 1} + \frac{1}{24}\, T_{4,\, 4,\, 0}\Big)\Big) \sqrt{7}$$

$$+ (77792\, z\, S_{4,\, 2,\, 2} - 1352\, T_{4,\, -2,\, 1}) y^{5} - 282880\, x \Big(S_{4,\, -2,\, 2}\, z - \frac{39}{1360}\, T_{4,\, 2,\, 1}\Big) y^{4} + (($$

$$-254592\, z\, S_{4,\, 2,\, 2} - 21632\, T_{4,\, -2,\, 1}) x^{2} - 297024\, S_{4,\, 2,\, 2}\, z^{3} + 20280\, T_{4,\, -2,\, 1}\, z^{2} + ($$

$$-1664\, S_{4,\, 2,\, 1} + 23296\, S_{4,\, 2,\, 2}) z + 1144\, T_{4,\, -2,\, 1} - 208\, T_{4,\, -2,\, 0}) y^{3} - 155584\, x \Big(\Big(S_{4,\, -2,\, 2}\, z$$

$$+ \frac{13}{748}\, T_{4,\, 2,\, 1}\Big) x^{2} + \frac{15}{22}\, S_{4,\, -2,\, 2}\, z^{3} - \frac{117}{374}\, T_{4,\, 2,\, 1}\, z^{2} + \Big(\frac{27}{374}\, S_{4,\, -2,\, 1} - \frac{189}{187}\, S_{4,\, -2,\, 2}\Big) z$$

$$+ \frac{3}{68}\, T_{4,\, 2,\, 1} - \frac{3}{374}\, T_{4,\, 2,\, 0}\Big) y^{2} + \Big((-332384\, z\, S_{4,\, 2,\, 2} - 20280\, T_{4,\, -2,\, 1}) x^{4} + ($$

$$-1103232\, S_{4,\, 2,\, 2}\, z^{3} - 36504\, T_{4,\, -2,\, 1}\, z^{2} + (-27456\, S_{4,\, 2,\, 1} + 384384\, S_{4,\, 2,\, 2}) z$$

$$+ 17160\, T_{4,\, -2,\, 1} - 3120\, T_{4,\, -2,\, 0}) x^{2} - 34944\, \Big(\frac{459}{28}\, S_{4,\, 2,\, 2}\, z^{4} - \frac{65}{56}\, T_{4,\, -2,\, 1}\, z^{3} + (S_{4,\, 2,\, 1}$$

$$- 14\, S_{4,\, 2,\, 2}) z^{2} + \Big(\frac{33}{56}\, T_{4,\, -2,\, 1} - \frac{3}{28}\, T_{4,\, -2,\, 0}\Big) z - \frac{5}{14}\, S_{4,\, 2,\, 1} + \frac{55}{28}\, S_{4,\, 2,\, 2}\Big) z\Big) y$$

$$+ 127296\, x \Big(\Big(S_{4,\, -2,\, 2}\, z - \frac{13}{153}\, T_{4,\, 2,\, 1}\Big) x^{4} + \Big(\frac{11}{2}\, S_{4,\, -2,\, 2}\, z^{3} - \frac{13}{204}\, T_{4,\, 2,\, 1}\, z^{2}$$

$$+ \Big(\frac{35}{306}\, S_{4,\, -2,\, 1} - \frac{245}{153}\, S_{4,\, -2,\, 2}\Big) z + \frac{11}{153}\, T_{4,\, 2,\, 1} - \frac{2}{153}\, T_{4,\, 2,\, 0}\Big) x^{2}$$

$$+\frac{14}{51}\left(\frac{459}{28}S_{4,-2,2}z^4+\frac{65}{56}T_{4,2,1}z^3+\left(S_{4,-2,1}-14S_{4,-2,2}\right)z^2+\left(-\frac{33}{56}T_{4,2,1}\right.\right.$$

$$\left.\left.+\frac{3}{28}T_{4,2,0}\right)z-\frac{5}{14}S_{4,-2,1}+\frac{55}{28}S_{4,-2,2}\right)z\Big)\Big)\sqrt{5}+\frac{1}{416}\Big(\Big(\Big(-2535y^6T_{3,1,2}$$

$$-25350xy^5T_{3,-1,2}+\left(-32955x^2T_{3,1,2}+15210z^2T_{3,1,2}-81120zS_{3,-1,2}-572T_{3,1,1}\right.$$

$$\left.+3718T_{3,1,2}\right)y^4-50700x\left(x^2T_{3,-1,2}+3T_{3,-1,2}z^2+\frac{136}{65}zS_{3,1,2}+\frac{22}{195}T_{3,-1,1}\right.$$

$$\left.-\frac{11}{15}T_{3,-1,2}\right)y^3+\left(-58305x^4T_{3,1,2}+\left(-121680z^2T_{3,1,2}-56160zS_{3,-1,2}\right.\right.$$

$$\left.-6864T_{3,1,1}+44616T_{3,1,2}\right)x^2+88725T_{3,1,2}z^4-24960z^3S_{3,-1,2}+\left(5148T_{3,1,1}\right.$$

$$\left.-33462T_{3,1,2}\right)z^2+\left(-3744S_{3,-1,1}+44928S_{3,-1,2}\right)z+468T_{3,1,1}-1287T_{3,1,2}$$

$$\left.-104T_{3,1,0}\right)y^2-25350x\left(x^4T_{3,-1,2}+\left(6T_{3,-1,2}z^2+\frac{272}{65}zS_{3,1,2}+\frac{44}{195}T_{3,-1,1}\right.\right.$$

$$\left.-\frac{22}{15}T_{3,-1,2}\right)x^2+5z^4T_{3,-1,2}+\frac{512}{65}z^3S_{3,1,2}+\left(\frac{44}{65}T_{3,-1,1}-\frac{22}{5}T_{3,-1,2}\right)z^2$$

$$\left.+\left(\frac{96}{325}S_{3,1,1}-\frac{1152}{325}S_{3,1,2}\right)z-\frac{12}{65}T_{3,-1,1}+\frac{33}{65}T_{3,-1,2}+\frac{8}{195}T_{3,-1,0}\right)y$$

$$-27885x^6T_{3,1,2}+\left(-136890z^2T_{3,1,2}+24960zS_{3,-1,2}-6292T_{3,1,1}\right.$$

$$\left.+40898T_{3,1,2}\right)x^4+\left(-38025T_{3,1,2}z^4+174720z^3S_{3,-1,2}+\left(-12012T_{3,1,1}\right.\right.$$

$$\left.+78078T_{3,1,2}\right)z^2+\left(3744S_{3,-1,1}-44928S_{3,-1,2}\right)z+5148T_{3,1,1}-14157T_{3,1,2}$$

$$\left.-1144T_{3,1,0}\right)x^2+11440\left(\frac{273}{44}T_{3,1,2}z^5+\frac{144}{11}z^4S_{3,-1,2}+\left(T_{3,1,1}-\frac{13}{2}T_{3,1,2}\right)z^3\right.$$

$$+\left(\frac{48}{55}S_{3,-1,1}-\frac{576}{55}S_{3,-1,2}\right)z^2+\left(-\frac{27}{55}T_{3,1,1}+\frac{27}{20}T_{3,1,2}+\frac{6}{55}T_{3,1,0}\right)z$$

$$\left.-\frac{16}{55}S_{3,-1,1}+\frac{72}{55}S_{3,-1,2}\right)z\Big)\sqrt{2}+\Big(8580y^6S_{6,-1,1}+6240xy^5S_{6,1,1}$$

$$+\left(19500x^2S_{6,-1,1}-11700z^2S_{6,-1,1}+520zT_{6,1,0}-9100S_{6,-1,1}\right)y^4$$

$$+12480\left(x^2S_{6,1,1}+\frac{3}{4}S_{6,1,1}z^2+\frac{2}{3}zT_{6,-1,0}-\frac{7}{12}S_{6,1,1}\right)xy^3+\left(13260x^4S_{6,-1,1}\right.$$

$$+\left(-32760z^2S_{6,-1,1}+9360zT_{6,1,0}-10920S_{6,-1,1}\right)x^2-146640S_{6,-1,1}z^4$$

$$\left.-4160z^3T_{6,1,0}+65520S_{6,-1,1}z^2\right)y^2+6240x\left(x^4S_{6,1,1}+\left(\frac{3}{2}S_{6,1,1}z^2+\frac{4}{3}zT_{6,-1,0}\right.\right.$$

$$\left.\left.-\frac{7}{6}S_{6,1,1}\right)x^2-19S_{6,1,1}z^4-\frac{8}{3}z^3T_{6,-1,0}+7S_{6,1,1}z^2\right)y+2340x^6S_{6,-1,1}+\Big($$

$$-21060z^2S_{6,-1,1}+8840zT_{6,1,0}-1820S_{6,-1,1}\Big)x^4-28080\Big(S_{6,-1,1}z^2+\frac{20}{27}zT_{6,1,0}$$

$$- \frac{7}{9} S_{6,\,-1,\,1}\Big) z^2 x^2 + 26208 \left(S_{6,\,-1,\,1} z^2 + \frac{2}{21} z\, T_{6,\,1,\,0} - \frac{5}{9} S_{6,\,-1,\,1} \right) z^4 \Big) \sqrt{7}$$

$$- 39780\, y^6 S_{2,\,-1,\,3} + \left(-159120\, x\, S_{2,\,1,\,3} - 397800\, z\, S_{2,\,2,\,3} + 7436\, T_{2,\,-2,\,2} \right) y^5$$

$$+ \Big(39780\, x^2 S_{2,\,-1,\,3} + \left(79560\, z\, S_{2,\,-2,\,3} + 7436\, T_{2,\,2,\,2} \right) x + 119340\, z^2 S_{2,\,-1,\,3}$$

$$+ 14872\, T_{2,\,1,\,2}\, z - 2704\, S_{2,\,-1,\,2} + 27300\, S_{2,\,-1,\,3} \Big) y^4 + \Big(-318240\, x^3 S_{2,\,1,\,3} + \Big($$

$$-1432080\, z\, S_{2,\,2,\,3} + 14872\, T_{2,\,-2,\,2} \Big) x^2 + \Big(-954720\, z^2 S_{2,\,1,\,3} - 29744\, z\, T_{2,\,-1,\,2}$$

$$- 21632\, S_{2,\,1,\,2} + 218400\, S_{2,\,1,\,3} \Big) x - 1113840\, z^3 S_{2,\,2,\,3} + 44616\, z^2 T_{2,\,-2,\,2} + \Big($$

$$-54080\, S_{2,\,2,\,2} + 546000\, S_{2,\,2,\,3} \Big) z + 1872\, T_{2,\,-2,\,1} - 10296\, T_{2,\,-2,\,2} \Big) y^3$$

$$+ \Big(198900\, x^4 S_{2,\,-1,\,3} + \left(795600\, z\, S_{2,\,-2,\,3} + 14872\, T_{2,\,2,\,2} \right) x^3 + \Big(1193400\, z^2 S_{2,\,-1,\,3}$$

$$+ 16224\, S_{2,\,-1,\,2} - 163800\, S_{2,\,-1,\,3} \Big) x^2 + \Big(795600\, z^3 S_{2,\,-2,\,3} + 44616\, T_{2,\,2,\,2} z^2$$

$$+ \left(32448\, S_{2,\,-2,\,2} - 327600\, S_{2,\,-2,\,3} \right) z + 1872\, T_{2,\,2,\,1} - 10296\, T_{2,\,2,\,2} \Big) x$$

$$+ 994500\, z^4 S_{2,\,-1,\,3} + 59488\, T_{2,\,1,\,2} z^3 + \left(48672\, S_{2,\,-1,\,2} - 491400\, S_{2,\,-1,\,3} \right) z^2$$

$$+ \left(3744\, T_{2,\,1,\,1} - 20592\, T_{2,\,1,\,2} \right) z + 416\, S_{2,\,-1,\,1} - 4160\, S_{2,\,-1,\,2} + 17316\, S_{2,\,-1,\,3} \Big) y^2 + \Big($$

$$-159120\, x^5 S_{2,\,1,\,3} + \left(-1034280\, z\, S_{2,\,2,\,3} + 7436\, T_{2,\,-2,\,2} \right) x^4 + \Big(-954720\, z^2 S_{2,\,1,\,3}$$

$$- 29744\, z\, T_{2,\,-1,\,2} - 21632\, S_{2,\,1,\,2} + 218400\, S_{2,\,1,\,3} \Big) x^3 + \Big(-1750320\, z^3 S_{2,\,2,\,3}$$

$$+ 44616\, z^2 T_{2,\,-2,\,2} + \left(-97344\, S_{2,\,2,\,2} + 982800\, S_{2,\,2,\,3} \right) z + 1872\, T_{2,\,-2,\,1}$$

$$- 10296\, T_{2,\,-2,\,2} \Big) x^2 + \Big(-795600\, z^4 S_{2,\,1,\,3} - 29744\, z^3 T_{2,\,-1,\,2} + \Big(-64896\, S_{2,\,1,\,2}$$

$$+ 655200\, S_{2,\,1,\,3} \Big) z^2 + \left(-3744\, T_{2,\,-1,\,1} + 20592\, T_{2,\,-1,\,2} \right) z - 1664\, S_{2,\,1,\,1} + 16640\, S_{2,\,1,\,2}$$

$$- 69264\, S_{2,\,1,\,3} \Big) x - 716040\, z^5 S_{2,\,2,\,3} + 37180\, z^4 T_{2,\,-2,\,2} + \Big(-75712\, S_{2,\,2,\,2}$$

$$+ 764400\, S_{2,\,2,\,3} \Big) z^3 + \left(5616\, T_{2,\,-2,\,1} - 30888\, T_{2,\,-2,\,2} \right) z^2 + \Big(-4160\, S_{2,\,2,\,1} + 41600\, S_{2,\,2,\,2}$$

$$- 173160\, S_{2,\,2,\,3} \Big) z - 1456\, T_{2,\,-2,\,1} + 3276\, T_{2,\,-2,\,2} + 416\, T_{2,\,-2,\,0} \Big) y + 119340\, x^6 S_{2,\,-1,\,3}$$

$$+ \left(716040\, z\, S_{2,\,-2,\,3} + 7436\, T_{2,\,2,\,2} \right) x^5 + \Big(1074060\, z^2 S_{2,\,-1,\,3} - 14872\, z\, T_{2,\,1,\,2}$$

$$+ 18928\, S_{2,\,-1,\,2} - 191100\, S_{2,\,-1,\,3} \Big) x^4 + \Big(1432080\, z^3 S_{2,\,-2,\,3} + 44616\, T_{2,\,2,\,2} z^2$$

$$+ \left(75712\, S_{2,\,-2,\,2} - 764400\, S_{2,\,-2,\,3}\right) z + 1872\, T_{2,\,2,\,1} - 10296\, T_{2,\,2,\,2}\Big) x^3$$

$$+ \Big(1790100\, z^4 S_{2,\,-1,\,3} + 29744\, T_{2,\,1,\,2}\, z^3 + \left(113568\, S_{2,\,-1,\,2} - 1146600\, S_{2,\,-1,\,3}\right) z^2$$

$$+ 2080\, S_{2,\,-1,\,1} - 20800\, S_{2,\,-1,\,2} + 86580\, S_{2,\,-1,\,3}\Big) x^2 + \Big(716040\, z^5 S_{2,\,-2,\,3}$$

$$+ 37180\, T_{2,\,2,\,2}\, z^4 + \left(75712\, S_{2,\,-2,\,2} - 764400\, S_{2,\,-2,\,3}\right) z^3 + \Big(5616\, T_{2,\,2,\,1}$$

$$- 30888\, T_{2,\,2,\,2}\Big) z^2 + \left(4160\, S_{2,\,-2,\,1} - 41600\, S_{2,\,-2,\,2} + 173160\, S_{2,\,-2,\,3}\right) z - 1456\, T_{2,\,2,\,1}$$

$$+ 3276\, T_{2,\,2,\,2} + 416\, T_{2,\,2,\,0}\Big) x + 835380\, z^6 S_{2,\,-1,\,3} + 44616\, z^5\, T_{2,\,1,\,2} + \Big(94640\, S_{2,\,-1,\,2}$$

$$- 955500\, S_{2,\,-1,\,3}\Big) z^4 + \left(7488\, T_{2,\,1,\,1} - 41184\, T_{2,\,1,\,2}\right) z^3 + \Big(6240\, S_{2,\,-1,\,1} - 62400\, S_{2,\,-1,\,2}$$

$$+ 259740\, S_{2,\,-1,\,3}\Big) z^2 + \left(-2912\, T_{2,\,1,\,1} + 6552\, T_{2,\,1,\,2} + 832\, T_{2,\,1,\,0}\right) z - 1248\, S_{2,\,-1,\,1}$$

$$+ 4368\, S_{2,\,-1,\,2} - 9828\, S_{2,\,-1,\,3}\Big) \sqrt{3} + \frac{1}{416} \Big(55770\, x^2\, T_{1,\,1,\,3} + \Big(\Big(-111540\, T_{1,\,0,\,3}$$

$$- 60840\, T_{3,\,0,\,2} + 128700\, T_{5,\,0,\,1}\Big) z - 22308\, T_{2,\,0,\,2} + 20280\, T_{4,\,0,\,1} - 5460\, T_{6,\,0,\,0}\Big) x$$

$$+ 167310\, z^2\, T_{1,\,1,\,3} + 243360\, z\, S_{1,\,-1,\,3} + 5148\, T_{1,\,1,\,2} - 33462\, T_{1,\,1,\,3}\Big) y^4 + \frac{1}{416} \Big(\Big($$

$$-716040\, S_{2,\,0,\,3} + 106080\, S_{4,\,0,\,2} + 98280\, S_{6,\,0,\,1}\Big) z - 60840\, S_{1,\,0,\,3} - 18720\, S_{3,\,0,\,2}$$

$$+ 17160\, S_{5,\,0,\,1}\Big) y^5 + \frac{1}{416} \Big(\left(29016\, z\, S_{6,\,4,\,1} + 312\, T_{6,\,-4,\,0}\right) y^5 - 65520\, x \Big(S_{6,\,-4,\,1}\, z$$

$$+ \frac{1}{84}\, T_{6,\,4,\,0}\Big) y^4 + \Big(\left(28080\, z\, S_{6,\,4,\,1} + 6240\, T_{6,\,-4,\,0}\right) x^2 + 112320\, S_{6,\,4,\,1}\, z^3$$

$$- 9360\, z^2\, T_{6,\,-4,\,0} - 43680\, S_{6,\,4,\,1}\, z\Big) y^3 - 187200\, x \Big(\Big(S_{6,\,-4,\,1}\, z - \frac{7}{120}\, T_{6,\,4,\,0}\Big) x^2$$

$$+ \frac{9}{5} \Big(S_{6,\,-4,\,1}\, z^2 + \frac{1}{12}\, z\, T_{6,\,4,\,0} - \frac{7}{18}\, S_{6,\,-4,\,1}\Big) z\Big) y^2 - 173160\, x^2 \Big(\Big(S_{6,\,4,\,1}\, z$$

$$+ \frac{5}{111}\, T_{6,\,-4,\,0}\Big) x^2 + \frac{72}{37} \Big(S_{6,\,4,\,1}\, z^2 - \frac{1}{12}\, z\, T_{6,\,-4,\,0} - \frac{7}{18}\, S_{6,\,4,\,1}\Big) z\Big) y$$

$$+ 50544\, x^3 \Big(\Big(S_{6,\,-4,\,1}\, z - \frac{13}{324}\, T_{6,\,4,\,0}\Big) x^2 + \frac{20}{9} \Big(S_{6,\,-4,\,1}\, z^2 + \frac{1}{12}\, z\, T_{6,\,4,\,0}$$

$$- \frac{7}{18}\, S_{6,\,-4,\,1}\Big) z\Big)\Big) \sqrt{7} + \frac{1}{416} \left(25740\, T_{1,\,1,\,2} - 167310\, T_{1,\,1,\,3}\right) z^4$$

$$+ \frac{1}{416} \left(54912\, S_{1,\,-1,\,2} - 470496\, S_{1,\,-1,\,3}\right) z^3 + \frac{1}{416} \Big(4368\, T_{1,\,1,\,1} - 19656\, T_{1,\,1,\,2}$$

$$+ 54054\, T_{1,\,1,\,3}\Big) z^2 + \frac{1}{416} \left(3328\, S_{1,\,-1,\,1} - 26624\, S_{1,\,-1,\,2} + 90272\, S_{1,\,-1,\,3}\right) z + \frac{1}{416} \Big(\Big($$

$$-111540\, T_{1,\,0,\,3} - 365040\, T_{3,\,0,\,2} - 280800\, T_{5,\,0,\,1}\Big) z^5 + \Big(-111540\, T_{2,\,0,\,2} - 135200\, T_{4,\,0,\,1}$$

$$- 43680\, T_{6,\,0,\,0}\big)\, z^4 + \big(-20592\, T_{1,\,0,\,2} + 133848\, T_{1,\,0,\,3} - 54912\, T_{3,\,0,\,1} + 356928\, T_{3,\,0,\,2}$$

$$+ 162240\, T_{5,\,0,\,1} - 24960\, T_{5,\,0,\,0} - 157500\big)\, z^3 + \big(-16848\, T_{2,\,0,\,1} + 92664\, T_{2,\,0,\,2}$$

$$+ 68640\, T_{4,\,0,\,1} - 12480\, T_{4,\,0,\,0}\big)\, z^2 + \big(-2912\, T_{1,\,0,\,1} + 13104\, T_{1,\,0,\,2} - 36036\, T_{1,\,0,\,3}$$

$$+ 22464\, T_{3,\,0,\,1} - 61776\, T_{3,\,0,\,2} - 4992\, T_{3,\,0,\,0} + 132300\big)\, z + 4368\, T_{2,\,0,\,1} - 9828\, T_{2,\,0,\,2}$$

$$- 1248\, T_{2,\,0,\,0}\big)\, x + \frac{1}{416}\, \Big(\big(-223080\, T_{1,\,0,\,3} - 425880\, T_{3,\,0,\,2} + 93600\, T_{5,\,0,\,1}\big)\, z^3 + \big($$

$$-133848\, T_{2,\,0,\,2} - 20280\, T_{4,\,0,\,1} + 65520\, T_{6,\,0,\,0}\big)\, z^2 + \big(-20592\, T_{1,\,0,\,2} + 133848\, T_{1,\,0,\,3}$$

$$- 20592\, T_{3,\,0,\,1} + 133848\, T_{3,\,0,\,2} - 121680\, T_{5,\,0,\,1} + 18720\, T_{5,\,0,\,0} - 126000\big)\, z$$

$$- 5616\, T_{2,\,0,\,1} + 30888\, T_{2,\,0,\,2} - 17160\, T_{4,\,0,\,1} + 3120\, T_{4,\,0,\,0}\Big)\, x^3 + \frac{1}{416}\, \Big(278850\, z^4\, T_{1,\,1,\,3}$$

$$+ 973440\, z^3\, S_{1,\,-1,\,3} + \big(30888\, T_{1,\,1,\,2} - 200772\, T_{1,\,1,\,3}\big)\, z^2 + \big(54912\, S_{1,\,-1,\,2}$$

$$- 470496\, S_{1,\,-1,\,3}\big)\, z + 1456\, T_{1,\,1,\,1} - 6552\, T_{1,\,1,\,2} + 18018\, T_{1,\,1,\,3}\Big)\, x^2$$

$$+ \frac{1}{416}\, \big(167310\, z^2\, T_{1,\,1,\,3} + 486720\, z\, S_{1,\,-1,\,3} + 5148\, T_{1,\,1,\,2} - 33462\, T_{1,\,1,\,3}\big)\, x^4$$

$$+ \frac{1}{416}\, \Big(\big(-111540\, T_{1,\,0,\,3} - 60840\, T_{3,\,0,\,2} + 128700\, T_{5,\,0,\,1}\big)\, z - 22308\, T_{2,\,0,\,2}$$

$$+ 20280\, T_{4,\,0,\,1} - 5460\, T_{6,\,0,\,0}\Big)\, x^5 + \frac{1}{416}\, \bigg(\Big(\big(-716040\, S_{2,\,0,\,3} + 106080\, S_{4,\,0,\,2}$$

$$+ 98280\, S_{6,\,0,\,1}\big)\, z - 60840\, S_{1,\,0,\,3} - 18720\, S_{3,\,0,\,2} + 17160\, S_{5,\,0,\,1}\Big)\, x^4 - 243360\, x^3\, z\, S_{1,\,1,\,3}$$

$$+ \Big(\big(-2386800\, S_{2,\,0,\,3} - 884000\, S_{4,\,0,\,2} + 262080\, S_{6,\,0,\,1}\big)\, z^3 + \big(-365040\, S_{1,\,0,\,3}$$

$$- 393120\, S_{3,\,0,\,2} + 37440\, S_{5,\,0,\,1}\big)\, z^2 + \big(-97344\, S_{2,\,0,\,2} + 982800\, S_{2,\,0,\,3} - 6240\, S_{4,\,0,\,1}$$

$$+ 87360\, S_{4,\,0,\,2} - 131040\, S_{6,\,0,\,1}\big)\, z - 9152\, S_{1,\,0,\,2} + 78416\, S_{1,\,0,\,3} - 3744\, S_{3,\,0,\,1}$$

$$+ 44928\, S_{3,\,0,\,2} - 18720\, S_{5,\,0,\,1}\Big)\, x^2 - 243360\, \left(z^2\, S_{1,\,1,\,3} + \frac{44}{585}\, S_{1,\,1,\,2} - \frac{29}{45}\, S_{1,\,1,\,3}\right)\, z\, x$$

$$+ \big(-1670760\, S_{2,\,0,\,3} - 1485120\, S_{4,\,0,\,2} - 366912\, S_{6,\,0,\,1}\big)\, z^5 + \big(-304200\, S_{1,\,0,\,3}$$

$$- 561600\, S_{3,\,0,\,2} - 187200\, S_{5,\,0,\,1}\big)\, z^4 + \big(-162240\, S_{2,\,0,\,2} + 1638000\, S_{2,\,0,\,3} - 83200\, S_{4,\,0,\,1}$$

$$+ 1164800\, S_{4,\,0,\,2} + 174720\, S_{6,\,0,\,1}\big)\, z^3 + \big(-27456\, S_{1,\,0,\,2} + 235248\, S_{1,\,0,\,3} - 29952\, S_{3,\,0,\,1}$$

$$+ 359424\, S_{3,\,0,\,2} + 74880\, S_{5,\,0,\,1}\big)\, z^2 + \big(-7488\, S_{2,\,0,\,1} + 74880\, S_{2,\,0,\,2} - 311688\, S_{2,\,0,\,3}$$

$$+ 24960\, S_{4,\, 0,\, 1} - 137280\, S_{4,\, 0,\, 2}\big)\, z - 832\, S_{1,\, 0,\, 1} + 6656\, S_{1,\, 0,\, 2} - 22568\, S_{1,\, 0,\, 3}$$

$$+ 4992\, S_{3,\, 0,\, 1} - 22464\, S_{3,\, 0,\, 2}\big)\, y + \frac{1}{416}\, \big(55770\, x^4\, T_{1,\, 1,\, 3} + \big(\big(-223080\, T_{1,\, 0,\, 3}$$

$$- 121680\, T_{3,\, 0,\, 2} + 257400\, T_{5,\, 0,\, 1}\big)\, z - 44616\, T_{2,\, 0,\, 2} + 40560\, T_{4,\, 0,\, 1} - 10920\, T_{6,\, 0,\, 0}\big)\, x^3$$

$$+ \big(334620\, z^2\, T_{1,\, 1,\, 3} + 730080\, z\, S_{1,\, -1,\, 3} + 10296\, T_{1,\, 1,\, 2} - 66924\, T_{1,\, 1,\, 3}\big)\, x^2 + \big(\big($$

$$-223080\, T_{1,\, 0,\, 3} - 425880\, T_{3,\, 0,\, 2} + 93600\, T_{5,\, 0,\, 1}\big)\, z^3 + \big(-133848\, T_{2,\, 0,\, 2} - 20280\, T_{4,\, 0,\, 1}$$

$$+ 65520\, T_{6,\, 0,\, 0}\big)\, z^2 + \big(-20592\, T_{1,\, 0,\, 2} + 133848\, T_{1,\, 0,\, 3} - 20592\, T_{3,\, 0,\, 1} + 133848\, T_{3,\, 0,\, 2}$$

$$- 121680\, T_{5,\, 0,\, 1} + 18720\, T_{5,\, 0,\, 0} - 189000\big)\, z - 5616\, T_{2,\, 0,\, 1} + 30888\, T_{2,\, 0,\, 2} - 17160\, T_{4,\, 0,\, 1}$$

$$+ 3120\, T_{4,\, 0,\, 0}\big)\, x + 278850\, z^4\, T_{1,\, 1,\, 3} + 730080\, z^3\, S_{1,\, -1,\, 3} + \big(30888\, T_{1,\, 1,\, 2}$$

$$- 200772\, T_{1,\, 1,\, 3}\big)\, z^2 + \big(36608\, S_{1,\, -1,\, 2} - 313664\, S_{1,\, -1,\, 3}\big)\, z + 1456\, T_{1,\, 1,\, 1} - 6552\, T_{1,\, 1,\, 2}$$

$$+ 18018\, T_{1,\, 1,\, 3}\big)\, y^2 + \frac{1}{416}\, \big(\big(\big(-1432080\, S_{2,\, 0,\, 3} + 212160\, S_{4,\, 0,\, 2} + 196560\, S_{6,\, 0,\, 1}\big)\, z$$

$$- 121680\, S_{1,\, 0,\, 3} - 37440\, S_{3,\, 0,\, 2} + 34320\, S_{5,\, 0,\, 1}\big)\, x^2 - 243360\, x\, z\, S_{1,\, 1,\, 3} + \big($$

$$-2386800\, S_{2,\, 0,\, 3} - 884000\, S_{4,\, 0,\, 2} + 262080\, S_{6,\, 0,\, 1}\big)\, z^3 + \big(-365040\, S_{1,\, 0,\, 3} - 393120\, S_{3,\, 0,\, 2}$$

$$+ 37440\, S_{5,\, 0,\, 1}\big)\, z^2 + \big(-97344\, S_{2,\, 0,\, 2} + 982800\, S_{2,\, 0,\, 3} - 6240\, S_{4,\, 0,\, 1} + 87360\, S_{4,\, 0,\, 2}$$

$$- 131040\, S_{6,\, 0,\, 1}\big)\, z - 9152\, S_{1,\, 0,\, 2} + 78416\, S_{1,\, 0,\, 3} - 3744\, S_{3,\, 0,\, 1} + 44928\, S_{3,\, 0,\, 2}$$

$$- 18720\, S_{5,\, 0,\, 1}\big)\, y^3 - \frac{105}{16}\, T_{1,\, 1,\, 3} + \frac{35}{8}\, T_{1,\, 1,\, 2} - \frac{5}{2}\, T_{1,\, 1,\, 1} + T_{1,\, 1,\, 0}\Big)\, \bar{e}_y + \bigg(\frac{1}{208}\, \bigg(\bigg($$

$$-1950\, \bigg(x^6\, S_{6,\, 2,\, 1} + \bigg(2\, S_{6,\, -2,\, 1}\, y - 3\, S_{6,\, 3,\, 1}\, z + \frac{3}{50}\, T_{6,\, -3,\, 0}\bigg)\, x^5 + \bigg(y^2\, S_{6,\, 2,\, 1} + \bigg($$

$$-9\, S_{6,\, -3,\, 1}\, z - \frac{9}{50}\, T_{6,\, 3,\, 0}\bigg)\, y - \frac{24}{5}\, S_{6,\, 2,\, 1}\, z^2 + \frac{16}{75}\, z\, T_{6,\, -2,\, 0} - \frac{56}{75}\, S_{6,\, 2,\, 1}\bigg)\, x^4$$

$$+ \bigg(4\, S_{6,\, -2,\, 1}\, y^3 + \bigg(-\frac{3}{25}\, T_{6,\, -3,\, 0} + 6\, S_{6,\, 3,\, 1}\, z\bigg)\, y^2 + \bigg(-\frac{112}{75}\, S_{6,\, -2,\, 1} - \frac{48}{5}\, S_{6,\, -2,\, 1}\, z^2$$

$$- \frac{32}{75}\, z\, T_{6,\, 2,\, 0}\bigg)\, y - \frac{16}{5}\, \bigg(S_{6,\, 3,\, 1}\, z^2 + \frac{3}{20}\, z\, T_{6,\, -3,\, 0} - \frac{7}{10}\, S_{6,\, 3,\, 1}\bigg)\, z\bigg)\, x^3 + \bigg(-y^4\, S_{6,\, 2,\, 1}$$

$$+ \bigg(-6\, S_{6,\, -3,\, 1}\, z - \frac{3}{25}\, T_{6,\, 3,\, 0}\bigg)\, y^3 - \frac{48}{5}\, \bigg(S_{6,\, -3,\, 1}\, z^2 - \frac{3}{20}\, z\, T_{6,\, 3,\, 0} - \frac{7}{10}\, S_{6,\, -3,\, 1}\bigg)\, z\, y$$

$$- \frac{32}{5}\, \bigg(S_{6,\, 2,\, 1}\, z^2 + \frac{1}{15}\, z\, T_{6,\, -2,\, 0} - \frac{7}{10}\, S_{6,\, 2,\, 1}\bigg)\, z^2\bigg)\, x^2 + 2\, \bigg(S_{6,\, -2,\, 1}\, y^4 + \bigg(\frac{9}{2}\, S_{6,\, 3,\, 1}\, z$$

$$- \frac{9}{100}\, T_{6,\, -3,\, 0}\bigg)\, y^3 + \bigg(-\frac{24}{5}\, S_{6,\, -2,\, 1}\, z^2 - \frac{16}{75}\, z\, T_{6,\, 2,\, 0} - \frac{56}{75}\, S_{6,\, -2,\, 1}\bigg)\, y^2$$

$$+ \frac{24}{5} \Big(S_{6, 3, 1} z^2 + \frac{3}{20} z T_{6, -3, 0} - \frac{7}{10} S_{6, 3, 1}\Big) z y - \frac{32}{5} \Big(S_{6, -2, 1} z^2 - \frac{1}{15} z T_{6, 2, 0}$$

$$- \frac{7}{10} S_{6, -2, 1}\Big) z^2\Big) y x + 3 \Big(-\frac{1}{3} y^4 S_{6, 2, 1} + \Big(S_{6, -3, 1} z + \frac{1}{50} T_{6, 3, 0}\Big) y^3 + \Big(\frac{8}{5} S_{6, 2, 1} z^2$$

$$- \frac{16}{225} z T_{6, -2, 0} + \frac{56}{225} S_{6, 2, 1}\Big) y^2 + \frac{16}{15} \Big(S_{6, -3, 1} z^2 - \frac{3}{20} z T_{6, 3, 0} - \frac{7}{10} S_{6, -3, 1}\Big) z y$$

$$+ \frac{32}{15} \Big(S_{6, 2, 1} z^2 + \frac{1}{15} z T_{6, -2, 0} - \frac{7}{10} S_{6, 2, 1}\Big) z^2\Big) y^2\Big) \sqrt{7} \sqrt{2} + \Big(-780 x^6 T_{5, -2, 1}$$

$$+ 1560 y x^5 T_{5, 2, 1} + \big(-780 y^2 T_{5, -2, 1} + 2340 z^2 T_{5, -2, 1} + 5200 z S_{5, 2, 1} - 104 T_{5, -2, 0}$$

$$+ 676 T_{5, -2, 1}\big) x^4 + 3120 y \Big(T_{5, 2, 1} y^2 - \frac{3}{2} T_{5, 2, 1} z^2 + \frac{10}{3} z S_{5, -2, 1} - \frac{13}{30} T_{5, 2, 1}$$

$$+ \frac{1}{15} T_{5, 2, 0}\Big) x^3 + \Big(780 y^4 T_{5, -2, 1} + 7800 z \Big(T_{5, -2, 1} z^3 + \frac{56}{75} S_{5, 2, 1} z^2 + \Big(-\frac{13}{25} T_{5, -2, 1}$$

$$+ \frac{2}{25} T_{5, -2, 0}\Big) z - \frac{12}{25} S_{5, 2, 1}\Big)\Big) x^2 + 1560 y \Big(T_{5, 2, 1} y^4 + \Big(-3 T_{5, 2, 1} z^2 + \frac{20}{3} z S_{5, -2, 1}$$

$$- \frac{13}{15} T_{5, 2, 1} + \frac{2}{15} T_{5, 2, 0}\Big) y^2 - 10 \Big(T_{5, 2, 1} z^3 - \frac{56}{75} S_{5, -2, 1} z^2 + \Big(-\frac{13}{25} T_{5, 2, 1}$$

$$+ \frac{2}{25} T_{5, 2, 0}\Big) z + \frac{12}{25} S_{5, -2, 1}\Big) z\Big) x + 780 \Big(y^4 T_{5, -2, 1} + \Big(-3 T_{5, -2, 1} z^2 - \frac{20}{3} S_{5, 2, 1} z$$

$$- \frac{13}{15} T_{5, -2, 1} + \frac{2}{15} T_{5, -2, 0}\Big) y^2 - 10 z \Big(T_{5, -2, 1} z^3 + \frac{56}{75} S_{5, 2, 1} z^2 + \Big(-\frac{13}{25} T_{5, -2, 1}$$

$$+ \frac{2}{25} T_{5, -2, 0}\Big) z - \frac{12}{25} S_{5, 2, 1}\Big)\Big) y^2\Big) \sqrt{7} + 5070 x^6 T_{3, -2, 2} + \big(-10140 y T_{3, 2, 2}$$

$$- 4290 z T_{5, -1, 1} - 5252 S_{5, 1, 1}\big) x^5 + \big(5070 T_{3, -2, 2} y^2 + (4290 z T_{5, 1, 1} - 5252 S_{5, -1, 1}) y$$

$$+ 30420 T_{3, -2, 2} z^2 + 6240 z S_{3, 2, 2} + 1144 T_{3, -2, 1} - 7436 T_{3, -2, 2}\big) x^4 + \big(-20280 T_{3, 2, 2} y^3$$

$$+ (-8580 z T_{5, -1, 1} - 10504 S_{5, 1, 1}) y^2 + (-60840 z^2 T_{3, 2, 2} + 12480 z S_{3, -2, 2}$$

$$- 2288 T_{3, 2, 1} + 14872 T_{3, 2, 2}) y - 3120 T_{5, -1, 1} z^3 + 14352 S_{5, 1, 1} z^2 + (4056 T_{5, -1, 1}$$

$$- 624 T_{5, -1, 0}) z + 3744 S_{5, 1, 1}\big) x^3 + \big(-5070 y^4 T_{3, -2, 2} + (8580 z T_{5, 1, 1}$$

$$- 10504 S_{5, -1, 1}) y^3 + (3120 T_{5, 1, 1} z^3 + 14352 S_{5, -1, 1} z^2 + (-4056 T_{5, 1, 1} + 624 T_{5, 1, 0}) z$$

$$+ 3744 S_{5, -1, 1}) y + 25350 T_{3, -2, 2} z^4 - 12480 z^3 S_{3, 2, 2} + (3432 T_{3, -2, 1}$$

$$- 22308 T_{3, -2, 2}) z^2 - 936 T_{3, -2, 1} + 2574 T_{3, -2, 2} + 208 T_{3, -2, 0}\big) x^2 + \Big(-10140 T_{3, 2, 2} y^5$$

$$+ (-4290 z T_{5, -1, 1} - 5252 S_{5, 1, 1}) y^4 + (-60840 z^2 T_{3, 2, 2} + 12480 z S_{3, -2, 2}$$

$$- 2288 T_{3, 2, 1} + 14872 T_{3, 2, 2}) y^3 + \big(-3120 T_{5, -1, 1} z^3 + 14352 S_{5, 1, 1} z^2 + (4056 T_{5, -1, 1}$$

$$- 624\, T_{5,\ -1,\ 0}\big)\, z + 3744\, S_{5,\ 1,\ 1}\big)\, y^2 + \big(-50700\, z^4\, T_{3,\ 2,\ 2} - 24960\, z^3\, S_{3,\ -2,\ 2} + \big($$

$$-6864\, T_{3,\ 2,\ 1} + 44616\, T_{3,\ 2,\ 2}\big)\, z^2 + 1872\, T_{3,\ 2,\ 1} - 5148\, T_{3,\ 2,\ 2} - 416\, T_{3,\ 2,\ 0}\big)\, y$$

$$+ 9360\left(T_{5,\ -1,\ 1}\, z^3 + \frac{22}{9}\, S_{5,\ 1,\ 1}\, z^2 + \left(-\frac{26}{45}\, T_{5,\ -1,\ 1} + \frac{4}{45}\, T_{5,\ -1,\ 0}\right) z - \frac{8}{5}\, S_{5,\ 1,\ 1}\right) z^2\Big)\, x$$

$$- 1144\left(\frac{195}{44}\, T_{3,\ -2,\ 2}\, y^5 + \left(-\frac{15}{4}\, T_{5,\ 1,\ 1}\, z + \frac{101}{22}\, S_{5,\ -1,\ 1}\right) y^4 + \left(\frac{585}{22}\, T_{3,\ -2,\ 2}\, z^2\right.\right.$$

$$\left. + \frac{60}{11}\, z\, S_{3,\ 2,\ 2} + T_{3,\ -2,\ 1} - \frac{13}{2}\, T_{3,\ -2,\ 2}\right) y^3 + \left(-\frac{30}{11}\, T_{5,\ 1,\ 1}\, z^3 - \frac{138}{11}\, S_{5,\ -1,\ 1}\, z^2\right.$$

$$\left. + \left(\frac{39}{11}\, T_{5,\ 1,\ 1} - \frac{6}{11}\, T_{5,\ 1,\ 0}\right) z - \frac{36}{11}\, S_{5,\ -1,\ 1}\right) y^2 + \left(\frac{975}{44}\, T_{3,\ -2,\ 2}\, z^4 - \frac{120}{11}\, z^3\, S_{3,\ 2,\ 2}\right.$$

$$\left. + \left(3\, T_{3,\ -2,\ 1} - \frac{39}{2}\, T_{3,\ -2,\ 2}\right) z^2 - \frac{9}{11}\, T_{3,\ -2,\ 1} + \frac{9}{4}\, T_{3,\ -2,\ 2} + \frac{2}{11}\, T_{3,\ -2,\ 0}\right) y$$

$$\left. + \frac{90}{11}\, z^2\left(T_{5,\ 1,\ 1}\, z^3 - \frac{22}{9}\, S_{5,\ -1,\ 1}\, z^2 + \left(-\frac{26}{45}\, T_{5,\ 1,\ 1} + \frac{4}{45}\, T_{5,\ 1,\ 0}\right) z + \frac{8}{5}\, S_{5,\ -1,\ 1}\right)\right) y\Big)$$

$$\sqrt{3} + \Big(\Big(\big(\big(4095\, T_{5,\ -3,\ 1} + 1768\, S_{4,\ 3,\ 2}\big)\, z + 1014\, T_{4,\ -3,\ 1} + 1794\, S_{5,\ 3,\ 1}\big)\, x^5$$

$$- 3042\, y\left(\left(\frac{105}{26}\, T_{5,\ 3,\ 1} - \frac{68}{39}\, S_{4,\ -3,\ 2}\right) z + T_{4,\ 3,\ 1} - \frac{23}{13}\, S_{5,\ -3,\ 1}\right) x^4 + \big(\big(\big($$

$$-8190\, T_{5,\ -3,\ 1} - 3536\, S_{4,\ 3,\ 2}\big)\, z - 2028\, T_{4,\ -3,\ 1} - 3588\, S_{5,\ 3,\ 1}\big)\, y^2 + \big(9360\, T_{5,\ -3,\ 1}$$

$$- 12376\, S_{4,\ 3,\ 2}\big)\, z^3 + \big(3042\, T_{4,\ -3,\ 1} + 1872\, S_{5,\ 3,\ 1}\big)\, z^2 + \big(-4056\, T_{5,\ -3,\ 1} + 624\, T_{5,\ -3,\ 0}$$

$$- 104\, S_{4,\ 3,\ 1} + 1456\, S_{4,\ 3,\ 2}\big)\, z - 858\, T_{4,\ -3,\ 1} + 156\, T_{4,\ -3,\ 0} - 1248\, S_{5,\ 3,\ 1}\big)\, x^3$$

$$- 2028\left(\left(\left(\frac{105}{26}\, T_{5,\ 3,\ 1} - \frac{68}{39}\, S_{4,\ -3,\ 2}\right) z + T_{4,\ 3,\ 1} - \frac{23}{13}\, S_{5,\ -3,\ 1}\right) y^2 + \left(\frac{180}{13}\, T_{5,\ 3,\ 1}\right.\right.$$

$$\left. + \frac{238}{13}\, S_{4,\ -3,\ 2}\right) z^3 + \left(\frac{9}{2}\, T_{4,\ 3,\ 1} - \frac{36}{13}\, S_{5,\ -3,\ 1}\right) z^2 + \left(-6\, T_{5,\ 3,\ 1} + \frac{12}{13}\, T_{5,\ 3,\ 0}\right.$$

$$\left.\left. + \frac{2}{13}\, S_{4,\ -3,\ 1} - \frac{28}{13}\, S_{4,\ -3,\ 2}\right) z - \frac{33}{26}\, T_{4,\ 3,\ 1} + \frac{3}{13}\, T_{4,\ 3,\ 0} + \frac{24}{13}\, S_{5,\ -3,\ 1}\right) y\, x^2$$

$$- 3042\left(\left(\left(\frac{105}{26}\, T_{5,\ -3,\ 1} + \frac{68}{39}\, S_{4,\ 3,\ 2}\right) z + T_{4,\ -3,\ 1} + \frac{23}{13}\, S_{5,\ 3,\ 1}\right) y^2 + \left(\frac{120}{13}\, T_{5,\ -3,\ 1}\right.\right.$$

$$\left. - \frac{476}{39}\, S_{4,\ 3,\ 2}\right) z^3 + \left(3\, T_{4,\ -3,\ 1} + \frac{24}{13}\, S_{5,\ 3,\ 1}\right) z^2 + \left(-4\, T_{5,\ -3,\ 1} + \frac{8}{13}\, T_{5,\ -3,\ 0}\right.$$

$$\left.\left. - \frac{4}{39}\, S_{4,\ 3,\ 1} + \frac{56}{39}\, S_{4,\ 3,\ 2}\right) z - \frac{11}{13}\, T_{4,\ -3,\ 1} + \frac{2}{13}\, T_{4,\ -3,\ 0} - \frac{16}{13}\, S_{5,\ 3,\ 1}\right) y^2\, x$$

$$+ 1014\, y^3\left(\left(\left(\frac{105}{26}\, T_{5,\ 3,\ 1} - \frac{68}{39}\, S_{4,\ -3,\ 2}\right) z + T_{4,\ 3,\ 1} - \frac{23}{13}\, S_{5,\ -3,\ 1}\right) y^2 + \left(\frac{120}{13}\, T_{5,\ 3,\ 1}\right.\right.$$

$$\left. + \frac{476}{39}\, S_{4,\ -3,\ 2}\right) z^3 + \left(3\, T_{4,\ 3,\ 1} - \frac{24}{13}\, S_{5,\ -3,\ 1}\right) z^2 + \left(-4\, T_{5,\ 3,\ 1} + \frac{8}{13}\, T_{5,\ 3,\ 0}\right.$$

$$\left.\left. + \frac{4}{39}\, S_{4,\ -3,\ 1} - \frac{56}{39}\, S_{4,\ -3,\ 2}\right) z - \frac{11}{13}\, T_{4,\ 3,\ 1} + \frac{2}{13}\, T_{4,\ 3,\ 0} + \frac{16}{13}\, S_{5,\ -3,\ 1}\right)\Big)\, \sqrt{7}$$

$$+ \left(\left(15210\, T_{3,\,-3,\,2} + 90168\, S_{4,\,1,\,2} \right) z - 1014\, T_{4,\,-1,\,1} - 4680\, S_{3,\,3,\,2} \right) x^{5} - 45630\, \Big(\Big(T_{3,\,3,\,2}$$

$$- \frac{1156}{585}\, S_{4,\,-1,\,2} \Big) z - \frac{1}{45}\, T_{4,\,1,\,1} + \frac{4}{13}\, S_{3,\,-3,\,2} \Big) y\, x^{4} + \Big(\Big(\big(-30420\, T_{3,\,-3,\,2}$$

$$+ 180336\, S_{4,\,1,\,2} \big) z - 2028\, T_{4,\,-1,\,1} + 9360\, S_{3,\,3,\,2} \Big) y^{2} + \big(15210\, T_{3,\,-3,\,2}$$

$$+ 305864\, S_{4,\,1,\,2} \big) z^{3} + \left(1014\, T_{4,\,-1,\,1} - 14040\, S_{3,\,3,\,2} \right) z^{2} + \big(1716\, T_{3,\,-3,\,1} - 11154\, T_{3,\,-3,\,2}$$

$$+ 9048\, S_{4,\,1,\,1} - 126672\, S_{4,\,1,\,2} \big) z + 858\, T_{4,\,-1,\,1} - 156\, T_{4,\,-1,\,0} - 312\, S_{3,\,3,\,1}$$

$$+ 3744\, S_{3,\,3,\,2} \Big) x^{3} - 5148\, \Big(\Big(\Big(\frac{65}{11}\, T_{3,\,3,\,2} - \frac{1156}{33}\, S_{4,\,-1,\,2} \Big) z - \frac{13}{33}\, T_{4,\,1,\,1}$$

$$+ \frac{20}{11}\, S_{3,\,-3,\,2} \Big) y^{2} + \Big(\frac{195}{22}\, T_{3,\,3,\,2} - \frac{5882}{99}\, S_{4,\,-1,\,2} \Big) z^{3} + \Big(\frac{13}{66}\, T_{4,\,1,\,1} + \frac{90}{11}\, S_{3,\,-3,\,2} \Big) z^{2}$$

$$+ \Big(T_{3,\,3,\,1} - \frac{13}{2}\, T_{3,\,3,\,2} - \frac{58}{33}\, S_{4,\,-1,\,1} + \frac{812}{33}\, S_{4,\,-1,\,2} \Big) z + \frac{1}{6}\, T_{4,\,1,\,1} - \frac{1}{33}\, T_{4,\,1,\,0}$$

$$+ \frac{2}{11}\, S_{3,\,-3,\,1} - \frac{24}{11}\, S_{3,\,-3,\,2} \Big) y\, x^{2} + \Big(\Big(\big(-45630\, T_{3,\,-3,\,2} + 90168\, S_{4,\,1,\,2} \big) z$$

$$- 1014\, T_{4,\,-1,\,1} + 14040\, S_{3,\,3,\,2} \Big) y^{4} + \Big(\big(-45630\, T_{3,\,-3,\,2} + 305864\, S_{4,\,1,\,2} \big) z^{3}$$

$$+ \left(1014\, T_{4,\,-1,\,1} + 42120\, S_{3,\,3,\,2} \right) z^{2} + \big(-5148\, T_{3,\,-3,\,1} + 33462\, T_{3,\,-3,\,2} + 9048\, S_{4,\,1,\,1}$$

$$- 126672\, S_{4,\,1,\,2} \big) z + 858\, T_{4,\,-1,\,1} - 156\, T_{4,\,-1,\,0} + 936\, S_{3,\,3,\,1} - 11232\, S_{3,\,3,\,2} \Big) y^{2}$$

$$+ 6760\, \Big(\frac{1122}{65}\, z^{4}\, S_{4,\,1,\,2} + T_{4,\,-1,\,1}\, z^{3} + \Big(\frac{8}{5}\, S_{4,\,1,\,1} - \frac{112}{5}\, S_{4,\,1,\,2} \Big) z^{2} + \Big(-\frac{33}{65}\, T_{4,\,-1,\,1}$$

$$+ \frac{6}{65}\, T_{4,\,-1,\,0} \Big) z - \frac{12}{13}\, S_{4,\,1,\,1} + \frac{66}{13}\, S_{4,\,1,\,2} \Big) z \Big) x + 1716\, y\, \Big(\Big(\Big(\frac{195}{22}\, T_{3,\,3,\,2}$$

$$+ \frac{578}{11}\, S_{4,\,-1,\,2} \Big) z + \frac{13}{22}\, T_{4,\,1,\,1} + \frac{30}{11}\, S_{3,\,-3,\,2} \Big) y^{4} + \Big(\Big(\frac{195}{22}\, T_{3,\,3,\,2} + \frac{5882}{33}\, S_{4,\,-1,\,2} \Big) z^{3}$$

$$+ \Big(-\frac{13}{22}\, T_{4,\,1,\,1} + \frac{90}{11}\, S_{3,\,-3,\,2} \Big) z^{2} + \Big(T_{3,\,3,\,1} - \frac{13}{2}\, T_{3,\,3,\,2} + \frac{58}{11}\, S_{4,\,-1,\,1}$$

$$- \frac{812}{11}\, S_{4,\,-1,\,2} \Big) z - \frac{1}{2}\, T_{4,\,1,\,1} + \frac{1}{11}\, T_{4,\,1,\,0} + \frac{2}{11}\, S_{3,\,-3,\,1} - \frac{24}{11}\, S_{3,\,-3,\,2} \Big) y^{2} - \frac{130}{33}\, z\, \Big($$

$$-\frac{1122}{65}\, S_{4,\,-1,\,2}\, z^{4} + T_{4,\,1,\,1}\, z^{3} + \Big(-\frac{8}{5}\, S_{4,\,-1,\,1} + \frac{112}{5}\, S_{4,\,-1,\,2} \Big) z^{2} + \Big(-\frac{33}{65}\, T_{4,\,1,\,1}$$

$$+ \frac{6}{65}\, T_{4,\,1,\,0} \Big) z + \frac{12}{13}\, S_{4,\,-1,\,1} - \frac{66}{13}\, S_{4,\,-1,\,2} \Big) \Big) \Big) \sqrt{2} + \Big(\big(2340\, T_{5,\,-4,\,1}$$

$$- 3536\, S_{4,\,4,\,2} \big) x^{6} - 9360\, \Big(T_{5,\,4,\,1} + \frac{68}{45}\, S_{4,\,-4,\,2} \Big) y\, x^{5} + \Big(\big(-11700\, T_{5,\,-4,\,1}$$

$$+ 17680\, S_{4,\,4,\,2} \big) y^{2} + \left(7020\, T_{5,\,-4,\,1} - 10608\, S_{4,\,4,\,2} \right) z^{2} + \left(1352\, T_{4,\,-4,\,1} - 312\, S_{5,\,4,\,1} \right) z$$

$$- 2028\, T_{5,\,-4,\,1} + 312\, T_{5,\,-4,\,0} - 208\, S_{4,\,4,\,1} + 2912\, S_{4,\,4,\,2} \Big) x^{4} - 5408\, \Big(\Big(\frac{135}{26}\, T_{5,\,4,\,1}$$

$$+\frac{102}{13} S_{4,\,-4,\,2}\Big) z^2+\Big(T_{4,\,4,\,1}+\frac{3}{13} S_{5,\,-4,\,1}\Big) z-\frac{3}{2} T_{5,\,4,\,1}+\frac{3}{13} T_{5,\,4,\,0}+\frac{2}{13} S_{4,\,-4,\,1}$$

$$-\frac{28}{13} S_{4,\,-4,\,2}\Big) y x^3-8112 y^2 \Big(\Big(\frac{75}{52} T_{5,\,-4,\,1}-\frac{85}{39} S_{4,\,4,\,2}\Big) y^2+\Big(\frac{135}{26} T_{5,\,-4,\,1}$$

$$-\frac{102}{13} S_{4,\,4,\,2}\Big) z^2+\Big(T_{4,\,-4,\,1}-\frac{3}{13} S_{5,\,4,\,1}\Big) z-\frac{3}{2} T_{5,\,-4,\,1}+\frac{3}{13} T_{5,\,-4,\,0}-\frac{2}{13} S_{4,\,4,\,1}$$

$$+\frac{28}{13} S_{4,\,4,\,2}\Big) x^2+5408 \Big(\Big(\frac{45}{26} T_{5,\,4,\,1}+\frac{34}{13} S_{4,\,-4,\,2}\Big) y^2+\Big(\frac{135}{26} T_{5,\,4,\,1}$$

$$+\frac{102}{13} S_{4,\,-4,\,2}\Big) z^2+\Big(T_{4,\,4,\,1}+\frac{3}{13} S_{5,\,-4,\,1}\Big) z-\frac{3}{2} T_{5,\,4,\,1}+\frac{3}{13} T_{5,\,4,\,0}+\frac{2}{13} S_{4,\,-4,\,1}$$

$$-\frac{28}{13} S_{4,\,-4,\,2}\Big) y^3 x+1352 \Big(\Big(\frac{45}{26} T_{5,\,-4,\,1}-\frac{34}{13} S_{4,\,4,\,2}\Big) y^2+\Big(\frac{135}{26} T_{5,\,-4,\,1}$$

$$-\frac{102}{13} S_{4,\,4,\,2}\Big) z^2+\Big(T_{4,\,-4,\,1}-\frac{3}{13} S_{5,\,4,\,1}\Big) z-\frac{3}{2} T_{5,\,-4,\,1}+\frac{3}{13} T_{5,\,-4,\,0}-\frac{2}{13} S_{4,\,4,\,1}$$

$$+\frac{28}{13} S_{4,\,4,\,2}\Big) y^4\Big) \sqrt{7}+54808 x^6 S_{4,\,2,\,2}+109616 x^5 y S_{4,\,-2,\,2}+\big(54808 y^2 S_{4,\,2,\,2}$$

$$+180336 z^2 S_{4,\,2,\,2}+6760 z T_{4,\,-2,\,1}+4784 S_{4,\,2,\,1}-66976 S_{4,\,2,\,2}\big) x^4-13520 \Big($$

$$-\frac{1054}{65} y^2 S_{4,\,-2,\,2}-\frac{1734}{65} z^2 S_{4,\,-2,\,2}+T_{4,\,2,\,1} z-\frac{46}{65} S_{4,\,-2,\,1}+\frac{644}{65} S_{4,\,-2,\,2}\Big) y x^3+\big($$

$$-54808 y^4 S_{4,\,2,\,2}+26520 S_{4,\,2,\,2} z^4+16224 T_{4,\,-2,\,1} z^3+\big(5616 S_{4,\,2,\,1}-78624 S_{4,\,2,\,2}\big) z^2$$

$$+\big(-6864 T_{4,\,-2,\,1}+1248 T_{4,\,-2,\,0}\big) z-3120 S_{4,\,2,\,1}+17160 S_{4,\,2,\,2}\big) x^2-13520 \Big($$

$$-\frac{527}{65} y^4 S_{4,\,-2,\,2}+\Big(-\frac{1734}{65} z^2 S_{4,\,-2,\,2}+T_{4,\,2,\,1} z-\frac{46}{65} S_{4,\,-2,\,1}+\frac{644}{65} S_{4,\,-2,\,2}\Big) y^2$$

$$-\frac{51}{13} S_{4,\,-2,\,2} z^4+\frac{12}{5} T_{4,\,2,\,1} z^3+\Big(-\frac{54}{65} S_{4,\,-2,\,1}+\frac{756}{65} S_{4,\,-2,\,2}\Big) z^2+\Big(-\frac{66}{65} T_{4,\,2,\,1}$$

$$+\frac{12}{65} T_{4,\,2,\,0}\Big) z+\frac{6}{13} S_{4,\,-2,\,1}-\frac{33}{13} S_{4,\,-2,\,2}\Big) y x-6760 y^2 \Big(\frac{527}{65} y^4 S_{4,\,2,\,2}$$

$$+\Big(\frac{1734}{65} z^2 S_{4,\,2,\,2}+T_{4,\,-2,\,1} z+\frac{46}{65} S_{4,\,2,\,1}-\frac{644}{65} S_{4,\,2,\,2}\Big) y^2+\frac{51}{13} S_{4,\,2,\,2} z^4$$

$$+\frac{12}{5} T_{4,\,-2,\,1} z^3+\Big(\frac{54}{65} S_{4,\,2,\,1}-\frac{756}{65} S_{4,\,2,\,2}\Big) z^2+\Big(-\frac{66}{65} T_{4,\,-2,\,1}+\frac{12}{65} T_{4,\,-2,\,0}\Big) z$$

$$-\frac{6}{13} S_{4,\,2,\,1}+\frac{33}{13} S_{4,\,2,\,2}\Big)\Big) \sqrt{5}+\frac{1}{208} \Big(\Big(\big(5070 z T_{3,\,-1,\,2}+29640 S_{3,\,1,\,2}\big) x^5$$

$$-5070 \Big(T_{3,\,1,\,2} z-\frac{76}{13} S_{3,\,-1,\,2}\Big) y x^4+\big(\big(10140 z T_{3,\,-1,\,2}+59280 S_{3,\,1,\,2}\big) y^2$$

$$+35490 T_{3,\,-1,\,2} z^3+107640 z^2 S_{3,\,1,\,2}+\big(1716 T_{3,\,-1,\,1}-11154 T_{3,\,-1,\,2}\big) z+2808 S_{3,\,1,\,1}$$

$$-33696 S_{3,\,1,\,2}\big) x^3-1716 \Big(\Big(\frac{65}{11} T_{3,\,1,\,2} z-\frac{380}{11} S_{3,\,-1,\,2}\Big) y^2+\frac{455}{22} T_{3,\,1,\,2} z^3$$

$$-\frac{690}{11} z^2 S_{3,\,-1,\,2}+\Big(T_{3,\,1,\,1}-\frac{13}{2} T_{3,\,1,\,2}\Big) z-\frac{18}{11} S_{3,\,-1,\,1}+\frac{216}{11} S_{3,\,-1,\,2}\Big) y x^2$$

$$+ ((5070\, z\, T_{3,\,-1,\,2} + 29640\, S_{3,\,1,\,2})\, y^4 + (35490\, T_{3,\,-1,\,2}\, z^3 + 107640\, z^2\, S_{3,\,1,\,2}$$

$$+ (1716\, T_{3,\,-1,\,1} - 11154\, T_{3,\,-1,\,2})\, z + 2808\, S_{3,\,1,\,1} - 33696\, S_{3,\,1,\,2})\, y^2 + 30420\, T_{3,\,-1,\,2}\, z^5$$

$$+ 31200\, z^4\, S_{3,\,1,\,2} + (4576\, T_{3,\,-1,\,1} - 29744\, T_{3,\,-1,\,2})\, z^3 + (3744\, S_{3,\,1,\,1} - 44928\, S_{3,\,1,\,2})\, z^2$$

$$+ (-1872\, T_{3,\,-1,\,1} + 5148\, T_{3,\,-1,\,2} + 416\, T_{3,\,-1,\,0})\, z - 1664\, S_{3,\,1,\,1} + 7488\, S_{3,\,1,\,2})\, x$$

$$- 1716\, y\, \Big(\Big(\frac{65}{22}\, T_{3,\,1,\,2}\, z - \frac{190}{11}\, S_{3,\,-1,\,2}\Big)\, y^4 + \Big(\frac{455}{22}\, T_{3,\,1,\,2}\, z^3 - \frac{690}{11}\, z^2\, S_{3,\,-1,\,2}$$

$$+ \Big(T_{3,\,1,\,1} - \frac{13}{2}\, T_{3,\,1,\,2}\Big)\, z - \frac{18}{11}\, S_{3,\,-1,\,1} + \frac{216}{11}\, S_{3,\,-1,\,2}\Big)\, y^2 + \frac{195}{11}\, T_{3,\,1,\,2}\, z^5$$

$$- \frac{200}{11}\, z^4\, S_{3,\,-1,\,2} + \Big(\frac{8}{3}\, T_{3,\,1,\,1} - \frac{52}{3}\, T_{3,\,1,\,2}\Big)\, z^3 + \Big(-\frac{24}{11}\, S_{3,\,-1,\,1} + \frac{288}{11}\, S_{3,\,-1,\,2}\Big)\, z^2$$

$$+ \Big(-\frac{12}{11}\, T_{3,\,1,\,1} + 3\, T_{3,\,1,\,2} + \frac{8}{33}\, T_{3,\,1,\,0}\Big)\, z + \frac{32}{33}\, S_{3,\,-1,\,1} - \frac{48}{11}\, S_{3,\,-1,\,2}\Big)\Big)\, \sqrt{2} + \Big(($$

$$-28860\, z\, S_{6,\,1,\,1} + 130\, T_{6,\,-1,\,0})\, x^5 - 28860\, y\, \Big(S_{6,\,-1,\,1}\, z + \frac{1}{222}\, T_{6,\,1,\,0}\Big)\, x^4 + \Big(($$

$$-57720\, z\, S_{6,\,1,\,1} + 260\, T_{6,\,-1,\,0})\, y^2 + 6240\, \Big(S_{6,\,1,\,1}\, z^2 - \frac{1}{4}\, z\, T_{6,\,-1,\,0} + \frac{7}{2}\, S_{6,\,1,\,1}\Big)\, z\Big)\, x^3$$

$$- 57720\, \Big(\Big(S_{6,\,-1,\,1}\, z + \frac{1}{222}\, T_{6,\,1,\,0}\Big)\, y^2 - \frac{4}{37}\, \Big(S_{6,\,-1,\,1}\, z^2 + \frac{1}{4}\, z\, T_{6,\,1,\,0}$$

$$+ \frac{7}{2}\, S_{6,\,-1,\,1}\Big)\, z\Big)\, y\, x^2 + \Big((-28860\, z\, S_{6,\,1,\,1} + 130\, T_{6,\,-1,\,0})\, y^4 + 6240\, \Big(S_{6,\,1,\,1}\, z^2$$

$$- \frac{1}{4}\, z\, T_{6,\,-1,\,0} + \frac{7}{2}\, S_{6,\,1,\,1}\Big)\, z\, y^2 + 41184\, S_{6,\,1,\,1}\, z^5 + 1040\, z^4\, T_{6,\,-1,\,0} - 29120\, S_{6,\,1,\,1}\, z^3\Big)\, x$$

$$- 28860\, y\, \Big(\Big(S_{6,\,-1,\,1}\, z + \frac{1}{222}\, T_{6,\,1,\,0}\Big)\, y^4 - \frac{8}{37}\, \Big(S_{6,\,-1,\,1}\, z^2 + \frac{1}{4}\, z\, T_{6,\,1,\,0}$$

$$+ \frac{7}{2}\, S_{6,\,-1,\,1}\Big)\, z\, y^2 - \frac{264}{185}\, z^3\, \Big(S_{6,\,-1,\,1}\, z^2 - \frac{5}{198}\, z\, T_{6,\,1,\,0} - \frac{70}{99}\, S_{6,\,-1,\,1}\Big)\Big)\Big)\, \sqrt{7}$$

$$- 39780\, x^6\, S_{2,\,2,\,3} + (-79560\, y\, S_{2,\,-2,\,3} + 198900\, z\, S_{2,\,1,\,3} + 3718\, T_{2,\,-1,\,2})\, x^5 + ($$

$$-39780\, y^2\, S_{2,\,2,\,3} + (198900\, z\, S_{2,\,-1,\,3} - 3718\, T_{2,\,1,\,2})\, y - 238680\, z^2\, S_{2,\,2,\,3}$$

$$+ 14872\, z\, T_{2,\,-2,\,2} - 5408\, S_{2,\,2,\,2} + 54600\, S_{2,\,2,\,3})\, x^4 + (-159120\, y^3\, S_{2,\,-2,\,3}$$

$$+ (397800\, z\, S_{2,\,1,\,3} + 7436\, T_{2,\,-1,\,2})\, y^2 + (-477360\, z^2\, S_{2,\,-2,\,3} - 29744\, z\, T_{2,\,2,\,2}$$

$$- 10816\, S_{2,\,-2,\,2} + 109200\, S_{2,\,-2,\,3})\, y + 79560\, z^3\, S_{2,\,1,\,3} + 22308\, z^2\, T_{2,\,-1,\,2}$$

$$+ (16224\, S_{2,\,1,\,2} - 163800\, S_{2,\,1,\,3})\, z + 936\, T_{2,\,-1,\,1} - 5148\, T_{2,\,-1,\,2})\, x^3 + (39780\, y^4\, S_{2,\,2,\,3}$$

$$+ (397800\, z\, S_{2,\,-1,\,3} - 7436\, T_{2,\,1,\,2})\, y^3 + (79560\, z^3\, S_{2,\,-1,\,3} - 22308\, T_{2,\,1,\,2}\, z^2$$

$$+\left(16224\,S_{2,\,-1,\,2}-163800\,S_{2,\,-1,\,3}\right)z-936\,T_{2,\,1,\,1}+5148\,T_{2,\,1,\,2}\Big)\,y-198900\,z^{4}\,S_{2,\,2,\,3}$$

$$+14872\,z^{3}\,T_{2,\,-2,\,2}+\left(-16224\,S_{2,\,2,\,2}+163800\,S_{2,\,2,\,3}\right)z^{2}+\Big(1872\,T_{2,\,-2,\,1}$$

$$-10296\,T_{2,\,-2,\,2}\Big)\,z-416\,S_{2,\,2,\,1}+4160\,S_{2,\,2,\,2}-17316\,S_{2,\,2,\,3}\Big)\,x^{2}+\Big(-79560\,y^{5}\,S_{2,\,-2,\,3}$$

$$+\left(198900\,z\,S_{2,\,1,\,3}+3718\,T_{2,\,-1,\,2}\right)y^{4}+\Big(-477360\,z^{2}\,S_{2,\,-2,\,3}-29744\,z\,T_{2,\,2,\,2}$$

$$-10816\,S_{2,\,-2,\,2}+109200\,S_{2,\,-2,\,3}\Big)\,y^{3}+\Big(79560\,z^{3}\,S_{2,\,1,\,3}+22308\,z^{2}\,T_{2,\,-1,\,2}$$

$$+\left(16224\,S_{2,\,1,\,2}-163800\,S_{2,\,1,\,3}\right)z+936\,T_{2,\,-1,\,1}-5148\,T_{2,\,-1,\,2}\Big)\,y^{2}+\Big($$

$$-397800\,S_{2,\,-2,\,3}\,z^{4}-29744\,T_{2,\,2,\,2}\,z^{3}+\left(-32448\,S_{2,\,-2,\,2}+327600\,S_{2,\,-2,\,3}\right)z^{2}+\Big($$

$$-3744\,T_{2,\,2,\,1}+20592\,T_{2,\,2,\,2}\Big)\,z-832\,S_{2,\,-2,\,1}+8320\,S_{2,\,-2,\,2}-34632\,S_{2,\,-2,\,3}\Big)\,y$$

$$-119340\,z^{5}\,S_{2,\,1,\,3}+18590\,z^{4}\,T_{2,\,-1,\,2}+\left(-5408\,S_{2,\,1,\,2}+54600\,S_{2,\,1,\,3}\right)z^{3}$$

$$+\left(2808\,T_{2,\,-1,\,1}-15444\,T_{2,\,-1,\,2}\right)z^{2}+\left(416\,S_{2,\,1,\,1}-4160\,S_{2,\,1,\,2}+17316\,S_{2,\,1,\,3}\right)z$$

$$-728\,T_{2,\,-1,\,1}+1638\,T_{2,\,-1,\,2}+208\,T_{2,\,-1,\,0}\Big)\,x-1872\,y\,\Bigg(-\frac{85}{4}\,y^{5}\,S_{2,\,2,\,3}+\Bigg(\frac{143}{72}\,T_{2,\,1,\,2}$$

$$-\frac{425}{4}\,z\,S_{2,\,-1,\,3}\Bigg)\,y^{4}+\Bigg(-\frac{26}{9}\,S_{2,\,2,\,2}+\frac{175}{6}\,S_{2,\,2,\,3}+\frac{143}{18}\,z\,T_{2,\,-2,\,2}$$

$$-\frac{255}{2}\,z^{2}\,S_{2,\,2,\,3}\Bigg)\,y^{3}+\Bigg(-\frac{85}{2}\,z^{3}\,S_{2,\,-1,\,3}+\frac{143}{12}\,T_{2,\,1,\,2}\,z^{2}+\Bigg(-\frac{26}{3}\,S_{2,\,-1,\,2}$$

$$+\frac{175}{2}\,S_{2,\,-1,\,3}\Bigg)\,z+\frac{1}{2}\,T_{2,\,1,\,1}-\frac{11}{4}\,T_{2,\,1,\,2}\Bigg)\,y^{2}+\Bigg(-\frac{425}{4}\,z^{4}\,S_{2,\,2,\,3}+\frac{143}{18}\,z^{3}\,T_{2,\,-2,\,2}$$

$$+\Bigg(-\frac{26}{3}\,S_{2,\,2,\,2}+\frac{175}{2}\,S_{2,\,2,\,3}\Bigg)\,z^{2}+\Bigg(T_{2,\,-2,\,1}-\frac{11}{2}\,T_{2,\,-2,\,2}\Bigg)\,z-\frac{2}{9}\,S_{2,\,2,\,1}+\frac{20}{9}\,S_{2,\,2,\,2}$$

$$-\frac{37}{4}\,S_{2,\,2,\,3}\Bigg)\,y+\frac{255}{4}\,z^{5}\,S_{2,\,-1,\,3}+\frac{715}{72}\,T_{2,\,1,\,2}\,z^{4}+\Bigg(\frac{26}{9}\,S_{2,\,-1,\,2}-\frac{175}{6}\,S_{2,\,-1,\,3}\Bigg)\,z^{3}$$

$$+\Bigg(\frac{3}{2}\,T_{2,\,1,\,1}-\frac{33}{4}\,T_{2,\,1,\,2}\Bigg)\,z^{2}+\Bigg(-\frac{37}{4}\,S_{2,\,-1,\,3}-\frac{2}{9}\,S_{2,\,-1,\,1}+\frac{20}{9}\,S_{2,\,-1,\,2}\Bigg)\,z$$

$$-\frac{7}{18}\,T_{2,\,1,\,1}+\frac{7}{8}\,T_{2,\,1,\,2}+\frac{1}{9}\,T_{2,\,1,\,0}\Bigg)\Bigg)\,\sqrt{3}+\frac{1}{208}\,\Big(7488\,x^{6}\,S_{6,\,4,\,1}+29952\,x^{5}\,y\,S_{6,\,-4,\,1}$$

$$+\left(-37440\,y^{2}\,S_{6,\,4,\,1}+7020\,z^{2}\,S_{6,\,4,\,1}+3120\,z\,T_{6,\,-4,\,0}-5460\,S_{6,\,4,\,1}\right)x^{4}$$

$$+28080\,y\,\Bigg(S_{6,\,-4,\,1}\,z^{2}-\frac{4}{9}\,z\,T_{6,\,4,\,0}-\frac{7}{9}\,S_{6,\,-4,\,1}\Bigg)\,x^{3}-37440\,y^{2}\,\Bigg(S_{6,\,4,\,1}\,y^{2}+\frac{9}{8}\,S_{6,\,4,\,1}\,z^{2}$$

$$+\frac{1}{2}\,z\,T_{6,\,-4,\,0}-\frac{7}{8}\,S_{6,\,4,\,1}\Bigg)\,x^{2}-29952\,\Bigg(S_{6,\,-4,\,1}\,y^{2}+\frac{15}{16}\,S_{6,\,-4,\,1}\,z^{2}-\frac{5}{12}\,z\,T_{6,\,4,\,0}$$

$$-\frac{35}{48}\,S_{6,\,-4,\,1}\Bigg)\,y^{3}\,x+7488\,\Bigg(S_{6,\,4,\,1}\,y^{2}+\frac{15}{16}\,S_{6,\,4,\,1}\,z^{2}+\frac{5}{12}\,z\,T_{6,\,-4,\,0}-\frac{35}{48}\,S_{6,\,4,\,1}\Bigg)\,y^{4}\Big)$$

$$\sqrt{7}+\frac{1}{208}\left(21840 S_{6,0,1}+159120 S_{2,0,3}-106080 S_{4,0,2}\right) x^6+\frac{1}{208}\left(55770 z T_{1,-1,3}\right.$$

$$\left.-30420 S_{1,1,3}\right) x^5+\frac{1}{208}\left(\left(477360 S_{2,0,3}-318240 S_{4,0,2}+65520 S_{6,0,1}\right) y^2+\left(\right.\right.$$

$$-55770 z T_{1,1,3}-30420 S_{1,-1,3}\big) y+\big(1074060 S_{2,0,3}-159120 S_{4,0,2}$$

$$-147420 S_{6,0,1}\big) z^2+\left(182520 S_{1,0,3}+56160 S_{3,0,2}-51480 S_{5,0,1}\right) z+24336 S_{2,0,2}$$

$$-245700 S_{2,0,3}-9360 S_{4,0,1}+131040 S_{4,0,2}-16380 S_{6,0,1}\big) x^4$$

$$+\frac{1}{208}\left(\left(111540 z T_{1,-1,3}-60840 S_{1,1,3}\right) y^2-31500 y+111540 z^3 T_{1,-1,3}\right.$$

$$-182520 z^2 S_{1,1,3}+\left(10296 T_{1,-1,2}-66924 T_{1,-1,3}\right) z-4576 S_{1,1,2}+39208 S_{1,1,3}\big) x^3$$

$$+\frac{1}{208}\left(\left(477360 S_{2,0,3}-318240 S_{4,0,2}+65520 S_{6,0,1}\right) y^4+\left(-111540 z T_{1,1,3}\right.\right.$$

$$-60840 S_{1,-1,3}\big) y^3+\left(\left(2148120 S_{2,0,3}-318240 S_{4,0,2}-294840 S_{6,0,1}\right) z^2\right.$$

$$+\left(365040 S_{1,0,3}+112320 S_{3,0,2}-102960 S_{5,0,1}\right) z+48672 S_{2,0,2}-491400 S_{2,0,3}$$

$$-18720 S_{4,0,1}+262080 S_{4,0,2}-32760 S_{6,0,1}\big) y^2+\big(-111540 z^3 T_{1,1,3}$$

$$-182520 z^2 S_{1,-1,3}+\left(-10296 T_{1,1,2}+66924 T_{1,1,3}\right) z-4576 S_{1,-1,2}$$

$$+39208 S_{1,-1,3}\big) y+\left(1193400 S_{2,0,3}+442000 S_{4,0,2}-131040 S_{6,0,1}\right) z^4$$

$$+\left(243360 S_{1,0,3}+262080 S_{3,0,2}-24960 S_{5,0,1}\right) z^3+\big(97344 S_{2,0,2}-982800 S_{2,0,3}$$

$$+6240 S_{4,0,1}-87360 S_{4,0,2}+131040 S_{6,0,1}\big) z^2+\big(-156832 S_{1,0,3}+7488 S_{3,0,1}$$

$$-89856 S_{3,0,2}+37440 S_{5,0,1}+18304 S_{1,0,2}\big) z+2496 S_{2,0,1}-24960 S_{2,0,2}$$

$$+103896 S_{2,0,3}+6240 S_{4,0,1}-34320 S_{4,0,2}\big) x^2+\frac{1}{208}\left(\left(55770 z T_{1,-1,3}\right.\right.$$

$$-30420 S_{1,1,3}\big) y^4+31500 y^3+\big(111540 z^3 T_{1,-1,3}-182520 z^2 S_{1,1,3}+\big(10296 T_{1,-1,2}$$

$$-66924 T_{1,-1,3}\big) z-4576 S_{1,1,2}+39208 S_{1,1,3}\big) y^2+55770 z^5 T_{1,-1,3}$$

$$-152100 z^4 S_{1,1,3}+\left(10296 T_{1,-1,2}-66924 T_{1,-1,3}\right) z^3+\big(-13728 S_{1,1,2}$$

$$+117624 S_{1,1,3}\big) z^2+\left(1456 T_{1,-1,1}-6552 T_{1,-1,2}+18018 T_{1,-1,3}\right) z-416 S_{1,1,1}$$

$$+3328 S_{1,1,2}-11284 S_{1,1,3}\big) x+\frac{1}{208}\big(21840 S_{6,0,1}+159120 S_{2,0,3}$$

$$- 106080\, S_{4,\,0,\,2}\big)\, y^6 + \frac{1}{208}\,\big(-55770\, z\, T_{1,\,1,\,3} - 30420\, S_{1,\,-1,\,3}\big)\, y^5$$
$$+ \frac{1}{208}\,\Big(\big(1074060\, S_{2,\,0,\,3} - 159120\, S_{4,\,0,\,2} - 147420\, S_{6,\,0,\,1}\big)\, z^2 + \big(182520\, S_{1,\,0,\,3}$$
$$+ 56160\, S_{3,\,0,\,2} - 51480\, S_{5,\,0,\,1}\big)\, z + 24336\, S_{2,\,0,\,2} - 245700\, S_{2,\,0,\,3} - 9360\, S_{4,\,0,\,1}$$
$$+ 131040\, S_{4,\,0,\,2} - 16380\, S_{6,\,0,\,1}\Big)\, y^4 + \frac{1}{208}\,\Big(-111540\, z^3\, T_{1,\,1,\,3} - 182520\, z^2\, S_{1,\,-1,\,3} + \big($$
$$-10296\, T_{1,\,1,\,2} + 66924\, T_{1,\,1,\,3}\big)\, z - 4576\, S_{1,\,-1,\,2} + 39208\, S_{1,\,-1,\,3}\Big)\, y^3$$
$$+ \frac{1}{208}\,\Big(\big(1193400\, S_{2,\,0,\,3} + 442000\, S_{4,\,0,\,2} - 131040\, S_{6,\,0,\,1}\big)\, z^4 + \big(243360\, S_{1,\,0,\,3}$$
$$+ 262080\, S_{3,\,0,\,2} - 24960\, S_{5,\,0,\,1}\big)\, z^3 + \big(97344\, S_{2,\,0,\,2} - 982800\, S_{2,\,0,\,3} + 6240\, S_{4,\,0,\,1}$$
$$- 87360\, S_{4,\,0,\,2} + 131040\, S_{6,\,0,\,1}\big)\, z^2 + \big(-156832\, S_{1,\,0,\,3} + 7488\, S_{3,\,0,\,1} - 89856\, S_{3,\,0,\,2}$$
$$+ 37440\, S_{5,\,0,\,1} + 18304\, S_{1,\,0,\,2}\big)\, z + 2496\, S_{2,\,0,\,1} - 24960\, S_{2,\,0,\,2} + 103896\, S_{2,\,0,\,3}$$
$$+ 6240\, S_{4,\,0,\,1} - 34320\, S_{4,\,0,\,2}\Big)\, y^2 + \frac{1}{208}\,\Big(-55770\, z^5\, T_{1,\,1,\,3} - 152100\, z^4\, S_{1,\,-1,\,3} + \big($$
$$-10296\, T_{1,\,1,\,2} + 66924\, T_{1,\,1,\,3}\big)\, z^3 + \big(-13728\, S_{1,\,-1,\,2} + 117624\, S_{1,\,-1,\,3}\big)\, z^2 + \big($$
$$-1456\, T_{1,\,1,\,1} + 6552\, T_{1,\,1,\,2} - 18018\, T_{1,\,1,\,3}\big)\, z - 416\, S_{1,\,-1,\,1} + 3328\, S_{1,\,-1,\,2}$$
$$- 11284\, S_{1,\,-1,\,3}\Big)\, y + \frac{1}{208}\,\big(278460\, S_{2,\,0,\,3} + 247520\, S_{4,\,0,\,2} + 61152\, S_{6,\,0,\,1}\big)\, z^6$$
$$+ \frac{1}{208}\,\big(60840\, S_{1,\,0,\,3} + 112320\, S_{3,\,0,\,2} + 37440\, S_{5,\,0,\,1}\big)\, z^5 + \frac{1}{208}\,\big(40560\, S_{2,\,0,\,2}$$
$$- 409500\, S_{2,\,0,\,3} + 20800\, S_{4,\,0,\,1} - 291200\, S_{4,\,0,\,2} - 43680\, S_{6,\,0,\,1}\big)\, z^4 + \frac{1}{208}\,\big($$
$$-78416\, S_{1,\,0,\,3} + 9984\, S_{3,\,0,\,1} - 119808\, S_{3,\,0,\,2} - 24960\, S_{5,\,0,\,1} + 9152\, S_{1,\,0,\,2}\big)\, z^3$$
$$+ \frac{1}{208}\,\big(3744\, S_{2,\,0,\,1} - 37440\, S_{2,\,0,\,2} + 155844\, S_{2,\,0,\,3} - 12480\, S_{4,\,0,\,1} + 68640\, S_{4,\,0,\,2}\big)\, z^2$$
$$+ \frac{1}{208}\,\big(22568\, S_{1,\,0,\,3} - 4992\, S_{3,\,0,\,1} + 22464\, S_{3,\,0,\,2} + 832\, S_{1,\,0,\,1} - 6656\, S_{1,\,0,\,2}\big)\, z$$
$$- 6\, S_{2,\,0,\,1} + 21\, S_{2,\,0,\,2} - \frac{189}{4}\, S_{2,\,0,\,3}\Big)\, \bar{e}_z$$

```
> constraints := `union`(seq({coeffs(collect(eqn[i], [x,y,z],
  distributed), [x,y,z])}, i=1..3)):
> nops(constraints); nops(variables);
```

252

167 **(30)**

```
> sol1 := solve(constraints, variables);
```

$$sol1 := \left\{S_{1,\,-1,\,1}=0,\ S_{1,\,-1,\,2}=0,\ S_{1,\,-1,\,3}=0,\ S_{1,\,0,\,1}=0,\ S_{1,\,0,\,2}=0,\ S_{1,\,0,\,3}=0,\ S_{1,\,1,\,1}=0,\ S_{1,\,1,\,2}\right. \tag{31}$$

$$=0,\ S_{1,\,1,\,3}=0,\ S_{2,\,-2,\,1}=0,\ S_{2,\,-2,\,2}=0,\ S_{2,\,-2,\,3}=0,\ S_{2,\,-1,\,1}=0,\ S_{2,\,-1,\,2}=0,\ S_{2,\,-1,\,3}=0,$$

$$S_{2,\,0,\,1}=0,\ S_{2,\,0,\,2}=0,\ S_{2,\,0,\,3}=0,\ S_{2,\,1,\,1}=0,\ S_{2,\,1,\,2}=0,\ S_{2,\,1,\,3}=0,\ S_{2,\,2,\,1}=0,\ S_{2,\,2,\,2}=0,\ S_{2,\,2,\,3}$$

$$=0,\ S_{3,\,-3,\,1}=0,\ S_{3,\,-3,\,2}=0,\ S_{3,\,-2,\,1}=0,\ S_{3,\,-2,\,2}=0,\ S_{3,\,-1,\,1}=0,\ S_{3,\,-1,\,2}=0,\ S_{3,\,0,\,1}=0,$$

$$S_{3,\,0,\,2}=0,\ S_{3,\,1,\,1}=0,\ S_{3,\,1,\,2}=0,\ S_{3,\,2,\,1}=0,\ S_{3,\,2,\,2}=0,\ S_{3,\,3,\,1}=0,\ S_{3,\,3,\,2}=0,\ S_{4,\,-4,\,1}$$

$$=\frac{45}{208}\sqrt{7}\,\sqrt{5},\ S_{4,\,-4,\,2}=0,\ S_{4,\,-3,\,1}=0,\ S_{4,\,-3,\,2}=0,\ S_{4,\,-2,\,1}=0,\ S_{4,\,-2,\,2}=0,\ S_{4,\,-1,\,1}=0,$$

$$S_{4,\,-1,\,2}=0,\ S_{4,\,0,\,1}=0,\ S_{4,\,0,\,2}=0,\ S_{4,\,1,\,1}=0,\ S_{4,\,1,\,2}=0,\ S_{4,\,2,\,1}=0,\ S_{4,\,2,\,2}=0,\ S_{4,\,3,\,1}=0,$$

$$S_{4,\,3,\,2}=0,\ S_{4,\,4,\,1}=0,\ S_{4,\,4,\,2}=0,\ S_{5,\,-4,\,1}=0,\ S_{5,\,-3,\,1}=0,\ S_{5,\,-2,\,1}=0,\ S_{5,\,-1,\,1}=0,\ S_{5,\,0,\,1}=0,$$

$$S_{5,\,1,\,1}=0,\ S_{5,\,2,\,1}=0,\ S_{5,\,3,\,1}=0,\ S_{5,\,4,\,1}=0,\ S_{6,\,-4,\,1}=0,\ S_{6,\,-3,\,1}=0,\ S_{6,\,-2,\,1}=0,\ S_{6,\,-1,\,1}=0,$$

$$S_{6,\,0,\,1}=0,\ S_{6,\,1,\,1}=0,\ S_{6,\,2,\,1}=0,\ S_{6,\,3,\,1}=0,\ S_{6,\,4,\,1}=0,\ T_{1,\,-1,\,0}=0,\ T_{1,\,-1,\,1}=0,\ T_{1,\,-1,\,2}=0,$$

$$T_{1,\,-1,\,3}=0,\ T_{1,\,0,\,0}=T_{1,\,0,\,0},\ T_{1,\,0,\,1}=\frac{675}{52}-\frac{12}{7}\,T_{3,\,0,\,0},\ T_{1,\,0,\,2}=-\frac{3675}{572}+\frac{24}{11}\,T_{5,\,0,\,0},$$

$$T_{1,\,0,\,3}=0,\ T_{1,\,1,\,0}=0,\ T_{1,\,1,\,1}=0,\ T_{1,\,1,\,2}=0,\ T_{1,\,1,\,3}=0,\ T_{2,\,-2,\,0}=0,\ T_{2,\,-2,\,1}=0,\ T_{2,\,-2,\,2}=0,$$

$$T_{2,\,-1,\,0}=0,\ T_{2,\,-1,\,1}=0,\ T_{2,\,-1,\,2}=0,\ T_{2,\,0,\,0}=0,\ T_{2,\,0,\,1}=0,\ T_{2,\,0,\,2}=0,\ T_{2,\,1,\,0}=0,\ T_{2,\,1,\,1}=0,$$

$$T_{2,\,1,\,2}=0,\ T_{2,\,2,\,0}=0,\ T_{2,\,2,\,1}=0,\ T_{2,\,2,\,2}=0,\ T_{3,\,-3,\,0}=0,\ T_{3,\,-3,\,1}=0,\ T_{3,\,-3,\,2}=0,\ T_{3,\,-2,\,0}=0,$$

$$T_{3,\,-2,\,1}=0,\ T_{3,\,-2,\,2}=0,\ T_{3,\,-1,\,0}=0,\ T_{3,\,-1,\,1}=0,\ T_{3,\,-1,\,2}=0,\ T_{3,\,0,\,0}=T_{3,\,0,\,0},\ T_{3,\,0,\,1}=-\frac{525}{1144}$$

$$-\frac{14}{11}\,T_{5,\,0,\,0},\ T_{3,\,0,\,2}=0,\ T_{3,\,1,\,0}=0,\ T_{3,\,1,\,1}=0,\ T_{3,\,1,\,2}=0,\ T_{3,\,2,\,0}=0,\ T_{3,\,2,\,1}=0,\ T_{3,\,2,\,2}=0,$$

$$T_{3,\,3,\,0}=0,\ T_{3,\,3,\,1}=0,\ T_{3,\,3,\,2}=0,\ T_{4,\,-4,\,0}=0,\ T_{4,\,-4,\,1}=0,\ T_{4,\,-3,\,0}=0,\ T_{4,\,-3,\,1}=0,\ T_{4,\,-2,\,0}$$

$$=0,\ T_{4,\,-2,\,1}=0,\ T_{4,\,-1,\,0}=0,\ T_{4,\,-1,\,1}=0,\ T_{4,\,0,\,0}=0,\ T_{4,\,0,\,1}=0,\ T_{4,\,1,\,0}=0,\ T_{4,\,1,\,1}=0,\ T_{4,\,2,\,0}$$

$$=0,\ T_{4,\,2,\,1}=0,\ T_{4,\,3,\,0}=0,\ T_{4,\,3,\,1}=0,\ T_{4,\,4,\,0}=0,\ T_{4,\,4,\,1}=0,\ T_{5,\,-4,\,0}=0,\ T_{5,\,-4,\,1}=0,\ T_{5,\,-3,\,0}$$

$$=0,\ T_{5,\,-3,\,1}=0,\ T_{5,\,-2,\,0}=0,\ T_{5,\,-2,\,1}=0,\ T_{5,\,-1,\,0}=0,\ T_{5,\,-1,\,1}=0,\ T_{5,\,0,\,0}=T_{5,\,0,\,0},\ T_{5,\,0,\,1}=0,$$

$$T_{5,\,1,\,0}=0,\ T_{5,\,1,\,1}=0,\ T_{5,\,2,\,0}=0,\ T_{5,\,2,\,1}=0,\ T_{5,\,3,\,0}=0,\ T_{5,\,3,\,1}=0,\ T_{5,\,4,\,0}=-\frac{45}{52}\sqrt{7}\,\sqrt{5},$$

$$T_{5,\,4,\,1}=0,\ T_{6,\,-4,\,0}=0,\ T_{6,\,-3,\,0}=0,\ T_{6,\,-2,\,0}=0,\ T_{6,\,-1,\,0}=0,\ T_{6,\,0,\,0}=0,\ T_{6,\,1,\,0}=0,\ T_{6,\,2,\,0}=0,$$

$T_{6,3,0} = 0, T_{6,4,0} = 0\}$

> # set geostrophic component to zero to remove degeneracy

```
> u_soln := simplify(subs(sol1, u_cart));
```

$$u\_soln := -\frac{315}{8} y \left(\left(T_{5,0,0} - \frac{75}{26} \right) y^4 + \left(2 x^2 T_{5,0,0} - \frac{50}{13} z^2 - \frac{4}{21} T_{3,0,0} - \frac{12}{11} T_{5,0,0} + \frac{590}{143} \right) y^2 + \left(T_{5,0,0} - \frac{25}{26} \right) x^4 + \left(-\frac{75}{13} z^2 - \frac{4}{21} T_{3,0,0} - \frac{12}{11} T_{5,0,0} + \frac{315}{143} \right) x^2 - \frac{125}{52} z^4 + \frac{105}{26} z^2 + \frac{8}{315} T_{1,0,0} + \frac{16}{147} T_{3,0,0} + \frac{8}{33} T_{5,0,0} - \frac{18475}{12012} \right) \bar{e}_x + \frac{315}{8} x \left(\left(T_{5,0,0} - \frac{75}{26} \right) x^4 + \left(2 y^2 T_{5,0,0} - \frac{50}{13} z^2 - \frac{4}{21} T_{3,0,0} - \frac{12}{11} T_{5,0,0} + \frac{590}{143} \right) x^2 + \left(T_{5,0,0} - \frac{25}{26} \right) y^4 + \left(-\frac{75}{13} z^2 - \frac{4}{21} T_{3,0,0} - \frac{12}{11} T_{5,0,0} + \frac{315}{143} \right) y^2 - \frac{125}{52} z^4 + \frac{105}{26} z^2 + \frac{8}{315} T_{1,0,0} + \frac{16}{147} T_{3,0,0} + \frac{8}{33} T_{5,0,0} - \frac{18475}{12012} \right) \bar{e}_y - \frac{7875}{52} x y z \left(x^2 - y^2 \right) \bar{e}_z \quad (32)$$

```
> u_cyl := simplify(MapToBasis(u_soln, cylindrical[s,phi,z]));
```

$$u\_cyl := -\frac{7875}{52} s^3 \sin(\phi) \left(s^2 - z^2 - 1 \right) \left(\cos(\phi)^2 - \frac{1}{2} \right) \cos(\phi) \bar{e}_s + \frac{315}{8} s \left(-\frac{100}{13} s^2 \left(s^2 - \frac{1}{2} z^2 - \frac{1}{2} \right) \cos(\phi)^4 + \frac{100}{13} s^2 \left(s^2 - \frac{1}{2} z^2 - \frac{1}{2} \right) \cos(\phi)^2 + \left(T_{5,0,0} - \frac{75}{26} \right) s^4 + \left(-\frac{50}{13} z^2 - \frac{4}{21} T_{3,0,0} - \frac{12}{11} T_{5,0,0} + \frac{590}{143} \right) s^2 - \frac{125}{52} z^4 + \frac{105}{26} z^2 + \frac{8}{315} T_{1,0,0} + \frac{16}{147} T_{3,0,0} + \frac{8}{33} T_{5,0,0} - \frac{18475}{12012} \right) \bar{e}_\phi - \frac{7875}{52} s^4 z \left(2 \cos(\phi)^2 - 1 \right) \cos(\phi) \sin(\phi) \bar{e}_z \quad (33)$$

> $SetCoordinates(cylindrical[s, \mathrm{phi}, z])$

$$cylindrical_{s,\phi,z} \quad (34)$$

```
> geostrophic_component := simplify((int(int(u_cyl[2], phi = 0 .. 2*Pi), z = -sqrt(-s^2+1) .. sqrt(-s^2+1)))/(4*Pi*sqrt(-s^2+1)))
```

$$geostrophic\_component := \frac{315}{8} s \left(\left(T_{5,0,0} - \frac{25}{26} \right) s^4 + \left(-\frac{4}{21} T_{3,0,0} - \frac{12}{11} T_{5,0,0} + \frac{20}{11} \right) s^2 + \frac{8}{315} T_{1,0,0} + \frac{16}{147} T_{3,0,0} + \frac{8}{33} T_{5,0,0} - \frac{2020}{3003} \right) \quad (35)$$

```
> u_cyl := simplify(u_cyl - VectorField([0, geostrophic_component, 0])); #remove geostrophic component from u_cyl
```

$$u\_cyl := -\frac{7875}{52} s^3 \sin(\phi) \left(s^2 - z^2 - 1 \right) \left(\cos(\phi)^2 - \frac{1}{2} \right) \cos(\phi) \bar{e}_s - \frac{7875}{26} s \left(s^2 \left(s^2 \right.\right. \quad (36)$$

$$-\frac{1}{2}z^2-\frac{1}{2}\Big)\cos(\phi)^4-s^2\left(s^2-\frac{1}{2}z^2-\frac{1}{2}\right)\cos(\phi)^2+\frac{1}{4}s^4+\left(\frac{1}{2}z^2-\frac{3}{10}\right)s^2$$
$$+\frac{5}{16}z^4-\frac{21}{40}z^2+\frac{9}{80}\Big)\bar{e}_\phi-\frac{7875}{52}s^4 z\left(2\cos(\phi)^2-1\right)\cos(\phi)\sin(\phi)\bar{e}_z$$

```
> simplify(int(int( u_cyl[2],z=-sqrt(1-s^2)..sqrt(1-s^2)),phi=0..2*
  Pi)); #check that no geostrophic component remains
```

$$0 \tag{37}$$

```
> map(factor, simplify(MapToBasis(u_soln, spherical[r,theta,phi])))
  ;  # added
```

$$-\frac{7875}{104}\sin(\theta)^4\sin(\phi)\cos(\phi)(r-1)(r+1)\left(2\cos(\phi)^2-1\right)r^3\bar{e}_r \tag{38}$$
$$+\frac{7875}{104}\sin(\theta)^3\sin(\phi)\cos(\phi)\cos(\theta)\left(r^2+1\right)\left(2\cos(\phi)^2-1\right)r^3\bar{e}_\theta$$
$$-\frac{1}{32032}r\sin(\theta)\Big(1939875+14553000\cos(\phi)^4\cos(\theta)^4r^4$$
$$-24255000\cos(\phi)^4r^4\cos(\theta)^2-1261260\cos(\theta)^4r^4T_{5,0,0}$$
$$+4851000\cos(\phi)^4\cos(\theta)^2r^2+24255000r^4\cos(\phi)^2\cos(\theta)^2$$
$$+2522520\cos(\theta)^2r^4T_{5,0,0}+110250r^2\cos(\theta)^2+1819125r^4\cos(\theta)^4$$
$$+9702000r^4\cos(\phi)^4-4851000r^2\cos(\phi)^4-9702000\cos(\phi)^2r^4-2425500r^4\cos(\theta)^2$$
$$+4851000\cos(\phi)^2r^2-240240\cos(\theta)^2r^2T_{3,0,0}-1375920\cos(\theta)^2r^2T_{5,0,0}$$
$$+240240r^2T_{3,0,0}+1375920r^2T_{5,0,0}-1261260r^4T_{5,0,0}$$
$$-14553000\cos(\phi)^2r^4\cos(\theta)^4+3638250r^4-4851000\cos(\phi)^2r^2\cos(\theta)^2$$
$$-32032T_{1,0,0}-5203800r^2-305760T_{5,0,0}-137280T_{3,0,0}\Big)\bar{e}_\phi$$

> # Construct the ODE for geostrophic flow

```
> B_cyl := simplify(MapToBasis(B_cart, cylindrical[s,phi,z]));
```

$$B\_cyl := -\frac{3}{104}\sqrt{5}\sqrt{26}\,s\left(15s^2+25z^2-21\right)\left(2\cos(\phi)^2-1\right)\bar{e}_s \tag{39}$$
$$+\frac{75}{52}s\cos(\phi)\sqrt{26}\sin(\phi)\sqrt{5}\left(s^2+z^2-\frac{21}{25}\right)\bar{e}_\phi+\frac{15}{26}s^2\sqrt{26}\,z\Big(\cos(\phi)^2$$
$$-\frac{1}{2}\Big)\sqrt{5}\,\bar{e}_z$$

> $B\_sph := simplify(MapToBasis(B\_cyl, spherical[r, \theta, \phi]));$

$$B\_sph := -\frac{9}{104}\sin(\theta)^2\left(5r^2-7\right)\left(2\cos(\phi)^2-1\right)\sqrt{5}\sqrt{26}\,r\bar{e}_r \tag{40}$$
$$-\frac{3}{104}\cos(\theta)\sqrt{5}\sqrt{26}\,r\sin(\theta)\left(25r^2-21\right)\left(2\cos(\phi)^2-1\right)\bar{e}_\theta$$

$$+\frac{75}{52} r \sin(\theta) \cos(\phi) \sqrt{26} \left(r^2 - \frac{21}{25}\right) \sin(\phi) \sqrt{5}\, \bar{e}_{\phi}$$

```
> SetCoordinates(cylindrical[s, phi, z])
```

$$cylindrical_{s,\,\phi,\,z} \tag{41}$$

```
> int(int(CrossProduct(Curl(B_cyl), B_cyl)[2], z=-sqrt(1-s^2)..sqrt(1-s^2)), phi=0..2·Pi);#check that it is a Taylor state
```

$$0 \tag{42}$$

```
> u_geo := ug(s) · VectorField([0, 1, 0]);#introduce geostrophic component
```

$$u\_geo := (ug(s))\bar{e}_{\phi} \tag{43}$$

```
> eta := 1;
```

$$\eta := 1 \tag{44}$$

```
> Delta_t := 1/1000000000;
#Define timestep size - choose a small value to compare to instantaneous solutions
```

$$Delta\_t := \frac{1}{1000000000} \tag{45}$$

```
> #Discretise the induction equation and use it the evolve the magnetic field by a finite timestep
> B_nplusone_cyl := simplify(B_cyl + Delta_t·(Curl(CrossProduct(u_cyl, B_cyl))
    + Curl(CrossProduct(u_geo, B_cyl)) + eta · Laplacian(B_cyl)));
```

$$B\_nplusone\_cyl := -\frac{189}{2163200000}\Bigg(s^5 \sin(\phi)\left(s^2 + \frac{7}{5} - 9z^2\right)\cos(\phi)^5 - s^5 \sin(\phi)\Bigg(s^2 + \frac{7}{5} - 9z^2\Bigg)\cos(\phi)^3 + \frac{208000000}{21} s\left(s^2 + \frac{5}{3}z^2 - \frac{699999993}{500000000}\right)\cos(\phi)^2 - \sin(\phi)\Bigg(\Bigg(\left(-\frac{52}{2625}s^2 - \frac{52}{1575}z^2 + \frac{52}{1875}\right)ug(s) + s\Bigg(s^6 + \left(6z^2 - \frac{37}{10}\right)s^4 + \left(\frac{55}{8}z^4 - \frac{207}{20}z^2 + \frac{639}{200}\right)s^2 + \frac{25}{8}z^6 - \frac{63}{8}z^4 + \frac{1107}{200}z^2 - \frac{189}{200}\Bigg)\Bigg)\cos(\phi) - \frac{104000000}{21} s\left(s^2 + \frac{5}{3}z^2 - \frac{699999993}{500000000}\right)\Bigg)\Bigg)\sqrt{26}\sqrt{5}\,\bar{e}_s - \frac{567}{2163200000}\sqrt{26}\sqrt{5}\Bigg(\frac{26}{7875} s\left(s^2 + \frac{5}{3}z^2 - \frac{7}{5}\right)\left(\cos(\phi)^2 - \frac{1}{2}\right)\left(\frac{d}{ds}ug(s)\right) + s^5\left(s^2 + \frac{7}{15} - 3z^2\right)\cos(\phi)^6 - \frac{3}{2}s^5\left(s^2 + \frac{7}{15} - 3z^2\right)\cos(\phi)^4 + \Bigg(\Bigg(-\frac{26}{5625} + \frac{26}{3375}s^2 + \frac{26}{4725}z^2\Bigg)ug(s) - \frac{4}{3}s\Bigg(s^6 + \left(\frac{9}{2}z^2 - \frac{59}{20}\right)s^4 + \Bigg(\frac{85}{32}z^4 - \frac{351}{80}z^2 + \frac{1233}{800}\Bigg)s^2 + \frac{25}{32}z^6 - \frac{63}{32}z^4 + \frac{1107}{800}z^2 - \frac{189}{800}\Bigg)\Bigg)\cos(\phi)^2 - \frac{1040000000}{189} s\Bigg(s^2 \tag{46}$$

$$+z^2 - \frac{2099999979}{2500000000}\Big) \sin(\phi)\cos(\phi) + \left(-\frac{13}{3375} s^2 - \frac{13}{4725} z^2 + \frac{13}{5625}\right) ug(s)$$
$$+ \frac{11}{12} s \left(s^6 + \left(\frac{27}{11} z^2 - \frac{111}{55}\right) s^4 + \left(\frac{85}{44} z^4 - \frac{351}{110} z^2 + \frac{1233}{1100}\right) s^2 + \frac{25}{44} z^6 - \frac{63}{44} z^4\right.$$
$$\left. + \frac{1107}{1100} z^2 - \frac{189}{1100}\right)\Big)\bar{e}_\phi - \frac{189}{216320000} s\sqrt{26}\,\Big(s^5\cos(\phi)^5\sin(\phi) - \cos(\phi)^3\sin(\phi)\, s^5$$
$$- \frac{41600000}{63} s\cos(\phi)^2 + \frac{7}{50}\sin(\phi)\left(-\frac{104}{11025} ug(s) + s^3 + \left(\frac{25}{28} z^4 - \frac{3}{2} z^2\right.\right.$$
$$\left.\left. + \frac{9}{28}\right) s\right)\cos(\phi) + \frac{20800000}{63} s\Big)\sqrt{5}\, z\bar{e}_z$$

```
> SetCoordinates(spherical[r, theta, phi])
```

$$spherical_{r,\,\theta,\,\phi} \tag{47}$$

```
> #numerically expand B_nplusone
```

```
> N := 5; # order of chebysev expansion
```

$$N := 5 \tag{48}$$

```
> M := N + 1 : # order of expansion including logarithmic term
> with(orthopoly) :
```

```
> chebsum := add(a||n·s·T(n, 2 s^2 − 1), n = 1 ..N) :
> expansion := eval(chebsum) + b·s·ln(s);
```

$$expansion := a1\, s\,(2 s^2 - 1) + a2\, s\,(-1 + 2 (2 s^2 - 1)^2) + a3\, s\,(4 (2 s^2 - 1)^3 - 6 s^2 + 3)$$
$$+ a4\, s\,(1 + 8 (2 s^2 - 1)^4 - 8 (2 s^2 - 1)^2) + a5\, s\,(16 (2 s^2 - 1)^5 - 20 (2 s^2 - 1)^3$$
$$+ 10 s^2 - 5) + b\, s\ln(s) \tag{49}$$

```
> B_cyl_approx := simplify(subs(ug(s) = expansion, B_nplusone_cyl));
```

$$B\_cyl\_approx := -\frac{9}{5200000000}\sqrt{5}\, s\sqrt{26}\,\Big(\frac{2625}{52} s^4\left(s^2 + \frac{7}{5} - 9 z^2\right)\sin(\phi)\cos(\phi)^5 \tag{50}$$
$$- \frac{2625}{52} s^4\left(s^2 + \frac{7}{5} - 9 z^2\right)\sin(\phi)\cos(\phi)^3 + \Big(500000000 s^2 + \frac{2500000000}{3} z^2$$
$$- 699999993\Big)\cos(\phi)^2 + \Big(b\left(s^2 + \frac{5}{3} z^2 - \frac{7}{5}\right)\ln(s) + 512 s^{12} a5 + \Big(128\, a4$$

$$- \frac{9984}{5}\, a5 + \frac{2560}{3}\, z^2\, a5 \Big) s^{10} + \Big(\Big(\frac{640}{3}\, a4 - \frac{6400}{3}\, a5 \Big) z^2 + 32\, a3 - \frac{2176}{5}\, a4$$

$$+ 2912\, a5 \Big) s^8 + \Big(\Big(\frac{160}{3}\, a3 - \frac{1280}{3}\, a4 + \frac{5600}{3}\, a5 \Big) z^2 + 8\, a2 - \frac{464}{5}\, a3 + \frac{2592}{5}\, a4$$

$$- 1968\, a5 - \frac{2625}{52} \Big) s^6 + \Big(\Big(-\frac{7875}{26} + \frac{40}{3}\, a2 - 80\, a3 + \frac{800}{3}\, a4 - \frac{2000}{3}\, a5 \Big) z^2$$

$$+ 2\, a1 - \frac{96}{5}\, a2 - 256\, a4 + 610\, a5 + \frac{426}{5}\, a3 + \frac{19425}{104} \Big) s^4 + \Big(-\frac{144375}{416}\, z^4$$

$$+ \Big(\frac{108675}{208} + \frac{10}{3}\, a1 - \frac{40}{3}\, a2 + 30\, a3 - \frac{160}{3}\, a4 + \frac{250}{3}\, a5 \Big) z^2 + \frac{229}{5}\, a4 - 71\, a5$$

$$+ \frac{61}{5}\, a2 - \frac{131}{5}\, a3 - \frac{67095}{416} - \frac{19}{5}\, a1 \Big) s^2 - \frac{65625}{416} \Big(z^4 - \frac{42}{25}\, z^2 + \frac{416}{39375}\, a1$$

$$- \frac{416}{39375}\, a2 + \frac{416}{39375}\, a3 - \frac{416}{39375}\, a4 + \frac{416}{39375}\, a5 + \frac{9}{25} \Big) \Big(z^2 - \frac{21}{25} \Big) \Big)$$

$$\sin(\phi) \cos(\phi) + \frac{699999993}{2} - 250000000\, s^2 - \frac{1250000000}{3}\, z^2 \Big) \bar{e}_s$$

$$- \frac{3}{1040000000} \sqrt{5}\, s \sqrt{26} \Big(\frac{4725}{52}\, s^4 \Big(s^2 + \frac{7}{15} - 3\, z^2 \Big) \cos(\phi)^6 - \frac{14175}{104}\, s^4 \Big(s^2$$

$$+ \frac{7}{15} - 3\, z^2 \Big) \cos(\phi)^4 + \Big(b \Big(s^2 + z^2 - \frac{21}{25} \Big) \ln(s) + 2048\, s^{12}\, a5 + \Big(\frac{2176}{5}\, a4$$

$$- \frac{173312}{25}\, a5 + 3072\, z^2\, a5 \Big) s^{10} + \Big((640\, a4 - 6400\, a5)\, z^2 + \frac{448}{5}\, a3 - \frac{6272}{5}\, a4$$

$$+ 8512\, a5 \Big) s^8 + \Big((128\, a3 - 1024\, a4 + 4480\, a5)\, z^2 + \frac{88}{5}\, a2 - \frac{5328}{25}\, a3 + \frac{30304}{25}\, a4$$

$$- \frac{23216}{5}\, a5 - \frac{1575}{13} \Big) s^6 + \Big(\Big(-\frac{14175}{26} + 24\, a2 - 144\, a3 + 480\, a4 - 1200\, a5 \Big) z^2$$

$$+ \frac{16}{5}\, a1 - \frac{824}{25}\, a2 - \frac{2272}{5}\, a4 + 1088\, a5 + \frac{3744}{25}\, a3 + \frac{18585}{52} \Big) s^4 + \Big(-\frac{133875}{416}\, z^4$$

$$+ \Big(\frac{8505}{16} + 4\, a1 - 16\, a2 + 36\, a3 - 64\, a4 + 100\, a5 \Big) z^2 + \frac{1369}{25}\, a4 - 85\, a5 + \frac{361}{25}\, a2$$

$$- \frac{781}{25}\, a3 - \frac{77679}{416} - \frac{109}{25}\, a1 + \frac{3}{10}\, b \Big) s^2 + \frac{1}{2} \Big(z^2 - \frac{21}{25} \Big) \Big(-\frac{39375}{208}\, z^4$$

$$+ \frac{33075}{104}\, z^2 + b - 2\, a1 + 2\, a2 - 2\, a3 + 2\, a4 - 2\, a5 - \frac{14175}{208} \Big) \Big) \cos(\phi)^2$$

$$- 500000000 \sin(\phi) \Big(s^2 + z^2 - \frac{2099999979}{2500000000} \Big) \cos(\phi) - \frac{1}{2}\, b \Big(s^2 + z^2 - \frac{21}{25} \Big) \ln(s)$$

$$- 1024\, s^{12}\, a5 + \Big(-\frac{1088}{5}\, a4 + \frac{86656}{25}\, a5 - 1536\, z^2\, a5 \Big) s^{10} + \Big((-320\, a4$$

$$+ 3200\, a5)\, z^2 - \frac{224}{5}\, a3 + \frac{3136}{5}\, a4 - 4256\, a5 \Big) s^8 + \Big((-64\, a3 + 512\, a4 - 2240\, a5)\, z^2$$

$$- \frac{44}{5}\, a2 + \frac{2664}{25}\, a3 - \frac{15152}{25}\, a4 + \frac{11608}{5}\, a5 + \frac{17325}{208} \Big) s^6 + \Big(\Big(\frac{42525}{208} - 12\, a2$$

$$+ 72\, a3 - 240\, a4 + 600\, a5 \Big) z^2 - \frac{8}{5}\, a1 + \frac{412}{25}\, a2 + \frac{1136}{5}\, a4 - 544\, a5 - \frac{1872}{25}\, a3$$

$$- \frac{34965}{208} \Big) s^4 + \Big(\frac{133875}{832} z^4 + \Big(- \frac{8505}{32} - 2\, a1 + 8\, a2 - 18\, a3 + 32\, a4 - 50\, a5 \Big) z^2$$
$$- \frac{1369}{50} a4 + \frac{85}{2} a5 - \frac{361}{50} a2 + \frac{781}{50} a3 + \frac{77679}{832} + \frac{109}{50} a1 - \frac{3}{20} b \Big) s^2 - \frac{1}{4} \Big(z^2$$
$$- \frac{21}{25} \Big) \Big(- \frac{39375}{208} z^4 + \frac{33075}{104} z^2 + b - 2\, a1 + 2\, a2 - 2\, a3 + 2\, a4 - 2\, a5$$
$$- \frac{14175}{208} \Big) \Big) \bar{e}_{\phi} + \frac{3}{2600000000} \sqrt{5}\, s^2 \sqrt{26} \Big(-250000000 - \frac{39375}{52} \cos(\phi)^5 \sin(\phi)\, s^4$$
$$+ \frac{39375}{52} \cos(\phi)^3 \sin(\phi)\, s^4 + 500000000 \cos(\phi)^2 + \Big(b \ln(s) + 512\, a5\, s^{10} + (128\, a4$$
$$- 1280\, a5)\, s^8 + (32\, a3 - 256\, a4 + 1120\, a5)\, s^6 + (8\, a2 + 160\, a4 - 400\, a5 - 48\, a3)\, s^4$$
$$+ \Big(-32\, a4 + 50\, a5 - 8\, a2 + 18\, a3 - \frac{11025}{104} + 2\, a1 \Big) s^2 - \frac{14175}{416} + \frac{33075}{208} z^2 - a5$$
$$+ a4 - a1 + a2 - a3 - \frac{39375}{416} z^4 \Big) \sin(\phi) \cos(\phi) \Big) z\bar{e}_{z}$$

> *B_sph_approx* := *simplify*(*MapToBasis*(*B_cyl_approx*, *spherical*[r, theta, phi]));

$$B\_sph\_approx := - \frac{9}{10400000000} \Big(\cos(\phi) \sin(\phi)\, b \Big(- \frac{7}{5} + r^2 \Big) \ln\big(r^2 \sin(\theta)^2 \big) \tag{51}$$
$$+ \frac{2625}{26} \Big(r^2 + \frac{7}{5} \Big) \sin(\phi)\, r^4 (\cos(\theta) - 1)^2 (\cos(\theta) + 1)^2 \cos(\phi)^5 - \frac{2625}{26} \Big(r^2$$
$$+ \frac{7}{5} \Big) \sin(\phi)\, r^4 (\cos(\theta) - 1)^2 (\cos(\theta) + 1)^2 \cos(\phi)^3 + (1000000000\, r^2$$
$$- 1399999986) \cos(\phi)^2 - 1024 \sin(\phi) \Big(r^{10}\, a5 \Big(- \frac{7}{5} + r^2 \Big) \cos(\theta)^{10} - 5\, r^8 \Big(r^2\, a5$$
$$+ \frac{1}{20} a4 - \frac{1}{2} a5 \Big) \Big(- \frac{7}{5} + r^2 \Big) \cos(\theta)^8 + 10 \Big(a5\, r^4 + \Big(\frac{1}{10} a4 - a5 \Big) r^2 + \frac{1}{160} a3$$
$$- \frac{1}{20} a4 + \frac{7}{32} a5 \Big) r^6 \Big(- \frac{7}{5} + r^2 \Big) \cos(\theta)^6 - 10\, r^4 \Big(a5\, r^8 + \Big(\frac{3}{20} a4 - \frac{29}{10} a5 \Big) r^6$$
$$+ \Big(\frac{3}{160} a3 - \frac{9}{25} a4 + \frac{441}{160} a5 \Big) r^4 + \Big(\frac{1}{640} a2 - \frac{57}{1600} a3 + \frac{193}{800} a4 - \frac{319}{320} a5$$
$$+ \frac{8925}{425984} \Big) r^2 - \frac{7}{3200} a2 + \frac{21}{1600} a3 - \frac{7}{160} a4 + \frac{7}{64} a5 - \frac{735}{32768} \Big) \cos(\theta)^4$$
$$+ 5\, r^2 \Big(a5\, r^{10} + \Big(\frac{1}{5} a4 - \frac{17}{5} a5 \Big) r^8 + \Big(\frac{3}{80} a3 - \frac{29}{50} a4 + \frac{329}{80} a5 \Big) r^6 + \Big(\frac{1}{160} a2$$
$$- \frac{9}{100} a3 + \frac{109}{200} a4 - \frac{43}{20} a5 + \frac{1575}{26624} \Big) r^4 + \Big(\frac{1}{1280} a1 - \frac{19}{1600} a2 + \frac{381}{6400} a3$$
$$- \frac{3}{16} a4 + \frac{117}{256} a5 - \frac{9135}{106496} \Big) r^2 - \frac{7}{6400} a1 + \frac{7}{1600} a2 - \frac{63}{6400} a3 + \frac{7}{400} a4$$
$$- \frac{7}{256} a5 + \frac{3969}{106496} \Big) \cos(\theta)^2 - a5\, r^{12} + \Big(\frac{39}{10} a5 - \frac{1}{4} a4 \Big) r^{10} + \Big(\frac{17}{20} a4 - \frac{91}{16} a5$$
$$- \frac{1}{16} a3 \Big) r^8 + \Big(\frac{29}{160} a3 - \frac{81}{80} a4 + \frac{123}{32} a5 - \frac{1}{64} a2 + \frac{2625}{26624} \Big) r^6 + \Big(\frac{3}{80} a2$$

$$- \frac{213}{1280}\, a3 - \frac{305}{256}\, a5 + \frac{1}{2}\, a4 - \frac{19425}{53248} - \frac{1}{256}\, a1 \Big) r^4 + \Big(\frac{71}{512}\, a5 + \frac{131}{2560}\, a3$$

$$- \frac{229}{2560}\, a4 + \frac{67095}{212992} + \frac{19}{2560}\, a1 - \frac{61}{2560}\, a2 \Big) r^2 - \frac{19845}{212992} - \frac{7}{2560}\, a1$$

$$- \frac{7}{2560}\, a5 + \frac{7}{2560}\, a2 - \frac{7}{2560}\, a3 + \frac{7}{2560}\, a4 \Big) \cos(\phi) - 500000000\, r^2$$

$$+ 699999993 \Big) \sqrt{26}\, r \sqrt{5}\, \sin(\theta)^2 \overline{e}_r$$

$$- \frac{3}{2080000000} \sqrt{26}\, r \cos(\theta) \Big(\cos(\phi) \sin(\phi)\, b \Big(r^2 - \frac{21}{25} \Big) \ln\big(r^2 \sin(\theta)^2 \big)$$

$$- \frac{14175}{26} \sin(\phi)\, r^4 (\cos(\theta) - 1)^2 \Big(r^2 - \frac{7}{45} \Big) (\cos(\theta) + 1)^2 \cos(\phi)^5$$

$$+ \frac{14175}{26} \sin(\phi)\, r^4 (\cos(\theta) - 1)^2 \Big(r^2 - \frac{7}{45} \Big) (\cos(\theta) + 1)^2 \cos(\phi)^3$$

$$+ \Big(1000000000\, r^2 - \frac{4199999958}{5} \Big) \cos(\phi)^2 - 1024 \sin(\phi) \Big(r^{10}\, a5 \Big(r^2$$

$$- \frac{21}{25} \Big) \cos(\theta)^{10} - 5\, r^8 \Big(r^2\, a5 + \frac{1}{20}\, a4 - \frac{1}{2}\, a5 \Big) \Big(r^2 - \frac{21}{25} \Big) \cos(\theta)^8 + 10 \Big(a5\, r^4$$

$$+ \Big(\frac{1}{10}\, a4 - a5 \Big) r^2 + \frac{1}{160}\, a3 - \frac{1}{20}\, a4 + \frac{7}{32}\, a5 \Big) r^6 \Big(r^2 - \frac{21}{25} \Big) \cos(\theta)^6 - 10 \Big(a5\, r^8$$

$$+ \Big(\frac{3}{20}\, a4 - \frac{117}{50}\, a5 \Big) r^6 + \Big(\frac{3}{160}\, a3 - \frac{69}{250}\, a4 + \frac{1533}{800}\, a5 \Big) r^4 + \Big(\frac{1}{640}\, a2$$

$$- \frac{201}{8000}\, a3 + \frac{629}{4000}\, a4 - \frac{1007}{1600}\, a5 + \frac{2205}{425984} \Big) r^2 - \frac{21}{16000}\, a2 + \frac{63}{8000}\, a3$$

$$- \frac{21}{800}\, a4 + \frac{21}{320}\, a5 - \frac{441}{32768} \Big) r^4 \cos(\theta)^4 + 5\, r^2 \Big(a5\, r^{10} + \Big(\frac{1}{5}\, a4 - \frac{71}{25}\, a5 \Big) r^8$$

$$+ \Big(\frac{3}{80}\, a3 - \frac{117}{250}\, a4 + \frac{1197}{400}\, a5 \Big) r^6 + \Big(\frac{1}{160}\, a2 - \frac{69}{1000}\, a3 + \frac{377}{1000}\, a4 - \frac{283}{200}\, a5$$

$$+ \frac{945}{26624} \Big) r^4 + \Big(\frac{1}{1280}\, a1 - \frac{67}{8000}\, a2 + \frac{1233}{32000}\, a3 - \frac{47}{400}\, a4 + \frac{361}{1280}\, a5$$

$$- \frac{9891}{106496} \Big) r^2 - \frac{21}{32000}\, a1 + \frac{21}{8000}\, a2 - \frac{189}{32000}\, a3 + \frac{21}{2000}\, a4 - \frac{21}{1280}\, a5$$

$$+ \frac{11907}{532480} \Big) \cos(\theta)^2 - a5\, r^{12} + \Big(\frac{167}{50}\, a5 - \frac{1}{4}\, a4 \Big) r^{10} + \Big(\frac{71}{100}\, a4 - \frac{343}{80}\, a5$$

$$- \frac{1}{16}\, a3 \Big) r^8 + \Big(\frac{117}{800}\, a3 - \frac{293}{400}\, a4 + \frac{419}{160}\, a5 - \frac{1}{64}\, a2 + \frac{1575}{26624} \Big) r^6 + \Big(\frac{23}{800}\, a2$$

$$- \frac{729}{6400}\, a3 - \frac{193}{256}\, a5 + \frac{13}{40}\, a4 - \frac{7245}{53248} - \frac{1}{256}\, a1 \Big) r^4 + \Big(\frac{43}{512}\, a5 + \frac{403}{12800}\, a3$$

$$- \frac{697}{12800}\, a4 + \frac{45927}{212992} + \frac{67}{12800}\, a1 - \frac{193}{12800}\, a2 \Big) r^2 - \frac{11907}{212992} - \frac{21}{12800}\, a1$$

$$- \frac{21}{12800}\, a5 + \frac{21}{12800}\, a2 - \frac{21}{12800}\, a3 + \frac{21}{12800}\, a4 \Big) \cos(\phi) + \frac{2099999979}{5}$$

$$- 500000000\, r^2 \Big) \sqrt{5}\, \sin(\theta) \overline{e}_\theta - \frac{3}{5200000000} \sqrt{26} \Big(\frac{5}{2}\, b \Big(\cos(\phi)^2 - \frac{1}{2} \Big) \Big(r^2$$

$$-\frac{21}{25}\Big)\ln\big(r^2\sin(\theta)^2\big)-5120\,r^{12}\left(\cos(\phi)^2-\frac{1}{2}\right)a5\cos(\theta)^{12}+15360\,\Big(r^2\,a5$$

$$+\frac{1}{15}\,a4+\frac{13}{75}\,a5\Big)\,r^{10}\left(\cos(\phi)^2-\frac{1}{2}\right)\cos(\theta)^{10}-1920\,r^8\left(\cos(\phi)^2-\frac{1}{2}\right)\Big(\Big(a4$$

$$+\frac{118}{5}\,a5\Big)\,r^2+\frac{1}{10}\,a3+\frac{3}{5}\,a4-\frac{21}{2}\,a5\Big)\cos(\theta)^8-\frac{23625}{13}\,r^6\Big(\cos(\phi)^2$$

$$-\frac{1}{2}\Big)\Big(\cos(\phi)^4-\cos(\phi)^2+\frac{26624}{945}\,a5\,r^6+\left(\frac{6656}{4725}\,a4-\frac{2010112}{23625}\,a5\right)r^4+\Big($$

$$-\frac{1664}{23625}\,a3-\frac{126464}{23625}\,a4+\frac{38272}{675}\,a5\Big)\,r^2-\frac{416}{23625}\,a2-\frac{832}{4375}\,a3+\frac{237952}{118125}\,a4$$

$$-\frac{223808}{23625}\,a5+\frac{1}{24}\Big)\cos(\theta)^6+\frac{212625}{52}\,r^4\left(\cos(\phi)^2-\frac{1}{2}\right)\Big(\left(r^2+\frac{7}{135}\right)\cos(\phi)^4$$

$$+\left(-r^2-\frac{7}{135}\right)\cos(\phi)^2+\frac{53248}{2835}\,a5\,r^8+\left(\frac{13312}{6075}\,a4-\frac{1624064}{30375}\,a5\right)r^6$$

$$+\left(\frac{13312}{70875}\,a3-\frac{386048}{70875}\,a4+\frac{93184}{2025}\,a5\right)r^4+\Big(\frac{416}{70875}\,a2-\frac{50752}{118125}\,a3$$

$$+\frac{1159808}{354375}\,a4-\frac{999232}{70875}\,a5-\frac{1}{216}\Big)\,r^2-\frac{208}{212625}\,a1-\frac{22048}{1063125}\,a2+\frac{16432}{118125}\,a3$$

$$-\frac{101504}{212625}\,a4+\frac{51376}{42525}\,a5+\frac{19}{360}\Big)\cos(\theta)^4+r^2\Big(\Big(-\frac{70875}{26}\,r^4$$

$$-\frac{11025}{26}\,r^2\Big)\cos(\phi)^4+\left(\frac{70875}{26}\,r^4+\frac{11025}{26}\,r^2\right)\cos(\phi)^2-46080\,a5\,r^{10}+(-7680\,a4$$

$$+141312\,a5)\,r^8+(-1152\,a3+19968\,a4-147840\,a5)\,r^6+\Big(-144\,a2+\frac{12384}{5}\,a3$$

$$+63648\,a5-\frac{78912}{5}\,a4+\frac{23625}{52}\Big)\,r^4+\Big(-10380\,a5-\frac{6588}{5}\,a3+4224\,a4$$

$$-\frac{146475}{208}-12\,a1+\frac{1248}{5}\,a2\Big)\,r^2+\frac{84}{5}\,a1+420\,a5+b-\frac{336}{5}\,a2+\frac{756}{5}\,a3$$

$$-\frac{1344}{5}\,a4+\frac{19845}{208}\Big)\left(\cos(\phi)^2-\frac{1}{2}\right)\cos(\theta)^2+\left(\frac{11025}{52}\,r^4+\frac{23625}{52}\,r^6\right)\cos(\phi)^6$$

$$-\frac{70875}{104}\,r^4\left(r^2+\frac{7}{15}\right)\cos(\phi)^4+\Big(10240\,a5\,r^{12}+\left(-\frac{173312}{5}\,a5+2176\,a4\right)r^{10}+($$

$$-6272\,a4+42560\,a5+448\,a3)\,r^8+\Big(-\frac{5328}{5}\,a3+\frac{30304}{5}\,a4-23216\,a5+88\,a2$$

$$-\frac{7875}{13}\Big)\,r^6+\left(-\frac{824}{5}\,a2+\frac{3744}{5}\,a3+5440\,a5-2272\,a4+\frac{92925}{52}+16\,a1\right)r^4$$

$$+\left(-425\,a5-\frac{781}{5}\,a3+\frac{1369}{5}\,a4-\frac{388395}{416}-\frac{109}{5}\,a1+\frac{361}{5}\,a2+\frac{3}{2}\,b\right)r^2$$

$$+\frac{21}{5}\,a1+\frac{59535}{416}+\frac{21}{5}\,a5-\frac{21}{10}\,b-\frac{21}{5}\,a2+\frac{21}{5}\,a3-\frac{21}{5}\,a4\Big)\cos(\phi)^2+($$

$$-2500000000\,r^2+2099999979)\sin(\phi)\cos(\phi)-5120\,a5\,r^{12}+\Big(\frac{86656}{5}\,a5$$

$$-1088\,a4\Big)\,r^{10}+(3136\,a4-21280\,a5-224\,a3)\,r^8+\Big(\frac{2664}{5}\,a3-\frac{15152}{5}\,a4$$

$$+ 11608\, a5 - 44\, a2 + \frac{86625}{208}\Big) r^6 + \Big(\frac{412}{5}\, a2 - \frac{1872}{5}\, a3 - 2720\, a5 + 1136\, a4 - \frac{174825}{208} - 8\, a1\Big) r^4 + \Big(\frac{425}{2}\, a5 + \frac{781}{10}\, a3 - \frac{1369}{10}\, a4 + \frac{388395}{832} + \frac{109}{10}\, a1 - \frac{361}{10}\, a2 - \frac{3}{4}\, b\Big) r^2 - \frac{21}{10}\, a1 - \frac{59535}{832} - \frac{21}{10}\, a5 + \frac{21}{20}\, b + \frac{21}{10}\, a2 - \frac{21}{10}\, a3 + \frac{21}{10}\, a4\Big) r \sqrt{5} \sin(\theta) \bar{e}_{\phi}$$

> #find toroidal and poloidal components of B_nplusone

```
> toroidal_part := simplify(expand(Curl(B_sph_approx)[1]*r^2)) :
```

```
> poloidal_part := simplify(B_sph_approx[1]*r^2) :
```

> #Project B_nplusone onto boundary conditions

```
> #Define maximum values of m,l,n which are considered
> Mmax := 4;
```

$$Mmax := 4 \quad (52)$$

```
> Lmax := 10;
```

$$Lmax := 10 \quad (53)$$

```
> Nmax := 10;
```

$$Nmax := 10 \quad (54)$$

```
> #initialise poloidal array
> pol_proj := Array(0 .. Lmax, 0 .. Nmax, -Mmax .. Mmax );
```

pol_proj := [0..10 x 0..10 x -4..4 Array
Data Type: anything
Storage: rectangular
Order: Fortran_order] **(55)**

```
> #Project poloidal component
```

```
> for m from -Mmax to Mmax do:
   for i from max(1, abs(m)) to Lmax do:
   poloidal_part_l := (int(int( poloidal_part * L2(i, m)·sin(theta), theta = 0 ..Pi), phi = 0 ..2·Pi))
        / (4 * Pi / (2 * i + 1)) / (i * (i + 1)) :
   for n from 1 to Nmax do
   pol_proj[i, n, m] := int( i * (i + 1) / r^2 * poloidal_part_l * Psi_n(i, n)
        + diff(poloidal_part_l, r) * diff(Psi_n(i, n), r) , r = 0 ..1) / int( i * (i + 1) / r^2 * Psi_n(i,
        n)^2 + diff(Psi_n(i, n), r)^2, r = 0 ..1) :
  od:
   od:
   od:
```

```
> #initialise toroidal array
> tor_proj := Array(0 .. Lmax, 0 .. Nmax, -Mmax .. Mmax );
```

$$tor\_proj := \begin{bmatrix} \text{0..10 x 0..10 x -4..4 Array} \\ \text{Data Type: anything} \\ \text{Storage: rectangular} \\ \text{Order: Fortran\_order} \end{bmatrix} \quad \textbf{(56)}$$

```
> #Project toroidal component

> for m from -Mmax to Mmax do:
   for i from max(1, abs(m)) to Lmax do:
   tor_part_l := (int(int( expand(toroidal_part* L2(i, m)·sin(theta)), theta = 0 ..Pi), phi = 0 ..2
       ·Pi)) / (4 * Pi / (2 * i + 1)) / (i * (i + 1)) :
  for n from 1 to Nmax do
   tor_proj[i, n, m] := int( expand(tor_part_l * Chi_n(i, n)) , r = 0 ..1) / int( expand(Chi_n(i, n)
       ^2) , r = 0 ..1) :
  od: print (i);
   od:
   od:
> #Recomplie magnetic field

> B_next2 := simplify(expand(evalm(Curl(Curl( add(add(add(pol_proj[i, n, m] * Psi_n(i, n)
       * L2(i, m), n = 1 ..Nmax), i = max(1, abs(m)) ..Lmax), m =-Mmax ..Mmax)
       · VectorField([1, 0, 0]))) + Curl(add(add(add(tor_proj[i, n, m] * Chi_n(i, n) * L2(i,
       m), n = 1 ..Nmax), i = max(1, abs(m)) ..Lmax), m =-Mmax ..Mmax) · VectorField([1, 0,
       0]))))) :

> B_proj_sph[1] := op(B_next2)[1] :
> B_proj_sph[2] := op(B_next2)[2] :
> B_proj_sph[3] := op(B_next2)[3] :
> #Projected B_nplusone
> B_proj_sph := B_proj_sph[1]·VectorField([1, 0, 0]) + B_proj_sph[2]·VectorField([0, 1, 0])
       + B_proj_sph[3]·VectorField([0, 0, 1]) :
> B_proj_cyl := simplify(MapToBasis(B_proj_sph, cylindrical[s, phi, z]));
```

$$B\_proj\_cyl := -\frac{9}{10400000000}\sqrt{2}\left(\left(b\left(s^2+\frac{5}{3}z^2-\frac{7}{5}\right)\ln(2)+\frac{42082751353}{1138229248}\right.\right.$$
$$+\frac{9251651977}{1244938240}a5+\frac{6285901997}{1834060800}a4+\left(\left(\frac{2605406615988883157}{30446848}a5\right.\right.$$
$$\left.+\frac{51271458371}{256}a3+\frac{1069963247761301}{413680}a4+\frac{1442883208475711392855 4357}{201806190320025600}b\right)$$
$$s^{16}+\left(-\frac{5008856446076461452795}{231335151104}a5+\frac{234052080735}{1024}a2\right.$$
$$+\frac{2581658995194155}{1945088}a3-\frac{2083222484170769 3281}{5029025024}a4$$
$$\left.-\frac{24854337584188839287275 9591}{106480794031357952 0}b+\frac{1843160135788125}{6815744}\right)s^{14} \quad \textbf{(57)}$$

$$+\left(\frac{9746194916247393528228150511}{5065046119797903360}\,a5-\frac{755063102082452813965}{523612717056}\right.$$

$$+\frac{12317261383063}{675840}\,a1-\frac{583701836339624303}{737510400}\,a2-\frac{77703287788665213401867}{11207208038400}\,a3$$

$$-\frac{201974597967829731082019779}{22939520470099200}\,a4$$

$$\left.+\frac{224783184663627996429375869564943119}{1049120976775291803795456000}\,b\right)s^{12}$$

$$+\left(\frac{58208067940216765966697713702403}{1337509845367966347264}\,a5+\frac{27787100019837527229175}{10166813589504}\right.$$

$$-\frac{5290607343889049}{52490240}\,a1+\frac{9536159288053236643}{11455994880}\,a2$$

$$+\frac{1922994767263159148460097}{174085298196480}\,a3+\frac{9685690712975183722599738311}{356327217968874240}\,a4$$

$$\left.+\frac{12967117062420893088362157297605877323}{138518939633564361161126707200}\,b\right)s^{10}+\Bigg($$

$$-\frac{20983488989103415378398232655553383}{390887252308788164987904}\,a5-\frac{7816622536343684978394275}{3395715738894336}$$

$$+\frac{622806906963247045}{3068054528}\,a1+\frac{62854117422317629861}{1674007251840}\,a2$$

$$-\frac{384511350581992411568571877}{50876428397921280}\,a3-\frac{22819581332790856007704239317849}{885161350336929721440}\,a4$$

$$\left.-\frac{7931498697067033139858422176321934463189}{25908582469061878111577139314688}\,b\right)s^{8}$$

$$+\left(\frac{19922340469965922878744622531073459}{781774504617576329975808}\,a5+\frac{1228642022814730436047475}{1697857869447168}\right.$$

$$-\frac{1219660328261042265}{6136109056}\,a1-\frac{9042696943595627064229}{13392058014720}\,a2$$

$$+\frac{2689920688693680197050654169}{1729798565529323520}\,a3+\frac{70261438817342731028807745754883}{7081290802695437771520}\,a4$$

$$\left.+\frac{3221369998672925373488168560712968508657}{14720785493785158017941556428800}\,b\right)s^{6}+\Bigg($$

$$-\frac{21274420973932600425206901500875}{5990609230786025517056}\,a5+\frac{268681789616173269757125}{2263810492596224}$$

$$+\frac{1245980213004738705}{12272218112}\,a1+\frac{149161941034348406935}{297601289216}\,a2$$

$$+\frac{1885405255258767913031979}{2651032284336128}\,a3-\frac{6699730140903092997817689035}{16278829431483764992}\,a4$$

$$\left.-\frac{1160100441936833755708311002618611}{18194024834736321861255168}\,b\right)s^{4}+\Bigg($$

$$-\frac{14937524867676592171976141659271}{17971827692358076551168}\,a5-\frac{4319187768795896960075}{35372038946816}$$

$$-\frac{45279045175935519}{1753174016}\,a1-\frac{95622655008295406867}{637717048320}\,a2$$

$$- \frac{2308266222774891208237573 9}{5680783466434560} a3 - \frac{557374924785069616129177039 81}{81394147157418824960} a4$$

$$+ \frac{363074985444556386184223199458087}{852844914128265087246336 00} b \Big) s^2 + \frac{420048767946995969312 5}{212232233680896}$$

$$+ \frac{288677000022657788161962780824983}{33260951651002338402607104 0} b + \frac{2243546085610771}{876587008} a1$$

$$+ \frac{218536102318564873 79}{1339205801472} a2 + \frac{600816726626600006280 13}{108451320722841 6} a3$$

$$+ \frac{574779520596079263465294972 1}{453481677019904881 92} a4 + \frac{766652480796519366707900521218 85}{350450640000982492747776} a5 \Big)$$

$$z^{12} + \Big(\Big(\frac{2304520236398976907}{365362176} a5 + \frac{4358073961535}{9216} a3 + \frac{6989084383480251 1}{29784960} a4$$

$$+ \frac{62734643365470955907714491}{908127856440115200} b \Big) s^{18} + \Big(- \frac{436159178303528812041}{15954148352} a5$$

$$+ \frac{276607004505}{2048} a2 - \frac{887865101232451}{670720} a3 - \frac{161471999982893813 59}{1734146560} a4$$

$$- \frac{300203960128417362480334157}{881220364397445120} b + \frac{167560012344375}{1048576} \Big) s^{16}$$

$$+ \Big(\frac{17504675095254700285245068226 5}{405203689583832268 8} a5 + \frac{1759608769009}{540672} a1$$

$$- \frac{748718678865950 45}{118001664} a2 - \frac{647639535429134075 45}{1793153286144} a3$$

$$+ \frac{442584425063686768872738 71}{3670323275215872} a4 + \frac{331238937745125460079555136893174 3}{476873171261496274452480 0} b$$

$$- \frac{283754717603504483525}{261806358528} \Big) s^{14} + \Big(- \frac{202384902231445849550285610272257}{802505907220779808358 4} a5$$

$$+ \frac{169160717239193647422175}{610008815370 24} - \frac{262353036175657 7}{62988288} a1$$

$$+ \frac{935835090928766296 1}{8591996160} a2 + \frac{411022733832754484821443 7}{1044511789178880} a3$$

$$- \frac{102786876236814238715771599 9}{534490826953311360} a4$$

$$- \frac{281669770586744943132932249998533593 71}{3777789262733573486212546560 0} b \Big) s^{12} + \Big($$

$$- \frac{2025098375457816157495867350395410 91}{312709801847030531990323 20} a5 - \frac{60323889792283380285920 21}{1697857869447168}$$

$$+ \frac{1554460039088775478 01}{1227221811200} a1 - \frac{200274875004069545787241}{267841160294400} a2$$

$$- \frac{223673190660769520879520559 69}{40701142718337024 00} a3$$

$$- \frac{131686793196162173248339419932427 7}{141625816053908755430400} a4$$

$$+ \frac{641870840232224946166006933564672375595859}{147207854937851580179415564288000} b \Big) s^{10}$$

$$+\left(\frac{24619041016815558072240280421831581}{1563549009235152659951616}\,a5+\frac{129775676408290972365175}{53058058420224}\right.$$

$$-\frac{2141630966918979471}{12272218112}\,a1-\frac{184307613782292360253}{5356823205888}\,a2$$

$$+\frac{2081918818989303715317112379}{691919426211729408}\,a3+\frac{125863093556214843305772971170567}{14162581605390875543040}\,a4$$

$$\left.-\frac{3540205991693456491321827822785360376641}{29441570987570316035883112857600}\,b\right)s^{8}+\left(\right.$$

$$-\frac{522621281837202714056230849178533}{71887310769432306204672}\,a5-\frac{966536526102229964086175}{1131905246298112}$$

$$+\frac{6272205967209354133}{49088872448}\,a1+\frac{1276248486077655798901}{3571215470592}\,a2$$

$$-\frac{59299769467907364816184487}{159061937060167680}\,a3-\frac{2872834071704185466309019860309}{976729765889025899520}\,a4$$

$$\left.+\frac{605369252655726504495722284279141}{363880496694726437225103360}\,b\right)s^{6}$$

$$+\left(\frac{32063024610839075026614701162275}{35943655384716153102336}\,a5+\frac{111206745695725802939425}{1131905246298112}\right.$$

$$-\frac{179479124647909437}{3506348032}\,a1-\frac{27124149382035508133}{127543409664}\,a2$$

$$-\frac{40040041644542013638789}{133665493327872}\,a3+\frac{32456063585219505315588061}{1479893584680342272}\,a4$$

$$\left.+\frac{1496636348558717251532761557493829}{272910372521044827918827520}\,b\right)s^{4}$$

$$+\left(\frac{6205831432379231361082126980509}{30808847472613845516288}\,a5+\frac{73332877267606645525}{4081389109248}\right.$$

$$+\frac{73660877816613217}{7012696064}\,a1+\frac{560757763227655542289}{10713646411776}\,a2$$

$$+\frac{11882743220175402344011573}{95437162236100608}\,a3+\frac{36612971483270871865616591849}{195345953177805179904}\,a4$$

$$\left.-\frac{67621117178102620676828283013787}{409365558781567241878241280}\,b\right)s^{2}-\frac{67713458127759425695}{16325556436992}$$

$$-\frac{39364048076763714568228701365833}{207880947818764615016294400}\,b-\frac{3742024602773169}{4382935040}\,a1$$

$$-\frac{4546739531426390521}{956575572480}\,a2-\frac{8331943598248202699653}{592381163612160}\,a3$$

$$\left.-\frac{6537223897482082717507636069}{226740838509952440960}\,a4-\frac{11460335042112204749062537050233}{250321885714987494819840}\,a5\right)$$

$$z^{10}+\left(\left(-\frac{944105318816033801}{91340544}\,a5-\frac{1794501042985}{2304}\,a3-\frac{57344748811393771}{14892480}\,a4\right.\right.$$

$$\left.-\frac{411936417190843147387067843}{3632511425760460800}\,b\right)s^{8}+\left(\frac{8583629748038023245413}{462670302208}\,a5\right.$$

$$-\frac{1172920086410625}{13631488}-\frac{148942233195}{2048}\,a2+\frac{46848975277396229}{58352640}\,a3$$

$$+ \frac{933571959572555920211}{150870750720} a4 + \frac{16451536980621573937907882291}{76666171702577725440} b \Big) s^{6} + \Big($$
$$- \frac{35171816665560250097433823}{15665091092158464} a5 + \frac{9638194567964612825}{32388415488} + \frac{1759608769009}{202752} a1$$
$$+ \frac{1104330358909183}{4561920} a2 + \frac{38521087310545404971}{34661468160} a3$$
$$+ \frac{109416189887279619028139}{141893941052160} a4 - \frac{24404538175020077024208536018909}{324470405188234578493440} b \Big) s^{4}$$
$$+ \Big(- \frac{55988764908769804275829426588009}{6018794304155848562688} a5 - \frac{3581641208129445475175}{11437665288192}$$
$$- \frac{568727881553897}{47241216} a1 - \frac{2900085089638749071}{12887994240} a2$$
$$- \frac{1248137950022561516403731}{783383841884160} a3 - \frac{231580482074625289232435651}{50108515026872940} a4$$
$$- \frac{24257473741648011039844306663610749}{442709679226590642915532800} b \Big) s^{2} + \frac{883381111890755751047683}{8913753814597632}$$
$$+ \frac{30437530108838534562836346088004370165961}{1062656702832616094420156104704000} b + \frac{12960342159615284551}{3221457254400} a1$$
$$+ \frac{44036250860193796086889}{703083045772800} a2 + \frac{463179630061524849634715891}{971277269414860800} a3$$
$$+ \frac{608223716428128934203023062772653}{3717677767141510483004800} a4$$
$$+ \frac{27410595624269084955000808458539449}{7462392998622319513405440} a5 \Big) z^{20}$$
$$+ \Big(\Big(\frac{179423034150428705836679}{30270928548003840} b + \frac{256357291855}{3072} a3 + \frac{353307764637691}{1985664} a4$$
$$+ \frac{29267523664225435}{121787392} a5 \Big) s^{4} + \Big(- \frac{279266687240625}{18743296} - \frac{35462436475}{2816} a2$$
$$- \frac{4757891592239183}{16046976} a3 - \frac{43941951433442464649}{41489456448} a4$$
$$- \frac{1548539880070128185835}{636171665536} a5 - \frac{126003356947620251497297111}{4791635731411107840} b \Big) s^{2}$$
$$+ \frac{10443430644531899}{983347200} a2 + \frac{2595013084975368378811}{14942944051200} a3$$
$$+ \frac{3366693621875931176457559}{4829372730547200} a4 + \frac{348372409490301081915177889}{204648328072642560} a5$$
$$+ \frac{2074910359073224902866491518997}{124185721682681321472000} b + \frac{1759608769009}{8110080} a1$$
$$+ \frac{313601480574950435}{21817196544} \Big) z^{24} + \Big(\Big(- \frac{3601891976167770019}{243574784} a5$$
$$- \frac{3230101877373}{2048} a3 - \frac{40608239037321949}{6618880} a4$$
$$- \frac{2496399853917314406009277}{13453746021335040} b \Big) s^{10} + \Big(\frac{44669401495205443032755}{925340604416} a5$$

$$- \frac{1172920086410625}{27262976} - \frac{148942233195}{4096}\, a2 + \frac{32120948069670173}{7780352}\, a3$$

$$+ \frac{3785050509075264771 09}{20116100096}\, a4 + \frac{8787538208692971956958425 0723}{153332343405155450880}\, b \Big) s^{8} + \Big($$

$$- \frac{1124378200666848910742856928913}{24312221375029936128}\, a5 + \frac{4189736387658378777 5}{120833703936}$$

$$+ \frac{22874913997117}{1081344}\, a1 + \frac{12161773044650300 89}{3540049920}\, a2$$

$$- \frac{1251648234500621807460 59}{53794598584320}\, a3 - \frac{1770580389341326651761912043}{110109698256476160}\, a4$$

$$- \frac{344669346156546304551062112459406 69}{5722478055137955293429 7600}\, b \Big) s^{6}$$

$$+ \Big(\frac{11663371242457616524580752880 5489}{1605011814441559616716 8}\, a5 - \frac{8727084057947664580937 5}{122001763074048}$$

$$- \frac{1895545033001893}{41992192}\, a1 - \frac{205901016418124908 09}{34367984640}\, a2$$

$$- \frac{3425698151383773230891989}{2089023578357760}\, a3 + \frac{2825473880725704809281905 7}{267245413476655680}\, a4$$

$$+ \frac{88228853885736278639444449749840915 37}{41555681890069308348338012160}\, b \Big) s^{4}$$

$$+ \Big(\frac{52825541916130314625987202633557 91}{59563771780386767998156 8}\, a5 + \frac{1852003392962710357695 5}{35372038946816}$$

$$+ \frac{2159173291135765177}{70126960640}\, a1 + \frac{1902981760694712082501}{5101736386560}\, a2$$

$$+ \frac{1469457677554485899263087 09}{77525986130165760}\, a3 + \frac{13068368190706572206555758391017}{2697634591503023912960}\, a4$$

$$+ \frac{22511806616037184925673399479691237451}{963860954950218679746173337600}\, b \Big) s^{2} - \frac{395149107444112499543 75}{318348350521344}$$

$$- \frac{557112589287020050873009841285213798 99}{276014728008471712836404183 0400}\, b - \frac{8230168293765611 7}{12272218112}\, a1$$

$$- \frac{3091453919334340672601}{40176174044160}\, a2 - \frac{4277363669293967595275244 23}{943526490288721920}\, a3$$

$$- \frac{6031571475723033199479289698073}{4248774481617262662912}\, a4$$

$$- \frac{12890022310644228449472365023800 05}{4264224570641325436231 68}\, a5 \Big) z^{18} + \Big(\Big(\frac{8357340619897567}{48400560}\, a4$$

$$+ \frac{51271458371}{1152}\, a3 + \frac{2467456270025299 39}{593713536}\, a5 + \frac{3854525256063501837897089}{7378538833575936 00}\, b \Big)$$

$$s^{2} - \frac{141775224922301}{2766720}\, a3 - \frac{20438048656998993167}{92993609280}\, a4$$

$$- \frac{1581893566087658647 61}{285180401792}\, a5 - \frac{13827321845278138654690 3}{23347550415421440}\, b - \frac{4255492377}{2816}\, a2$$

$$- \frac{33512002468875}{18743296} \Big) z^{26} + \Big(\Big(\frac{742803769223448017}{365362176}\, a5 + \frac{2409758543437}{9216}\, a3$$

$$+\frac{26922135087524321}{29784960}\,a4+\frac{10083356318430225156120209}{363251142576046080}\,b\Big)\,s^{20}+\Big($$

$$-\frac{28112273761454664 20405}{231335151104}\,a5+\frac{167560012344375}{3407872}+\frac{21277461885}{512}\,a2$$

$$-\frac{7309323715181569}{5835264}\,a3-\frac{38151060650771590031}{7543537536}\,a4$$

$$-\frac{12876119213903189111904140851}{76666171702577725440}\,b\Big)\,s^{18}+\Big(\frac{6148814626524783336828814093}{213265099780964352}\,a5$$

$$-\frac{1759608769009}{540672}\,a1-\frac{156062671683798551}{590008320}\,a2+\frac{19188651846984644532781}{8965766430720}\,a3$$

$$+\frac{202646064629365700919459437}{18351616376079360}\,a4+\frac{16701672706396272417652011231197009}{38149853700919701956198400}\,b$$

$$-\frac{228651212896019516225}{523612717056}\Big)\,s^{16}+\Big(-\frac{278775017410349820431641636733593}{8025059072207798083584}\,a5$$

$$+\frac{29817295773343}{62988288}\,a1+\frac{43181324970853865557}{68735969280}\,a2$$

$$-\frac{1268552355939757556137907}{1044511789178880}\,a3-\frac{119331758899769730829434191}{10229489511068160}\,a4$$

$$-\frac{303589591299919861517869916581880741}{472223657841696685776568320}\,b+\frac{89487097354795724749375}{61000881537024}\Big)\,s^{14}$$

$$+\Big(\frac{7492743830537196968969704350779121}{347455335385589479989248}\,a5-\frac{442012545065870098858485}{174139268661248}$$

$$+\frac{371732735606628591}{11156561920}\,a1-\frac{2102683085788140988119}{2976012892160}\,a2$$

$$-\frac{33251151899287751357612421}{45223491909263360}\,a3+\frac{25215746516124977071847368753551}{4720860535130291847680}\,a4$$

$$+\frac{16587513700586412775738843588450929 7181}{284459623068312232230754713600}\,b\Big)\,s^{12}+\Big($$

$$-\frac{5186512048299831604800391893156985}{1042366006156768439967744}\,a5+\frac{1425899182391298469056875}{565952623149056}$$

$$-\frac{1968222235366031619}{24544436224}\,a1+\frac{3013255228571325721777}{8928038676480}\,a2$$

$$+\frac{18230231163413046890 6818093}{135670475727790080}\,a3+\frac{377545499734441630064409676697}{944172107026058369536}\,a4$$

$$-\frac{3300762319551136786491769617374901835667}{9813856995856772011961037619200}\,b\Big)\,s^{10}+\Big($$

$$-\frac{25546173988321068577918260605605}{179718276923580765511 68}\,a5-\frac{3342645494083142054664625}{2263810492596224}$$

$$+\frac{1058107023382060405}{12272218112}\,a1+\frac{19335766938223432355}{446401933824}\,a2$$

$$-\frac{4753857078871524453657467}{7953096853008384}\,a3-\frac{11230363716851138702521401713}{8139414715741882496}\,a4$$

$$+\frac{23205884966305407704417558478228967}{189850693927683358552227840}\,b\Big)\,s^{8}$$

$$+\left(\frac{10529104679722050555362461338075}{1078309661541484593070 08}a5+\frac{16792265528221615313918 75}{3395715738894336}\right.$$
$$-\frac{176768651742155135}{3506348032}a1-\frac{984433863218108897 21}{765260457984}a2$$
$$-\frac{684372109569041598287 81}{6816940159721472}a3+\frac{39614937880468726829789470145}{97672976588902589952}a4$$
$$\left.-\frac{881390208981273274266966317841991 79}{32749244702525379350259302 40}b\right)s^6+\left(\right.$$
$$-\frac{12312160268820957497876055245390 5}{14018025600039299709911 04}a5-\frac{5608198097679819519601 25}{6791431477788672}$$
$$+\frac{576494884264610 65}{3506348032}a1+\frac{294181337036746654 01}{486983927808}a2$$
$$+\frac{422600623257698758731699 7}{47718581118050304}a3+\frac{52749970856811360119544014387}{1269748695655733669376}a4$$
$$\left.+\frac{156924684231502864872631755692411 9}{448147559087189401635127296}b\right)s^4+\left(\right.$$
$$-\frac{750697448674671439592270154178 3}{200257508571989995855872}a5+\frac{77281775538055967427 5}{212232233680896}$$
$$-\frac{9752201525856687}{3506348032}a1-\frac{9368708697373713113}{765260457984}a2$$
$$-\frac{137019777448888669257049}{5212954239787008}a3-\frac{66341774727578963116264376 93}{1813926708079619527 68}a4$$
$$\left.-\frac{185669423964414713418189726303816 1}{532175226416037414441713664 0}b\right)s^2+\frac{41176944104519703421 75}{11036076151406592}$$
$$+\frac{754628294186524548797403291689903 9}{206217900236214498096164044800}b+\frac{9205823971709647}{49088872448}a1$$
$$+\frac{1293190995013935912 85}{139277403353088}a2+\frac{27434667006904877649087 5}{112789373551755264}a3$$
$$\left.+\frac{56925777710210006883857743009}{126974869565573366937 60}a4+\frac{1670993489359452442692948441349}{2548731927279872674529 28}a5\right)$$
$$z^8+\left(\frac{753678487563403}{24200280}a4+\frac{1043636703136697}{12906816}a5\right.$$
$$\left.+\frac{341303902130113775491 37}{36892694167879680}b+\frac{51271458371}{7488}a3\right)z^{28}+\left(\left(\right.\right.$$
$$-\frac{842405922337617789661954 1}{302709285480038400}b-\frac{666528958823}{6144}a3-\frac{19658908499779231}{19856640}a4$$
$$\left.-\frac{759083370890498707}{243574784}a5\right)s^6+\left(-\frac{1396333436203125}{27262976}-\frac{177312182375}{4096}a2\right.$$
$$-\frac{10612138020590843}{23341056}a3-\frac{39949606102258097741}{60348300288}a4$$
$$\left.-\frac{225258076290836460615}{925340604416}a5-\frac{29723881184205158165496361}{19166542925644431360}b\right)s^4$$
$$+\left(\frac{256533367921167543 5}{24544346112}+\frac{15893594603008220475843421 57}{550548491282380800}a4\right.$$

$$+\frac{760856678279683739214836772587}{121561106875149680640}\,a5$$
$$+\frac{139392674631363952681195494706391}{2588291883491673858048000}\,b+\frac{33432566611171}{16220160}\,a1$$
$$+\frac{1411101443783798249}{17700249600}\,a2+\frac{23940297419830716495076 1}{268972992921600}\,a3\Big)\,s^{2}$$
$$-\frac{331766064880549}{236206080}\,a1-\frac{345548493243488959}{10310395392}\,a2$$
$$-\frac{55213037093836466282695}{156676768376832}\,a3-\frac{2105928721977284145807873223}{1603472480859934080}\,a4$$
$$-\frac{18570277425605860790594132349385}{6018794304155848562688}\,a5$$
$$-\frac{2128296133929234336598390524797429939}{77916903543879953153133772800}\,b-\frac{571581503360005778675}{11437665288192}\Big)\,z^{22}$$
$$+\Bigg(\bigg(\Big(\frac{303380449227836685}{121787392}\,a5-\frac{769071875565}{1024}\,a3-\frac{226411290669153}{661888}\,a4$$
$$-\frac{33542888505750672166267}{1681718252666880}\,b\Big)\,s^{14}+\Big(\frac{7904349943326722476605}{462670302208}\,a5$$
$$+\frac{3518760259231875}{13631488}+\frac{446826699585}{2048}\,a2+\frac{21275116795287635}{3890176}\,a3$$
$$+\frac{121569813109353941129}{10058050048}\,a4+\frac{140947070695501064201689279}{555551968859258880}\,b\Big)\,s^{12}+\Big($$
$$-\frac{1466382897795420834405442884583}{20260184479191613440}\,a5-\frac{131026656269918288705}{130903179264}$$
$$+\frac{86220829681441}{2703360}\,a1-\frac{1329379190058755941}{2950041600}\,a2$$
$$-\frac{539890945328552545759549}{44828832153600}\,a3-\frac{3337715073228624100404031913}{91758081880396800}\,a4$$
$$-\frac{394272537500287300336518120595461559}{524560488387645901897728000}\,b\Big)\,s^{10}$$
$$+\Big(\frac{3798141674704136985271343510917 81}{40125295361038990417 92}\,a5+\frac{33272552090916167941625}{30500440768512}$$
$$-\frac{3976375406660059}{31494144}\,a1-\frac{134352730996427527}{1562181120}\,a2$$
$$+\frac{56877666037833214564165 19}{522255894589440}\,a3+\frac{22170801472302852381288747809}{534490826953311360}\,a4$$
$$+\frac{81078027653069598347324527611448799951}{83111363780138616696676024320}\,b\Big)\,s^{8}+\Big($$
$$-\frac{38879620212743239756991439485439989}{754816763079039215149056}\,a5-\frac{532663271834975150581105}{11885005086130176}$$
$$+\frac{159857551800172691803}{859055267840}\,a1+\frac{161825623743379002125209}{187488812206080}\,a2$$
$$-\frac{305129493307146489605931031}{98244137595985920}\,a3-\frac{6001631430646874152487823202241}{310777652782871877120}\,a4$$

$$- \frac{2448483803111763568457895174575207739483 1}{39086223552464040254534477414400} b \Big) s^6$$

$$+ \Big(\frac{285015657600041029943805819204703 55}{36482810215486895398871 04} a5 - \frac{1162543127436217733150575}{1980834181021696}$$

$$- \frac{1107232234255091556 3}{85905526784} a1 - \frac{10030157494177258728959}{12499254147072} a2$$

$$- \frac{118808390073669688459023403}{94969333009453056} a3 + \frac{6799297927633082955393369859249}{6609204749182408586752} a4$$

$$+ \frac{3143170045497148434177397293535483011 29}{171742497427493510209318158336 0} b \Big) s^4$$

$$+ \Big(\frac{1291964558419258191190550027383255}{5032111753860261434327 04} a5 + \frac{415245096011052574843 25}{123802136313856}$$

$$+ \frac{131571360775529634 7}{31238373376} a1 + \frac{7384203489813755214101}{24998508294144} a2$$

$$+ \frac{210116112086849731508139433}{222686711884234752} a3 + \frac{2489292057468299055584607243175}{1367421672244636259328} a4$$

$$- \frac{1074781796152210296176881639097 3}{99498573314964260178739 2} b \Big) s^2 - \frac{5942469535604232053125}{106116116840448}$$

$$- \frac{2457867073165531192898607944170 57}{731009926395655789068288 00} b - \frac{7971804797258419}{1534027264} a1$$

$$- \frac{6505974895613980489 7}{1674007251840} a2 - \frac{2092595622258335999346 79}{1355641509035520} a3$$

$$- \frac{120250306454654628584915379 13}{30522805184032059360} a4 - \frac{45052417891453538913004267477 3}{612675944057661700608} a5 \Big)$$

$$z^{14} + \Big(\Big(\Big(- \frac{552820583732106487}{60893696} a5 - \frac{2512301460179}{1536} a3 - \frac{115898510993149 43}{2482080} a4$$

$$- \frac{61595415863940943103768 53}{41753004893798400} b \Big) s^{12} + \Big(\frac{245387783282579448951 87}{462670302208} a5$$

$$+ \frac{368632027157625}{3407872} + \frac{46810416147}{512} a2 + \frac{1327704448219248 97}{19450880} a3$$

$$+ \frac{373517492523853100 09}{1571570320} a4 + \frac{6755966800233174055700396297}{1022215622701036339 2} b \Big) s^{10} + \Big($$

$$- \frac{188354371455519032465065544915}{20260184479191613 44} a5 - \frac{146800790825603487125}{1047225434112}$$

$$+ \frac{8798043845045}{270336} a1 + \frac{1410557680775411}{14750208} a2 - \frac{817973977105411503850 9}{896576643072} a3$$

$$- \frac{1751436566853661388138389 7}{458790409401984} a4 - \frac{369673342194260201653208011483553 03}{3357187125680933772145459 2} b \Big)$$

$$s^8 + \Big(\frac{101339920298570958264095769869256 1}{160501181444155961671 68} a5 - \frac{5451577473987808876037 5}{122001763074048}$$

$$- \frac{1102525801279253}{11452416} a1 - \frac{98137766535304613449}{137471938560} a2$$

$$+ \frac{740242237489785558885365 9}{2089023578357760} a3 + \frac{9752617893581927131975332844 3}{427592661562649088 0} a4$$

$$+ \frac{7361136027499562834598807963783105349}{8748564608435643862808002560} b \Big) s^{6} + \Big($$

$$- \frac{23220407185123628036657991616218851}{2304177487293909183086592} a5 + \frac{85426360649926237837982545}{95080040689041408}$$

$$+ \frac{174330506020839007307}{1718110535680} a1 + \frac{323872453724719789867631}{374977624412160} a2$$

$$+ \frac{9491600859834603516401864429}{5698159980567183360} a3 - \frac{12365270480162101033104118867493}{11663302498557191623680} a4$$

$$- \frac{304559001230943174371918471125546444891757}{1133500483021457167381499845017600} b \Big) s^{4} + \Big($$

$$- \frac{13161043102261533071556139153801063}{2304177487293909183086592} a5 - \frac{476063202429380921975}{892803867648}$$

$$- \frac{15546343052791944861}{343622107136} a1 - \frac{13714736163814209007549}{34088874946560} a2$$

$$- \frac{152957303199223318269074719687}{96868719669642117120} a3$$

$$- \frac{696774613865475011621384062535147}{198276142475472257602560} a4$$

$$+ \frac{14484904119250552554015339500052229021}{2576137461412402653139772375040} b \Big) s^{2}$$

$$+ \frac{202784244169070101967675}{1980834181021696} + \frac{935830724272107716744985955661101}{95518630382365689771589632} b$$

$$+ \frac{2473403738877550583}{343622107136} a1 + \frac{1629842435776855435787}{24998508294144} a2$$

$$+ \frac{10474504132030101187810739}{33740410891550720} a3 + \frac{6019565731416738206488428116509}{6837108361223181296640} a4$$

$$+ \frac{80649623660578427748628638610043}{45746470489638740312064} a5 \Big) z^{16} + \Big(\Big(\frac{24697539753747161}{243574784} a5$$

$$+ \frac{358900208597}{6144} a3 + \frac{2114103027989533}{19856640} a4 + \frac{1110786937519617889066781}{302709285480038400} b \Big)$$

$$s^{22} + \Big(- \frac{1401195726028329887523}{925340604416} a5 + \frac{9929482213}{4096} a2 - \frac{45749870064257219}{116705280} a3$$

$$- \frac{290289271843835195981}{301741501440} a4 - \frac{1068047841590951772615511391}{38333085851288862720} b$$

$$+ \frac{78194672427375}{27262976} \Big) s^{20} + \Big(\frac{48264615077143081586912784625}{8104073791676645376} a5$$

$$- \frac{8541132276472749625}{130903179264} - \frac{8798043845045}{3244032} a1 - \frac{9753330585519745}{236003328} a2$$

$$+ \frac{3659997400905340939535}{3586306572288} a3 + \frac{133891309602378676767825}{42927757604864} a4$$

$$+ \frac{1914312308436364852954743642736003}{20982419535505836075909120} b \Big) s^{18} + \Big($$

$$- \frac{4969678190672094649916064444649}{445836615122655449088} a5 + \frac{98041363979221}{10498048} a1$$

$$+\frac{147611742726346429}{954666240}\,a2-\frac{76741963634398909383731}{58028432732160}\,a3$$
$$-\frac{75818362315799960508153289}{14846967415369760}\,a4-\frac{143351188117102376894331084085840633}{839508725051905219158343680}\,b$$
$$+\frac{1188307467722808677125}{3388937863168}\Bigg)s^{16}+\Bigg(\frac{13425021263259354714934038409108237}{1152088743646954591543296}\,a5$$
$$-\frac{6728177694683046589}{859055267840}\,a1-\frac{47971584028618899595447}{187488812206080}\,a2$$
$$+\frac{2428135034796185694922768877}{2849079990283591680}\,a3+\frac{463788541238460057789539626335977}{991380712377361288012 80}\,a4$$
$$+\frac{10473684583581412887813356126010269740679}{51522749228248053062795447500800}\,b$$
$$-\frac{10306494464623479526656335}{11885005086130176}\Bigg)s^{14}+\Bigg($$
$$-\frac{3682100259158406864171142750924513}{521183003078384219983872}\,a5+\frac{339260204166750083367275}{282976311574528}$$
$$-\frac{124280958050157207}{12272218112}\,a1+\frac{1867135342112576703413}{8928038676480}\,a2$$
$$-\frac{198461885100083957917028323}{1153199043686215680}\,a3-\frac{11242146572101555462464391677451}{4720860535130291847680}\,a4$$
$$-\frac{15849546880512058143361844350222918765 9}{98138569958567720119610376192 0}\,b\Bigg)s^{12}$$
$$+\Bigg(\frac{5644306283002077411203724956577 3}{2396243692314410206822 4}\,a5-\frac{565927544962845451457325}{565952623149056}$$
$$+\frac{1309051226877627129}{49088872448}\,a1-\frac{6883407071627971923}{108218650624}\,a2$$
$$-\frac{4152758878474462204609473}{5302064568672256 0}\,a3+\frac{11017993698911815121014861977}{1915156403703972352 0}\,a4$$
$$+\frac{459200103971661840052350116797483}{527363038688009329311744 0}\,b\Bigg)s^{10}+\Bigg($$
$$-\frac{8699860623827035581186176083 75}{2567403956051153793024}\,a5+\frac{223516564641729458146 25}{43534817165312}$$
$$-\frac{3895490170285155}{159379456}\,a1-\frac{4231733180409574 45}{15942926208}\,a2$$
$$+\frac{2180299985864791231421 5}{113615669328691 2}\,a3-\frac{14383480083802180198295875}{5813867654101344 64}\,a4$$
$$-\frac{4936654656864164464145600441534791}{1559487842977399016679014 40}\,b\Bigg)s^{8}$$
$$+\Bigg(\frac{2461566806229522018674943353 05}{6675250285732999861862 4}\,a5-\frac{897046284193629005826 25}{565952623149056}$$
$$+\frac{41777251471639855}{3506348032}\,a1+\frac{7771283123419576919}{255086819328}\,a2$$
$$+\frac{6402797486416327973708 3}{227231338657382 4}\,a3+\frac{413138192792702173248070617}{60464223602653984256}\,a4$$

$$+\frac{6116716273354995022234353521457467}{810933678348247488673087488}\,b\Big)\,s^{6}+\Big($$

$$-\frac{98413165147637434847316 0793}{114695022091632300032}\,a5+\frac{316285629915165630825}{11669126250496}$$

$$-\frac{5678455752551583}{1753174016}\,a1-\frac{19297766460644987}{1753174016}\,a2-\frac{50737762921462400019}{2985655349248}\,a3$$

$$-\frac{19130479528323911077963241}{1246685022735133696}\,a4-\frac{205856974721771749319630888917}{183336425743409180835840}\,b\Big)\,s^{4}$$

$$+\Big(\frac{9555699909115937788047096342769}{1869070080005239961321472}\,a5-\frac{950019744673007801425}{459836506308608}$$

$$+\frac{44881906120643019}{98177744896}\,a1+\frac{169098812271225528707}{92851602235392}\,a2$$

$$+\frac{2979760950430685777446799}{827122072712871936}\,a3+\frac{3699061898246701244551713989}{769544664033777981440}\,a4$$

$$+\frac{6873336420225906325443807620721647}{6873930007873816603205468 1600}\,b\Big)\,s^{2}+\frac{507654051110850342925}{19313133264961536}$$

$$-\frac{54777294198954918913216364775407}{10476630330405245867763302400}\,b-\frac{3305657466188027}{128858290176}\,a1$$

$$-\frac{13957419945364215403}{121867727933952}\,a2-\frac{2487811610129519750223167}{9259509968421672960}\,a3$$

$$-\frac{548282219357090058962571}{1221740898611192960}\,a4-\frac{21486952275543142510633241}{35186137880002357248}\,a5\Big)\,z^{6}+\Big(\Big($$

$$-\frac{794775067195725}{7611712}\,a5-\frac{9350294908185}{330944}\,a4-\frac{1344206495679472701759}{1793832802844672}\,b\Big)\,s^{24}$$

$$+\Big(\frac{2431919365239140782635}{5089373324288}\,a5-\frac{49647411065}{22528}\,a2-\frac{3005597409973361}{128375808}\,a3$$

$$+\frac{31681323364014353869}{331915651584}\,a4+\frac{351773275037300197746914753}{76666171702577725440}\,b$$

$$-\frac{390973362136875}{149946368}\Big)\,s^{22}+\Big(-\frac{12137085246706788377881759433}{15195138359393710080}\,a5$$

$$-\frac{1759608769009}{2027520}\,a1+\frac{1949104575374873}{276566400}\,a2+\frac{4023855988878306840301}{33621624115200}\,a3$$

$$-\frac{40012817472872357064217}{1075290022035900}\,a4-\frac{38526672115170158062875634817414647}{3147362930325875411386368000}\,b$$

$$+\frac{40120814245511760565}{3141676302336}\Big)\,s^{20}+\Big(\frac{4846366220113499728547565152593}{12037588608311697125376}\,a5$$

$$-\frac{394386330288648620125}{91501322305536}+\frac{391521739383593}{94482432}\,a1-\frac{256919116385877757}{103103953920}\,a2$$

$$-\frac{400190177988918798653893}{1566767683768320}\,a3-\frac{930728303800532445336470321}{3206944961719868160}\,a4$$

$$+\frac{4587001135346286448725777522357031073}{2493340913404158500900280728 72960}\,b\Big)\,s^{18}$$

$$+\Big(\frac{6829499305353342850578537737981}{13478870769268557413376}\,a5-\frac{116771782686372307}{15340272640}\,a1$$

$$- \frac{5334387036135268451}{304364954880} a2 + \frac{514105701511595517837199}{1754359599928320} a3$$

$$+ \frac{38038557625207478494493430709}{61045610368064118720} a4$$

$$- \frac{33438365717328334213912173464910939797}{2030453171556573519716076748800} b - \frac{277891103225441292661955}{3395715738894336} \Bigg)$$

$$s^{16} + \Bigg(- \frac{378620386511760799578991635375745}{390887252308788164987904} a5 + \frac{17959862493168609}{3068054528} a1$$

$$+ \frac{80336588539530047345}{2678411602944} a2 - \frac{33620299954115158468591835}{172979856552932352} a3$$

$$- \frac{864158569547247137652405197287}{1416258160539087554304} a4$$

$$+ \frac{5794964336322014238516854255335309127}{736039274689257900897077821440} b + \frac{375858746571079819105525}{1697857869447168} \Bigg) s^{14}$$

$$+ \Bigg(\frac{921230934251023240808879684045}{1283701978025576896512} a5 - \frac{639899067928315806565325}{2263810492596224}$$

$$+ \frac{370823422986211}{876587008} a1 - \frac{562481358912547621}{31885852416} a2$$

$$+ \frac{44045467932573644778067}{568078346643456} a3 + \frac{18081669815735369160811733}{52853342310012224} a4$$

$$- \frac{81099728244602701455374154888151}{311897568595479803335802880} b \Bigg) s^{12} + \Bigg($$

$$- \frac{379660942352542462939946845217}{1283701978025576896512} a5 + \frac{119570254282553650543625}{565952623149056}$$

$$- \frac{3889240563830583}{876587008} a1 - \frac{28421943197599613}{19928657760} a2$$

$$- \frac{69317352952895935621931}{2840391733217280} a3 - \frac{172239690378149674608212233}{1453466913525336160} a4$$

$$- \frac{1742487296045589656896014010769453}{779743921488699508339507200} b \Bigg) s^{10}$$

$$+ \Bigg(\frac{1854232140360614086112681731625}{2503218857149874948 1984} a5 - \frac{330211386450889390188875}{3395715738894336}$$

$$+ \frac{1640805975321625}{438293504} a1 + \frac{724396688567748181}{95657557248} a2$$

$$+ \frac{10112420541363780178387}{852117519965184} a3 + \frac{337826390821239943275189421}{11337041925497622048} a4$$

$$+ \frac{14773695389969903469323948177625893}{9731204140178969864077049856} b \Bigg) s^{8} + \Bigg($$

$$- \frac{168463307533104253613671923955}{1251609428574937474 0992} a5 + \frac{23173860419696944496525}{848928934723584}$$

$$- \frac{686715272794341}{438293504} a1 - \frac{3334225046645980439}{765260457984} a2 - \frac{125036362678724471827}{2036310249168} a3$$

$$- \frac{1444814127086447108086971439}{181392670807961952768} a4 - \frac{476298917677353802932000464116663}{935692705786439410007408640} b \Bigg)$$

$$
s^{6} + \left(\frac{122227111394991403044054325}{47748571428705292288}\, a5 - \frac{85295053002959311575}{19431849721856}
$$
$$
+ \frac{246113402031}{684032}\, a1 + \frac{53815496339249}{45616384}\, a2 + \frac{39844172543522201611}{21130238930944}\, a3
$$
$$
+ \frac{71456026953846832812709}{32437888198848704}\, a4 + \frac{4250169972508117243613945645207}{44955193184541616861347840}\, b \right) s^{4} + \Big(
$$
$$
- \frac{1192233541697295123489215}{2741776977662521344}\, a5 + \frac{2867174673834347448925}{8277057113554944}
$$
$$
- \frac{1565084010465653}{36816654336}\, a1 - \frac{16345782980114543141}{104458052514816}\, a2
$$
$$
- \frac{457860648725995344864263}{1587344566015143936}\, a3 - \frac{21629032753712895642337}{57120353701302528}\, a4
$$
$$
- \frac{2066890988944276580394296518777}{224499221365826697166356480}\, b \Big) s^{2} - \frac{31928953857107840825}{3820180206256128}
$$
$$
+ \frac{33513615377539410098246587}{84302348904162571714560}\, b + \frac{9085179471875}{4602081792}\, a1
$$
$$
+ \frac{120820074211689137}{15066065266560}\, a2 + \frac{1021279748898570437}{60264261066240}\, a3 + \frac{553480296551207647}{21343592460960}\, a4
$$
$$
+ \frac{130473901847494775011}{3893071264879104}\, a5 \Big) z^{4} + \Big(\Big(- \frac{176842213950432793}{9499416576}\, a5
$$
$$
- \frac{358900208597}{239616}\, a3 - \frac{5470235547034069}{774408960}\, a4 - \frac{4935526789816091162984983}{23611324267442995200}\, b \Big)
$$
$$
s^{26} + \Big(\frac{1220118010663701507485}{10178746648576}\, a5 - \frac{167560012344375}{299892736} - \frac{21277461885}{45056}\, a2
$$
$$
+ \frac{2013002278537273}{256751616}\, a3 + \frac{28721957964951609289}{663831303168}\, a4
$$
$$
+ \frac{244885121632017362680336459}{153332343405155450880}\, b \Big) s^{24} + \Big(
$$
$$
- \frac{40919729595057019097215769273}{121561106875149680640}\, a5 - \frac{1759608769009}{16220160}\, a1
$$
$$
+ \frac{46284727633235329}{17700249600}\, a2 - \frac{4392180160343988060419}{268972992921600}\, a3
$$
$$
- \frac{5710576055431534907182073}{50049862843852800}\, a4 - \frac{17369247530027242758959895730593449}{3147362930325875411386368000}\, b
$$
$$
+ \frac{7411909787446532515}{1570838151168} \Big) s^{22} + \Big(\frac{25999777150922162329886106436363}{48150354433246788501504}\, a5
$$
$$
+ \frac{1198589065124503}{1889648640}\, a1 - \frac{619912130226855673}{103103953920}\, a2
$$
$$
+ \frac{99960516690278404407017}{6267070735073280}\, a3 + \frac{54100122718239250299976267}{320694496171986816}\, a4
$$
$$
+ \frac{14181778209071538455601647749831742881}{1246670456702079250450140364800}\, b - \frac{470277975197151452975}{33273208111104} \Big) s^{20}
$$
$$
+ \Big(- \frac{142669504101226736420356339041966521}{262676233551505646871871488}\, a5 + \frac{5950845078355914559275}{3241365023490048}
$$

$$- \frac{3154597038550799153}{2061732642816} a1 + \frac{1675095240081147331058 9}{2249865746472960} a2$$

$$- \frac{14516101680320284867421 8099}{341889598834031 00160} a3 - \frac{1061848346945543754686175826 8383}{699798149913431497420 80} a4$$

$$- \frac{2109877788570061313619567566698807178 6863}{136020057962574860085779981402 1120} b \Bigg) s^{18}$$

$$+ \Bigg(\frac{32110242268152892771388366134 4813}{912070255387172384971776} a5 + \frac{20591713640985825}{10738190848} a1$$

$$- \frac{347488504956332316877}{62496270735360} a2 - \frac{1364329568270041992825 4207}{20180983264508774 40} a3$$

$$+ \frac{27333341406606617658598053 15299}{330460237459120429337 60} a4$$

$$+ \frac{591686459944146149616715447979724 69943}{4040999939470435534336897843200} b - \frac{37901526279968379 3025}{90037917319168} \Bigg) s^{16}$$

$$+ \Bigg(- \frac{71987725894307547781758867371525}{50321117538602614343 2704} a5 - \frac{4166145356869211 95}{343622107136} a1$$

$$+ \frac{7767845013755984172 5}{24998508294144} a2 + \frac{6693614611056755050957 99}{74228903961411584} a3$$

$$- \frac{318054661826379306064650415 45}{136742167224463625932 8} a4$$

$$- \frac{37125893568862032041582180979855359}{382074521529462759086358 5280} b - \frac{136510905990831938318 75}{720303338553344} \Bigg) s^{14}$$

$$+ \Bigg(\frac{478416679662813226905078880507}{154044237363069227581 44} a5 + \frac{878377143416771802492 5}{308701430808576}$$

$$+ \frac{509777644010687}{3506348032} a1 - \frac{396707666483911716 1}{191315114496 0} a2$$

$$- \frac{211478827708951747348439}{34084700798607360} a3 - \frac{35624740235108023280643599}{3488320592460806784 0} a4$$

$$+ \frac{10535195464659937793313899874381637}{233923176444660985250185216 00} b \Bigg) s^{12}$$

$$+ \Bigg(\frac{443044986622019057845517298895}{280360512000785994198220 8} a5 - \frac{31453196860608176661 25}{154350715404288}$$

$$+ \frac{2424012818392313}{7012696064} a1 + \frac{170634256025131387 85}{10713646411776} a2$$

$$+ \frac{315114294004282823155205}{9543716223610060 8} a3 + \frac{10159216361079395603271936241}{2539497391311467338752} a4$$

$$- \frac{240877987765798173616645080036195989}{17029607245313197262134837248 0} b \Bigg) s^{10} + \Bigg($$

$$- \frac{2355459732956191504219550463 41}{100128754285994997927936} a5 + \frac{265053283989650746712 5}{308701430808576}$$

$$- \frac{478708770867015}{1753174016} a1 - \frac{1371803153733281539}{1530520915968} a2$$

$$- \frac{380394519380864690174 3}{2606477119893504} a3 - \frac{660063036019318291684820 89}{329804856014476277 76} a4$$

$$+ \frac{683405024056481658553933115424955 3}{243280103504474246601926246 40} b \Big) s^8$$

$$+ \Big(\frac{45138980838849420957017073735 67}{560721024001571988396441 6} a5 - \frac{175123464413633239591 25}{802623720102297 6}$$

$$+ \frac{964920214515885 5}{9817774489 6} a1 + \frac{8642097688759527884 9}{27855480670617 6} a2$$

$$+ \frac{117975662048831153406179 3}{248136621813861580 8} a3 + \frac{517650768918902430551193467 9}{8464991304371557795840} a4$$

$$- \frac{3903379448221745583862878911405187 61}{1319794561511772787815449886720 0} b \Big) s^6 + \Big($$

$$- \frac{12928601832498520431779370 5}{844467309120056573952} a5 + \frac{537145203488427667853 75}{168550981221482496} $$

$$- \frac{195853779454635 85}{1030866321408} a1 - \frac{2260663443340781247 5}{365603183801856} a2$$

$$- \frac{436169779821386809279230 7}{4444564784842403020 8} a3 - \frac{53720599626913635413505 7}{439826723500029465 6} a4$$

$$+ \frac{2588925086914524524521940512730 9}{502878255859451801652638515 20} b \Big) s^4 + \Big(\frac{300510829154679784301 95}{1744095926665838592} a5$$

$$- \frac{153387290376039872525}{6685315360948224} + \frac{1280274493429641}{687244214272} a1 + \frac{783194387179855685}{122719949807616} a2$$

$$+ \frac{592755944848492462 09}{5399677791535104} a3 + \frac{270068932588932309 89}{1912385884502016} a4$$

$$+ \frac{106878180614990953734520138 9}{590116442329138002001920 0} b \Big) s^2 + \frac{421781504143275}{1276310683648}$$

$$- \frac{6032958911414509084 91}{4135485186939617280 0} b - \frac{8398429821247}{117143900160} a1 - \frac{82713272877511}{322145725440} a2$$

$$- \frac{8230701001458713}{16751577722880} a3 - \frac{724440186163339}{1046973607680} a4 - \frac{4972136705560906 1}{56955364257792} a5 \Big) z^2$$

$$+ \Big(\frac{2285627644223}{21511360} a4 + \frac{38855669951791}{98952256} a5 + \frac{492875715082473323978 3}{174898698277355520 0} b \Big) s^{28}$$

$$+ \Big(\frac{804288059253}{19450880} a3 - \frac{91113110191672331}{163443313280} a4 - \frac{1415092065737077030 1}{6014713928704} a5$$

$$- \frac{893544262456462737489750 5}{398664092853404172288} b \Big) s^{26} + \Big(\frac{26032058192281225}{241667407872}$$

$$+ \frac{12317261383063}{885012480} a2 - \frac{89408254616520259}{420270301440} a3 + \frac{30411213590641451224 09}{250249314219264 0} a4$$

$$+ \frac{116617749164761828663939 7}{18993922949242137 6} a5 + \frac{2029171339133049068444633370573 79}{251789034426070032910909440 0} b \Big) s^{24}$$

$$+ \Big(\frac{13590944081}{3817472} a1 - \frac{174531436403935}{2499489792} a2 + \frac{1916988828640643209 03}{417804715671552} a3$$

$$- \frac{118250631369817025326531 3}{855185323125298176} a4 - \frac{146416423207376755392197121815}{160501181444155961671 68} a5$$

$$- \frac{144077893091121534913141917829418731}{831113637801386166966760243200} b - \frac{524123950801315025}{853159182336} \Big) s^{22} + \Big($$

$$-\frac{13131625980683639}{780959334400}\,a1+\frac{25680175797671032469}{170444374732800}\,a2$$
$$-\frac{1357762598511434127980929}{2590072718439628800}\,a3+\frac{72604363819274012139064140473}{90125519307032844364800}\,a4$$
$$+\frac{168311247159855446554124054757469}{19899714662992852035747840}\,a5$$
$$+\frac{877482677472396616190925916862716567}{349899824979613263584349388800 0}\,b+\frac{954732176039892354479}{664895389433856}\Bigg)s^{20}+\Bigg($$
$$-\frac{416787870256450690675}{249335771037696}+\frac{962961474323277}{31238373376}\,a1-\frac{3889316605702315463}{20453324967936}\,a2$$
$$+\frac{94316590408318161371665}{310808726212755456}\,a3-\frac{7981999031512751503246016431}{540753115842197066188 80}\,a4$$
$$-\frac{6037863714575111319992269011841}{11939828797795711221448704}\,a5$$
$$-\frac{162529541446894751810807918138625272581}{6182729907389766367535453700009600}\,b\Bigg)s^{18}+\Bigg(-\frac{743057389846629}{31238373376}\,a1$$
$$+\frac{130038276777201169}{757530554368}\,a2-\frac{5900256663552258659}{1984730052444160}\,a3$$
$$-\frac{356777959865655971032713}{12187358932661642240}\,a4+\frac{30546245016134595001594509623}{152488234965462467706 88}\,a5$$
$$+\frac{2165969331194818855277730746671717}{10188653907452340242302894080}\,b+\frac{84587865630642519225}{110815898238976}\Bigg)s^{16}+\Bigg($$
$$-\frac{4896505627299}{2231312384}\,a1-\frac{21990774987197731}{162327975936}\,a2-\frac{13453659148519405763}{85059859390464}\,a3$$
$$-\frac{291257831841736226060051}{2959787169360684544}\,a4-\frac{2062362194613512007268819565}{3267605034974195736576}\,a5$$
$$-\frac{19028931177194783584377274075591}{136455186260522413959413760}\,b+\frac{3510138032865567475}{7915421302784}\Bigg)s^{14}+\Bigg($$
$$-\frac{545156592637391271775}{617402861617152}+\frac{6325110696861}{318758912}\,a1+\frac{6849529600705049}{69569132544}\,a2$$
$$+\frac{5821886315410353799}{36454225453056}\,a3+\frac{2792629542268610775395797}{16490242800723813888}\,a4$$
$$+\frac{4931863234486651567104990901}{18205228051999090532352}\,a5$$
$$+\frac{3670271850535744595118252918322013}{48656020700894849320385249280}\,b\Bigg)s^{12}+\Bigg(\frac{31061431647334657415}{51450238468096}$$
$$-\frac{28193191292871}{1593794560}\,a1-\frac{6641593645393403}{115948554240}\,a2-\frac{73945730065559689219}{789841551482880}\,a3$$
$$-\frac{3261966043702153666480707}{27483738001206356480}\,a4-\frac{2388277764898544124677 26351}{15969498291227272396 80}\,a5$$
$$-\frac{1324622767443204647414595748182973}{40546683917412374433654374400}\,b\Bigg)s^{10}+\Bigg(-\frac{95305751966826070025}{411601907744768}$$
$$+\frac{18074908083573}{2231312384}\,a1+\frac{235169859437969}{10145498496}\,a2+\frac{40068310450439295361}{1105778172076032}\,a3$$

$$+ \frac{143957456524089958993367}{3006033843881945240}\, a4 + \frac{2671151409140156437316079151}{4247886545466454457548 8}\, a5$$

$$+ \frac{37620446650652676205427230527956051}{35194521640313941008411996979 20}\, b \Big) s^8 + \Big(\frac{683374727970178405075}{12965460093960192}$$

$$- \frac{66917738846349}{31238373376}\, a1 - \frac{1223373123935560 45}{20453324967936}\, a2$$

$$- \frac{2041867267503818543430571}{2222282392421201510 40}\, a3 - \frac{1020863439822337729728193}{879653447000005893120}\, a4$$

$$- \frac{1454628371220077319133105 3}{84446730912005657395 2}\, a5 - \frac{24964199558310569072266498184809 21}{100575651171890360330527703040 0}\, b \Big)$$

$$s^6 + \Big(- \frac{1911302618685041273425}{285240122067124224} + \frac{10097819568581}{31238373376}\, a1$$

$$+ \frac{1005548316762974053}{1124932873236480}\, a2 + \frac{662117336039235751 3}{4499731492945920}\, a3$$

$$+ \frac{2497316340954742919}{1593654903751680}\, a4 + \frac{843308631531696169487}{2906826544443064 32}\, a5$$

$$+ \frac{6105711717949740178970882297}{15736438462110346720051200}\, b \Big) s^4 + \Big(\frac{739917952189785}{2552621367296}$$

$$- \frac{4417914728387}{156191866880}\, a1 - \frac{5152749235147}{85905526784}\, a2 - \frac{619533154463441}{4467087392768}\, a3$$

$$- \frac{141884134677099}{1395964810240}\, a4 - \frac{19345949397592305}{75940485677056}\, a5 - \frac{5978442834107661486 71}{14336348648057339904}\, b \Big) s^2$$

$$+ \frac{1006563529}{478822400}\, a1 + \frac{40247363}{51302400}\, a2 + \frac{26313817147}{5267046400}\, a3 + \frac{341948006306762087}{78481369055232000}\, b \Big)$$

$$\sin(2 \arctan(s \sin(\phi), s \cos(\phi))) - \frac{4754806245189}{43778048} \cos(2 \arctan(s \sin(\phi),$$

$$s \cos(\phi))) \Big(\frac{23}{3} z^{20} + \Big(70 s^2 - \frac{350}{9} \Big) z^{18} + \Big(285 s^4 - \frac{950}{3} s^2 + \frac{10925}{129} \Big) z^{16}$$

$$+ \Big(680 s^6 - \frac{3400}{3} s^4 + \frac{78200}{129} s^2 - \frac{547400}{5289} \Big) z^{14} + \Big(1050 s^8 - \frac{7000}{3} s^6 + \frac{80500}{43} s^4$$

$$- \frac{1127000}{1763} s^2 + \frac{5353250}{68757} \Big) z^{12} + \Big(1092 s^{10} - \frac{9100}{3} s^8 + \frac{418600}{129} s^6 - \frac{2930200}{1763} s^4$$

$$+ \frac{2141300}{5289} s^2 - \frac{7280420}{195693} \Big) z^{10} + \Big(770 s^{12} - \frac{7700}{3} s^{10} + \frac{442750}{129} s^8 - \frac{12397000}{5289} s^6$$

$$+ \frac{58885750}{68757} s^4 - \frac{400423100}{2544009} s^2 + \frac{28601650}{2544009} \Big) z^8 + \Big(360 s^{14} - 1400 s^{12} + \frac{96600}{43} s^{10}$$

$$- \frac{3381000}{1763} s^8 + \frac{21413000}{22919} s^6 - \frac{218412600}{848003} s^4 + \frac{31201800}{848003} s^2 - \frac{1485800}{717541} \Big) z^6$$

$$+ \Big(105 s^{16} - \frac{1400}{3} s^{14} + \frac{112700}{129} s^{12} - \frac{1577800}{1763} s^{10} + \frac{37472750}{68757} s^8$$

$$- \frac{509629400}{2544009} s^6 + \frac{36402100}{848003} s^4 - \frac{10400600}{2152623} s^2 + \frac{1300075}{6066483} \Big) z^4 + \Big(\frac{50}{3} s^{18}$$

$$- \frac{250}{3} s^{16} + \frac{23000}{129} s^{14} - \frac{1127000}{5289} s^{12} + \frac{10706500}{68757} s^{10} - \frac{182010500}{2544009} s^8$$

$$+ \frac{52003000}{2544009} s^{6} - \frac{7429000}{2152623} s^{4} + \frac{1857250}{6066483} s^{2} - \frac{109445270587687250}{14264418735567} \Big) z^{2} + s^{20}$$

$$- \frac{50}{9} s^{18} + \frac{575}{43} s^{16} - \frac{32200}{1763} s^{14} + \frac{1070650}{68757} s^{12} - \frac{7280420}{848003} s^{10} + \frac{2600150}{848003} s^{8}$$

$$- \frac{1485800}{2152623} s^{6} + \frac{185725}{2022161} s^{4} - \frac{21889054117537450}{4754806245189} s^{2} + \frac{4377804868292675}{679258035027} \Big) \Big)$$

$$\sqrt{5} \sqrt{13}\, s\bar{e}_{s} - \frac{3}{2080000000} \sqrt{2} \Big(\Big(b \Big(s^{2} + z^{2} - \frac{21}{25} \Big) \ln(2) + \Big(\Big($$

$$- \frac{101568759032951}{61440} a3 - \frac{279778166898313643}{39713280} a4 - \frac{8656651160034898055}{487149568} a5$$

$$- \frac{1276114236943712483087283457}{6054185709600768000} b \Big) z^{20} + \Big(\frac{625557379419}{2048} a2$$

$$+ \frac{10305037468346929}{1341440} a3 + \frac{10701795249999833917}{346829312} a4$$

$$+ \frac{2416762535159179805955}{31908296704} a5 + \frac{7041655207295499854532798749}{88122036439744512 00} b$$

$$+ \frac{4926264362924625}{13631488} \Big) z^{18} + \Big(- \frac{36951784149189}{3604480} a1 - \frac{602822647238744499}{437043200} a2$$

$$- \frac{99982917282714494317191}{6641308467200} a3 - \frac{2265197787887976719705610501}{40781369724620800} a4$$

$$- \frac{391506226095584028156865969797}{3001508811732090880} a5$$

$$- \frac{17622991253053870624382910818404453}{17269481099181758087168000} b - \frac{177349176411335738145}{116358381568} \Big) z^{16}$$

$$+ \Big(\frac{916033337680513}{20996096} a1 + \frac{11915656886484801623}{4582397952} a2$$

$$+ \frac{1122897856797021367776407}{69634119278592} a3 + \frac{7441091556942671790744061387}{142530887187549696} a4$$

$$+ \frac{302116421100926701516759894783385}{2675019690735932694528} a5$$

$$+ \frac{12613275547478310062281150286328469}{63395395713301767121797120} b + \frac{2557547586265819175875}{924255780864} \Big) z^{14} + \Big($$

$$- \frac{93625929223896470427}{1227221811200} a1 - \frac{78685699285552382717753}{29760128921600} a2$$

$$- \frac{4618865803184780670204555197}{452234919092633600} a3 - \frac{248721749168113306893563346962823}{944172107026058369536 0} a4$$

$$- \frac{162334930562102769773455830740112583}{3474553353855894799892480} a5$$

$$+ \frac{5976071885479917697126294522992717588871}{79964760706981105282645491 71200} b$$

$$- \frac{327109044214368233751671 7}{1131905246298112} \Big) z^{12}$$

$$+ \Big(\frac{2127168169929077335792301462196067}{7445471472548345999769 60} a5$$

$$
- \frac{5582498272896778093381030025566240992229}{7009897854183408579972169728000}\,b + \frac{1235726345929911849}{17531740160}\,a1
$$
$$
+ \frac{9934099890264027564707}{6377170483200}\,a2 + \frac{6350908370062716852367733761}{1647427205266022400}\,a3
$$
$$
+ \frac{20082839596411018879315871348867}{3372043239378779891200}\,a4 + \frac{1112794970173271370423305}{565952623149056}\Big) z^{10}
$$
$$
+ \Big(- \frac{3587697089968864899}{98177744896}\,a1 - \frac{1252774992954142680865}{2380810313728}\,a2
$$
$$
- \frac{87908131014847416014256629}{106041291373445120}\,a3 + \frac{27674489247356328881324886381}{130230635451870119936}\,a4
$$
$$
+ \frac{236882663443938819343210912005265}{47924873846288204136448}\,a5
$$
$$
+ \frac{144896092451334350631589558348047851}{428094701993795808500121600}\,b - \frac{2100367249741902564538725}{2263810492596224}\Big) z^{8}
$$
$$
+ \Big(\frac{35679868436232969}{3506348032}\,a1 + \frac{116541762449372845201}{1275434096640}\,a2
$$
$$
+ \frac{1050271479932412224798173}{11361566932869120}\,a3 - \frac{7904200179756327288288675199}{23255470616405378560}\,a4
$$
$$
- \frac{8904374892971563536163449962969}{5134807912102307586048}\,a5
$$
$$
- \frac{42981254401240090782563830553287823}{779743921488699508339507200}\,b + \frac{172632573138831567981275}{565952623149056}\Big) z^{6}
$$
$$
+ \Big(- \frac{8213244624837405}{7012696064}\,a1 - \frac{855013814069085007}{170057879552}\,a2
$$
$$
- \frac{8179208361701046588139}{1514875591049216}\,a3 + \frac{4890293281114391166183604317}{120928447205307968512}\,a4
$$
$$
+ \frac{7998995294380810559630695936615}{44501668571553332412416}\,a5
$$
$$
- \frac{123781263716941910288262036748445}{56907626550754209731444736}\,b - \frac{74211867953012968051125}{1131905246298112}\Big) z^{4} + \Big(
$$
$$
- \frac{757815504685353}{17531740160}\,a1 - \frac{60647120984737247}{159429262080}\,a2 + \frac{2399870056636384758247}{8688257066311680}\,a3
$$
$$
+ \frac{82854885111305512799361839}{151160559006634960640}\,a4 + \frac{139777035843516619776787805713}{333762514286649993093120}\,a5
$$
$$
+ \frac{43125368737057625179108616142254483}{40546683917412374433654374400}\,b + \frac{2260812939782541733085}{282976311574528}\Big) z^{2}
$$
$$
- \frac{2157205771413244050665}{5350824800681984} + \frac{3201515373240215290928810421624157487}{87986304100784852521029992448000}\,b
$$
$$
+ \frac{14657490113179539}{981777448960}\,a1 + \frac{19626934116052510327}{928516022353920}\,a2
$$
$$
+ \frac{34822784810160621439547}{1654244145425743872}\,a3 + \frac{3447982436509614500012757363}{846499130437155779584000}\,a4
$$
$$
+ \frac{1145165426816496971274048961913}{18690700800052399613214720}\,a5\Big) s^{8} + \Big(\Big(\Big(- \frac{19021711055641}{15360}\,a3
$$

$$- \frac{273817536042947813}{49641600} a4 - \frac{864254636564604 9101}{608936960} a5$$

$$- \frac{9942383009074490414688 4811}{605418570960076800} b \Big) z^{18} + \Big(\frac{21077453743281}{40960} a2$$

$$+ \frac{51550615095177921}{13414400} a3 + \frac{488292053669387309049}{34682931200} a4$$

$$+ \frac{1050000211031377315085 1}{319082967040} a5 + \frac{37671126616268790811044 0941}{29374012146581504 00} b$$

$$+ \frac{2553614588128275}{4194304} \Big) z^{16} + \Big(- \frac{142528310289729}{4505600} a1 - \frac{10705370369632709 19}{546304000} a2$$

$$- \frac{3223027344116105297249 1}{8301635584000} a3 - \frac{28542440296983313521699 0201}{50976712155776000} a4$$

$$- \frac{62741270305425021869168 18097}{3751886014665113600} a5$$

$$+ \frac{33327680999545674498763930539489223}{323802770609657964134400 00} b - \frac{32765844677158992357}{14544797696} \Big) z^{14}$$

$$+ \Big(\frac{29847026581290373}{262451200} a1 + \frac{691485962761653627 47}{22911989760} a2$$

$$+ \frac{68328347053196120481787}{174085298196480} a3 - \frac{58929119082816245128765735633}{356327217968874240 0} a4$$

$$- \frac{834060804196102124063567 44272631}{1337509845367966347264} a5$$

$$- \frac{1690840231926083768733112149693739025411}{6925946981678218058056335360 00} b + \frac{17701524451145558285465}{5083406794752} \Big)$$

$$z^{12} + \Big(\frac{4006780539986220794805306225059606059}{5211830030783842199838 7200} a5$$

$$+ \frac{557104150207330184599883991093515804947999}{2453464248964193002990259404 80000} b$$

$$- \frac{1007684437359365069187}{6136109056000} a1 - \frac{10662678038614060595310 91}{446401933824000} a2$$

$$+ \frac{13513072477557016812549936281}{678352378638950 4000} a3$$

$$+ \frac{5594118118973970655979612971045553}{23604302675651459238 4000} a4 - \frac{841558850149753355057 0291}{2829763115745280} \Big) z^{10}$$

$$+ \Big(\frac{192717825582332349}{1593794560} a1 + \frac{4288072168774637762037}{4251446988800} a2$$

$$- \frac{8174474837909014439910794 07}{549142401755340800} a3 - \frac{17772296465961856415292681306357}{1348817295751511956480} a4$$

$$- \frac{2024133528447545619036999700621329}{4963647648365563999846 4} a5$$

$$- \frac{7690935909715835115969512115986125417 07}{77887753935371206444135219 2000} b$$

$$+ \frac{178536977836702097788507 5}{1131905246298112} \Big) z^{8} + \Big(- \frac{1154546193237454668 3}{245444362240} a1$$

$$- \frac{1220537016609838946969}{5952025784320} a2 + \frac{111075312266159564164840211}{265103228433612800} a3$$

$$+ \frac{5446743625857649968021209988657}{1627882943148376499200} a4$$

$$+ \frac{52539407919319342498114340947423}{5209225418074804797440} a5$$

$$+ \frac{275940276624102333790313749479384137}{1819402483473632186125516800} b - \frac{28540862264870903320995}{51450238468096} \Big) z^6$$

$$+ \Big(\frac{9508751732758239}{1095733760} a1 + \frac{22994692050802057983}{2125723494400} a2$$

$$- \frac{86926721289544353173643}{2366993111014400} a3 - \frac{3099883357536138211962974589}{10570668462002444800} a4$$

$$- \frac{918072555781822860313752293151}{1069751648354647413760} a5$$

$$+ \frac{2735040090056403720280406115245423}{144397022497907316359168000} b + \frac{18535799610469548226575}{141488155787264} \Big) z^4 + \Big($$

$$- \frac{13835135235182931}{35063480320} a1 + \frac{8496747212200252409}{5952025784320} a2$$

$$- \frac{7441124964851304121877}{4820058698792960} a3 - \frac{6792404753733467749364777421}{846499130437155779584} a4$$

$$- \frac{8301666337496128563684734334625}{311511680000873326886912} a5$$

$$- \frac{23537712238933453532913090841160821}{4113431701766472768631603200} b - \frac{10741303760830330630125}{565952623149056} \Big) z^2$$

$$+ \frac{255916565120130269341}{205800953872384} - \frac{31594884337452726164830759584979579}{202733419587061872168271872000} b$$

$$- \frac{4197215370177153}{87658700800} a1 - \frac{860291453575570921}{25508681932800} a2$$

$$- \frac{1453908918147432136727}{43441285331558400} a3 - \frac{68952526819307765788730779}{549674760024127129600} a4$$

$$- \frac{234428182111687638956104744517}{1668812571433249965465600} a5 \Big) s^{10} + \Big(\frac{10443430644531899}{1638912000} a2$$

$$+ \frac{2595013084975368378811}{24904906752000} a3 + \frac{3366693621875931176457559}{8048954550912000} a4$$

$$+ \frac{348372409490301081915177889}{341080546787737600} a5 + \frac{2074910359073224902866491518997}{206976202804468869120000} b$$

$$+ \frac{1759608769009}{13516800} a1 + \frac{62720296114990087}{7272398848} \Big) z^{24} + \Big(- \frac{331766064880549}{393676800} a1$$

$$- \frac{345548493243488959}{17183992320} a2 - \frac{11042607418767293256539}{52225589458944} a3$$

$$- \frac{2105928721977284145807873223}{2672454134766556800} a4 - \frac{3714055485121172158118826469877}{2006264768051949520896} a5$$

$$- \frac{2128296133929234336598390524797429939}{129861505906466588588556288000} b - \frac{114316300672001155735}{3812555096064} \Big) z^{22}$$

$$+\Bigg(\Bigg(-\frac{8562333547957}{10240}\,a3-\frac{115661265619276829}{33094400}\,a4-\frac{1063821204492579939}{1217873920}\,a5$$

$$-\frac{528723819204886987853604 19}{504515475800064000}\,b\Bigg)z^{22}+\Bigg(\frac{619296585737324 11}{13414400}\,a3$$

$$+\frac{650690128344311631289}{34682931200}\,a4+\frac{14783754496774062780561}{319082967040}\,a5$$

$$+\frac{82440325539863440573930331}{151934545585766400}\,b+\frac{804288059253}{8192}\,a2+\frac{6333768466617375}{54525952}\Bigg)z^{20}$$

$$+\Bigg(\frac{1759608769009}{1351680}\,a1-\frac{167435267214124427}{327782400}\,a2$$

$$-\frac{274800192764831803061 09}{2490490675200}\,a3-\frac{1323063985696201068880629013}{30586027293465600}\,a4$$

$$-\frac{39392098037589748006399179581}{375188601466511360}\,a5$$

$$-\frac{1341146090877105402866283431489179 01}{1165689974194768670883840 00}\,b-\frac{302297594161084504 15}{58179190784}\Bigg)z^{18}+\Bigg($$

$$-\frac{2331848933132381}{419921920}\,a1+\frac{2585538462970108508 3}{22911989760}\,a2$$

$$+\frac{20795843517275218170727133}{1392682385571840}\,a3+\frac{1991696606077624734258567 6671}{3563272179688742 40}\,a4$$

$$+\frac{141547585731799452289392753689 8855}{107000787629437307781 12}\,a5$$

$$+\frac{3469250215432690661962501671592410353 71}{2770378792671287223222534144 00}\,b+\frac{1036891234856244962792 5}{10166813589504}\Bigg)$$

$$z^{16}+\Bigg(\frac{225391922662451671 23}{2147638169600}\,a1-\frac{1075992679433974858924 91}{78120338419200}\,a2$$

$$-\frac{2981394938943250753091698 2373}{237423332523632 6400}\,a3$$

$$-\frac{9137294377505856249320275007334 67}{20653764841195026833600}\,a4$$

$$-\frac{18385131746239133900164783149016424 87}{182414051077434476994355 20}\,a5$$

$$-\frac{5556819954920955467865192667977689908242 7}{821377161609751570566304235520 00}\,b$$

$$-\frac{476614649370941306638619 9}{3961668362043392}\Bigg)z^{14}+\Bigg(-\frac{2048042037834745 29}{17531740160}\,a1$$

$$+\frac{128790789380538471146 33}{12754340966400}\,a2+\frac{1014015255779166639435552217}{149766109569638400}\,a3$$

$$+\frac{1481703144948484669587719492799 29}{67440864787575597824 00}\,a4$$

$$+\frac{3185373488399832134833918238967 07}{676861042958940545433 60}\,a5$$

$$+\frac{25806762346880047406733474035051055 2159}{3504948927091704289986084864000}\,b$$

$$+\frac{112913849171386649997180 5}{1131905246298112}\Big) z^{12}+\Big($$

$$-\frac{19234047967938915113007501132566 9}{14976523076965063792640}\, a5$$

$$+\frac{696410983156313273546654981737913 59}{60646749449121072870850560 0}\, b+\frac{262544357853031839}{30680545280}\, a1$$

$$-\frac{2679592646980619344 61}{595202578432}\, a2-\frac{7708784550048595931093415 1}{33137903554201600}\, a3$$

$$-\frac{54622789872590196331645658217 43}{813941471574188249600}\, a4-\frac{709564580152986298344015}{1131905246298112}\Big) z^{10}+\Big($$

$$-\frac{2758702894057695}{637517824}\, a1+\frac{147864742528797944989}{1275434096640}\, a2$$

$$+\frac{110830766912358871526995 19}{22723133865738240}\, a3+\frac{190893888447738566132812184189}{1627882943148376499 20}\, a4$$

$$+\frac{124040665197993286752310562225963}{7188731076943230620467 2}\, a5$$

$$-\frac{342391098128195781081244592639611967}{5458207450420896558376550400}\, b+\frac{1659958714854996499721 75}{565952623149056}\Big) z^{8}$$

$$+\Big(\frac{2661401816565441}{1753174016}\, a1-\frac{225658595390441923}{15942926208}\, a2$$

$$-\frac{6396725186079087599879 3}{1136156693286912}\, a3-\frac{14900891576266087156766094761}{151160559006634960640}\, a4$$

$$-\frac{11840668642858490517299096821 51}{333762514286649993093 12}\, a5$$

$$+\frac{46523765359976770225428812976969847}{405466839174123744336543744 0}\, b-\frac{532325250940217877441 25}{565952623149056}\Big) z^{6}$$

$$+\Big(-\frac{6217647116499081}{17531740160}\, a1+\frac{118555308689160151}{5101736386560}\, a2$$

$$+\frac{235807830852370429296 77}{8688257066311680}\, a3+\frac{31329555338238886608750681 11}{120928447205307968512 0}\, a4$$

$$-\frac{386037070394316827573854559 03}{351328962406999992729 6}\, a5$$

$$-\frac{2180149111051745294983632933048571}{2027334195870618721682718720 0}\, b+\frac{4195065277235911232283 5}{2263810492596224}\Big) z^{4}$$

$$+\Big(\frac{6054168935244057}{122722181120}\, a1+\frac{318463156779010937 29}{232129005588480}\, a2$$

$$+\frac{233539170291857233089 89}{10339025908910899 20}\, a3-\frac{8934059739712185578822047 71}{2116247826092889448960 0}\, a4$$

$$+\frac{4985147161970471190637346369 47}{2336337600006549951651840}\, a5$$

$$-\frac{254679331437761611768788769077853585 9}{21996576025196213130257498112000}\, b-\frac{567906977663391015888 05}{29429536403750912}\Big) z^{2}$$

$$+\frac{181066778093663257165}{2340985850298368}-\frac{340400704248960136225189107397030 3}{5587536176216131129473761280 00}\, b$$

$$- \frac{10444761111506027}{3436221071360}\ a1 - \frac{2353461951676460777}{406225759779840}\ a2$$
$$- \frac{1609818826838247219748391}{246920265824577945600}\ a3 - \frac{200533842663939180489257}{24434817972223859200}\ a4$$
$$- \frac{16374727640226156629836673}{938297010133396193280}\ a5 \Big) s^{6} + \Big(\Big(- \frac{563986042081}{3072}\ a3$$
$$- \frac{7362914108305481}{9928320}\ a4 - \frac{221991186338482517}{121787392}\ a5$$
$$- \frac{116527907280575125745321}{5219125611724800}\ b \Big) z^{24} + \Big(\frac{404271775815}{45056}\ a2$$
$$+ \frac{17101712955786433}{14755840}\ a3 + \frac{178755157654614999943}{38151224320}\ a4$$
$$+ \frac{4055342235346664783907}{350991263744}\ a5 + \frac{161494763410330393892618377}{1101525455496806400}\ b$$
$$+ \frac{3183640234543125}{299892736} \Big) z^{22} + \Big(- \frac{106418199343408443862343}{33206542336000}\ a3$$
$$- \frac{2634713384667965403782284473}{203906848623104000}\ a4 - \frac{478092052051437027610544124581}{15007544058660454400}\ a5$$
$$- \frac{19813122135351555853465527744080941}{4857041559144869462016000 0}\ b + \frac{47509436763243}{18022400}\ a1$$
$$- \frac{117754503540975287}{2185216000}\ a2 - \frac{2920171327895770977}{116358381568} \Big) z^{20} + \Big($$
$$- \frac{1583736651734107}{114524160}\ a1 + \frac{37213439921289835211}{274943877120}\ a2$$
$$+ \frac{21280870411408550494823729}{4178047156715520}\ a3 + \frac{174621633749433407157035796319}{8551853231252981760}\ a4$$
$$+ \frac{94685730878940991861127082892643}{1888249193460658372608}\ a5$$
$$+ \frac{26080825870188298718799282231710691461}{41555681890069308348338012160}\ b - \frac{1337126818092572577875}{122001763074048} \Big) z^{18}$$
$$+ \Big(\frac{98480704496653127883}{3123837337600}\ a1 - \frac{92325532643665461883751}{499970165882880}\ a2$$
$$- \frac{38972245762483894436414621471}{7597546640756244480}\ a3$$
$$- \frac{1585847149048447150288822083614543}{77755349990381277491200}\ a4$$
$$- \frac{2899212259106069566900991716686676861}{58372496344779032638193664}\ a5$$
$$- \frac{10952276280014242427683260113839910516116 43}{1889167471702428612302499741696000}\ b$$
$$+ \frac{2590922889989891008934783}{31693346896347136} \Big) z^{16} + \Big(- \frac{50178037468194301 77}{122722181120}\ a1$$
$$+ \frac{109352238991610105833}{744003223040}\ a2 + \frac{2613191453447616182328452201}{768799362457477120}\ a3$$

$$+ \frac{17948610017103346873501179701 1}{13411535611165601840} a4$$

$$+ \frac{1116421055452625105046486377252041 1}{3474553353855894799892 48} a5$$

$$+ \frac{6918139485432991389142637419518897983}{2152161621898414914903736320 0} b - \frac{21355880345798615641005}{282976311574528} \Big) z^{14}$$

$$+ \Big(\frac{1628178519591140684 7}{490888724480} a1 - \frac{785201173121261876783}{11904051568640} a2$$

$$- \frac{1576820128300357321495563 31}{106041291373445120} a3 - \frac{75540709374993144043681981196 9}{1302306354518701199 36} a4$$

$$- \frac{652544854899342924618810863933393}{47924873846288204136448} a5$$

$$- \frac{579181522317632113479747128137309}{60646749449121072870850 56} b - \frac{20722649587080411411615}{2263810492596224} \Big) z^{12}$$

$$+ \Big(\frac{44030045659219750144165053345302 7}{1198121846157205103411 20} a5$$

$$+ \frac{70209489015862811846552603286437}{10986729972666861027328000} b - \frac{611069162915641599}{35063480320} a1$$

$$+ \frac{52038239748067515069}{4251446988800} a2 + \frac{15805509077637845581946823}{37871889776230400} a3$$

$$+ \frac{24020533476667070548748263277 1}{147989358468034227200} a4 + \frac{67099228110284013097965}{1131905246298112} \Big) z^{10}$$

$$+ \Big(\frac{83349565212019437}{14025392128} a1 + \frac{5772239983842275165}{2380810313728} a2$$

$$- \frac{1471938724482573066663415}{21208258274689024} a3 - \frac{46780941841968610595476824162 9}{1692998260874311559168} a4$$

$$- \frac{366485073721229700164457216840245}{62302336000174665377382 4} a5$$

$$+ \frac{4338528900818925249543033555370213}{94608929140628873678526873 6} b - \frac{9036370911409230161625}{205800953872384} \Big) z^{8} + \Big($$

$$- \frac{22500479061739431}{17531740160} a1 - \frac{917856543151181039}{510173638656} a2 + \frac{856731328033232036927}{157968310296576} a3$$

$$+ \frac{15209343186570803060946602741}{604642236026539842560} a4 + \frac{1583178341528635185848155757740 7}{333762514286649993093120} a5$$

$$- \frac{6167027149686493396151223604500853}{5068335489676546804206796800} b + \frac{17634182069496685342405}{1131905246298112} \Big) z^{6}$$

$$+ \Big(\frac{163364120284882059}{981777448960} a1 + \frac{1161585295025697780 97}{309505340784640} a2$$

$$+ \frac{805616482052547008762 29}{275707357570957312 0} a3 - \frac{145252659681657314770258204 53}{1692998260874311559168 0} a4$$

$$- \frac{141938399131550046082858090988 9}{12460467200034933075476 48} a5$$

$$+ \frac{2080612727847966021704927192508873 7}{366609600419936885504291635200} b - \frac{170150168438241766315815}{58859072807501824} \Big) z^{4}$$

$$+\left(-\frac{40353778365501861}{3436221071360}a1-\frac{35057243129922556093}{1083268692746240}a2\right.$$
$$-\frac{409447987272530828472229}{1496486459542896640}a3-\frac{14754395948038998358461}{1503681105983006720}a4$$
$$-\frac{1457747725790794160581849}{62553134008893079552}a5+\frac{53626598629861955514698791747567}{9312560293693551882456268800}b$$
$$\left.+\frac{53175214050704443661385}{206006754826256384}\right)z^{2}+\frac{7633728381222806895285329627}{11240313187221676228608000}b$$
$$+\frac{355929977455359}{981777448960}a1+\frac{2538440828870767327}{3214093923532800}a2+\frac{14654187660876563617}{12856375694131200}a3$$
$$+\frac{740349478602306049}{910659945000960}a4+\frac{458056140696181124299}{166104373968175104}a5$$
$$\left.-\frac{322023946741293608795}{40748588866732032}\right)s^{4}+\left(\frac{7420211216632651749}{1718110535680}a1\right.$$
$$+\frac{1629842435776855435787}{41664180490240}a2+\frac{31423512396090303563432217}{168702054457753600}a3$$
$$+\frac{6019565731416738206488428116509}{11395180602038635494400}a4$$
$$+\frac{80649623660578427748628638610043}{76244117482731233853440}a5$$
$$\left.+\frac{935830724272107716744985955661101}{159197717303942816285982720}b+\frac{121670546501442061180605}{1980834181021696}\right)z^{16}$$
$$+\left(\frac{753678487563403}{40333800}a4+\frac{1043636703136697}{21511360}a5+\frac{34130390213011377549137}{61487823613132800}b\right.$$
$$\left.+\frac{51271458371}{12480}a3\right)z^{28}+\left(-\frac{141775224922301}{4611200}a3-\frac{20438048656998993167}{154989348800}a4\right.$$
$$-\frac{474568069826297594283}{1425902008960}a5-\frac{138273218452781386546903}{38912584025702400}b-\frac{12766477131}{14080}a2$$
$$\left.-\frac{20107201481325}{18743296}\right)z^{26}+\left(\frac{463179630061524849634715891}{1618795449024768000}a3\right.$$
$$+\frac{608223716428128934203023062772653}{619612945235850805008000}a4$$
$$+\frac{27410595624269084955000808458539449}{12437321664370532522342400}a5$$
$$+\frac{30437530108838534562836346088004370165961}{1771094504721026824033593507840000}b+\frac{12960342159615284551}{5369095424000}a1$$
$$\left.+\frac{44036250860193796086889}{1171805076288000}a2+\frac{883381111890755751047683}{14856256357662720}\right)z^{20}+\Bigg($$
$$-\frac{246905048812968351}{61361090560}a1-\frac{3091453919334340672601}{66960290073600}a2$$
$$-\frac{427736366929396759527524423}{1572544150481203200}a3-\frac{6031571475723033199479289698073}{7081290802695437771520}a4$$
$$-\frac{257800446212884568989447300476001}{142140819021377514541056}a5$$

$$- \frac{557112589287020050873009841285213798 99}{4600245466807861880606736384000} b - \frac{7902982148882249990875}{106116116840448} \Big) z^{18}$$

$$+ \Big(- \frac{2391541439177525 7}{7670136320} a1 - \frac{6505974895613980489 7}{2790012086400} a2$$

$$- \frac{20925956222583359993467 9}{2259402515059200} a3 - \frac{12025030645465462858491537913}{5087134197338676560 0} a4$$

$$- \frac{4505241789145353891300426747 73}{102112657342943616768 0} a5$$

$$- \frac{24578670731655311928986079441705 7}{12183498773260929817804800 0} b - \frac{1188493907120846410625}{35372038946816} \Big) z^{14}$$

$$+ \Big(\frac{673063825683231 3}{4382935040} a1 + \frac{21853610231856487379}{2232009669120} a2$$

$$+ \frac{600816726626600006280 13}{1807522012047360} a3 + \frac{574779520596079263465294972 1}{75580279503317480320} a4$$

$$+ \frac{1533304961593038733415801042437 7}{116816880000327497582592} a5$$

$$+ \frac{2886770000226577881619627808249 83}{5543491941833723067101184 00} b + \frac{840097535893991938625}{70744077893632} \Big) z^{12} + \Big($$

$$- \frac{1146033504211220474906253705023 3}{417203142858312491366400} a5$$

$$- \frac{3936404807676371456822870136583 3}{346468246364607691693824000} b - \frac{11226073808319507}{21914675200} a1$$

$$- \frac{4546739531426390521}{1594292620800} a2 - \frac{833194359824820269965 3}{987301939353600} a3$$

$$- \frac{653722389748208271750763606 9}{37790139751658740160 0} a4 - \frac{1354269162555188513 9}{5441852145664} \Big) z^{10}$$

$$+ \Big(\frac{2761747191512894 1}{245444362240} a1 + \frac{2586381990027871825 7}{46425801117696} a2$$

$$+ \frac{548693340138097552981 75}{37596457850585088} a3 + \frac{5692577771021000688385774300 9}{211624782609288944896 00} a4$$

$$+ \frac{1670993489359452442692948441349}{4247886545466454457548 80} a5$$

$$+ \frac{7546282941865245487974032916899039}{3436965003936908301602734080 00} b + \frac{823538882090394068435}{3678692050468864} \Big) z^{8} + \Big($$

$$- \frac{330565746618802 7}{214763816960} a1 - \frac{1395741994536421540 3}{203112879889920} a2$$

$$- \frac{24878116101295197502231 67}{1543251661403612160 0} a3 - \frac{1644846658071270176887713}{610870449305596480 0} a4$$

$$- \frac{2148695227554314251063324 1}{5864356313333726208 0} a5 - \frac{5477729419895491891321636477540 7}{17461050550675409779605504000} b$$

$$+ \frac{10153081022217006858 5}{643771108832051 2} \Big) z^{6} + \Big(\frac{1817035894375}{1534027264} a1 + \frac{1208200742116891 37}{25110108777600} a2$$

$$+ \frac{1021279748898570437}{100440435110400} a3 + \frac{553480296551207647}{35572654101600} a4$$

$$+ \frac{130473901847494775011}{6488452108131840}\, a5 + \frac{33513615377539410098246587}{140503914840270952857600}\, b$$

$$- \frac{6385790771421568165}{1273393402085376} \Big) z^{4} + \Big(\Big(- \frac{51271458371}{24960}\, a3 + \frac{173870767328549}{80667600}\, a4$$

$$+ \frac{18045255382957793}{989522560}\, a5 + \frac{33729710164459108299391}{1229756472262656000}\, b \Big) z^{26} + \Big($$

$$- \frac{21277461885}{5632}\, a2 + \frac{34310728471377}{1844480}\, a3 + \frac{219273203003605491}{4768903040}\, a4$$

$$+ \frac{3712612469779331859}{43873907968}\, a5 + \frac{1259346082852911818771957}{275381363874201600}\, b$$

$$- \frac{167560012344375}{37486592} \Big) z^{24} + \Big(\frac{1759608769009}{1802240}\, a1 + \frac{5227705674364187}{218521600}\, a2$$

$$- \frac{215101954032434324037}{3320654233600}\, a3 - \frac{6226400808299589407707627}{20390684862310400}\, a4$$

$$- \frac{11409079564260014322306 6909}{136432218715095040}\, a5 - \frac{1077600517483954321631426574557}{40475346326207245516800}\, b$$

$$+ \frac{277319518391677335}{7272398848} \Big) z^{22} + \Big(\frac{1221246528988488381392389}{10445117891788800}\, a3$$

$$+ \frac{3422141331557695824363371293}{4275926615626490880}\, a4 + \frac{193291414725583093892209393216751}{80250590722077980835840}\, a5$$

$$+ \frac{17213791992236853032051986228549887307}{259723011812933177177112576000}\, b - \frac{18053714665511629}{3149414400}\, a1$$

$$- \frac{46916452467112577219}{687359692800}\, a2 - \frac{153296154216552231605}{1173093875712} \Big) z^{20}$$

$$+ \Big(\frac{23054567800543044337}{1561918668800}\, a1 + \frac{433844698117648083479069}{3749776244121600}\, a2$$

$$- \frac{6657908678497420392160803619}{56981599805671833600}\, a3 - \frac{136517074490925704203674725598959}{116633024985571916236800}\, a4$$

$$- \frac{1621410519281066087066424279979158281}{437793722585842744786452480}\, a5$$

$$- \frac{2634561756129095676248756008070663862941}{28337512075536429184537496125440}\, b$$

$$+ \frac{1436638951594820426309587}{5942502543065088} \Big) z^{18} + \Big(- \frac{1342340089318399509}{61361090560}\, a1$$

$$- \frac{2882323808588736425029}{22320096691200}\, a2 + \frac{321126283600731725197324123}{5765995218431078400}\, a3$$

$$+ \frac{12557548669865438307168393611159}{11802151337825729619200}\, a4$$

$$+ \frac{9128935763967564098441820825110689}{2605915015391921099919360}\, a5$$

$$+ \frac{7702646615199543706037430897632875667}{95838447225163789179307008000}\, b - \frac{6885041788588264594385}{25725119234048} \Big) z^{16}$$

$$+ \Big(\frac{35368896819323726607}{1718110535680}\, a1 + \frac{4135088262375366060577}{41664180490240}\, a2$$

$$+\frac{464315735827225013372353}{74228903961411584}\,a3-\frac{1420507480178335812718447091797}{2279036120407727098880}\,a4$$
$$-\frac{18910352076189619331957353294043}{8828266234842563919872}\,a5-\frac{391325378182330676112089807299}{8969896174438968688640}\,b$$
$$+\frac{180226031601213989277195}{990417090510848}\Big)z^{14}+\Big(-\frac{224336115269635863}{17531740160}\,a1$$
$$-\frac{13597584717198532679}{255086819328}\,a2-\frac{290829993990340219715479}{11361566932869120}\,a3$$
$$+\frac{38037552820189394325664809139}{162788294314837649920}\,a4+\frac{30640484416723633218013466421101}{35943655384716153102336}\,a5$$
$$+\frac{4858001408859974975220000253340843}{341137965651306034898534400}\,b-\frac{10267642002992761954595}{141488155787264}\Big)z^{12}+\Big($$
$$-\frac{66839976185748390627086863668599}{311511680000873326886912}\,a5$$
$$-\frac{6849075545068529133352648634480639}{2956529035644652302453964800}\,b+\frac{185716973688205563}{35063480320}\,a1$$
$$+\frac{23670803047535412023}{1190405156864}\,a2+\frac{10164023984958689806819}{623772302196736}\,a3$$
$$-\frac{223775509828201738551252632163}{4232495652185778897920}\,a4+\frac{1857580639364640354375}{141488155787264}\Big)z^{10}+\Big($$
$$-\frac{12591031711272939}{8765870080}\,a1-\frac{3201917102695237241}{637717048320}\,a2$$
$$-\frac{23545505933863796735023}{4344128533155840}\,a3+\frac{897643625908496744015475547}{151160559006634960640}\,a4$$
$$+\frac{5258765111304226034375577501311}{166881257143324996546560}\,a5$$
$$-\frac{30251305636463692014601141672043}{8869587106933956907361894400}\,b+\frac{46177702380368360095}{35372038946816}\Big)z^{8}$$
$$+\Big(\frac{120029073288312453}{490888724480}\,a1+\frac{378996828958865917421}{464258011176960}\,a2$$
$$+\frac{4252366039796160388475777}{4135610363564359680}\,a3-\frac{239270846014681931150693959}{42324956521857788979200}\,a4$$
$$-\frac{20238307804032656064764773431809}{9345350400026199806607360}\,a5$$
$$+\frac{1141805363460074432513316353388289}{20217441199628872362369024000}\,b-\frac{4195088392353933912535}{3678692050468864}\Big)z^{6}+\Big($$
$$-\frac{41399768356502133}{1718110535680}\,a1-\frac{436503209356482087}{5583859240960}\,a2$$
$$-\frac{1800724697018415979155}{16970465005125632}\,a3-\frac{1301574694737945787095}{20152427193586688}\,a4$$
$$+\frac{2206818083724942734727}{322438835097387008}\,a5-\frac{41594845823555462786385693053}{8000481351970405397299200}\,b$$
$$+\frac{575536918618876717735}{265472622198784}\Big)z^{4}+\Big(\frac{582445339737981}{490888724480}\,a1$$

$$+ \frac{5928771876389573221}{1607046961766400}\, a2 + \frac{32895644273849226211}{6428187847065600}\, a3$$

$$+ \frac{10730312023171386791}{2276649862502400}\, a4 + \frac{2009794514958630452453}{415260934920437760}\, a5$$

$$- \frac{1184079359532786089563537}{1405039148402709528576000}\, b - \frac{41405739781820237485}{2546786804170752} \Big)\, z^2$$

$$- \frac{43257371682049125783197}{896021790503583744000}\, b - \frac{38544880943}{1525311200}\, a1 - \frac{5482056727237}{134227385600}\, a2$$

$$- \frac{93139958608237}{872478006400}\, a3 - \frac{16524174354517}{436239003200}\, a4 - \frac{592133172045907}{2966425221760}\, a5$$

$$+ \frac{4591820599943715}{20420970938368} \Big)\, s^2 + \Big(-\frac{8398429821247}{195239833600}\, a1 - \frac{82713272877511}{536909542400}\, a2$$

$$- \frac{8230701001458713}{27919296204800}\, a3 - \frac{724440186163339}{1744956012800}\, a4 - \frac{49721367055609061}{94925607096320}\, a5$$

$$- \frac{603295891141450908491}{68924753115660288000}\, b + \frac{253068902485965}{1276310683648} \Big)\, z^2 + \Big(\Big(\frac{3845359377825}{1024}\, a3$$

$$+ \frac{50960591037903513}{3309440}\, a4 + \frac{4643939746740574143}{121787392}\, a5$$

$$+ \frac{972816291082962327462841}{2102147815833600}\, b \Big)\, z^{14} + \Big(\frac{1008551693349}{4096}\, a2$$

$$- \frac{18494657491662069}{1341440}\, a3 - \frac{203502297344441259003}{3468293120}\, a4$$

$$- \frac{4722201052899850330047}{31908296704}\, a5 - \frac{373692028160985798973531929}{183587575916134400}\, b$$

$$+ \frac{7942344585123375}{27262976} \Big)\, z^{12} + \Big(\frac{13766226526552804096356882099}{64687689908019200}\, a5$$

$$+ \frac{12950583827639767824427576512547}{4229398780167946240000}\, b - \frac{100297699833513}{2252800}\, a1$$

$$- \frac{31073060857803027}{68288000}\, a2 + \frac{2769312281853034935297}{143131648000}\, a3$$

$$+ \frac{18404154645528878281852773}{219727207568000}\, a4 - \frac{17684930552672851461}{29089595392} \Big)\, z^{10}$$

$$+ \Big(\frac{22054448750585953}{209960960}\, a1 + \frac{163068874078423219}{1041454080}\, a2$$

$$- \frac{8946251294492795183325121}{696341192785920}\, a3 - \frac{9836793975490070151522724477}{1781636089844371 20}\, a4$$

$$- \frac{746310463814056863232811636223059}{5350039381471865389056}\, a5$$

$$- \frac{50532893305724922995425277978004037}{27375284512562126711685120}\, b + \frac{990018961307311987625}{2541703397376} \Big)\, z^8 + \Big($$

$$- \frac{95529713384728902201}{1073819084800}\, a1 + \frac{4760537574885298670227}{31248135367680}\, a2$$

$$+ \frac{921899860503772131254767793}{237423332523632640}\, a3 + \frac{1293976805382239321880477535987589}{82615059364780107334400}\, a4$$

$$+ \frac{69476904326050792944021471467425471}{1824140510774344769943552}\, a5$$

$$+ \frac{27618630611930770607267758603367352493 7}{12628124810845111044802805760 00}\, b - \frac{2070669165306475108392 89}{3961668362043392}\Bigg)$$

$$z^{6} + \Bigg(\frac{3823893607938009723}{122722181120}\, a1 - \frac{20748367196387340191 39}{17856077352960}\, a2$$

$$- \frac{74683204224971504343412654 1}{2306398087372431360}\, a3 - \frac{62705813409401486746461057415 3}{858338279114598517760}\, a4$$

$$- \frac{94245603854376188642504977431878 3}{1042366006156768439967744}\, a5$$

$$+ \frac{22343398256852165183560319009995728501 7}{12267321244820965014951297024 00}\, b - \frac{1615876009540751794844 5}{1131905246298112}\Bigg)$$

$$z^{4} + \Bigg(- \frac{2784276783136338789}{859055267840}\, a1 + \frac{193760122691607977 93}{946913192960}\, a2$$

$$- \frac{429195343600397786499159}{10916015288442880}\, a3 - \frac{162109045944454003644600939647}{569759030101931774720}\, a4$$

$$- \frac{74412467619894502085686855346437}{83868529231004357238784}\, a5$$

$$- \frac{75387419353475873488527594310215427}{15919771730394281628598272 00}\, b - \frac{366298042828510438228 65}{7923336724086784}\Bigg) z^{2}$$

$$+ \frac{1281623112629088174 95}{51450238468096} - \frac{857991787491139359999441307595579 3}{68227593130261206979706880 00}\, b$$

$$- \frac{5335497958710414 3}{245444362240}\, a1 + \frac{130063299563978205 07}{44640193382400}\, a2$$

$$- \frac{193979612940058265475 07}{795309685300838400}\, a3 - \frac{116428162859746831656906942 7}{813941471574188249600}\, a4$$

$$- \frac{132410815946389927436229355432 9}{35943655384716153102336 0}\, a5\Bigg) s^{14} + \Bigg(\Bigg(\frac{41068438155171}{10240}\, a3$$

$$+ \frac{552217484891782467}{33094400}\, a4 + \frac{506796151697376527 97}{1217873920}\, a5$$

$$+ \frac{561266800275271864707111 41}{112114550177792000}\, b\Bigg) z^{12} + \Bigg(- \frac{895136258983885385555 7}{79770741760}\, a5$$

$$- \frac{41843645273668684601934021 1}{293740121465815040}\, b - \frac{12766477131}{512}\, a2 - \frac{3609390290576864 7}{3353600}\, a3$$

$$- \frac{486615787578256534 71}{1083841600}\, a4 - \frac{100536007406625}{3407872}\Bigg) z^{10} + \Bigg(- \frac{47509436763243}{1802240}\, a1$$

$$+ \frac{5247872504999222 7}{218521600}\, a2 + \frac{335021096930841472091 43}{3320654233600}\, a3$$

$$+ \frac{836194236483548585507844213}{20390684862310400}\, a4 + \frac{151841338732137069584839002261}{15007544058660454 40}\, a5$$

$$+ \frac{120867827261105204013416856822239}{1023882278607614115840 00}\, b + \frac{1296014748841141843 5}{58179190784}\Bigg) z^{8}$$

$$+ \Bigg(\frac{4789585839125581}{104980480}\, a1 - \frac{8832608033773116577}{22911989760}\, a2$$

$$
- \frac{1239291170954804450329813}{348170596392960} a3 - \frac{9082174770548659395139944173}{712654435937748480} a4
$$
$$
- \frac{78093938815151543123832382065215}{2675019690735932694528} a5
$$
$$
- \frac{46244191939842961506446578578151771}{740742992692857546316185600} b - \frac{3490445276659795329575}{10166813589504} \Bigg) z^6 + \Bigg(
$$
$$
- \frac{209774955464535584253}{8590552678400} a1 + \frac{135863306176216999847387}{624962707353600} a2
$$
$$
+ \frac{987073164933624212624942543}{9496933300945305600} a3 - \frac{281907583007773748138262720103717}{330460237459120429337600} a4
$$
$$
- \frac{276201258577546416270589962116986883}{72965620430973790797742080} a5
$$
$$
- \frac{194274432767687080465129022215954606715269}{686969989709974040837272633344000} b
$$
$$
+ \frac{134496115872212905628287}{720303338553344} \Bigg) z^4 + \Bigg(\frac{409913895528304947}{122722181120} a1
$$
$$
- \frac{577145061445211200919}{14880064460800} a2 + \frac{467069344383821676373286393}{3843996812287385600} a3
$$
$$
+ \frac{1042052088959568626207353582587}{1388488392685379955200} a4
$$
$$
+ \frac{3815721136251413746410145742982443}{1737276676927947399946240} a5
$$
$$
+ \frac{1282086706871958553428920898903042244027}{16356428326427953353268396032000} b - \frac{17173212403335949720635}{565952623149056} \Bigg)
$$
$$
z^2 - \frac{2010620246037589222935}{1440606677106688} + \frac{1445131782296489446068251253260537}{599332582791314131900170240} b
$$
$$
+ \frac{1194605638066502103}{3436221071360} a1 - \frac{13715726320680764983}{16665672196096} a2
$$
$$
+ \frac{1654517347852688925255873}{3711445198070579200} a3 + \frac{126965363756372702107942721171}{22790361204077270988800} a4
$$
$$
+ \frac{28277010300117594584178864492947}{1677370584620087144775680} a5 \Bigg) s^{16} + \Bigg(\Bigg(\frac{697369778412814217}{30446848} a5
$$
$$
+ \frac{414682565781807959653694839}{1513546427400192000} b + \frac{8357247714473}{3840} a3
$$
$$
+ \frac{4538326290682147}{496416} a4 \Bigg) z^{10} + \Bigg(- \frac{472359653847}{4096} a2 - \frac{4971052734348011}{1341440} a3
$$
$$
- \frac{10367474052096421843}{693658624} a4 - \frac{1173292149893670670785}{31908296704} a5
$$
$$
- \frac{1800036887113305568963555163}{4406101821987225600} b - \frac{3719832274045125}{27262976} \Bigg) z^8 + \Bigg(
$$
$$
- \frac{51028654301261}{5406720} a1 + \frac{182622141640283849}{655564800} a2 + \frac{13961644363359583370641}{9961962700800} a3
$$
$$
+ \frac{262121589666906743150816551}{61172054586931200} a4 + \frac{13313263474734301278211270049}{1500754405866045440} a5
$$

$$- \frac{1147125096598241886475108277785577}{18801451196689817272320000} b + \frac{4386637953310117085}{14544797696} \Big) z^6$$

$$+ \Big(\frac{1266516603641341}{125976576} a1 - \frac{5648307294845810695}{27494387712} a2$$

$$+ \frac{15987692965441414604305}{37982246879232} a3 + \frac{2566457306673272695135606501}{855185323125298176} a4$$

$$+ \frac{13139925089662743623132757790015}{1459101649492326924288} a5$$

$$+ \frac{23824285994679856852744825802321799913}{83111363780138616696676024320} b - \frac{1634843771583791318375}{7625110192128} \Big) z^4$$

$$+ \Big(- \frac{31197912908297140993}{17181105356800} a1 + \frac{167524125506216208112337}{3749776244121600} a2$$

$$- \frac{13178927877233134947485165287}{56981599805671833600} a3 - \frac{99228420007495526904926975041651}{79310456990188903041024} a4$$

$$- \frac{1523784260798985828639799928307716693}{437793722585842744786452480} a5$$

$$- \frac{205616158494783126479339984786969284968283}{2267000966042914334762999690035200} b$$

$$+ \frac{569097188067832120725629}{11885005086130176} \Big) z^2 + \frac{167912079038395825505}{308701430808576}$$

$$- \frac{513055487275484021233816272490630566079}{147207854937851580179415564288000} b - \frac{51725229888064707}{122722181120} a1$$

$$+ \frac{399899643783692363329}{267841160294400} a2 - \frac{64838203904163540460843229}{34595971310586470400} a3$$

$$- \frac{127999951403256376214725010983}{8330930356112279731200} a4$$

$$- \frac{733669500475674508672766381754143}{15635490092351526599516160} a5 \Big) s^{18} + \Big(\Big(\frac{13792022301799}{30720} a3$$

$$+ \frac{191145147468406007}{99283200} a4 + \frac{5931369037867971539}{1217873920} a5$$

$$+ \frac{34849131807727377823917607}{605418570960076800} b \Big) z^8 + \Big(- \frac{1459633885311}{20480} a2$$

$$+ \frac{225730480655379}{6707200} a3 + \frac{8815472484273651441}{17341465600} a4 + \frac{269170413443984720409}{159541483520} a5$$

$$+ \frac{131407123020855589138263403}{2203050910993612800} b - \frac{2298923369364825}{27262976} \Big) z^6 + \Big($$

$$- \frac{29913349073153}{18022400} a1 + \frac{217032544846035317}{2185216000} a2 - \frac{21706012423587608130987}{33206542336000} a3$$

$$- \frac{678475809540981580876737157}{203906848623104000} a4 - \frac{135314111362276657570202854929}{15007544058660454400} a5$$

$$- \frac{11895975684639033871534187621519057}{64760554121931592826880000} b + \frac{12923076914208285027}{116358381568} \Big) z^4$$

$$+ \Big(\frac{2574916790319211}{6298828800} a1 - \frac{8383542794823328997}{274943877120} a2$$

$$+\frac{1136608036994727686129113}{4178047156715520}\,a3+\frac{56944857431645996797582975147}{42759266156264908800}\,a4$$

$$+\frac{114337896146311670185583305886603}{32100236288831192334336}\,a5$$

$$+\frac{149714268946968116043203234968869110591}{2077784094503465417416900608000}\,b-\frac{4225057514443574878195}{122001763074048}\Bigg)\,z^{2}$$

$$+\frac{418776132929774402346044089641717511165309}{113350048302145716738149998450176 0000}\,b+\frac{58733250714850819757}{171811053568000}\,a1$$

$$-\frac{69670814165886246844717}{37497762441216000}\,a2+\frac{2400945305159013318655476347}{569815998056718336000}\,a3$$

$$+\frac{554978827729332060925494648390431}{19827614247547225760256000}\,a4$$

$$+\frac{362278783105688521505926485777550033}{4377937225858427447864524800}\,a5-\frac{40150898547438889744829}{43218200313200640}\Bigg)\,s^{20}$$

$$+\Bigg(\Bigg(-\frac{815695350964025880217247}{52191256117248000}\,b-\frac{3947902294567}{30720}\,a3$$

$$-\frac{51540398758138367}{99283200}\,a4-\frac{1553938304369377619}{1217873920}\,a5\Bigg)\,z^{6}+\Bigg($$

$$-\frac{13840457019645375}{599785472}-\frac{1757518351701}{90112}\,a2+\frac{2306589955347349}{5902336}\,a3$$

$$+\frac{133010444333221591451}{76302448640}\,a4+\frac{3147654850802780302299}{701982527488}\,a5$$

$$+\frac{298023870174310888646400229}{4406101821987225600}\,b\Bigg)\,z^{4}+\Bigg(\frac{57649337769444645}{4475322368}$$

$$+\frac{1228594170574589}{109260800}\,a2-\frac{20032170125442781579}{103770444800}\,a3$$

$$-\frac{8956047044864024256542349}{10195342431155200}\,a4-\frac{106922906565941753605050523}{46898575183313920}\,a5$$

$$-\frac{1456455049596172088732034843841001}{38856332473158955696128000}\,b\Bigg)\,z^{2}$$

$$-\frac{15030084387914213661803157234103}{160501181444155961671680}\,a5-\frac{1010218580161843}{6298828800}\,a1$$

$$+\frac{516609608965035793}{343679846400}\,a2-\frac{1207947241131099413021 57}{20890235783577600}\,a3$$

$$-\frac{35363085017711273585610457}{1068981653906622720}\,a4$$

$$-\frac{11501070949597530404801719471319210893}{4155568189006930834833801216000}\,b+\frac{3585361845519523895}{2772767342592}\Bigg)\,s^{22}$$

$$+\Bigg(\frac{358900208597}{798720}\,a3+\frac{962394957099569}{516272640}\,a4+\frac{29400211885491461}{6332944384}\,a5$$

$$+\frac{4403221017527019973088419}{78704414224809984000}\,b\Bigg)\,s^{28}+\Bigg(\Bigg(-\frac{2512301460179}{199680}\,a3$$

$$-\frac{33915710812207903}{645340800}\,a4-\frac{1039519640880976591}{7916180480}\,a5$$

$$- \frac{62020127857672852507028627}{39352207112404992000} b \Big) z^{2} - \frac{1212061155289930657527}{45628864286720} a5$$

$$+ \frac{100536007406625}{599785472} + \frac{12766477131}{90112} a2 - \frac{332425768875889}{147558400} a3$$

$$- \frac{50823186916448810173}{4959659161600} a4 - \frac{2058677052986627647337843}{4980810755289907200} b \Big) s^{26} + \Big(\Big($$

$$- \frac{358900208597}{4096} a3 - \frac{4403221017527019973088419}{403612380640051200} b - \frac{962394957099569}{2647552} a4$$

$$- \frac{441003178282371915}{487149568} a5 \Big) z^{4} + \Big(- \frac{301608022219875}{149946368} - \frac{38299431393}{22528} a2$$

$$+ \frac{1117205645710103}{14755840} a3 + \frac{6240877017739980577}{19075612160} a4$$

$$+ \frac{291443073492367948821}{350991263744} a5 + \frac{101332155511473012514490117}{8812203643974451200} b \Big) z^{2}$$

$$+ \frac{992255980559827236685279099}{15007544058660454400} a5 - \frac{89505382721110517}{116358381568} + \frac{1759608769009}{54067200} a1$$

$$- \frac{417755788686971}{595968000} a2 + \frac{481186830038172627691}{99619627008000} a3$$

$$+ \frac{1358572314466580921163491}{55610958715392000} a4 + \frac{32241557512834188399234400025099597}{2331379948389537341767680000} b \Big)$$

$$s^{24} + \Big(\Big(\frac{24046313975999}{20480} a3 + \frac{296430931491380959}{66188800} a4$$

$$+ \frac{26015105382290365089}{2435747840} a5 + \frac{274013925885814256704641457}{2018061903200256000} b \Big) z^{16}$$

$$+ \Big(\frac{1034084647611}{2048} a2 - \frac{4549585346212677}{670720} a3 - \frac{52631701606151342637}{1734146560} a4$$

$$- \frac{1248392006333960936463}{15954148352} a5 - \frac{947423393352792687692685389}{734350303664537600} b$$

$$+ \frac{8143416599936625}{13631488} \Big) z^{14} + \Big(- \frac{86220829681441}{1802240} a1 - \frac{66589533127059607}{43704320} a2$$

$$+ \frac{9490677747252611393617}{664130846720} a3 + \frac{55744999105382596606837835}{815627394492416} a4$$

$$+ \frac{2858478638139132960527215857}{15797414798589952} a5$$

$$+ \frac{125733481724407948041707465341840229}{38856332473158955696128000} b - \frac{105892471632172627185}{58179190784} \Big) z^{12} + \Big($$

$$- \frac{5185527050642869693286814930105221}{26750196907359326945280} a5$$

$$- \frac{220662141144515754320329644383241693541}{62963154378892891436875776000} b + \frac{149474375530650079}{1049804800} a1$$

$$+ \frac{396676957800490511039}{229119897600} a2 - \frac{51219631836790503423743959}{3481705963929600} a3$$

$$- \frac{206704629869785894300530885}{285061774375099392} a4 + \frac{44725211429534022140285}{20333627179008} \Big) z^{10} + \Big($$

$$- \frac{40085047748349591237 9}{2454443622400} a1 - \frac{5223921007452432422531 7}{59520257843200} a2$$

$$+ \frac{65107739011818436947912929 7}{822245307441152 00} a3 + \frac{37088957184146974081476449565772 77}{944172107026058369536 00} a4$$

$$+ \frac{66257319631144848925105069107567603}{631736973428344509071360} a5$$

$$+ \frac{37587450111798254808742917972840750597 61}{21808571101903937804357861376 00} b$$

$$- \frac{31167109697701840198432 29}{2263810492596224} \Big) z^8 + \Big(\frac{1563535267168013451}{17531740160} a1$$

$$+ \frac{164500368386322150321}{1062861747200} a2 - \frac{1148545138624638802934460879}{549142401755340800} a3$$

$$- \frac{17071129716785133666301980874143}{168602161968938994560 0} a4$$

$$- \frac{65860496634996778970785443099280 77}{248182382418278199992320} a5$$

$$- \frac{95855003470117815894932078068203396661}{38943876967685603222067609 6000} b + \frac{718127288761005904387 95}{141488155787264} \Big) z^6$$

$$+ \Big(- \frac{110098483944861959 43}{490888724480} a1 + \frac{217982434209140880847}{11904051568640} a2$$

$$+ \frac{20033951147088665061563731}{1060412913734451 20} a3 + \frac{50880775544439505593849718149 3}{651153177259350599680} a4$$

$$+ \frac{45713493972355283047079273494 27}{2522361781383589691392} a5$$

$$- \frac{275607668761420478614259498806450639}{3638804966947264372251033600} b - \frac{28825015095293811931906 5}{2263810492596224} \Big) z^4$$

$$+ \Big(\frac{256888896979533}{150487040} a1 - \frac{71814517170593677}{10947932160} a2 + \frac{802879212407252077469}{97524179681280} a3$$

$$+ \frac{12929506212873003629547667}{199617773531376640} a4 + \frac{954029024701162302663020599 1}{4407560439572796211 2} a5$$

$$+ \frac{66742769264959126036569268092919}{3346540435573817632358400} b + \frac{114266117246856309805}{4857962430464} \Big) z^2$$

$$- \frac{475757309695863816625}{205800953872384} + \frac{16829169048983087102121325803184181}{3339138675551607306300948480 0} b$$

$$+ \frac{7914157647622761}{70126960640} a1 - \frac{211828297631221289}{7142430941184} a2 + \frac{809539638991224540379}{6362477482406707 2} a3$$

$$+ \frac{30485038881753376848041920 51}{846499130437155779584 0} a4 + \frac{101102540533419760084972417307 3}{1869070080005239961321472} a5 \Big)$$

$$s^{12} + \frac{126248254059}{5691146240} + \frac{27754955931}{6224691200} a5 + \frac{6285901997}{3056768000} a4 + \frac{3019690587}{2394112000} a1$$

$$+ \frac{40247363}{85504000} a2 + \frac{78941451441}{26335232000} a3 + \frac{341948006306762087}{130802281758720000} b \Big)$$

$$\cos(2 \arctan(s \sin(\phi), s \cos(\phi))) + \frac{109360543639347}{218890240} \sin(2 \arctan(s \sin(\phi),$$

$$s \cos(\phi))) \Big(s^{20} + \Big(10 z^2 - \frac{350}{69} \Big) s^{18} + \Big(45 z^4 - \frac{1050}{23} z^2 + \frac{475}{43} \Big) s^{16} + \Big(120 z^6$$

$$- \frac{4200}{23} z^4 + \frac{3800}{43} z^2 - \frac{23800}{1763} \Big) s^{14} + \Big(210 z^8 - \frac{9800}{23} z^6 + \frac{13300}{43} z^4 - \frac{166600}{1763} z^2$$

$$+ \frac{232750}{22919} \Big) s^{12} + \Big(252 z^{10} - \frac{14700}{23} z^8 + \frac{26600}{43} z^6 - \frac{499800}{1763} z^4 + \frac{1396500}{22919} z^2$$

$$- \frac{316540}{65231} \Big) s^{10} + \Big(210 z^{12} - \frac{14700}{23} z^{10} + \frac{33250}{43} z^8 - \frac{833000}{1763} z^6 + \frac{3491250}{22919} z^4$$

$$- \frac{1582700}{65231} z^2 + \frac{1243550}{848003} \Big) s^8 + \Big(120 z^{14} - \frac{9800}{23} z^{12} + \frac{26600}{43} z^{10} - \frac{833000}{1763} z^8$$

$$+ \frac{4655000}{22919} z^6 - \frac{3165400}{65231} z^4 + \frac{4974200}{848003} z^2 - \frac{193800}{717541} \Big) s^6 + \Big(45 z^{16} - \frac{4200}{23} z^{14}$$

$$+ \frac{13300}{43} z^{12} - \frac{499800}{1763} z^{10} + \frac{3491250}{22919} z^8 - \frac{3165400}{65231} z^6 + \frac{7461300}{848003} z^4 - \frac{581400}{717541} z^2$$

$$+ \frac{56525}{2022161} \Big) s^4 + \Big(10 z^{18} - \frac{1050}{23} z^{16} + \frac{3800}{43} z^{14} - \frac{166600}{1763} z^{12} + \frac{1396500}{22919} z^{10}$$

$$- \frac{1582700}{65231} z^8 + \frac{4974200}{848003} z^6 - \frac{581400}{717541} z^4 + \frac{113050}{2022161} z^2 - \frac{109445270587687250}{109360543639347} \Big)$$

$$s^2 - \frac{109445270587687250}{109360543639347} z^2 + \frac{56525}{2022161} z^4 - \frac{193800}{717541} z^6 + \frac{1243550}{848003} z^8$$

$$- \frac{316540}{65231} z^{10} + \frac{232750}{22919} z^{12} - \frac{23800}{1763} z^{14} + \frac{475}{43} z^{16} - \frac{350}{69} z^{18}$$

$$+ \frac{4377804868292675}{5207644935207} + z^{20} \Big) \Big) \sqrt{5} \sqrt{13} \, \bar{se}_{\phi}$$

$$+ \frac{3}{5200000000} \sqrt{2} \Big(\Big(\Big(\Big(\frac{51271458371}{768} a3 + \frac{105426839900447}{496416} a4$$

$$+ \frac{1373363644670441 5}{30446848} a5 + \frac{2497058309245268499 57221}{37838660685004800} b \Big) z^{24} + \Big($$

$$- \frac{106387309425}{5632} a2 - \frac{4598642556507089}{10697984} a3 - \frac{3533824224651278 3825}{27659637632} a4$$

$$- \frac{3316984639554669582975}{1272343331072} a5 - \frac{4052751173957368289 21741}{12478218050549760} b$$

$$- \frac{837800061721875}{37486592} \Big) z^{22} + \Big(\frac{2222301527357629542 73001}{179315328614400} a3$$

$$+ \frac{12504585410755980078578874 37}{367032327521587200} a4 + \frac{5367088391162054524261034 55467}{81040737916766453760} a5$$

$$+ \frac{531862027427412124542339508262831}{8196257631056967217152000} b + \frac{33432566611171}{10813440} a1$$

$$+ \frac{1375627731000576809}{11800166400} a2 + \frac{1747691862519650015 5}{130903179264} \Big) z^{20} + \Big($$

$$- \frac{4470438348054055}{251953152} a1 - \frac{878186588324709744 37}{274943877120} a2$$

$$- \frac{884562730431319319106 1813}{4178047156715520} a3 - \frac{455247791563514213649708 99641}{8551853231252981760} a4$$

$$- \frac{16478892800741311173881624729573}{168948612046479959654 4} a5$$

$$- \frac{19063307735413742082272879180508 61}{29513978615106042861035520} b - \frac{51233679679264930881 25}{15250220384256} \Big) z^{18}$$

$$+ \Big(\frac{305447674546295285667}{6872442142720} a1 + \frac{255770418112644987519431}{499970165882880} a2$$

$$+ \frac{181623787723785126105232635 59}{7597546640756244480} a3$$

$$+ \frac{14374639032865019378915726572046 27}{2643681899672963434700 80} a4$$

$$+ \frac{541282850430715331890996264407969781}{583724963447790326381936 64} a5$$

$$+ \frac{26698621221852164654789793824610546608 29}{944583735851214306151249870848 00} b$$

$$+ \frac{178915049632227395933289 5}{3961668362043392} \Big) z^{16} + \Big(- \frac{109451450625453645 93}{171811053568} a1$$

$$- \frac{301384865220880537729 1}{5681479157760} a2 - \frac{304107959616476214991195525 13}{16144786611607019520} a3$$

$$- \frac{127020023157576240121509579700621}{330460237459120429337 60} a4$$

$$- \frac{23130313141936228294735446370041 29}{384029581215651530514432} a5$$

$$+ \frac{71370194020489610928901285201973348 9}{268347652230458609702059622400} b - \frac{77848015503389487587 5}{2380810313728} \Big) z^{14}$$

$$+ \Big(\frac{513488042940068391}{8925249536} a1 + \frac{88652776366110362893 1}{2380810313728} a2$$

$$+ \frac{225716300929743862990660 63}{21208258274689024} a3 + \frac{253434992200356500487786573025}{1302306354518701199 36} a4$$

$$+ \frac{1331512280287359532676274932003 05}{479248738462882041364 48} a5$$

$$- \frac{561917032906096826659805160739 7}{758084368114013410885632} b + \frac{242475899469323219699 25}{282976311574528} \Big) z^{12} + \Big($$

$$- \frac{225383917206511864801783933163 07}{23962436923144102068224} a5 + \frac{749638352471321659162408568382 1}{473802730071258381803520 0} b$$

$$- \frac{238141370976975297}{7012696064} a1 - \frac{152680654788199818459}{850289397760} a2$$

$$- \frac{329326639239584295797538 3}{7574377955246080} a3 - \frac{23436324332133119453067678179 1}{3255765886296752998 40} a4$$

$$+ \frac{756096103079672910187 5}{141488155787264} \Big) z^{10} + \Big(\frac{184292639699166795}{14025392128} a1$$

$$+ \frac{421845561014157967273}{7142430941184} a2 + \frac{808790618424027189419502 1}{63624774824067072} a3$$

$$+ \frac{330227415820018539023152153421}{169299826087431155916 8} a4$$

$$+\frac{63806617040633819090821136875505}{267010011429319994474496}\,a5$$

$$+\frac{676920458324243173173738817058933}{709566968554716552588951552}\,b-\frac{16464708738561641624875}{282976311574528}\Bigg)z^8+\Bigg($$

$$-\frac{11387389212027009}{3506348032}\,a1-\frac{3282254831762379697}{255086819328}\,a2$$

$$-\frac{44697803603492544761501}{1737651413262336}\,a3-\frac{2279303701056969816572036581}{60464223602653984256}\,a4$$

$$-\frac{29980950008170858136834248301 63}{66752502857329998618624}\,a5-\frac{3746895008679770792043133326193}{6521755225686733020119040}\,b$$

$$+\frac{3356186510151003017825}{141488155787264}\Bigg)z^6+\Bigg(\frac{94494167663162913}{196355489792}\,a1$$

$$+\frac{1082172505145883665 95}{61901068156928}\,a2+\frac{1855480018411308731657935}{551414715141914624}\,a3$$

$$+\frac{7423187202374441967629287887}{1539089328067555962880}\,a4+\frac{7160733649450152637736335907249}{124604672000349330754764 8}\,a5$$

$$+\frac{2598882359247937050122489762556551}{22913100026246055344018227200}\,b-\frac{36861939943061624031675}{7357384100937728}\Bigg)z^4+\Bigg($$

$$-\frac{25719496435759883}{687244214272}\,a1-\frac{256534421544757331321}{1949883646943232}\,a2$$

$$-\frac{7372217238428384451626057}{29630431898949353472}\,a3-\frac{4105840822828583854420463}{11728712626667452416}\,a4$$

$$-\frac{236705820763108496226859675}{562978206080037715968}\,a5-\frac{2109227265944384707794952658053}{261915758260131146694082560}\,b$$

$$+\frac{40622956697968527041525}{77252533059846144}\Bigg)z^2-\frac{741360679027511894125}{35655015258390528}$$

$$+\frac{378826698247003335935158321}{3934109615527586680012800}\,b+\frac{1507226706970563}{1374488428544}\,a1$$

$$+\frac{18430653601371694619}{4499731492945920}\,a2+\frac{135782927347988925149}{17998925971783680}\,a3$$

$$+\frac{63991156523290033369}{6374619615006720}\,a4+\frac{1302467800979520250097}{105702783434293248}\,a5\Bigg)s^2$$

$$+\Bigg(\frac{51271458371}{4992}\,a3+\frac{122524522018271}{3226704}\,a4+\frac{17609111060077855}{197904512}\,a5$$

$$+\frac{284271140813427576573881}{245951294452531200}\,b\Bigg)z^{26}+\Bigg(-\frac{12766477131}{5632}\,a2$$

$$-\frac{4005315498925333}{53489920}\,a3-\frac{36425345724174883567}{138298188160}\,a4$$

$$-\frac{764832294758381653983}{1272343331072}\,a5-\frac{3847036670639665943186413}{532403970156789760}\,b$$

$$-\frac{100536007406625}{37486592}\Bigg)z^{24}+\Bigg(\frac{1759608769009}{5406720}\,a1+\frac{8365464907227001}{536371200}\,a2$$

$$+\frac{22080204877869259288979}{89657664307200}\,a3+\frac{150387258998918505152849023}{183516163760793600}\,a4$$

$$+\frac{73527747308201247439822952393}{40520368958383226880}\,a5+\frac{3251766384302972914430029396661 21}{16392515262113934434304000}\,b$$

$$+\frac{1241998284337448995}{65451589632}\Bigg)z^{22}+\Bigg(\Bigg(-\frac{1794501042985}{1536}\,a3-\frac{4329499568235499}{992832}\,a4$$

$$-\frac{27201923821354555}{2647552}\,a5-\frac{16052260328624212085611477}{12108371419201536 0}\,b\Bigg)z^{18}+\Bigg($$

$$-\frac{446826699585}{8192}\,a2+\frac{94876519875510975}{15560704}\,a3+\frac{8458583614245120270 39}{40232200192}\,a4$$

$$+\frac{86017175202086624181105}{1850681208832}\,a5+\frac{1127966925225160425504759421 3}{17036927045017272320}\,b$$

$$-\frac{3518760259231875}{54525952}\Bigg)z^{16}+\Bigg(\frac{8798043845045}{180224}\,a1+\frac{4422821846178407}{39333888}\,a2$$

$$-\frac{78647285626689754608 13}{597717762048}\,a3-\frac{511200459478993205247285 89}{1223441091738624}\,a4$$

$$-\frac{11655773657996856649000541259 5}{1350678965279440896}\,a5$$

$$-\frac{870795363197188536929529292837060 9}{6994139845168612025303040}\,b-\frac{19644817830808323125}{43634393088}\Bigg)z^{14}+\Bigg($$

$$-\frac{1924276288789127}{10498048}\,a1-\frac{17071657418953109}{2082908160}\,a2$$

$$+\frac{26363280058054526207594 47}{174085298196480}\,a3+\frac{313549219038402199340274088 33}{712654435937748480}\,a4$$

$$+\frac{1126750480786117576486417360004 93}{1337509845367966347264}\,a5$$

$$+\frac{592076412015279952099578763400198405 3}{5540757585342574446445068288}\,b+\frac{621447222780038283212 5}{2541703397376}\Bigg)z^{12}$$

$$+\Bigg(-\frac{949425375501983725327786660963064 11}{20847320123135368799354 88}\,a5$$

$$-\frac{343963292135961015207684779410963102745 69}{10795242695442449213157141381120 0}\,b+\frac{6919477286762110781 1}{245444362240}\,a1$$

$$-\frac{24614808393274766398 61}{17856077352960}\,a2-\frac{268576339770943144255022312 9}{271340951455580160}\,a3$$

$$-\frac{245369558377158200925090512127113}{944172107026058369536 0}\,a4-\frac{25253404885338631473619 85}{565952623149056}\Bigg)z^{10}$$

$$+\Bigg(-\frac{55852525340475733 35}{24544436224}\,a1+\frac{40950383742889628847 1}{3571215470592}\,a2$$

$$+\frac{16211312115478411957622000 89}{461279617474486272}\,a3+\frac{153330792502414326210408352783 99}{1888344214052116739072}\,a4$$

$$+\frac{132418670686217023458308793876015 35}{10423660061567684399677 44}\,a5$$

$$-\frac{243573900460237798360976150711903175877}{196277139917135440239220752384 0}\,b$$

$$+\frac{47117246304724044440691 25}{1131905246298112}\Bigg)z^{8}+\Bigg(\frac{501548343088169071 5}{49088872448}\,a1$$

$$
\begin{aligned}
& - \frac{20339654796071356255}{1190405156864}\, a2 - \frac{53092849165342823956517 35}{10604129137344512}\, a3 \\
& - \frac{592925015425895583140189206 77}{6511531772593505996 8}\, a4 - \frac{274901787434244204914227642295 45}{2396243692314410206822 4}\, a5 \\
& + \frac{420551789298145245078150136547271 07}{36388049669472643722510336 0}\, b - \frac{1230992626394759777653875}{565952623149056} \Big) z^6 \\
& + \Big(- \frac{982193973454395}{39844864}\, a1 - \frac{1356348729547212375}{85028939776}\, a2 \\
& - \frac{2420506751382869073843}{47339862220288}\, a3 - \frac{32644871360020180959099795}{244794427541109248}\, a4 \\
& - \frac{480239720164792453274104875}{23255470616405378 56}\, a5 - \frac{467603794490415618985329854691007}{173276426997488779631001 60}\, b \\
& + \frac{1370421988366595957887 5}{21767408582656} \Big) z^4 + \Big(\frac{19155788655414855}{7012696064}\, a1 \\
& + \frac{3661443427546575023}{510173638656}\, a2 + \frac{10172585180313285595015 1}{4544626773147648}\, a3 \\
& + \frac{451987247310208154125978593 1}{1209284472053079685 12}\, a4 + \frac{605634595153209727630210089584 5}{133505005714659997237248}\, a5 \\
& + \frac{1703183027539230972106026550694483}{1621867356696494977346174976}\, b - \frac{5129486015862077093787 5}{565952623149056} \Big) z^2 \\
& + \frac{9568402530074439871 75}{205800953872384} + \frac{534598242998278793887450766736 97}{2796323028787060305769267 20}\, b \\
& - \frac{277007301162261}{3506348032}\, a1 - \frac{879970142715200851}{1020347277312}\, a2 - \frac{2637469126966677265727}{1737651413262336}\, a3 \\
& - \frac{331818056654494596395760 25}{219869904009650851 84}\, a4 - \frac{1328083826086289321494963575 89}{667525028573299986186 24}\, a5 \Big) s^8 \\
& + \Big(\Big(\Big(\frac{358900208597}{4096}\, a3 + \frac{378936554828825}{2647552}\, a4 + \frac{44251464738265995}{487149568}\, a5 \\
& + \frac{1022069308804772690750687}{201806190320025600}\, b \Big) z^4 + \Big(- \frac{1172920086410625}{299892736} \\
& - \frac{148942233195}{45056}\, a2 - \frac{1038322350768073}{42791936}\, a3 + \frac{3804862171817349097}{221277101056}\, a4 \\
& + \frac{887559459915167702145}{5089373324288}\, a5 + \frac{408123452305847253206934 29}{25555390567525908480}\, b \Big) z^2 \\
& - \frac{738571801409896710082296959 3}{8104073791676645376 0}\, a5 - \frac{1759608769009}{10813440}\, a1 \\
& + \frac{47762798999202889}{11800166400}\, a2 - \frac{581843482716245075897 9}{179315328614400}\, a3 \\
& - \frac{249433383668638729983779 3}{333665752292352 00}\, a4 - \frac{791389402859297530721042155692836 9}{2098241953550583607590912000}\, b \\
& + \frac{421358002847275014 5}{523612717056} \Big) s^{22} + \Big(\Big(\Big(- \frac{1870058981637}{165472}\, a4 - \frac{158955013439145}{3805856}\, a5 \\
& - \frac{1344206495679472701759}{4484582007111680}\, b \Big) z^2 + \frac{69222093296821581169 5}{20357493297152}\, a5
\end{aligned}
$$

$$- \frac{502680037033125}{599785472} - \frac{63832385655}{90112}\, a2 + \frac{222533432618006\,5}{171167744}\, a3$$

$$+ \frac{1231329707754113268\,1}{442554202112}\, a4 + \frac{1917068232365530062901322\,9}{170369270450172723\,20}\, b \Big) s^{24} + \Big($$

$$- \frac{3509672996629922\,5}{6332944384}\, a5 - \frac{358900208597}{159744}\, a3 - \frac{468699385947401}{103254528}\, a4$$

$$- \frac{150442148215187688186019}{983805177810124800}\, b \Big) s^{26} + \Big(- \frac{925865159238729}{876587008}\, a1$$

$$- \frac{21412470814855819}{3985731552}\, a2 - \frac{5731023813890145076\,87}{39492077574144}\, a3$$

$$- \frac{804681741310512078594221\,5}{290693382705067232}\, a4 - \frac{5455444272671342064230033779\,1}{1283701978025576896512}\, a5$$

$$- \frac{409237463739092286261021487156031}{1773917421386791381472378880}\, b + \frac{7526795048315526025}{4421504868352} \Big) z^{8} + \Big($$

$$- \frac{24539417449353105}{3506348032}\, a1 - \frac{621178647822755050\,33}{1275434096640}\, a2$$

$$- \frac{183531781559687020209311}{1032869721169920}\, a3 - \frac{6607982106484850084487203795\,9}{16278829431483764992\,0}\, a4$$

$$- \frac{23248441992708900150614058182\,99}{3267605034974195736576}\, a5$$

$$- \frac{1198169799510040123667588036900779}{34113796565130603489853440\,0}\, b - \frac{402479203354905517452\,5}{141488155787264} \Big) z^{12}$$

$$+ \Big(\frac{1938237239526587242839318241346\,93}{934535040002619980660736}\, a5$$

$$+ \frac{3416195925195439865901892826378\,81}{3547834842773582762944757\,76}\, b + \frac{23295299382861249}{7012696064}\, a1$$

$$+ \frac{696775891583890822\,03}{3571215470592}\, a2 + \frac{174988928299060651134581}{289203521927577\,6}\, a3$$

$$+ \frac{89817629365412101379471659\,3}{7113438070900468736}\, a4 + \frac{542303206803543681575}{141488155787264} \Big) z^{10} + \Big($$

$$- \frac{1638015759289308321}{171811053568}\, a1 - \frac{2233870314972993513\,9}{214763816960}\, a2$$

$$- \frac{2893453721624374045276977}{5043669669355520}\, a3 - \frac{529157243931832109109606775677}{34068065717435095808\,0}\, a4$$

$$- \frac{6943876355523366332906116387779}{227946330618474822860\,8}\, a5$$

$$- \frac{53249235247361776989833270199758839}{245908501471210638902231040\,0}\, b - \frac{2235665410632769535775}{20420970938368} \Big) z^{16}$$

$$+ \Big(\frac{3431213419501722153}{343622107136}\, a1 + \frac{7088879144834762911\,75}{8332836098048}\, a2$$

$$+ \frac{12663958954380707210588397}{33740410891550720}\, a3 + \frac{213303053170497797462922052604\,9}{227903612040772709888\,0}\, a4$$

$$+ \frac{2654795493379715284192150677394\,3}{1524882349654624677068\,8}\, a5$$

$$+ \frac{40680514694731342258147976273965 3}{39799429325985704071495680} b + \frac{7280308685646549419 0775}{990417090510848} \Big) z^{14} + \Big($$

$$- \frac{1834757898644975986 4807}{37982246879232} a3 - \frac{6460948260952220343277329037}{4275926615626490880} a4$$

$$- \frac{4714769061757142693963117829545}{145910164949232692 4288} a5$$

$$- \frac{960533711662662543532279499152152 79}{30555648448580373785542 65600} b - \frac{1306881707561911}{629882880} a1$$

$$- \frac{1320847554950165365}{27494387712} a2 - \frac{8899466495695080355 75}{15250220384256} \Big) z^{20}$$

$$+ \Big(\frac{200629843046284986 49}{3436221071360} a1 + \frac{6550448499026778806 7727}{749955248824320} a2$$

$$+ \frac{6519812241091056301778233 73}{1036029087375851520} a3 + \frac{7287942637131017714549815387399 63}{396552284950944515205120} a4$$

$$+ \frac{300508292645155973065956724988467 27}{795988586519714081429 9136} a5$$

$$+ \frac{45115503862406415268769915022554877897 53}{14168756037768214592268748062720 0} b$$

$$+ \frac{6044736548994746460011 95}{5942502543065088} \Big) z^{18} + \Big(\Big(- \frac{969030563 2119}{4096} a3$$

$$- \frac{1875875593664299 5}{2647552} a4 - \frac{699346843819319146 5}{487149568} a5$$

$$- \frac{124542554568939166806465 9}{5605727508889600} b \Big) z^{16} + \Big(\frac{140431248441}{1024} a2$$

$$+ \frac{6028285705637583}{607840} a3 + \frac{679880903645803616697}{25145125120} a4$$

$$+ \frac{1807334321501487019 77}{3614611736} a5 + \frac{836129279820110987992664 17}{106480794031357952} b$$

$$+ \frac{1105896081472875}{6815744} \Big) z^{14} + \Big(\frac{86220829681441}{1802240} a1 - \frac{13618967601100422 61}{1966694400} a2$$

$$- \frac{498983585330545347236029}{29885888102400} a3 - \frac{2478073242362841436012099673}{61172054586931200} a4$$

$$- \frac{3904417813623725541362231224 1}{5872517240345395 20} a5$$

$$- \frac{39274272932240016219915431282963543}{43713374032303825158144000} b - \frac{710972046172034671 15}{43634393088} \Big) z^{12}$$

$$+ \Big(\frac{41927601058870370759051985495 43}{1049027329700365762 56} a5$$

$$+ \frac{31577018459400932966770254548747551}{18618137047522091553914880 0} b - \frac{3028377280487367 9}{209960960} a1$$

$$+ \frac{1974116194059781032 1}{15274659840} a2 + \frac{32958428658598827666 28669}{232113730928640} a3$$

$$+ \frac{139151795146014776385001871 57}{475102957291832320} a4 + \frac{363825364972229326232 5}{847234465792} \Big) z^{10}$$

$$+\left(\frac{82703502798224525607}{490888724480}\,a1-\frac{41462056377226506258893}{35712154705920}\,a2\right.$$
$$-\frac{3318316016946733059436621877}{542681902911160320}\,a3-\frac{176752556784466592679162886808777}{18883442140521167390720}\,a4$$
$$-\frac{33146678059156264260900198239265823}{4169464024627073759870976}\,a5$$
$$+\frac{989021500255624645571009898873202693417}{2453464248964193002990259404800}\,b$$
$$\left.-\frac{2919051943170322691365285}{565952623149056}\right)z^8+\left(-\frac{2309221015468956693}{24544436224}\,a1\right.$$
$$+\frac{1538134605339932402771}{2976012892160}\,a2+\frac{44213021672027699241200249}{45223491909263360}\,a3$$
$$+\frac{415819717265600945210786715933}{4720860535130291847680}\,a4$$
$$-\frac{778150854817135831263741191920949}{347455335385589479989248}\,a5$$
$$\left.-\frac{63114041701906182435411997913316968539}{204455354080349416915854950400}\,b+\frac{915190999820106549474975}{282976311574528}\right)$$
$$z^6+\left(\frac{2376405017069952843}{98177744896}\,a1-\frac{1999149181527555465}{216437301248}\,a2\right.$$
$$+\frac{14497695712049072444440797}{106041291373445120}\,a3+\frac{25106480441236228583443831227}{38303128074079447040}\,a4$$
$$+\frac{3387845408416788609963630283479}{2819110226252247302144}\,a5$$
$$\left.+\frac{133153814743840659923012826086731}{1783727924974149202083840}\,b-\frac{599831483586497067063825}{565952623149056}\right)z^4+\left(\right.$$
$$-\frac{12814817315927445}{7012696064}\,a1-\frac{3046026012417071867}{850289397760}\,a2$$
$$-\frac{432895318931085903681059}{7574377955246080}\,a3-\frac{5353455821526691293427017609}{46510941232810757120}\,a4$$
$$-\frac{490733846943239516594787294713}{3423205274734871724032}\,a5-\frac{55679971403580795547304141771933}{32489330062029146180812800}\,b$$
$$\left.+\frac{11317299454965139312425}{70744077893632}\right)z^2-\frac{359327399995604954825}{51450238468096}$$
$$-\frac{2656313828911524889381703641408811}{2838267874218866210355806208}\,b-\frac{2069755363723197}{14025392128}\,a1$$
$$+\frac{15826727307276192089}{7142430941184}\,a2+\frac{209336079037464164739053}{63624774824067072}\,a3$$
$$\left.+\frac{2972405404955787146949146281}{1692998260874311559168}\,a4+\frac{5577970106882232774429739517191}{1869070080005239961321472}\,a5\right)$$
$$s^{10}+\left(\frac{21323194776506523}{98177744896}\,a1+\frac{90799434952641036403}{92851602235392}\,a2\right.$$
$$+\frac{176963631984259146444221}{75192915701170176}\,a3+\frac{34911050875756709153080545319}{8464991304371557795840}\,a4$$

$$+\frac{1007435159204263412896718850499}{16991546181865817830 1952}\,a5$$

$$+\frac{3118475334647738942848066816675567}{68739300078738166032054681600}\,b-\frac{35179293975049578436 75}{3678692050468864}\Big)z^{6}+\Big($$

$$-\frac{9248987245914887}{343622107136}\,a1-\frac{106859309023641110789}{974941823471616}\,a2$$

$$-\frac{17780033026170353563387153}{74076079747373383680}\,a3-\frac{11429692995443432595456367}{29321781566668631040}\,a4$$

$$-\frac{148809724524198634221287959}{281489103040018857984}\,a5-\frac{122554889549867219966617079997217}{20953260660810491735526604800}\,b$$

$$+\frac{15364232015135378953075}{77252533059846144}\Big)z^{4}+\Big(\Big(-\frac{2307215626695}{2048}\,a3$$

$$-\frac{1384513259367699}{1323776}\,a4+\frac{430551364278546855}{243574784}\,a5$$

$$-\frac{98770644409660343731139}{2242291003555840}\,b\Big)z^{12}+\Big(-\frac{5582875937463288675}{346829312}\,a5$$

$$-\frac{27711517157832819646650289}{146870060732907520}\,b+\frac{702156242205}{2048}\,a2+\frac{110195865204477}{67072}\,a3$$

$$-\frac{848759452567419435}{346829312}\,a4+\frac{5529480407364375}{13631488}\Big)z^{10}+\Big(\frac{1759608769009}{360448}\,a1$$

$$-\frac{364604068417589329}{393338880}\,a2+\frac{4875194000885141856419}{5977177620480}\,a3$$

$$+\frac{126560717097548540002970803}{12234410917386240}\,a4+\frac{81813617075804697214655933273}{2701357930558881792}\,a5$$

$$+\frac{4180896312690006670363124055231259}{6358308950153283659366400}\,b-\frac{125125729355019273475}{87268786176}\Big)z^{8}$$

$$+\Big(\frac{72092545107479}{20996096}\,a1+\frac{19973635636505438411}{22911989760}\,a2$$

$$-\frac{890820321999800152645051}{348170596392960}\,a3-\frac{686360048162772101855414827}{64786766903431680}\,a4$$

$$-\frac{59231167544731659666748647101009}{2675019690735932694528}\,a5$$

$$-\frac{773235355321997298773658998223179369}{1259263087577857828737515520}\,b+\frac{2030448772657972848125}{1270851698688}\Big)z^{6}$$

$$+\Big(-\frac{28668583075810854423}{1718110535680}\,a1-\frac{7800439585315394257283}{24998508294144}\,a2$$

$$+\frac{561650588066133438591682345}{379877332037812224}\,a3+\frac{278770139901567751897858937317841}{66092047491824085867520}\,a4$$

$$+\frac{95168236454327066895740355454729495}{14593124086194758159548416}\,a5$$

$$+\frac{2663650772042564277126229625911246394123}{13739399794199480816745452666880}\,b$$

$$-\frac{2490442587617451124725895}{3961668362043392}\Big)z^{4}+\Big(\frac{2008741008069247257}{171811053568}\,a1$$

$$
\begin{aligned}
&+\frac{45630944275474377727}{3124813536768}\,a2-\frac{850360539968236393889461531}{3228957322321403904}\,a3\\
&-\frac{836736279805846647341005546913}{1652301187295602146688}\,a4\\
&-\frac{3590673520689813429471396584050565}{7296562043097379079774208}\,a5\\
&+\frac{7819588904172621513330006176849219309}{6869699897099740408372726333440}\,b+\frac{50264100636911298036175}{3961668362043392}\Bigg)z^{2}\\
&+\frac{19871721033271735181325}{720303338553344}-\frac{32177514776470746911651313183504643}{5094326953726170121151447040}\,b\\
&-\frac{1635981727635399729}{687244214272}\,a1+\frac{145821368009061319301}{16665672196096}\,a2\\
&-\frac{342819627928247926638891}{148457807922823168}\,a3-\frac{26113621519019219220686211409}{9116144481630908039552}\,a4\\
&-\frac{10132540760533645153231725776885}{3354741169240174289555136}\,a5\Bigg)s^{14}+\Bigg(\Bigg(\frac{2409758543437}{6144}\,a3\\
&+\frac{3647088793307473}{3971328}\,a4+\frac{349007105072039345}{243574784}\,a5\\
&+\frac{9091927846951856361814897}{302709285480038400}\,b\Bigg)z^{6}+\Bigg(\frac{234584017282125}{54525952}+\frac{29788446639}{8192}\,a2\\
&-\frac{43520383564007903}{77803520}\,a3-\frac{212418124017339742397}{201161000960}\,a4\\
&-\frac{1931155723126670384253}{1850681208832}\,a5-\frac{884282586921482679556525921}{25555390567525908480}\,b\Bigg)z^{4}\\
&+\Bigg(\frac{18406018284744424285}{523612717056}-\frac{1759608769009}{1351680}\,a1+\frac{37282717590614153}{2950041600}\,a2\\
&+\frac{2819401084815413308021}{22414416076800}\,a3+\frac{8708032453466595890129629}{917580818803968 00}\,a4\\
&-\frac{2427483231317987799397580993}{10130092239595806720}\,a5\\
&-\frac{3936512888281227042898014949528751}{1049120976775291803795456000}\,b\Bigg)z^{2}\\
&+\frac{195134865955786403198476715801}{1395662447340486623232}\,a5+\frac{1171678995844123}{1259765760}\,a1\\
&-\frac{689395731004931443}{68735969280}\,a2+\frac{189806321944173096827237}{4178047156715520}\,a3\\
&+\frac{1545033012151733831638915}{13362270673832784}\,a4+\frac{6314266798317155147928610429460136577}{831113637801386166966760243200}\,b\\
&-\frac{9906163933918934675}{346595917824}\Bigg)s^{20}+\Bigg(\Bigg(\frac{256357291855}{2048}\,a3+\frac{321962014088347}{1323776}\,a4\\
&+\frac{66487460619122385}{243574784}\,a5+\frac{1667490871911651060772 37}{20180619032002560}\,b\Bigg)z^{22}+\Bigg(\\
&-\frac{9965490420951431}{15560704}\,a3-\frac{374558320840394531 81}{40232200192}\,a4
\end{aligned}
$$

$$-\frac{795935833362985945545}{1850681208832}\ a5-\frac{7300550427616682755784429}{1111103937718517760}\ b$$

$$-\frac{531936547125}{8192}\ a2-\frac{4189000308609375}{54525952}\Bigg)z^{20}+\Bigg(\frac{1759608769009}{135168}\ a1$$

$$+\frac{207330105992483159}{590008320}\ a2+\frac{3214614474691748473919}{2241441607680}\ a3$$

$$+\frac{22527956758207889637792787}{18351616376079360}\ a4-\frac{1599777727682255646581028427}{1013009223959580672}\ a5$$

$$-\frac{708269202058143884313542806592971}{9122791102393841772134400}\ b+\frac{186263268174065117525}{523612717056}\Bigg)z^{18}+\Bigg($$

$$-\frac{5570024798790699}{83984384}\ a1-\frac{6383381494492444163}{7637329920}\ a2$$

$$-\frac{877643893866534238094243}{464227461857280}\ a3-\frac{630714267175847240556083}{1855870926921220}\ a4$$

$$+\frac{20192437986190275424667135393063}{3566692920981243592704}\ a5$$

$$+\frac{4167084638170543318299200746828921003}{18469191951141914821483560960}\ b-\frac{65171774805570059375}{105904308224}\Bigg)z^{16}$$

$$+\Bigg(\frac{62440407902319398913}{429527633920}\ a1+\frac{898082972289254960059}{781203384192}\ a2$$

$$+\frac{161552517034906815116973101}{94969333009453056}\ a3-\frac{5804103391678773651970706669863}{8261505936478010733440}\ a4$$

$$-\frac{27766986514346301059268345016925125}{3648281021548689539887104}\ a5$$

$$-\frac{20828968293037679757362187851631630652061}{75566698868097144492099989667840}\ b$$

$$+\frac{1446671958747144315307105}{3961668362043392}\Bigg)z^{14}+\Bigg(-\frac{4393082025388131309}{24544436224}\ a1$$

$$-\frac{1204290788020035706821}{1190405156864}\ a2-\frac{10570269585489275440038327}{9044698381852672}\ a3$$

$$+\frac{1338320301746837349992619801825}{1888344214052116739072}\ a4$$

$$+\frac{1884197151606863889172892052746895}{347455335385589479989248}\ a5$$

$$+\frac{18938472872495894317062104376749231059}{109042855509519689021789306880}\ b+\frac{321803363380654691424675}{1131905246298112}\Bigg)$$

$$z^{12}+\Bigg(-\frac{12540029316220401072370984229745}{5990609230786025517056}\ a5$$

$$-\frac{407391644272778311131409751737423}{8086233259882809716113408}\ b+\frac{1662866289629272611}{12272218112}\ a1$$

$$+\frac{353844845058692842737}{595202578432}\ a2+\frac{1725016755146628251012433}{2651032284336128}\ a3$$

$$-\frac{5152530111806387262790434525}{32557658862967529984}\ a4-\frac{731874128405620734301275}{1131905246298112}\Bigg)z^{10}+\Bigg($$

$$- \frac{4545243314042833335}{7012696064} a1 - \frac{200606773265200556999}{85028939776} a2$$

$$- \frac{25338942001941438848213}{89110328885248} a3 - \frac{1927162027680550137472032 3}{174105127609452032} a4$$

$$+ \frac{822685364399250409930914368 7955}{23962436923144102068224} a5$$

$$- \frac{3764809431227232824274790554 51287}{36388049669472643722510 3360} b + \frac{280941527579647201435875}{565952623149056} \Big) z^8$$

$$+ \Big(\frac{339080713924302 75}{1753174016} a1 + \frac{2529729207097051747}{40581993984} a2$$

$$+ \frac{7040041084889340584164 63}{7953096853008384} a3 + \frac{17379720733022300196254518463}{21162478260928894489 6} a4$$

$$+ \frac{7397274405166958469854514422585}{23363376000065499516518 4} a5$$

$$+ \frac{26876829920283170041383429280519757}{5676535748437732420711612416} b - \frac{115756130794606552610125}{565952623149056} \Big) z^6$$

$$+ \Big(- \frac{11941403814351531}{3506348032} a1 - \frac{3564410403668323145}{340115759104} a2$$

$$- \frac{10107867897883393472035}{579217137754112} a3 - \frac{52499963134414084780533222 27}{24185689441061593702 4} a4$$

$$- \frac{474286634224844157954892321489}{22250834285776666206208} a5$$

$$- \frac{15058263072113768304986927467 34527}{1351556130580412481121812480} b + \frac{1063620539137013324549 25}{2263810492596224} \Big) z^4$$

$$+ \Big(\frac{173435132577519}{557828096} a1 + \frac{1205090923908456957}{1190405156864} a2$$

$$+ \frac{321222973183090502986 89}{17231709848184832} a3 + \frac{21301634739403576260635875 23}{846499130437155779584} a4$$

$$+ \frac{1115384268802193049066103537 95}{38938960000109165860864} a5$$

$$+ \frac{72995669265845769195758231571589 69}{977625601119831694678111027 20} b - \frac{124766603740587370835 25}{2263810492596224} \Big) z^2$$

$$+ \frac{3451868120802421551025}{14045915101790208} + \frac{905365216578305831396295791948 9}{1341008682291871471073702707 2} b$$

$$- \frac{7221521943930109}{687244214272} a1 - \frac{1304231227156141513}{30466931983488} a2$$

$$- \frac{2301096331903161337639879}{2963043189894935347 2} a3 - \frac{28802507033057158359772 3}{293217815666686310 4} a4$$

$$- \frac{672460867769277156411384 05}{5629782060800377159 68} a5 \Big) s^4 + \Big(\Big(\frac{4358073961535}{6144} a3$$

$$+ \frac{8914262765713979}{3971328} a4 + \frac{115670154278309831 5}{243574784} a5$$

$$+ \frac{211299410263903052481679 7}{30270928548003840} b \Big) z^8 + \Big(\frac{63832385655}{1024} a2$$

$$- \frac{1820695552306555}{972544} a3 - \frac{2463450494518659 3197}{5029025024} a4$$

$$- \frac{965096445514472199645}{115667575552} a5 - \frac{185230152679824780759288857}{1064807940313579520} b$$

$$+ \frac{502680037033125}{6815744} \Big) z^6 + \Big(- \frac{8798043845045}{2162688} a1 - \frac{2492431574972105 9}{472006656} a2$$

$$+ \frac{1035408791583557938 7437}{7172613144576} a3 + \frac{4524462327883519998335 4353}{14681293100863488} a4$$

$$+ \frac{6069416013444299999553149 6915}{1620814758335329 0752} a5 + \frac{2159122548755560019610548857 40593}{2098241953550583607590912} b$$

$$- \frac{876309402742146625}{32725794816} \Big) z^4 + \Big(\frac{1586269509494657}{251953152} a1 - \frac{421151782861555141 3}{274943877120} a2$$

$$- \frac{116130293926250451123 0397}{4178047156715520} a3 - \frac{311224981319848551120128 6729}{8551853231252981760} a4$$

$$+ \frac{101872196243073443192604055657}{3210023628883119233 4336} a5$$

$$+ \frac{11026826934769836786692789963 64923}{2187141152108910965702 00064} b - \frac{14907644761098938091 25}{15250220384256} \Big) z^2$$

$$+ \frac{27449401981723187855}{527565921792} - \frac{115582097053716849514055396067525 33506237}{113350048302145716738149984 5017600} b$$

$$- \frac{15560074327760692717}{6872442142720} a1 + \frac{2139168588482091719 7941}{1499910497648640} a2$$

$$- \frac{874483319639523134496757051}{2279263992226873 3440} a3 - \frac{89911458292749239059914703284727}{79310456990188903041 0240} a4$$

$$- \frac{2357817044591568262186767165394 1009}{1751174890343370979145 80992} a5 \Big) s^{18} + \Big(\Big(\frac{413536672533340215}{60893696} a5$$

$$+ \frac{2148119877504056559832829}{3363436505333760 0} b + \frac{153814375113}{512} a3 + \frac{742653967514577}{330944} a4 \Big) z^{10}$$

$$+ \Big(\frac{829821013515}{4096} a2 - \frac{16468046590130049}{7780352} a3 - \frac{172089012723082892805}{20116100096} a4$$

$$- \frac{1857999148920819146 4975}{925340604416} a5 - \frac{2835560089360964059966604029}{8518463522508636160} b$$

$$+ \frac{502680037033125}{2097152} \Big) z^8 + \Big(- \frac{1759608769009}{360448} a1 - \frac{147933279170976971}{393338880} a2$$

$$+ \frac{20159276707884042409261}{5977177620480} a3 + \frac{1260927296333472776020 12457}{12234410917386240} a4$$

$$+ \frac{51832397071260300623603773687}{27013579305588817 92} a5$$

$$+ \frac{13816088158465180804150089496215 43}{317915447507664182968 3200} b - \frac{216015560091970360 75}{43634393088} \Big) z^6$$

$$+ \Big(\frac{621885770475801}{41992192} a1 + \frac{2909575949238003491}{15274659840} a2$$

$$- \frac{4534271707789586880519 91}{232113730928640} a3 - \frac{22531236626411803375966438 97}{4751029572918323 20} a4$$

$$- \frac{510957711233730347952076398403}{77536802630027034624} a5$$

$$- \frac{1479826638786594338918512197738 15533}{839508725051905219158343680} b + \frac{319477912542103779625}{1694468931584} \Big) z^{4} + \Big($$

$$- \frac{42177313006414272219}{3436221071360} a1 - \frac{147489879812878853}{22725916631040} a2$$

$$+ \frac{1303035301022345162304776033}{3798773320378122240} a3 + \frac{15012629119554750881259454898137}{26436818996729634347008} a4$$

$$+ \frac{10787691087413162715137393098672387}{29186248172389516319096832} a5$$

$$- \frac{54258791537606109925421938211226594953}{13739399794199480816745452666880} b + \frac{411160405094665555321345}{3961668362043392} \Big)$$

$$z^{2} - \frac{380805896776736778 22725}{720303338553344} + \frac{9498033799320969229606931043710923849}{995608680739092812807641497600} b$$

$$+ \frac{260482626239896827}{85905526784} a1 - \frac{548713515513568894217}{41664180490240} a2$$

$$+ \frac{49710971527112899313246471}{2690797768601169920} a3 + \frac{4784658107840368701398404833189}{66092047491824085867520} a4$$

$$+ \frac{100825636684078351928534262007097}{1216093673849563179962368} a5 \Big) s^{16} + \Big(\Big(- \frac{666528958823}{4096} a3$$

$$- \frac{2840949580838675}{2647552} a4 - \frac{1535484271671819705}{487149568} a5$$

$$- \frac{6218792452464311538476633}{201806190320025600} b \Big) z^{20} + \Big(- \frac{446826699585}{4096} a2$$

$$+ \frac{4814950906920833}{3890176} a3 + \frac{135476586374365543195}{20116100096} a4$$

$$+ \frac{8410801442894751937575}{462670302208} a5 + \frac{89690659581647254792704077}{370367979239505920} b$$

$$- \frac{3518760259231875}{27262976} \Big) z^{18} + \Big(\frac{22874913997117}{720896} a1 + \frac{385520586457001723}{786677760} a2$$

$$- \frac{43300099921552503461593}{11954355240960} a3 - \frac{424464657127374173293546241}{24468821834772480} a4$$

$$- \frac{235918754315323826187695768731}{5402715861117763584} a5$$

$$- \frac{8538648358103981283663327185170193}{12716617900306567318732800} b + \frac{4357994778375607775}{13425967104} \Big) z^{16} + \Big($$

$$- \frac{268751034878807}{1908736} a1 - \frac{21829934865422273101}{22911989760} a2$$

$$+ \frac{1920347974527178691317001}{348170596392960} a3 + \frac{17159249368166016857144894767}{712654435937748480} a4$$

$$+ \frac{15250397127996689851250293580 0059}{2675019690735932694528} a5$$

$$+ \frac{12594337706470262808351277489387209833}{13851893963356436116112670720} b + \frac{214147026749673208625}{1270851698688} \Big) z^{14}$$

$$+\left(\frac{64691862890753301879}{245444362240}\,a1+\frac{18931827434171382225463}{17856077352960}\,a2\right.$$

$$-\frac{1300331587461926993451698813}{271340951455580160}\,a3-\frac{3367166807560797799671135043415}{171667655822919703552}\,a4$$

$$-\frac{3985532867698879334285923479009809}{90640522274501603475456}\,a5$$

$$-\frac{13832361859024369473802711789571220633817}{21590485390884898426314282762240}\,b$$

$$\left.-\frac{874140787346154354014915}{565952623149056}\right)z^{12}+\left(\frac{7009097969050061799147172544937677}{347455335385589479989248}\,a5\right.$$

$$+\frac{323885726542578245409970773712680076703}{1635642832642795335326839603200}\,b-\frac{6627183039007160643}{24544436224}\,a1$$

$$-\frac{1123430145097053059989}{1488006446080}\,a2+\frac{1807260071485641448463075231}{768799362457477120}\,a3$$

$$\left.+\frac{22215254118463503835166079836853}{2360430267565145923840}\,a4+\frac{1381830306769108975119225}{565952623149056}\right)z^{10}$$

$$+\left(\frac{16101258695076841455}{98177744896}\,a1+\frac{867859591092065332525}{2380810313728}\,a2\right.$$

$$-\frac{11360683571112618014671339}{21208258274689024}\,a3-\frac{314851082031228325890195649225}{130230635451870119936}\,a4$$

$$-\frac{245140030750281871092531664916725}{47924873846288204136448}\,a5$$

$$\left.+\frac{3804503693563665282796235797768847}{242586997796484291483402240}\,b-\frac{2202102564585423222070875}{1131905246298112}\right)z^{8}$$

$$+\left(-\frac{209130156910752285}{3506348032}\,a1-\frac{31157622682743233143}{255086819328}\,a2\right.$$

$$-\frac{56597354881853550767389}{2272313386573824}\,a3+\frac{6178182569700065664493170199}{32557658862967529984}\,a4$$

$$+\frac{18422292593376710937258721563595}{35943655384716153102336}\,a5$$

$$\left.-\frac{535664921581420278958968552926449}{18821405001451367442677760}\,b+\frac{124836076600698806926625}{141488155787264}\right)z^{6}$$

$$+\left(\frac{86971931440275405}{7012696064}\,a1+\frac{14155388459291225917}{510173638656}\,a2\right.$$

$$+\frac{186336353746923622618609}{4544626773147648}\,a3+\frac{5920151225165287018622457155}{120928447205307968512}\,a4$$

$$+\frac{5670728362627393698910819896155}{1335050057146599997237248}\,a5$$

$$\left.+\frac{10914613710917796177877442136143629}{1621867356696494977346174976}\,b-\frac{127999759248938211068875}{565952623149056}\right)z^{4}$$

$$+\left(-\frac{4534482540441699}{3506348032}\,a1-\frac{1962887277636870581}{510173638656}\,a2\right.$$

$$-\frac{1036905692172380866043}{133665493327872}\,a3-\frac{1320588746183824218487987417}{120928447205307968512}\,a4$$

$$- \frac{831745589599902707998779423041}{66752502857329998618624}\, a5$$

$$- \frac{1492519589038923708568660061834453}{40546683917412374433654374 40}\, b + \frac{335210157995523586767 25}{1131905246298112} \Bigg) z^{2}$$

$$- \frac{3899296388232431700775}{2675412400340992} - \frac{189990937220632287760884066013726831}{8798630410078485252102999244800}\, b$$

$$+ \frac{9201388557136821}{196355489792}\, a1 + \frac{44941302491236258313}{185703204470784}\, a2$$

$$+ \frac{725018962457054680703105}{1654244145425743872}\, a3 + \frac{8713543490645505235746886797}{16929982608743115591680}\, a4$$

$$+ \frac{2380754433248509525983175517407}{3738140160010479922642944}\, a5 \Bigg) s^{6} + \Bigg(\Bigg(- \frac{2512301460179}{1024}\, a3$$

$$- \frac{3759630800930879}{661888}\, a4 - \frac{1062571196815652685}{121787392}\, a5$$

$$- \frac{1174073622117466360460443}{6306443447500800}\, b \Bigg) z^{14} + \Bigg(\frac{1340480098755}{4096}\, a2$$

$$+ \frac{60321871599756837}{7780352}\, a3 + \frac{300128695244989073547}{20116100096}\, a4$$

$$+ \frac{15682225680549076799115}{925340604416}\, a5 + \frac{374771368958712826252784289}{1064807940313579520}\, b$$

$$+ \frac{10556280777695625}{27262976} \Bigg) z^{12} + \Bigg(- \frac{8368191307212892009102962049}{3376697413198602240}\, a5$$

$$+ \frac{142261703336143757282901919018133}{1150351948218521714688000}\, b + \frac{12317261383063}{450560}\, a1$$

$$- \frac{72369961338393131}{61459200}\, a2 - \frac{66296990992539405351547}{7471472025600}\, a3$$

$$- \frac{11243190715949897462257829}{955813352920800}\, a4 - \frac{182251252957994826935}{87268786176} \Bigg) z^{10} + \Bigg($$

$$- \frac{2397485779801385}{41992192}\, a1 + \frac{18195167978789131687}{11455994880}\, a2$$

$$+ \frac{2714935419714206113157677}{696341192785920}\, a3 - \frac{77411704739266303232207717}{178163608984437120}\, a4$$

$$- \frac{80983106610590312304689778513577}{5350039381471865389056}\, a5$$

$$- \frac{4337315768265632693442144847944 13273}{629631543788928914368757760}\, b + \frac{4696500141569953600625}{1270851698688} \Bigg) z^{8}$$

$$+ \Bigg(\frac{102338863110631953}{2789140480}\, a1 - \frac{1467272272033347562809}{1488006446080}\, a2$$

$$+ \frac{1678712701180345126338087}{11305872977315840}\, a3 + \frac{114525991749306308015853259052 31}{2360430267565145923840}\, a4$$

$$+ \frac{1107253648800010302363170763326433}{86863833846397369997312}\, a5$$

$$+ \frac{36853065676042165016760075486276847069}{68151784693449805638618316800}\, b - \frac{127812592047621034001655}{43534817165312} \Bigg)$$

$$z^6 + \left(-\frac{75120001849927359}{24544436224} a1 + \frac{1548535850967898511393}{5952025784320} a2 - \frac{46828905160455169951858363 3}{768799362457477120} a3 - \frac{2075543475047774486276624999750 1}{944172107026058369536 0} a4 - \frac{130129496960225878272868105283025 1}{347455335385589479989248} a5 - \frac{5902475595931517930915611868575827066 7}{4089107081606988338317099008 00} b + \frac{121890674912286293908612 5}{1131905246298112}\right)$$

$$z^4 + \left(-\frac{105480840243176931}{24544436224} a1 - \frac{275139212788805201 3}{297601289216} a2 + \frac{729936113070231830077871}{5302064568672256} a3 + \frac{4263987600830222616089647 47}{14798935846803422 72} a4 + \frac{400530640582106282387613367838 5}{119812184615720510341 12} a5 + \frac{5407772455960997168396344526212 9}{4548506208684080465313792 0} b - \frac{162892328416367045357175}{1131905246298112}\right) z^2$$

$$- \frac{932300231875155613 75}{51450238468096} + \frac{5732820560549493628205524711162 79}{1949359803721748770848768 00} b + \frac{7252803516555753}{7012696064} a1 - \frac{6004878991356975061}{1275434096640} a2 - \frac{834860373273635862994 19}{22723133865738240} a3 + \frac{111403041509482356881400301}{23255470616405378560} a4 + \frac{3359727438685213350347954628 7}{10269615824204615172096} a5\Big) s^{12} + \Big(\frac{1201509860803993}{687244214272} a1 + \frac{14883326485019037601}{2249865746472960} a2 + \frac{120828811643849292871}{8999462985891840} a3 + \frac{64985582808910867451}{3187309807503360} a4 + \frac{1534453474207518123107 3}{581365308888612864} a5 + \frac{7152840559388495257724491 79}{196705480776379334000640 0} b - \frac{33499858455097654947 5}{17827507629195264}\Big) z^2 + \frac{12556958705391105}{20420970938368} + \ln(2)\, b - \frac{20141325181838867}{37970242838528} a5 - \frac{293459009195161}{697982405120} a4 - \frac{3386254663753}{78095933440} a1 - \frac{33434784709309}{214763816960} a2 - \frac{3332039966024447}{11167718481920} a3 - \frac{19761869671979192089 1}{224005447625895936 00} b\Big) \sin(2 \arctan(s \sin(\phi), s \cos(\phi)))$$

$$- \frac{23774031225945}{21889024} \cos(2 \arctan(s \sin(\phi), s \cos(\phi))) \Big(s^{18} + (9 z^2 - 5) s^{16} + \Big(36 z^4 - 40 z^2 + \frac{460}{43}\Big) s^{14} + \Big(84 z^6 - 140 z^4 + \frac{3220}{43} z^2 - \frac{22540}{1763}\Big) s^{12} + \Big(126 z^8 - 280 z^6 + \frac{9660}{43} z^4 - \frac{135240}{1763} z^2 + \frac{214130}{22919}\Big) s^{10} + \Big(126 z^{10} - 350 z^8 + \frac{16100}{43} z^6 - \frac{338100}{1763} z^4 + \frac{1070650}{22919} z^2 - \frac{3640210}{848003}\Big) s^8 + \Big(84 z^{12} - 280 z^{10} + \frac{16100}{43} z^8$$

$$- \frac{450800}{1763} z^6 + \frac{2141300}{22919} z^4 - \frac{14560840}{848003} z^2 + \frac{1040060}{848003} \Big) s^6 + \Big(36 z^{14} - 140 z^{12} + \frac{9660}{43} z^{10} - \frac{338100}{1763} z^8 + \frac{2141300}{22919} z^6 - \frac{21841260}{848003} z^4 + \frac{3120180}{848003} z^2 - \frac{148580}{717541} \Big) s^4 + \Big(9 z^{16} - 40 z^{14} + \frac{3220}{43} z^{12} - \frac{135240}{1763} z^{10} + \frac{1070650}{22919} z^8 - \frac{14560840}{848003} z^6 + \frac{3120180}{848003} z^4 - \frac{297160}{717541} z^2 + \frac{37145}{2022161} \Big) s^2 - \frac{2188905411753745}{4754806245189} + \frac{37145}{2022161} z^2 - \frac{148580}{717541} z^4 + \frac{1040060}{848003} z^6 - \frac{3640210}{848003} z^8 + \frac{214130}{22919} z^{10} - \frac{22540}{1763} z^{12} + \frac{460}{43} z^{14} - 5 z^{16} + z^{18} \Big) \Big) z \sqrt{5} \sqrt{13} s^2 e_z$$

```
> SetCoordinates(cylindrical[s, phi, z]);
```

$$cylindrical_{s, \phi, z} \qquad (58)$$

```
> #Calculate taylor constraint
> Taylor_integral := int(int(s·CrossProduct(Curl(B_proj_cyl), B_proj_cyl)[2], z=-sqrt(1-s^2)..sqrt(1 - s^2)), phi=0..2·Pi) :
> #numerical solution to enforce Taylor constraint being zero for B_nplusone
> numerical_approx := eval(chebsum) + b·s·ln(s) : # numerical expansion for geostrophic flow
> #Minimise the squared Residual of the ODE
> Residual := simplify(subs(ug(s) = numerical_approx, Taylor_integral)) :
> Int_Res := int( Residual^2, s=0..1) :
> Int_Res2 := collect(Int_Res, seq( a||i, i=1..N), factor) :
> Min_Int_Res2 := evalf(minimize(Int_Res2, seq( a||i, i=1..N), b, location)) :
> Solutionarray := {op(op(op(Min_Int_Res2[2]))[1])} :
> vars := [seq( a||i,i=1..N),b]:
> Solutionarray2 := evalf(subs(Solutionarray, vars)) :
> #calculate constant so the angular momentum is zero
```

```
> numerical_app := numerical_approx + s·Const :
> ang_mom := evalf(int(int(int(expand(numerical_app * s^2), phi = 0 .. 2 * Pi), z = -sqrt(1-s
      ^2) .. sqrt(1-s^2)), s = 0 .. 1)) :

> for i from 1 to N do a || i := (Solutionarray2[i]) end do:
> b := rhs(Solutionarray[M]) :

> C_value := solve(ang_mom = 0):

> numerical_solution := subs(Const = C_value, numerical_app) :
> #Plot geostrophic flow solution
> plot([numerical_solution], s = 0 ..1);
```

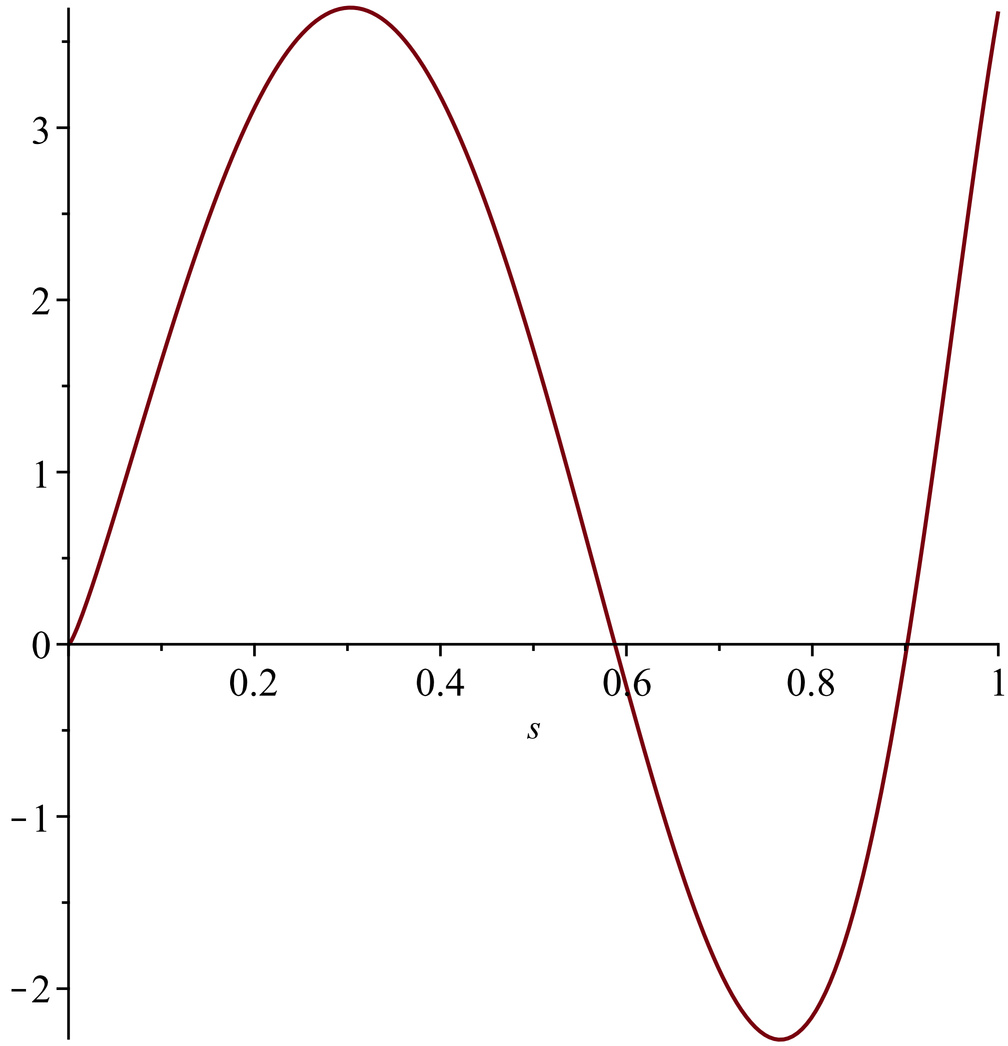

Supplement: ESM5 - ESM8 are PDF versions of the maple worksheets ESM1 - ESM4 respectively. [file rspa20180412supp5.pdf]
